# Supplementary material for: 2-Oxabicyclo[2.2.2]octane as a new bioisostere of the phenyl ring
Source: Nat Commun. 2023 Oct 2;14:5608. doi: 10.1038/s41467-023-41298-3 (PMC10545790; doi:10.1038/s41467-023-41298-3)
Supplement: Supplementary file 1 — Supplementary Information [file 41467_2023_41298_MOESM1_ESM.pdf]

Supplementary Information

2-Oxabicyclo[2.2.2]octane as a new bioisostere of the phenyl ring

Vadym V. Levterov,[1] Yaroslav Panasiuk,[1] Kateryna Sahun,[1] Oleksandr Stashkevych,[1] Valentyn Badlo,[1] Oleh Shablykin,[1,2] Iryna Sadkova,[1] Lina Bortnichuk,[1] Oleksii Klymenko-Ulianov,[1] Yuliia Holota,[1] Leonid Lachmann,[3] Petro Borysko,[1] Kateryna Horbatok,[1] Iryna Bodenchuk,[1] Julia P. Bas,[4] Dmytro Dudenko,[1] Pavel K. Mykhailiuk[1]*

[1] Enamine Ltd. Winston Churchill street 78, 02094 Kyiv (Ukraine), E-mail: Pavel.Mykhailiuk@gmail.com
[2] V.P. Kukhar Institute of Bioorganic Chemistry and Petrochemistry NAS of Ukraine, 02094 Kyiv (Ukraine).
[3] Bienta, Winston Churchill street 78, 02094 Kyiv (Ukraine).
[4] Taras Shevchenko National University of Kyiv, Chemistry Department, Volodymyrska 64, 01601 Kyiv (Ukraine).

Contents

Design of the "Ideal" Bioisoster of the *para*-substituted Phenyl Ring 5
Experimental Section. Data description and procedures 6
Synthesis of Imatinib analogues 52
Copies of ^{1}H, $^{13}C\{^{1}H\}$ and $^{19}F\{^{1}H\}$ NMR spectra 58
Compound 8 58
Compound SI-1 60
Compound 5 62
Compound 6 64
Compound SI-2 66
Compound 9 68
Compound SI-3 70
Compound 10 72
Compound 11 74
Compound 12 76
Compound 13 78
Compound 14 80
Compound 15 83
Compound 16 86
Compound 17 89
Compound 18 91
Compound 19 93
Compound 20 95
Compound 21 97

Compound 22 99
Compound 23 101
Compound 24 103
Compound 25 105
Compound 26 108
Compound 27 110
Compound SI-4 112
Compound 30 114
Compound SI-5 116
Compound 31 118
Compound SI-6 120
Compound SI-7 122
Compound 32 124
Compound SI-8 126
Compound SI-9 128
Compound 33 130
Compound SI-10 132
Compound 34 134
Compound 35 136
Compound SI-11 138
Compound 36 140
Compound 37 142
Compound 39 144
Compound 40 146
Compound SI-12 148
Compound SI-13 150
Compound SI-14 152
Compound 42 154
Compound 43 156
Compound 44 158
Compound 45 160
Compound 46 162
Compound SI-15 164
Compound 47 166
Compound 48 168
Compound 49 170

Compound 50 172
Compound 51 174
Compound SI-16 176
Compound 52 178
Compound SI-17 180
Compound 53 182
Compound 54 184
Compound SI-18 186
Compound 55 188
Compound 56 190
Compound 57 192
Compound SI-19 194
Compound 58 196
Compound 59 198
Compound 60 200
Compound 61 202
Compound 62 204
Compound 63 206
Compound 64 208
Compound SI-20 210
Compound 65 212
Compound SI-21 214
Compound 66 216
Compound SI-22 218
Compound 67 220
Compound 68 222
Compound SI-23 224
Compound 69 226
Compound 70 228
Compound 71 230
Compound 72 233
Compound 74 235
Compound 75 237
Compound 76 239
Compound 77 241
Compound 78 243

Compound 79 245
Compound 85 247
Compound SI-24 249
Compound SI-25 250
Compound SI-26 252
Compound 86 254
Compound SI-27 256
Compound 88 258
Compound SI-28 260
Compound 89 262
Crystallographic data (X-Ray) 264
Superposition of f 2-oxabicyclo[2.2.2]octane and *para*-substituted phenyl ring 277
Analysis of Aqueous Solubility...278
Determination of Distribution Coefficient (LogD, pH 7.4)...282
Assessment of Metabolic Stability in Human Liver Microsomes 285
Determination of pKa values 291
Biological activity of *Imatinib* and its analogues 85, 86. ABL1 Kinase Assay..294
Biological activity of Vorinostat and its analogues 88, 89 296
Generation and properties of virtual libraries based on *para*-aminobenzoic acid and 2-oxabicyclo[2.2.2]octane analog 301
Supplementary references..305

Design of the "Ideal" Bioisoster of the *para*-substituted Phenyl Ring

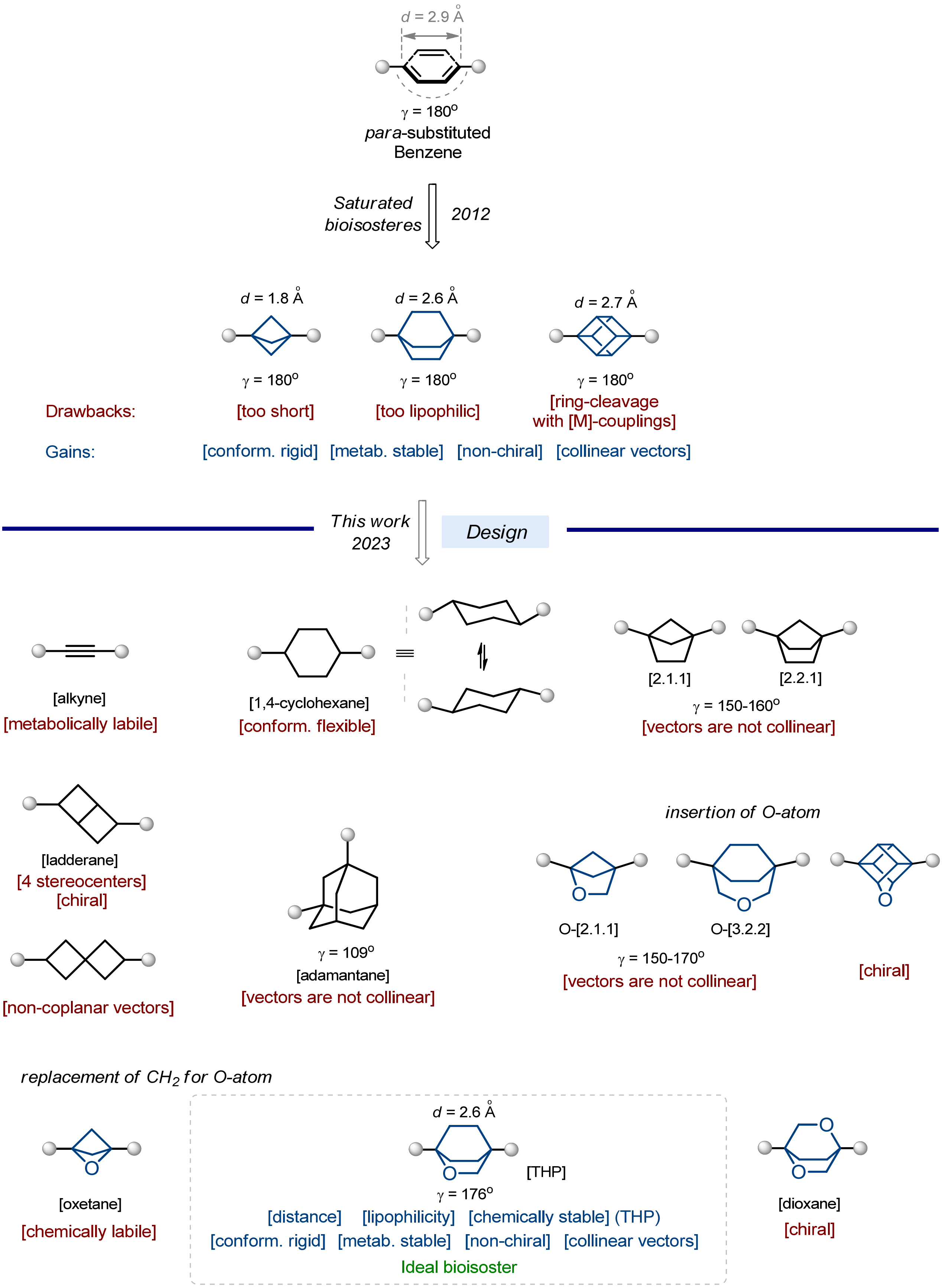

Supplementary Fig. 1. Design of 2-oxabicyclo[2.2.2]heptanes as the bioisostere of the *para*-substituted phenyl ring.

Experimental Section. Data description and procedures

General Considerations. All chemicals were provided by Enamine Ltd. (www.enamine.net). All solvents were treated according to standard methods. All reactions were monitored by thin-layer chromatography (TLC) and were visualized using UV light or TLC stains. Product purification was performed using silica gel column chromatography. TLC-characterization was performed with pre-coated silica gel GF254 (0.2 mm), while column chromatography characterization was performed with silica gel (100-200 mesh). ^{1}H-NMR spectra were recorded at 400 or 500 MHz (Varian); ^{19}F-NMR spectra were recorded at 376 MHz (Varian), ^{13}C NMR spectra were recorded at 126 or 151 MHz (Varian); ^{31}P-NMR spectra were recorded at 202 MHz (Varian) ^{1}H-NMR chemical shifts are calibrated using residual undeuterated solvents $CHCl_3$ (δ = 7.26 ppm), DMSO (δ = 2.50 ppm), D_2O (δ = 4.79 ppm) and CD_3CN (δ = 1.94 ppm). ^{13}C-NMR chemical shifts for ^{13}C-NMR are reported relative to the central $CHCl_3$ (δ = 77.16 ppm), DMSO (δ = 39.52 ppm) and CD_3CN (δ = 1.32 ppm). Coupling constants are given in Hz. High-resolution mass spectra (HRMS) were recorded on an Agilent LC/MSD TOF mass spectrometer by electrospray ionization time of flight reflectron experiments.

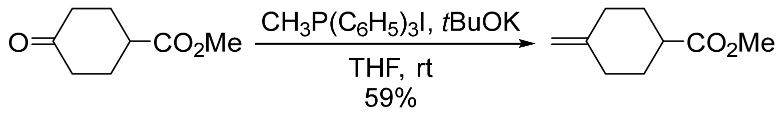

Methyl 4-methylenecyclohexane-1-carboxylate (8)

To a suspension of $CH_3P(C_6H_5)_3I$ (727.20 g, 1.80 mol, 1.20 equiv) in THF (3500 mL) was added *t*BuOK (201.60 g, 1.80 mol, 1.20 equiv) with vigorous stirring at room temperature. After 2 h, the mixture was cooled to 0 ºC (ice bath) and methyl 4-oxocyclohexane-1-carboxylate (234.00 g, 1.50 mol, 1.00 equiv) was added dropwise during ca. 1 h at 0-5 °C. The mixture was stirred for 48 h at room temperature and monitored with ^{1}H NMR. After consumption of the starting material, the mixture was concentrated under reduced pressure. The residue was diluted with water (1500 mL) and extracted with hexane (4 × 500 mL). The combined organic layers were dried over Na_2SO_4, filtered and concentrated under reduced pressure. Yield: 136.30 g, 0.88 mol, 59%, colorless oil. In some cases the final product was purified by distillation (b.p. = 63-64 °C, 4 mmHg). ^{1}H NMR (500 MHz, $CDCl_3$): δ 4.64 (s, 2H), 3.66 (s, 3H), 2.45 (tt, *J* = 11.0, 3.6 Hz, 1H), 2.33 (dt, *J* = 13.1, 3.6 Hz, 2H), 2.10 – 1.92 (m, 4H), 1.63 – 1.51 (m, 2H) ppm. ^{13}C{^{1}H} NMR (126 MHz, $CDCl_3$): δ 176.0, 147.7, 108.1, 51.7, 42.6, 33.8, 30.3 ppm. GCMS (M): 154. HRMS (ESI-TOF) *m/z*: $[M + H]^+$ calcd for $C_9H_{15}O_2$, 155.1072; found 155.1067.

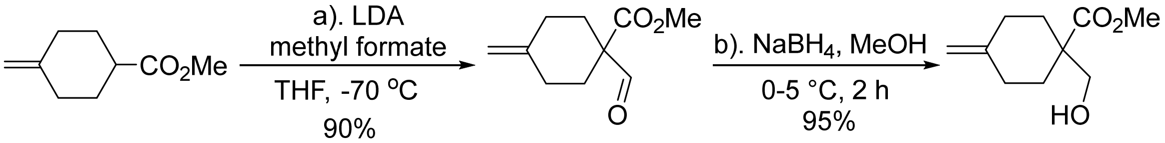

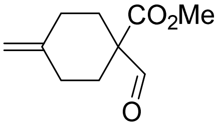

a). **Methyl 1-formyl-4-methylenecyclohexane-1-carboxylate (SI-1)**.

To a solution of DIPEA (361.20 g, 2.80 mol, 1.20 equiv) in THF (2000 mL) was added a solution of *n*BuLi (2.5M in hexane, 1000 mL, 2.60 mol, 1.13 equiv) dropwise at -10 – -20 °C under Ar. After addition, the mixture was cooled to -78 °C and methyl 4-methylenecyclohexane-1-carboxylate (354.20 g, 2.30 mol, 1.00 equiv) was added dropwise at -70 – -80 °C. The mixture was stirred for 45 min at the same temperature, and methyl formate (414.00 g, 6.90 mol, 3.00 equiv) was added dropwise during ca. 20 min at -70 – -80 °C. The resulting mixture was stirred overnight at room temperature and neutralized with a sat. aq. solution of NH_4Cl (600 mL). The mixture was partially concentrated under a reduced pressure and extracted with MeO*t*Bu (3 × 800 mL). The combined organic layers were dried over Na_2SO_4, filtered and concentrated under reduced pressure. Yield: 376.74 g, 2.07 mol, 90%, colorless oil. The crude product was immediatelly used for the next step without purification. ^{1}H NMR (500 MHz, $CDCl_3$): δ 9.55 (s, 1H), 4.68 (s, 2H), 3.76 (s, 3H), 2.23 – 2.10 (m, 6H), 2.00 – 1.94 (m, 2H) ppm. $^{13}C\{^1H\}$ NMR (126 MHz, $CDCl_3$): δ 198.9, 171.4, 146.2, 108.8, 59.2, 52.8, 30.9, 29.9 ppm. GCMS (M): 182. HRMS (ESI-TOF) *m/z*: $[M + H]^+$ calcd for $C_{10}H_{15}O_3$, 183.1021; found 183.1015.

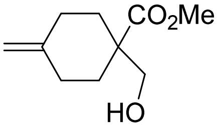

b). **Methyl 1-(hydroxymethyl)-4-methylenecyclohexane-1-carboxylate (5)**

To a solution of methyl 1-formyl-4-methylenecyclohexane-1-carboxylate (364.00 g, 2.00 mol, 1.00 equiv) in MeOH (3000 mL) was added $NaBH_4$ (26.60 g, 0.70 mol, 0.35 equiv) in portions at 0-5 °C. The mixture was stirred for 2 h and concentrated under reduced pressure. The residue was dissolved in MeO*t*Bu (1200 mL), washed with brine (2 × 300 mL) and dried over Na_2SO_4, filtered and concentrated. Yield: 349.60 g, 1.90 mol, 95%, colorless oil. The crude product was used for the next step without purification. ^{1}H NMR (500 MHz, $CDCl_3$): δ 4.65 (s, 2H), 3.74 (s, 3H), 3.65 (s, 2H), 2.22 – 2.10 (m, 6H), 1.47 – 1.41 (m, 2H) ppm. $^{13}C\{^1H\}$ NMR (151 MHz, $CDCl_3$): δ 176.5, 147.9, 107.9, 68.6, 52.2, 48.9, 31.9, 31.2 ppm. GCMS (M): 184. HRMS (ESI-TOF) *m/z*: $[M + H]^+$ calcd for $C_{10}H_{17}O_3$, 185.1178; found 185.1172.

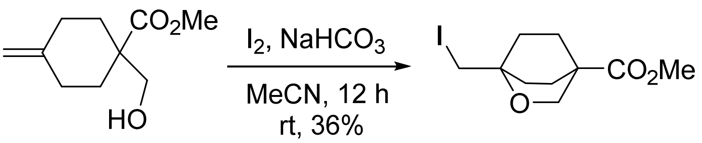

Methyl 1-(iodomethyl)-2-oxabicyclo[2.2.2]octane-4-carboxylate (6)

To a solution of methyl 1-(hydroxymethyl)-4-methylenecyclohexane-1-carboxylate (222.64 g, 1.21 mol, 1.00 equiv) in MeCN (4000 mL) were added $NaHCO_3$ (243.94 g, 2.90 mol, 2.40 equiv) in one portion and I_2 (736.60 g, 2.90 mol, 2.40 equiv) in four portions. The resulting mixture was stirred for 12 h at room temperature. Then sodium thiosulfate pentahydrate (900.24 g, 3.63 mol, 3.00 equiv) and distilled water (2000 mL) were added to the mixture. The colorless mixture was extracted with MeO*t*Bu (10 × 400 mL). The combined organic layers were concentrated under reduced pressure to dryiness. The residue was dissolved in MeO*t*Bu (1000 mL), washed with brine (1 × 400 mL), a sat. solution of $Na_2S_2O_3$ (3 × 400 mL), dried over Na_2SO_4, filtered through a plug of SiO_2 (0.5 L glass filter filed with 3 cm in high with silica gel) and concentrated. The final product was purified by column chromatography (SiO_2, hexane/EtOAc, 1:5, R_f = 0.70). Yield: 135.16 g, 0.436 mol, 36%, white solid, m.p. = 51-52 °C. ^{1}H NMR (400 MHz, $CDCl_3$): δ 4.00 (s, 2H), 3.67 (s, 3H), 3.16 (s, 2H), 2.09 – 1.88 (m, 6H), 1.81 – 1.68 (m, 2H) ppm. ^{13}C{^{1}H} NMR (126 MHz, $CDCl_3$): δ 174.9, 70.4, 69.5, 52.1, 39.6, 30.3, 27.7, 15.6 ppm. LCMS $(M+H)^+$: 311. HRMS (ESI-TOF) *m/z*: $[M + H]^+$ calcd for $C_{10}H_{16}IO_3$, 311.0144; found 311.0148.

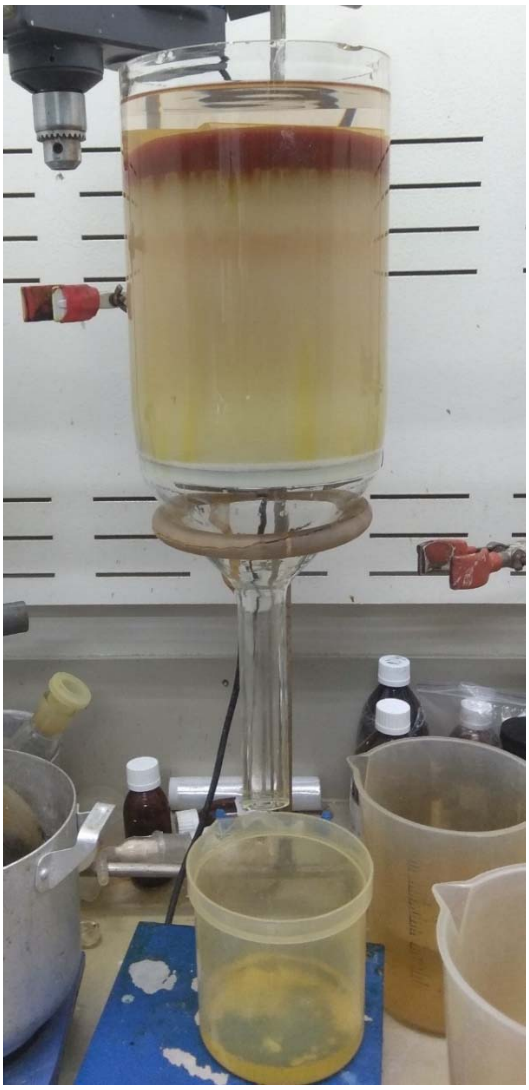

Supplementary Fig. 2. Purification of the final product **6** by column chromatography.

General procedure A for synthesis of 9-27 (compound **9** as an example)

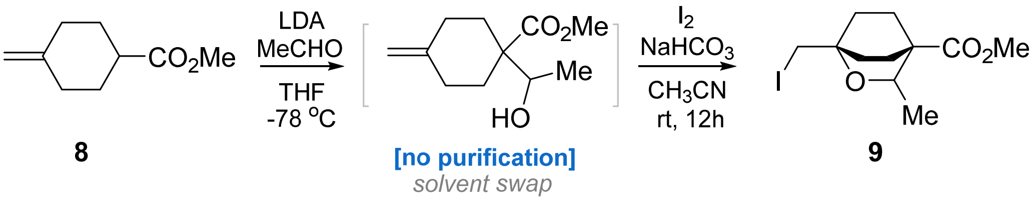

8 **9**

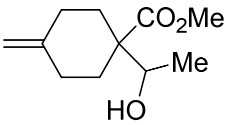

Methyl 1-(1-hydroxyethyl)-4-methylidenecyclohexane-1-carboxylate (SI-2)

To a solution of DIPEA (135.45 g, 1.05 mol, 1.05 equiv) in THF (1000 mL) was added a solution of *n*BuLi (2.5M in hexane, 500 mL, 1.15 mol, 1.15 equiv) dropwise (10 min) at -10 – -20 °C under Ar. After addition, the mixture was cooled to -78 °C and methyl 4-methylenecyclohexane-1-carboxylate **8** (154.00 g, 1.00 mol, 1.00 equiv) was added dropwise at -70 – -80 °C. The mixture was stirred for 45 min at the same temperature, and acetaldehyde (88.00 g, 2.00 mol, 2.00 equiv) was added dropwise at -70 – -80 °C. The resulting mixture was stirred for 2 h at -50 – -60 °C and neutralized with a sat. solution of NH_4Cl (300 mL). The mixture was partially concentrated and extracted with MeO*t*Bu (3 × 300 mL). The combined organic layers were dried over Na_2SO_4, filtered, and concentrated under reduced pressure. Yield: 168.00 g, 0.85 mol, 85%, colorless oil. The crude product (ca. 168 g, colorless oil) was directly used for the next step without purification. ^{1}H NMR (500 MHz, $CDCl_3$): δ 4.63 (s, 2H), 3.75 (s, 3H), 3.71 – 3.66 (m, 1H), 2.32 – 2.15 (m, 5H), 2.10 – 1.93 (m, 2H), 1.47 – 1.31 (m, 2H), 1.13 (d, *J* = 6.4 Hz, 3H) ppm. $^{13}C\{^1H\}$ NMR (151 MHz, $CDCl_3$): δ 175.8, 148.1, 107.6, 73.3, 52.4, 52.0, 32.4, 31.8, 31.7, 31.6, 18.9 ppm. GCMS (M): 198. HRMS (ESI-TOF) *m/z*: $[M + H]^+$ calcd for $C_{11}H_{19}O_3$, 199.1334; found 199.1325.

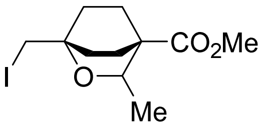

Methyl 1-(iodomethyl)-3-methyl-2-oxabicyclo[2.2.2]octane-4-carboxylate (9)

To a solution of methyl 1-(1-hydroxyethyl)-4-methylidenecyclohexane-1-carboxylate (168 g, 0.85 mol, 1.00 equiv) obtained in a previous step in MeCN (3000 mL) were added $NaHCO_3$ (181.44 g, 2.16 mol, 2.40 equiv) in one portion and I_2 (548.64 g, 2.16 mol, 2.40 equiv) in portions (~ 4). The resulting mixture was stirred for 12 h at room temperature. Then sodium thiosulfate pentahydrate (669.60 g, 2.70 mol, 3.00 equiv) and distilled water (1500 mL) were added to the mixture. The colorless mixture was extracted with MeO*t*Bu (10 × 200 mL). The combined organic layers were concentrated under reduced pressure. The residue was dissolved in MeO*t*Bu (500 mL), washed with

brine (1 × 200 mL), a sat. solution of $Na_2S_2O_3$ (3 × 200 mL), dried over Na_2SO_4, filtered through SiO_2 (hight: 2-3 sm, 0.5 L filter) and concentrated. The residue was purified by column chromatography (SiO_2, hexane/EtOAc, 1:5, R_f = 0.8). Yield: 162.1 g, 0.50 mol, 50% total yield, yellow oil. ^{1}H NMR (500 MHz, $CDCl_3$): δ 4.22 (q, J = 6.2 Hz, 1H), 3.66 (s, 3H), 3.14 (s, 2H), 2.20 – 2.08 (m, 2H), 2.06 – 1.95 (m, 1H), 1.87 – 1.63 (m, 5H), 1.13 (d, J = 6.3 Hz, 3H) ppm. $^{13}C\{^1H\}$ NMR (151 MHz, $CDCl_3$): δ 175.0, 74.7, 70.3, 52.0, 43.0, 30.3, 30.1, 30.0, 21.6, 18.4, 16.0 ppm. GCMS (M): 324. HRMS (ESI-TOF) *m/z*: $[M + H]^+$ calcd for $C_{11}H_{18}IO_3$, 325.0301; found 325.0290.

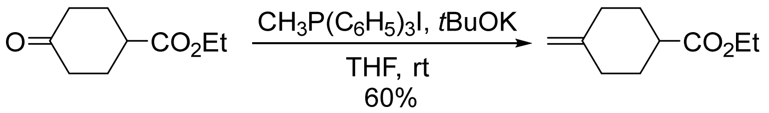

Ethyl 4-methylenecyclohexane-1-carboxylate

To a suspension of $CH_3P(C_6H_5)_3I$ (727.20 g, 1.80 mol, 1.20 equiv) in THF (3500 mL) was added *t*BuOK (201.60 g, 1.80 mol, 1.20 equiv) with vigorous stirring at room temperature. After 2 h, the mixture was cooled to 0 ºC (ice bath) and ethyl 4-oxocyclohexane-1-carboxylate (255.00 g, 1.50 mol, 1.00 equiv) was added dropwise during ca. 1 h at 0-5 °C. The mixture was stirred for 48 h at room temperature and monitored with ^{1}H NMR. After consumption of the starting material, the mixture was concentrated under reduced pressure. The residue was diluted with water (1500 mL) and extracted with hexane (4 × 500 mL). The combined organic layers were dried over Na_2SO_4, filtered and concentrated under reduced pressure. Yield: 151.20 g, 0.90 mol, 60%, colorless oil. In some cases the final product was purified by distillation (b.p. = 73-75 °C, 4 mmHg). ^{1}H NMR (500 MHz, $CDCl_3$): δ 4.64 (s, 2H), 4.12 (q, J = 7.1 Hz, 2H), 2.43 (tt, J = 11.0, 3.6 Hz, 1H), 2.33 (dt, J = 13.3, 3.8 Hz, 2H), 2.11 – 1.94 (m, 4H), 1.61 – 1.50 (m, 2H), 1.24 (t, J = 7.1 Hz, 1H) ppm. $^{13}C\{^1H\}$ NMR (126 MHz, $CDCl_3$): δ 175.6, 147.8, 108.0, 60.4, 42.8, 33.8, 30.3, 14.4 ppm. GCMS (M): 168. HRMS (ESI-TOF) *m/z*: $[M + H]^+$ calcd for $C_{10}H_{17}O_2$, 169.1229; found 169.1221.

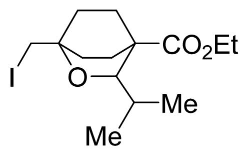

Ethyl 1-(iodomethyl)-3-isopropyl-2-oxabicyclo[2.2.2]octane-4-carboxylate (10)

General procedure A. Yield: 2.25 g, 0.0060 mol, 66%, yellow oil. R_f = 0.86 (hexane/EtOAc, 4:1). ^{1}H NMR (400 MHz, $CDCl_3$): δ 4.20 – 4.05 (m, 2H), 3.77 (d, J = 8.8 Hz, 1H), 3.18 (q, J = 13.8 Hz, 2H), 2.27 – 2.08 (m, 2H), 1.99 – 1.88 (m, 2H), 1.87 – 1.70 (m, 4H), 1.62 – 1.54 (m, 1H), 1.26 (t, J = 7.1 Hz, 3H), 1.09 (d, J = 6.5 Hz, 3H), 0.77 (d, J = 6.8 Hz, 3H) ppm. $^{13}C\{^1H\}$ NMR (126 MHz, $CDCl_3$): δ 175.5, 84.3, 69.8, 60.6, 41.9, 33.0, 32.2, 30.5, 30.0, 21.9, 19.9, 19.6, 16.6, 14.1 ppm. GCMS (M): 366. HRMS (ESI-TOF) *m/z*: $[M + H]^+$ calcd for $C_{14}H_{24}IO_3$, 367.0770; found 367.0757.

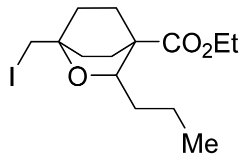

Ethyl 1-(iodomethyl)-3-propyl-2-oxabicyclo[2.2.2]octane-4-carboxylate (11)

General procedure A. Yield: 1.61 g, 0.0044 mol, 65%, yellow oil. R_f = 0.85 (hexane/EtOAc, 4:1). ^{1}H NMR (400 MHz, $CDCl_3$): δ 4.18 – 4.06 (m, 2H), 4.02 (d, J = 9.6 Hz, 1H), 3.14 (d, J = 3.8 Hz, 2H), 2.16 – 1.73 (m, 7H), 1.67 – 1.30 (m, 5H), 1.23 (t, J = 7.1 Hz, 3H), 0.91 (t, J = 7.3 Hz, 3H) ppm. $^{13}C\{^1H\}$ NMR (126 MHz, $CDCl_3$): δ 174.7, 78.4, 69.9, 60.7, 42.7, 35.5, 30.7, 30.18, 30.15, 22.1, 19.2, 16.4, 14.3 ppm. GCMS (M): 366. HRMS (ESI-TOF) *m/z*: $[M + H]^+$ calcd for $C_{14}H_{24}IO_3$, 367.0770; found 367.0756.

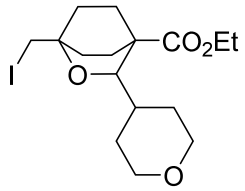

Ethyl 1-(iodomethyl)-3-(tetrahydro-2*H*-pyran-4-yl)-2-oxabicyclo[2.2.2]octane-4-carboxylate (12)

General procedure A. Yield: 0.10 g, 0.00025 mol, 51%, yellow oil. R_f = 0.74 (hexane/EtOAc, 4:1). ^{1}H NMR (400 MHz, $CDCl_3$): δ 4.19 – 3.97 (m, 3H), 3.93 – 3.79 (m, 2H), 3.44 – 3.28 (m, 2H), 3.16 (q, J = 15.1 Hz, 2H), 2.17 – 2.10 (m, 2H), 1.97 – 1.69 (m, 7H), 1.65 – 1.53 (m, 2H), 1.40 – 1.30 (m, 2H), 1.25 (t, J = 7.1 Hz, 3H) ppm. $^{13}C\{^1H\}$ NMR (151 MHz, $CDCl_3$): δ 175.3, 82.4, 69.8, 68.3, 67.5, 60.8, 41.5, 40.1, 32.1, 30.5, 29.9, 29.7, 29.4, 28.0, 21.9, 16.5, 14.2 ppm. HRMS (ESI-TOF) *m/z*: $[M + H]^+$ calcd for $C_{16}H_{26}IO_4$, 409.0876; found 409.0871.

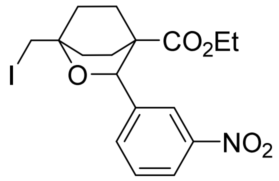

Ethyl 1-(iodomethyl)-3-(3-nitrophenyl)-2-oxabicyclo[2.2.2]octane-4-carboxylate (13)

General procedure A. Yield: 0.51 g, 0.0011 mol, 53%, colorless oil. R_f = 0.70 (hexane/EtOAc, 4:1). ^{1}H NMR (400 MHz, $CDCl_3$): δ 8.25 (s, 1H), 8.13 (d, J = 7.6 Hz, 1H), 7.75 (d, J = 7.7 Hz, 1H), 7.50 (t, J = 8.0 Hz, 1H), 5.34 (s, 1H), 4.03 (q, J = 7.1 Hz, 2H), 3.31 (q, J = 10.6 Hz, 2H), 2.55 – 2.40 (m, 1H), 2.27 – 2.14 (m, 2H), 2.00 – 1.61 (m, 5H), 1.17 (t, J = 7.1 Hz, 3H) ppm. $^{13}C\{^1H\}$ NMR (151 MHz, $CDCl_3$): δ 173.2, 148.2, 142.2, 133.4, 129.1, 123.0, 122.3, 79.9, 71.0, 61.2, 44.8, 30.5, 30.0,

29.9, 21.1, 15.3, 14.1 ppm. HRMS (ESI-TOF) *m/z*: $[M + H]^+$ calcd for $C_{17}H_{24}IN_2O_5$, 463.0730; found 463.0724.

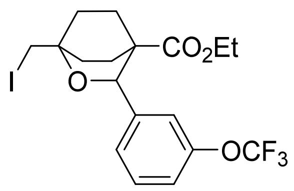

Ethyl 1-(iodomethyl)-3-(3-(trifluoromethoxy)phenyl)-2-oxabicyclo[2.2.2]octane-4-carboxylate (14)

General procedure A. Yield: 0.58 g, 0.0012 mol, 67%, colorless oil. R_f = 0.73 (hexane/EtOAc, 4:1). ^{1}H NMR (400 MHz, DMSO-d_6): δ 7.48 (t, *J* = 8.2 Hz, 1H), 7.37 – 7.24 (m, 3H), 5.14 (s, 1H), 4.00 – 3.79 (m, 2H), 3.41 (q, *J* = 10.5 Hz, 2H), 2.36 – 1.55 (m, 8H), 1.04 (t, *J* = 7.1 Hz, 3H) ppm. ^{13}C{^{1}H} NMR (151 MHz, DMSO-d_6): δ 172.6, 148.1, 142.5, 129.9, 125.8, 120.2, 120.1 (q, *J* = 256.1 Hz), 119.3, 79.1, 70.3, 60.2, 44.1, 29.5, 29.0, 28.9, 20.5, 17.4, 13.6 ppm. ^{19}F{^{1}H} NMR (376 MHz, DMSO-d_6): δ -57.2 (s) ppm. HRMS (ESI-TOF) *m/z*: $[M + H]^+$ calcd for $C_{18}H_{21}F_3IO_4$, 485.0437; found 485.0431.

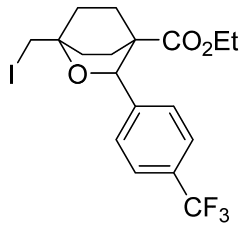

Ethyl 1-(iodomethyl)-3-(4-(trifluoromethyl)phenyl)-2-oxabicyclo[2.2.2]octane-4-carboxylate (15)

General procedure A. Yield: 0.42 g, 0.0009 mol, 96%, yellow oil. R_f = 0.73 (hexane/EtOAc, 4:1). ^{1}H NMR (400 MHz, DMSO-d_6): δ 7.71 (d, *J* = 8.1 Hz, 2H), 7.52 (d, *J* = 8.0 Hz, 2H), 5.17 (s, 1H), 3.99 – 3.83 (m, 2H), 3.41 (d, *J* = 2.6 Hz, 1H), 2.38 – 2.27 (m, 1H), 2.11 – 2.02 (m, 2H), 1.94 – 1.67 (m, 5H), 1.01 (t, *J* = 7.1 Hz, 3H) ppm. ^{13}C{^{1}H} NMR (151 MHz, DMSO-d_6): δ 172.5, 144.3, 128.3 (q, *J* = 31.7 Hz), 127.7, 124.7 (q, *J* = 3.6 Hz), 124.2 (q, *J* = 272.0 Hz), 79.3, 70.3, 60.3, 44.1, 29.6, 29.0, 28.9, 20.5, 17.3, 13.7 ppm. ^{19}F{^{1}H} NMR (376 MHz, DMSO-d_6): δ -61.4 ppm. HRMS (ESI-TOF) *m/z*: $[M + H]^+$ calcd for $C_{18}H_{21}F_3IO_3$, 469.0487; found 469.0481.

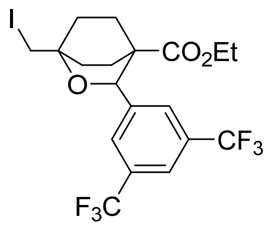

Ethyl 3-(3,5-bis(trifluoromethyl)phenyl)-1-(iodomethyl)-2-oxabicyclo[2.2.2]octane-4-carboxylate (16)

General procedure A. Yield: 0.94 g, 0.00175 mol, 43%, yellow oil. R_f = 0.66 (hexane/EtOAc, 4:1). ^{1}H NMR (400 MHz, DMSO-d_6): δ 8.09 (s, 1H), 7.94 (s, 2H), 5.35 (s, 1H), 3.92 (q, *J* = 7.1 Hz, 1H), 3.46 (q, *J* = 12.1 Hz, 1H), 2.33 (t, *J* = 12.3 Hz, 1H), 2.09 (t, *J* = 8.7 Hz, 2H), 2.00 – 1.82 (m, 3H), 1.75 – 1.62 (m, 3H), 1.05 (t, *J* = 7.1 Hz, 3H) ppm. ^{13}C{^{1}H} NMR (126 MHz, DMSO-d_6): δ 172.5, 143.2, 130.0 (q, *J* = 32.7 Hz), 127.4, 123.3 (q, *J* = 272.7 Hz), 121.7 (m,), 78.4, 70.5, 60.5, 44.2, 29.4, 28.9, 28.8, 20.4, 17.3, 13.5 ppm. ^{19}F{^{1}H} NMR (376 MHz, DMSO-d_6): δ -61.9 (s) ppm. HRMS (ESI-TOF) *m/z*: $[M + H]^+$ calcd for $C_{19}H_{20}F_6IO_3$, 537.0361; found 537.0352.

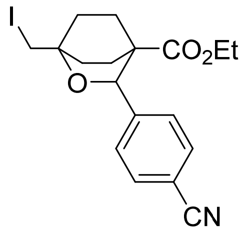

Ethyl 3-(4-cyanophenyl)-1-(iodomethyl)-2-oxabicyclo[2.2.2]octane-4-carboxylate (17)

General procedure A. Yield: 0.23 g, 0.00054 mol, 61%, yellow oil. R_f = 0.69 (hexane/EtOAc, 4:1). ^{1}H NMR (400 MHz, DMSO-d_6): δ 7.82 (d, *J* = 7.8 Hz, 2H), 7.49 (d, *J* = 7.8 Hz, 2H), 5.16 (s, 1H), 3.92 (q, *J* = 7.0 Hz, 1H), 3.41 (d, *J* = 3.7 Hz, 1H), 2.32 (t, *J* = 11.4 Hz, 1H), 2.10 – 2.00 (m, 2H), 1.95 – 1.66 (m, 5H), 1.04 (t, *J* = 7.1 Hz, 3H) ppm. ^{13}C{^{1}H} NMR (126 MHz, DMSO-d_6): δ 172.5, 145.2, 131.8, 127.9, 118.7, 110.5, 79.3, 70.4, 60.4, 44.1, 29.6, 29.0, 28.8, 20.5, 17.2, 13.8 ppm. HRMS (ESI-TOF) *m/z*: $[M + H]^+$ calcd for $C_{18}H_{21}INO_3$, 426.0566; found 426.0559.

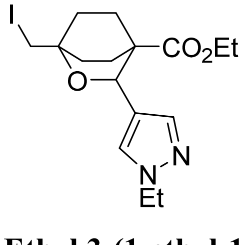

Ethyl 3-(1-ethyl-1*H*-pyrazol-4-yl)-1-(iodomethyl)-2-oxabicyclo[2.2.2]octane-4-carboxylate (18)

General procedure A. Yield: 0.27 g, 0.00065 mol, 39%, yellow oil. R_f = 0.70 (hexane/EtOAc, 4:1). ^{1}H NMR (400 MHz, DMSO-d_6): δ 7.54 (s, 1H), 7.26 (s, 1H), 5.05 (s, 1H), 4.07 (q, *J* = 7.2 Hz, 2H), 4.04 – 3.90 (m, 2H), 3.32 (s, 2H), 2.12 (t, *J* = 11.4 Hz, 1H), 2.02 – 1.60 (m, 7H), 1.32 (t, *J* = 7.2 Hz,

3H), 1.10 (t, J = 7.1 Hz, 3H) ppm. $^{13}C\{^1H\}$ NMR (126 MHz, DMSO-d$_6$): δ 173.2, 136.7, 127.3, 120.6, 73.5, 69.9, 60.1, 46.1, 43.7, 29.5, 29.2, 29.0, 20.8, 17.7, 15.5, 13.9 ppm. LCMS $(M+H)^+$: 419. HRMS (ESI-TOF) *m/z*: $[M + H]^+$ calcd for $C_{16}H_{24}IN_2O_3$, 419.0821; found 419.0821.

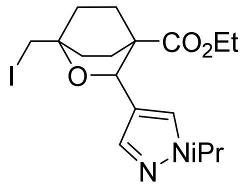

Ethyl 1-(iodomethyl)-3-(1-isopropyl-1*H*-pyrazol-4-yl)-2-oxabicyclo[2.2.2]octane-4-carboxylate (19)

General procedure A. Yield: 1.51 g, 0.0035 mol, 42%, yellow oil. ^1H NMR (400 MHz, DMSO-d$_6$): δ 7.55 (s, 1H), 7.27 (s, 1H), 5.05 (s, 1H), 4.43 (p, J = 6.6 Hz, 1H), 4.05 – 3.93 (m, 2H), 3.33 (s, 2H), 2.12 (t, J = 11.5 Hz, 1H), 1.98 – 1.57 (m, 7H), 1.36 (d, J = 6.6 Hz, 6H), 1.09 (t, J = 7.1 Hz, 3H) ppm. $^{13}C\{^1H\}$ NMR (126 MHz, DMSO-d$_6$): δ 173.2, 136.3, 125.6, 120.3, 73.6, 69.9, 60.1, 52.7, 43.7, 29.5, 29.1, 29.0, 22.7, 22.6, 20.9, 17.7, 13.9 ppm. LCMS $(M+H)^+$: 433. HRMS (ESI-TOF) *m/z*: $[M + H]^+$ calcd for $C_{17}H_{26}IN_2O_3$, 433.0988; found 433.0984.

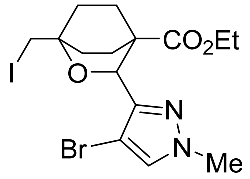

Ethyl 3-(4-bromo-1-methyl-1*H*-pyrazol-3-yl)-1-(iodomethyl)-2-oxabicyclo[2.2.2]octane-4-carboxylate (20)

General procedure A. Yield: 0.23 g, 0.00048 mol, 40%, yellow solid, m.p. = 102-103 °C. R_f = 0.68 (hexane/EtOAc, 4:1). ^1H NMR (400 MHz, DMSO-d$_6$): δ 7.83 (s, 1H), 5.04 (s, 1H), 3.93 – 3.81 (m, 2H), 3.79 (s, 3H), 3.26 (q, J = 10.5 Hz, 2H), 2.68 (t, J = 11.5 Hz, 1H), 2.13 – 1.71 (m, 7H), 0.98 (t, J = 7.1 Hz, 3H) ppm. $^{13}C\{^1H\}$ NMR (126 MHz, DMSO-d$_6$): δ 172.8, 147.3, 131.0, 92.8, 72.0, 70.5, 59.9, 42.4, 30.1, 29.7, 29.1, 22.1, 17.0, 13.7 ppm. HRMS (ESI-TOF) *m/z*: $[M + H]^+$ calcd for $C_{15}H_{21}BrIN_2O_3$, 482.9780; found 482.9769.

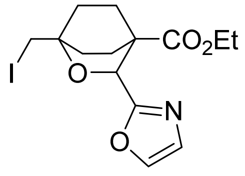

Ethyl 1-(iodomethyl)-3-(oxazol-2-yl)-2-oxabicyclo[2.2.2]octane-4-carboxylate (21)

General procedure A. Yield: 0.137 g, 0.00035 mol, 55%, yellow solid, m.p. = 44-45 °C. R_f = 0.75 (hexane/EtOAc, 4:1). ^1H NMR (400 MHz, DMSO-d$_6$): δ 8.09 (s, 1H), 7.19 (s, 1H), 5.13 (s, 1H),

4.01 – 3.87 (m, 2H), 3.32 (s, 2H), 2.37 (t, J = 11.8 Hz, 1H), 2.12 – 1.72 (m, 7H), 1.01 (t, J = 7.1 Hz, 3H) ppm. $^{13}C\{^{1}H\}$ NMR (126 MHz, DMSO-d6): δ 172.4, 161.6, 139.8, 127.0, 73.1, 70.7, 60.2, 41.9, 29.3, 29.0, 22.2, 16.3, 13.7 ppm. LCMS $(M+H)^+$: 392. HRMS (ESI-TOF) *m/z*: $[M + H]^+$ calcd for $C_{14}H_{19}INO_4$, 392.0359; found 392.0355.

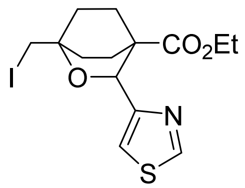

Ethyl 1-(iodomethyl)-3-(thiazol-4-yl)-2-oxabicyclo[2.2.2]octane-4-carboxylate (22)

General procedure A. Yield: 0.98 g, 0.0024 mol, 39%, yellow solid. ^{1}H NMR (400 MHz, CD_3CN): δ 8.77 (d, J = 2.0 Hz, 1H), 7.65 (s, 1H), 5.32 (s, 1H), 4.08 – 3.98 (m, 2H), 3.34 (s, 2H), 2.12 – 1.98 (m, 2H), 1.89 – 1.50 (m, 6H), 1.15 (t, J = 7.1 Hz, 3H) ppm. $^{13}C\{^{1}H\}$ NMR (126 MHz, CD_3CN): δ 174.3, 158.9, 154.0, 117.1, 79.0, 71.6, 61.3, 44.3, 30.6, 30.4, 30.3, 22.5, 17.4, 14.3 ppm. LCMS $(M+H)^+$: 408. HRMS (ESI-TOF) *m/z*: $[M + H]^+$ calcd for $C_{14}H_{19}INO_3S$, 408.0130; found 408.0126.

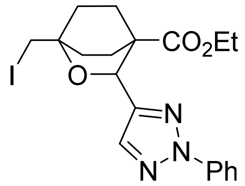

Ethyl 1-(iodomethyl)-3-(2-phenyl-2*H*-1,2,3-triazol-4-yl)-2-oxabicyclo[2.2.2]octane-4-carboxylate (23)

General procedure A. Yield: 0.23 g, 0.0005 mol, 99%, yellow oil. ^{1}H NMR (400 MHz, DMSO-d6): δ 8.13 (s, 1H), 7.93 (d, J = 8.1 Hz, 2H), 7.56 (t, J = 7.8 Hz, 2H), 7.41 (t, J = 7.4 Hz, 1H), 5.33 (s, 1H), 4.05 (q, J = 7.0 Hz, 2H), 3.39 (s, 2H), 2.22 (t, J = 11.4 Hz, 1H), 2.08 – 1.67 (m, 7H), 1.09 (t, J = 7.1 Hz, 3H) ppm. $^{13}C\{^{1}H\}$ NMR (126 MHz, DMSO-d6): δ 172.6, 149.8, 139.1, 135.1, 129.7, 127.7, 118.1, 73.5, 70.4, 60.3, 42.9, 29.3, 29.0, 21.4, 17.1, 13.9 ppm. LCMS $(M+H)^+$: 468. HRMS (ESI-TOF) *m/z*: $[M + H]^+$ calcd for $C_{19}H_{23}IN_3O_3$, 468.0784; found 468.0790.

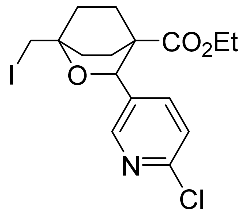

Ethyl 3-(6-chloropyridin-3-yl)-1-(iodomethyl)-2-oxabicyclo[2.2.2]octane-4-carboxylate (24)

General procedure A. Yield: 0.33 g, 0.00076 mol, 80%, yellow oil. R_f = 0.64 (hexane/EtOAc, 4:1). ^{1}H NMR (400 MHz, $CDCl_3$): δ 8.36 (s, 1H), 7.71 (dd, J = 8.2, 2.2 Hz, 1H), 7.29 (d, J = 8.3 Hz, 1H),

5.25 (s, 1H), 4.09 – 3.93 (m, 2H), 3.27 (q, *J* = 10.6 Hz, 2H), 2.42 (t, *J* = 12.0 Hz, 1H), 2.27 – 2.07 (m, 2H), 2.01 – 1.71 (m, 5H), 1.14 (t, *J* = 7.1 Hz, 3H) ppm. $^{13}C\{^1H\}$ NMR (126 MHz, $CDCl_3$): δ 173.1, 151.0, 148.9, 138.0, 134.4, 123.7, 78.2, 70.9, 61.2, 44.6, 30.4, 29.9, 20.9, 15.3, 14.1 ppm. LCMS $(M+H)^+$: 436. HRMS (ESI-TOF) *m/z*: $[M + H]^+$ calcd for $C_{16}H_{20}ClINO_3$, 436.0176; found 436.0164.

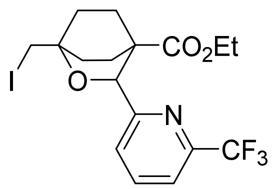

Ethyl 1-(iodomethyl)-3-(6-(trifluoromethyl)pyridin-2-yl)-2-oxabicyclo[2.2.2]octane-4-carboxylate (25)

General procedure A. Yield: 0.42 g, 0.00089 mol, 63%, colorless oil. R_f = 0.63 (hexane/EtOAc, 4:1). ^1H NMR (400 MHz, DMSO-d_6): δ δ 8.67 (s, 1H), 7.99 (d, *J* = 7.9 Hz, 1H), 7.92 (d, *J* = 8.1 Hz, 1H), 5.28 (s, 1H), 3.99 – 3.87 (m, 2H), 3.43 (q, *J* = 10.4 Hz, 2H), 2.33 (t, *J* = 11.6 Hz, 1H), 2.15 – 1.71 (m, 7H), 1.02 (t, *J* = 7.0 Hz, 3H) ppm. $^{13}C\{^1H\}$ NMR (151 MHz, DMSO-d_6): δ 172.4, 148.8, 145.8 (q, *J* = 33.9 Hz), 139.1, 136.9, 121.6 (q, *J* = 274.0 Hz), 120.1, 77.5, 70.5, 60.5, 44.0, 29.5, 28.9, 28.8, 20.5, 17.0, 13.6 ppm. $^{19}F\{^1H\}$ NMR (376 MHz, DMSO-d_6): δ -66.8 (s) ppm. LCMS $(M+H)^+$: 470. HRMS (ESI-TOF) *m/z*: $[M + H]^+$ calcd for $C_{17}H_{20}F_3INO_3$, 470.0440; found 470.0432.

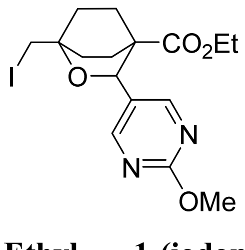

Ethyl 1-(iodomethyl)-3-(2-methoxypyrimidin-5-yl)-2-oxabicyclo[2.2.2]octane-4-carboxylate (26)

General procedure A. Yield: 0.44 g, 0.001 mol, 63%, yellow oil. R_f = 0.74 (hexane/EtOAc, 4:1). ^1H NMR (400 MHz, DMSO-d_6): δ 8.50 (s, 2H), 5.14 (s, 1H), 4.01 – 3.93 (m, 2H), 3.91 (s, 2H), 3.39 (q, *J* = 10.6 Hz, 2H), 2.26 (t, *J* = 11.4 Hz, 1H), 2.09 – 1.70 (m, 7H), 1.06 (t, *J* = 7.1 Hz, 3H) ppm. $^{13}C\{^1H\}$ NMR (126 MHz, DMSO-d_6): δ 172.5, 164.9, 158.3, 126.2, 75.9, 70.4, 60.5, 54.6, 43.8, 29.5, 28.9, 28.8, 20.4, 17.1, 13.8 ppm. LCMS $(M+H)^+$: 433. HRMS (ESI-TOF) *m/z*: $[M + H]^+$ calcd for $C_{16}H_{22}IN_2O_4$, 433.0624; found 433.0614.

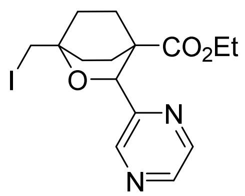

Ethyl 1-(iodomethyl)-3-(pyrazin-2-yl)-2-oxabicyclo[2.2.2]octane-4-carboxylate (27)

General procedure A. Yield: 0.15 g, 0.00037 mol, 77%, white solid, m.p. = 77-78 °C. R_f = 0.77 (hexane/EtOAc, 4:1). ^{1}H NMR (400 MHz, DMSO-d$_6$): δ 8.97 (s, 1H), 8.57 (d, *J* = 2.2 Hz, 1H), 8.52 (s, 1H), 5.19 (s, 1H), 4.00 (q, *J* = 7.1 Hz, 2H), 3.44 (q, *J* = 10.6 Hz, 2H), 2.26 (t, *J* = 10.8 Hz, 1H), 2.11 – 1.98 (m, 1H), 1.97 – 1.78 (m, 3H), 1.74 – 1.54 (m, 3H), 1.08 (t, *J* = 7.1 Hz, 3H) ppm. ^{13}C{^{1}H} NMR (126 MHz, DMSO-d$_6$): δ 172.7, 155.2, 143.7, 143.16, 143.15, 78.9, 70.6, 60.1, 42.6, 29.3, 29.2, 29.0, 21.3, 17.2, 13.8 ppm. LCMS (M+H)$^+$: 403. HRMS (ESI-TOF) *m/z*: [M + H]$^+$ calcd for $C_{15}H_{20}IN_2O_3$, 403.0519; found 403.0504.

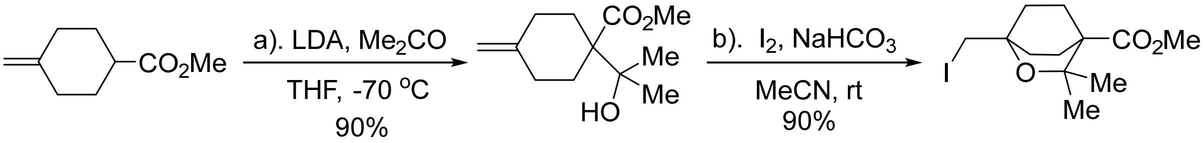

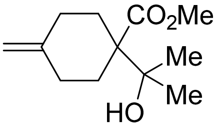

a). **Methyl 1-(2-hydroxypropan-2-yl)-4-methylenecyclohexane-1-carboxylate (SI-4)**

To a solution of DIPEA (135.45 g, 1.05 mol, 1.05 equiv) in THF (1000 mL) was added a solution of *n*BuLi (2.5M in hexane, 500 mL, 1.15 mol, 1.15 equiv) dropwise during ca. 10 min at -10 – -20 °C under Ar. After addition, the mixture was cooled to -78 °C and methyl 4-methylenecyclohexane-1-carboxylate (154.00 g, 1.00 mol, 1.00 equiv) was added dropwise at -70 – -80 °C. The mixture was stirred for 45 min at the same temperature, and acetone (116.00 g, 2.00 mol, 2.00 equiv) was added dropwise at -70 – -80 °C. The resulting mixture was stirred for 2 h at -50 – -60 °C and neutralized with a sat. solution of NH_4Cl (300 mL). The mixture was partially concentrated and extracted with MeO*t*Bu (3 × 300 mL). The combined organic layers were dried over Na_2SO_4, filtered and concentrated under reduced pressure. Yield: 190.80 g, 0.90 mol, 90%, colorless oil. The crude product was used for the next step without purification. ^{1}H NMR (500 MHz, CDCl$_3$): δ 7.26 (s, 1H), 4.62 (s, 2H), 3.77 (s, 3H), 2.30 – 2.23 (m, 4H), 2.10 – 2.03 (m, 2H), 1.48 – 1.41 (m, 2H), 1.16 (s, 6H) ppm. ^{13}C{^{1}H} NMR (126 MHz, CDCl$_3$): δ 176.4, 148.1, 107.4, 73.9, 55.6, 51.9, 32.3, 30.5, 26.0 ppm. HRMS (ESI-TOF) *m/z*: [M + H]$^+$ calcd for $C_{12}H_{21}O_3$, 213.1491; found 213.1480.

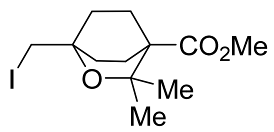

b). **Methyl 1-(iodomethyl)-3,3-dimethyl-2-oxabicyclo[2.2.2]octane-4-carboxylate (30)**

To a solution of methyl 1-(2-hydroxypropan-2-yl)-4-methylenecyclohexane-1-carboxylate (190.80 g, 0.90 mol, 1.00 equiv) in MeCN (3000 mL) were added $NaHCO_3$ (181.44 g, 2.16 mol, 2.40 equiv) in one portion and I_2 (548.64 g, 2.16 mol, 2.40 equiv) in four portions. The resulting mixture was stirred for 12 h at room temperature. Then sodium thiosulfate pentahydrate (669.60 g, 2.70 mol, 3.00 equiv) and distilled water (1500 mL) were added to the mixture. The colorless mixture was extracted with MeO*t*Bu (10 × 200 mL). The combined organic layers were concentrated under reduced pressure. The residue was dissolved in MeO*t*Bu (500 mL), washed with brine (1 × 200 mL), a sat. solution of $Na_2S_2O_3$ (3 × 200 mL), dried over Na_2SO_4, filtered through SiO_2 (0.5 L glass filter filed with 3 cm in high with silica gel) and concentrated. The formed resulting crystals of the desired product were filtered off, washed with a mixture of hexane/Et_2O = 10:1 (ca. 200 mL) on the filter, and dried on air to give the pure product (149.10 g). The combined mother liquor was evaporated, and purified by column chromatography (SiO_2, hexane/EtOAc, 1:5, R_f = 0.7) to provide the second portion of the pure product (124.70 g). Combined yield: 273.80 g, 0.81 mol, 90%, yellow solid, m.p. = 68-69 °C. ^1H NMR (500 MHz, $CDCl_3$): δ 3.66 (s, 3H), 3.12 (s, 2H), 2.50 – 2.33 (m, 2H), 1.94 – 1.81 (m, 2H), 1.78 – 1.62 (m, 4H), 1.28 (s, 6H) ppm. $^{13}C\{^1H\}$ NMR (151 MHz, $CDCl_3$): δ 174.7, 76.8, 70.4, 52.0, 45.4, 30.0, 26.9, 25.8, 16.8 ppm. HRMS (ESI-TOF) *m/z*: $[M + H]^+$ calcd for $C_{12}H_{20}IO_3$, 339.0457; found 339.0452.

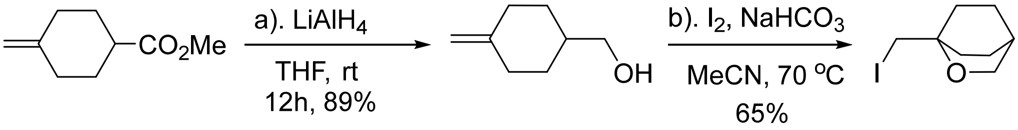

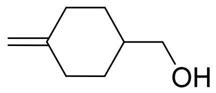

a). **(4-Methylenecyclohexyl)methanol (SI-5)**

To a suspension of $LiAlH_4$ (21.20 g, 0.56 mol, 0.65 equiv) in THF (2000 mL) was added a solution of methyl 4-methylenecyclohexane-1-carboxylate (149.52 g, 0.86 mol, 1.00 equiv) in THF (150 mL) at 0-30 °C under Ar. The resulting mixture was stirred at room temperature overnight. The excess of $LiAlH_4$ was neutralized with water (21 mL, 1.17 mol, 1.36 equiv) and 100 mL of a 20% aq. solution of NaOH. The suspension was filtered through Na_2SO_4, washed with THF and concentrated under reduced pressure. The product was used for the next step without purification. Yield: 96.01 g, 0.762 mol, 89%, colorless oil. ^1H NMR (400 MHz, $CDCl_3$): δ 4.62 (s, 2H), 3.48 (d, *J* = 6.4 Hz, 2H), 2.33 (d, *J* = 13.4 Hz, 2H), 2.04 (t, *J* = 12.9 Hz, 2H), 1.87 (dd, *J* = 12.5, 2.4 Hz,

2H), 1.74 – 1.52 (m, 1H), 1.44 (br s, 1H), 1.14 – 0.94 (m, 2H) ppm. $^{13}C\{^1H\}$ NMR (126 MHz, $CDCl_3$): δ 149.5, 107.2, 68.1, 40.1, 34.3, 30.9 ppm. GCMS (M): 126. HRMS (ESI-TOF) *m/z*: not found.

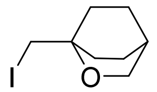

b). **1-(Iodomethyl)-2-oxabicyclo[2.2.2]octane (31)**

$NaHCO_3$ (230.00 g, 2.73 mol, 3.00 equiv) and I_2 (694.00 g, 2.73 mol, 3 equiv) were added to 2800 mL of MeCN (~0.3M in relation to alcohol). The solution was heated to 70 °C, and a solution of (4-methylenecyclohexyl)methanol (115.00 g, 0.91 mol, 1.00 equiv) in 200 mL of MeCN was added dropwise over 20 min. The heating was stopped, and the mixture was stirred for 40 min, then sodium thiosulfate pentahydrate (677.04 g, 2.73 mol, 3.00 equiv) and water (1000 mL) were added. The colorless mixture was extracted with MeO*t*Bu (2 × 400 mL). The combined organic layers were concentrated under reduced pressure. The residue was dissolved in MeO*t*Bu (1500 mL), washed with brine (1 × 300 mL) dried over Na_2SO_4, filtered and concentrated. The final product was purified by column chromatography (SiO_2, hexane/EtOAc, 4:1, R_f = 0.7). Yield: 150.00 g, 0.595 mol, 65%, yellow oil. ^1H NMR (400 MHz, $CDCl_3$): δ 3.92 (s, 2H), 3.11 (s, 2H), 2.06 – 1.89 (m, 2H), 1.89 – 1.74 (m, 2H), 1.74 – 1.59 (m, 5H) ppm. $^{13}C\{^1H\}$ NMR (126 MHz, $CDCl_3$): δ 70.7, 68.7, 31.2, 25.9, 24.7, 17.2 ppm. GCMS (M): 252. HRMS (ESI-TOF) *m/z*: $[M + H]^+$ calcd for $C_8H_{14}IO$, 253.0089; found 253.0084.

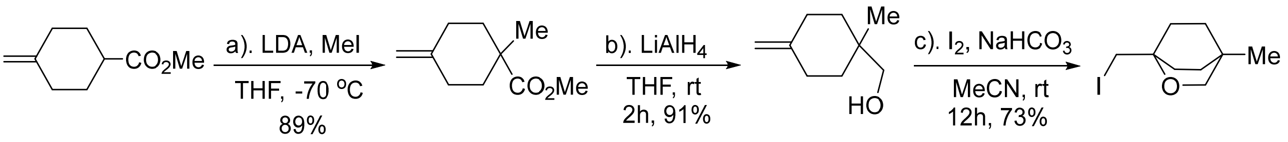

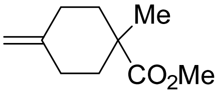

a). **Methyl 1-methyl-4-methylenecyclohexane-1-carboxylate (SI-6)**

To a solution of DIPEA (135.45 g, 1.05 mol, 1.05 equiv) in THF (1000 mL) was added a solution of *n*BuLi (2.5M in hexane, 460 mL, 1.15 mol, 1.15 equiv) dropwise at -10 – -20 °C under Ar. After addition, the mixture was cooled to -78 °C and methyl 4-methylenecyclohexane-1-carboxylate (154.00 g, 1.00 mol, 1.00 equiv) was added dropwise at -70 – -80 °C. The mixture was stirred for 45 min at the same temperature, and MeI (213.00 g, 1.50 mol, 1.50 equiv) was added dropwise at -70 – -80 °C. The resulting mixture was stirred overnight at room temperature and neutralized with a sat. solution of NH_4Cl (300 mL). The mixture was partially concentrated and extracted with MeO*t*Bu (3 × 300 mL). The combined organic layers were dried over Na_2SO_4, filtered and

concentrated under reduced pressure. Yield: 149.52 g, 0.89 mol, 89%, colorless oil. The crude product was used for the next step without purification. ^{1}H NMR (500 MHz, $CDCl_3$): δ 4.62 (s, 2H), 3.69 (s, 3H), 2.25 – 2.03 (m, 6H), 1.41 – 1.27 (m, 2H), 1.19 (s, 3H) ppm. $^{13}C\{^1H\}$ NMR (126 MHz, $CDCl_3$): δ 177.9, 148.4, 107.4, 51.9, 43.2, 36.8, 31.9, 26.2 ppm. GCMS (M): 168. HRMS (ESI-TOF) *m/z*: not found.

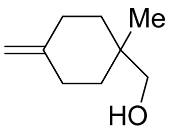

b). **(1-Methyl-4-methylenecyclohexyl)methanol (SI-7)**

To a suspension of $LiAlH_4$ (22.80 g, 0.60 mol, 0.70 equiv) in THF (1500 mL) was added a solution of methyl 1-methyl-4-methylenecyclohexane-1-carboxylate (149.52 g, 0.89 mol, 1.00 equiv) in THF (150 mL) at 5-15 °C under Ar. The resulting mixture was stirred at room temperature for 2 h. The excess of $LiAlH_4$ was neutralized with water and a sat. solution of NaOH. The suspension was filtered through Na_2SO_4. The filtrate was extracted with EtOAc (3 × 400 mL). The combined organic layers were concentrated under reduced pressure and used for the next step without purification. Yield: 113.40 g, 0.81 mol, 91%, colorless oil. ^{1}H NMR (500 MHz, $CDCl_3$): δ 4.61 (s, 2H), 3.39 (s, 2H), 2.30 – 2.10 (m, 4H), 1.47 – 1.37 (m, 4H), 0.98 (s, 3H) ppm. $^{13}C\{^1H\}$ NMR (126 MHz, $CDCl_3$): δ 149.6, 107.0, 72.1, 35.4, 35.1, 30.6, 21.5 ppm. GCMS (M): 140. HRMS (ESI-TOF) *m/z*: $[M + H]^+$ calcd for $C_9H_{17}O$, 141.1279; found 141.1272.

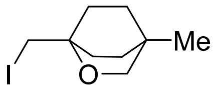

c). **1-(Iodomethyl)-4-methyl-2-oxabicyclo[2.2.2]octane (32)**

To a solution of (1-methyl-4-methylenecyclohexyl)methanol (113.40 g, 0.81 mol, 1.00 equiv) in MeCN (2500 mL) were added $NaHCO_3$ (162.96 g, 1.94 mol, 2.40 equiv) in one portion and I_2 (492.76 g, 1.94 mol, 2.40 equiv) in four portions. The resulting mixture was stirred for 12 h at room temperature. Then sodium thiosulfate pentahydrate (602.64 g, 2.43 mol, 3.00 equiv) and distilled water (1500 mL) were added to the mixture. The colorless mixture was extracted with MeO*t*Bu (3 × 200 mL). The combined organic layers were concentrated under reduced pressure. The residue was dissolved in MeO*t*Bu (500 mL), washed with brine (1 × 200 mL), a sat. solution of $Na_2S_2O_3$ (3 × 200 mL), dried over Na_2SO_4, filtered through SiO_2 and concentrated. The final product was purified by column chromatography (SiO_2, hexane/EtOAc, 1:5). Yield: 156.94 g, 0.59 mol, 73%, yellow oil. ^{1}H NMR (400 MHz, $CDCl_3$): δ 3.63 (s, 2H), 3.14 (s, 1H), 2.02 – 1.85 (m, 2H), 1.76 – 1.62 (m, 2H), 1.62 – 1.48 (m, 4H), 0.78 (s, 3H) ppm. $^{13}C\{^1H\}$ NMR (126 MHz, $CDCl_3$): δ 75.7, 69.0, 32.0, 31.1,

28.9, 23.2, 16.9 ppm. GCMS (M): 266. HRMS (ESI-TOF) *m/z*: $[M + H]^+$ calcd for $C_9H_{16}IO$, 267.0246; found 267.0234.

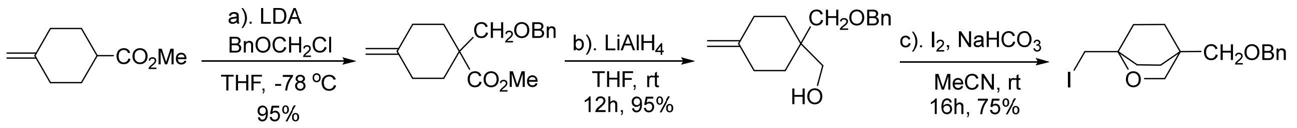

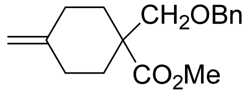

a). **Methyl 1-((benzyloxy)methyl)-4-methylenecyclohexane-1-carboxylate (SI-8)**

To a solution of DIPEA (180.60 g, 1.40 mol, 1.20 equiv) in THF (1000 mL) was added a solution of *n*BuLi (2.5M in hexane, 520 mL, 1.30 mol, 1.13 equiv) dropwise at -10 – -20 °C under Ar. After addition, the mixture was cooled to -78 °C and methyl 4-methylenecyclohexane-1-carboxylate (177.10 g, 1.15 mol, 1.00 equiv) was added dropwise at -70 – -80 °C. The mixture was stirred for 45 min at the same temperature, and benzyl chloromethyl ether (180.00 g, 1.15 mol, 1.00 equiv) was added dropwise at -70 – -60 °C. The resulting mixture was stirred overnight at room temperature and neutralized with a sat. solution of NH_4Cl (300 mL). The mixture was partially concentrated and extracted with MeO*t*Bu (3 × 300 mL). The combined organic layers were dried over Na_2SO_4, filtered and concentrated under reduced pressure. Yield: 299.35 g, 1.09 mol, 95%, colorless oil. The crude methyl 1-((benzyloxy)methyl)-4-methylenecyclohexane-1-carboxylate was used for the next step without purification. ^1H NMR (500 MHz, $CDCl_3$): δ 7.35 – 7.20 (m, 5H), 4.62 (s, 2H), 4.49 (s, 2H), 3.71 (s, 3H), 3.46 (s, 2H), 2.25 – 2.09 (m, 6H), 1.45 – 1.38 (m, 2H) ppm. $^{13}C\{^1H\}$ NMR (151 MHz, $CDCl_3$): δ 175.7, 148.4, 138.4, 128.4, 127.6, 127.5, 107.5, 76.7, 73.3, 52.0, 48.3, 32.6, 31.5 ppm. HRMS (ESI-TOF) *m/z*: $[M + H]^+$ calcd for $C_{17}H_{23}O_3$, 275.1647; found 275.1635.

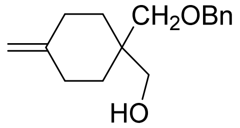

b). **(1-((Benzyloxy)methyl)-4-methylenecyclohexyl)methanol (SI-9)**

A solution of methyl 1-((benzyloxy)methyl)-4-methylenecyclohexane-1-carboxylate (27.40 g, 0.10 mol, 1.00 equiv) in THF (20 mL) was added dropwise to a suspension of $LiAlH_4$ (2.30 g, 0.06 mol, 0.60 equiv) in THF (150 mL) at 0-10 °C. The reaction mixture was warmed to room temperature and left stirring overnight. The mixture was quenched with water (2.2 mL, 0.12 mol, 1.20 equiv) and a sat. solution of NaOH (9.60 g, 0.24 mol, 2.40 equiv). The mixture was filtered through a thick pad of Na_2SO_4. The solid residue was washed with hot THF. The filtrated was concentrated. The

crude product was purified by distillation (b.p. = 78-79 °C, 1 mmHg) to give the desired product. Yield: 23.37 g, 0.095 mol, 95%, yellow oil. ^{1}H NMR (500 MHz, $CDCl_3$): δ 7.40 – 7.27 (m, 5H), 4.62 (s, 2H), 4.52 (s, 2H), 3.63 (d, *J* = 6.0 Hz, 2H), 3.45 (s, 2H), 2.82 (t, *J* = 6.0 Hz, 1H), 2.20 – 2.06 (m, 4H), 1.60 – 1.51 (m, 2H), 1.48 – 1.42 (m, 2H) ppm. ^{13}C{^{1}H} NMR (151 MHz, $CDCl_3$): δ 149.1, 138.1, 128.6, 127.9, 127.6, 107.3, 77.9, 73.8, 69.2, 38.4, 31.4, 30.3 ppm. HRMS (ESI-TOF) *m/z*: $[M + H]^+$ calcd for $C_{16}H_{23}O_2$, 247.1698; found 247.1686.

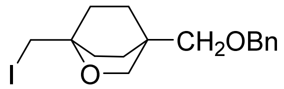

c). **4-((Benzyloxy)methyl)-1-(iodomethyl)-2-oxabicyclo[2.2.2]octane (33)**

To a solution of (1-((benzyloxy)methyl)-4-methylenecyclohexyl)methanol (23.37 g, 0.095 mol, 1.00 equiv) in MeCN (300 mL) were added $NaHCO_3$ (19.15 g, 0.228 mol, 2.40 equiv) in one portion and I_2 (57.91 g, 0.228 mol, 2.40 equiv) in four portions. The resulting mixture was stirred for 16 h at room temperature. Then sodium thiosulfate pentahydrate (70.68 g, 0.285 mol, 3.00 equiv) and distilled water (300 mL) were added to the mixture. The colorless mixture was extracted with MeO*t*Bu (3 × 200 mL). The combined organic layers were concentrated under reduced pressure to dryiness. The residue was dissolved in MeO*t*Bu (200 mL), washed with brine (1 × 100 mL), a sat. solution of $Na_2S_2O_3$ (3 × 100 mL), dried over Na_2SO_4, filtered through SiO_2 and concentrated. The final product was purified by column chromatography (SiO_2, hexane/EtOAc, 1:3). Yield: 26.50 g, 0.07 mol, 75%, yellow oil. ^{1}H NMR (400 MHz, $CDCl_3$): δ 7.38 – 7.13 (m, 5H), 4.48 (s, 2H), 3.82 (s, 2H), 3.17 (s, 2H), 3.15 (s, 2H), 2.03 – 1.90 (m, 2H), 1.83 – 1.66 (m, 4H), 1.65 – 1.55 (m, 2H) ppm. ^{13}C{^{1}H} NMR (126 MHz, $CDCl_3$): δ 138.6, 128.5, 127.7, 127.4, 75.2, 73.4, 72.5, 69.6, 33.7, 30.6, 27.5, 16.6 ppm. GCMS (M): 372. HRMS (ESI-TOF) *m/z*: $[M + NH_4]^+$ calcd for $C_{16}H_{25}INO_2$, 390.0930; found 390.0928.

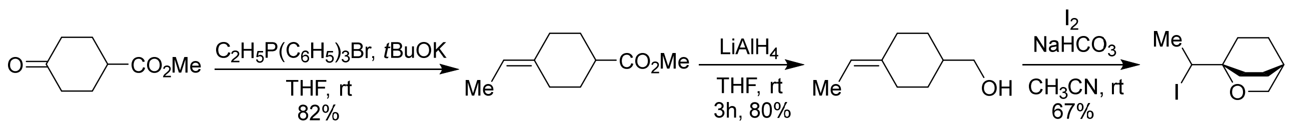

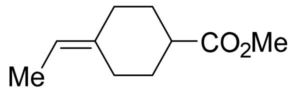

Methyl 4-ethylidenecyclohexane-1-carboxylate (SI-10)

To a suspension of $C_2H_5P(C_6H_5)_3Br$ (66.78 g, 0.18 mol, 1.20 equiv) in THF (350 mL) was added *t*BuOK (20.16 g, 0.18 mol, 1.20 equiv) with vigorous stirring at room temperature. After 2 h, the mixture was cooled to 0 ºC (ice bath) and methyl 4-oxocyclohexane-1-carboxylate (23.40 g, 0.15 mol, 1.50 equiv) was added dropwise (30 min) at 0-5 °C. The mixture was stirred for 48 h at room temperature and monitored with ^{1}H NMR. After consumption of the starting material, the mixture

was concentrated under reduced pressure. The residue was diluted with water (150 mL) and extracted with hexane (4 × 50 mL). The combined organic layers were dried over Na_2SO_4, filtered and concentrated under reduced pressure. Yield: 20.16 g, 0.12 mol, 82%, colorless oil. ^{1}H NMR (500 MHz, $CDCl_3$): δ 5.18 (q, *J* = 6.6 Hz, 1H), 3.66 (s, 3H), 2.59 (dt, *J* = 13.7, 3.8 Hz, 1H), 2.46 (tt, *J* = 11.2, 3.7 Hz, 1H), 2.22 (dt, *J* = 13.2, 3.6 Hz, 1H), 2.06 – 1.93 (m, 3H), 1.79 (t, *J* = 13.0 Hz, 1H), 1.56 (d, *J* = 6.7 Hz, 3H), 1.54 – 1.45 (m, 2H) ppm. $^{13}C\{^1H\}$ NMR (126 MHz, $CDCl_3$): δ 176.2, 137.8, 116.7, 51.7, 43.3, 35.4, 30.6, 29.6, 26.6, 12.8 ppm. GCMS (M): 168. HRMS (ESI-TOF) *m/z*: $[M + H]^+$ calcd for $C_{10}H_{17}O_2$, 169.1229; found 169.1191.

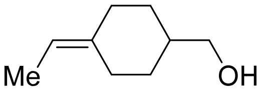

(4-Ethylidenecyclohexyl)methanol (34)

To a suspension of $LiAlH_4$ (11.66 g, 0.308 mol, 0.80 equiv) in THF (1500 mL) was added a solution of methyl 4-ethylidenecyclohexane-1-carboxylate (70.00 g, 0.384 mol, 1.00 equiv) at 0 °C dropwise over 1 h under Ar. The resulting mixture was stirred at room temperature for 3 h. The excess of $LiAlH_4$ was neutralized with water (15 mL) and 50 mL of a 20% aq. solution of NaOH dropwise over 30 min at 0 °C under Ar. The suspension was filtered through Na_2SO_4, washed with THF and concentrated under reduced pressure. The product was purified by distillation (b.p. = 65 °C, 1 mmHg). Yield: 43.40 g, 0.31 mol, 80%, purity ~ 90%, colorless oil. ^{1}H NMR (500 MHz, $CDCl_3$): δ 5.17 (q, *J* = 6.6 Hz, 1H), 3.47 (d, *J* = 6.4 Hz, 2H), 2.64 (d, *J* = 13.7 Hz, 1H), 2.21 (d, *J* = 13.5 Hz, 1H), 2.03 (t, *J* = 12.8 Hz, 1H), 1.88 – 1.79 (m, 2H), 1.78 – 1.63 (m, 2H), 1.57 (d, *J* = 6.7 Hz, 3H), 1.40 (s, 1H), 1.04 – 0.90 (m, 2H) ppm. $^{13}C\{^1H\}$ NMR (126 MHz, $CDCl_3$): δ 139.4, 115.9, 68.3, 40.8, 35.9, 31.1, 30.2, 27.0, 12.8 ppm. GCMS (M): 140. HRMS (ESI-TOF) *m/z*: $[M + H]^+$ calcd for $C_9H_{17}O$, 141.1279; found 141.1274.

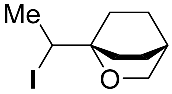

1-(1-Iodoethyl)-2-oxabicyclo[2.2.2]octane (35)

To a solution of (4-ethylidenecyclohexyl)methanol (43.00 g, 0.307 mol, 1.00 equiv) in MeCN (1000 mL) were added $NaHCO_3$ (77.28 g, 0.92 mol, 3.00 equiv) in one portion and I_2 (233.49 g, 0.92 mol, 3.00 equiv) in four portions. The resulting mixture was stirred for 12 h at room temperature. The mixture was concentrated under reduced pressure. The residue was diluted with MeO*t*Bu (200 mL) and washed with a sat. aq. solution of sodium thiosulfate (3 × 50 mL). The organic layer was dried over Na_2SO_4, filtered and concentrated. The final product was purified by column chromatography (SiO_2, hexane/MeO*t*Bu, 5:1, R_f = 0.75). Yield: 55.00 g, 67%, yellow solid. ^{1}H NMR (500 MHz, $CDCl_3$): δ 4.09 (q, *J* = 7.1 Hz, 1H), 3.91 (s, 2H), 1.99 – 1.93 (m, 1H), 1.84 (d, *J* = 7.1 Hz, 3H), 1.89

– 1.77 (m, 5H), 1.73 – 1.64 (m, 3H) ppm. $^{13}C\{^1H\}$ NMR (151 MHz, $CDCl_3$): δ 72.4, 70.6, 39.0, 29.3, 28.8, 26.0, 24.7, 24.6, 23.7 ppm. GCMS (M): 266. HRMS (ESI-TOF) *m/z*: $[M + H]^+$ calcd for $C_9H_{16}IO$, 267.0246; found 267.0239.

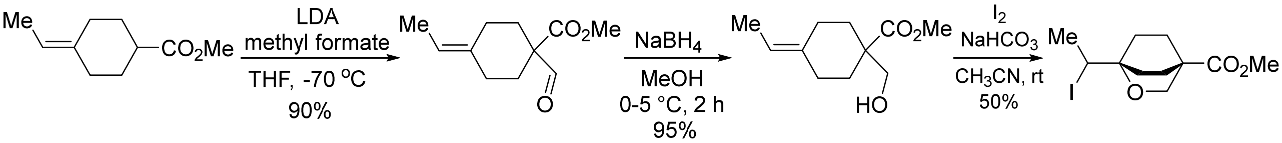

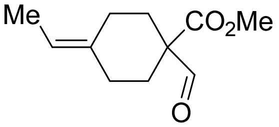

Methyl 4-ethylidene-1-formylcyclohexane-1-carboxylate (SI-11)

To a solution of DIPEA (18.06 g, 0.14 mol, 1.17 equiv) in THF (100 mL) was added a solution of *n*BuLi (2.5M in hexane, 50 mL, 0.13 mol, 1.08 equiv) dropwise at -10 – -20 °C under Ar over 10 min. After addition, the mixture was cooled to -78 °C and methyl 4-ethylidenecyclohexane-1-carboxylate (20.16 g, 0.12 mol, 1.00 equiv) was added dropwise at -70 – -80 °C. The mixture was stirred for 45 min at the same temperature, and methyl formate (21.60 g, 0.36 mol, 3.00 equiv) was added dropwise during ca. 20 min at -70 – -80 °C. The resulting mixture was stirred overnight at room temperature and neutralized with a sat. aq. solution of NH_4Cl (30 mL). The mixture was partially concentrated under a reduced pressure and extracted with MeO*t*Bu (3 × 50 mL). The combined organic layers were dried over Na_2SO_4, filtered and concentrated under reduced pressure. Yield: 21.17 g, 0.108 mol, 90%, colorless oil. The crude product was immediatelly used for the next step without purification. ^1H NMR (500 MHz, $CDCl_3$): δ 9.54 (s, 1H), 5.21 (q, *J* = 6.7 Hz, 1H), 3.75 (s, 3H), 2.20 (t, *J* = 6.2 Hz, 2H), 2.15 – 2.03 (m, 4H), 1.98 – 1.88 (m, 2H), 1.56 (d, *J* = 6.6 Hz, 3H) ppm. $^{13}C\{^1H\}$ NMR (126 MHz, $CDCl_3$): δ 199.1, 171.6, 136.3, 117.4, 59.7, 52.7, 32.4, 30.3, 29.2, 23.8, 12.8 ppm. GCMS (M): 196. HRMS (ESI-TOF) *m/z*: $[M + H]^+$ calcd for $C_{22}H_{36}NO_6$, 410.2543; found 410.2543.

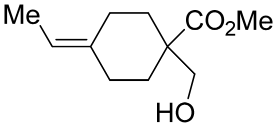

Methyl 4-ethylidene-1-(hydroxymethyl)cyclohexane-1-carboxylate (36)

To a solution of methyl 4-ethylidene-1-formylcyclohexane-1-carboxylate (19.60 g, 0.10 mol, 1.00 equiv) in MeOH (150 mL) was added $NaBH_4$ (1.33 g, 0.035 mol, 0.35 equiv) in portions at 0-5 °C over 30 min. The mixture was stirred for 2 h and concentrated under reduced pressure. The residue was dissolved in MeO*t*Bu (100 mL), washed with brine (2 × 20 mL) and dried over Na_2SO_4, filtered and concentrated. Yield: 18.81 g, 0.095 mol, 95%, colorless oil. The crude product was used for the

next step without purification. ^{1}H NMR (500 MHz, $CDCl_3$): δ 5.18 (q, J = 6.6 Hz, 1H), 3.73 (s, 3H), 3.64 (d, J = 6.2 Hz, 2H), 2.18 – 2.02 (m, 6H), 1.56 (d, J = 6.7 Hz, 3H), 1.43 – 1.37 (m, 2H) ppm. $^{13}C\{^1H\}$ NMR (126 MHz, $CDCl_3$): δ 176.8, 137.9, 116.5, 68.5, 52.1, 49.3, 32.6, 32.1, 31.1, 27.1, 24.0, 12.8 ppm. HRMS (ESI-TOF) *m/z*: $[M + Na]^+$ calcd for $C_{11}H_{18}NaO_3$, 221.1154; found 221.1164.

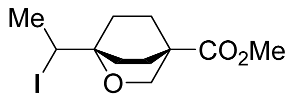

Methyl 1-(1-iodoethyl)-2-oxabicyclo[2.2.2]octane-4-carboxylate (37)

To a solution of methyl 4-ethylidene-1-(hydroxymethyl)cyclohexane-1-carboxylate (18.81 g, 0.095 mol, 1.00 equiv) in MeCN (300 mL) were added $NaHCO_3$ (19.15 g, 0.228 mol, 2.40 equiv) in one portion and I_2 (57.91 g, 0.228 mol, 2.40 equiv) in four portions. The resulting mixture was stirred for 16 h at room temperature. Then sodium thiosulfate pentahydrate (70.68 g, 0.285 mol, 3.00 equiv) and distilled water (150 mL) were added to the mixture. The colorless mixture was extracted with MeO*t*Bu (6 × 75 mL). The combined organic layers were concentrated under reduced pressure to dryiness. The residue was dissolved in MeO*t*Bu (250 mL), washed with brine (1 × 100 mL), a sat. solution of $Na_2S_2O_3$ (3 × 100 mL), dried over Na_2SO_4, filtered and concentrated. The final product was purified by column chromatography (SiO_2, hexane/EtOAc, 1:5, R_f = 0.70). Yield: 15.39 g, 0.0475 mol, 50%, yellow solid, m.p. = 37-38 °C. ^{1}H NMR (500 MHz, $CDCl_3$): δ 4.11 (q, J = 7.1 Hz, 1H), 3.98 (s, 2H), 3.66 (s, 3H), 2.05 – 1.86 (m, 8H), 1.85 (d, J = 7.1 Hz, 3H) ppm. $^{13}C\{^1H\}$ NMR (101 MHz, $CDCl_3$): δ 175.1, 73.2, 70.3, 52.1, 39.7, 37.0, 28.8, 27.8, 27.52, 27.47, 23.7 ppm. GCMS (M): 324. HRMS (ESI-TOF) *m/z*: $[M + H]^+$ calcd for $C_{11}H_{18}IO_3$, 325.0301; found 325.0297.

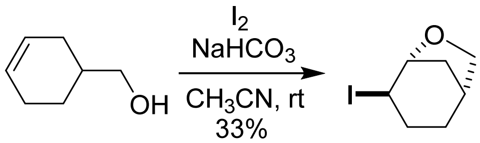

4-Iodo-6-oxabicyclo[3.2.1]octane (39)

In a flask fitted with a magnetic bar, cyclohex-3-en-1-ylmethanol (85.75 g, 0.766 mol, 1.00 equiv) was dissolved in 500 mL of dry CH_3CN under an argon atmosphere, and $NaHCO_3$ (192.66 g, 2.29 mol, 3.00 equiv) was added. The resulting mixture was stirred at room temperature for 5 min and cooled to 0 °C, and I_2 (582.00 g, 2.29 mol, 3.00 equiv) was added. The reaction mixture was left to warm to room temperature for 1 h. The mixture was diluted with MeO*t*Bu (700 mL) and washed with a 10% sodium thiosulfate solution until the color disappeared (3 × 400 mL). The aqueous layer was extracted with MeO*t*Bu (3 × 200 mL). The combination of extracts was dried, filtered, and

concentrated under reduced pressure. The residue was purified by column chromatography (SiO_2, hexane/EtOAc, 7:1, R_f = 0.5). Yield: 60.00 g, 0.25 mol, 33%, yellow oil. ^{1}H NMR (400 MHz, $CDCl_3$): δ 4.39 – 4.26 (m, 2H), 3.95 – 3.81 (m, 2H), 2.60 (d, J = 11.9 Hz, 1H), 2.40 – 2.26 (m, 2H), 1.96 – 1.78 (m, 3H), 1.58 – 1.43 (m, 1H) ppm. $^{13}C\{^1H\}$ NMR (126 MHz, $CDCl_3$): δ 79.4, 73.2, 35.0, 34.9, 31.3, 29.8, 26.3 ppm. GCMS (M): 238. HRMS (ESI-TOF) *m/z*: $[M + H]^+$ calcd for $C_7H_{12}IO$, 238.9933; found 238.9928.

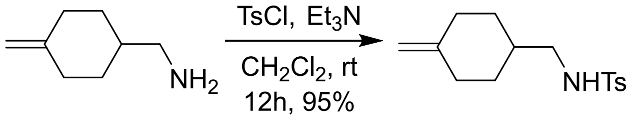

4-Methyl-*N*-((4-methylenecyclohexyl)methyl)benzenesulfonamide (40)

To a solution of (4-methylenecyclohexyl)methanamine hydrochloride (0.70 g, 4.33 mmol, 1.00 equiv) and Et_3N (1.32 g, 13.00 mmol, 3.00 equiv) in 10 mL of CH_2Cl_2 was added TsCl (0.83 g, 4.33 mmol, 1.00 equiv) in one portion at room temperature. The resulting mixture was stirred for 12 h and then washed with 1M HCl (3 mL). The organic layer was dried over Na_2SO_4, filtered and concentrated under reduced pressure. Yield: 1.15 g, 4.11 mmol, 95%, yellow solid, m.p. = 91-92 °C. ^{1}H NMR (500 MHz, $CDCl_3$): δ 7.74 (d, J = 8.2 Hz, 2H), 7.30 (d, J = 7.8 Hz, 2H), 4.72 (t, J = 5.7 Hz, 1H), 4.58 (s, 2H), 2.78 (t, J = 6.6 Hz, 2H), 2.42 (s, 3H), 2.25 (d, J = 13.4 Hz, 2H), 1.95 (t, J = 12.1 Hz, 2H), 1.78 (d, J = 11.5 Hz, 2H), 1.60 – 1.53 (m, 1H), 1.01 – 0.90 (m, 2H) ppm. $^{13}C\{^1H\}$ NMR (126 MHz, $CDCl_3$): δ 148.7, 143.5, 137.1, 129.8, 127.2, 107.6, 48.8, 37.5, 34.0, 31.7, 21.7 ppm. LCMS $(M+H)^+$: 280. HRMS (ESI-TOF) *m/z*: $[M + H]^+$ calcd for $C_{15}H_{22}NO_2S$, 280.1371; found 280.1364.

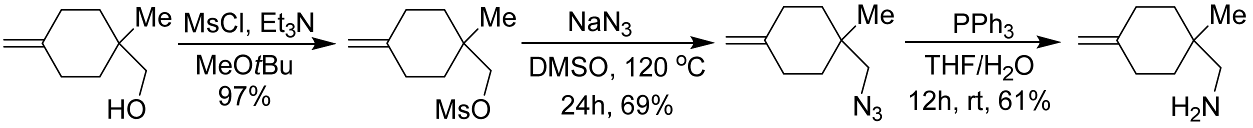

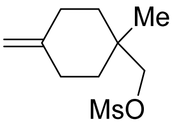

(1-Methyl-4-methylenecyclohexyl)methyl methanesulfonate (SI-12)

To a solution of (1-methyl-4-methylenecyclohexyl)methanol (12.00 g, 0.0857 mol, 1.00 equiv) in MeO*t*Bu (250 mL) was added Et_3N (17.30 g, 0.17 mo, 2.00 equiv). The resulting mixture was cooled to 0 °C and MsCl (14.70 g, 0.128 mol, 1.50 equiv) was added dropwise over 1 min. The mixture was filtered, washed with MeO*t*Bu (2 × 100 mL). The filtrate was concentrated under reduced pressure. Yield: 18.20 g, 0.0834 mol, 97%, yellow oil. ^{1}H NMR (500 MHz, $CDCl_3$): δ 4.64

(s, 2H), 3.98 (s, 2H), 3.01 (s, 3H), 2.33 – 2.14 (m, 4H), 1.52 – 1.41 (m, 4H), 1.06 (s, 3H) ppm. $^{13}C\{^1H\}$ NMR (151 MHz, $CDCl_3$): δ 148.1, 107.9, 77.6, 37.2, 35.2, 34.2, 30.3, 21.7 ppm. HRMS (ESI-TOF) *m/z*: $[M + NH_4]^+$ calcd for $C_{10}H_{22}NO_3S$, 236.1320; found 236.1317.

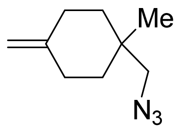

1-(Azidomethyl)-1-methyl-4-methylenecyclohexane (SI-13)

To a solution of (1-methyl-4-methylenecyclohexyl)methyl methanesulfonate (18.00 g, 0.082 mol, 1.00 equiv) in DMSO (150 mL) was added NaN_3 (16.00 g, 0.247 mol, 3.00 equiv). The resulting mixture was stirred at 120 °C (in an oil bath with a thermocouple) for 24 h. The mixture was cooled to room temperature and diluted with water (150 mL). The solution was extacted with MeO*t*Bu (3 × 150 mL). The combined organic layers were washed with brine (3 × 100 mL), dried over Na_2SO_4, filtered and concentrated under reduced pressure. Yield: 9.40 g, 0.057 mol, 69%, yellow oil. ^1H NMR (500 MHz, $CDCl_3$): δ 4.62 (s, 2H), 3.16 (s, 2H), 2.24 – 1.98 (m, 4H), 1.45 – 1.35 (m, 4H), 1.00 (s, 3H) ppm. $^{13}C\{^1H\}$ NMR (101 MHz, $CDCl_3$): δ 148.7, 107.5, 62.6, 36.4, 35.3, 30.5, 27.1, 22.6 ppm. HRMS (ESI-TOF) *m/z*: Not Found.

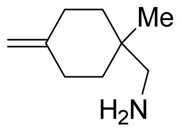

(1-Methyl-4-methylenecyclohexyl)methanamine (SI-14)

To a solution of 1-(azidomethyl)-1-methyl-4-methylenecyclohexane (9.00 g, 0.054 mol, 1.00 equiv) in THF (100 mL) and H_2O (30 mL) was added PPh_3 (20.00 g, 0.0765 mol, 1.40 equiv). The resulting mixture was stirred for 12 h at room temperature. The mixture was acidified with 3M HCl to pH = 2. The solution was washed with MeO*t*Bu (2 × 30 mL) and then alkalized with 1M NaOH to pH > 7. The mixture was extracted with MeO*t*Bu (3 × 100 mL). The combined organic layers were dried over Na_2SO_4, filtered and concentrated under reduced pressure. Yield: 4.60 g, 0.033 mol, 61%, brown oil. ^1H NMR (500 MHz, $CDCl_3$): δ 4.60 (s, 2H), 2.51 (s, 2H), 2.26 – 2.05 (m, 4H), 1.42 – 1.30 (m, 4H), 1.18 (s, 2H), 0.93 (s, 3H) ppm. $^{13}C\{^1H\}$ NMR (151 MHz, $CDCl_3$): δ 149.8, 106.8, 52.9, 36.5, 34.5, 30.8, 27.1, 22.1 ppm. HRMS (ESI-TOF) *m/z*: $[M + H]^+$ calcd for $C_9H_{18}N$, 140.1439; found 140.1434.

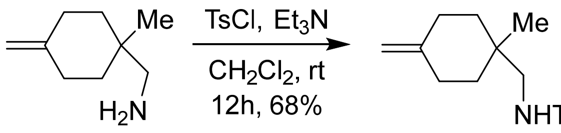

4-Methyl-*N*-((1-methyl-4-methylenecyclohexyl)methyl)benzenesulfonamide (42)

The same procedure as for **40** was used. Yield: 5.70 g, 0.0194 mol, 68%, brown oil. ^{1}H NMR (500 MHz, cdcl3) δ 7.74 (d, *J* = 6.6 Hz, 2H), 7.30 (d, *J* = 7.0 Hz, 2H), 4.59 (s, 2H), 2.81 – 2.71 (m, 2H), 2.43 (s, 3H), 2.18 – 2.00 (m, 4H), 1.62 (s, 1H), 1.34 (t, *J* = 5.7 Hz, 4H), 0.92 (s, 3H) ppm. $^{13}C\{^{1}H\}$ NMR (151 MHz, $CDCl_3$): δ 148.7, 143.4, 137.2, 129.9 (d, *J* = 12.6 Hz), 127.2, 107.4, 52.9, 36.4, 33.8, 30.4, 22.6, 21.7 ppm. HRMS (ESI-TOF) *m/z*: $[M + H]^{+}$ calcd for $C_{16}H_{24}NO_2S$, 294.1528; found 294.1523.

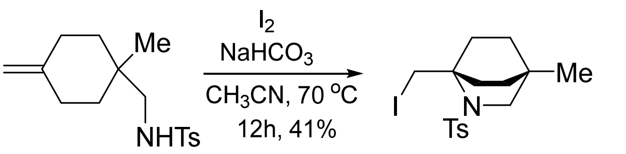

1-(Iodomethyl)-4-methyl-2-tosyl-2-azabicyclo[2.2.2]octane (43)

To a solution of 4-methyl-*N*-((1-methyl-4-methylenecyclohexyl)methyl)benzenesulfonamide (3.30 g, 0.011 mol, 1.00 equiv) in CH_3CN (50 mL) was added to a hot mixture of $NaHCO_3$ (11.40 g, 0.045 mol, 4.00 equiv) and I_2 (3.78 g, 0.045 mol, 4.00 equiv) in CH_3CN (150 mL) at 70 °C over 10 min. The resulting mixture was stirred at 70 °C (in an oil bath with a thermocouple) overnight. The mixture was concentrated under reduced pressure and diluted with MeO*t*Bu (300 mL). The solution was washed with a sat. aq. solution of sodium thiosulfate (2 × 100 mL), dried over Na_2SO_4, filtered and concentrated under reduced pressure. The final product was purified by column chromatography (SiO_2, gradient, hexane/MeO*t*Bu, 100% to 2:1). Yield: 1.89 g, 0.0045 mol, 41%, white solid, m.p. = 88-89 °C. ^{1}H NMR (500 MHz, $CDCl_3$): δ 7.83 (d, *J* = 8.1 Hz, 2H), 7.30 (d, *J* = 8.1 Hz, 2H), 3.77 (s, 2H), 3.17 (s, 2H), 2.43 (s, 3H), 2.00 – 1.91 (m, 2H), 1.80 – 1.72 (m, 2H), 1.49 – 1.39 (m, 4H), 0.86 (s, 3H) ppm. $^{13}C\{^{1}H\}$ NMR (151 MHz, $CDCl_3$): δ 143.4, 138.9, 129.7, 127.6, 57.9, 57.5, 33.3, 31.7, 30.4, 24.9, 21.7, 16.5 ppm. LCMS $(M+H)^{+}$: 420. HRMS (ESI-TOF) *m/z*: $[M + H]^{+}$ calcd for $C_{16}H_{23}INO_2S$, 420.0494; found 420.0485.

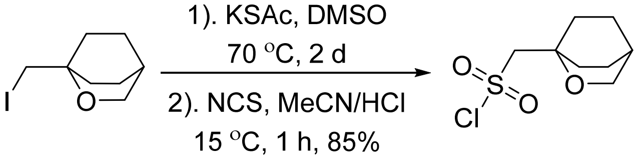

(2-Oxabicyclo[2.2.2]octan-1-yl)methanesulfonyl chloride (44)

To a solution of 1-(iodomethyl)-2-oxabicyclo[2.2.2]octane (25.20 g, 0.10 mol, 1.00 equiv) in DMSO (200 mL) was added KSAc (17.10 g, 0.15 mol, 1.50 equiv). The mixture was vigorously stirred at 70 °C for 2 d. The mixture was diluted with water (400 mL) and extracted with MeO*t*Bu (5 × 100 mL). The combined organic layers were washed with brine (3 × 100 mL), dried over Na_2SO_4, filtered and concentrated under reduced pressure. The crude product was dissolved in

MeCN (300 mL) and 2M HCl (50 mL), and then NCS (58.74 g, 0.44 mol, 4.40 equiv) was added in portions at 15-16 °C. The mixture was stirred for 1 h at room temperature and concentrated. The residue was dissolved in MeO*t*Bu (200 mL), washed with a saturated aq. solution of $NaHCO_3$ (3 × 100 mL). The organic layer was dried over Na_2SO_4, filtered and concentrated under reduced pressure to give the title product. Yield: 19.08 g, 0.085 mol, 85%, beige solid. ^{1}H NMR (500 MHz, $CDCl_3$): δ 3.95 (d, *J* = 1.2 Hz, 2H), 3.86 (s, 2H), 2.22 – 2.09 (m, 2H), 1.91 – 1.79 (m, 4H), 1.79 – 1.64 (m, 3H) ppm. $^{13}C\{^1H\}$ NMR (126 MHz, $CDCl_3$): δ 75.1, 70.6, 70.5, 31.2, 25.1, 24.3 ppm. HRMS (ESI-TOF) *m/z*: $[M + H]^+$ calcd for $C_8H_{14}ClO_3S$, 225.0352; found 225.0348.

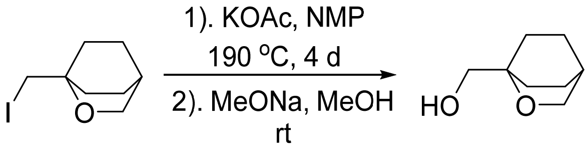

(2-Oxabicyclo[2.2.2]octan-1-yl)methanol (45)

To a solution of 1-(iodomethyl)-2-oxabicyclo[2.2.2]octane (150.00 g, 0.595 mol, 1.00 equiv) in NMP (1000 mL) was added KOAc (175.00 g, 1.785 mol, 3.00 equiv). The mixture was vigorously stirred overnight at 190 °C for 4 d. The mixture was diluted with water (1000 mL) and extracted with MeO*t*Bu (5 × 300 mL). The combined organic layers were washed with brine (5 × 300 mL), dried over Na_2SO_4, filtered and concentrated under reduced pressure. Yield: 94.94 g, 0.516 mol, 87%, colorless oil. The crude product was used for the next step without purification. To a solution of (2-oxabicyclo[2.2.2]octan-1-yl)methyl acetate (94.94 g, 0.516 mol, 1.00 equiv) in MeOH (1000 mL) was added MeONa (27.86 g, 0.516 mol, 1.00 equiv) in portions at 5-10 °C under Ar. The mixture was vigorously stirred overnight at room temperature. After that NH_4Cl (41.41 g, 0.774 mol, 1.50 equiv) was added, and the mixture was stirred for 1 h at room temperature. The mixture was concentrated under reduced pressure. The residue was diluted with MeO*t*Bu (1000 mL) and filtered. The filtrate was dried over Na_2SO_4, filtered and concentrated under reduced pressure. Yield: 65.90 g, 0.464 mol, 78%, yellow oil. The product was used for the next step without purification. ^{1}H NMR (500 MHz, $CDCl_3$): δ 3.89 (d, *J* = 1.2 Hz, 2H), 3.33 (s, 2H), 2.00 – 1.84 (m, 2H), 1.84 – 1.73 (m, 2H), 1.71 – 1.58 (m, 3H), 1.47 (td, *J* = 12.4, 4.2 Hz, 2H) ppm. $^{13}C\{^1H\}$ NMR (126 MHz, $CDCl_3$): δ 70.9, 70.2, 69.0, 28.1, 26.1, 24.3 ppm. HRMS (ESI-TOF) *m/z*: $[M + H]^+$ calcd for $C_8H_{15}O_2$, 143.1072; found 143.1065.

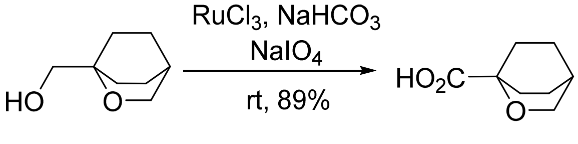

2-Oxabicyclo[2.2.2]octane-1-carboxylic acid (46)

To a solution of (2-oxabicyclo[2.2.2]octan-1-yl)methanol (30.00 g, 0.210 mol, 1.00 equiv) in a mixture of H_2O (200 mL), CH_3CN (200 mL) and CH_2Cl_2 (200 mL) were added $RuCl_3$ (1.20 g, 0.006 mol, 0.03 equiv) and $NaHCO_3$ (71.00 g, 0.844 mol, 4.00 equiv). The resulting mixture was cooled to 0 °C (ice-water bath), and $NaIO_4$ (135.00 g, 0.632 mol, 3.00 equiv) was added in portions. The mixture was vigorously stirred at room temperature until the appearance of a yellow color (~ 4-12 h). Then *i*-PrOH (32 mL, 0.42 mol, 2.00 equiv) was added, and the mixture was filtered and washed with water (2 × 50 mL). The layers were partitioned. An aqueous layer was washed with MeO*t*Bu (2 × 100 mL). The aqueous layer was acidified with 5M HCl to pH = 2 and extracted with EtOAc (4 × 300 mL). The combined organic phases were dried over Na_2SO_4, filtered and concentrated under reduced pressure. Yield: 29.40 g, 0.188 mol, 89%, yellow solid, m.p. = 77-78 °C. ^{1}H NMR (400 MHz, $CDCl_3$): δ 8.89 (br s, 1H), 4.03 (s, 2H), 2.15 – 1.94 (m, 4H), 1.91 – 1.70 (m, 4H) ppm. $^{13}C\{^1H\}$ NMR (126 MHz, $CDCl_3$): δ 176.1, 72.4, 70.9, 29.6, 25.6, 24.0 ppm. GCMS (M): 156. HRMS (ESI-TOF) *m/z*: $[M + H]^+$ calcd for $C_8H_{13}O_3$, 157.0865; found 157.0854.

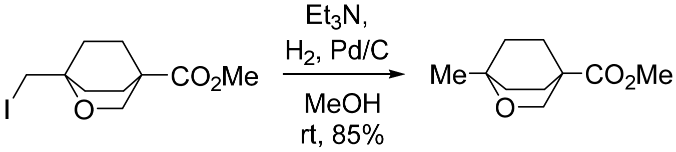

Methyl 1-methyl-2-oxabicyclo[2.2.2]octane-4-carboxylate (SI-15)

A round bottomed flask was charged with methyl 1-(iodomethyl)-2-oxabicyclo[2.2.2]octane-4-carboxylate (93.00 g, 0.30 mol, 1.00 equiv), Et_3N (31.90 g, 0.315 mol, 1.05 equiv) and Pd/C (5%) (10.00 g) in MeOH (750 mL). The mixture was hydrogenated under a rubber ball filled with H_2 at room temperature overnight. Pd/C was filtered out, and the reaction mixture was concentrated under reduced pressure. The solid residue was washed with MeO*t*Bu several times. The organic solution was dried over anhydrous Na_2SO_4, filtered and concentrated. The crude product was purified by distillation (1 mmHg, b.p. = 63-64 °C). Yield: 46.92 g, 0.255 mol, 85%, yellow oil. ^{1}H NMR (500 MHz, $CDCl_3$): δ 3.96 (s, 2H), 3.65 (s, 3H), 1.98 – 1.81 (m, 6H), 1.66 – 1.61 (m, 2H), 1.10 (s, 3H) ppm. $^{13}C\{^1H\}$ NMR (151 MHz, $CDCl_3$): δ 175.7, 70.0, 69.0, 52.0, 39.4, 32.1, 27.9, 26.4 ppm. GCMS (M): 184. HRMS (ESI-TOF) *m/z*: $[M + H]^+$ calcd for $C_{10}H_{17}O_3$, 185.1178; found 185.1171.

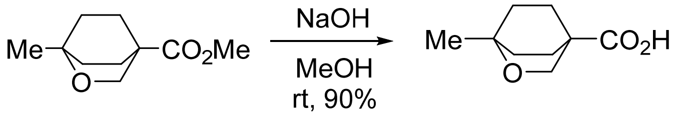

1-Methyl-2-oxabicyclo[2.2.2]octane-4-carboxylic acid (47)

Methyl 1-methyl-2-oxabicyclo[2.2.2]octane-4-carboxylate (10.86 g, 0.059 mol, 1.00 equiv) was dissolved in MeOH (10 mL) and NaOH (4.00 g, 0.07 mol, 1.20 equiv) in 50 mL of water was added. The solution was stirred at room temperature overnight. The solution was partially

concentrated under reduced pressure. The residue was washed with MeO*t*Bu (2 × 20 mL), and then acidified with HCl to pH ~ 2. The solution was extracted with EtOAc (5 × 20 mL). The combined fractions were washed with brine, dried over Na_2SO_4, filtered and concentrated under reduced pressure. The crude product was triturated in a mixture of hexane:Et_2O = 5:1 (ca. 50 mL) to give the desired product. Yield: 9.03 g, 0.0531 mol, 90%, white solid, m.p. = 137-138 °C. ^{1}H NMR (500 MHz, $CDCl_3$): δ 10.97 (br s, 1H), 3.98 (s, 2H), 2.06 – 1.77 (m, 6H), 1.63 (td, *J* = 12.6, 4.2 Hz, 2H), 1.11 (s, 3H) ppm. $^{13}C\{^1H\}$ NMR (126 MHz, $CDCl_3$): δ 181.3, 69.6, 69.3, 39.2, 32.0, 27.7, 26.3 ppm. GCMS (M): 170. HRMS (ESI-TOF) *m/z*: $[M + H]^+$ calcd for $C_9H_{15}O_3$, 171.1021; found 171.1015.

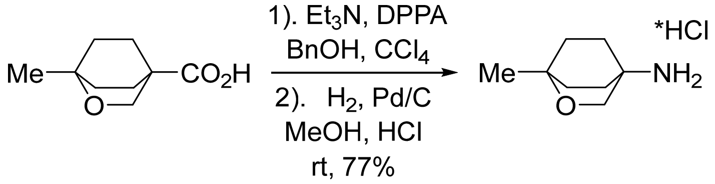

1-Methyl-2-oxabicyclo[2.2.2]octan-4-amine hydrochloride (48)

To a solution of 1-methyl-2-oxabicyclo[2.2.2]octane-4-carboxylic acid (5.95 g, 0.035 mol, 1.00 equiv) in CCl_4 (50 mL) was added Et_3N (5.2 mL, 0.037 mol, 1.05 equiv). The solution was heated to boiling and DPPA (9.63 g, 0.035 mol, 1.00 equiv) was added dropwise. The solution was heated at reflux for 2 h. After heating was stopped, BnOH (4.50 g, 0.042 mol, 1.20 equiv) was added in one portion. The mixture was left stirring for 10 h at room temperature. The mixture was concentrated under reduced pressure, and the residue was dissolved in MeO*t*Bu (200 mL). To the solution was added a saturated solution of $NaHCO_3$ (100 mL). The mixture was vigorously stirred for 2 h at room temperature. The layers were partitioned. The aqueous layer was additionally extracted with MeO*t*Bu (1 × 50 mL). The combined organic layers were dried over Na_2SO_4, filtered and concentrated under reduced pressure. The residue was triturated in a system of hexane-Et_2O to give the title product as a brown solid. The crude product (6.93 g, 0.030 mol, 1.00 equiv) was dissolved in 150 mL of MeOH and Pd/C (5%) (2.00 g) was added. The mixture was stirred under H_2-ballon at room temperature overnight. The reaction was monitored by TLC. After consumption of all starting material, Pd/C was filtered out, and the reaction mixture was concentrated under reduced pressure. The residue was dissolved in cold EtOAc (100 mL) and 5M HCl in dioxane was added dropwise to achieve a slightly acidic pH. The precipitate was filtered and dried. Yield: 4.79 g, 0.027 mol, 77%, white solid, m.p. = 214-215 °C. ^{1}H NMR (500 MHz, DMSO-d_6): δ 8.47 (br s, 3H), 3.75 (s, 2H), 1.94 – 1.80 (m, 4H), 1.78 – 1.64 (m, 4H), 1.01 (s, 3H) ppm. $^{13}C\{^1H\}$ NMR (126 MHz, DMSO-d_6): δ 68.8, 68.7, 48.8, 31.5, 27.9, 25.7 ppm. LCMS (M+H): 142. HRMS (ESI-TOF) *m/z*: $[M + H]^+$ calcd for $C_8H_{16}NO$, 142.232; found 142.1224.

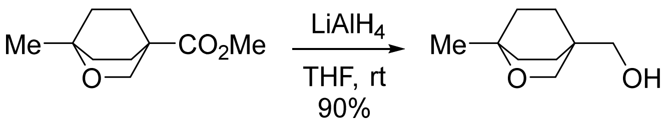

(1-Methyl-2-oxabicyclo[2.2.2]octan-4-yl)methanol (49)

A solution of methyl 1-methyl-2-oxabicyclo[2.2.2]octane-4-carboxylate (18.40 g, 0.10 mol, 1.00 equiv) in THF (200 mL) was added dropwise to a suspension of $LiAlH_4$ (2.30 g, 0.06 mol, 0.60 equiv) in THF (150 mL) at 0-10 °C. Then the reaction mixture was warmed to room temperature and left stirring overnight. The mixture was quenched with water (2.2 mL, 0.12 mol, 1.20 equiv) and a sat. aq. solution of NaOH (9.60 g, 0.24 mol, 2.40 equiv). The mixture was filtered through a thick pad of Na_2SO_4 (ca. 200 g). The solid residue was washed with hot THF (2 × 150 mL). The filtrated was concentrated. The crude product was purified by distillation (1 mmHg, b.p. = 78-79 °C) to give the title product. Yield: 14.04 g, 0.09 mol, 90%, colorless oil. ^{1}H NMR (500 MHz, $CDCl_3$): δ 3.74 (s, 2H), 3.30 (s, 2H), 1.85 – 1.75 (m, 2H), 1.66 – 1.58 (m, 4H), 1.53 – 1.46 (m, 2H), 1.09 (s, 3H) ppm. $^{13}C\{^1H\}$ NMR (126 MHz, $CDCl_3$): δ 159.2, 71.7, 69.3, 68.4, 33.9, 32.4, 27.2, 26.6 ppm. GCMS (M): 156. HRMS (ESI-TOF) *m/z*: $[M + H]^+$ calcd for $C_9H_{17}O_2$, 157.1229; found 157.1219.

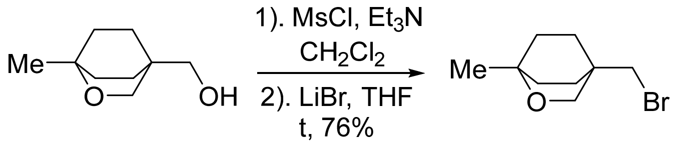

4-(Bromomethyl)-1-methyl-2-oxabicyclo[2.2.2]octane (50)

To a solution of (1-methyl-2-oxabicyclo[2.2.2]octan-4-yl)methanol (12.19 g, 0.078 mol, 1.00 equiv) in 150 mL of CH_2Cl_2 was added Et_3N (21.7 mL, 0.156 mol, 2.00 equiv). The mixture was cooled to 5 °C, and MsCl (14.90 g, 0.078 mol, 1.00 equiv) was added in portions. The mixture was stirred for 2 h at room temperature. Then it was washed with a solution of 0.5M HCl (1 × 100 mL) and brine (1 × 100 mL). The organic layer was dried over Na_2SO_4, filtered and concentrated under reduced pressure. To a solution of (1-methyl-2-oxabicyclo[2.2.2]octan-4-yl)methyl methanesulfonate (17.34 g, 0.074 mol, 1.00 equiv) in 150 mL of THF was added LiBr (25.75 g, 0.296 mol, 4.00 equiv). The resulting mixture was heated at reflux 24 h. The mixture was diluted with MeO*t*Bu (200 mL), washed with water (2 × 100 mL) and brine (1 × 100 mL). The solution was dried over Na_2SO_4, filtered and concentrated under reduced pressure to give the title product. Yield: 12.96 g, 0.059 mol, 76%, yellow oil. ^{1}H NMR (500 MHz, $CDCl_3$): δ 3.73 (s, 2H), 3.16 (s, 2H), 1.86 – 1.57 (m, 8H), 1.11 (s, 3H) ppm. $^{13}C\{^1H\}$ NMR (126 MHz, $CDCl_3$): δ 72.4, 69.2, 41.0, 33.0, 32.2, 29.1, 26.4 ppm. GCMS (M): 219. HRMS (ESI-TOF) *m/z*: $[M + H]^+$ calcd for $C_9H_{16}BrO$, 219.0385; found 219.0379.

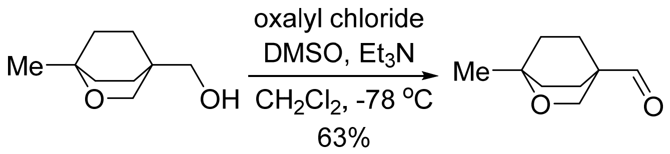

1-Methyl-2-oxabicyclo[2.2.2]octane-4-carbaldehyde (51)

To a solution of oxalyl chloride (57 mL, 0.66 mol, 2.60 equiv) in CH_2Cl_2 (500 mL) was added dry DMSO (96 mL, 1.35 mol, 5.25 equiv) dropwise under an agron atmosphere at -78 °C over 30 min. The resulting mixture was stirred at this temperature for 30 min, and then a solution of 4(1-methyl-2-oxabicyclo[2.2.2]octan-4-yl)methanol (40.00 g, 0.26 mol, 1.00 equiv) in CH_2Cl_2 (200 mL) was added dropwise at -78 °C over 15 min. The resulting mixture was stirred at this temperature for 1 h, then the flask was equipped with an overhead stirrer and Et_3N (230 mL, 1.65 mol, 6.50 equiv) was added dropwise over 1 h. The mixture was stirred at room temperature for 2 h and filtered. The solid residue was washed with MeO*t*Bu (200 mL). The filtrate was washed with distilled water (1 × 200 mL), 3M HCl (2 × 150 mL), 1M $NaHCO_3$ (1 × 150 mL), brine (1 × 150 mL) and concentrated under reduced pressure. The final product was purified by distillation (b.p. = 50 °C, 1 mmHg). Yield: 25.26 g, 0.164 mol, 63%, yellow oil. ^1H NMR (400 MHz, $CDCl_3$): δ 9.46 (s, 1H), 3.92 (s, 2H), 1.91 – 1.65 (m, 8H), 1.13 (s, 3H) ppm. $^{13}C\{^1H\}$ NMR (126 MHz, $CDCl_3$): δ 203.5, 69.8, 68.4, 44.5, 31.7, 26.4, 25.3 ppm. GCMS (M): 154. HRMS (ESI-TOF) m/z: [M + H]+ calcd for C9H15O2, 155.1072; found 155.1069.

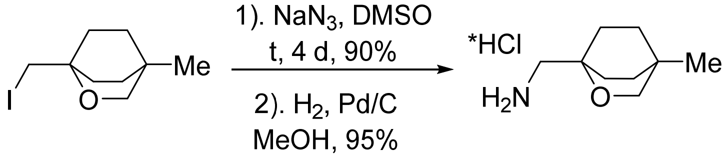

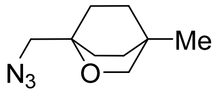

1). **1-(Azidomethyl)-4-methyl-2-oxabicyclo[2.2.2]octane (SI-16)**

To a solution of 1-(iodomethyl)-4-methyl-2-oxabicyclo[2.2.2]octane (26.60 g, 0.10 mol, 1.00 equiv) in 120 mL of DMSO was added NaN_3 (9.80 g, 0.15 mol, 1.50 equiv). The mixture was heated at 85 °C for 4 d. The mixture was diluted with water (120 mL) and extracted with MeO*t*Bu (6 × 50 mL). The combined organic phases were washed with brine (3 × 100 mL) and dried over Na_2SO_4, filtered and partially concentrated under reduced pressure. The azide was stored as a solution in MeOH. Yield: ~ 16.30 g, 0.09 mol, 90%. ^1H NMR (500 MHz, $CDCl_3$): δ 3.62 (s, 2H), 3.12 (s, 2H), 1.91 – 1.81 (m, 2H), 1.57 – 1.53 (m, 4H), 0.90 – 0.82 (m, 2H), 0.78 (s, 3H) ppm. $^{13}C\{^1H\}$ NMR (126 MHz, $CDCl_3$): δ 75.3, 71.3, 58.8, 31.5, 29.1, 29.0, 23.5 ppm. GCMS (M): 181. HRMS (ESI-TOF) *m/z*: $[M + H]^+$ calcd for $C_9H_{16}N_3O$, 182.1293; found 182.1283.

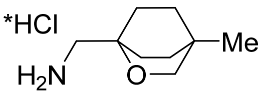

2). **(4-Methyl-2-oxabicyclo[2.2.2]octan-1-yl)methanamine hydrochloride (52)**

To a solution of azide (10.00 g, 0.055 mol, 1.00 equiv) in 150 mL of MeOH was added Pd/C (5%) (2.00 g). The mixture was stirred under H_2-ballon at room temperature overnight. The reaction was monitored by TLC. After consumption of all starting material, Pd/C was filtered out, and the reaction mixture was concentrated under reduced pressure. The residue was dissolved in cold EtOAc and 5M HCl in dioxane was added dropwise to achieve a slightly acidic pH = 2-3. The precipitate was filtered and dried. Yield: 9.96 g, 0.052 mol, 95%, white solid, m.p. = 173-174 °C. ^{1}H NMR (500 MHz, DMSO-d_6): δ 8.08 (br s, 3H), 3.52 (s, 2H), 2.68 (s, 2H), 1.89 – 1.70 (m, 2H), 1.66 – 1.41 (m, 6H), 0.74 (s, 3H) ppm. ^{13}C{^{1}H} NMR (126 MHz, DMSO-d_6): δ 74.0, 68.0, 45.6, 30.6, 28.3, 28.2, 23.1 ppm. LCMS (M+H): 156. HRMS (ESI-TOF) *m/z*: $[M + H]^+$ calcd for $C_9H_{18}NO$, 156.1388; found 156.1381.

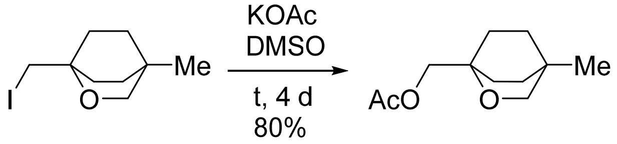

(4-Methyl-2-oxabicyclo[2.2.2]octan-1-yl)methyl acetate (SI-17)

To a solution of 1-(iodomethyl)-4-methyl-2-oxabicyclo[2.2.2]octane (26.60 g, 0.10 mol, 1.00 equiv) in DMSO (150 mL) was added KOAc (24.50 g, 0.25 mol, 2.50 equiv). The mixture was vigorously stirred overnight at 100 °C for 4 d. The mixture was diluted with water (130 mL) and extracted with MeO*t*Bu (5 × 50 mL). The combined organic layers were washed with brine (3 × 100 mL), dried over Na_2SO_4, filtered and concentrated under reduced pressure. Yield: 15.84 g, 0.08 mol, 80%, colorless oil. ^{1}H NMR (500 MHz, $CDCl_3$): δ 3.91 (s, 2H), 3.62 (s, 2H), 2.08 (s, 3H), 1.84 (t, J = 9.2 Hz, 2H), 1.60 – 1.49 (m, 6H), 0.77 (s, 3H) ppm. ^{13}C{^{1}H} NMR (151 MHz, $CDCl_3$): δ 171.2, 75.3, 69.8, 69.4, 31.4, 28.9, 28.2, 23.6, 21.1 ppm. GCMS (M): 198. HRMS (ESI-TOF) *m/z*: $[M + H]^+$ calcd for $C_{11}H_{19}O_3$, 199.1334; found 199.1327.

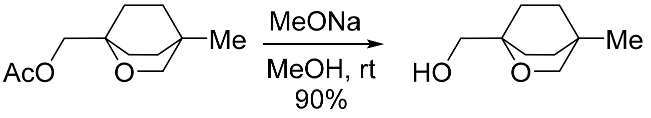

(4-Methyl-2-oxabicyclo[2.2.2]octan-1-yl)methanol (53)

To a solution of (4-methyl-2-oxabicyclo[2.2.2]octan-1-yl)methyl acetate (15.84 g, 0.08 mol, 1.00 equiv) in MeOH (200 mL) was added MeONa (4.75 g, 0.088 mol, 1.10 equiv) in portions at 5-10 °C under Ar. The mixture was vigorously stirred overnight at room temperature. After that NH_4Cl

(4.28 g, 0.08 mol, 1.00 equiv) was added, and the mixture was stirred for 2 h at room temperature. The precipitate was filtered, washed with MeO*t*Bu. The filtrate was dried over Na_2SO_4, filtered and concentrated under reduced pressure. The final product was purified by column chromatography (SiO_2, hexane/EtOAc, gradient, 4:1 to 1:3). R_f = 0.40 (hexane/EtOAc = 1:3). Yield: 11.23 g, 0.072 mol, 90%, colorless oil. ^{1}H NMR (500 MHz, $CDCl_3$): δ 3.60 (s, 2H), 3.36 (s, 2H), 2.22 (s, 1H), 1.95 – 1.80 (m, 2H), 1.62 – 1.39 (m, 6H), 0.76 (s, 3H) ppm. $^{13}C\{^1H\}$ NMR (126 MHz, $CDCl_3$): δ 75.3, 71.3, 68.8, 31.6, 29.1, 28.0, 23.6 ppm. GCMS (M): 156. HRMS (ESI-TOF) *m/z*: $[M + H]^+$ calcd for $C_9H_{17}O_2$, 157.1229; found 157.1221.

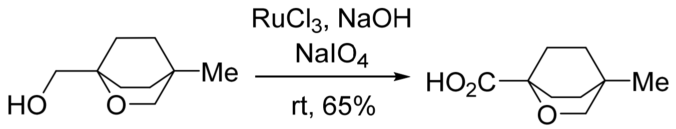

4-Methyl-2-oxabicyclo[2.2.2]octane-1-carboxylic acid (54)

To a solution of (4-methyl-2-oxabicyclo[2.2.2]octan-1-yl)methanol (15.60 g, 0.10 mol, 1.00 equiv), $RuCl_3$ (0.60 g, 0.003 mol, 0.03 equiv) and NaOH (16.00 g, 0.40 mol, 4.00 equiv) in a mixture of H_2O (180 mL), CH_3CN (120 mL) and CCl_4 (120 mL) was added $NaIO_4$ (64.30 g, 0.30 mol, 3.00 equiv) in portions at 0 °C. The mixture was vigorously stirred overnight at room temperature. Then the mixture was filtered and washed with water (1 × 100 mL). The layers were partitioned. An aqueous layer was washed with MeO*t*Bu (2 × 100 mL). The aqueous layer was acidified with 5M HCl to pH = 2 and extracted with EtOAc (4 × 100 mL). The combined organic phases were dried over Na_2SO_4, filtered and concentrated under reduced pressure. Yield: 11.05 g, 0.065 mol, 65%, white solid, m.p. = 58-59 °C. ^{1}H NMR (500 MHz, $CDCl_3$): δ 8.96 (br s, 1H), 3.72 (s, 2H), 2.09 – 1.96 (m, 4H), 1.67 – 1.52 (m, 4H), 0.81 (s, 3H) ppm. $^{13}C\{^1H\}$ NMR (126 MHz, $CDCl_3$): δ 176.2, 75.8, 72.7, 31.2, 29.4, 29.1, 23.0 ppm. GCMS (M): 170. HRMS (ESI-TOF) *m/z*: $[M + H]^+$ calcd for $C_9H_{15}O_3$, 171.1021; found 171.1014.

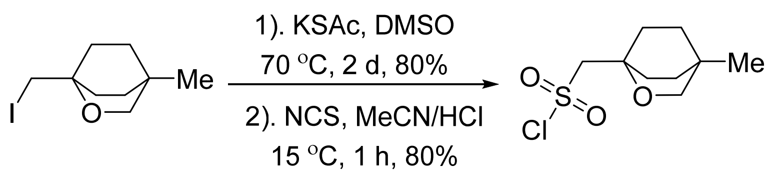

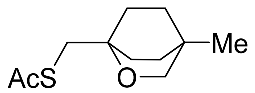

1). ***S*-((4-methyl-2-oxabicyclo[2.2.2]octan-1-yl)methyl) ethanethioate (SI-18)**

To a solution of 1-(iodomethyl)-4-methyl-2-oxabicyclo[2.2.2]octane (26.60 g, 0.10 mol, 1.00 equiv) in DMSO (200 mL) was added KSAc (17.10 g, 0.15 mol, 1.50 equiv). The mixture was vigorously stirred at 70 °C for 2 d. The mixture was diluted with water (400 mL) and extracted with MeO*t*Bu

(5 × 100 mL). The combined organic layers were washed with brine (3 × 100 mL), dried over Na_2SO_4, filtered and concentrated under reduced pressure. Yield: 17.12 g, 0.08 mol, 80%, brown oil. ^{1}H NMR (500 MHz, $CDCl_3$): δ 3.59 (s, 2H), 2.97 (s, 2H), 2.33 (s, 3H), 1.89 – 1.78 (m, 2H), 1.67 – 1.43 (m, 6H), 0.75 (s, 3H) ppm. $^{13}C\{^1H\}$ NMR (126 MHz, $CDCl_3$): δ 195.7, 75.5, 70.5, 38.3, 31.8, 30.6, 30.5, 28.8, 23.5 ppm. GCMS (M): 214. HRMS (ESI-TOF) *m/z*: $[M + H]^+$ calcd $C_{11}H_{19}O_2S$, 215.1106; found 215.1098.

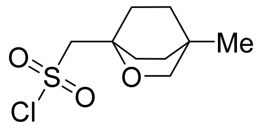

2). **(4-Methyl-2-oxabicyclo[2.2.2]octan-1-yl)methanesulfonyl chloride (55)**

S-((4-methyl-2-oxabicyclo[2.2.2]octan-1-yl)methyl) ethanethioate (17.12 g, 0.08 mol, 1.00 equiv) was dissolved in MeCN (500 mL) and 2M HCl (50 mL), and then NCS (46.99 g, 0.352 mol, 4.40 eqiuv) was added in six portions at 15-16 °C. The mixture was stirred for 1 h at room temperature and concentrated. The residue was dissolved in MeO*t*Bu (200 mL), washed with a saturated solution of $NaHCO_3$ (3 × 100 mL). The organic layer was dried over Na_2SO_4, filtered and concentrated under reduced pressure to give the title product. Yield: 15.26 g, 0.064 mol, 80%, yellow oil. ^{1}H NMR (500 MHz, $CDCl_3$): δ 3.89 (s, 2H), 3.66 (s, 2H), 2.16 – 2.07 (m, 2H), 1.90 – 1.79 (m, 2H), 1.67 – 1.51 (m, 4H), 0.79 (s, 3H) ppm. $^{13}C\{^1H\}$ NMR (126 MHz, $CDCl_3$): δ 75.4, 74.9, 70.8, 31.5, 31.0, 28.3, 23.3 ppm. HRMS (ESI-TOF) *m/z*: $[M + H]^+$ calcd $C_9H_{16}ClO_3S$, 239.0509; found 239.0502.

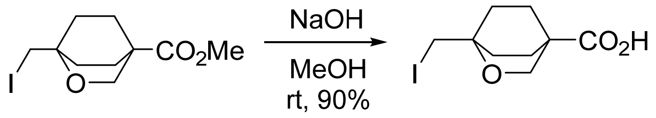

1-(Iodomethyl)-2-oxabicyclo[2.2.2]octane-4-carboxylic acid (56)

Methyl 1-(iodomethyl)-2-oxabicyclo[2.2.2]octane-4-carboxylate (31.00 g, 0.10 mol, 1.00 equiv) was dissolved in MeOH (100 mL) and NaOH (4.80 g, 0.12 mol, 1.20 equiv) in 100 mL of water was added. The solution was stirred at room temperature overnight. The solution was partially concentrated under reduced pressure. The residue was washed with MeO*t*Bu (2 × 50 mL), and then acidified with HCl to pH ~ 2. The solution was extracted with EtOAc (5 × 50 mL). The combined fractions were washed with brine, dried over Na_2SO_4, filtered and concentrated under reduced pressure. The crude product was triturated in a mixture of hexane/Et_2O = 5:1 (ca. 50 mL) to give the desired product. Yield: 26.64 g, 0.09 mol, 90%, white solid, m.p. = 65-67 °C. ^{1}H NMR (500 MHz, $CDCl_3$): δ 4.01 (s, 2H), 3.16 (s, 2H), 2.10 – 1.88 (m, 6H), 1.83 – 1.72 (m, 2H) ppm. $^{13}C\{^1H\}$ NMR

(126 MHz, $CDCl_3$): δ 180.8, 70.1, 69.7, 39.5, 30.2, 27.5, 15.3 ppm. GCMS (M): 296. HRMS (ESI-TOF) *m/z*: $[M - H]^-$ calcd for $C_9H_{12}IO_3$, 294.9831; found 294.9832.

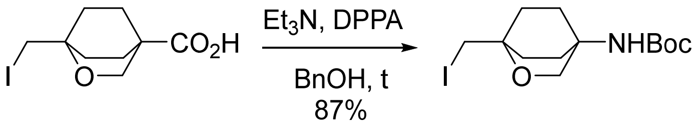

Tert-butyl (1-(iodomethyl)-2-oxabicyclo[2.2.2]octan-4-yl)carbamate (57)

To a solution of 1-(iodomethyl)-2-oxabicyclo[2.2.2]octane-4-carboxylic acid (10.36 g, 0.035 mol, 1.00 equiv) in *t*-BuOH (150 mL) was added Et_3N (5.2 mL, 0.037 mol, 1.05 equiv). The solution was heated to boiling and DPPA (9.63 g, 0.035 mol, 1.00 equiv) was added dropwise. The solution was heated at reflux overnight. After heating was stopped the mixture was concentrated under reduced pressure, and the residue was dissolved in EtOAc (150 mL). To the solution was added a saturated solution of $NaHCO_3$ (100 mL). The mixture was vigorously stirred for 2 h at room temperature. The layers were partitioned. The aqueous layer was additionally extracted with MeO*t*Bu (1 × 100 mL). The combined organic layer was dried over Na_2SO_4, filtered and concentrated under reduced pressure. The crude product was used for the next step without purification. Yield: 11.18 g, 0.03 mol, 87%, white solid, m.p. = 132-133 °C. ^1H NMR (500 MHz, $CDCl_3$): δ 4.28 (br s, 1H), 3.95 (s, 2H), 3.14 (s, 2H), 2.12 – 1.99 (m, 4H), 1.89 – 1.76 (m, 4H), 1.41 (s, 9H) ppm. $^{13}C\{^1H\}$ NMR (151 MHz, $CDCl_3$): δ 154.4, 79.7, 71.7, 69.3, 48.9, 30.8, 29.8, 28.5, 15.4 ppm. HRMS (ESI-TOF) *m/z*: $[M + H]^+$ calcd for $C_{13}H_{23}INO_3$, 368.0723; found 368.0714.

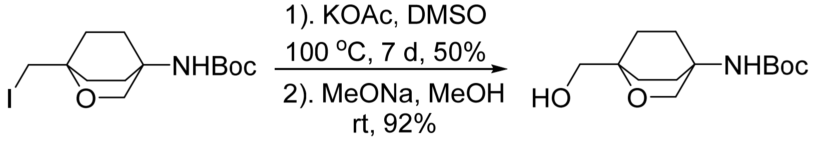

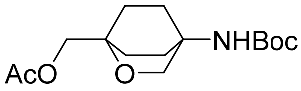

1). **(4-((Tert-butoxycarbonyl)amino)-2-oxabicyclo[2.2.2]octan-1-yl)methyl acetate (SI-19)**

To a solution of tert-butyl (1-(iodomethyl)-2-oxabicyclo[2.2.2]octan-4-yl)carbamate (36.70 g, 0.10 mol, 1.00 equiv) in DMSO (150 mL) was added KOAc (24.50 g, 0.25 mol, 2.50 equiv). The mixture was vigorously stirred overnight at 100 °C for 7 d. The mixture was diluted with water (130 mL) and extracted with MeO*t*Bu (5 × 50 mL). The combined organic layers were washed with brine (3 × 100 mL), dried over Na_2SO_4, filtered and concentrated under reduced pressure. Yield: 12.85 g, 0.05 mol, 50%, yellow solid, m.p. = 65-66 °C. ^1H NMR (500 MHz, $CDCl_3$): δ 4.32 (s, 1H), 3.95 (s, 2H), 3.92 (s, 2H), 2.08 (s, 3H), 2.07 – 2.05 (m, 2H), 1.97 – 1.82 (m, 4H), 1.67 – 1.61 (m, 2H), 1.41 (s, 9H) ppm. $^{13}C\{^1H\}$ NMR (126 MHz, $CDCl_3$): δ 171.1, 159.2, 71.3, 70.1, 68.8, 48.9, 29.3, 28.5,

28.1, 21.0 ppm. HRMS (ESI-TOF) *m/z*: $[M + Na]^+$ calcd for $C_{15}H_{25}NNaO_5$, 322.1630; found 322.1622.

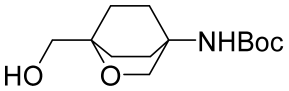

2). **Tert-butyl (1-(hydroxymethyl)-2-oxabicyclo[2.2.2]octan-4-yl)carbamate (58)**

The same procedure as for **53** was used. Yield: 3.55 g, 0.0138 mol, 92%, colorless oil. ^1H NMR (500 MHz, $CDCl_3$): δ 4.33 (br s, 1H), 3.95 (s, 2H), 3.38 (s, 2H), 2.09 – 1.96 (m, 4H), 1.81 (t, *J* = 9.7 Hz, 2H), 1.57 (td, *J* = 12.4, 3.8 Hz, 2H), 1.41 (s, 9H) ppm. $^{13}C\{^1H\}$ NMR (126 MHz, $CDCl_3$): δ 71.5, 71.3, 68.3, 49.1, 29.6, 28.5, 27.8 ppm. HRMS (ESI-TOF) *m/z*: $[M + H]^+$ calcd for $C_{13}H_{24}NO_4$, 258.1705; found 258.1694.

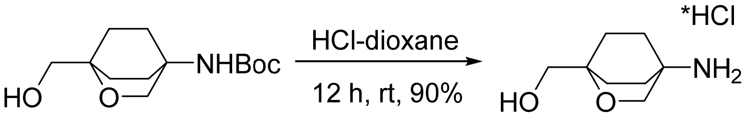

(4-Amino-2-oxabicyclo[2.2.2]octan-1-yl)methanol hydrochloride (59)

Tert-butyl (1-(hydroxymethyl)-2-oxabicyclo[2.2.2]octan-4-yl)carbamate (3.55 g, 0.0138 mol, 1.00 equiv) was added to 5M HCl in dioxane (~ 30 mL, 0.138 mol, 10 equiv) at 0 °C (ice-water bath). The mixture was stirred at room temperature overnight. The desired product was filtered and dried. Yield: 2.39 g, 0.0124 mol, 90%, white solid, m.p. = 197-199 °C. ^1H NMR (500 MHz, DMSO-d_6): δ 8.48 (br s, 3H), 3.75 (s, 2H), 3.16 (s, 2H), 1.92 – 1.59 (m, 8H) ppm. $^{13}C\{^1H\}$ NMR (151 MHz, DMSO-d_6): δ 71.7, 68.7, 66.4, 49.2, 27.5, 27.1 ppm. LCMS (M+H): 158. HRMS (ESI-TOF) *m/z*: $[M + H]^+$ calcd for $C_8H_{16}NO_2$, 158.1181; found 158.1173.

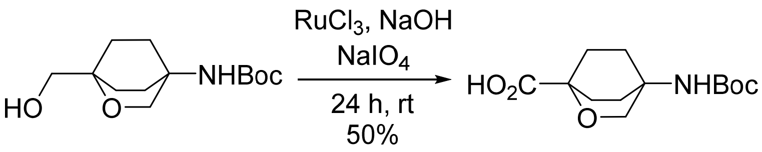

4-((Tert-butoxycarbonyl)amino)-2-oxabicyclo[2.2.2]octane-1-carboxylic acid (60)

The modified procedure for **54** was used: time = 24 h. Yield: 1.36 g, 0.005 mol, 50%, yellow solid, m.p. = 136-137 °C. ^1H NMR (500 MHz, DMSO-d_6): δ 12.46 (br s, 1H), 6.68 (br s, 1H), 3.81 (s, 2H), 2.06 – 1.84 (m, 6H), 1.85 – 1.73 (m, 2H), 1.36 (s, 9H) ppm. $^{13}C\{^1H\}$ NMR (151 MHz, DMSO-d_6): δ 173.9, 154.2, 77.7, 71.5, 70.1, 48.0, 28.9, 28.4, 28.2 ppm. HRMS (ESI-TOF) *m/z*: $[M + H]^+$ calcd for $C_{13}H_{25}N_2O_5$, 289.1763; found 289.1757.

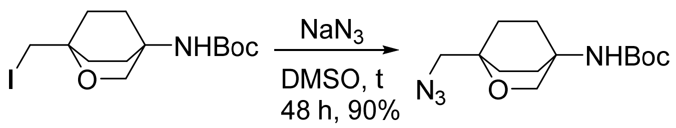

Tert-butyl (1-(azidomethyl)-2-oxabicyclo[2.2.2]octan-4-yl)carbamate (61)

To a solution of tert-butyl (1-(iodomethyl)-2-oxabicyclo[2.2.2]octan-4-yl)carbamate (36.72 g, 0.10 mol, 1.00 equiv) in 180 mL of DMSO was added NaN_3 (9.80 g, 0.15 mol, 1.50 equiv). The mixture was heated at 95 °C for 48 h. The mixture was diluted with water (400 mL) and extracted with MeO*t*Bu (6 × 100 mL). The combined organic phases were washed with brine (3 × 200 mL) and dried over Na_2SO_4, filtered and partially concentrated under reduced pressure. Yield: 25.38 g, 0.09 mol, 90%, yellow solid. ^1H NMR (500 MHz, $CDCl_3$): δ 4.30 (br s, 1H), 3.94 (s, 2H), 3.20 (s, 1H), 3.12 (s, 2H), 2.13 – 2.05 (m, 2H), 1.97 (td, *J* = 11.2, 4.8 Hz, 2H), 1.84 (t, *J* = 11.1 Hz, 2H), 1.64 (td, *J* = 12.4, 4.0 Hz, 2H), 1.41 (s, 9H), 1.18 (s, 3H) ppm. $^{13}C\{^1H\}$ NMR (151 MHz, $CDCl_3$): δ 154.4, 71.5, 71.3, 58.2, 49.6, 48.9, 29.4, 28.9, 28.5, 27.1 ppm. HRMS (ESI-TOF) *m/z*: $[M + Na]^+$ calcd for $C_{13}H_{22}N_4NaO_3$, 305.1590; found 305.1585.

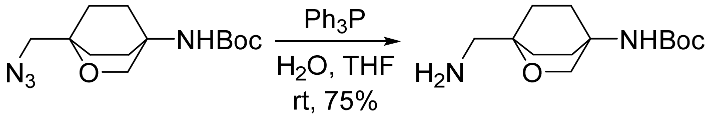

Tert-butyl (1-(aminomethyl)-2-oxabicyclo[2.2.2]octan-4-yl)carbamate (62)

A solution of tert-butyl (1-(azidomethyl)-2-oxabicyclo[2.2.2]octan-4-yl)carbamate (50.00 g, 0.177 mol, 1.00 equiv) in 300 mL of THF and H_2O (9.5 mL, 0.53 mol, 3.00 equiv) was cooled to 5 °C, and Ph_3P (55.70 g, 0.213 mol, 1.20 equiv) was added. The mixture was stirred at room temperature overnight and concentrated under reduced pressure. The crude product was purified by gradient elution chromatography (SiO_2, Ph_3P was washed with EtOAc and title product was obtained by washing it with MeOH + Et_3N (5%)). Yield: 33.28 g, 0.13 mol, 75%, white solid, m.p. = 115-116 °C. ^1H NMR (500 MHz, $CDCl_3$): δ 4.34 (br s, 1H), 3.92 (s, 2H), 2.57 (s, 2H), 2.11 – 2.03 (m, 4H), 1.92 (td, *J* = 11.1, 4.9 Hz, 2H), 1.87 – 1.78 (m, 2H), 1.57 (td, *J* = 12.5, 4.1 Hz, 2H), 1.40 (s, 9H) ppm. $^{13}C\{^1H\}$ NMR (151 MHz, $CDCl_3$): δ 154.4, 79.5, 71.3, 71.2, 49.6, 49.0, 29.7, 28.7, 28.5 ppm. LCMS (M+H): 257. HRMS (ESI-TOF) *m/z*: $[M + H]^+$ calcd for $C_{13}H_{25}N_2O_3$, 257.1865; found 257.1859.

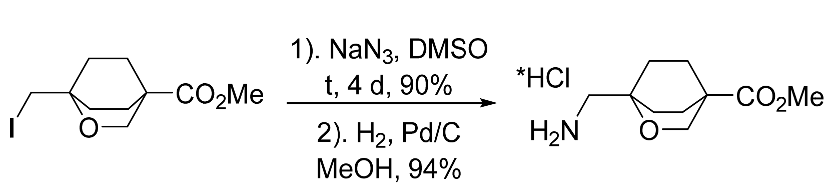

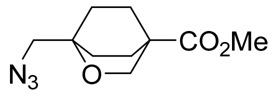

1). **Methyl 1-(azidomethyl)-2-oxabicyclo[2.2.2]octane-4-carboxylate (63)**

To a solution of methyl 1-(iodomethyl)-2-oxabicyclo[2.2.2]octane-4-carboxylate (31.00 g, 0.10 mol, 1.00 equiv) in 180 mL of DMSO was added NaN_3 (9.80 g, 0.15 mol, 1.50 equiv). The mixture was heated at 95 °C for 48 h. The mixture was diluted with water (400 mL) and extracted with MeO*t*Bu (6 × 100 mL). The combined organic phases were washed with brine (3 × 200 mL) and dried over Na_2SO_4, filtered and partially concentrated under reduced pressure. Yield: 20.25 g, 0.09 mol, 90%, yellow oil. ^{1}H NMR (500 MHz, $CDCl_3$): δ 3.98 (s, 2H), 3.66 (s, 3H), 3.14 (s, 2H), 2.06 – 1.87 (m, 6H), 1.66 – 1.52 (m, 2H) ppm. ^{13}C{^{1}H} NMR (151 MHz, $CDCl_3$): δ 175.1, 71.7, 69.9, 58.4, 52.1, 39.6, 28.4, 27.2 ppm. GCMS (M): 225. HRMS (ESI-TOF) *m/z*: $[M + H]^+$ calcd for $C_{10}H_{16}N_3O_3$, 226.1192; found 226.1175.

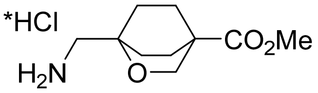

2). **Methyl 1-(aminomethyl)-2-oxabicyclo[2.2.2]octane-4-carboxylate hydrochloride (64)**

To a solution of azide (20.25 g, 0.09 mol, 1.00 equiv) in 300 mL of MeOH was added Pd/C (5%) (2.00 g). The mixture was stirred under H_2-ballon at room temperature overnight. The reaction was monitored by TLC. After consumption of all starting material, Pd/C was filtered out, and the reaction mixture was concentrated under reduced pressure. The residue was dissolved in cold EtOAc and 5M HCl in dioxane was added dropwise to achieve a slightly acidic pH = 2-3. The precipitate was filtered and dried. Yield: 20.01 g, 0.085 mol, 94%, white solid, m.p. = 172-173 °C. ^{1}H NMR (500 MHz, DMSO-d_6): δ 8.12 (br s, 3H), 3.86 (s, 2H), 3.60 (s, 3H), 2.73 (s, 2H), 1.93 – 1.77 (m, 6H), 1.69 – 1.56 (m, 2H) ppm. ^{13}C{^{1}H} NMR (126 MHz, DMSO-d_6): δ 174.2, 68.9, 68.6, 51.8, 45.2, 38.7, 27.5, 26.4 ppm. LCMS (M+H): 200. HRMS (ESI-TOF) *m/z*: $[M + H]^+$ calcd for $C_{10}H_{18}NO_3$, 200.1287; found 200.1283.

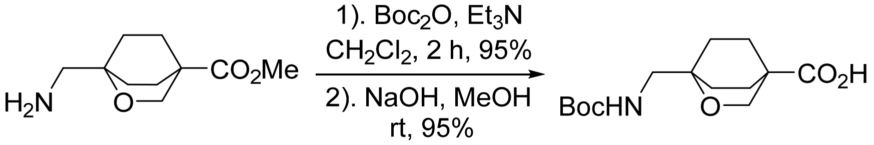

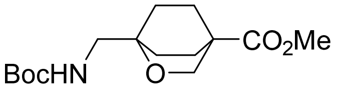

1). **Methyl 1-(((tert-butoxycarbonyl)amino)methyl)-2-oxabicyclo[2.2.2]octane-4-carboxylate (SI-20)**

Methyl 1-(aminomethyl)-2-oxabicyclo[2.2.2]octane-4-carboxylate (19.90 g, 0.10 mol, 1.00 equiv) was dissolved in CH_2Cl_2 (250 mL) and Et_3N (10.10 g, 0.10 mol, 1.00 equiv) was added to the mixture. Boc_2O (21.80 g, 0.10 mol, 1.00 equiv) in CH_2Cl_2 (25 mL) was added dropwise at 10 °C

(the mixture was cooled in a water bath). The solution was stirred for 2 h at room temperature. The mixture was washed with 0.5M HCl (1 × 100 mL), water (1 × 100 mL), brine (1 × 100 mL) and dried over Na_2SO_4, filtered and concentrated under reduced pressure to give the desired product as a colorless oil. Yield: 28.40 g, 0.095 mol, 95%. ^{1}H NMR (500 MHz, $CDCl_3$): δ 4.83 (br s, 1H), 3.93 (s, 2H), 3.65 (s, 3H), 3.07 (d, J = 5.7 Hz, 1H), 1.97 – 1.82 (m, 6H), 1.60 – 1.53 (m, 2H), 1.43 (s, 9H) ppm. ^{13}C{^{1}H} NMR (151 MHz, $CDCl_3$): δ 175.3, 156.3, 79.3, 71.2, 69.9, 52.0, 47.9, 39.6, 28.5, 28.2, 27.3 ppm. HRMS (ESI-TOF) *m/z*: $[M + Na]^+$ calcd for $C_{15}H_{25}NNaO_5$, 322.1630; found 322.1621.

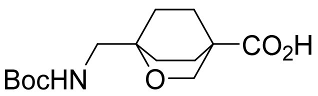

2). **1-(((Tert-butoxycarbonyl)amino)methyl)-2-oxabicyclo[2.2.2]octane-4-carboxylic acid (65)**. Methyl 1-(((tert-butoxycarbonyl)amino)methyl)-2-oxabicyclo[2.2.2]octane-4-carboxylate (28.40 g, 0.095 mol, 1.00 equiv) was dissolved in MeOH (300 mL) and NaOH (4.56 g, 0.114 mol, 1.20 equiv) in 100 mL of water was added. The solution was stirred at room temperature overnight. The solution was partially concentrated under reduced pressure. The residue was washed with MeO*t*Bu (2 × 100 mL), and then acidified with HCl to achieve a slightly acidic pH = 2-3. The solution was extracted with EtOAc (5 × 100 mL). The combined fractions were washed with brine, dried over Na_2SO_4, filtered and concentrated under reduced pressure. The crude product was triturated in a mixture of hexane:Et_2O = 5:1 (ca. 50 mL) to give the desired product. Yield: 25.72 g, 0.09 mol, 95%, white solid, m.p. = 106-107 °C. ^{1}H NMR (500 MHz, $CDCl_3$): δ 4.89 (s, 1H), 3.95 (s, 2H), 3.09 (d, J = 5.5 Hz, 2H), 2.08 – 1.78 (m, 6H), 1.64 – 1.54 (m, 2H), 1.43 (s, 9H) ppm. ^{13}C{^{1}H} NMR (151 MHz, $CDCl_3$): δ 180.4, 156.4, 79.5, 71.4, 69.6, 47.8, 39.4, 28.5, 28.1, 27.2 ppm. HRMS (ESI-TOF) *m/z*: $[M + Na]^+$ calcd for $C_{14}H_{23}NNaO_5$, 308.1474; found 308.1466.

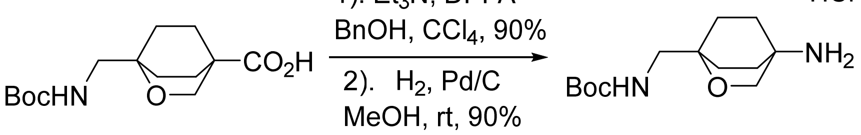

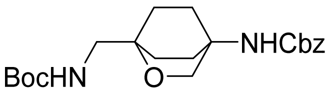

1). **Tert-butyl ((4-(((benzyloxy)carbonyl)amino)-2-oxabicyclo[2.2.2]octan-1-yl)methyl)carbamate (SI-21)**

To a solution of 1-(((tert-butoxycarbonyl)amino)methyl)-2-oxabicyclo[2.2.2]octane-4-carboxylic acid (25.72 g, 0.09 mol, 1.00 equiv) in 250 mL of CCl_4 was added Et_3N (13.2 mL, 0.0945 mol, 1.05 equiv). The solution was heated to boiling and DPPA (24.75 g, 0.09 mol, 1.00 equiv) was added

dropwise. The solution was heated at reflux for 2 h. Then heating was stopped and BnOH (11.66 g, 0.108 mol, 1.20 equiv) was added in one portion. The solution was heated at reflux overnight. The mixture was vigorously stirred for 2 h at room temperature and diluted with water (250 mL). The layers were partitioned. The aqueous layer was additionally extracted with EtOAc (1 × 150 mL). The combined organic layers were dried over Na_2SO_4, filtered and concentrated under reduced pressure. Yield: 31.59 g, 0.081 mol, 90%, colorless oil. The product was used for the next step without purification. ^{1}H NMR (500 MHz, DMSO-d_6): δ 7.40 – 7.29 (m, 5H), 7.13 (br s, 1H), 6.63 (t, J = 5.8 Hz, 1H), 4.95 (s, 2H), 3.76 (s, 2H), 2.85 (d, J = 6.1 Hz, 2H), 1.91 (br s, 2H), 1.78 – 1.57 (m, 6H), 1.36 (s, 9H) ppm. ^{13}C{^{1}H} NMR (126 MHz, DMSO-d_6): δ 155.8, 154.6, 137.0, 128.3, 127.8, 77.5, 70.7, 69.9, 64.9, 48.4, 47.2, 28.6, 28.3, 28.2 ppm. HRMS (ESI-TOF) *m/z*: $[M + Na]^+$ calcd for $C_{21}H_{30}N_2NaO_5$, 413.2052; found 413.2040.

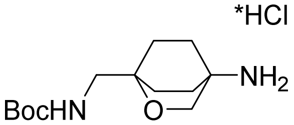

2). **Tert-butyl ((4-amino-2-oxabicyclo[2.2.2]octan-1-yl)methyl)carbamate hydrochloride (66)**. A round bottomed flask was charged with compound tert-butyl ((4-(((benzyloxy)carbonyl)amino)-2-oxabicyclo[2.2.2]octan-1-yl)methyl)carbamate (31.59 g, 0.081 mol, 1.00 equiv) and Pd/C (5%) (3.00 g) in MeOH (300 mL). The mixture was hydrogenated under a rubber ball filled with H_2 at room temperature overnight. After consumption of all starting material, Pd/C was filtered out, and the reaction mixture was concentrated under reduced pressure. The residue was dissolved in cold EtOAc (ca. 250 mL) and 5M HCl in dioxane was added dropwise to achieve slightly acidic pH. The precipitate was filtered and dried. Yield: 21.32 g, 0.0729 mol, 90%, white solid. ^{1}H NMR (500 MHz, DMSO-d_6): δ 8.47 (s, 3H), 6.71 (t, J = 6.0 Hz, 1H), 3.74 (s, 2H), 2.87 (d, J = 6.2 Hz, 2H), 1.91 – 1.78 (m, 4H), 1.76 – 1.62 (m, 4H), 1.36 (s, 9H) ppm. ^{13}C{^{1}H} NMR (126 MHz, DMSO-d_6): δ 155.8, 77.6, 71.3, 68.7, 48.9, 46.9, 28.2, 27.8, 27.4 ppm. LCMS (M+H): 257. HRMS (ESI-TOF) *m/z*: $[M + H]^+$ calcd for $C_{13}H_{25}N_2O_3$, 257.1865; found 257.1860.

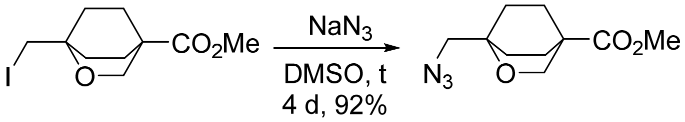

Methyl 1-(azidomethyl)-2-oxabicyclo[2.2.2]octane-4-carboxylate (SI-22)

To a solution of methyl 1-(iodomethyl)-2-oxabicyclo[2.2.2]octane-4-carboxylate (3.10 g, 0.01 mol, 1.00 equiv) in 60 mL of DMSO was added NaN_3 (0.98 g, 0.015 mol, 1.50 equiv). The mixture was heated at 85 °C for 4 d. The mixture was diluted with water (100 mL) and extracted with MeO*t*Bu (6 × 20 mL). The combined organic phases were washed with brine (3 × 50 mL) and dried over

Na_2SO_4, filtered and concentrated under reduced pressure. Yield: 2.07 g, 0.0092 mol, 92%, yellow oil. ^1H NMR (500 MHz, $CDCl_3$): δ 3.98 (s, 2H), 3.66 (s, 3H), 3.14 (s, 2H), 2.03 – 1.88 (m, 6H), 1.64 – 1.53 (m, 2H) ppm. $^{13}C\{^1H\}$ NMR (151 MHz, $CDCl_3$): δ 175.1, 71.7, 69.9, 58.4, 52.1, 39.6, 28.4, 27.2 ppm. GCMS (M): 225. HRMS (ESI-TOF) *m/z*: $[M + H]^+$ calcd for $C_{10}H_{16}N_3O_3$, 226.1192; found 226.1175.

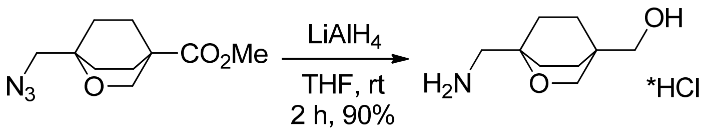

(1-(Aminomethyl)-2-oxabicyclo[2.2.2]octan-4-yl)methanol hydrochloride (67)

A methyl 1-(azidomethyl)-2-oxabicyclo[2.2.2]octane-4-carboxylate (2.07 g, 0.0092 mol, 1.00 equiv) in THF (50 mL) was added dropwise to a suspension of $LiAlH_4$ (0.44 g, 0.0115 mol, 1.25 equiv) in THF (50 mL) at 0-10 °C. Then the reaction mixture was warmed to room temperature and left stirring for 2 h. The mixture was quenched with water (0.3 mL, 0.0184 mol, 2.00 equiv) and a sat. aq. solution of NaOH (0.74 g, 0.0184 mol, 2.00 equiv). The mixture was filtered through a thick pad of Na_2SO_4 (ca. 100 g). The solid residue was washed with hot THF (2 × 50 mL). The mixture was concentrated under reduced pressure. The residue was dissolved in cold EtOAc and 5M HCl in dioxane was added dropwise to achieve a slightly acidic pH = 2-3. The precipitate was filtered and dried. Yield: 1.72 g, 0.00828 mol, 90%, white solid, m.p. = 157-159 °C. ^1H NMR (500 MHz, DMSO-d_6): δ 8.05 (br s, 3H), 4.57 (br s, 1H), 3.61 (s, 2H), 3.09 (s, 2H), 2.68 (s, 2H), 1.84 – 1.68 (m, 2H), 1.60 – 1.47 (m, 4H), 1.47 – 1.33 (m, 2H) ppm. $^{13}C\{^1H\}$ NMR (126 MHz, $CDCl_3$): δ 71.0, 68.4, 65.8, 45.5, 33.5, 27.9, 25.8 ppm. LCMS (M+H): 172. HRMS (ESI-TOF) *m/z*: $[M + H]^+$ calcd for $C_9H_{18}NO_2$, 172.1338; found 172.1330.

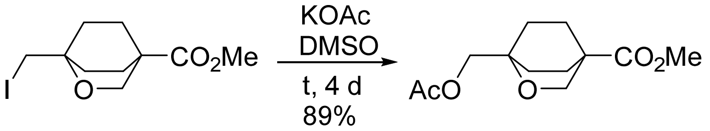

Methyl 1-(acetoxymethyl)-2-oxabicyclo[2.2.2]octane-4-carboxylate (68)

To a solution of methyl 1-(iodomethyl)-2-oxabicyclo[2.2.2]octane-4-carboxylate (3.10 g, 0.01 mol, 1.00 equiv) in DMSO (50 mL) was added KOAc (2.45 g, 0.025 mol, 2.50 equiv). The mixture was vigorously stirred overnight at 100 °C for 4 d. The mixture was diluted with water (100 mL) and extracted with MeO*t*Bu (5 × 20 mL). The combined organic layers were washed with brine (3 × 30 mL), dried over Na_2SO_4, filtered and concentrated under reduced pressure. Yield: 2.15 g, 0.0089 mol, 89%, yellow oil. ^1H NMR (500 MHz, $CDCl_3$): δ 3.98 (s, 2H), 3.93 (s, 2H), 3.66 (s, 3H), 2.09 (s, 3H), 2.01 – 1.90 (m, 6H), 1.63 – 1.56 (m, 2H) ppm. $^{13}C\{^1H\}$ NMR (126 MHz, $CDCl_3$): δ 175.2,

171.1, 70.3, 69.9, 68.9, 52.1, 39.6, 27.6, 27.1, 21.0 ppm. HRMS (ESI-TOF) *m/z*: $[M + H]^+$ calcd for $C_{12}H_{19}O_5$, 243.1232; found 243.1225.

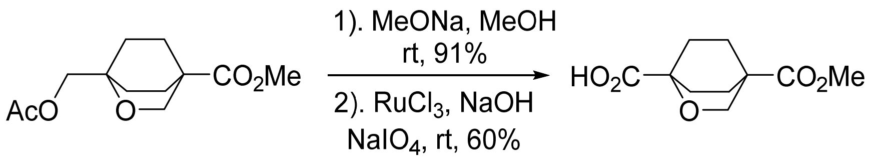

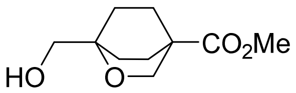

1). **Methyl 1-(hydroxymethyl)-2-oxabicyclo[2.2.2]octane-4-carboxylate (SI-23)**

To a solution of methyl 1-(acetoxymethyl)-2-oxabicyclo[2.2.2]octane-4-carboxylate (2.15 g, 0.0089 mol, 1.00 equiv) in MeOH (50 mL) was added MeONa (5.29 g, 0.00979 mol, 1.10 equiv) in three portions at 5-10 °C under Ar. The mixture was vigorously stirred overnight at room temperature. After that NH_4Cl (0.48 g, 0.0089 mol, 1.00 equiv) was added, and the mixture was stirred for 2 h at room temperature. The precipitate was filtered, washed with MeO*t*Bu (2 × 50 mL). The filtrate was dried over Na_2SO_4, filtered and concentrated under reduced pressure. The crude product was used for the next step without purification. Yield: 1.62 g, 0.0081 mol, 91%, colorless oil. ^{1}H NMR (500 MHz,$CDCl_3$): δ 3.97 (s, 2H), 3.66 (s, 3H), 3.39 (s, 2H), 2.02 – 1.89 (m, 6H), 1.58 – 1.45 (m, 2H) ppm. $^{13}C\{^1H\}$ NMR (151 MHz, $CDCl_3$): δ 175.3, 71.8, 69.9, 68.4, 52.0, 39.8, 27.3, 27.2 ppm. HRMS (ESI-TOF) *m/z*: $[M + H]^+$ calcd for $C_{10}H_{17}O_4$, 201.1127; found 201.1112.

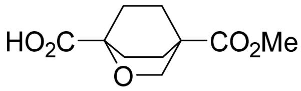

2). **4-(Methoxycarbonyl)-2-oxabicyclo[2.2.2]octane-1-carboxylic acid (69)**

The same procedure as for **54** was used. Yield: 1.05 g, 0.0049 mol, 60%, white solid, m.p. = 86-87 °C. ^{1}H NMR (500 MHz, $CDCl_3$): δ 8.66 (br s, 1H), 4.08 (s, 2H), 3.68 (s, 3H), 2.12 – 1.98 (m, 8H) ppm. $^{13}C\{^1H\}$ NMR (101 MHz, $CDCl_3$): δ 176.0, 174.4, 72.8, 70.5, 52.3, 39.6, 28.7, 27.0 ppm. LCMS $(M-H)^-$: 214. HRMS (ESI-TOF) *m/z*: $[M + H]^+$ calcd for $C_{10}H_{15}O_5$, 215.0919; found 215.0914.

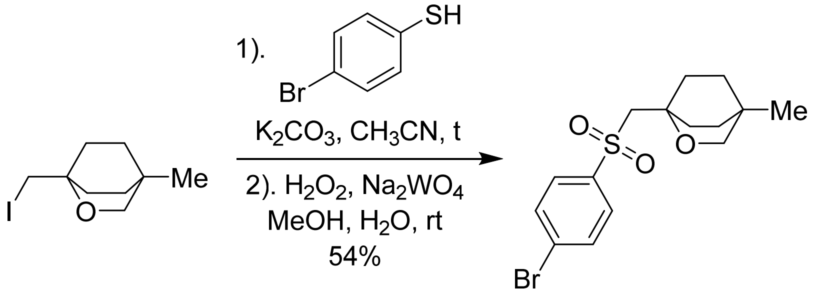

1-(((4-Bromophenyl)sulfonyl)methyl)-4-methyl-2-oxabicyclo[2.2.2]octane (70)

A solution of 4-bromobenzenethiol (0.78 g, 4.10 mmol, 1.08 equiv), 1-(iodomethyl)-4-methyl-2-oxabicyclo[2.2.2]octane (1.00 g, 3.80 mmol, 1.00 equiv) and K_2CO_3 (0.66 g, 4.70 mmol, 1.24 equiv) in 20 mL of CH_3CN was stirred at 90 °C (in an oil bath with a thermocouple) for 12 h. The mixture was cooled to room temperature and diluted with water (50 mL). The solution was extracted with EtOAc (3 × 50 mL). The combined organic layers were washed with brine (1 × 50 mL), dried over Na_2SO_4, filtered and concentrated under reduced pressure. The product (0.91 g, 2.78 mmol) was dissolved in a mixture of MeOH (5 mL) and H_2O (5 mL), and then Na_2WO_4 (41 mg, 0.14 mmol, 5 mol%) and H_2O_2 (35% w/w, 1 mL, 10.29 mmol, 3.70 equiv) were added to the mixture. The resulting mixture was stirred at room temperature overnight. After the reaction was finished (tlc-control), the solution was concentrated under reduced pressure. The residue was cooled with liquid nitrogen (~ -10 °C), and the formed precipitate was filtered. Yield over 2 steps: 0.74 g, 2.05 mmol, 54%, white solid, m.p. = 120-121 °C, purity ~ 90%. The analytically pure sample was obtained by high performance liquid chromatography: Rt = 1-6 min, 40-70%, H_2O/CH_3CN, flow 30 mL/min (loading pump 4 mL/min). ^1H NMR (500 MHz, DMSO-d_6): δ 7.86 – 7.73 (m, 4H), 3.46 (s, 2H), 3.32 (s, 2H), 2.00 – 1.90 (m, 2H), 1.74 – 1.64 (m, 2H), 1.50 – 1.36 (m, 4H), 0.68 (s, 3H) ppm. $^{13}C\{^1H\}$ NMR (151 MHz, DMSO-d_6): δ 140.9, 131.9, 129.8, 127.3, 73.8, 69.3, 62.8, 30.9, 30.2, 27.7, 23.1 ppm. LCMS $(M+H)^+$: 360. HRMS (ESI-TOF) *m/z*: $[M + H]^+$ calcd for $C_{15}H_{20}{}^{81}BrO_3S$, 361.0296; found 361.0290.

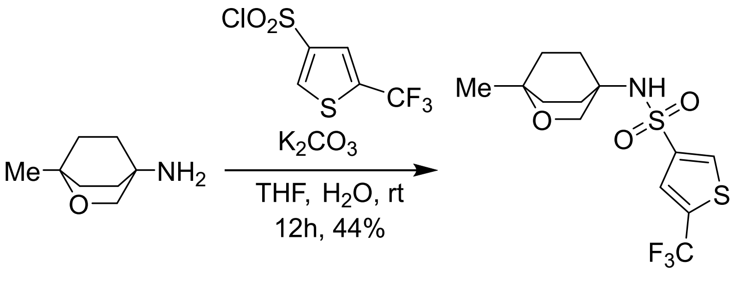

***N*-(1-methyl-2-oxabicyclo[2.2.2]octan-4-yl)-5-(trifluoromethyl)thiophene-3-sulfonamide (71)**

To a solution of 1-methyl-2-oxabicyclo[2.2.2]octan-4-amine (200 mg, 1.42 mmol, 1.00 equiv) in THF (8 mL) and water (1.6 mL) was added K_2CO_3 (196 mg, 1.42 mmol, 1.00 equiv) followed by 5-(trifluoromethyl)thiophene-3-sulfonyl chloride (355 mg, 1.42 mmol, 1.00 equiv). The resulting reaction mixture was stirred at ambient temperature overnight. The reaction mixture was diluted with H_2O (2 mL) and extracted with EtOAc (2 × 3 mL). The combined organic layers were washed with brine (1 × 2 mL), dried over Na_2SO_4 and concentrated under reduced pressure. The obtained crude product was purified by high performance liquid chromatography: Rt = 1-6 min, 35-75%, H_2O/CH_3CN/0.1 TFA, flow 30 mL/min (loading pump 4 mL/min). Yield: 220 mg, 0.62 mmol, 44%, white solid. ^1H NMR (500 MHz, DMSO-d_6): δ 8.50 (d, J = 1.5 Hz, 1H), 7.89 (s, 1H), 7.87 (br

s, 1H), 3.62 (s, 1H), 1.84 – 1.52 (m, 8H), 0.94 (s, 3H) ppm. $^{13}C\{^1H\}$ NMR (151 MHz, DMSO-d$_6$): δ 144.5, 133.9, 131.5 (q, J = 38.5 Hz), 127.6 (q, J = 3.7 Hz), 121.6 (q, J = 269.3 Hz), 70.9, 68.0, 52.0, 32.1, 29.6, 25.9 ppm. $^{19}F\{^1H\}$ NMR (376 MHz, DMSO-d$_6$): δ -55.0 (s) ppm. LCMS $(M-H)^-$: 354. HRMS (ESI-TOF) *m/z*: $[M + H]^+$ calcd for $C_{13}H_{17}F_3NO_3S_2$, 356.0602; found 356.0592.

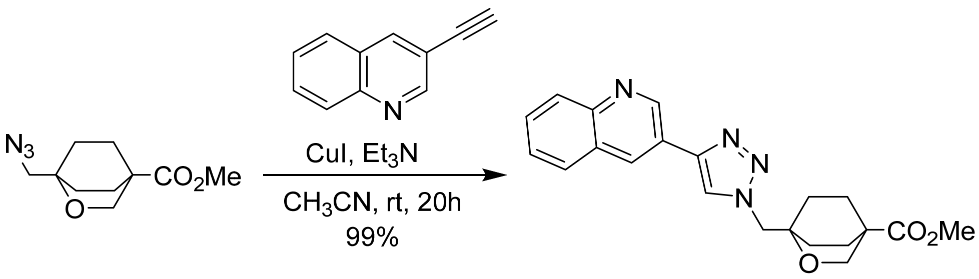

Methyl 1-((4-(quinolin-3-yl)-1*H*-1,2,3-triazol-1-yl)methyl)-2-oxabicyclo[2.2.2]octane-4-carboxylate (72)

To a solution of methyl 1-(azidomethyl)-2-oxabicyclo[2.2.2]octane-4-carboxylate (150 mg, 0.67 mmol, 1.00 equiv) and 3-ethynylquinoline (133 mg, 0.87 mmol, 1.30 equiv) in CH_3CN (5 mL) were added CuI (13 mg, 0.07 mmol, 0.10 equiv) and Et_3N (81 mg, 0.80 mmol, 1.20 equiv). The resulting solution was stirred for 20 h at room temperature, and then concentrated in *vacuo* (reaction was monitored by mass chromatography). The residue was diluted with EtOAc (20 mL), washed with a saturated aq. solution of NH_4Cl (1 × 10 mL) and brine (1 × 10 mL), dried over Na_2SO_4 and concentrated in *vacuo* to obtain crude product (280 mg, 0.66 mmol, 99% yield) with ~ 90% purity, white solid. The analytically pure sample was obtained by high performance liquid chromatography: Rt = 1-6 min, 45-70%, H_2O/CH_3OH, flow 30 mL/min (loading pump 4 mL/min). ^1H NMR (500 MHz, DMSO-d$_6$): δ 9.44 (s, 1H), 8.83 (s, 1H), 8.71 (s, 1H), 8.04 (t, J = 7.3 Hz, 1H), 7.77 (t, J = 7.6 Hz, 1H), 7.65 (t, J = 7.5 Hz, 1H), 4.45 (s, 2H), 3.86 (s, 2H), 3.58 (s, 3H), 1.94 – 1.62 (m, 8H) ppm. $^{13}C\{^1H\}$ NMR (151 MHz, DMSO-d$_6$): δ 174.3, 148.2, 130.9, 129.5, 128.8, 128.3, 127.2, 123.7, 70.3, 69.0, 56.5, 51.8, 38.8, 27.7, 26.5 ppm. LCMS $(M+H)^+$: 379. HRMS (ESI-TOF) *m/z*: $[M + H]^+$ calcd for $C_{21}H_{23}N_4O_3$, 379.1770; found 379.1765.

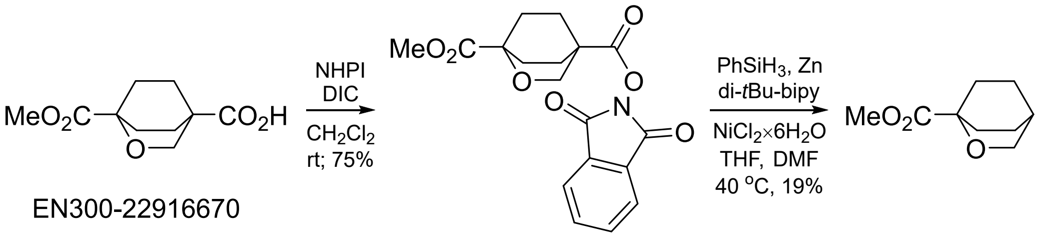

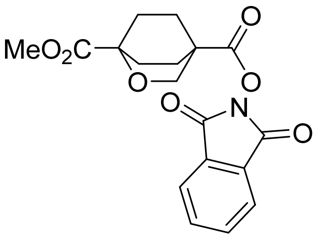

4-(1,3-Dioxoisoindolin-2-yl) 1-methyl 2-oxabicyclo[2.2.2]octane-1,4-dicarboxylate (74)

A round-bottom flask equipped with a stir bar was charged with a commercially available 1-(methoxycarbonyl)-2-oxabicyclo[2.2.2]octane-4-carboxylic acid (1.00 g, 0.0047 mol, 1.00 equiv), *N*-hydroxy-phthalimide (0.85 g, 0.0052 mol, 1.10 equiv) and DMAP (0.057 g, 0.00047 mol, 0.10 equiv). CH_2Cl_2 was added (10 mL) followed by DIC (0.65 g, 0.0052 mol, 1.10 equiv), and the mixture was allowed to stir vigorously for 2 h. The mixture was filtered over Celite, and rinsed with additional CH_2Cl_2/Et_2O (10 mL). The solvent was removed under reduced pressure, and purification by column chromatography (CH_2Cl_2) afforded the corresponding redox-active ester. Yield: 1.27 g, 0.0035 mol, 75%, white solid, m.p. = 133-134 °C. ^{1}H NMR (500 MHz, DMSO-d$_6$): δ 8.02 – 7.91 (m, 4H), 4.07 (s, 2H), 3.66 (s, 3H), 2.21 – 1.93 (m, 8H) ppm. ^{13}C{^{1}H} NMR (151 MHz, DMSO-d$_6$): δ 172.2, 170.8, 161.6, 135.6, 128.1, 124.0, 72.3, 68.0, 52.0, 38.3, 28.0, 26.3 ppm. LCMS $(M+H)^+$: 360. HRMS (ESI-TOF) *m/z*: $[M + NH_4]^+$ calcd for $C_{18}H_{21}N_2O_7$, 377.1349; found 377.1341.

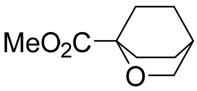

Methyl 2-oxabicyclo[2.2.2]octane-1-carboxylate (75)

A two-neck round bottom flask was charged with $NiCl_2$•$6H_2O$ (0.055 g, 0.233 mmol, 10 mol %) and 4,4'-di-tert-butyl-2,2'-bipyridine (0.125 g, 0.467 mmol, 20 mol %). The flask was equipped with a reflux condenser and rubber septum, and the apparatus was purged with Ar from a balloon for 5 min. DMF (2.3 mL, anhydrous) was added, and the mixture was stirred for 10 minutes. THF (10 mL) and *i*-PrOH (1.2 mL) were then added, followed by 4-(1,3-dioxoisoindolin-2-yl) 1-methyl 2-oxabicyclo[2.2.2]octane-1,4-dicarboxylate (834 mg, 2.32 mmol, 1.00 equiv) and Zn powder (0.076 g, 1.167 mmol, 0.5 equiv). Immediately following the addition of the Zn powder, $PhSiH_3$ (neat, 0.431 mL, 3.50 mmol, 1.50 equiv) was added dropwise. Upon completion of the addition of $PhSiH_3$, the reaction mixture was placed in a preheated 40 °C oil bath and stirred for 1 h. After 1 h the mixture was allowed to cool to ambient temperature. H_2O (distilled) and sat. aq. NH_4Cl were added (1:1 v/v 50 mL), and the mixture was transferred to a separatory funnel. The mixture was extracted with EtOAc (3 × 50 mL), and the organic extracts were filtered over a small plug of silica gel. The filtrate was concentrated on a rotary evaporator under reduced pressure at 40 °C, and the crude product was purified by flash column chromatography (SiO_2, hexane/EtOAc, 4:1). Yield: 75 mg, 0.44 mmol, 19%, colorless oil. ^{1}H NMR (500 MHz, $CDCl_3$): δ 4.00 (s, 2H), 3.74 (s, 3H), 2.15 –

2.03 (m, 2H), 2.03 – 1.94 (m, 2H), 1.89 – 1.80 (m, 2H), 1.79 – 1.67 (m, 3H) ppm. $^{13}C\{^1H\}$ NMR (151 MHz, $CDCl_3$): δ 174.3, 72.6, 70.5, 52.4, 29.9, 25.5, 24.1 ppm. GCMS (M): 170. HRMS (ESI-TOF) *m/z*: $[M + Na]^+$ calcd for $C_9H_{14}NaO_3$, 193.0841; found 193.0826.

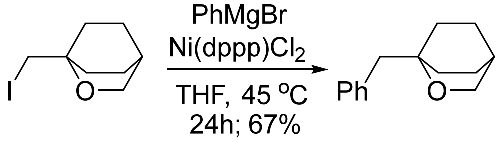

1-Benzyl-2-oxabicyclo[2.2.2]octane (76)

To a solution of [3-(diphenylphosphanyl)propyl]diphenylphosphane dichloronickel (0.21 g 0.38 mmol, 0.1 equiv) and 1-(iodomethyl)-2-oxabicyclo[2.2.2]octane (1.00 g, 3.96 mmol, 1.00 equiv) in 10 mL of THF was added bromo(phenyl)magnesium (1M in THF) (15.9 mL 15.88 mmol, 4.00 equiv) at room temperature. The resulting mixture was stirred at 45 °C for 24 h. The solution was cooled to room temperature, and a mixture of 50 mL of a saturated aq. solution of ammonium chloride and 100 mL of MeO*t*Bu was added. The organic layer was separated, dried over Na_2SO_4, filtered and concentrated under reduced pressure. The final product was purified by column chromatography (SiO_2, hexane/EtOAc, 4:1). Yield: 0.54 g, 2.65 mmol, 67%, colorless oil. ^1H NMR (500 MHz, DMSO-d_6): δ 7.24 (t, *J* = 7.3 Hz, 2H), 7.17 (t, *J* = 7.4 Hz, 1H), 7.14 (d, *J* = 7.0 Hz, 2H), 3.74 (s, 2H), 2.57 (s, 2H), 1.73 – 1.41 (m, 9H) ppm. $^{13}C\{^1H\}$ NMR (126 MHz, DMSO-d_6): δ 137.6, 130.5, 127.6, 125.8, 70.0, 69.0, 46.1, 30.6, 25.4, 24.1 ppm. HRMS (ESI-TOF) *m/z*: $[M + H]^+$ calcd for $C_{14}H_{19}O$, 203.1436; found 203.1430.

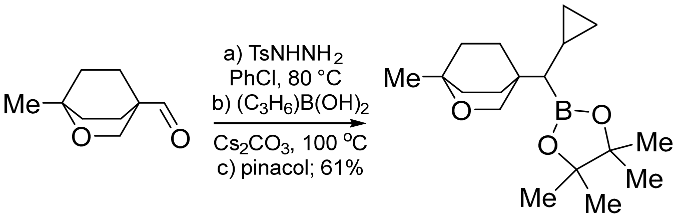

2-(Cyclopropyl(1-methyl-2-oxabicyclo[2.2.2]octan-4-yl)methyl)-4,4,5,5-tetramethyl-1,3,2-dioxaborolane (77)

A screw-capped culture tube was charged with 1-methyl-2-oxabicyclo[2.2.2]octane-4-carbaldehyde (0.61 g, 3.98 mmol, 1.00 equiv), *p*-toluenesulfonyl hydrazide (2.22 g, 11.94 mmol, 3.00 equiv) and chlorobenzene (40 mL). The mixture was then stirred at 80 °C (in an oil bath with a thermocouple) until the completion of the reaction showed by TLC analysis (usually 0.5 – 1 h). Cs_2CO_3 (3.89 g, 11.94 mmol, 3.00 equiv) and cyclopropylboronic acid (1.39 g, 11.94 mmol, 3.00 equiv) were added, and the headspace of the tube was purged with a gentle stream of argon for approximately 10 seconds. The tube was tightly sealed, and the system was stirred at 100 °C under argon atmosphere for 5 hours (in an oil bath with a thermocouple). After cooling the system to room temperature, 2-cyclopropyl-4,4,5,5-tetramethyl-1,3,2-dioxaborolane (3.34 g, 19.90 mmol, 5.00 equiv) was added,

the mixture was stirred at 100 °C for another 1 hour. The suspended solution was then filtered over Celite and washed with diethyl ether (50 mL). The solvent was removed under reduced pressure, and the crude residue was purified by column chromatography (SiO_2, hexane/MeO*t*Bu, 3:1, R_f = 0.70). Yield: 0.74 g, 2.42 mmol, 61%, white solid, m.p. = 60-61 °C. ^{1}H NMR (500 MHz, $CDCl_3$): δ 3.91 (dd, *J* = 8.5, 2.4 Hz, 1H), 3.69 (dd, *J* = 8.5, 2.6 Hz, 1H), 1.84 – 1.72 (m, 4H), 1.69 – 1.54 (m, 4H), 1.23 (s, 12H), 1.06 (s, 3H), 0.66 – 0.60 (m, 1H), 0.54 – 0.48 (m, 1H), 0.37 – 0.31 (m, 1H), 0.07 (d, *J* = 10.5 Hz, 1H), 0.01 – -0.04 (m, 1H), -0.05 – -0.11 (m, 1H) ppm. ^{13}C{^{1}H} NMR (151 MHz, $CDCl_3$): δ 83.2, 73.9, 68.6, 37.0, 34.1, 33.0, 30.0, 29.8, 26.9, 24.9, 7.7, 7.6, 2.5 ppm. GCMS (M): 306. HRMS (ESI-TOF) *m/z*: $[M + H]^+$ calcd for $C_{18}H_{32}BO_3$, 307.2445; found 307.2457.

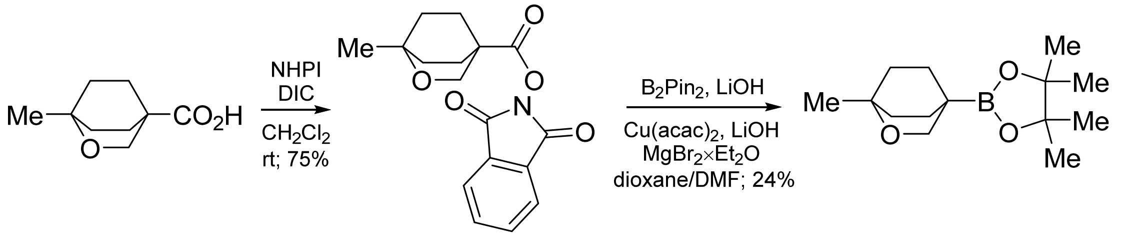

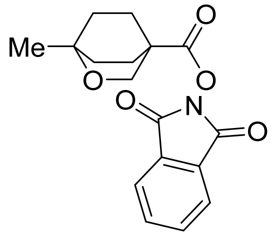

1,3-Dioxoisoindolin-2-yl 1-methyl-2-oxabicyclo[2.2.2]octane-4-carboxylate (78)

The same procedure as for **74** was used. Yield: 2.78 g, 0.0088 mol, 75%, white solid, m.p. = 133-134 °C. ^{1}H NMR (500 MHz, $CDCl_3$): δ 7.91 – 7.84 (m, 2H), 7.82 – 7.74 (m, 2H), 4.16 (s, 2H), 2.29 – 2.19 (m, 2H), 2.19 – 2.09 (m, 2H), 2.00 – 1.87 (m, 2H), 1.78 – 1.65 (m, 2H), 1.15 (s, 3H) ppm. ^{13}C{^{1}H} NMR (151 MHz, $CDCl_3$): δ 171.3, 161.9, 134.9, 129.1, 124.1, 69.2, 69.1, 39.0, 31.9, 27.8, 26.3 ppm. HRMS (ESI-TOF) *m/z*: $[M + H]^+$ calcd for $C_{17}H_{18}NO_5$, 316.1185; found 316.1175.

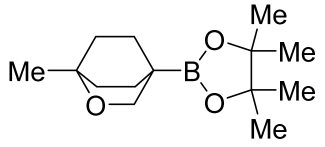

4,4,5,5-Tetramethyl-2-(1-methyl-2-oxabicyclo[2.2.2]octan-4-yl)-1,3,2-dioxaborolane (79)

To a round-bottom flask (150 mL) equipped with a stir bar were added redox-active ester (2.00 g 6.34 mmol, 1.00 equiv), B_2pin_2 (4.82 g, 19.00 mmol, 3.00 equiv), LiOH•H_2O (4.00 g, 95.2 mmol, 15.00 equiv), $Cu(acac)_2$, (0.50 g, 1.9 mmol, 0.30 equiv) and $MgCl_2$ (0.91 g, 9.52 mmol, 1.50 equiv). The tube was evacuated and backfilled with argon for 3 times. Degassed dioxane/DMF (4:1 ratio, 0.14 M, ~ 45 mL) was added and the resulting mixture was stirred under 1500 rpm at room

temperature until dark brown color was observed (approx. 20 min). The reaction mixture was diluted with Et_2O (100 mL) and saturated NH_4Cl (60 mL), and the resulting mixture was shaken vigorously until getting a clear biphasic solution. The organic phase was collected and dried over anhydrous Na_2SO_4, concentrated under reduced pressure and purified by column chromatography (SiO_2, hexane/MeO*t*Bu, 9:1). Yield: 0.40 g, 1.59 mmol, 25%, colorless oil. ^{1}H NMR (500 MHz, $CDCl_3$): δ 3.88 (s, 2H), 1.81 – 1.60 (m, 6H), 1.55 – 1.46 (m, 2H), 1.17 (s, 12H), 1.01 (s, 3H) ppm. ^{13}C{^{1}H} NMR (126 MHz, $CDCl_3$): δ 83.2, 70.9, 67.9, 32.7, 27.2, 26.4, 24.7 ppm. HRMS (ESI-TOF) *m/z*: $[M + H]^+$ calcd for $C_{14}H_{26}BO_3$, 253.1975; found 253.1969.

Synthesis of Imatinib analogues

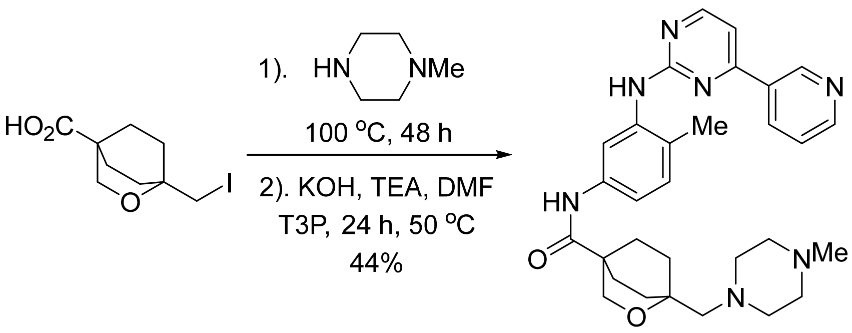

N-(4-methyl-3-((4-(pyridin-3-yl)pyrimidin-2-yl)amino)phenyl)-1-((4-methylpiperazin-1-yl)methyl)-2-oxabicyclo[2.2.2]octane-4-carboxamide (85)

1-(Iodomethyl)-2-oxabicyclo[2.2.2]octane-4-carboxylic acid (0.296 g, 1.00 mmol, 1.00 equiv) was mixed with methylpiperazine (0.50 g, 5.00 mmol, 5.00 equiv). The resulting mixture was heated at 100 °C for 48 h. The reaction mixture was diluted with water (5 mL) and KOH (0.056 g, 1.00 mmol, 1.00 equiv) was added, and water and excess of methylpiperazine were evaporated in *vacuo* (1 torr) in a water boiling bath. The resulting residue was dissolved in 5 mL of DMF and 6-methyl-N^1-(4-(pyridin-3-yl)pyrimidin-2-yl)benzene-1,3-diamine (0.278 g, 1.00 mmol, 1.00 equiv) and Et_3N (1.01 g, 10.00 mmol, 10.00 equiv) were added. The reaction mixture was stirred for 10 min at room temperature, and then 2,4,6-tripropyl-1,3,5,2,4,6-trioxatriphosphorinane-2,4,6-trioxide (T3P) (1.27 g, 2.00 mmol, 2.00 equiv, 50% solution in EtOAc) was added. The mixture was stirred at 50 °C for 24 h and concentrated. The final product was purified by HPLC (1-5 min, 40-60% H_2O/MeOH/0.1% NH_4OH, flow 30 mL/min). Yield: 0.23 g, 0.44 mmol, 44%, beige solid. ^{1}H NMR (500 MHz, $CDCl_3$): δ 9.17 (s, 1H), 8.66 (d, *J* = 4.7 Hz, 1H), 8.49 – 8.41 (m, 3H), 7.42 (s, 1H), 7.38 (dd, *J* = 7.8, 4.9 Hz, 1H), 7.16 – 7.11 (m, 2H), 7.09 (s, 2H), 4.00 (s, 2H), 3.44 (s, 2H), 3.41 (s, 3H), 2.56 (s, 2H), 2.45 (s, 2H), 2.26 (s, 7H), 2.12 – 2.04 (m, 2H), 2.01 – 1.88 (m, 4H), 1.65 – 1.54 (m, 2H) ppm. $^{13}C\{^1H\}$ NMR (126 MHz, $CDCl_3$): δ 172.8, 162.7, 160.6, 159.1, 151.4, 148.4, 137.8, 136.2, 135.1, 132.8, 130.7, 124.4, 123.8, 115.4, 113.4, 108.3, 72.4, 70.1, 65.4, 55.1, 54.5, 50.4, 45.7, 40.6, 29.2, 27.8, 17.6 ppm. LCMS $(M-H)^-$: 526. HRMS (ESI-TOF) *m/z*: $[M + H]^+$ calcd for $C_{30}H_{38}N_7O_2$, 528.3087; found 528.3084.

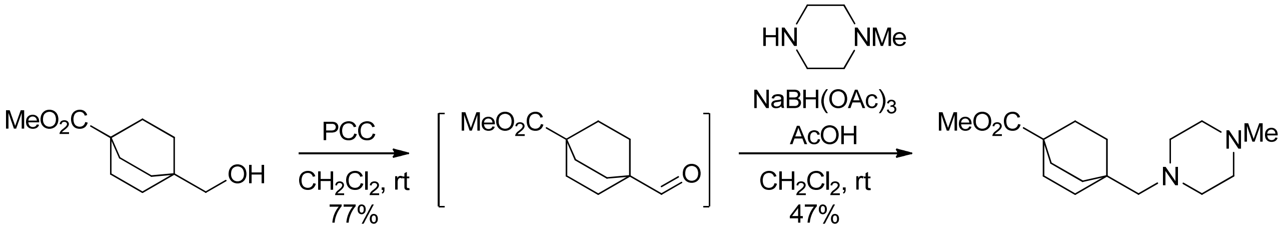

EN300-198570

Methyl 4-formylbicyclo[2.2.2]octane-1-carboxylate (SI-24)

To a mixture of methyl 4-(hydroxymethyl)bicyclo[2.2.2]octane-1-carboxylate (2.50 g, 12.60 mmol, 1.00 equiv) and SiO_2 (ca. 10 g) in CH_2Cl_2 (35 mL) was added pyridinium chlorochromate (PCC; 5.43 g, 25.20 mmol, 2.00 equiv) at room temperature. The mixture was stirred for 4 h at room temperature. The resulting mixture was passed through a layer of silica gel (ca. 20 g) and washed with a mixture of CH_2Cl_2:MeO*t*Bu = 1:1 (ca. 100 mL). The combined organic solvents were evaporated to give the product (ca. 90% purity) that was immediately used in the next step. Yield: 1.90 g, 9.69 mmol, 77%, white solid. ^{1}H NMR (400 MHz, $CDCl_3$): δ 9.47 (s, 1H), 3.67 (s, 3H), 1.88 – 1.83 (m, 6H), 1.72 – 1.66 (m, 6H) ppm.

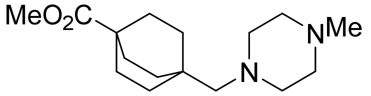

Methyl 4-((4-methylpiperazin-1-yl)methyl)bicyclo[2.2.2]octane-1-carboxylate (SI-25)

To a mixture of methyl 4-formylbicyclo[2.2.2]octane-1-carboxylate (2.50 g, 12.75 mmol, 1.00 equiv) and 1-methylpiperazine (1.40 g, 14.00 mmol, 1.10 equiv) and CH_2Cl_2 (35 mL) was added AcOH (0.10 g, 1.67 mmol, 0.13 equiv) at room temperature. The mixture was stirred for 5 min and sodium triacetoxyborohydride (4.33 g, 20.42 mmol, 1.60 equiv) was added in portions (~0.3 g) at room temperature. The resulting mixture was stirred overnight and a 10% solution of K_2CO_3 (30 mL) was added. The mixture was partially concentrated. The aqueous layer was extracted with CH_2Cl_2 (3 × 30 mL). The combined organic layers were concentrated under reduced pressure. The final product was purified by HPLC (1-5 min, 40-90% H_2O/MeOH/0.1% NH_4OH, flow 30 mL/min). Yield: 1.70 g, 6.07 mmol, 47%, white solid, m.p. = 87-88 °C. ^{1}H NMR (500 MHz, $CDCl_3$): δ 3.62 (s, 3H), 2.48 (br s, 4H), 2.38 (br s, 4H), 2.25 (s, 3H), 2.01 (s, 2H), 1.74 (dd, J = 9.7, 6.3 Hz, 6H), 1.41 (dd, J = 9.7, 6.4 Hz, 6H) ppm. $^{13}C\{^1H\}$ NMR (126 MHz, $CDCl_3$): δ 178.8, 68.1, 55.8, 55.7, 51.7, 46.2, 39.4, 33.5, 29.2, 28.6 ppm. LCMS $(M+H)^+$: 281. HRMS (ESI-TOF) *m/z*: $[M + H]^+$ calcd for $C_{16}H_{29}N_2O_2$, 281.2229; found 281.2224.

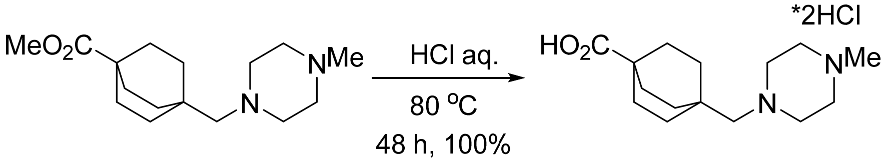

4-((4-Methylpiperazin-1-yl)methyl)bicyclo[2.2.2]octane-1-carboxylic acid dihydrochloride (SI-26)

Methyl 4-((4-methylpiperazin-1-yl)methyl)bicyclo[2.2.2]octane-1-carboxylate (1.00 g, 3.60 mmol) was dissolved in 20 mL of 5% aq. HCl. The flask was fitted with a reflux condenser and the mixture was heated at reflux for 48 h and then concentrated. The residue was dried at 80 °C at 0.1 torr to give the desired product as a beige solid, m.p. = 183-185 °C. Yield: 1.22 g, 3.60 mmol, 100%. ^{1}H

NMR (500 MHz, D_2O): δ 3.71 (br s, 8H), 3.21 (s, 2H), 3.03 (s, 3H), 1.83 (dd, J = 9.5, 6.0 Hz, 6H), 1.64 (dd, J = 9.3, 6.1 Hz, 6H) ppm. $^{13}C\{^1H\}$ NMR (151 MHz, D_2O): δ 182.3, 67.1, 51.2, 49.4, 42.8, 38.1, 31.3, 27.7, 27.0 ppm. LCMS $(M+H)^+$: 267. HRMS (ESI-TOF) *m/z*: $[M + H]^+$ calcd for $C_{15}H_{27}N_2O_2$, 267.2073; found 267.2068.

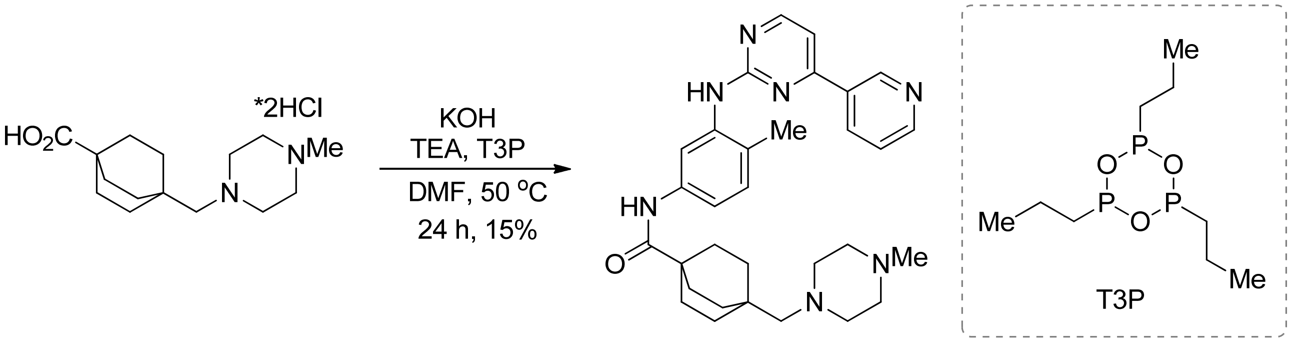

N-(4-methyl-3-((4-(pyridin-3-yl)pyrimidin-2-yl)amino)phenyl)-4-((4-methylpiperazin-1-yl)methyl)bicyclo[2.2.2]octane-1-carboxamide (86)

4-((4-Methylpiperazin-1-yl)methyl)bicyclo[2.2.2]octane-1-carboxylic acid dihydrochloride (0.34 g, 1.00 mmol, 1.00 equiv) was dissolved in 5 mL of DMF and 6-methyl-N^1-(4-(pyridin-3-yl)pyrimidin-2-yl)benzene-1,3-diamine (0.278 g, 1.00 mmol, 1.00 equiv) and Et_3N (1.01 g, 10.00 mmol, 10.00 equiv) were added. The reaction mixture was stirred for 10 min at room temperature, and then 2,4,6-tripropyl-1,3,5,2,4,6-trioxatriphosphorinane-2,4,6-trioxide (T3P) (1.27 g, 2.00 mmol, 2.00 equiv, 50% solution in EtOAc) was added. The solution was stirred at 50 °C for 24 h and concentrated. The final product was purified by HPLC (1-5 min, 30-65% H_2O/CH_3CN/0.1% NH_4OH, flow 30 mL/min). Yield: 80 mg, 0.15 mmol, 15%, beige solid. ^1H NMR (500 MHz, DMSO-d_6): δ 9.25 (s, 1H), 9.02 (s, 1H), 8.90 (s, 1H), 8.74 – 8.66 (m, 1H), 8.50 (dd, J = 5.0, 2.5 Hz, 1H), 8.46 (dd, J = 7.9, 1.8 Hz, 1H), 7.96 (s, 1H), 7.54 – 7.48 (m, 1H), 7.42 (dd, J = 5.0, 2.4 Hz, 1H), 7.32 (d, J = 8.2 Hz, 1H), 7.11 (d, J = 8.3 Hz, 1H), 2.41 (s, 4H), 2.29 (s, 4H), 2.18 (d, J = 1.4 Hz, 3H), 2.15 – 2.07 (m, 3H), 2.00 (s, 2H), 1.82 – 1.72 (m, 5H), 1.44 – 1.36 (m, 5H) ppm. $^{13}C\{^1H\}$ NMR (101 MHz, DMSO-d_6): δ 175.9, 161.5, 161.2, 159.5, 151.4, 148.2, 137.4, 134.4, 132.2, 129.8, 126.8, 123.8, 116.9, 107.2, 67.4, 55.3, 55.1, 45.7, 33.1, 28.9, 28.0, 17.6 ppm. LCMS $(M-H)^-$: 524. HRMS (ESI-TOF) *m/z*: $[M + H]^+$ calcd for $C_{31}H_{40}N_7O$, 526.3294; found 526.3288.

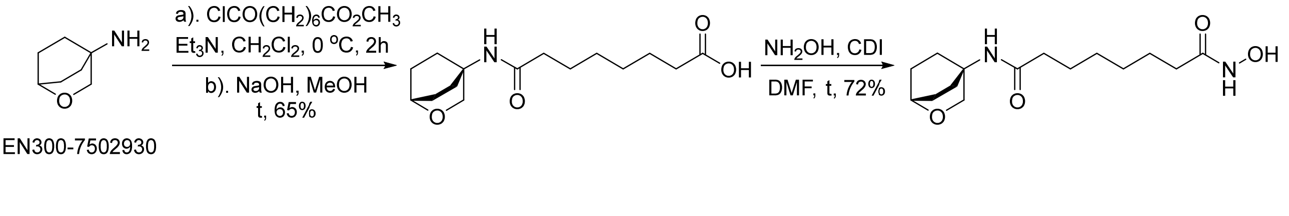

EN300-7502930

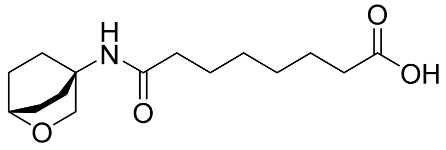

8-((2-Oxabicyclo[2.2.2]octan-4-yl)amino)-8-oxooctanoic acid (SI-27)

Methyl 8-chloro-8-oxooctanoate (0.50 g, 2.40 mmol, 1.00 equiv) was added to a solution of commercially available 2-oxabicyclo[2.2.2]octan-4-amine (0.305 g, 2.40 mmol, 1.00 equiv) and Et_3N (0.48 g, 2.40 mmol, 1.00 equiv) in 30 mL of CH_2Cl_2. The resulting mixture was stirred at 0° C for 2 h. The mixture was washed with water (2 × 10 mL), dried over Na_2SO_4, filtered and evaporated to give the crude ester as a brown oil, which was saponificated with a mixture of NaOH (0.12 g, 3.00 mmol, 1.25 equiv) and boiling MeOH (10 mL) for 30 min. The solvent was evaporated and 1M HCl was added to pH = 2-3 at 0 °C. The desired acid was filtered and dried. Yield: 0.44 g, 1.55 mmol, 65%, white solid. ^1H NMR (500 MHz, DMSO-d_6): δ 11.96 (br s, 1H), 7.33 (s, 1H), 3.79 (s, 2H), 3.62 (s, 1H), 2.17 (t, J = 7.4 Hz, 2H), 1.98 (t, J = 7.4 Hz, 2H), 1.97 – 1.82 (m, 4H), 1.76 (t, J = 11.7 Hz, 2H), 1.62 (t, J = 11.7 Hz, 2H), 1.52 – 1.35 (m, 4H), 1.31 – 1.13 (m, 4H) ppm. $^{13}C\{^1H\}$ NMR (126 MHz, DMSO-d_6): δ 174.5, 172.2, 69.4, 64.0, 48.6, 35.8, 33.6, 28.4, 28.3, 26.2, 25.2, 24.4 ppm. LCMS $(M+H)^+$: 284. HRMS (ESI-TOF) *m/z*: $[M + H]^+$ calcd for $C_{15}H_{26}NO_4$, 284.1862; found 284.1853.

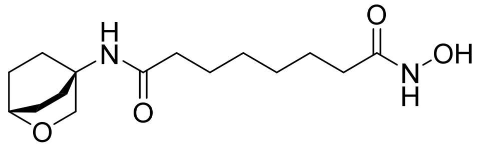

***N*[1]-(2-oxabicyclo[2.2.2]octan-4-yl)-*N*[8]-hydroxyoctanediamide (88)**

8-((2-Oxabicyclo[2.2.2]octan-4-yl)amino)-8-oxooctanoic acid (0.283 g, 1.00 mmol, 1.00 equiv) was dissolved in DMF (5 mL) and CDI (0.32 g, 2.0 mmol, 2.00 equiv) was added at 25-30 °C. The mixture was stirred for 30 min at this temperature, and hydroxylamine hydrochloride (0.28 g, 4.00 mmol, 4.00 equiv) was added. The mixture was stirred for 30 min at this temperature and then water (50 mL) was added. The mixture was stirred for 2 h at room temperature. The solution was extracted with EtOAc (3 × 30 mL). The combined organic layers were concentrated under reduced pressure. The final product was purified by HPLC: Rt = 0-5 min, 0-30%, H_2O/CH_3CN, flow 30 mL/min (loading pump 4 mL/min). Yield: 0.214 g, 0.72 mmol, 72%, beige solid. ^1H NMR (500 MHz, DMSO-d_6): δ 10.31 (s, 1H), 8.64 (s, 1H), 7.34 (s, 1H), 3.79 (s, 2H), 3.62 (s, 1H), 2.10 – 1.80 (m, 8H), 1.76 (t, J = 11.8 Hz, 2H), 1.62 (t, J = 11.7 Hz, 2H), 1.51 – 1.34 (m, 4H), 1.26 – 1.12 (m, 4H) ppm. $^{13}C\{^1H\}$ NMR (151 MHz, DMSO-d_6): δ 172.2, 169.1, 69.4, 64.0, 48.6, 35.8, 32.2, 28.4, 28.4, 28.3, 26.2, 25.2, 25.0 ppm. LCMS $(M+H)^+$: 299. HRMS (ESI-TOF) *m/z*: $[M + Na]^+$ calcd for $C_{15}H_{26}N_2NaO_4$, 321.1790; found 321.1784.

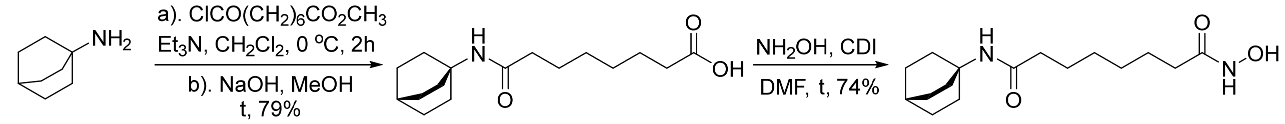

EN300-234104

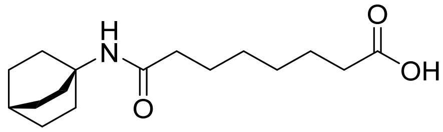

8-(Bicyclo[2.2.2]octan-1-ylamino)-8-oxooctanoic acid (SI-28)

Methyl 8-chloro-8-oxooctanoate (0.50 g, 2.40 mmol, 1.00 equiv) was added to a solution of commercially available bicyclo[2.2.2]octan-1-amine (0.30 g, 2.40 mmol, 1.00 equiv) and Et_3N (0.48 g, 2.40 mmol, 1.00 equiv) in 30 mL of CH_2Cl_2. The resulting mixture was stirred at 0° C for 2 h. The mixture was washed with water (2 × 10 mL), dried over Na_2SO_4, filtered and evaporated to give the crude ester as a brown oil, which was saponificated with a mixture of NaOH (0.12 g, 3.00 mmol, 1.25 equiv) and boiling MeOH (10 mL) for 30 min. The solvent was evaporated and 1M HCl was added to pH = 2-3 at 0 °C. The desired acid was filtered and dried. Yield: 0.53 g, 1.89 mmol, 79%, white solid, m.p. = 129-130 °C. ^{1}H NMR (500 MHz, DMSO-d$_6$): δ 11.96 (s, 1H), 7.12 (s, 1H), 2.17 (t, J = 7.4 Hz, 2H), 1.96 (t, J = 7.4 Hz, 2H), 1.88 – 1.60 (m, 6H), 1.60 – 1.29 (m, 11H), 1.29 – 1.14 (m, 4H) ppm. ^{13}C{^{1}H} NMR (151 MHz, DMSO-d$_6$): δ 174.4, 171.7, 49.4, 36.0, 33.6, 29.7, 28.29, 28.26, 25.8, 25.3, 24.4, 23.4 ppm. LCMS $(M+H)^+$: 282. HRMS (ESI-TOF) *m/z*: $[M + H]^+$ calcd for $C_{16}H_{28}NO_3$, 282.2069; found 282.2062.

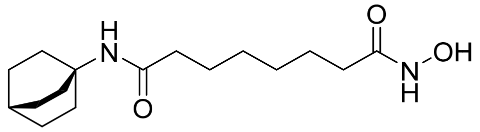

***N*^{1}-(bicyclo[2.2.2]octan-1-yl)-*N*^{8}-hydroxyoctanediamide (89)**

8-(Bicyclo[2.2.2]octan-1-ylamino)-8-oxooctanoic acid (0.28 g, 1.00 mmol, 1.00 equiv) was dissolved in DMF (5 mL) and CDI (0.32 g, 2.00 mmol, 2.00 equiv) was added at 25-30 °C. The mixture was stirred for 30 min at this temperature, and hydroxylamine hydrochloride (0.28 g, 4.00 mmol, 4.00 equiv) was added. The mixture was stirred for 30 min at this temperature and then water (50 mL) was added. The mixture was stirred for 2 h at room temperature. The solution was extracted with EtOAc (3 × 30 mL). The combined organic layers were concentrated under reduced pressure. The final product was purified by HPLC: Rt = 0-6 min, 15-40%, H_2O/CH_3CN, flow 30 mL/min (loading pump 4 mL/min). Yield: 0.22 g, 0.74 mmol, 74%, beige solid. ^{1}H NMR (500 MHz, DMSO-d$_6$): δ 10.27 (br s, 1H), 8.60 (br s, 1H), 7.12 (s, 1H), 1.95 (t, J = 7.4 Hz, 2H), 1.91 (t, J

= 7.4 Hz, 2H), 1.79 – 1.68 (m, 6H), 1.59 – 1.52 (m, 6H), 1.49 – 1.39 (m, 5H), 1.24 – 1.14 (m, 4H) ppm. $^{13}C\{^{1}H\}$ NMR (151 MHz, DMSO-d_6): δ 171.7, 169.1, 49.4, 36.0, 32.3, 29.7, 28.4, 28.3, 25.8, 25.3, 25.1, 23.4 ppm. LCMS $(M+H)^+$: 297. HRMS (ESI-TOF) *m/z*: $[M + H]^+$ calcd for $C_{16}H_{29}N_2O_3$, 297.2178; found 297.2174.

Copies of ^{1}H, $^{13}C\{^{1}H\}$ and $^{19}F\{^{1}H\}$ NMR spectra

Compound 8

^{1}H NMR (500 MHz, $CDCl_3$)

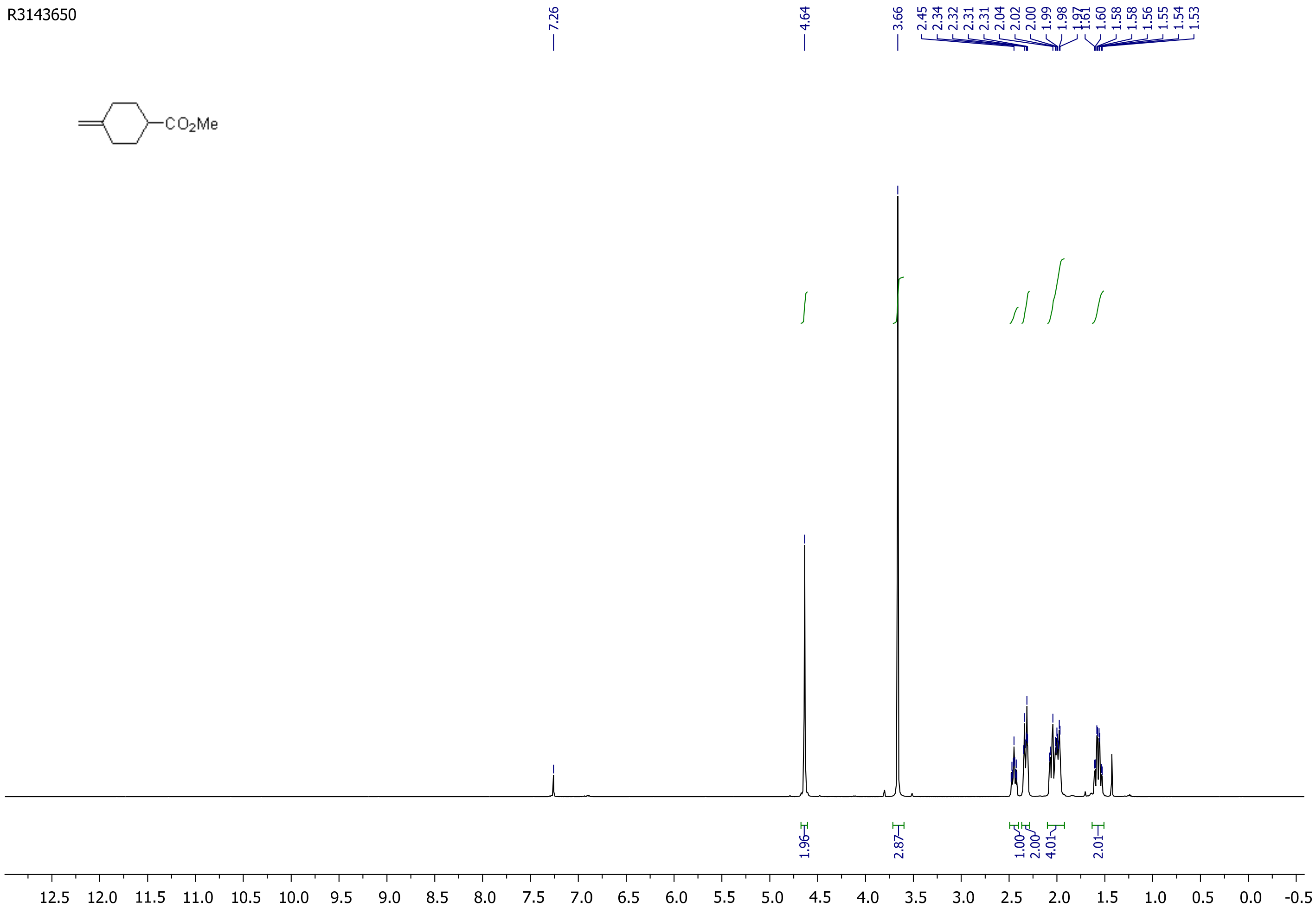

$^{13}C\{^1H\}$ NMR (126 MHz, $CDCl_3$)

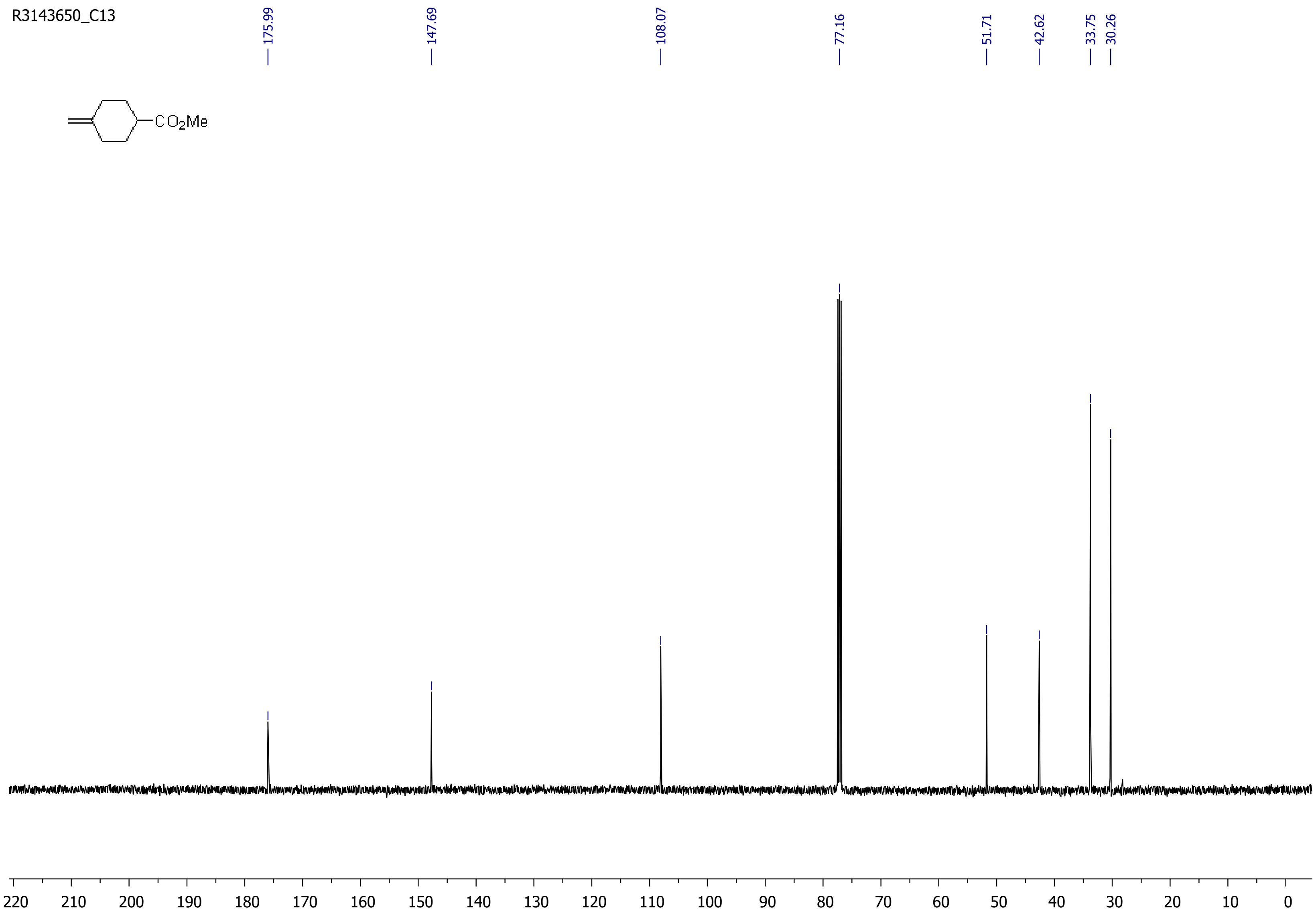

Compound SI-1

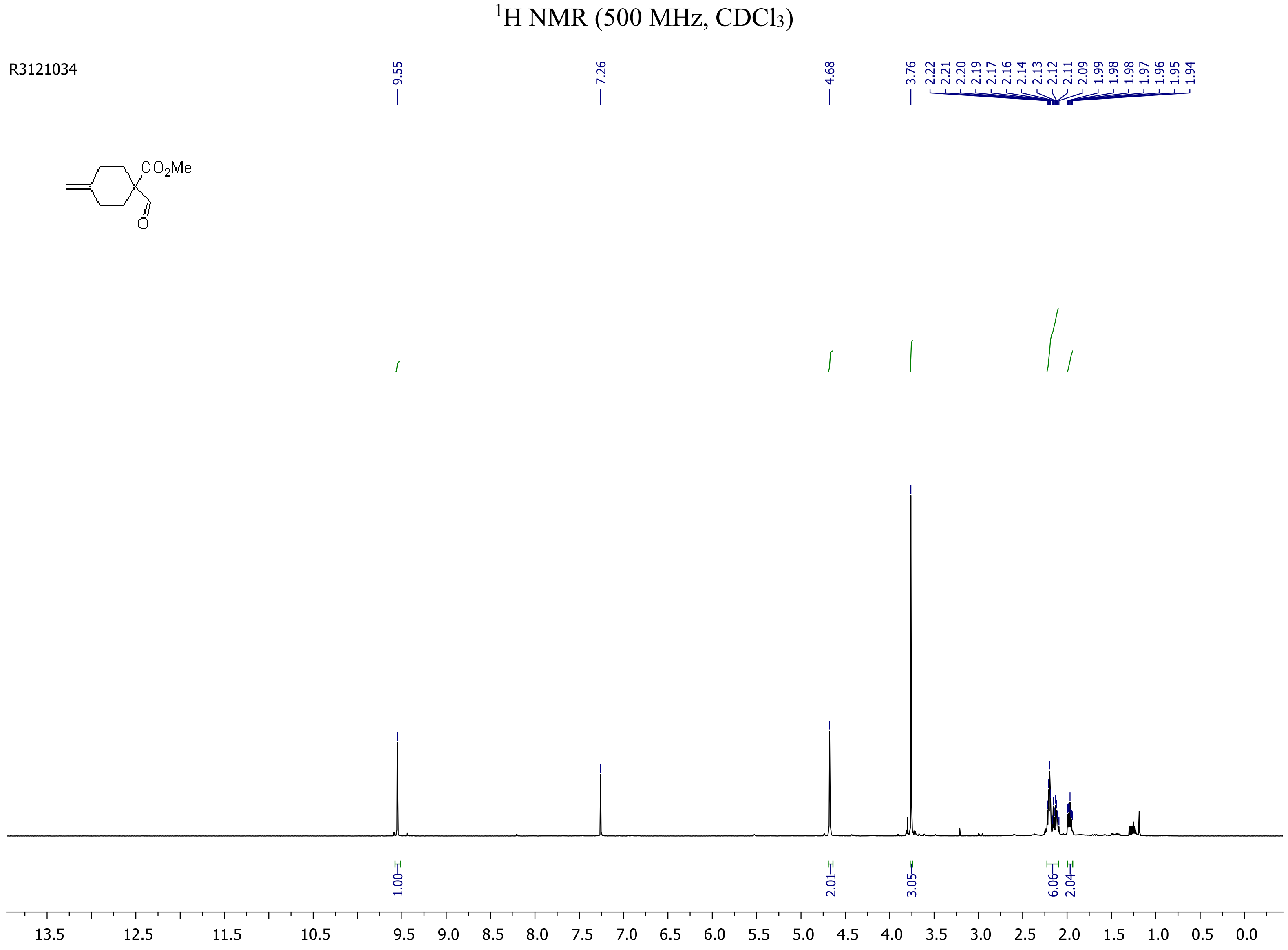

$^{13}C\{^{1}H\}$ NMR (126 MHz, $CDCl_3$)

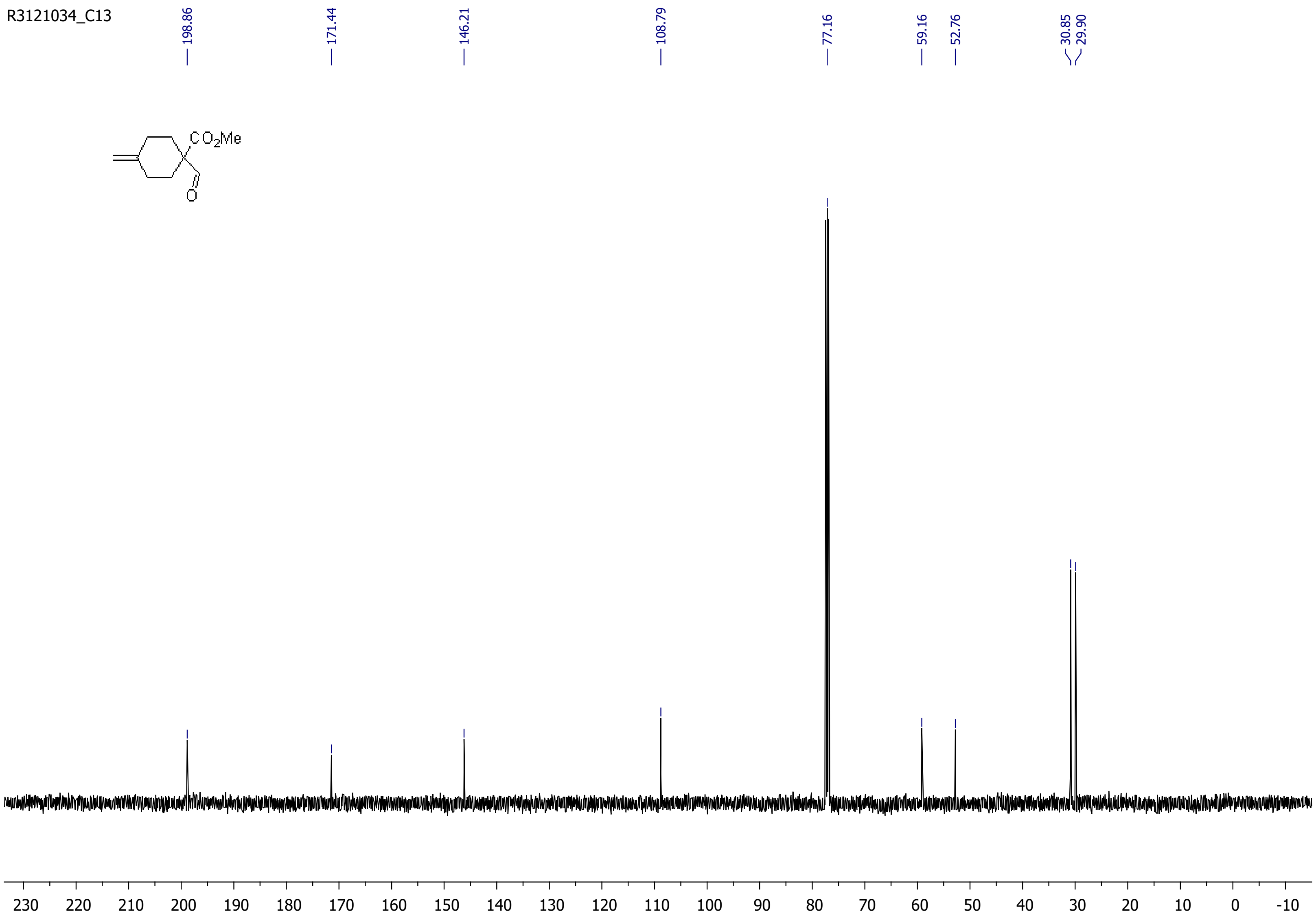

Compound 5

^{1}H NMR (500 MHz, $CDCl_3$)

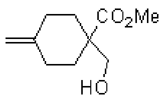

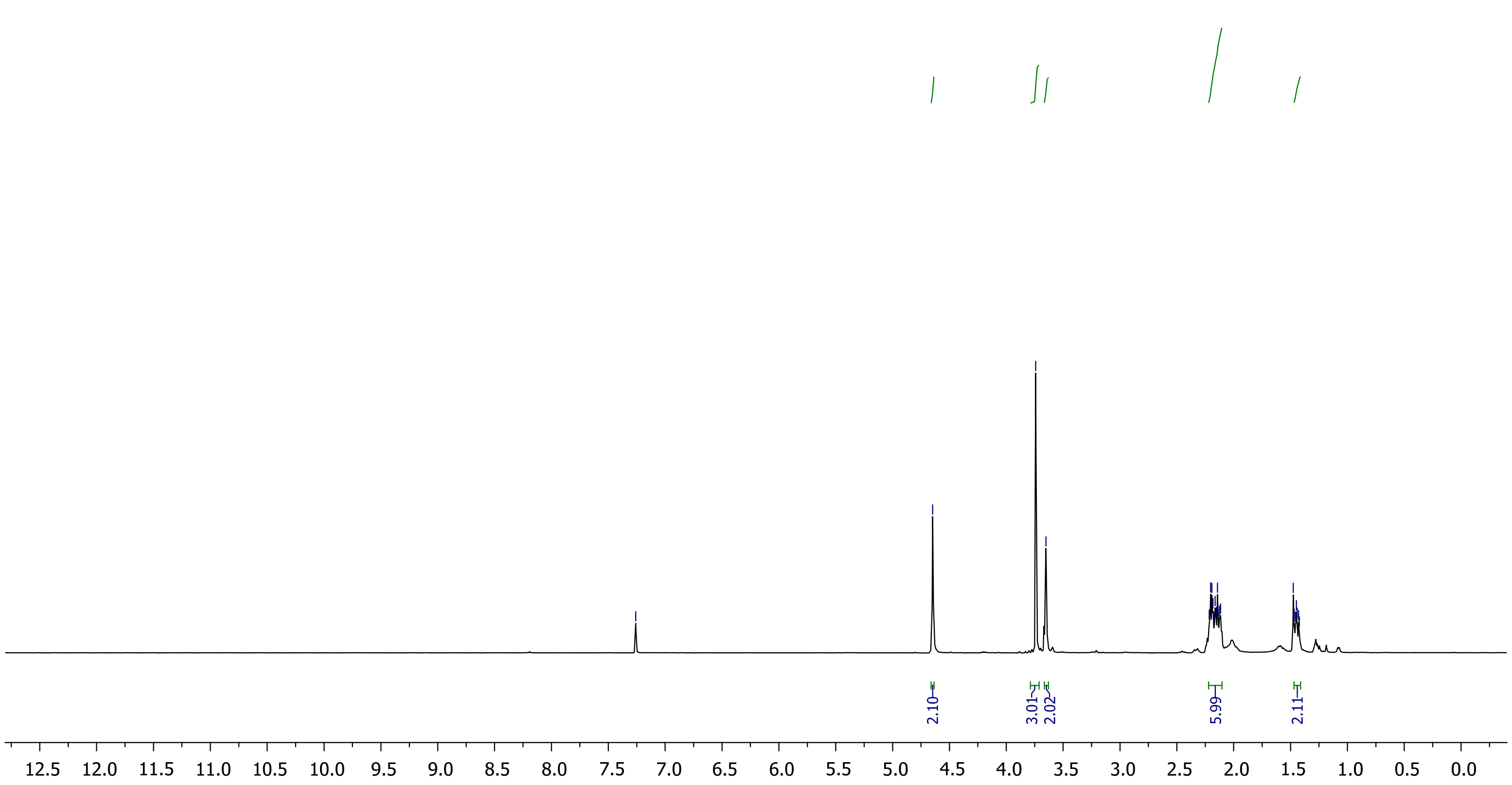

$^{13}C\{^{1}H\}$ NMR (151 MHz, $CDCl_3$)

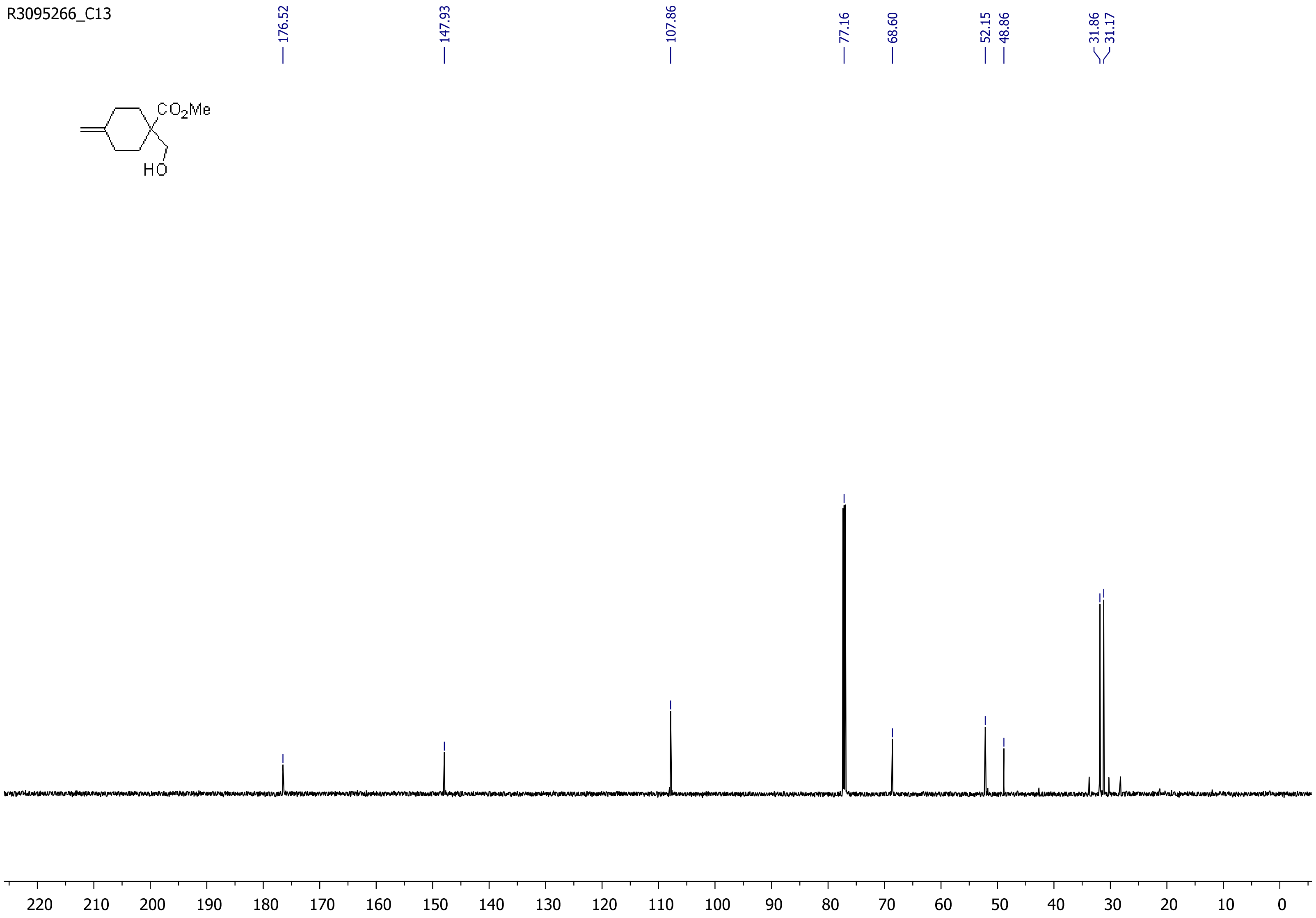

Compound 6

^{1}H NMR (400 MHz, $CDCl_3$)

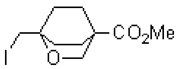

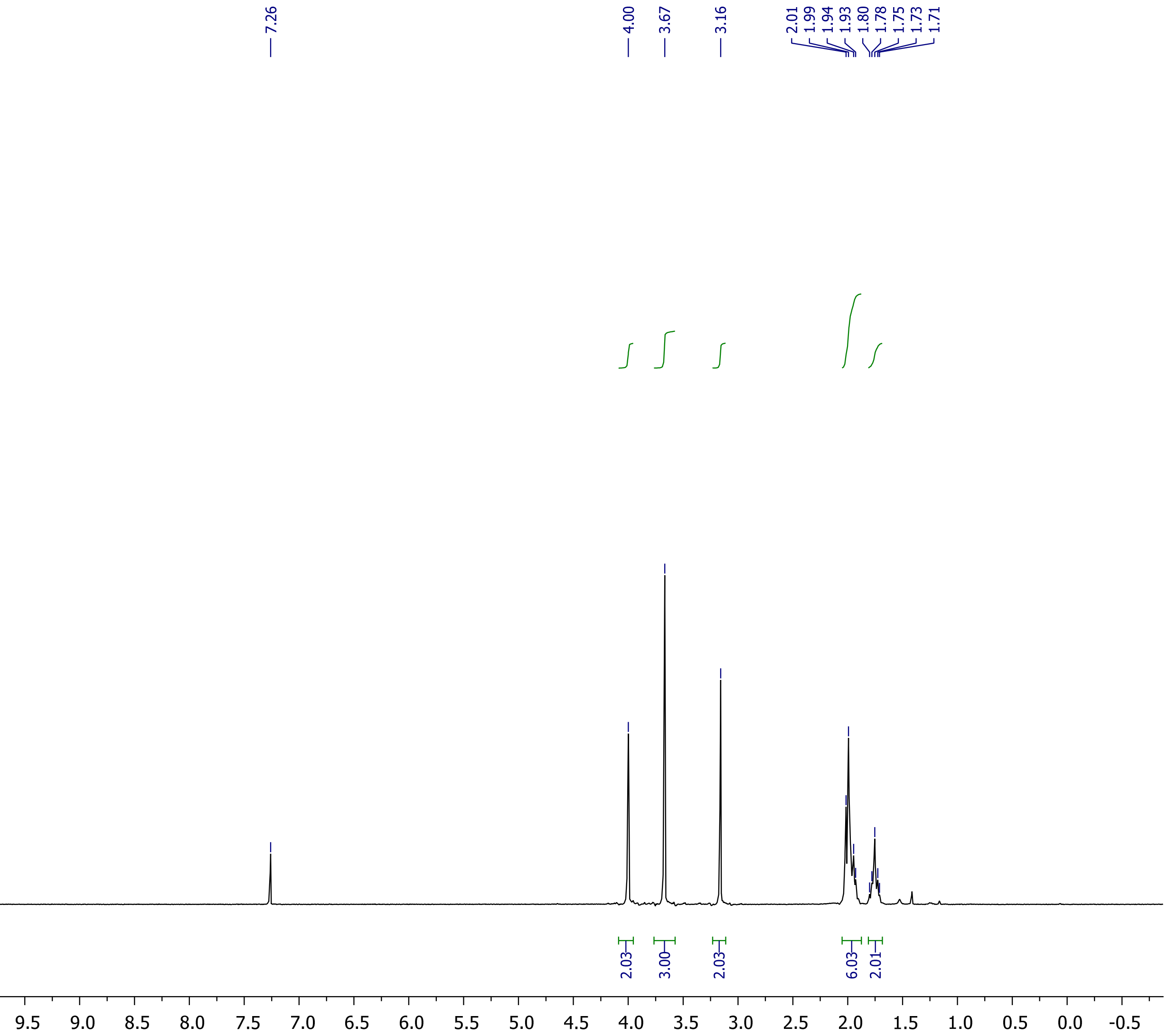

$^{13}C\{^1H\}$ NMR (126 MHz, $CDCl_3$)

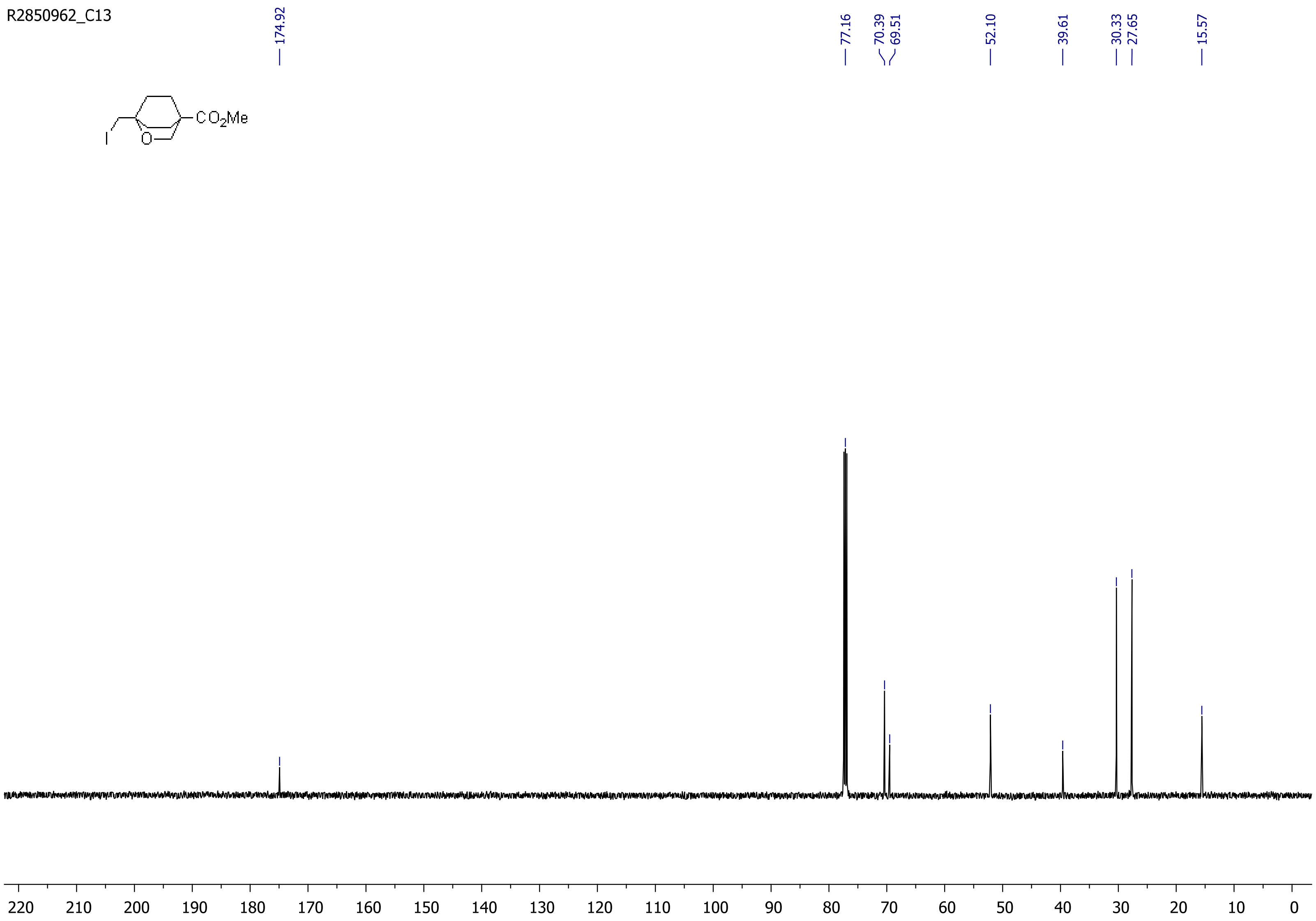

Compound SI-2

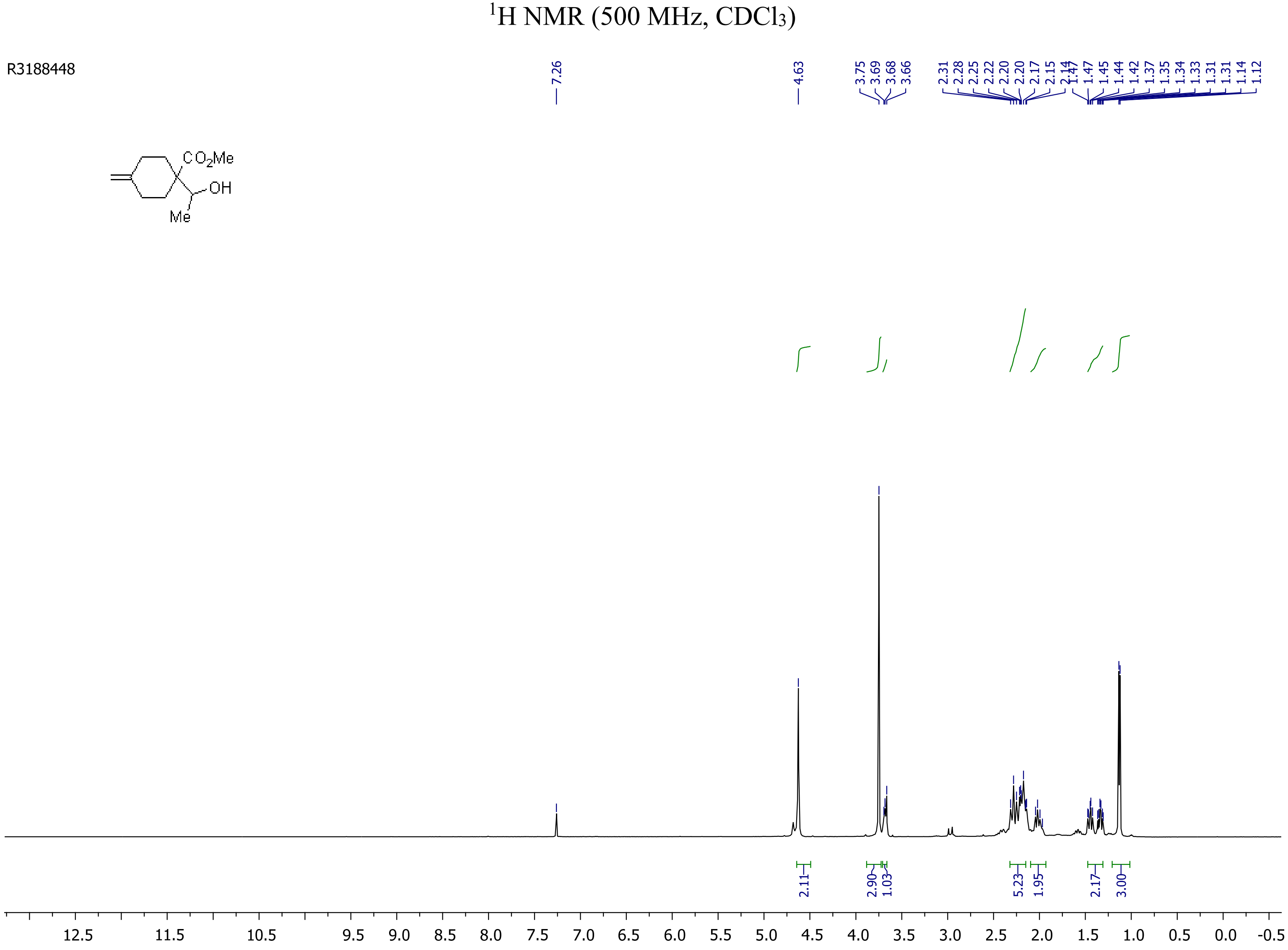

$^{13}C\{^1H\}$ NMR (151 MHz, $CDCl_3$)

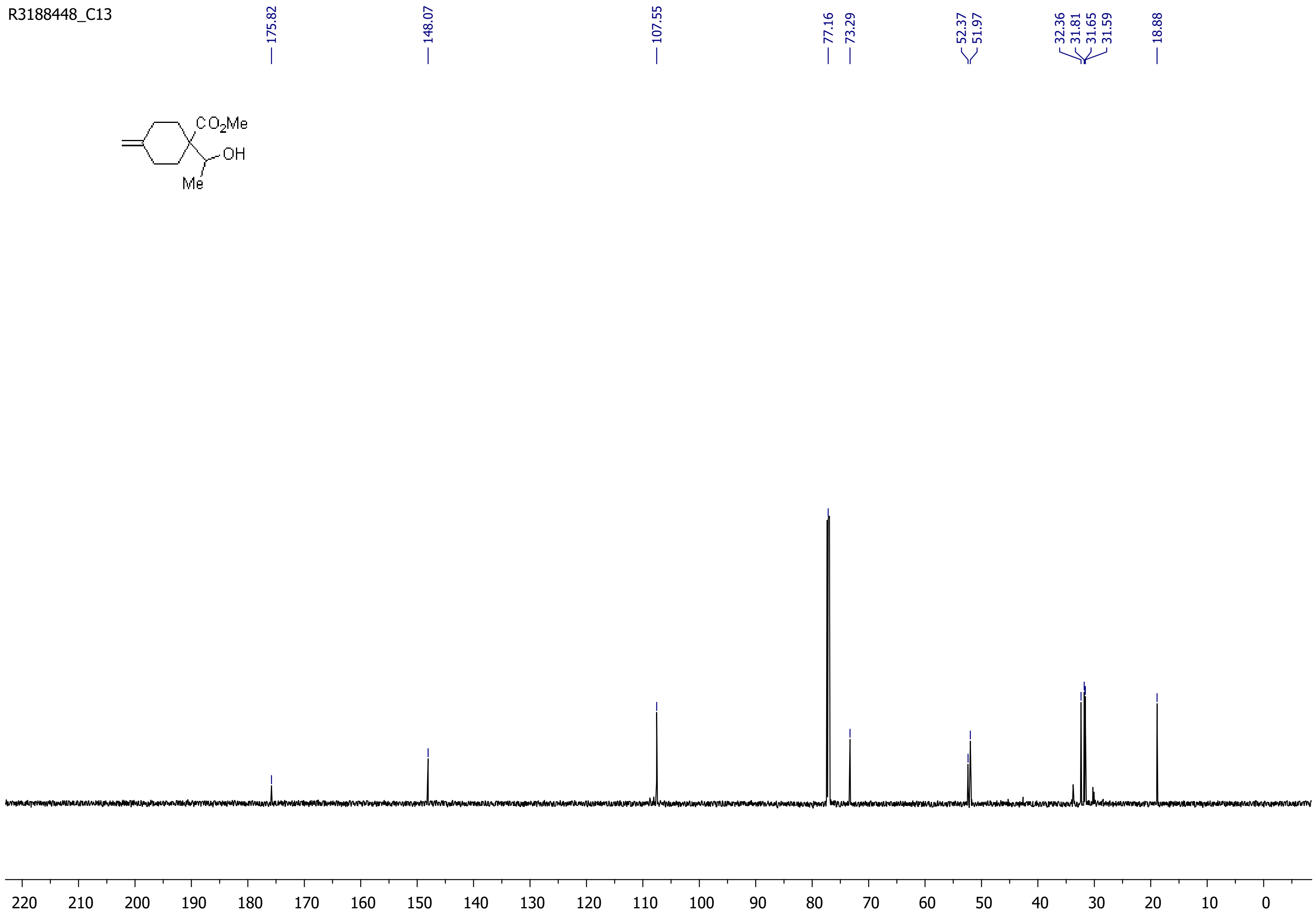

R3188448_C13
175.82
148.07
107.55
77.16
73.29
52.37
51.97
32.36
31.81
31.65
31.59
18.88
CO2Me
OH
Me
220 210 200 190 180 170 160 150 140 130 120 110 100 90 80 70 60 50 40 30 20 10 0

Compound 9

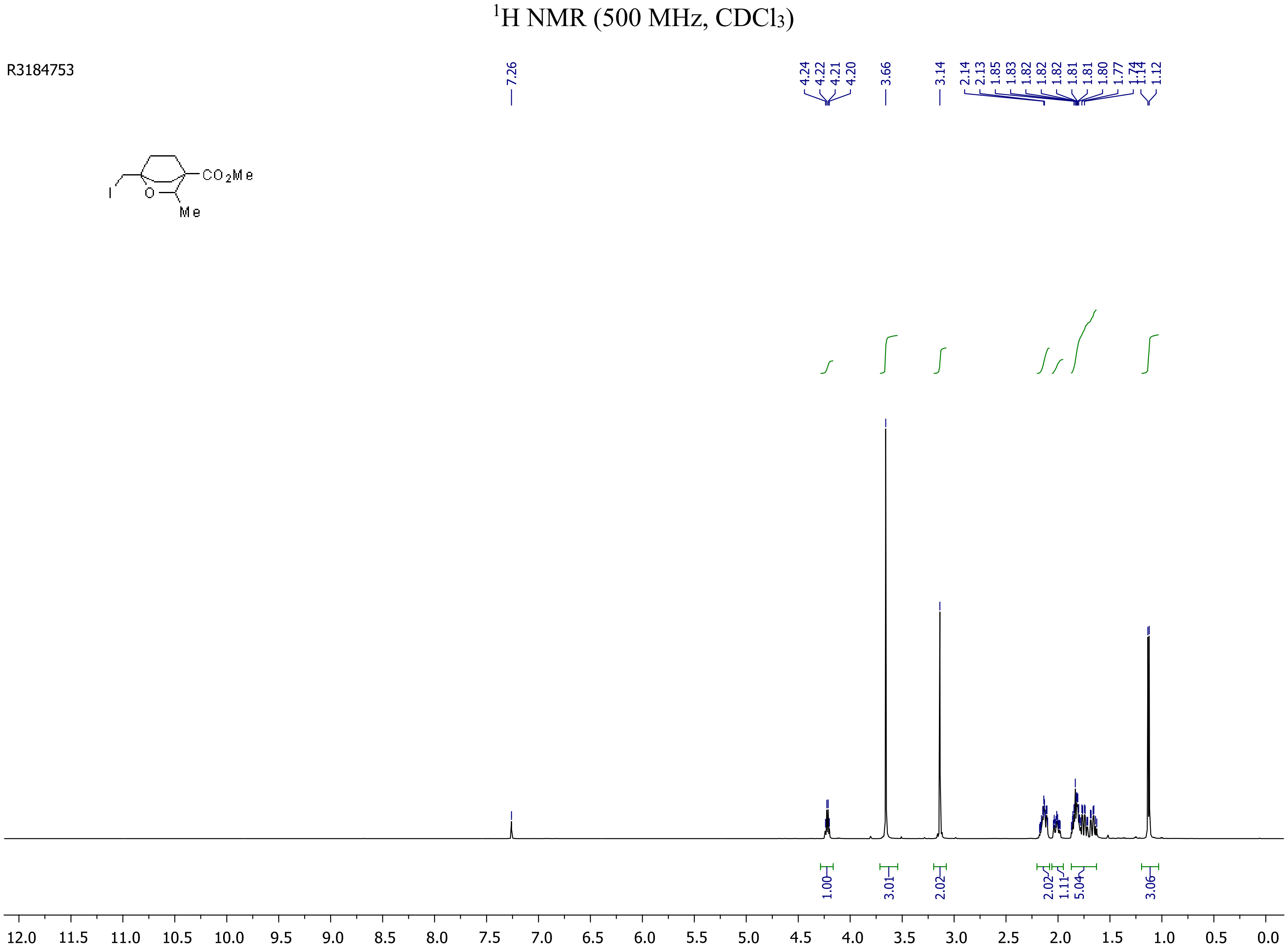

1H NMR (500 MHz, CDCl3)
R3184753
CO2Me
I
O
Me
7.26
4.24
4.22
4.21
4.20
3.66
3.14
2.14
2.13
1.85
1.83
1.82
1.82
1.82
1.81
1.81
1.80
1.77
1.14
1.12
1.00
3.01
2.02
2.02
1.11
5.04
3.06
12.0
11.5
11.0
10.5
10.0
9.5
9.0
8.5
8.0
7.5
7.0
6.5
6.0
5.5
5.0
4.5
4.0
3.5
3.0
2.5
2.0
1.5
1.0
0.5
0.0

$^{13}C\{^{1}H\}$ NMR (151 MHz, $CDCl_3$)

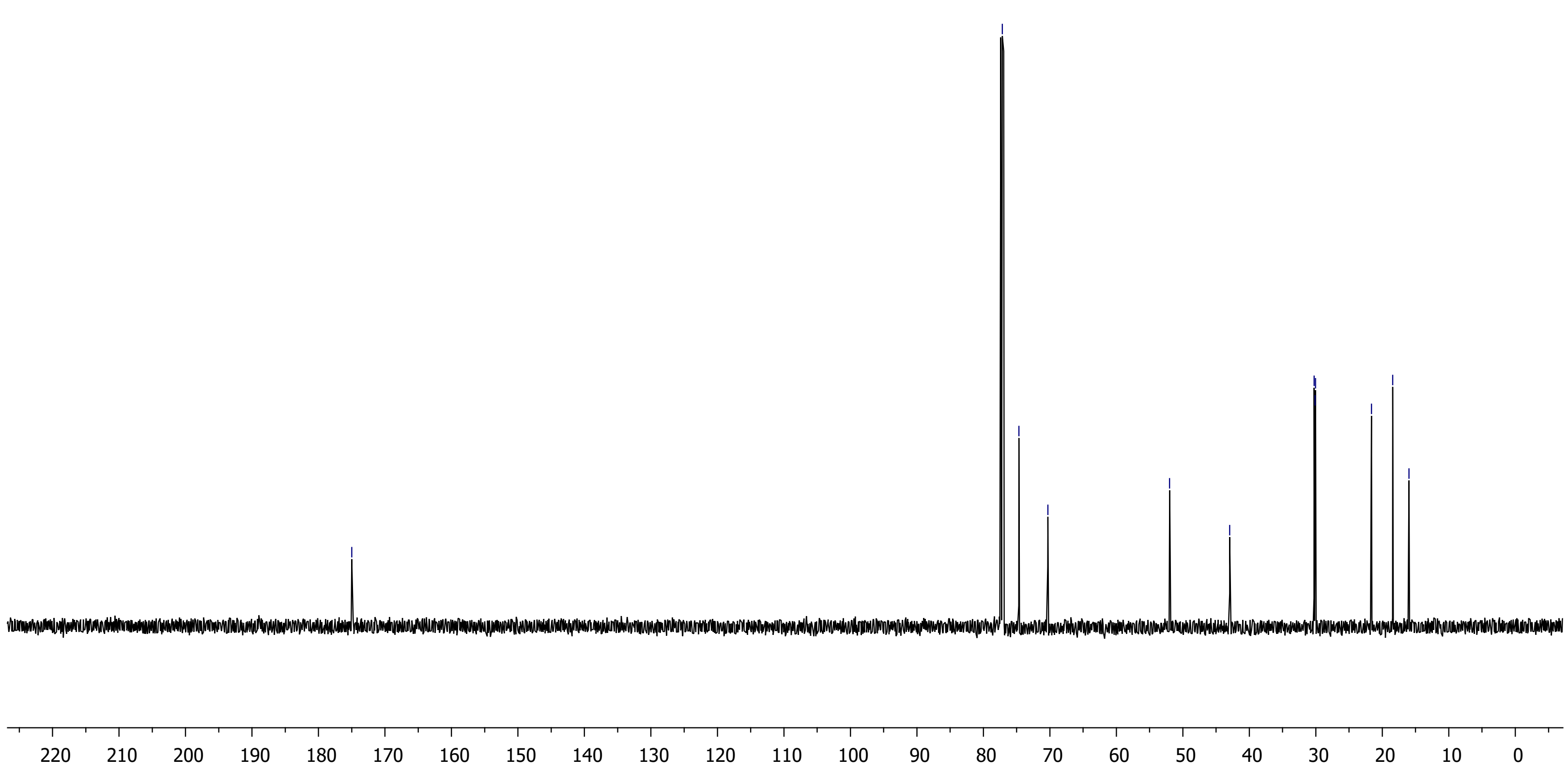

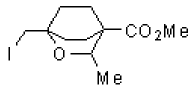

Compound SI-3

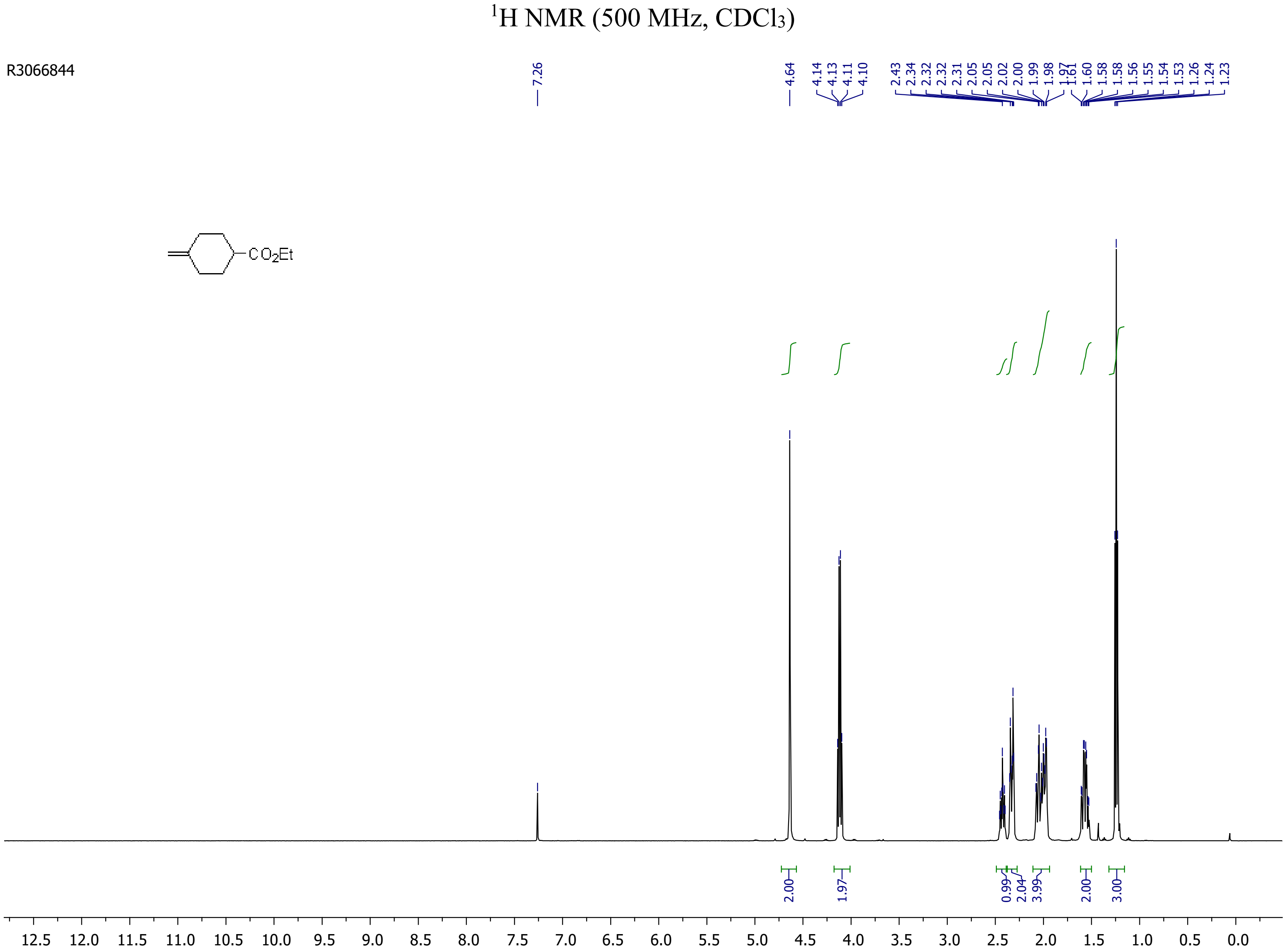

$^{13}C\{^{1}H\}$ NMR (126 MHz, $CDCl_3$)

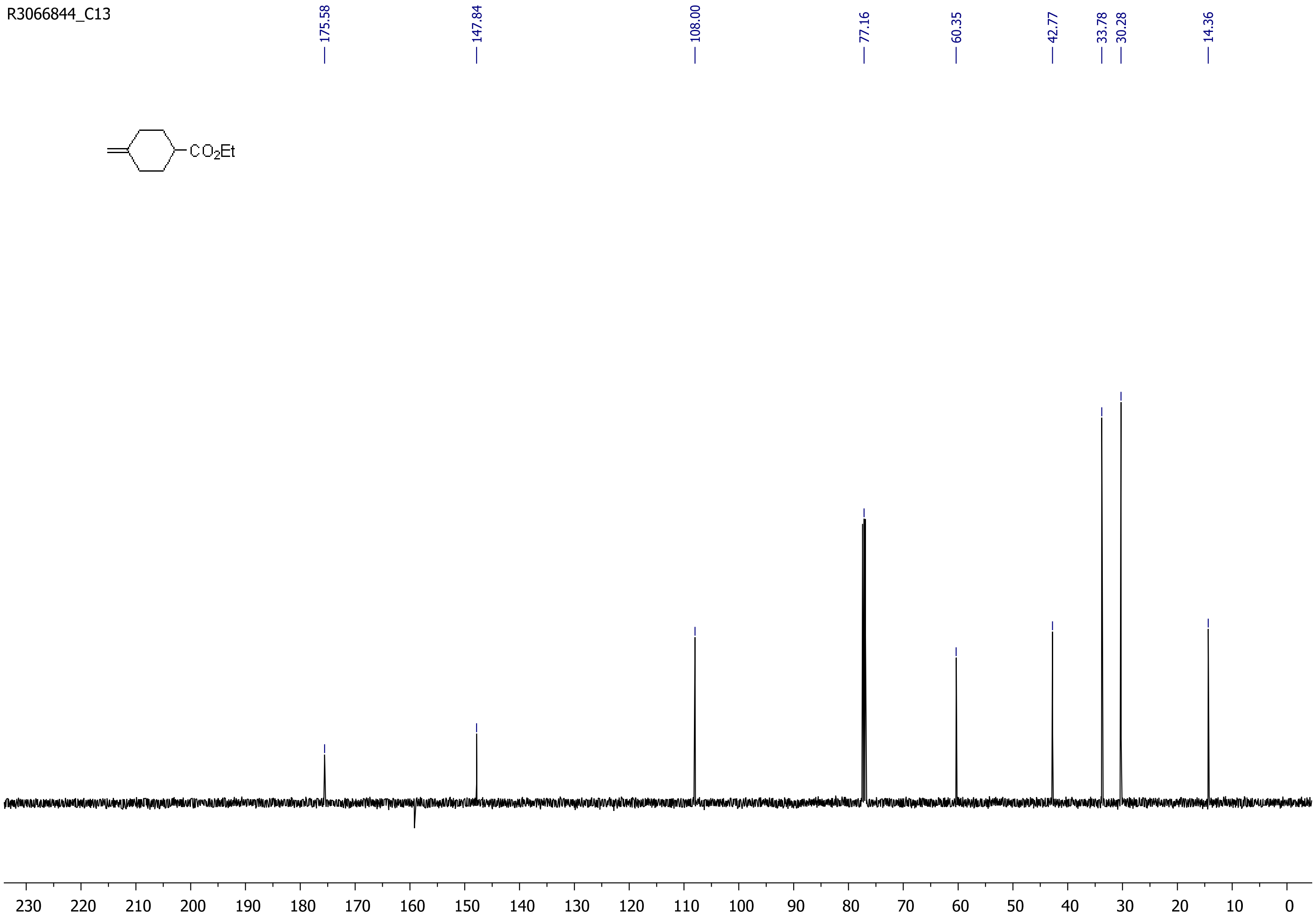

Compound 10

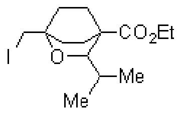
CO2Et
I
O
Me
Me

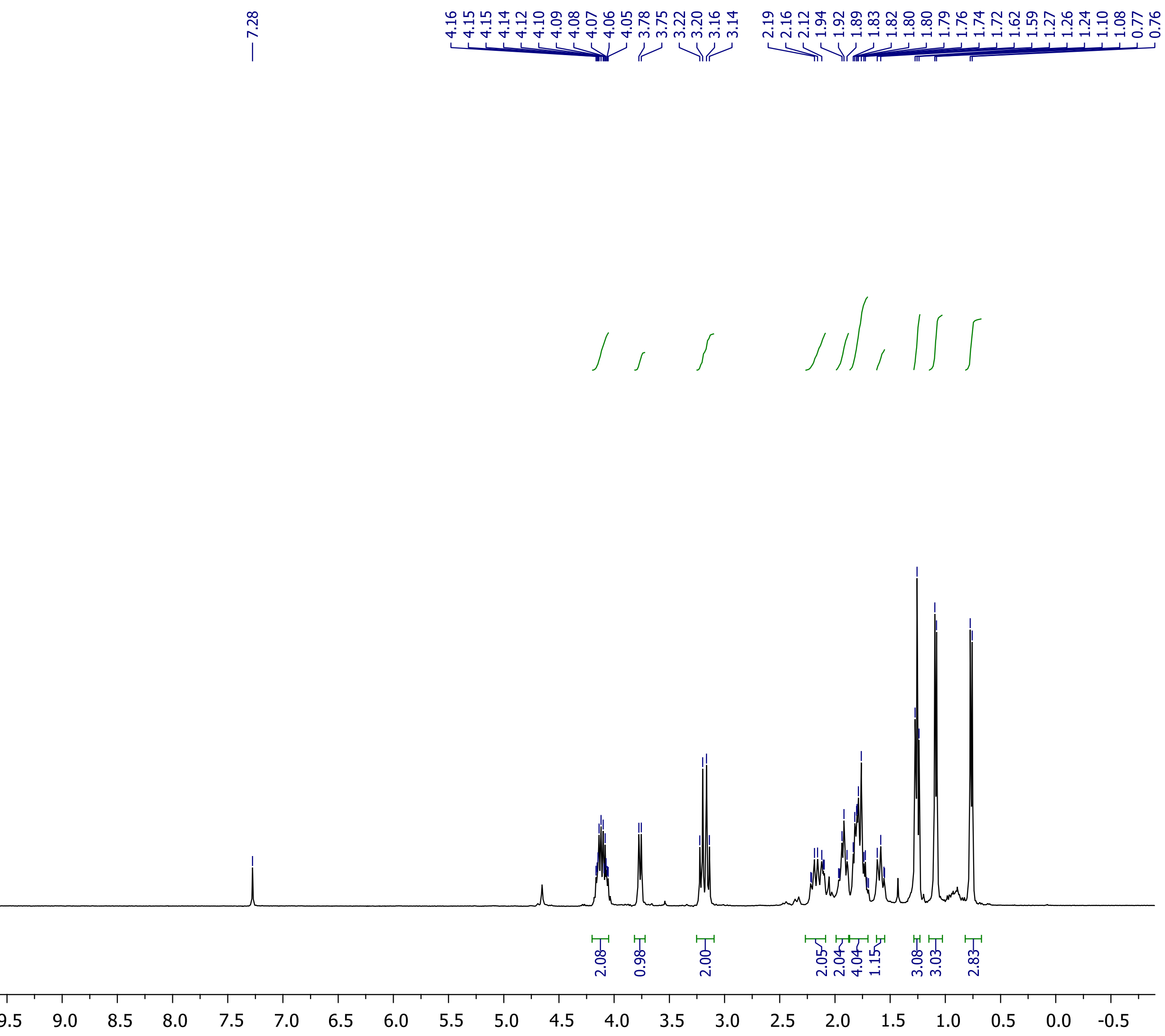
1H NMR (400 MHz, CDCl3)
R2708898
7.28
4.16 4.15 4.15 4.14 4.12 4.10 4.09 4.08 4.07 4.06 4.05 3.78 3.75 3.22 3.20 3.16 3.14
2.19 2.16 2.12 1.94 1.92 1.89 1.83 1.82 1.80 1.80 1.79 1.76 1.74 1.72 1.62 1.59 1.27 1.26 1.24 1.10 1.08 0.77 0.76
2.08 0.98 2.00 2.05 2.04 4.04 1.15 3.08 3.03 2.83
12.0 11.5 11.0 10.5 10.0 9.5 9.0 8.5 8.0 7.5 7.0 6.5 6.0 5.5 5.0 4.5 4.0 3.5 3.0 2.5 2.0 1.5 1.0 0.5 0.0 -0.5

$^{13}C\{^1H\}$ NMR (126 MHz, $CDCl_3$)

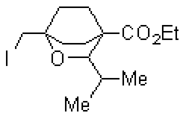

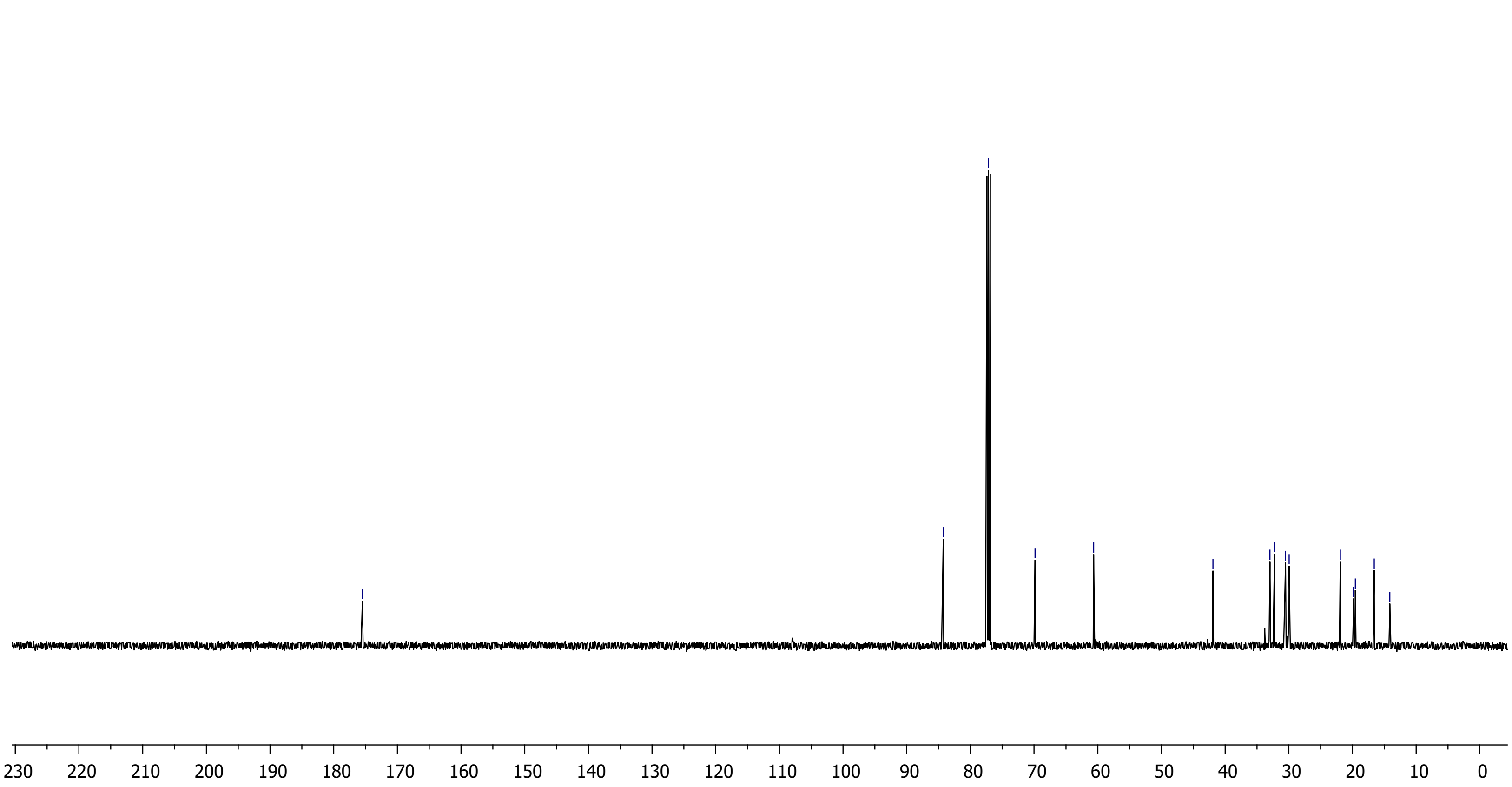

Compound 11

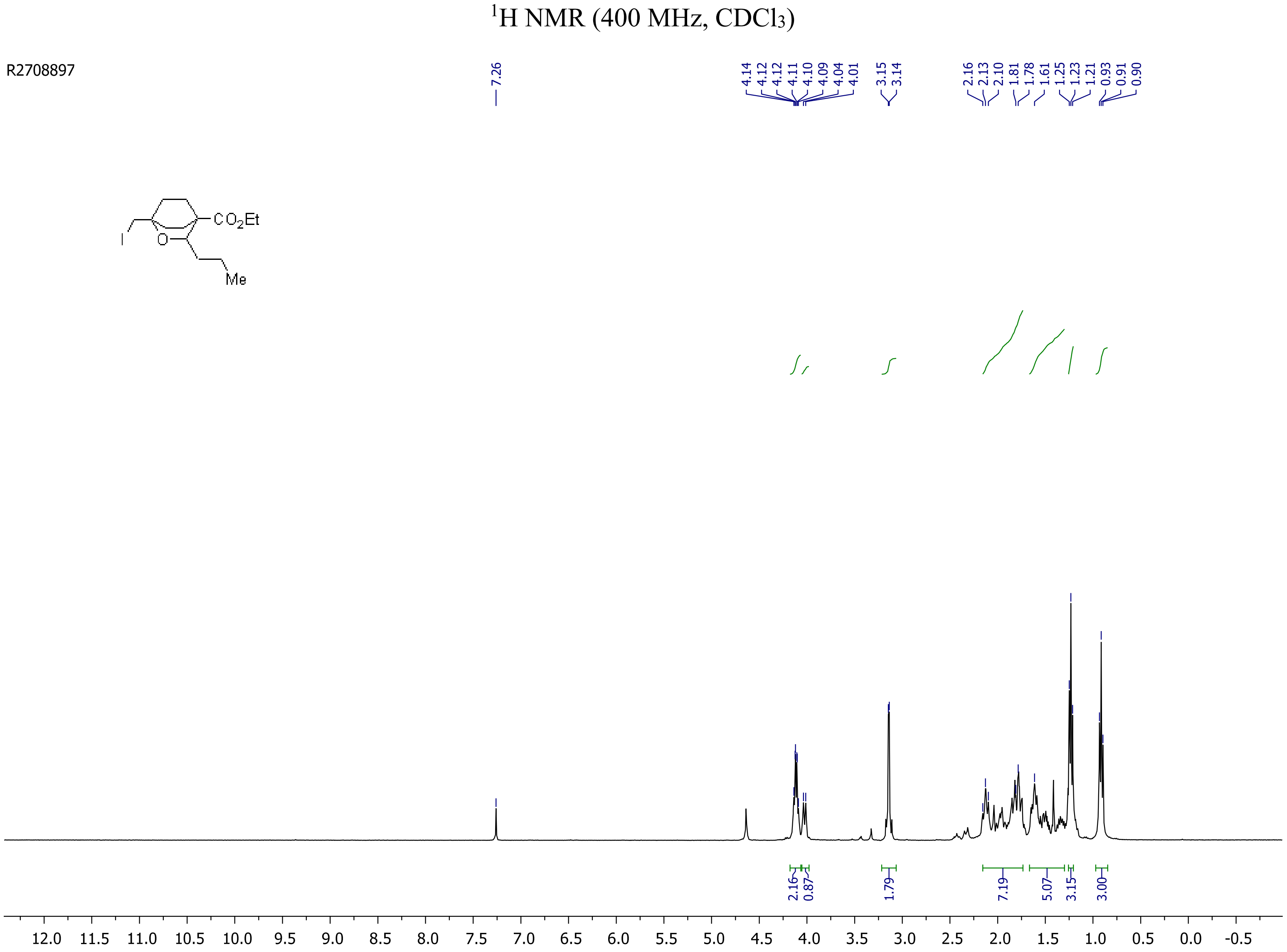

¹H NMR (400 MHz, CDCl3)
R2708897
7.26
4.14
4.12
4.12
4.11
4.10
4.09
4.04
4.01
3.15
3.14
2.16
2.13
2.10
1.81
1.78
1.61
1.25
1.23
1.21
0.93
0.91
0.90
CO2Et
I
O
Me
2.16
0.87
1.79
7.19
5.07
3.15
3.00
12.0
11.5
11.0
10.5
10.0
9.5
9.0
8.5
8.0
7.5
7.0
6.5
6.0
5.5
5.0
4.5
4.0
3.5
3.0
2.5
2.0
1.5
1.0
0.5
0.0
-0.5

$^{13}C\{^1H\}$ NMR (126 MHz, $CDCl_3$)

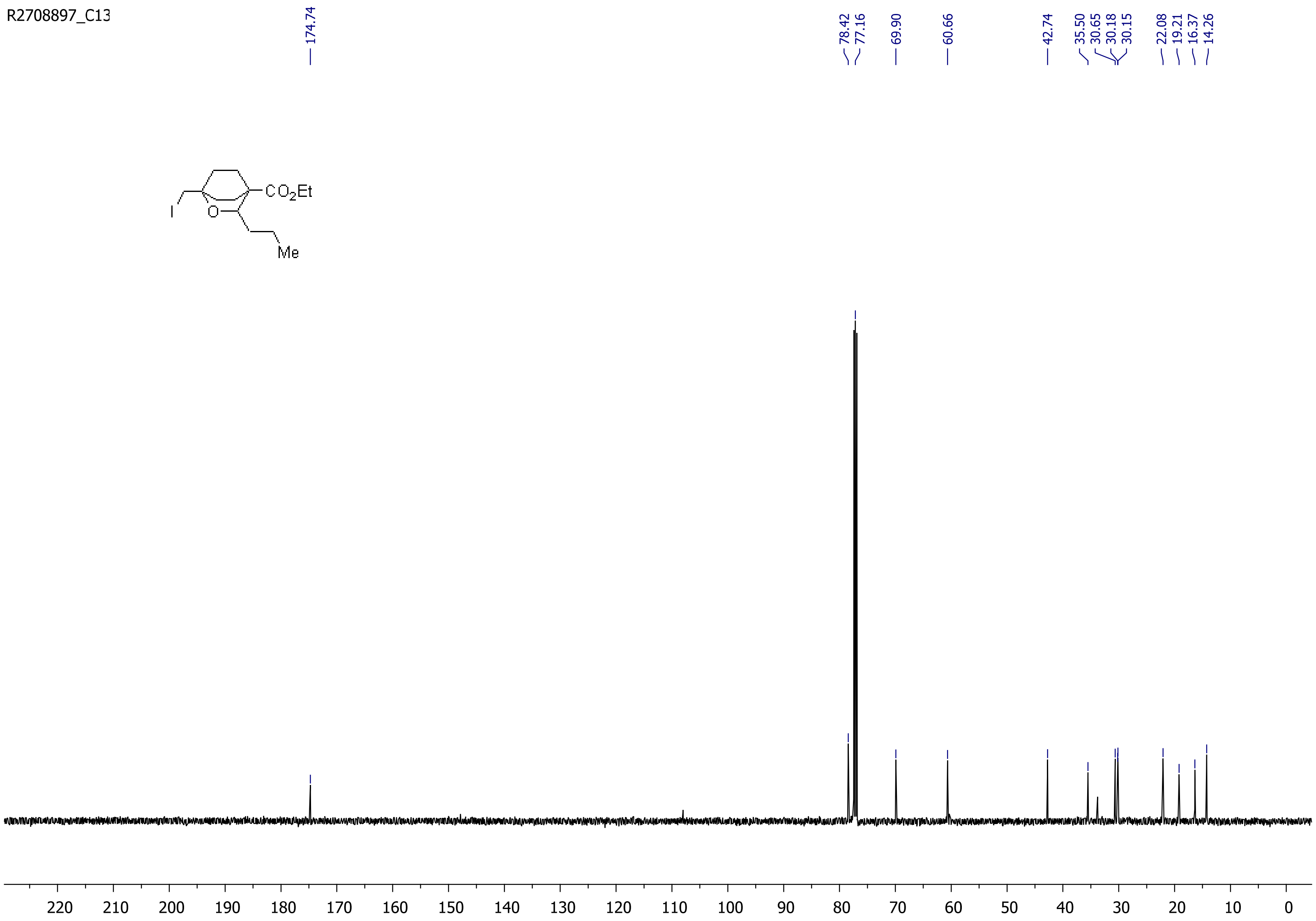

Compound 12

^{1}H NMR (400 MHz, $CDCl_3$)

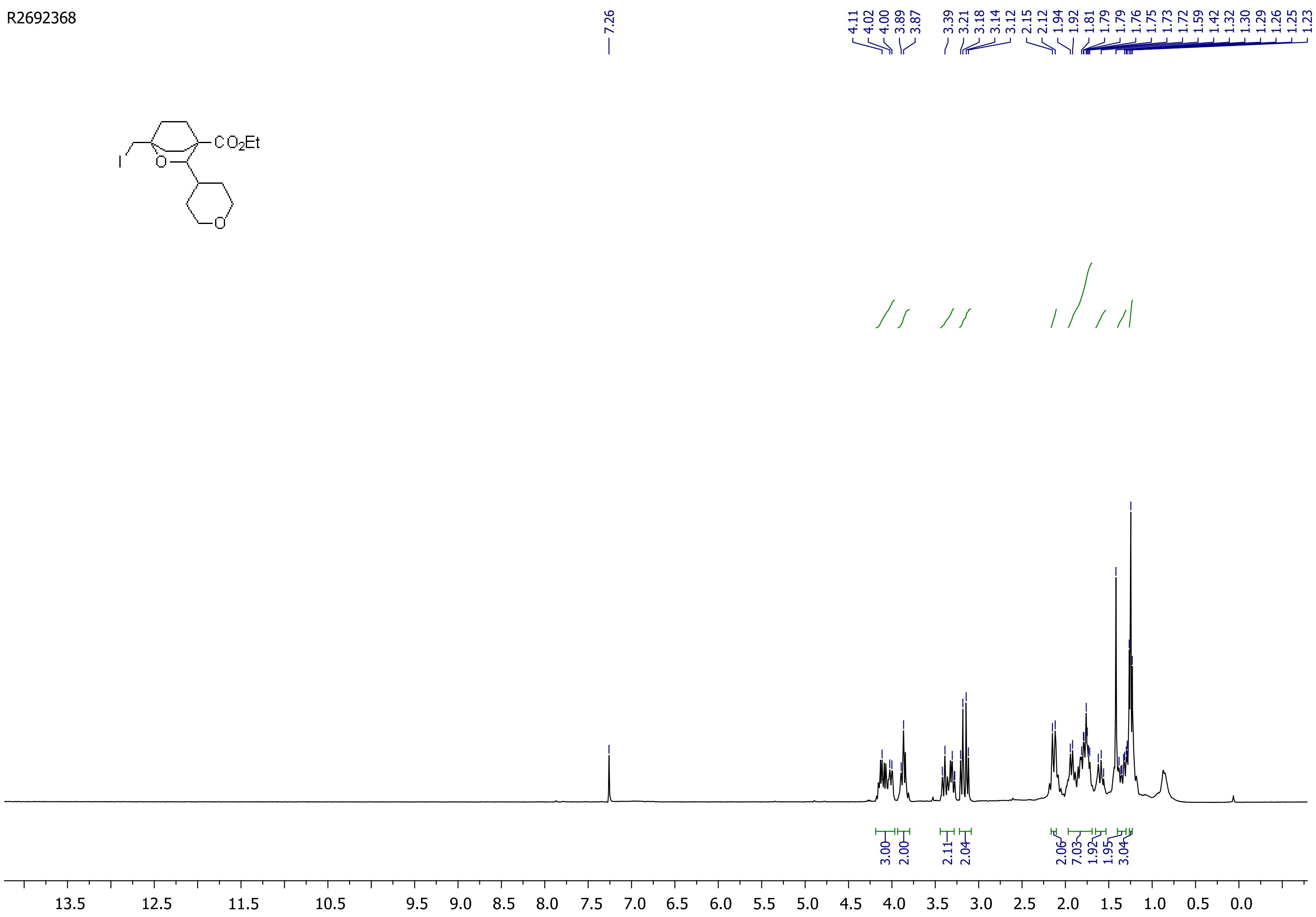

$^{13}C\{^1H\}$ NMR (151 MHz, $CDCl_3$)

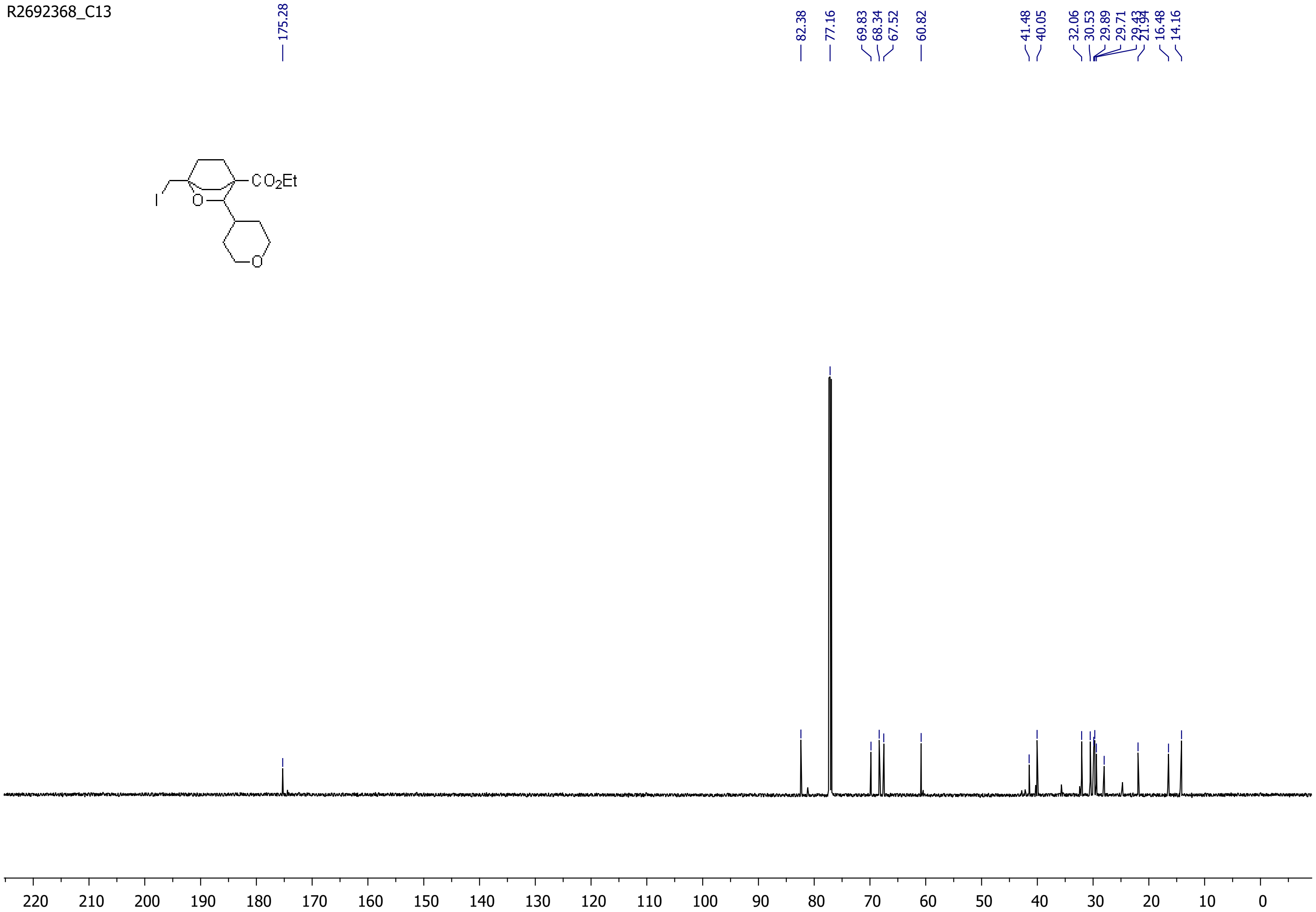

Compound 13

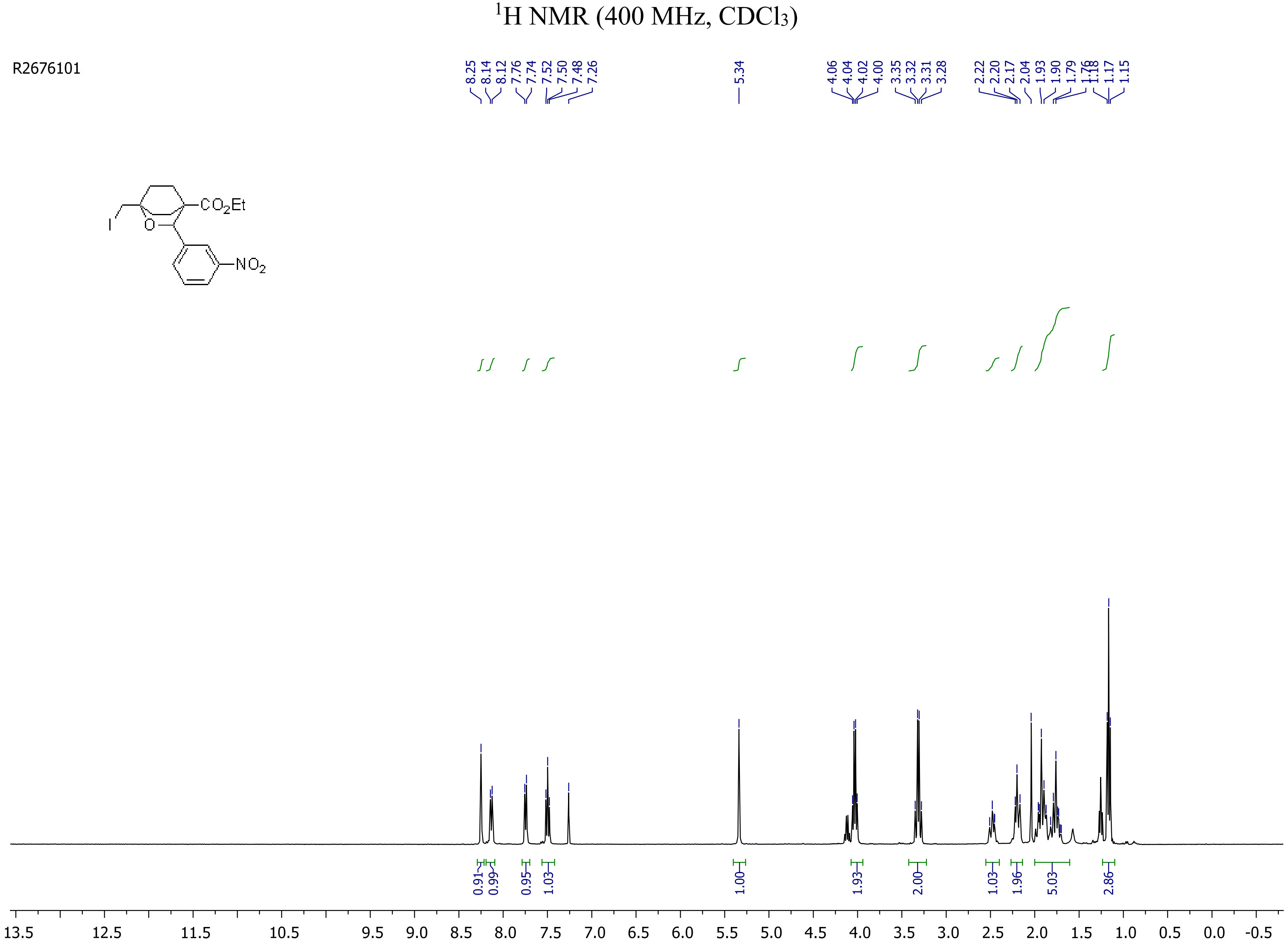

¹H NMR (400 MHz, CDCl3)
R2676101
8.25
8.14
8.12
7.76
7.74
7.52
7.50
7.48
7.26
5.34
4.06
4.04
4.02
4.00
3.35
3.32
3.31
3.28
2.22
2.20
2.17
2.04
1.93
1.90
1.79
1.76
1.18
1.17
1.15
CO2Et
I
O
NO2
0.91
0.99
0.95
1.03
1.00
1.93
2.00
1.03
1.96
5.03
2.86
13.5
12.5
11.5
10.5
9.5
9.0
8.5
8.0
7.5
7.0
6.5
6.0
5.5
5.0
4.5
4.0
3.5
3.0
2.5
2.0
1.5
1.0
0.5
0.0
-0.5

$^{13}C\{^1H\}$ NMR (151 MHz, $CDCl_3$)

R2676101_13C

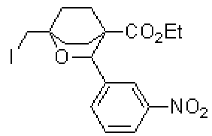

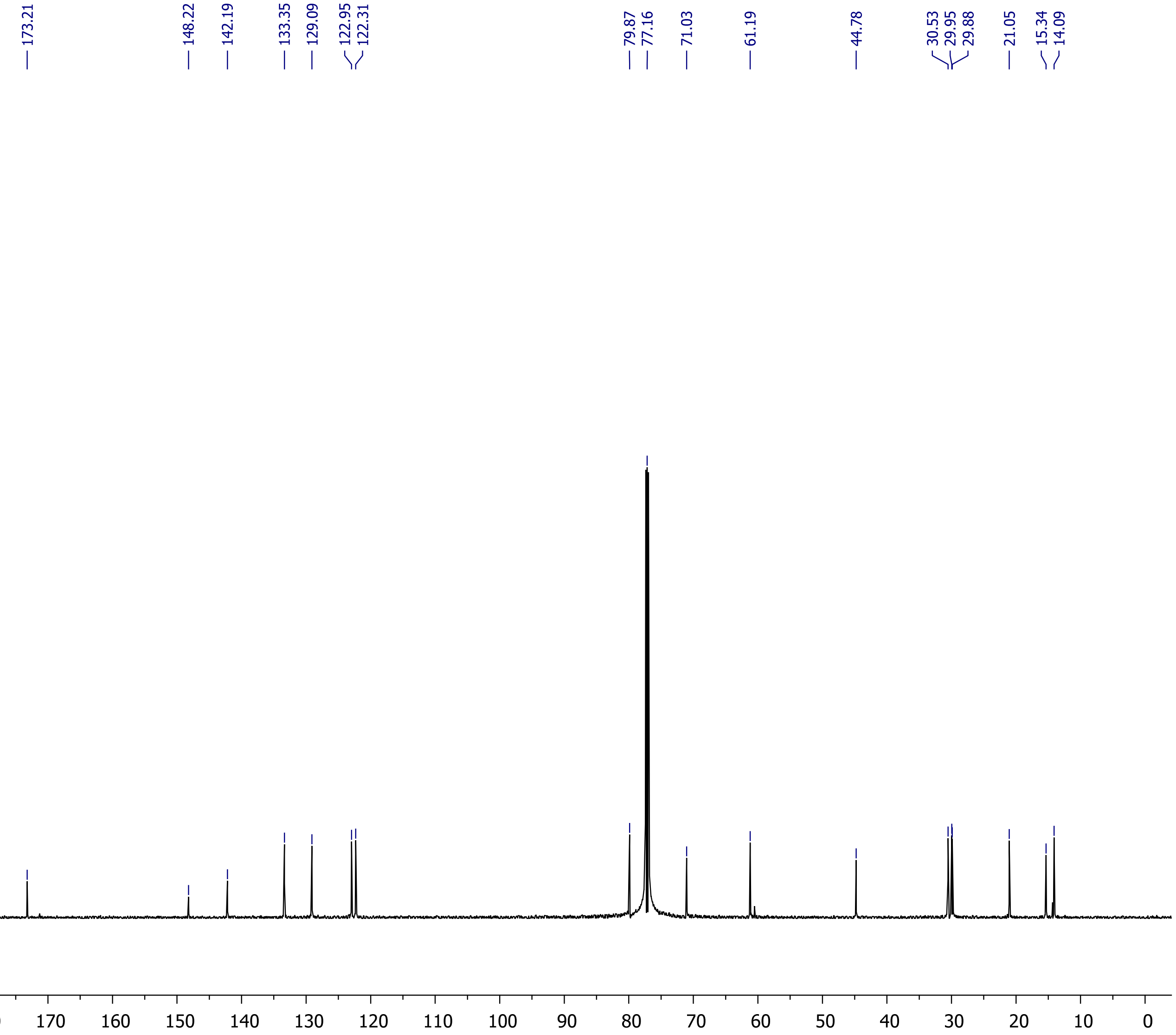

Compound 14

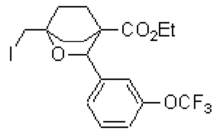

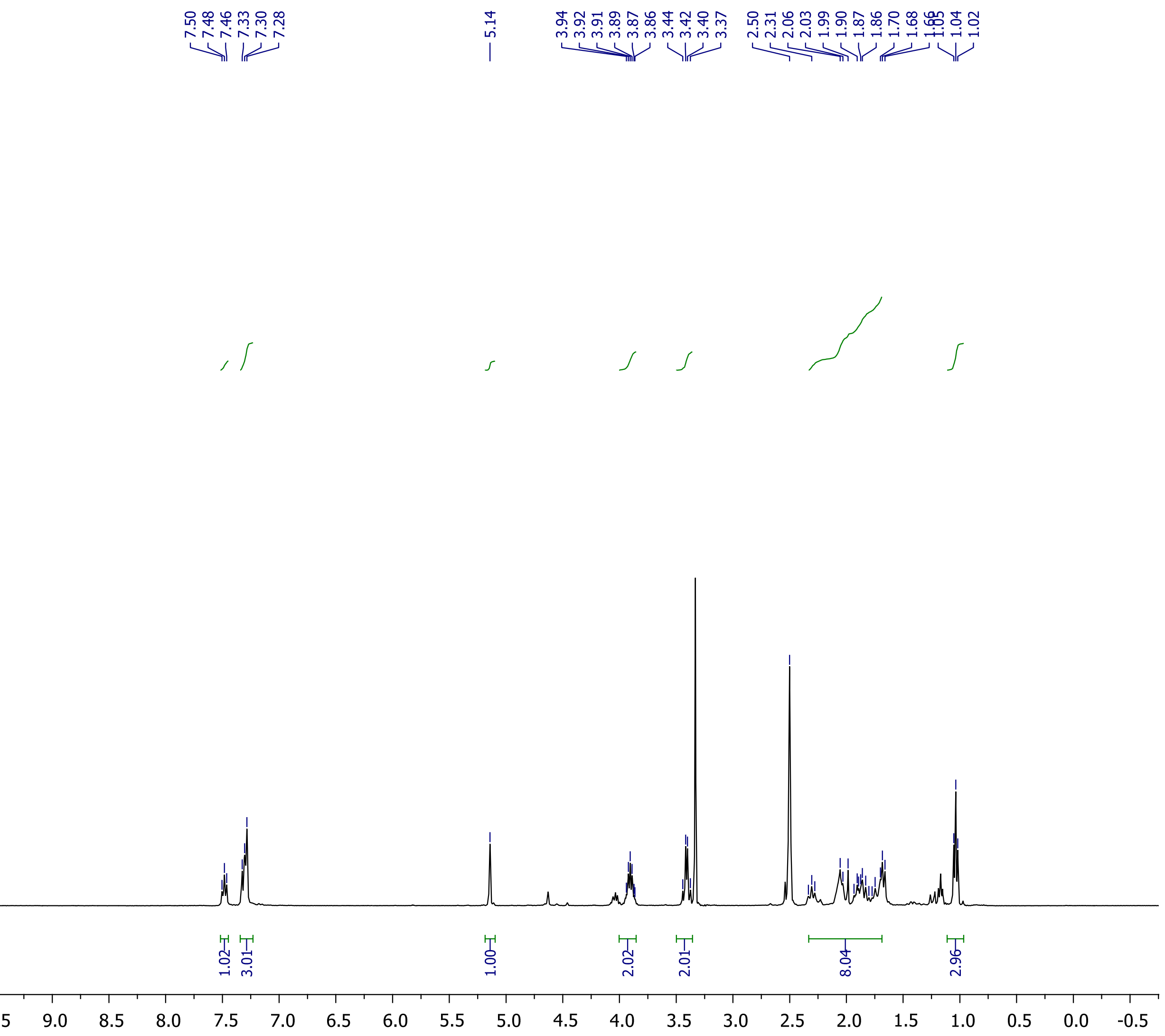

^{1}H NMR (400 MHz, DMSO-d6)
R2685296
7.50 7.48 7.46 7.33 7.30 7.28
5.14
3.94 3.92 3.91 3.89 3.87 3.86 3.44 3.42 3.40 3.37
2.50 2.31 2.06 2.03 1.99 1.90 1.87 1.86 1.70 1.68 1.66 1.05 1.04 1.02
1.02 3.01 1.00 2.02 2.01 8.04 2.96
12.0 11.5 11.0 10.5 10.0 9.5 9.0 8.5 8.0 7.5 7.0 6.5 6.0 5.5 5.0 4.5 4.0 3.5 3.0 2.5 2.0 1.5 1.0 0.5 0.0 -0.5

$^{13}C\{^1H\}$ NMR (151 MHz, DMSO-d6)

R2685296_13C

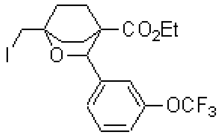

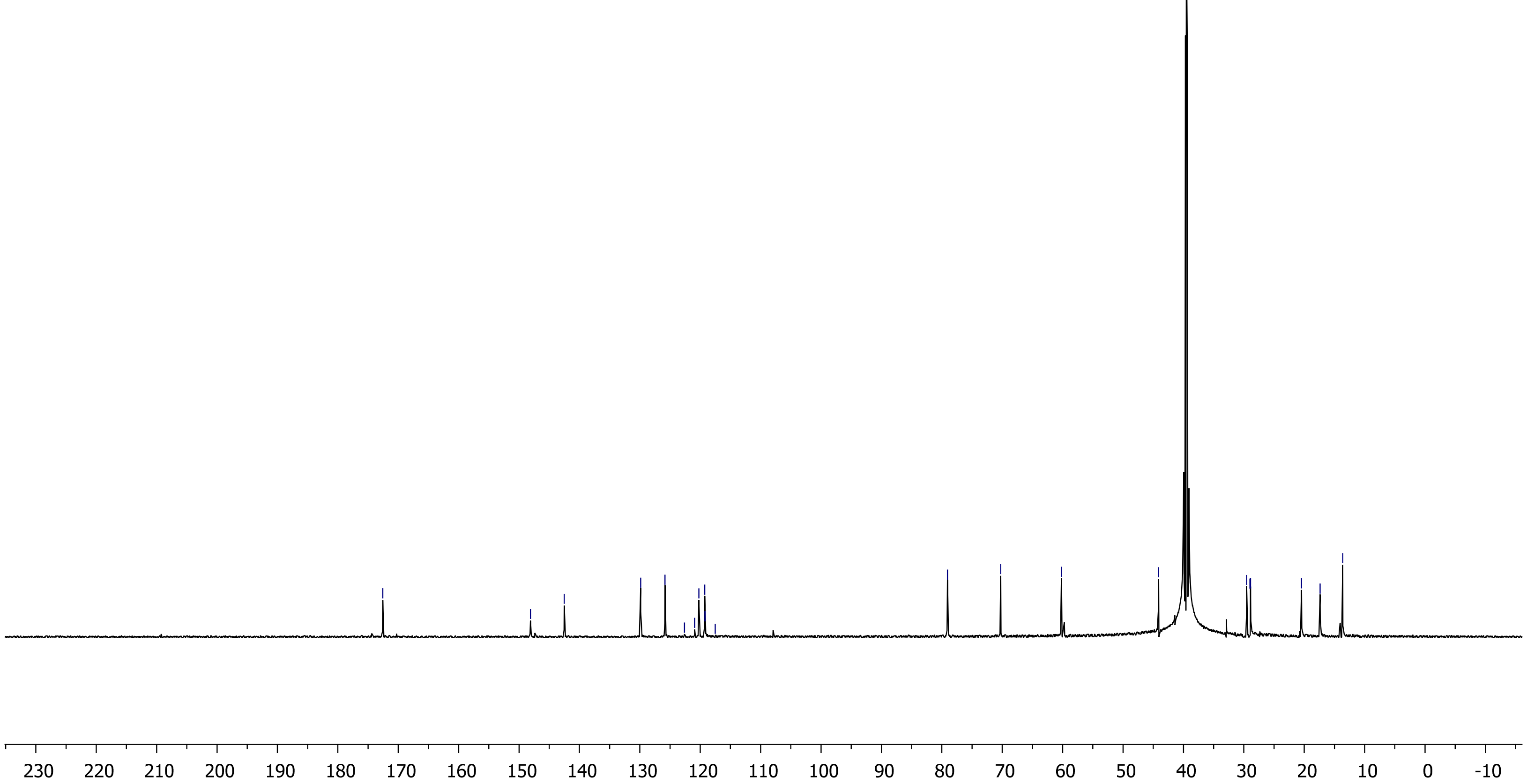

$^{19}F\{^1H\}$ NMR (376 MHz, DMSO-d6)

R2685296_F19{H}
19F-{1H}

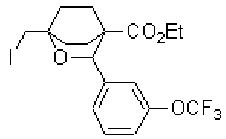

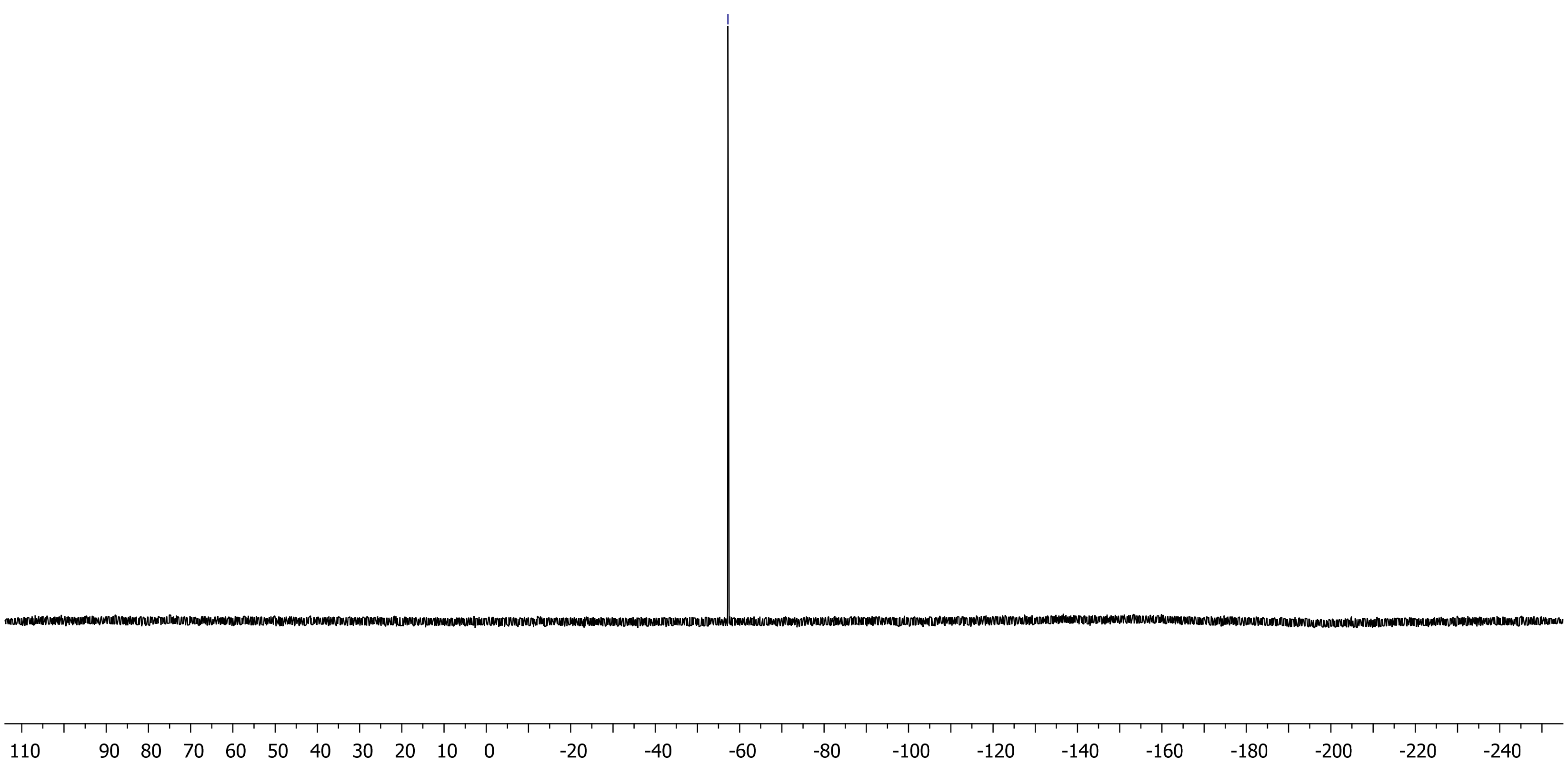

Compound 15

^{1}H NMR (400 MHz, DMSO-d$_6$)

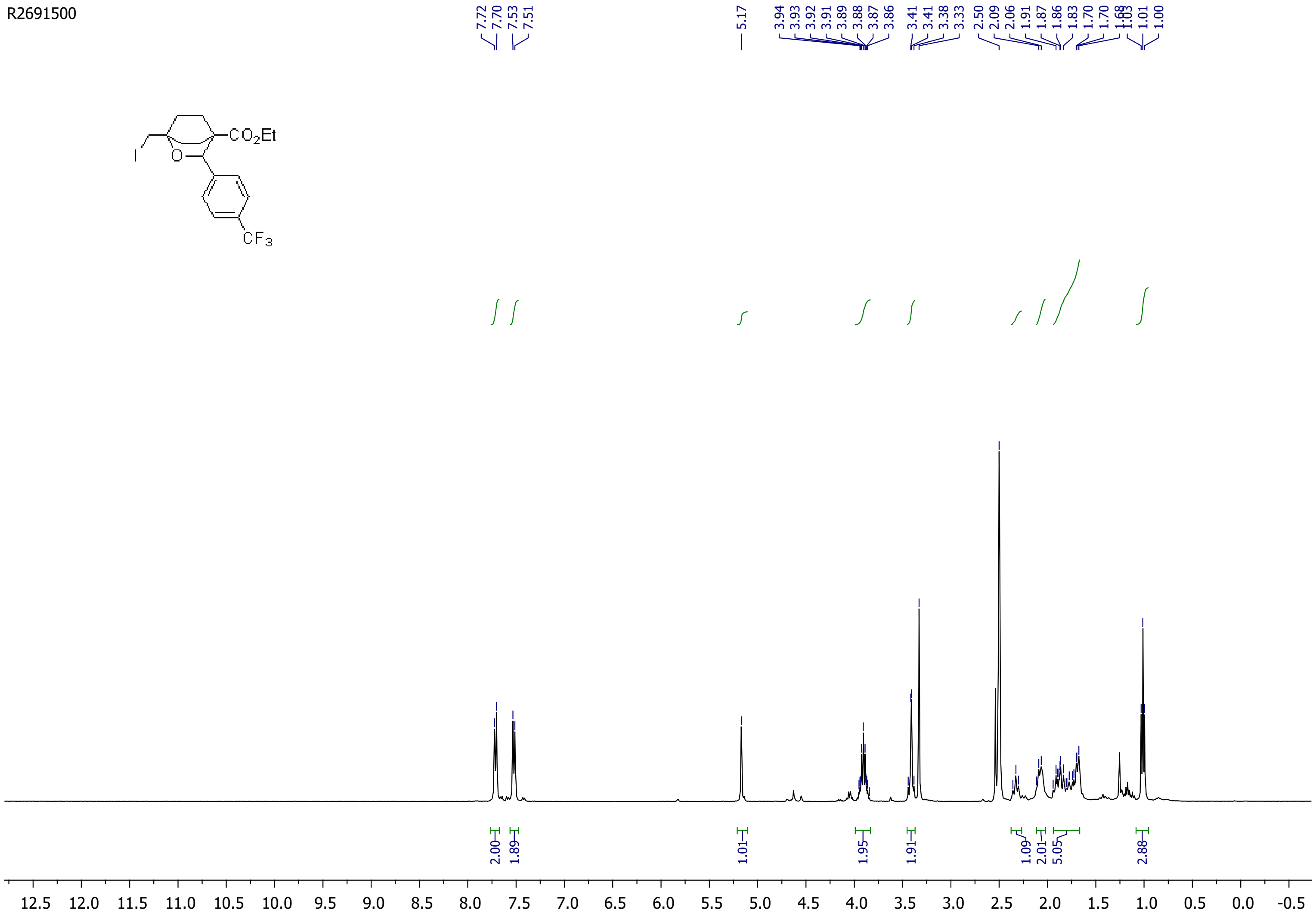

^{13}C{^{1}H} NMR (151 MHz, DMSO-d$_6$)

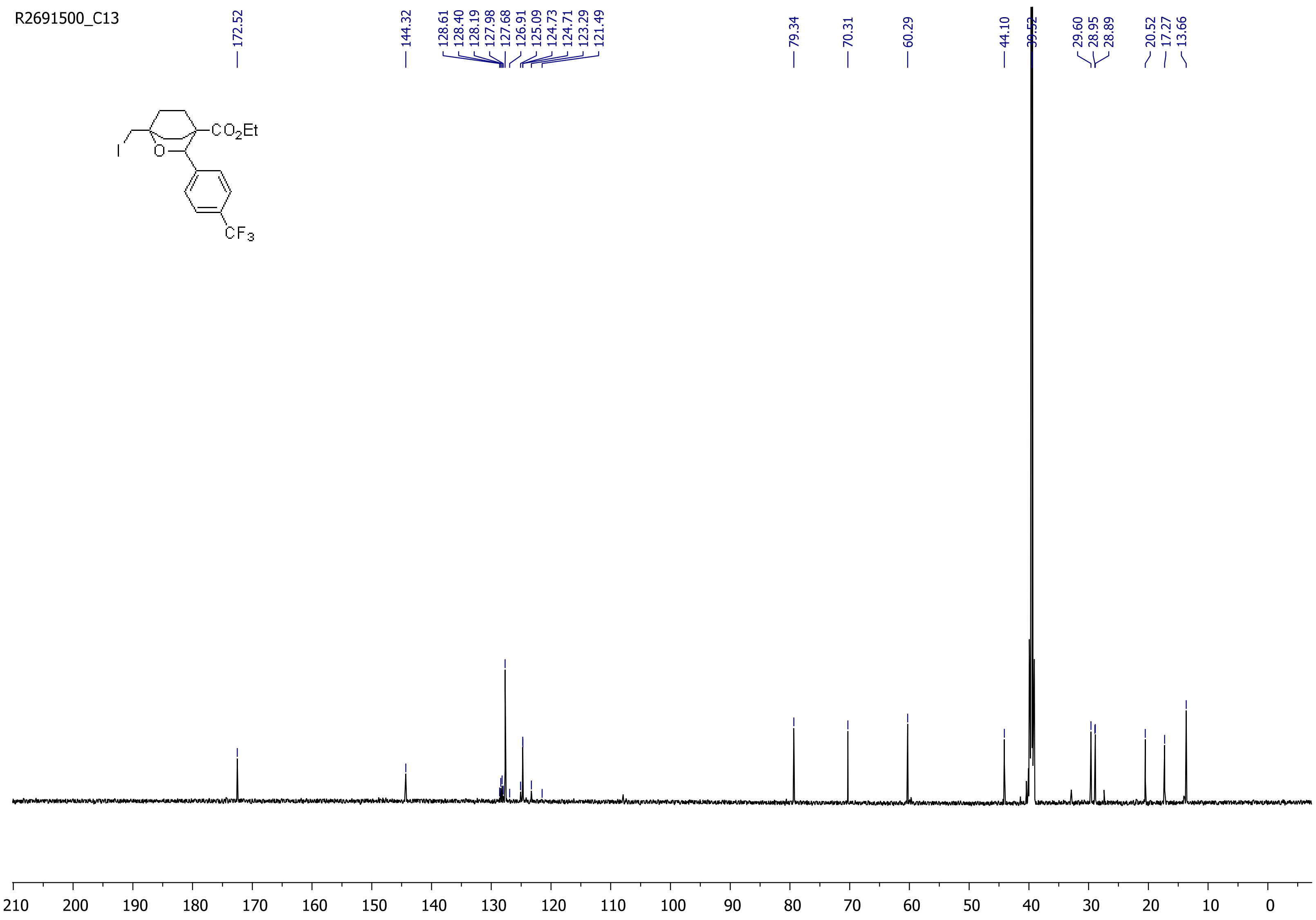

$^{19}F\{^1H\}$ NMR (376 MHz, DMSO-d$_6$)

R2691500_F19
19F-{1H}

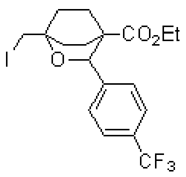

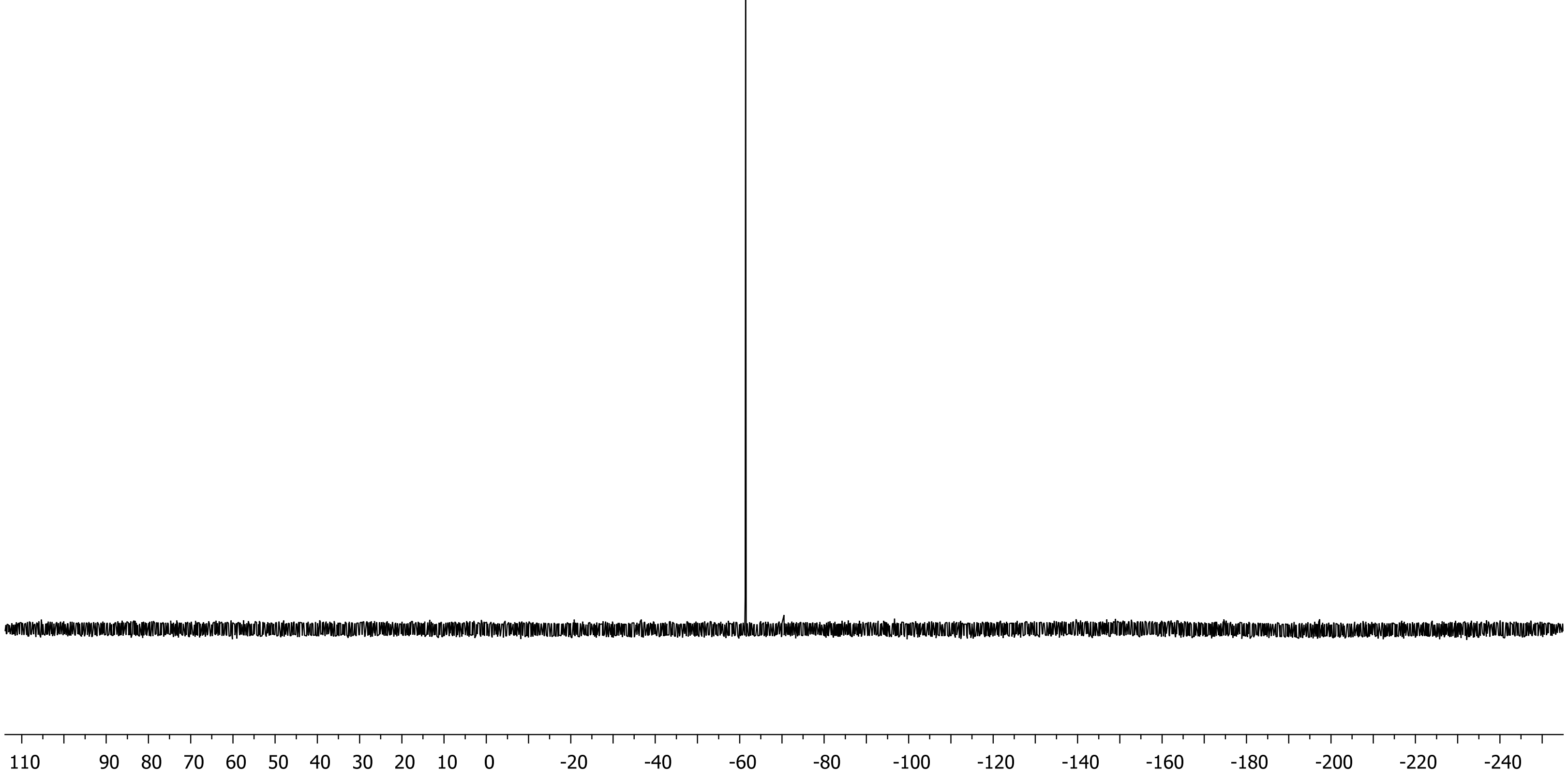

Compound 16

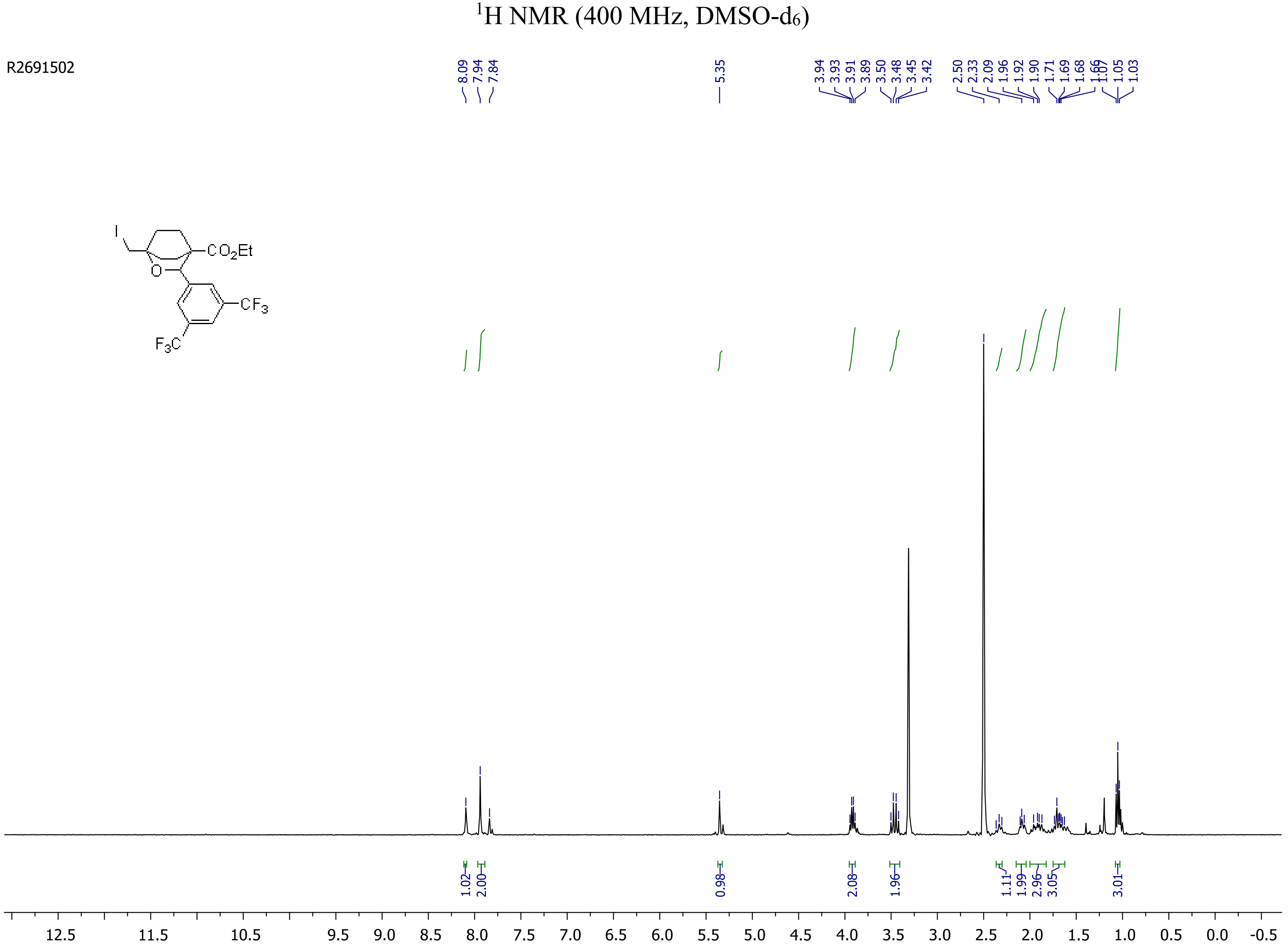

CO2Et
CF3
F3C
O
I
¹H NMR (400 MHz, DMSO-d6)
R2691502
8.09
7.94
7.84
5.35
3.94
3.93
3.91
3.89
3.50
3.48
3.45
3.42
2.50
2.33
2.09
1.96
1.92
1.90
1.71
1.69
1.68
1.66
1.07
1.05
1.03
1.02
2.00
0.98
2.08
1.96
1.11
1.99
2.96
3.05
3.01
12.5
11.5
10.5
9.5
9.0
8.5
8.0
7.5
7.0
6.5
6.0
5.5
5.0
4.5
4.0
3.5
3.0
2.5
2.0
1.5
1.0
0.5
0.0
-0.5

$^{13}C\{^1H\}$ NMR (126 MHz, DMSO-d6)

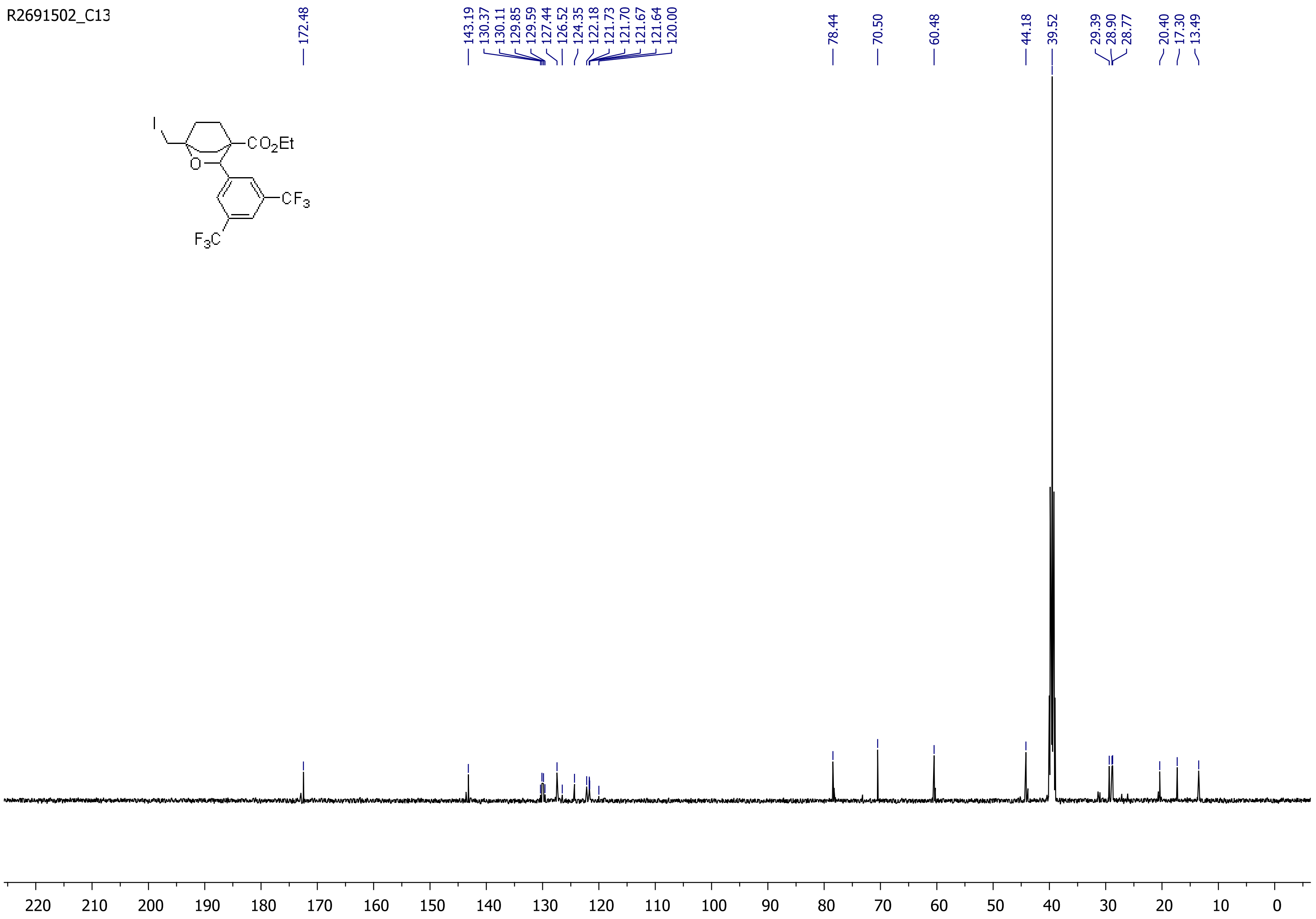

$^{19}F\{^{1}H\}$ NMR (376 MHz, DMSO-d_6)

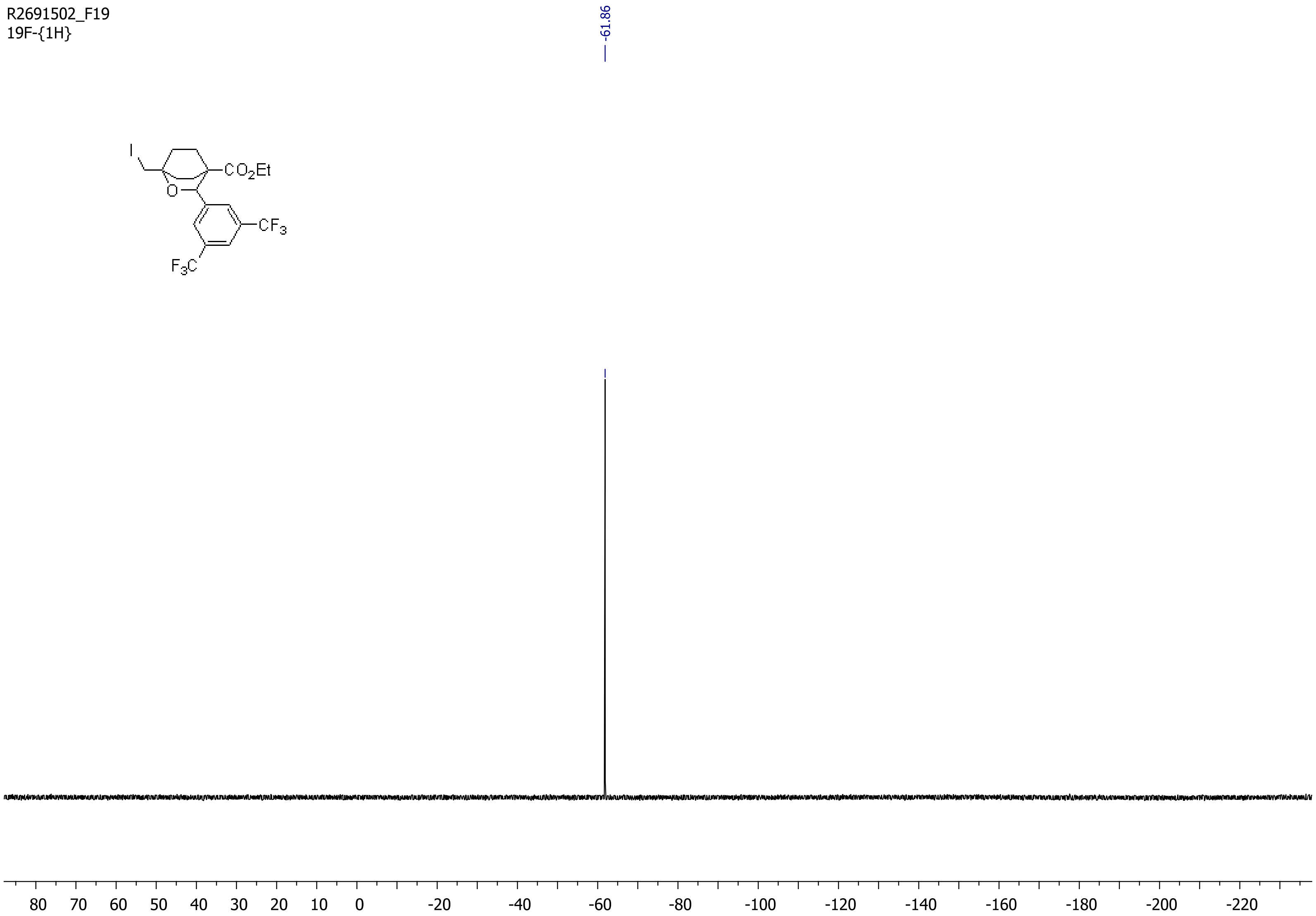

Compound 17

^{1}H NMR (400 MHz, DMSO-d$_6$)

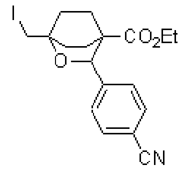

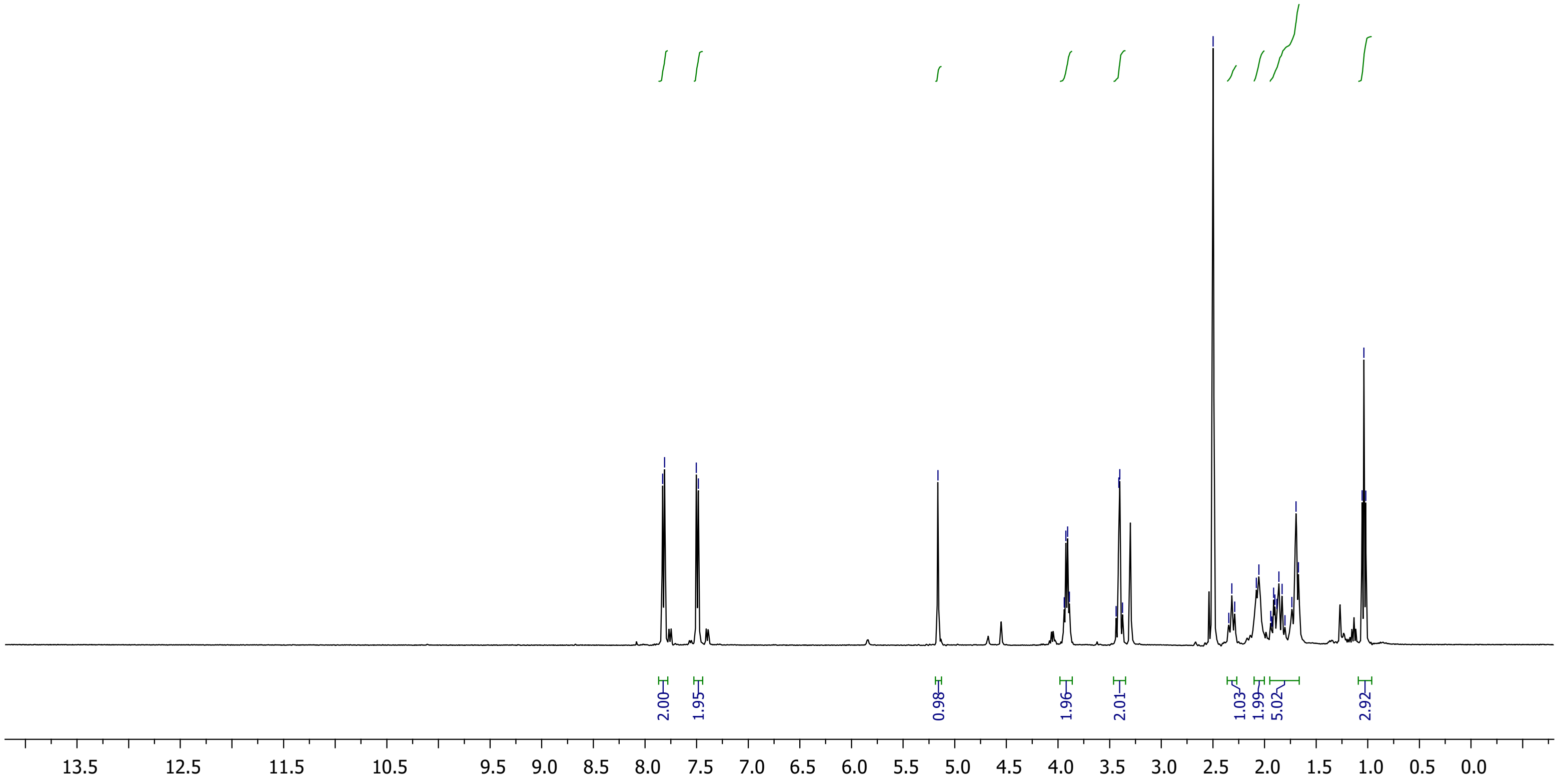

$^{13}C\{^{1}H\}$ NMR (126 MHz, DMSO-d6)

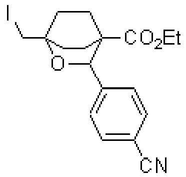

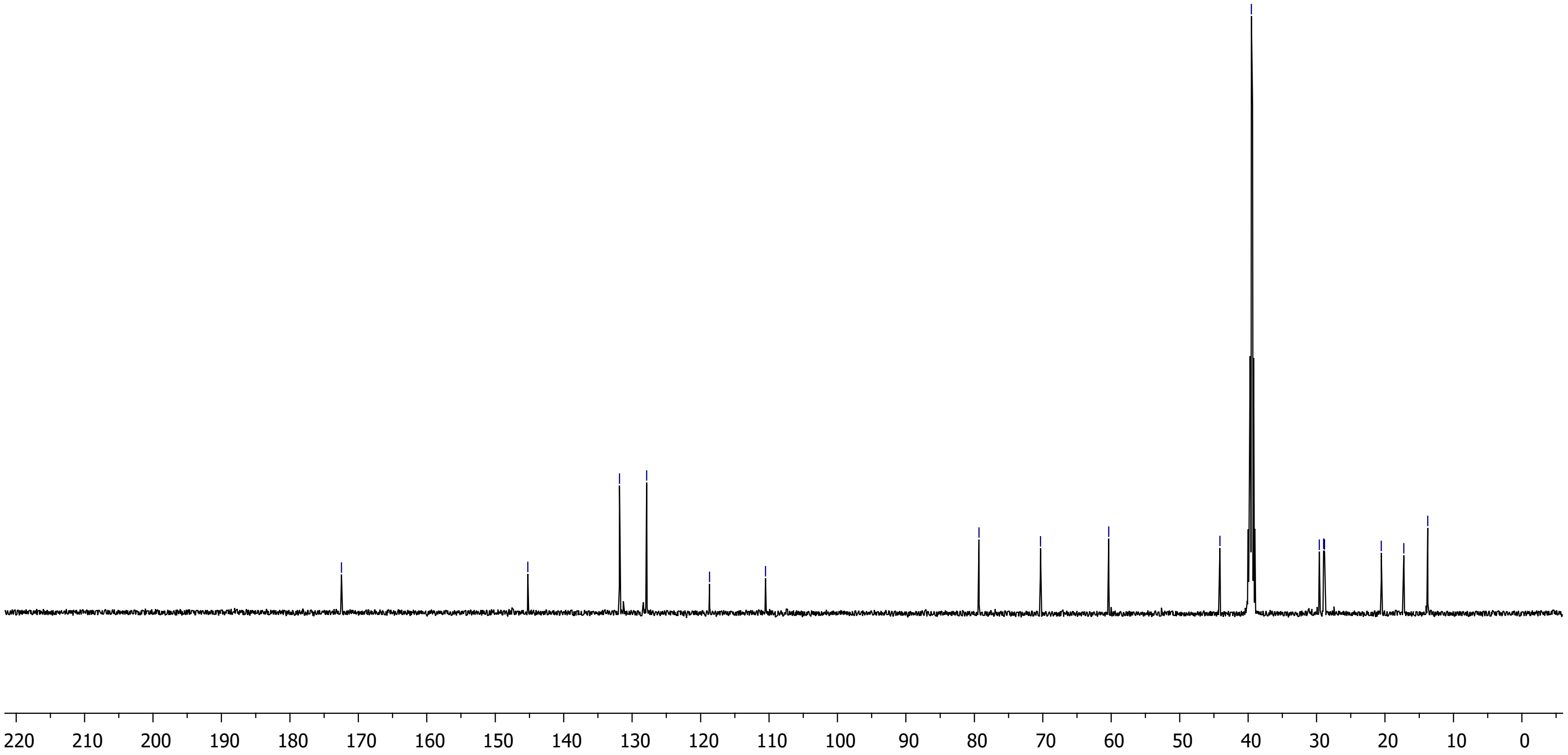

Compound 18

^1H NMR (400 MHz, DMSO-d$_6$)

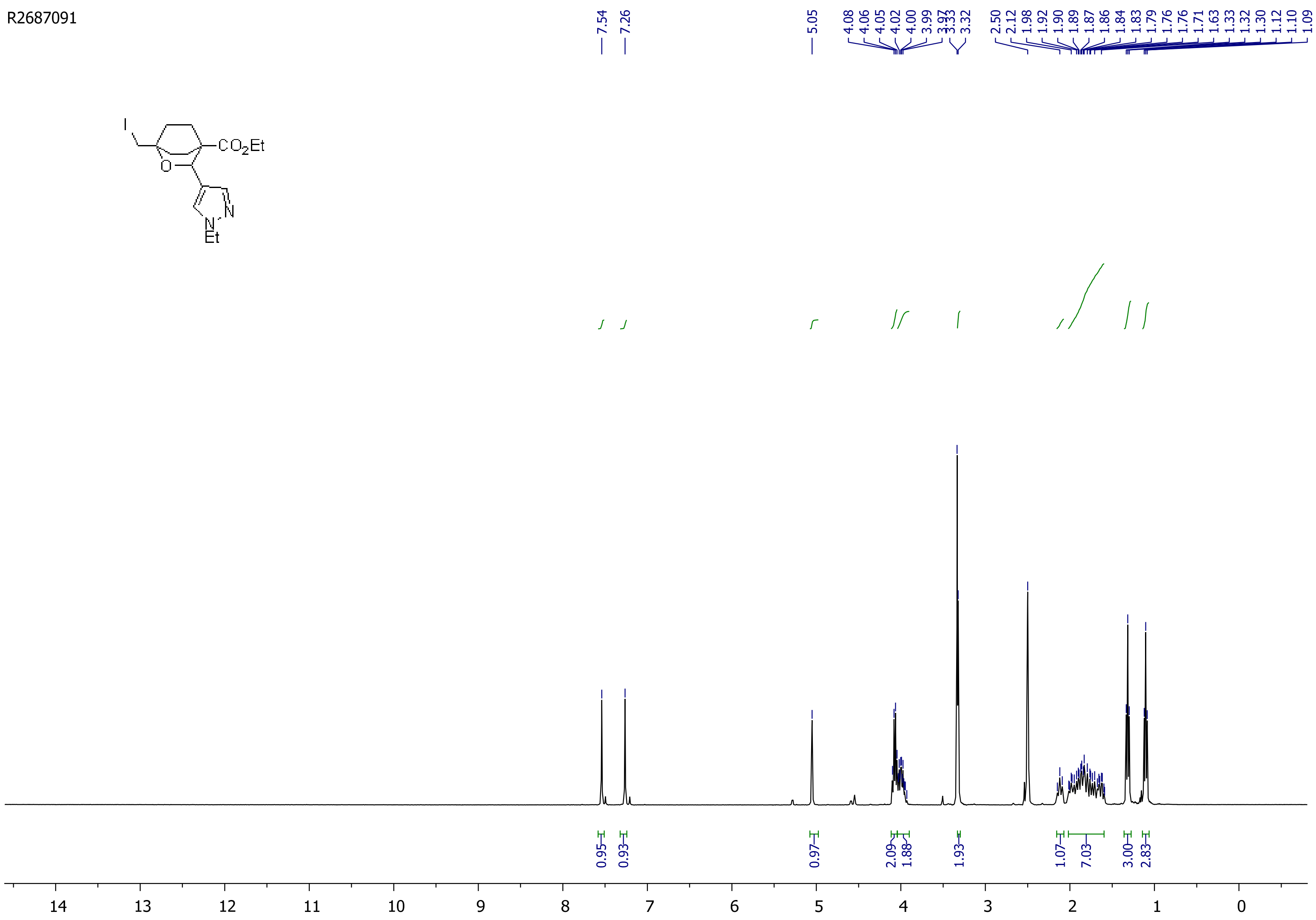

$^{13}C\{^1H\}$ NMR (126 MHz, DMSO-d6)

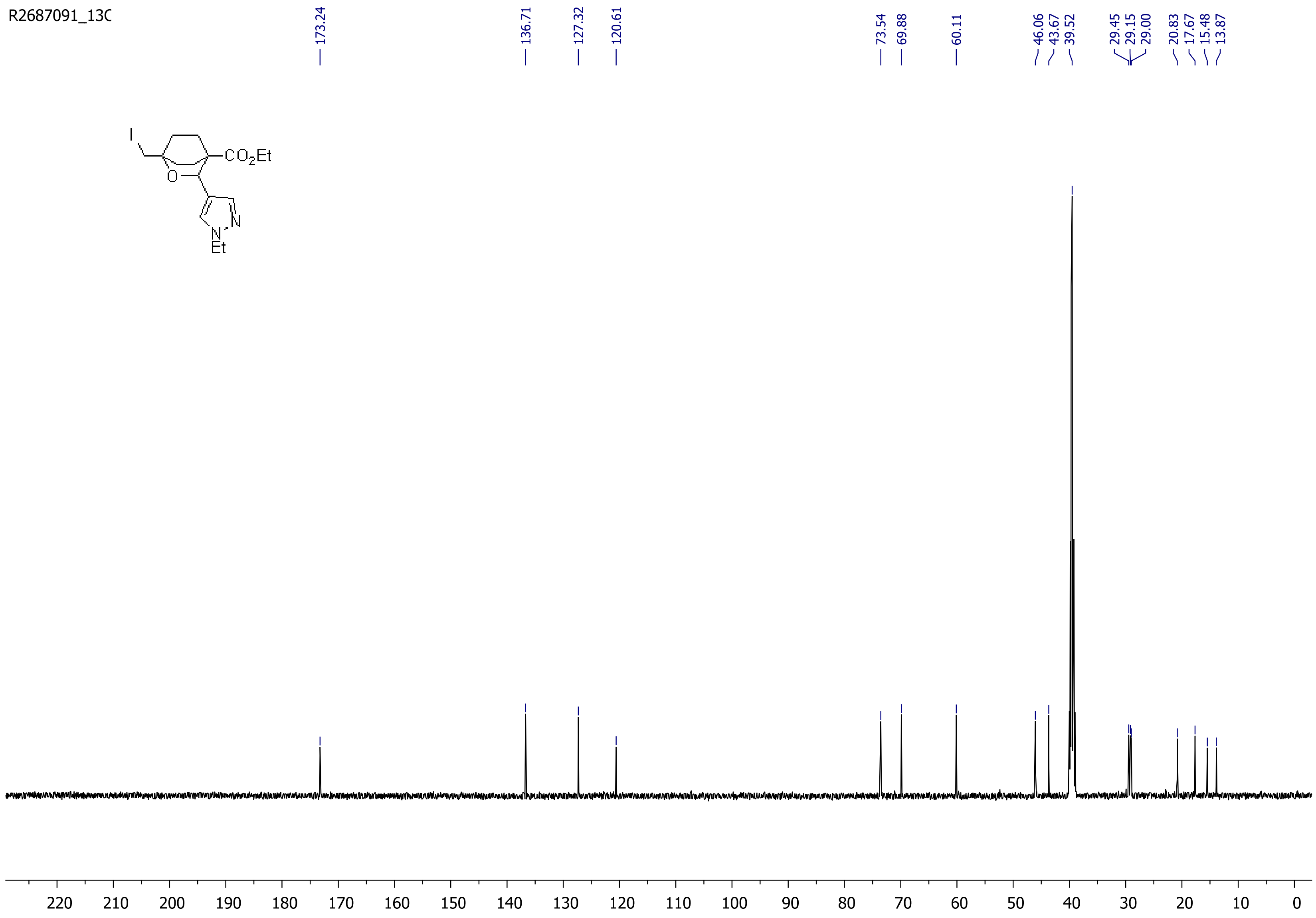

Compound 19

^{1}H NMR (400 MHz, DMSO-d$_6$)

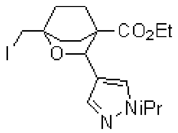

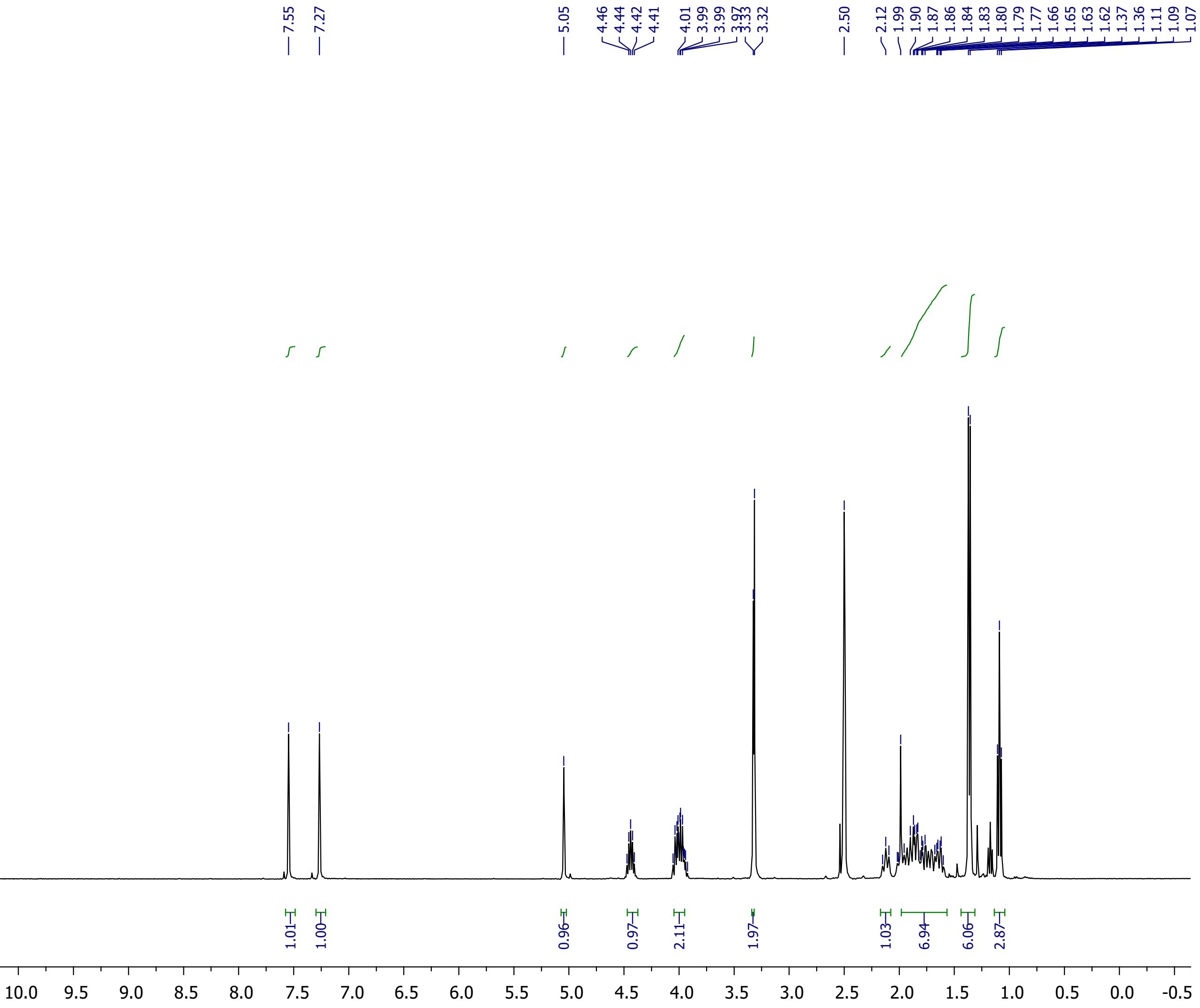

$^{13}C\{^{1}H\}$ NMR (126 MHz, DMSO-d$_6$)

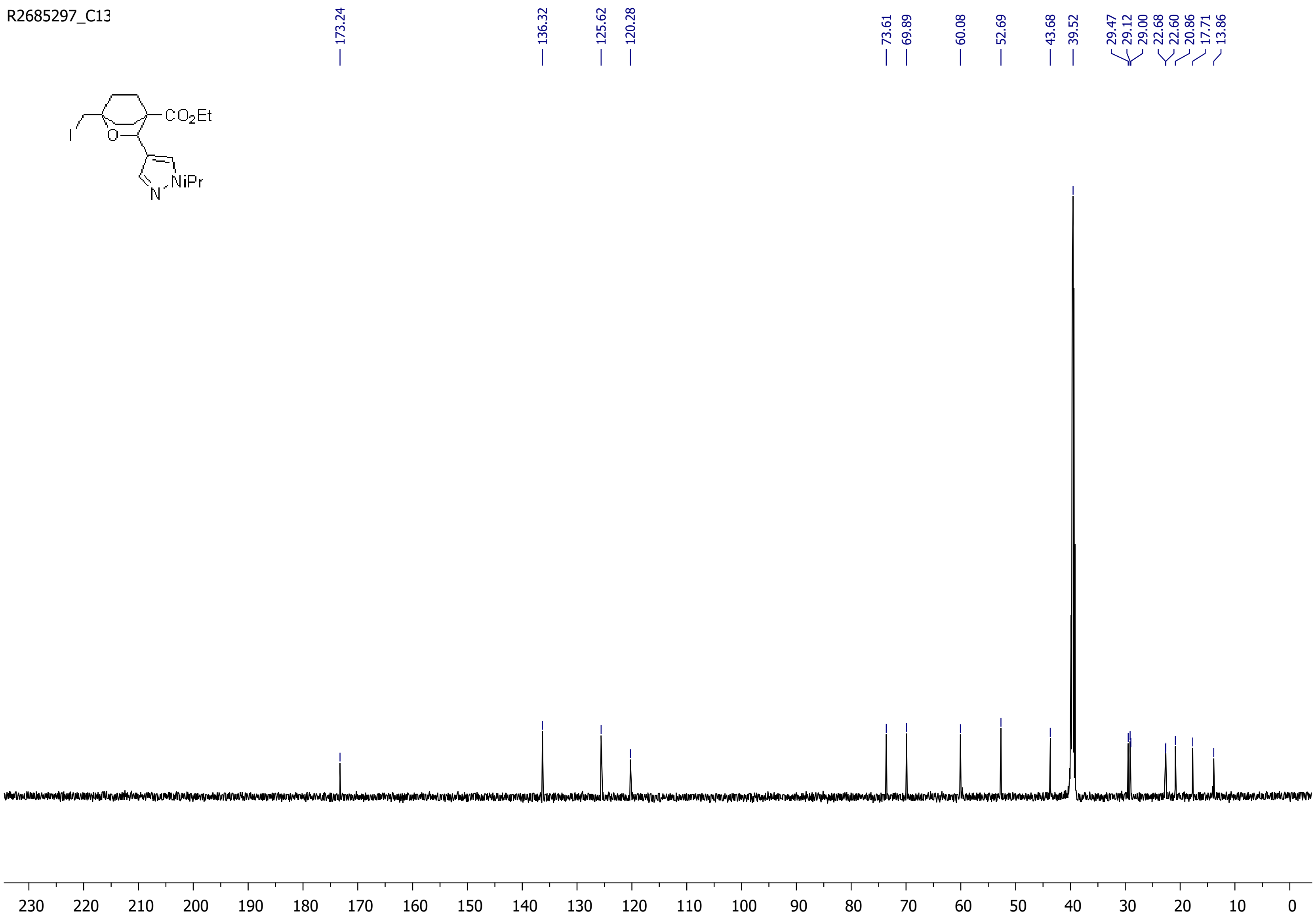

Compound 20

^1H NMR (400 MHz, DMSO-d_6)

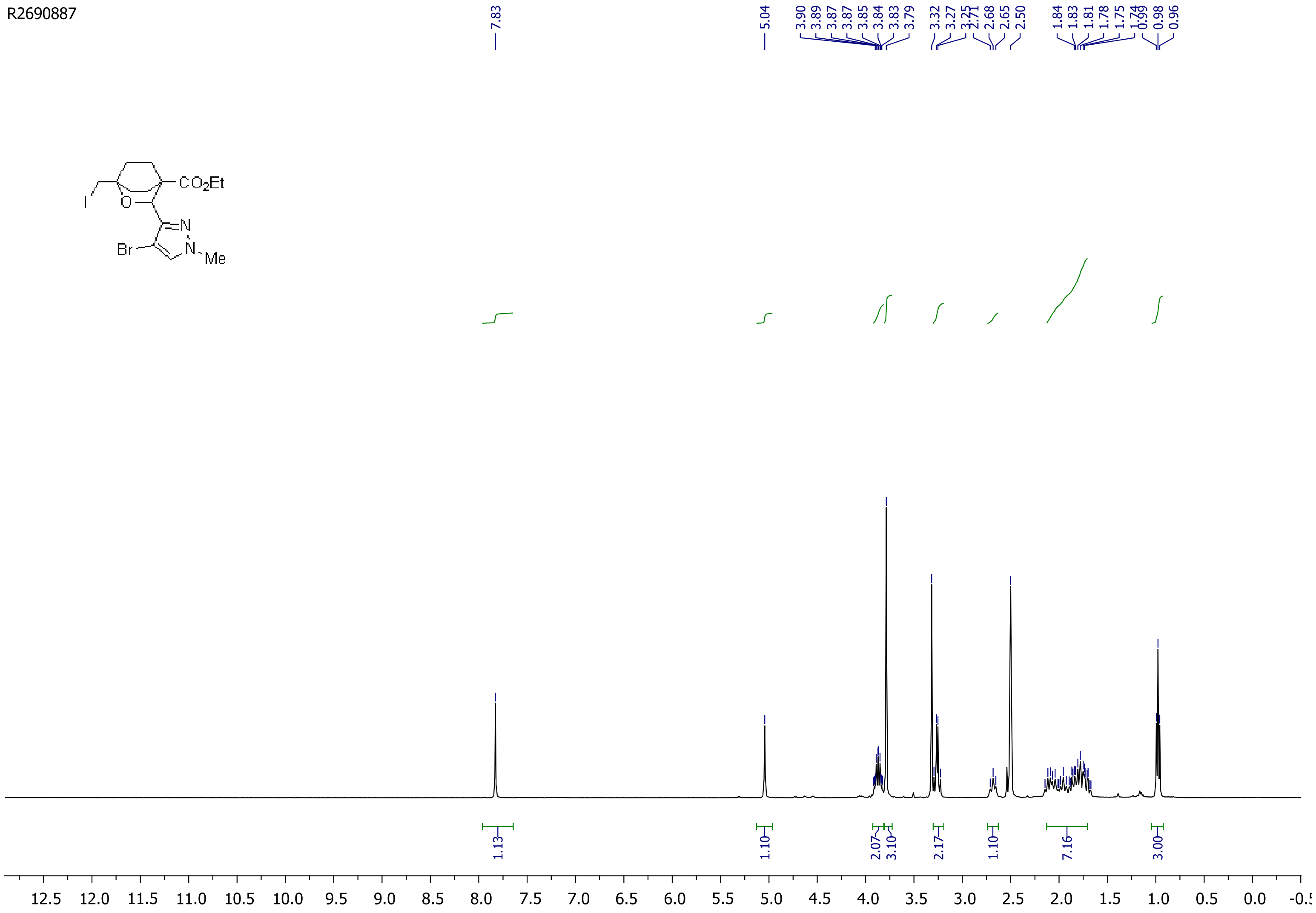

$^{13}C\{^1H\}$ NMR (126 MHz, DMSO-d6)

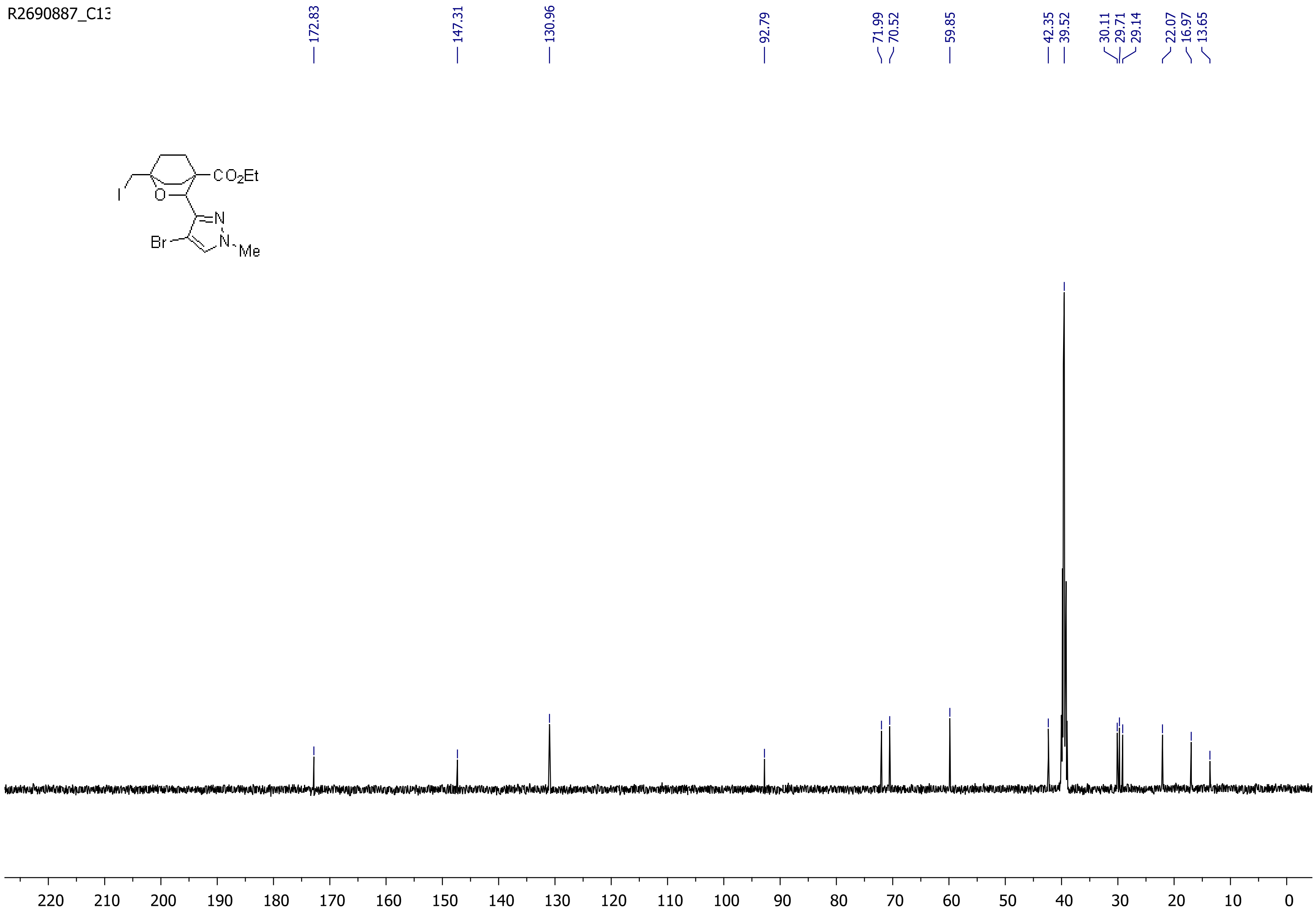

Compound 21

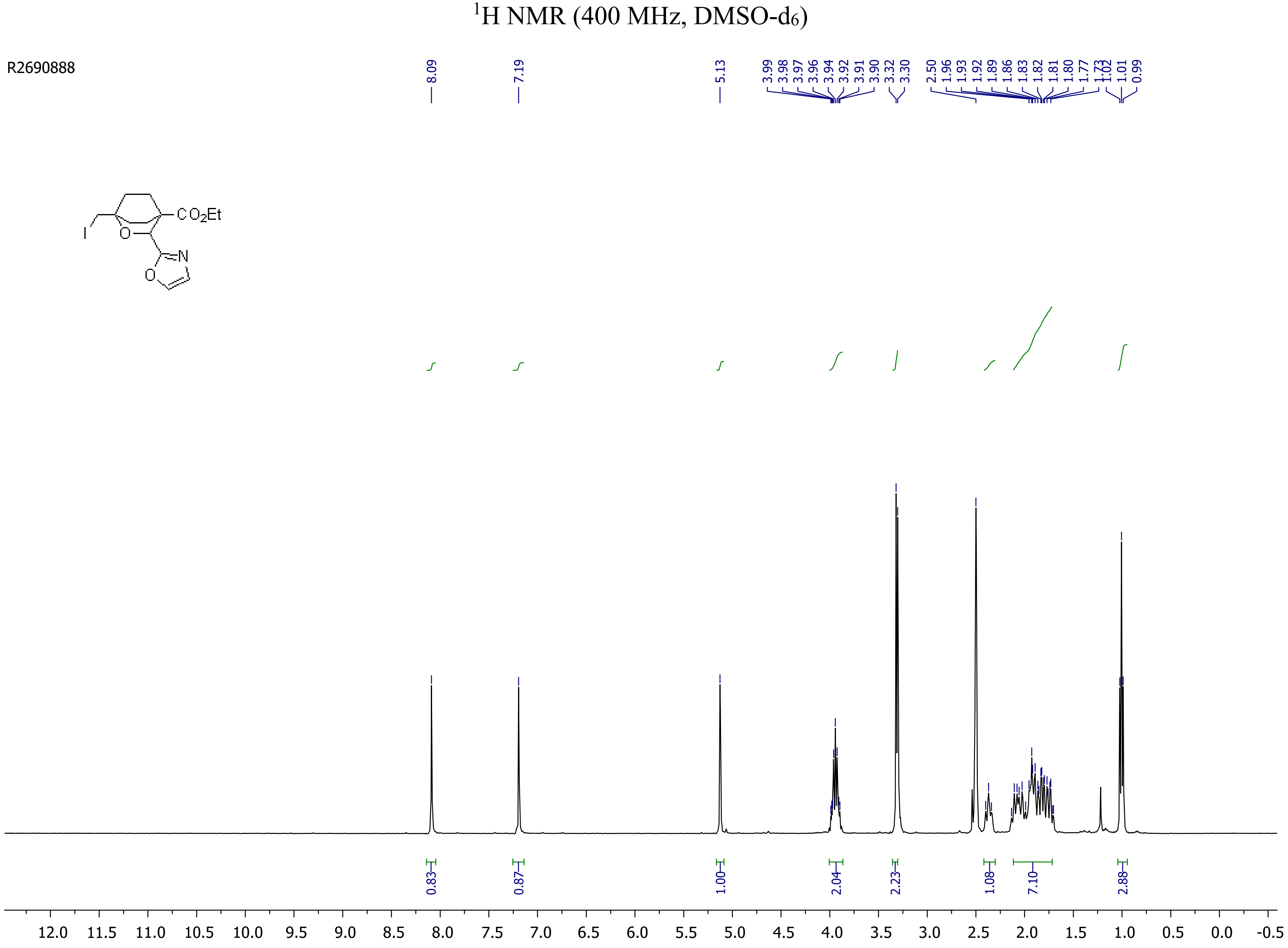

¹H NMR (400 MHz, DMSO-d6)
R2690888
8.09
7.19
5.13
3.99
3.98
3.97
3.96
3.94
3.92
3.91
3.90
3.32
3.30
2.50
1.96
1.93
1.92
1.89
1.86
1.83
1.82
1.81
1.80
1.77
1.73
1.03
1.01
0.99
0.83
0.87
1.00
2.04
2.23
1.08
7.10
2.88
12.0 11.5 11.0 10.5 10.0 9.5 9.0 8.5 8.0 7.5 7.0 6.5 6.0 5.5 5.0 4.5 4.0 3.5 3.0 2.5 2.0 1.5 1.0 0.5 0.0 -0.5

$^{13}C\{^{1}H\}$ NMR (126 MHz, DMSO-d6)

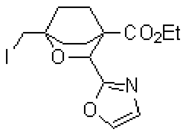

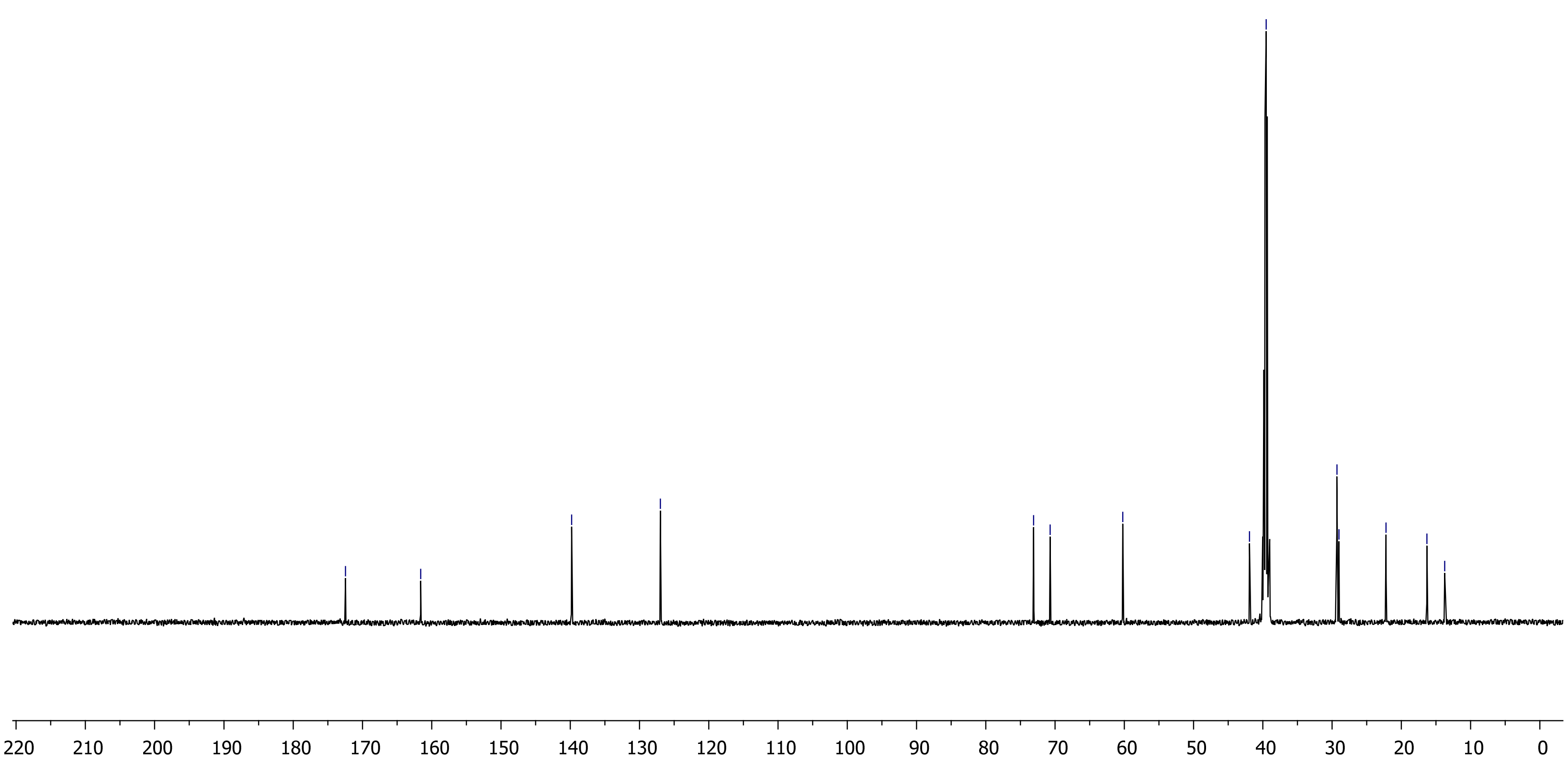

Compound 22

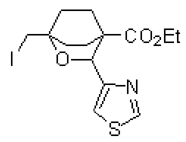

CO2Et
I
O
N
S

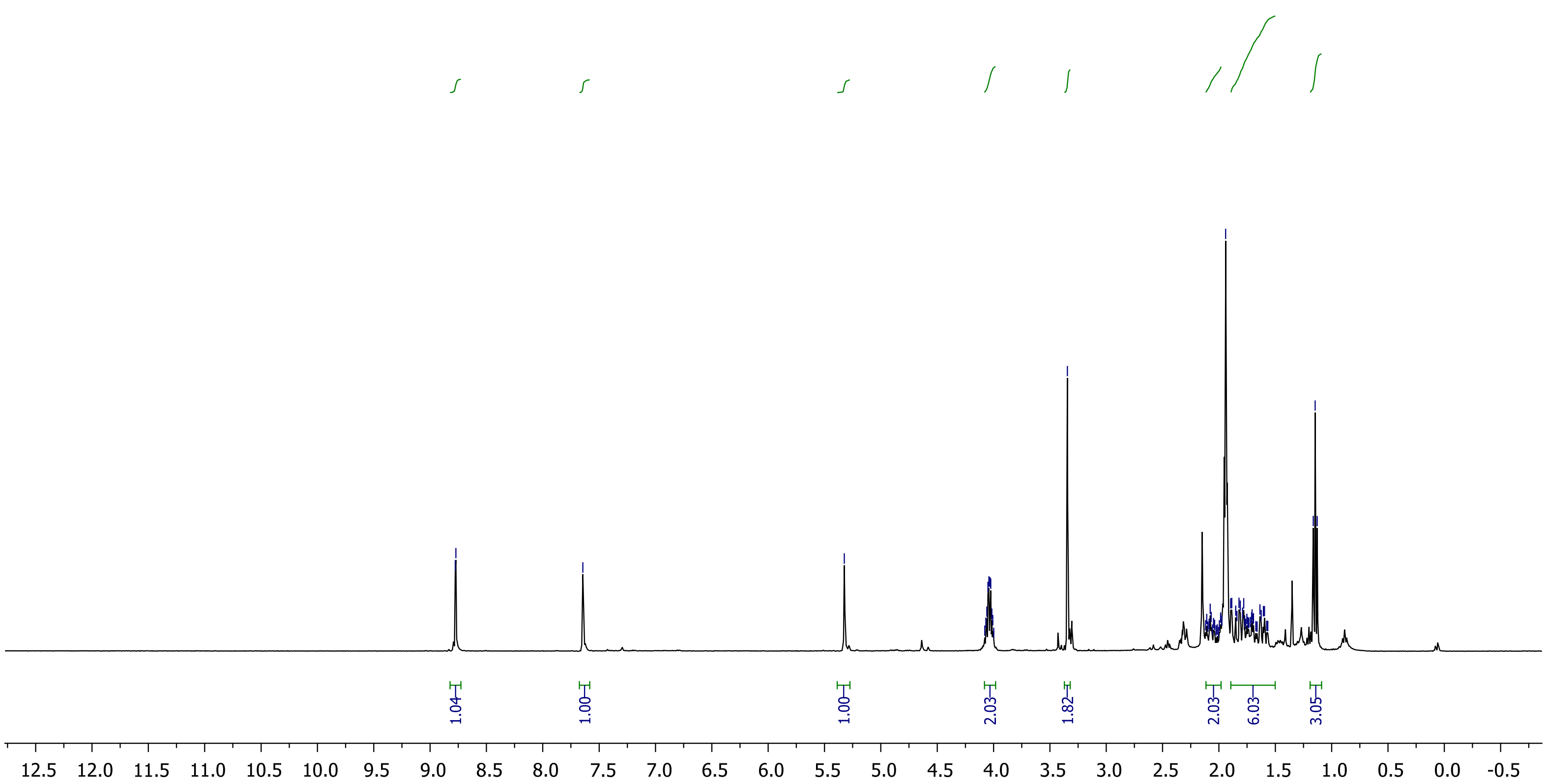

^{1}H NMR (400 MHz, CD$_3$CN)
R2692378
8.78
8.77
7.65
5.32
4.08
4.07
4.06
4.05
4.04
4.03
4.02
4.01
4.01
4.00
3.34
2.08
1.94
1.90
1.89
1.85
1.82
1.81
1.78
1.64
1.61
1.60
1.16
1.15
1.13
1.04
1.00
1.00
2.03
1.82
2.03
6.03
3.05
12.5 12.0 11.5 11.0 10.5 10.0 9.5 9.0 8.5 8.0 7.5 7.0 6.5 6.0 5.5 5.0 4.5 4.0 3.5 3.0 2.5 2.0 1.5 1.0 0.5 0.0 -0.5

$^{13}C\{^1H\}$ NMR (126 MHz, CD_3CN)

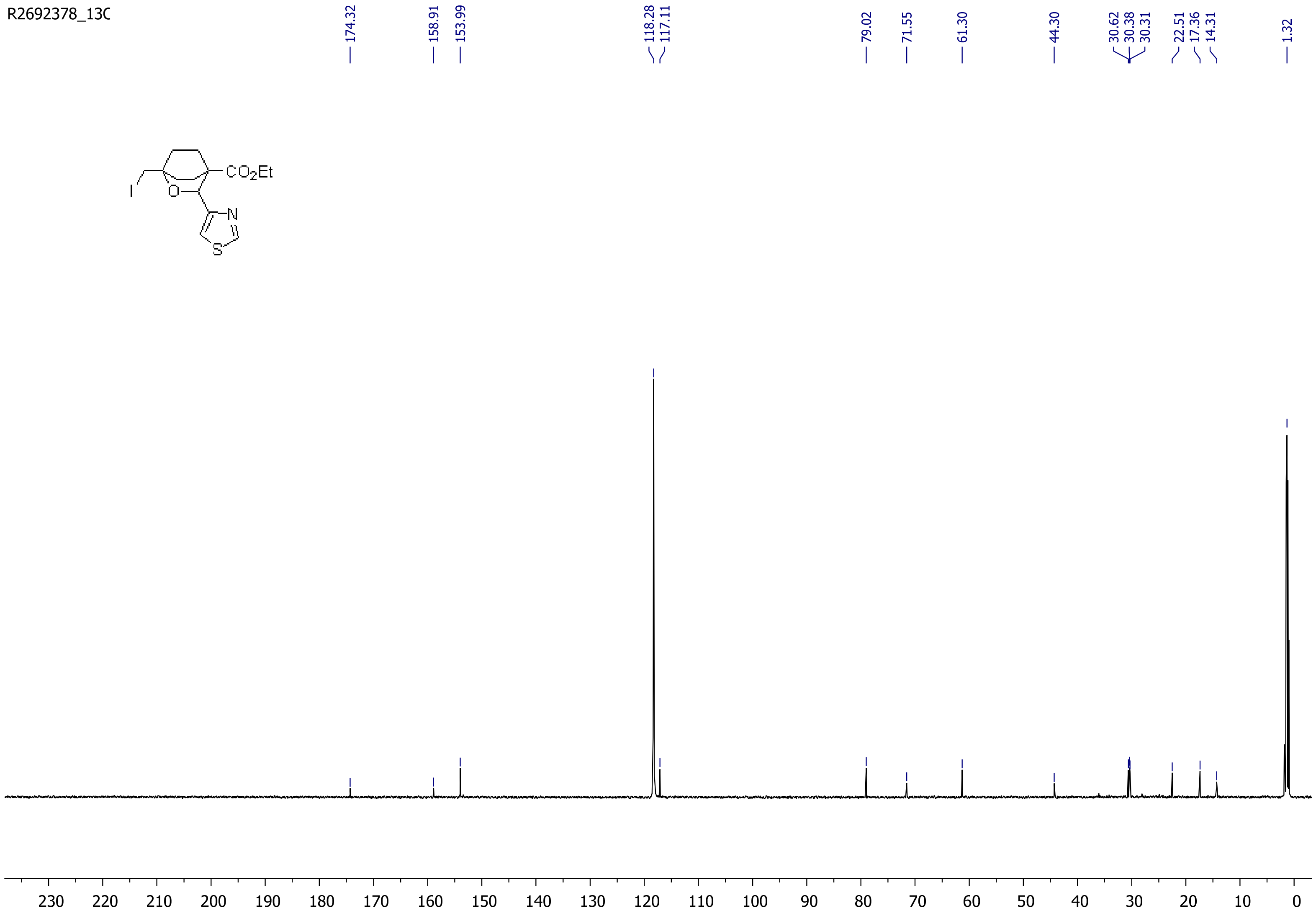

Compound 23

^{1}H NMR (400 MHz, DMSO-d_6)

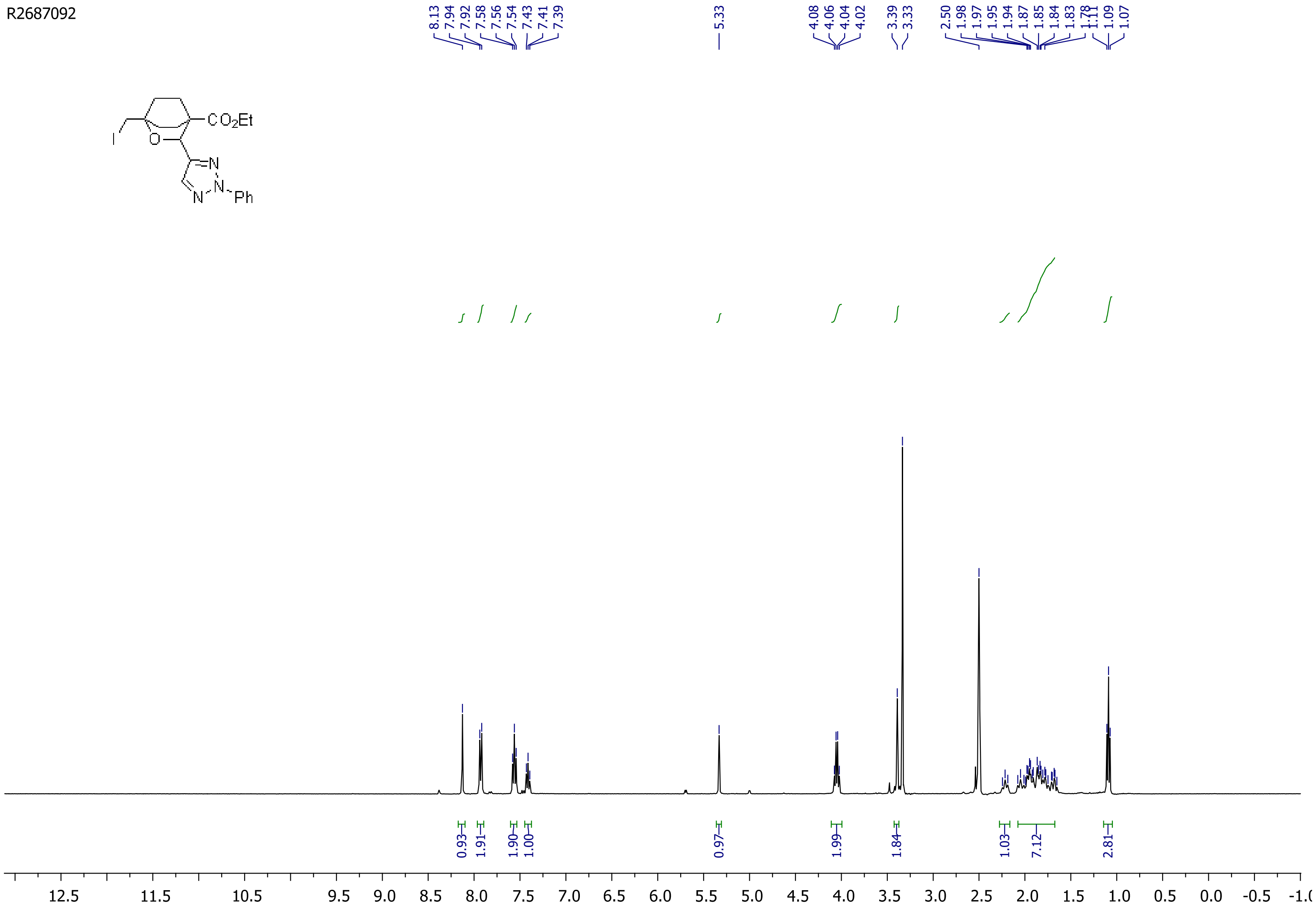

$^{13}C\{^{1}H\}$ NMR (126 MHz, DMSO-d_6)

R2687092_13C

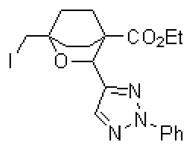

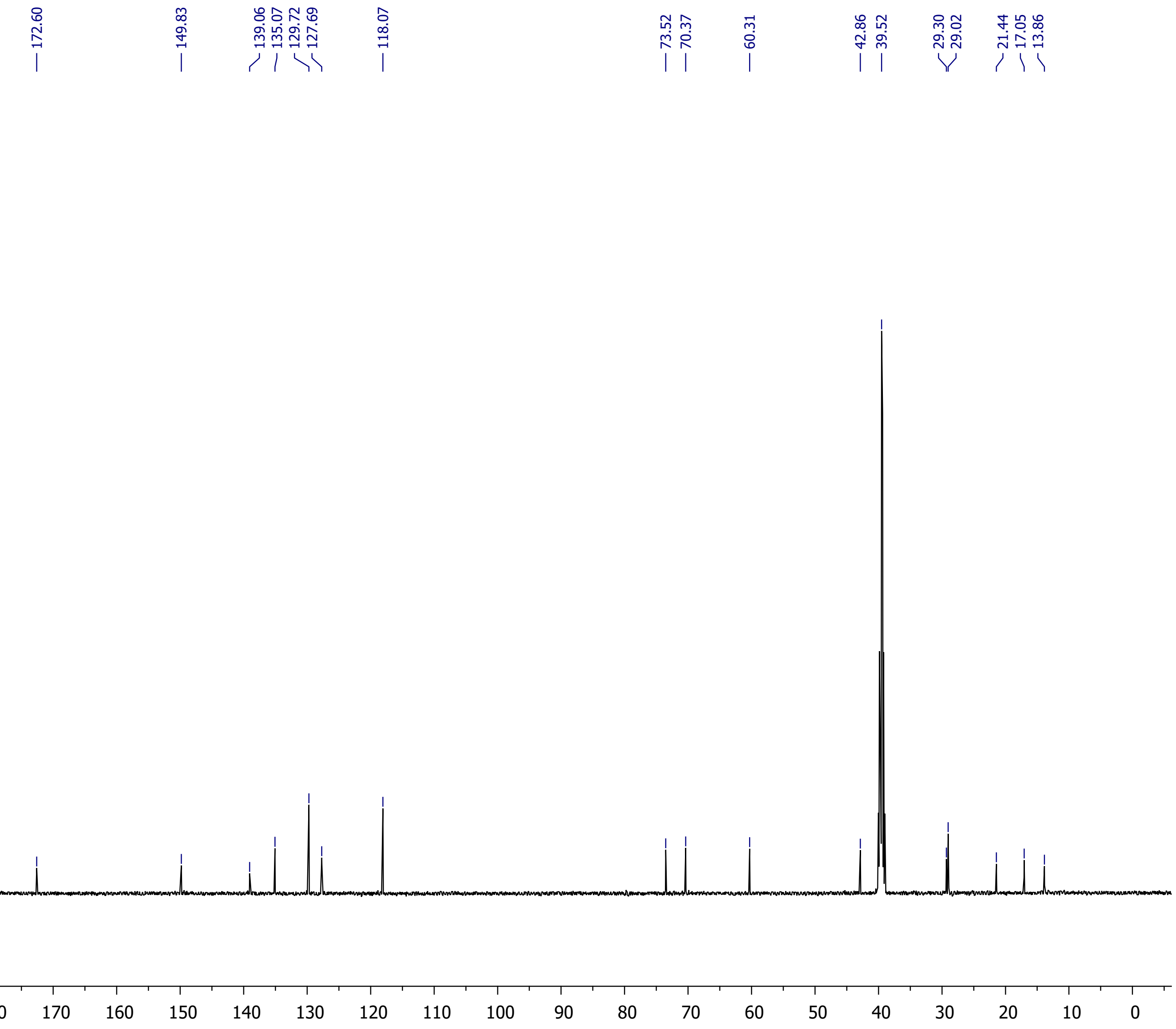

Compound 24

^{1}H NMR (400 MHz, $CDCl_3$)

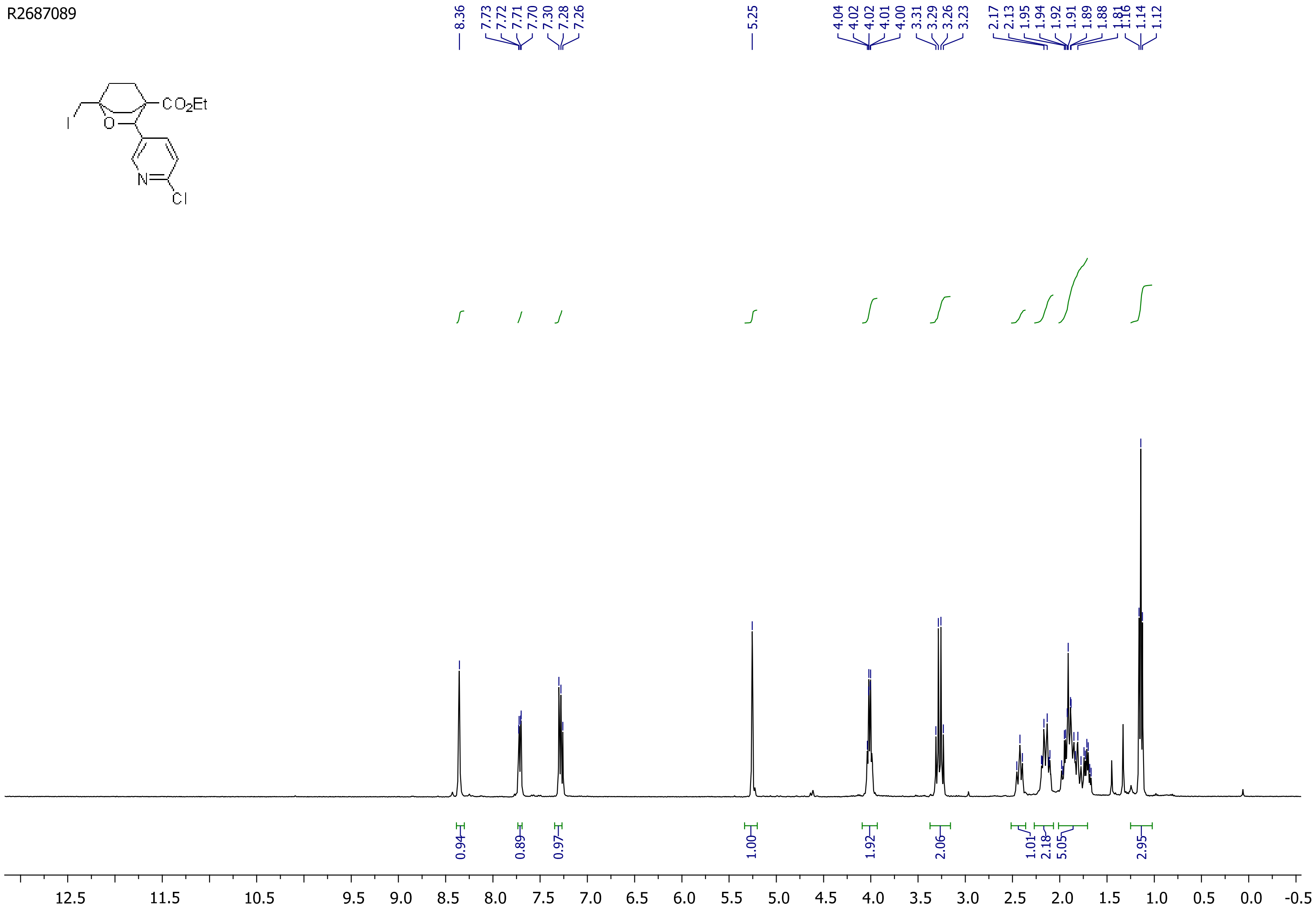

$^{13}C\{^{1}H\}$ NMR (126 MHz, $CDCl_3$)

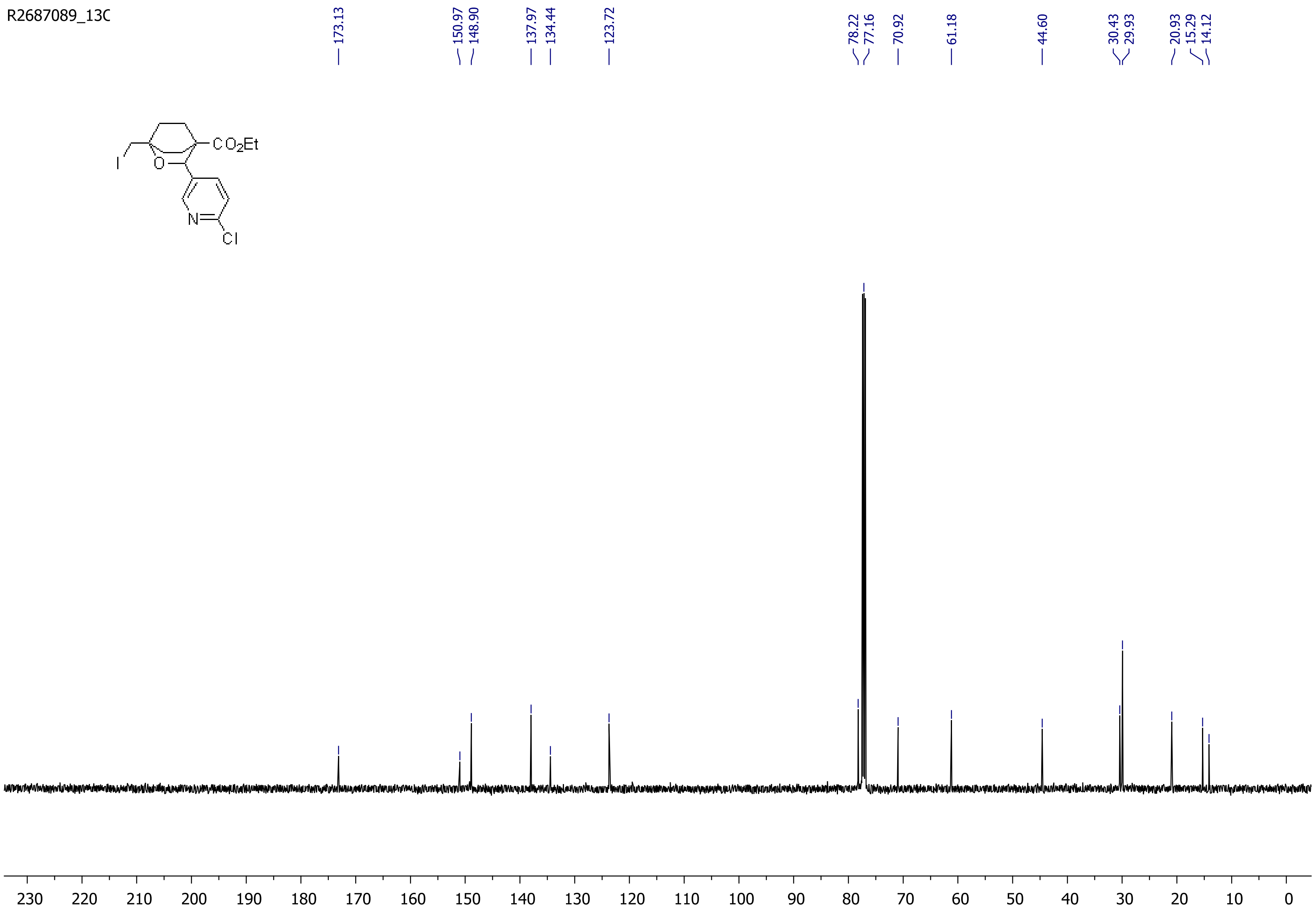

Compound 25

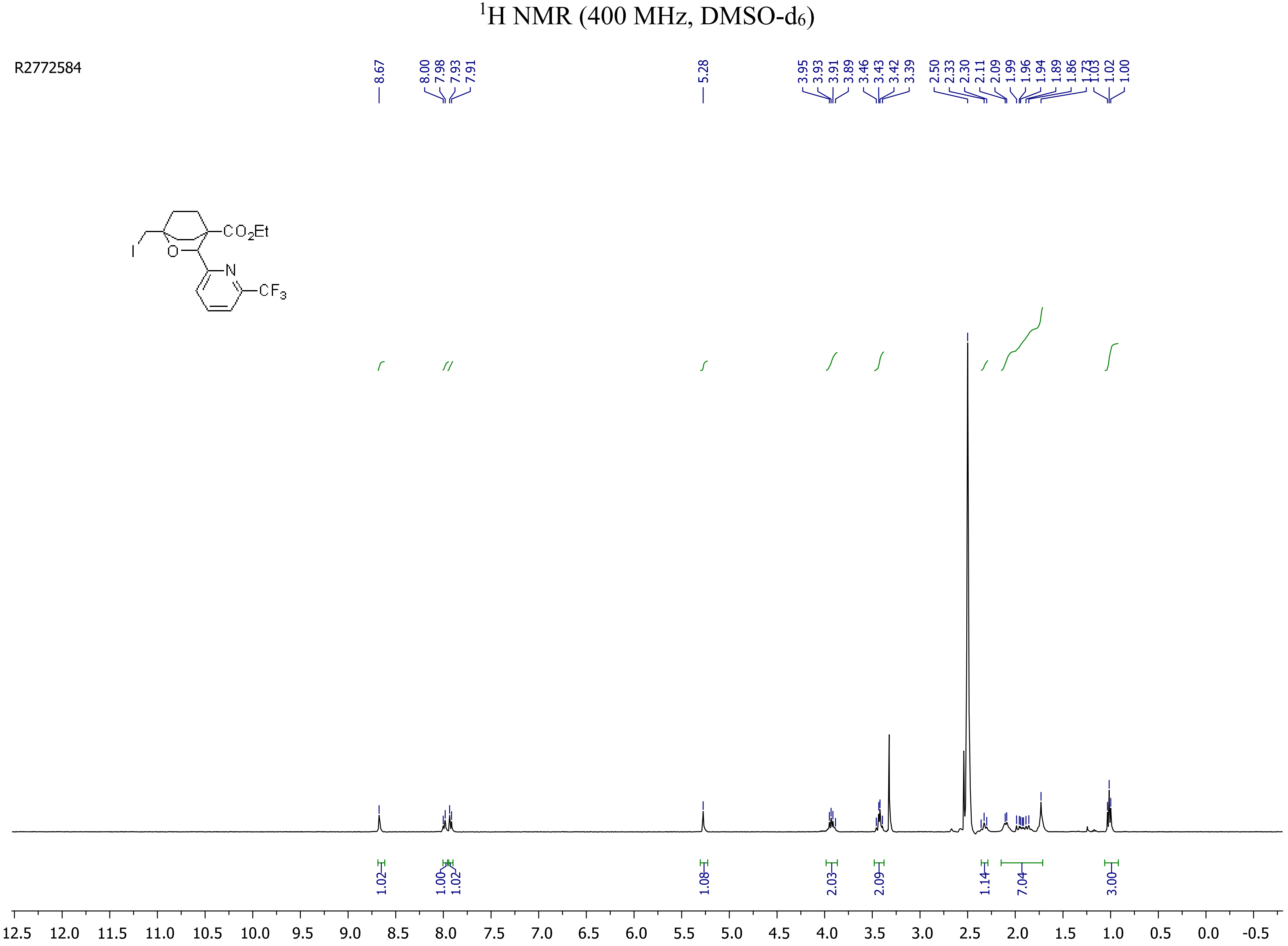

^{1}H NMR (400 MHz, DMSO-d6)
R2772584
8.67
8.00
7.98
7.93
7.91
5.28
3.95
3.93
3.91
3.89
3.46
3.43
3.42
3.39
2.50
2.33
2.30
2.11
2.09
1.99
1.96
1.94
1.89
1.86
1.73
1.03
1.02
1.00
CO2Et
I
O
N
CF3
1.02
1.00
1.02
1.08
2.03
2.09
1.14
7.04
3.00
12.5
12.0
11.5
11.0
10.5
10.0
9.5
9.0
8.5
8.0
7.5
7.0
6.5
6.0
5.5
5.0
4.5
4.0
3.5
3.0
2.5
2.0
1.5
1.0
0.5
0.0
-0.5

$^{13}C\{^1H\}$ NMR (151 MHz, DMSO-d6)

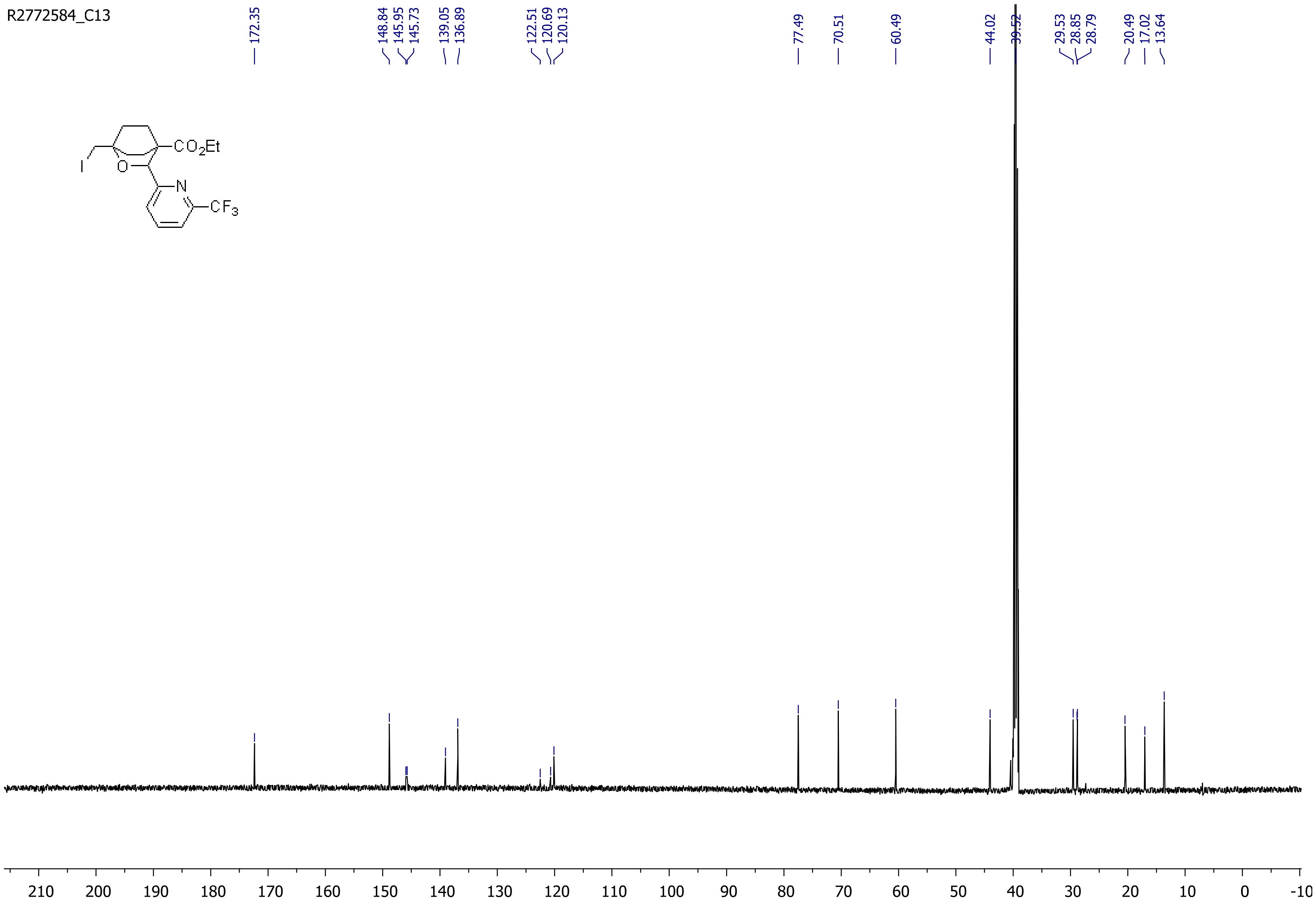

$^{19}F\{^1H\}$ NMR (376 MHz, DMSO-d_6)

R2772584_F19
19F-{1H}

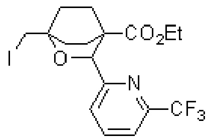

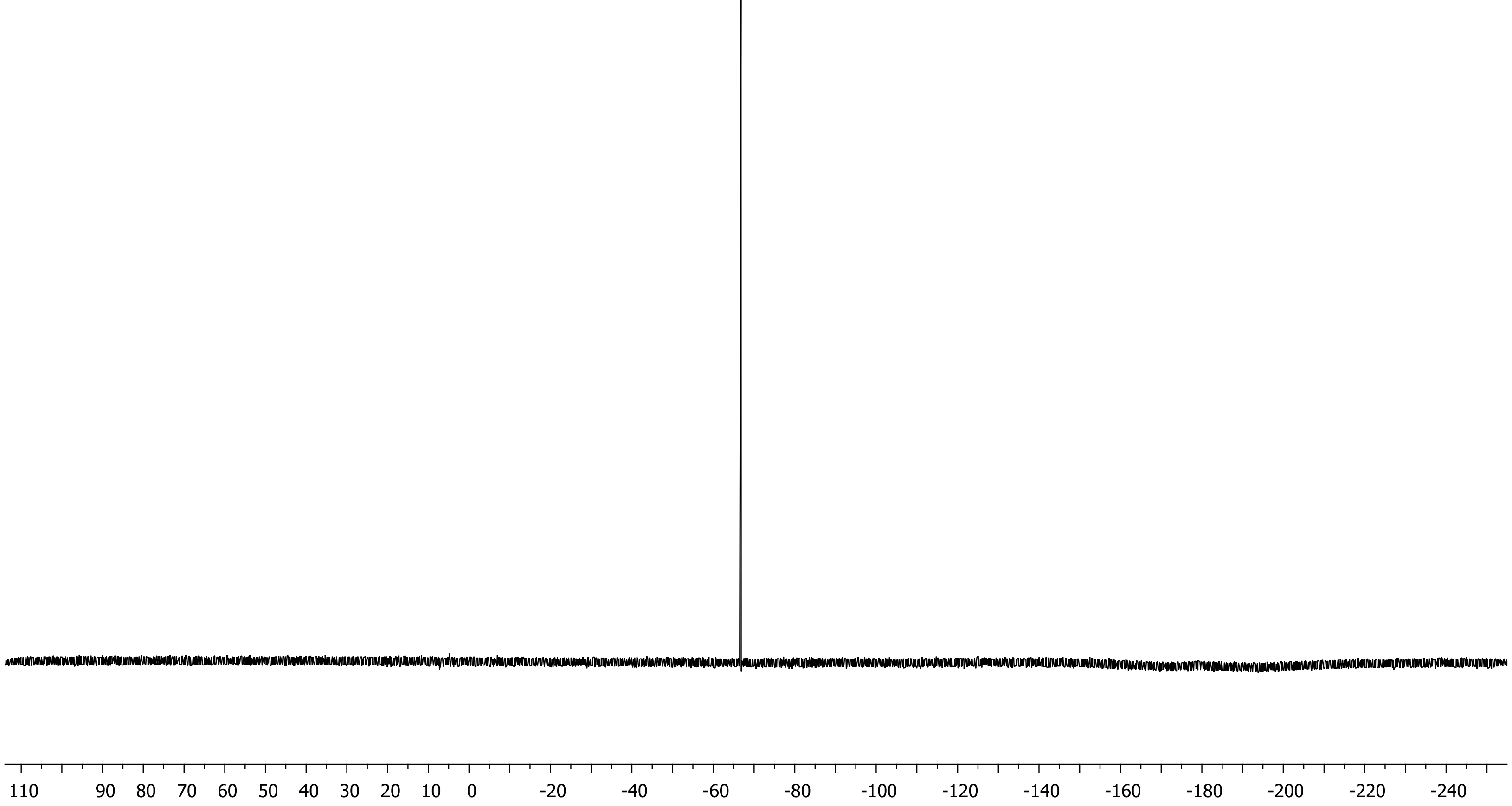

Compound 26

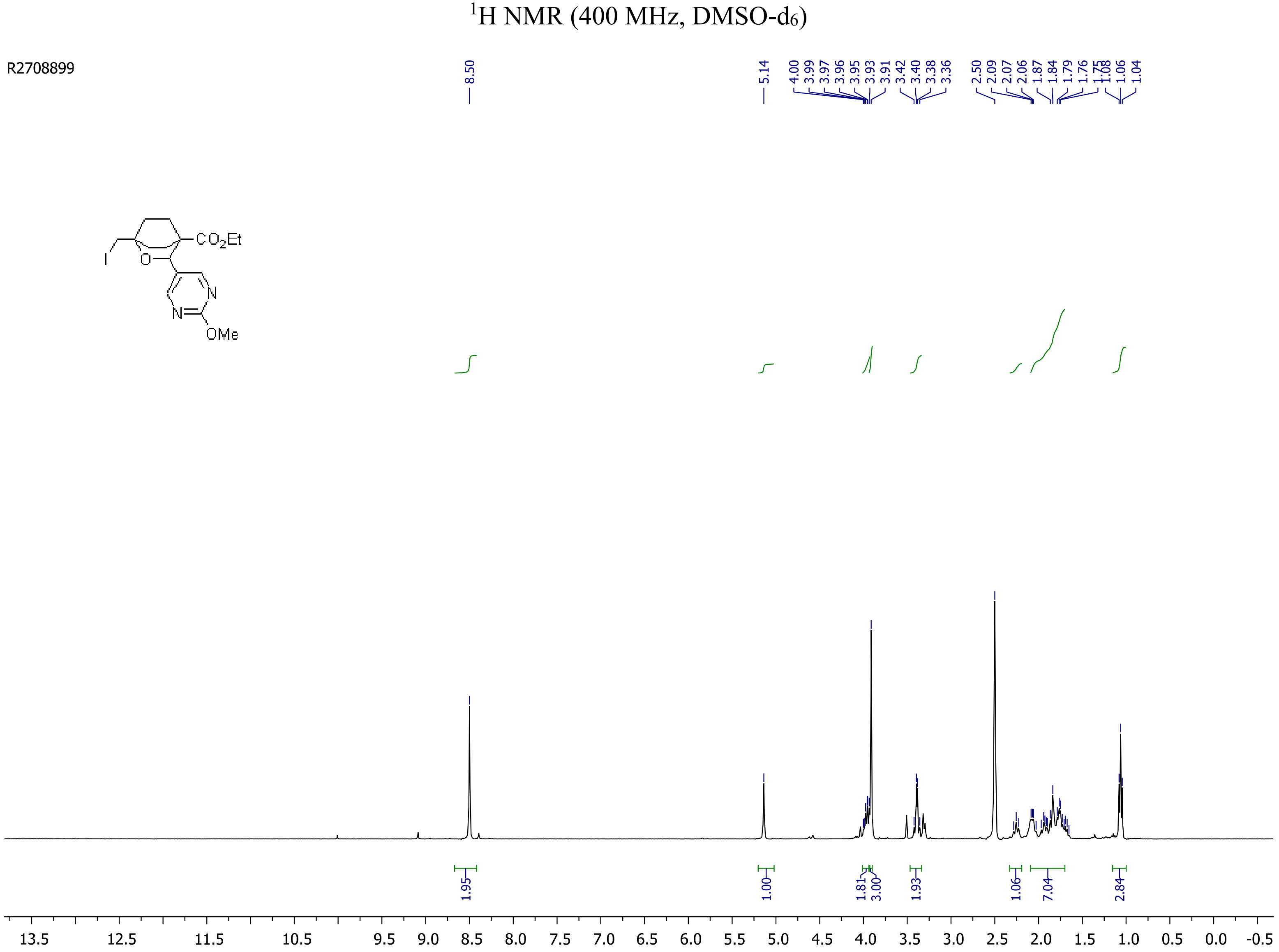

^{1}H NMR (400 MHz, DMSO-d6)
R2708899
CO2Et
I
O
N
N
OMe
8.50
5.14
4.00 3.99 3.97 3.96 3.95 3.93 3.91 3.42 3.40 3.38 3.36
2.50 2.09 2.07 2.06 1.87 1.84 1.79 1.76 1.75 1.08 1.06 1.04
1.95
1.00
1.81
3.00
1.93
1.06
7.04
2.84
13.5 12.5 11.5 10.5 9.5 9.0 8.5 8.0 7.5 7.0 6.5 6.0 5.5 5.0 4.5 4.0 3.5 3.0 2.5 2.0 1.5 1.0 0.5 0.0 -0.5

$^{13}C\{^{1}H\}$ NMR (126 MHz, DMSO-d6)

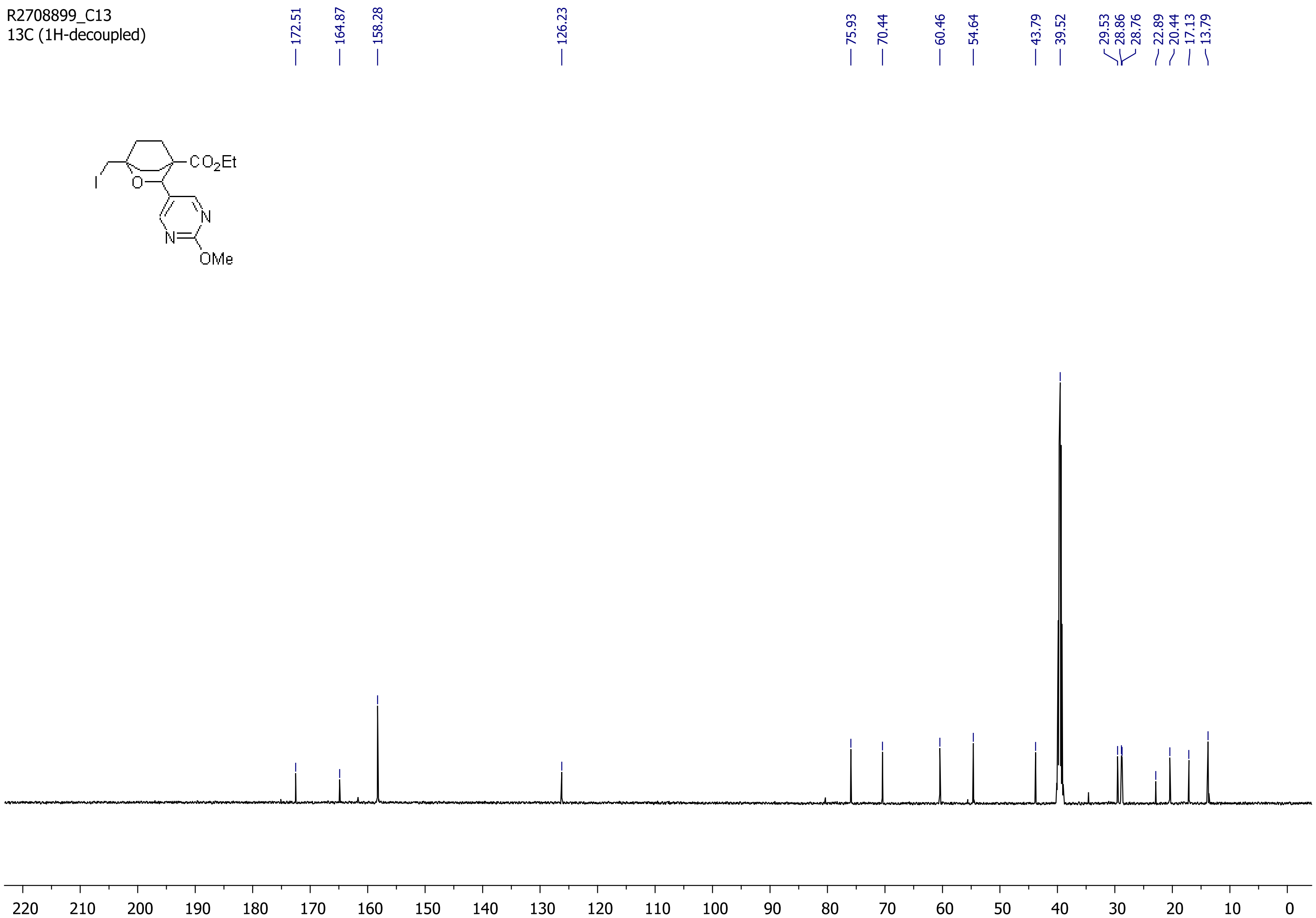

Compound 27

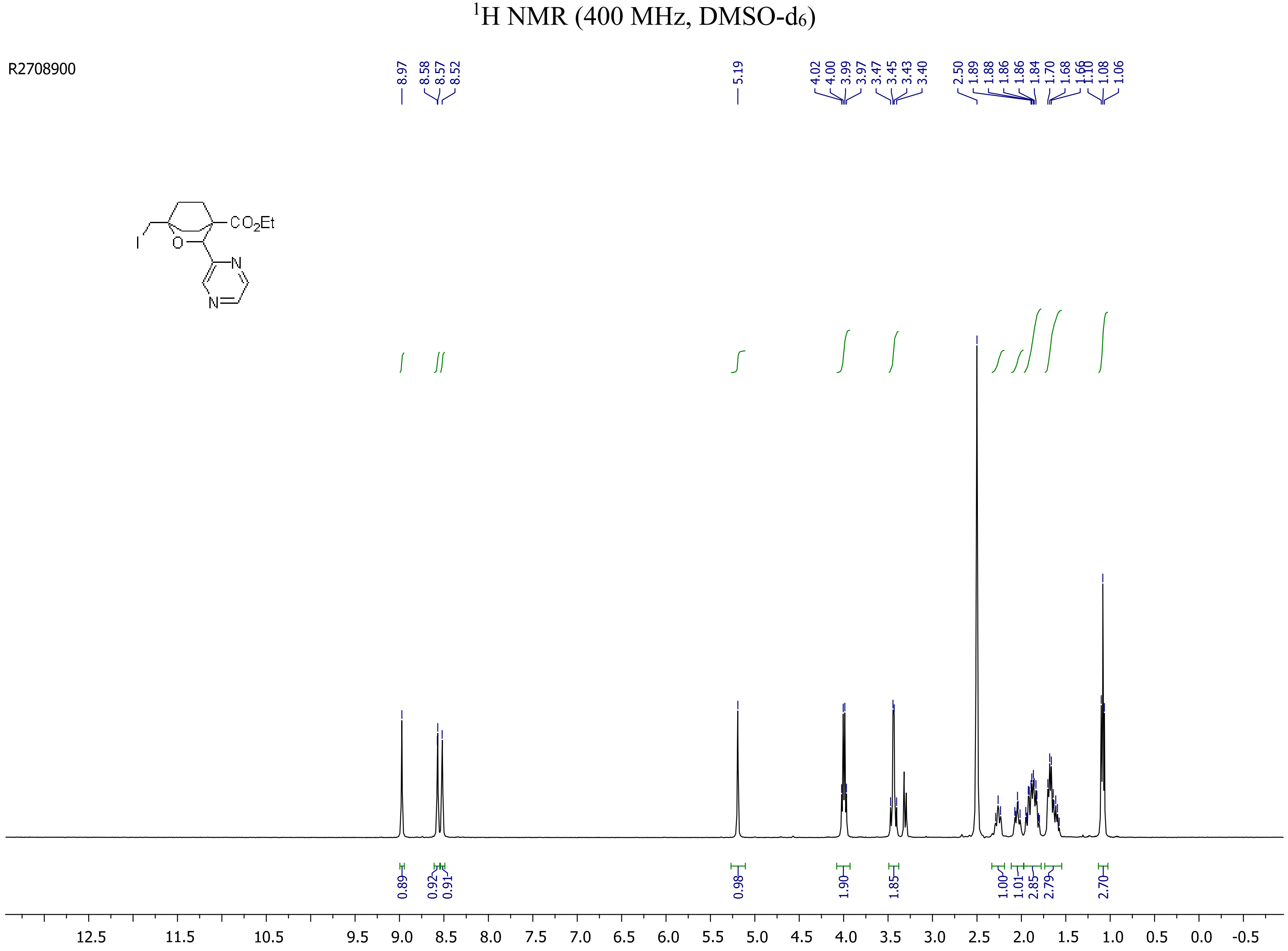

¹H NMR (400 MHz, DMSO-d₆)
R2708900
8.97
8.58
8.57
8.52
5.19
4.02
4.00
3.99
3.97
3.47
3.45
3.43
3.40
2.50
1.89
1.88
1.86
1.86
1.84
1.70
1.68
1.66
1.10
1.08
1.06
CO₂Et
I
O
N
N
0.89
0.92
0.91
0.98
1.90
1.85
1.00
1.01
2.85
2.79
2.70
12.5
11.5
10.5
9.5
9.0
8.5
8.0
7.5
7.0
6.5
6.0
5.5
5.0
4.5
4.0
3.5
3.0
2.5
2.0
1.5
1.0
0.5
0.0
-0.5

$^{13}C\{^1H\}$ NMR (126 MHz, DMSO-d6)

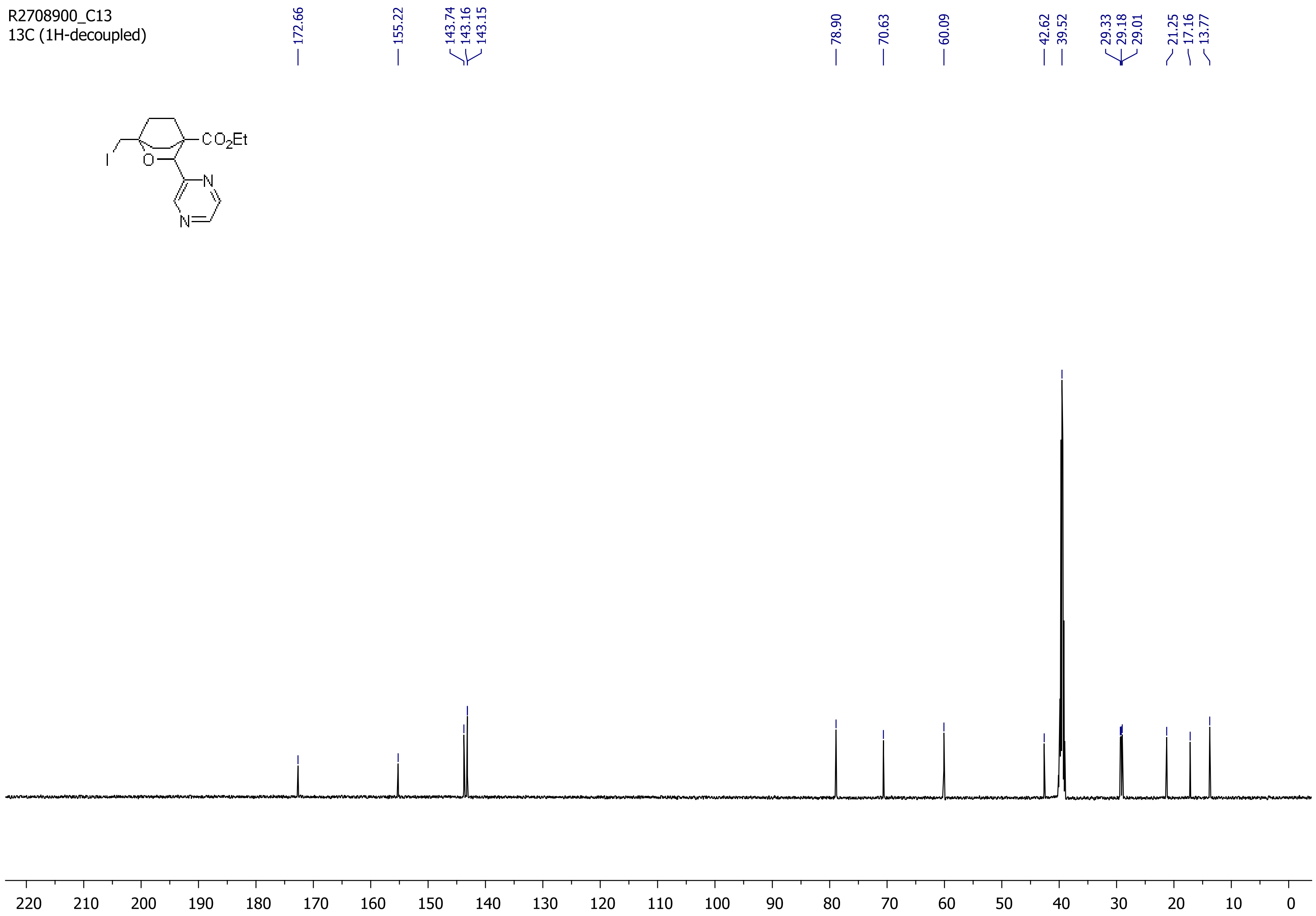

Compound SI-4

^1H NMR (500 MHz, $CDCl_3$)

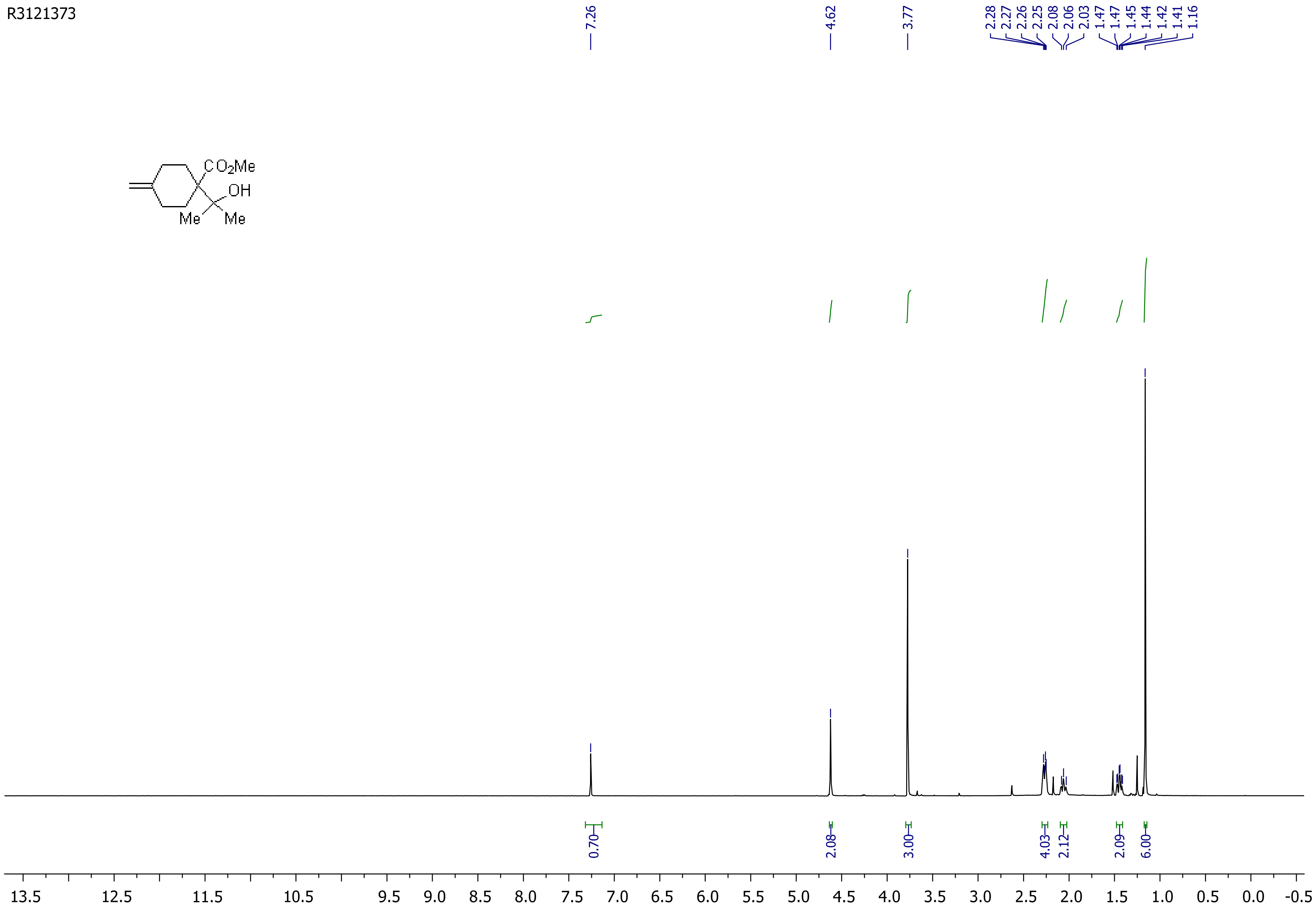

$^{13}C\{^1H\}$ NMR (126 MHz, $CDCl_3$)

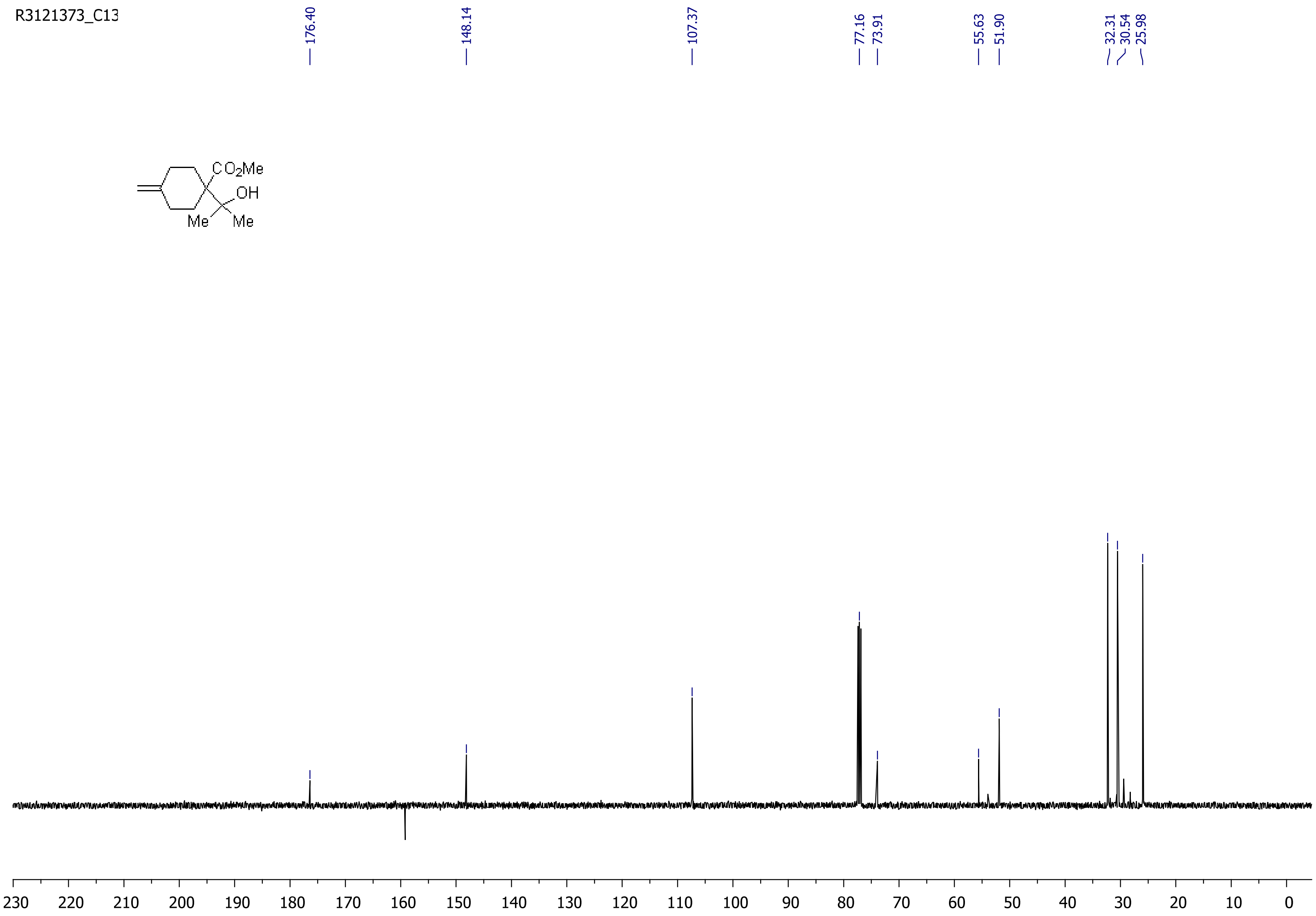

Compound 30

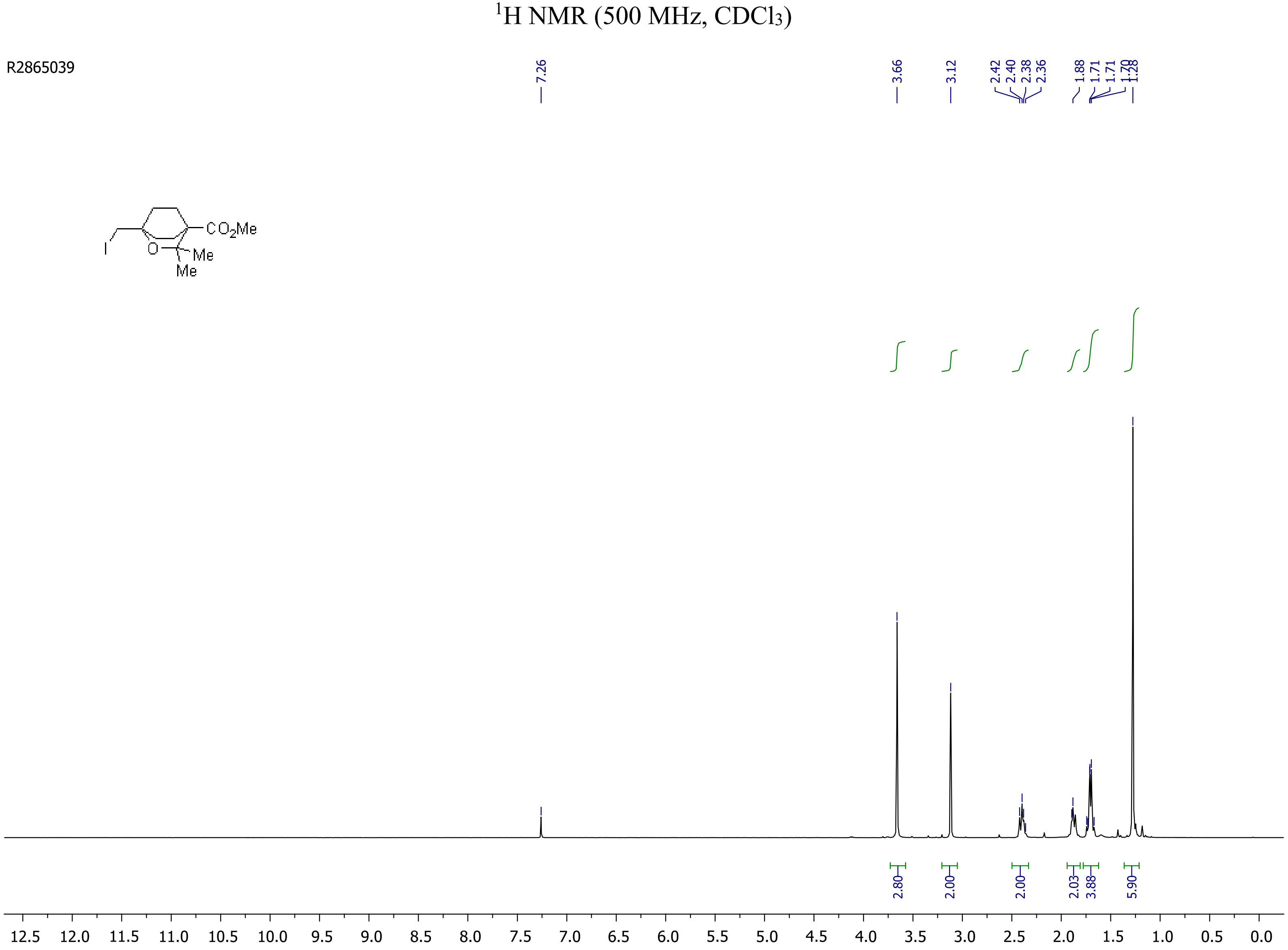

^{1}H NMR (500 MHz, CDCl$_3$)
R2865039
7.26
3.66
3.12
2.42
2.40
2.38
2.36
1.88
1.71
1.71
1.70
1.28
CO$_2$Me
O
Me
Me
I
2.80
2.00
2.00
2.03
3.88
5.90
12.5 12.0 11.5 11.0 10.5 10.0 9.5 9.0 8.5 8.0 7.5 7.0 6.5 6.0 5.5 5.0 4.5 4.0 3.5 3.0 2.5 2.0 1.5 1.0 0.5 0.0

$^{13}C\{^{1}H\}$ NMR (151 MHz, $CDCl_3$)

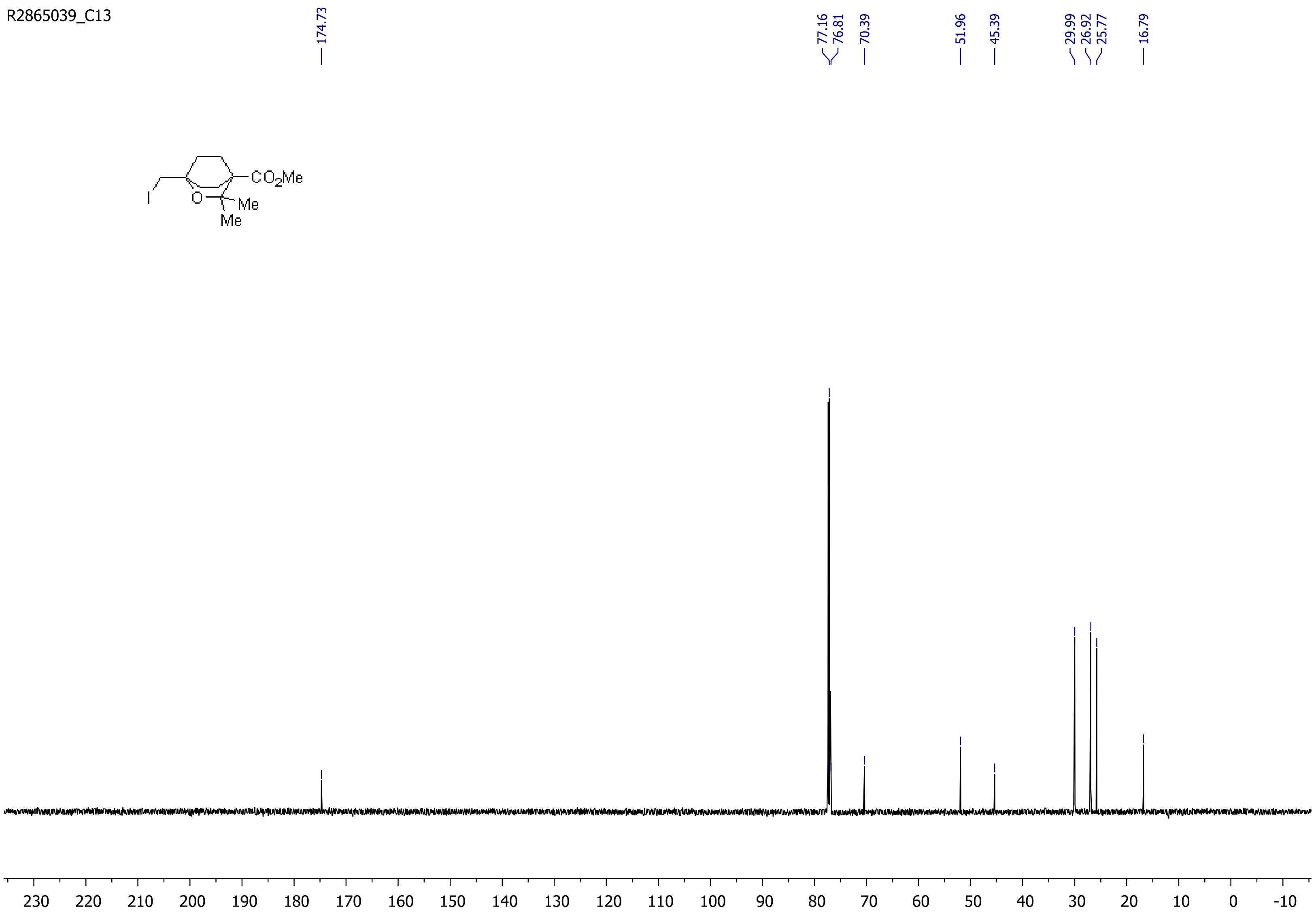

Compound SI-5

^{1}H NMR (400 MHz, $CDCl_3$)

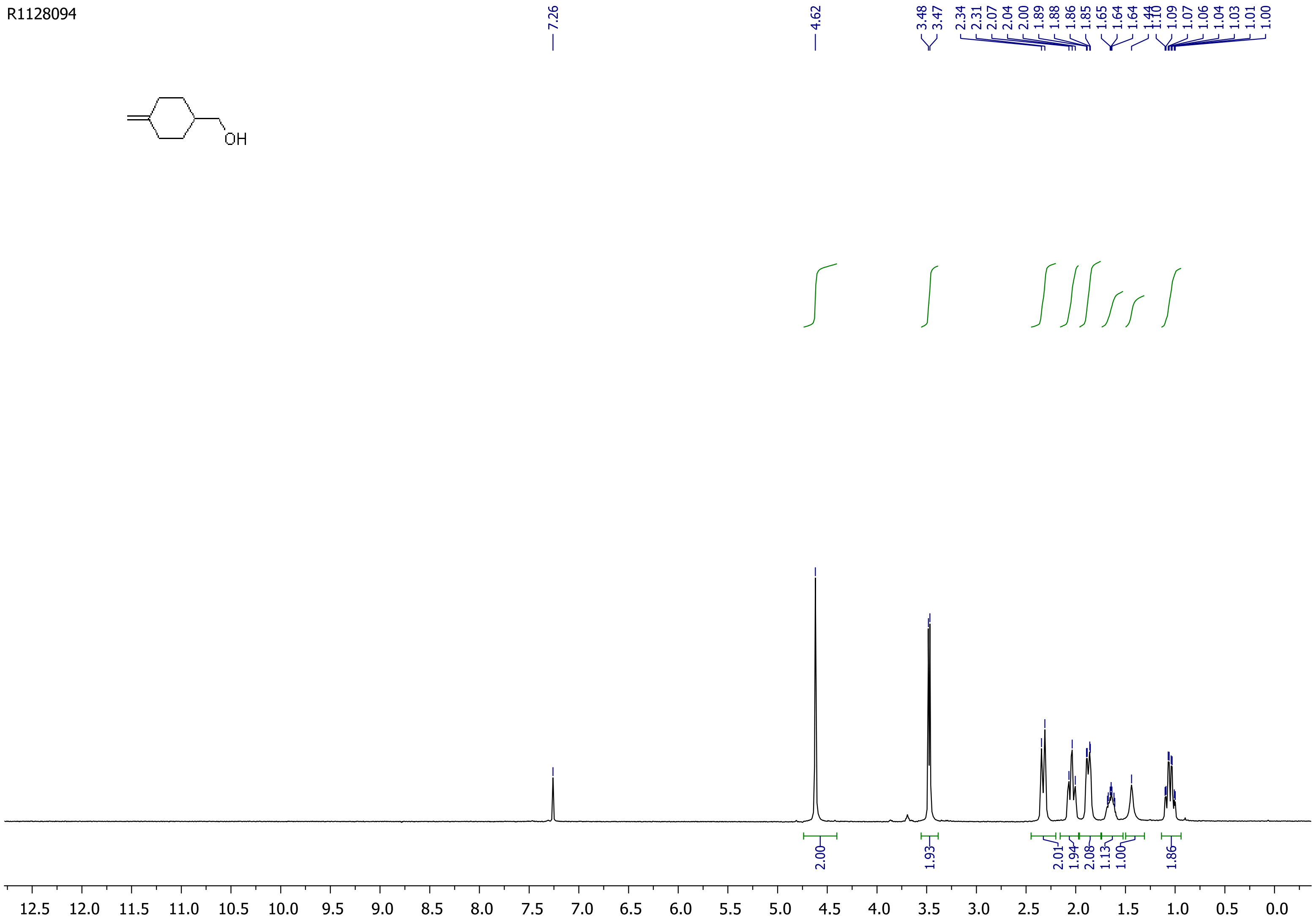

$^{13}C\{^1H\}$ NMR (126 MHz, $CDCl_3$)

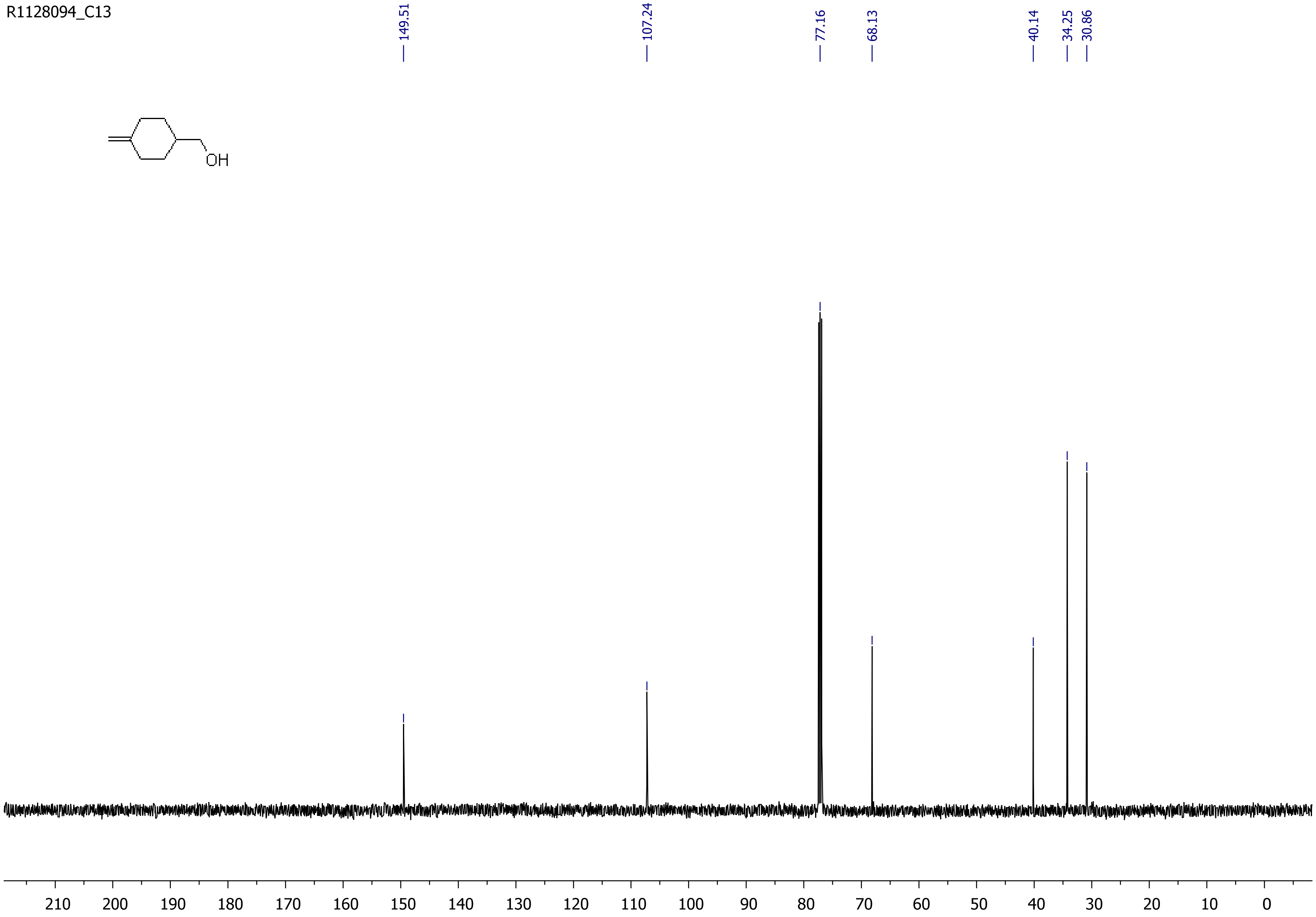

Compound 31

^{1}H NMR (400 MHz, $CDCl_3$)

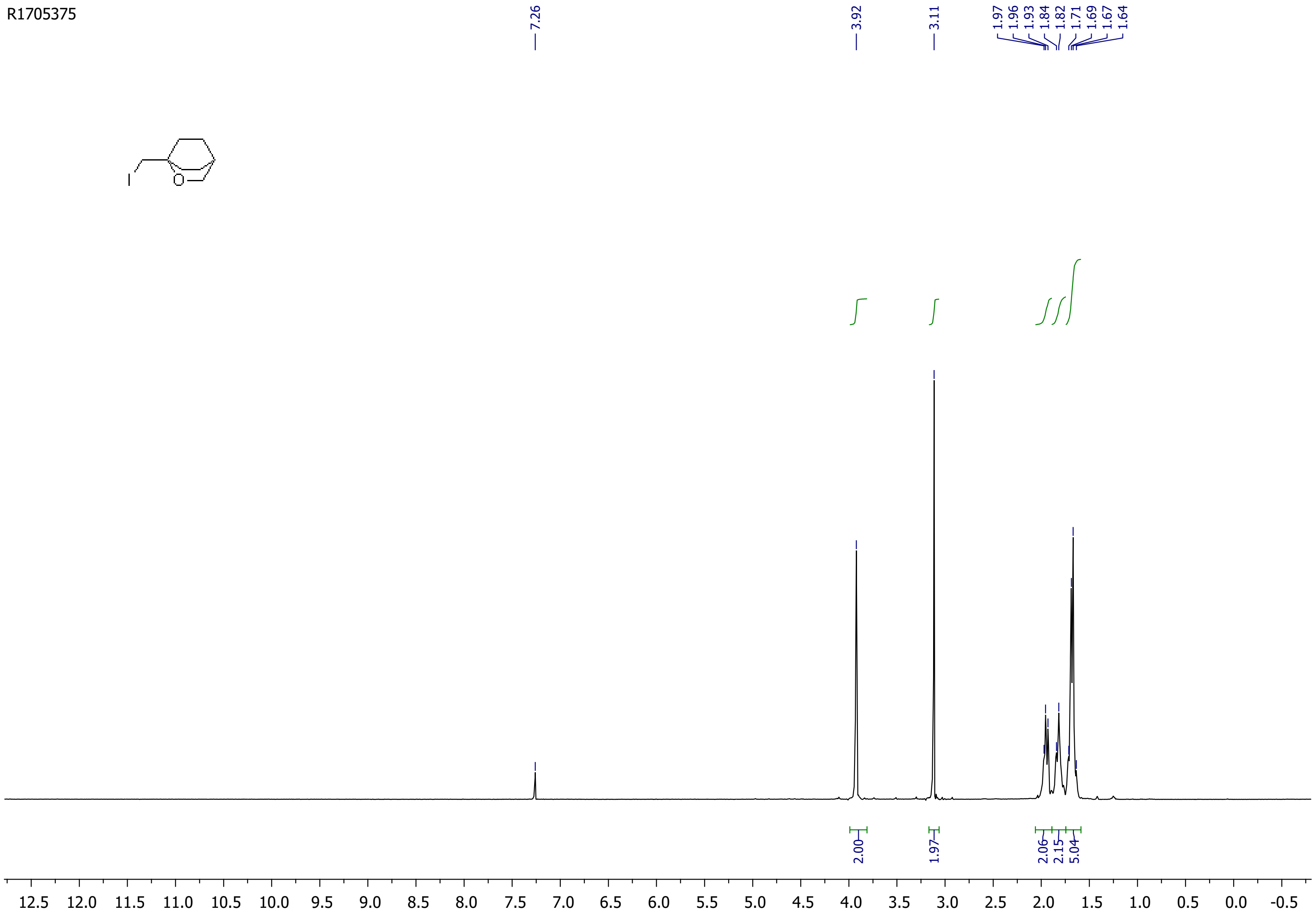

$^{13}C\{^{1}H\}$ NMR (126 MHz, $CDCl_3$)

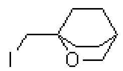

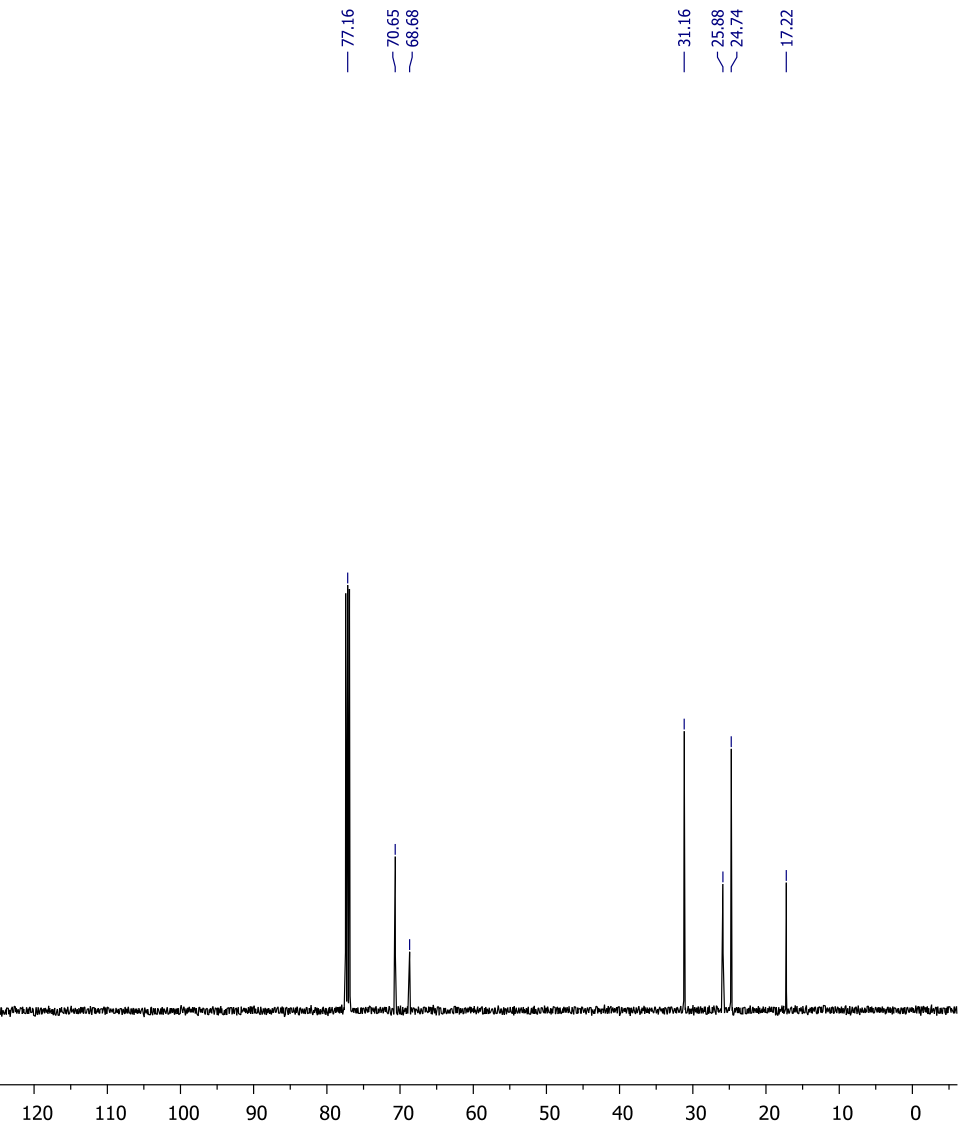

Compound SI-6

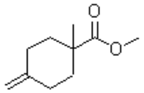

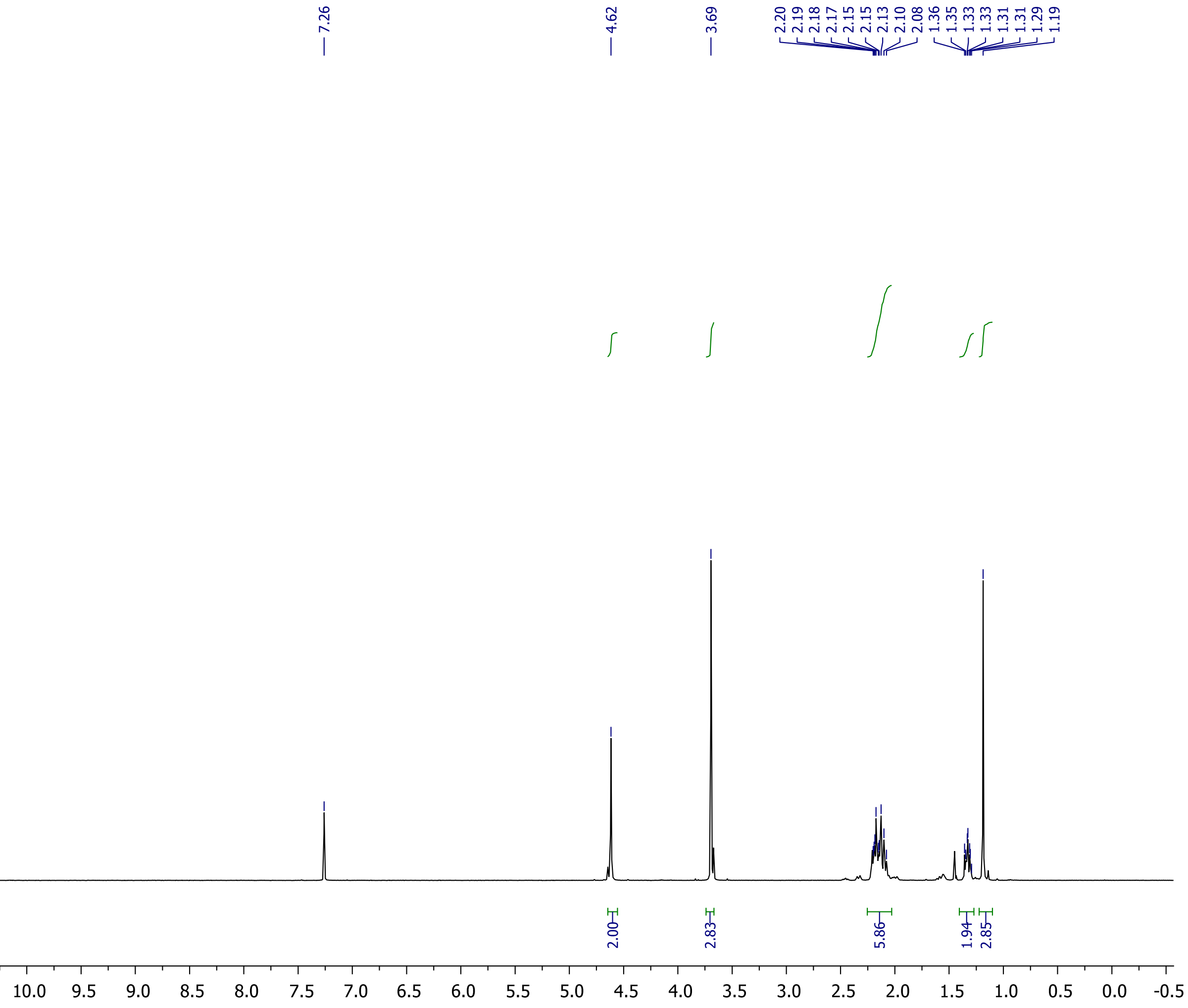

$^{13}C\{^1H\}$ NMR (126 MHz, $CDCl_3$)

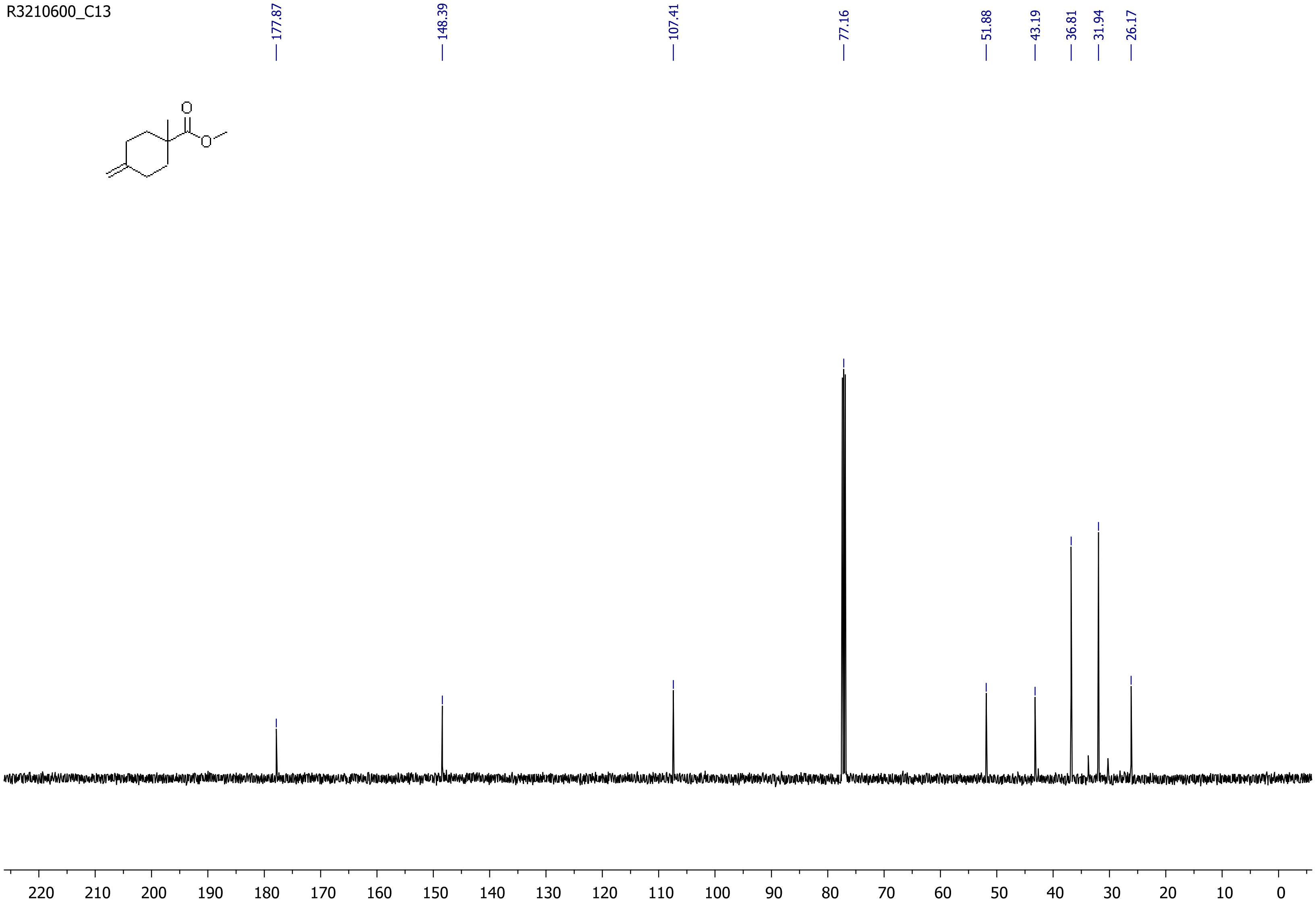

Compound SI-7

^{1}H NMR (500 MHz, $CDCl_3$)

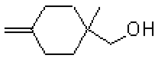

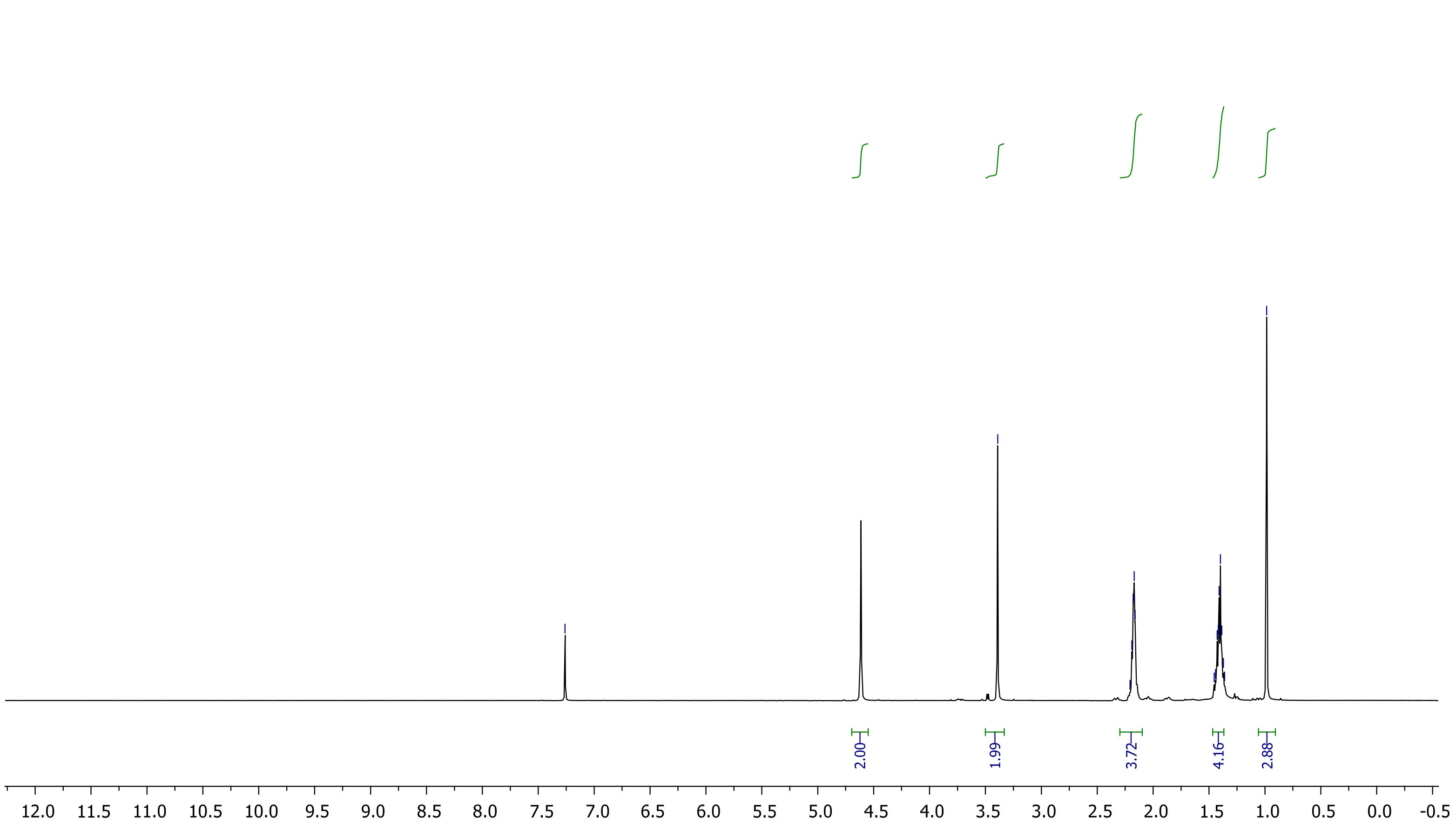

$^{13}C\{^{1}H\}$ NMR (126 MHz, $CDCl_3$)

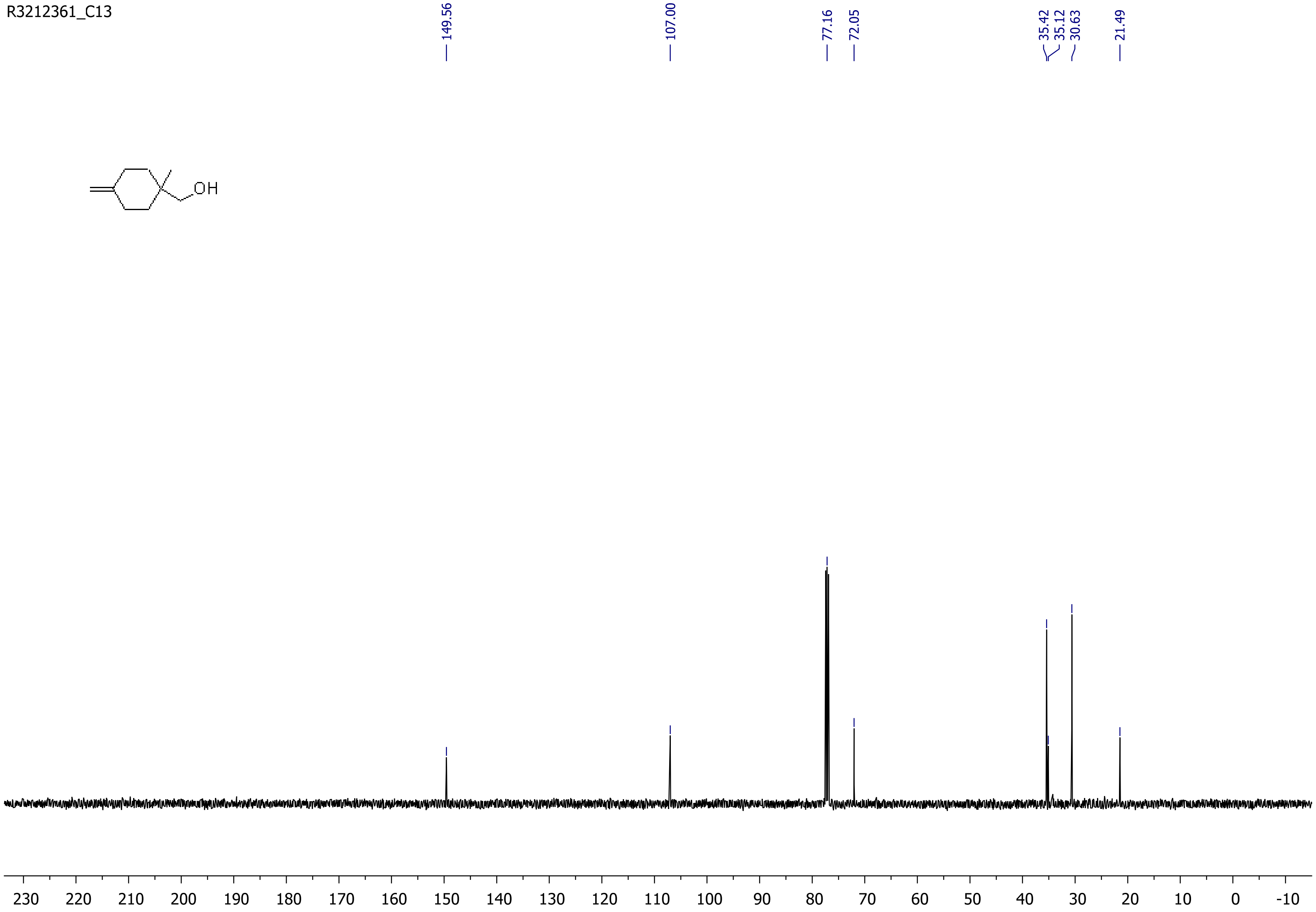

Compound 32

^{1}H NMR (400 MHz, $CDCl_3$)

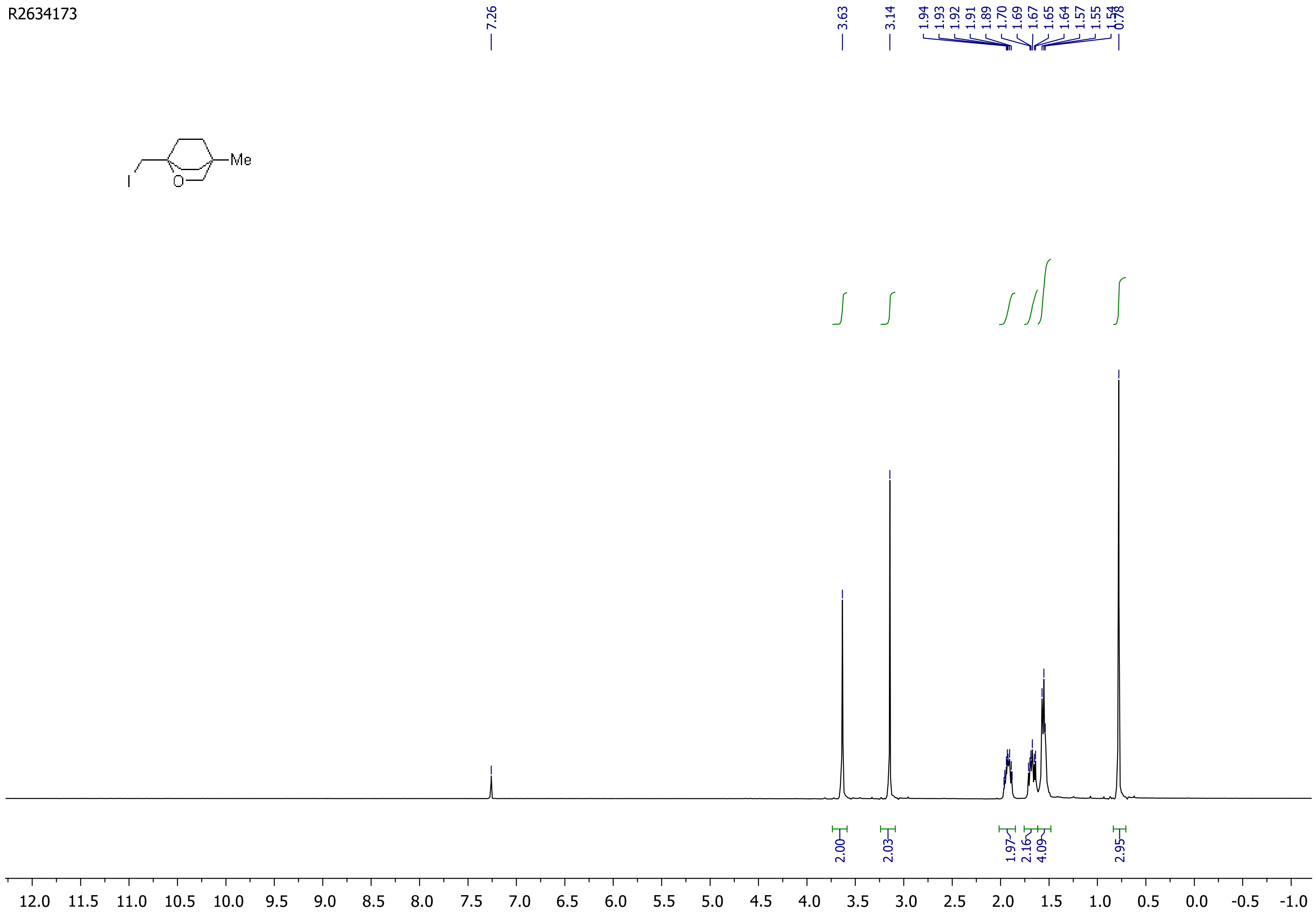

$^{13}C\{^{1}H\}$ NMR (126 MHz, $CDCl_3$)

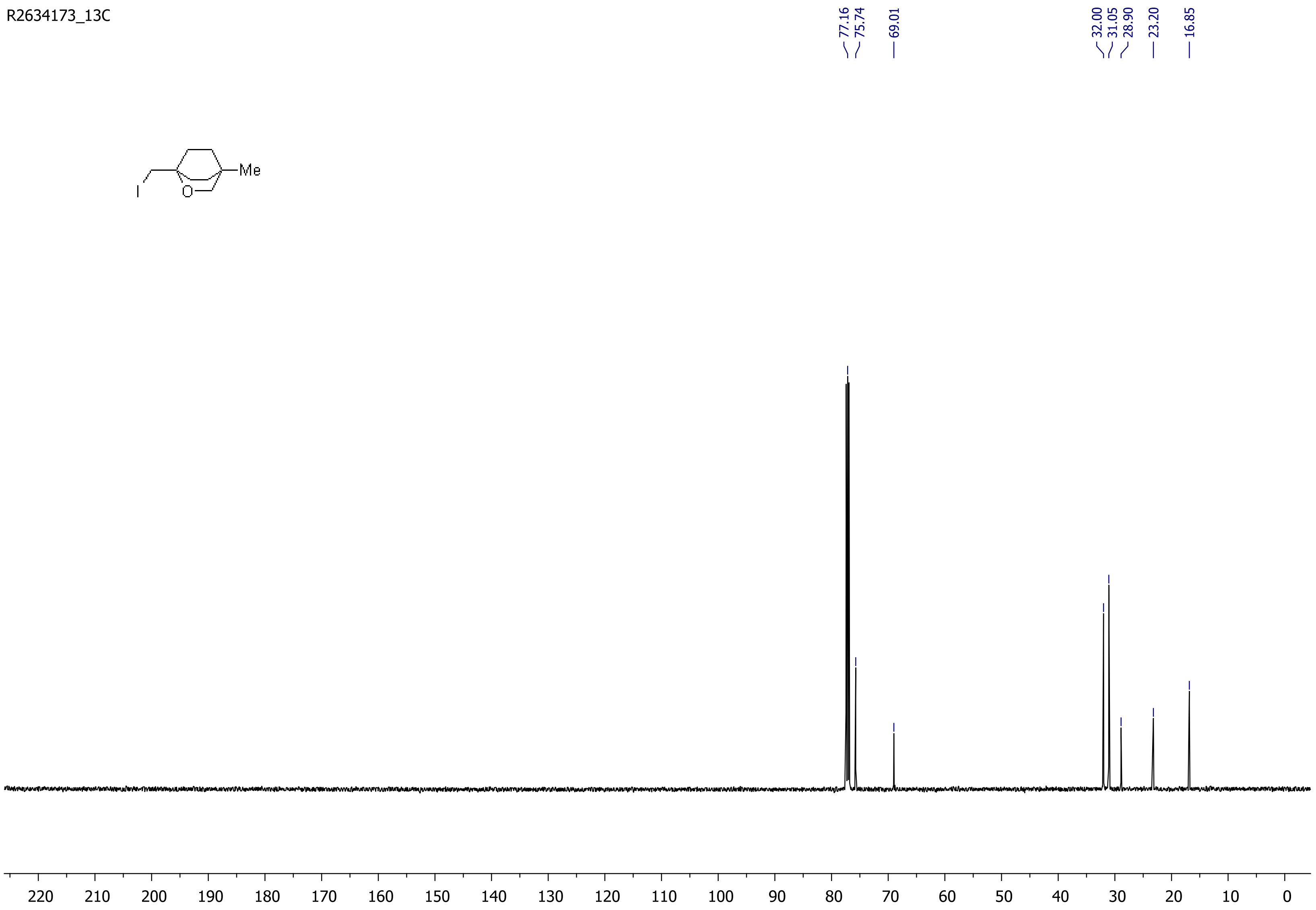

Compound SI-8

^{1}H NMR (500 MHz, $CDCl_3$)

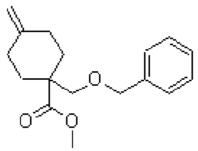

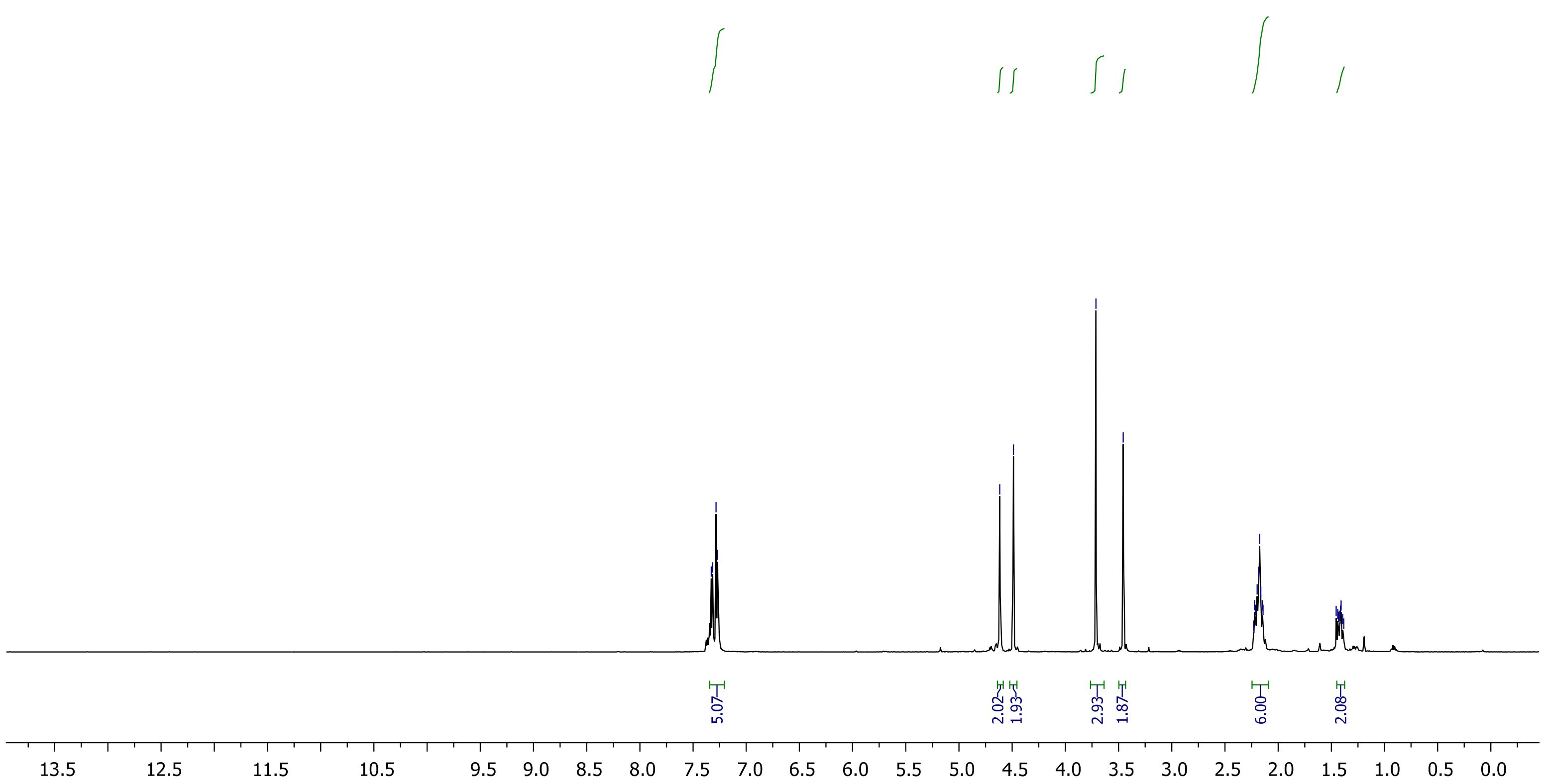

$^{13}C\{^{1}H\}$ NMR (151 MHz, $CDCl_3$)

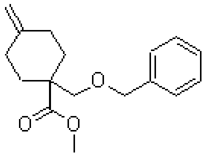

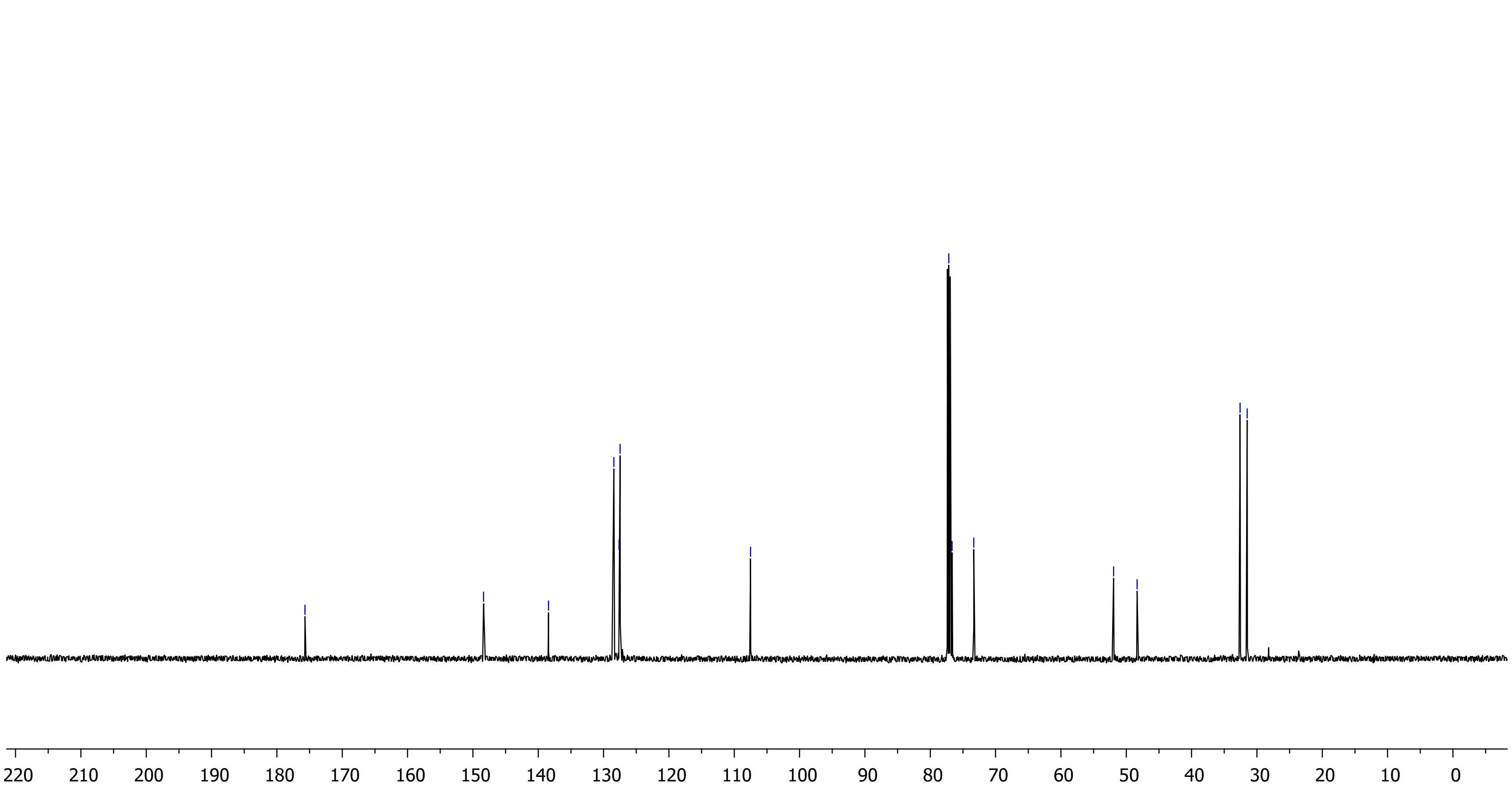

Compound SI-9

^{1}H NMR (500 MHz, $CDCl_3$)

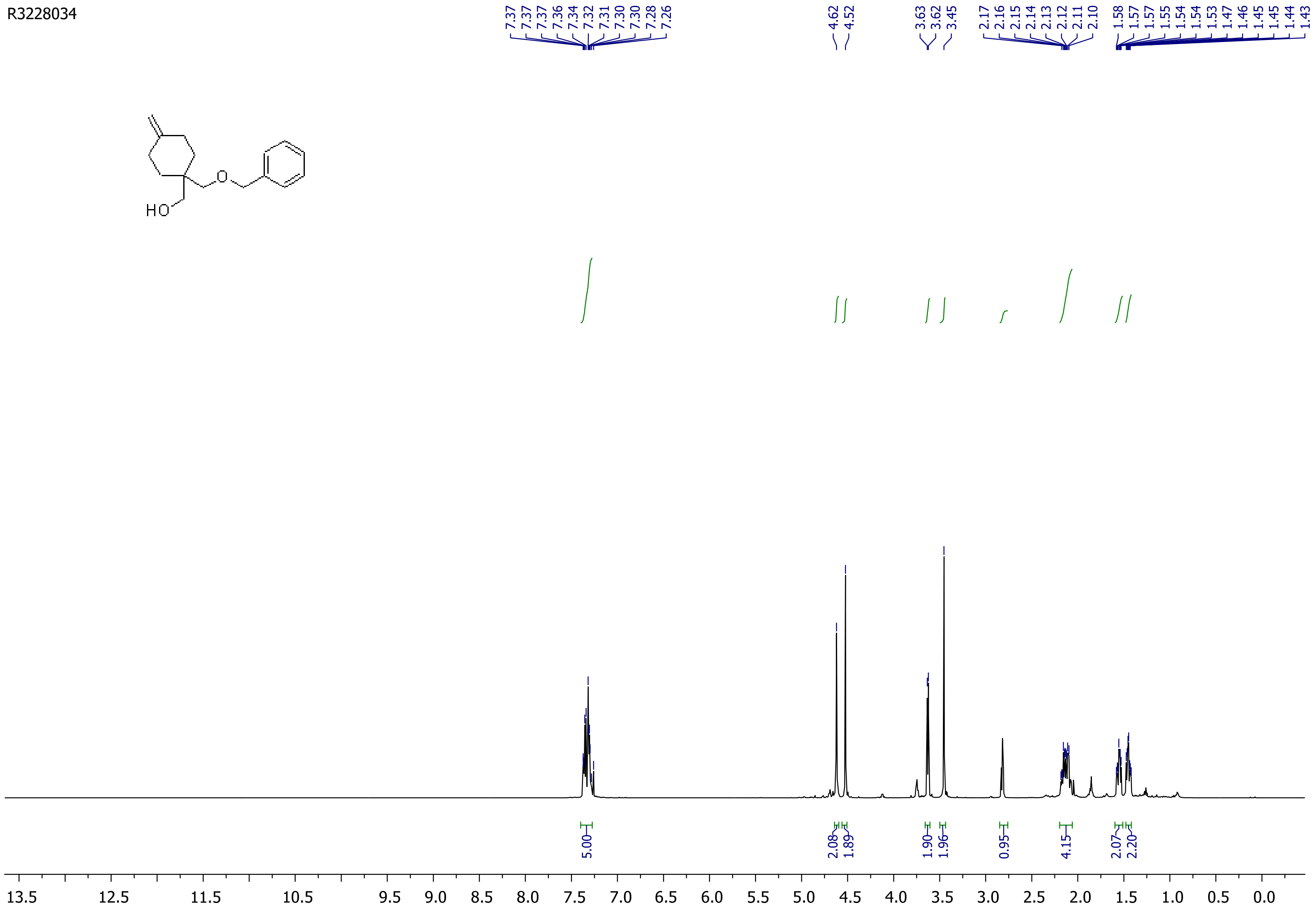

$^{13}C\{^{1}H\}$ NMR (151 MHz, $CDCl_3$)

R3228034_C13

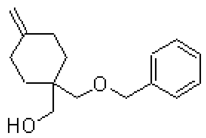

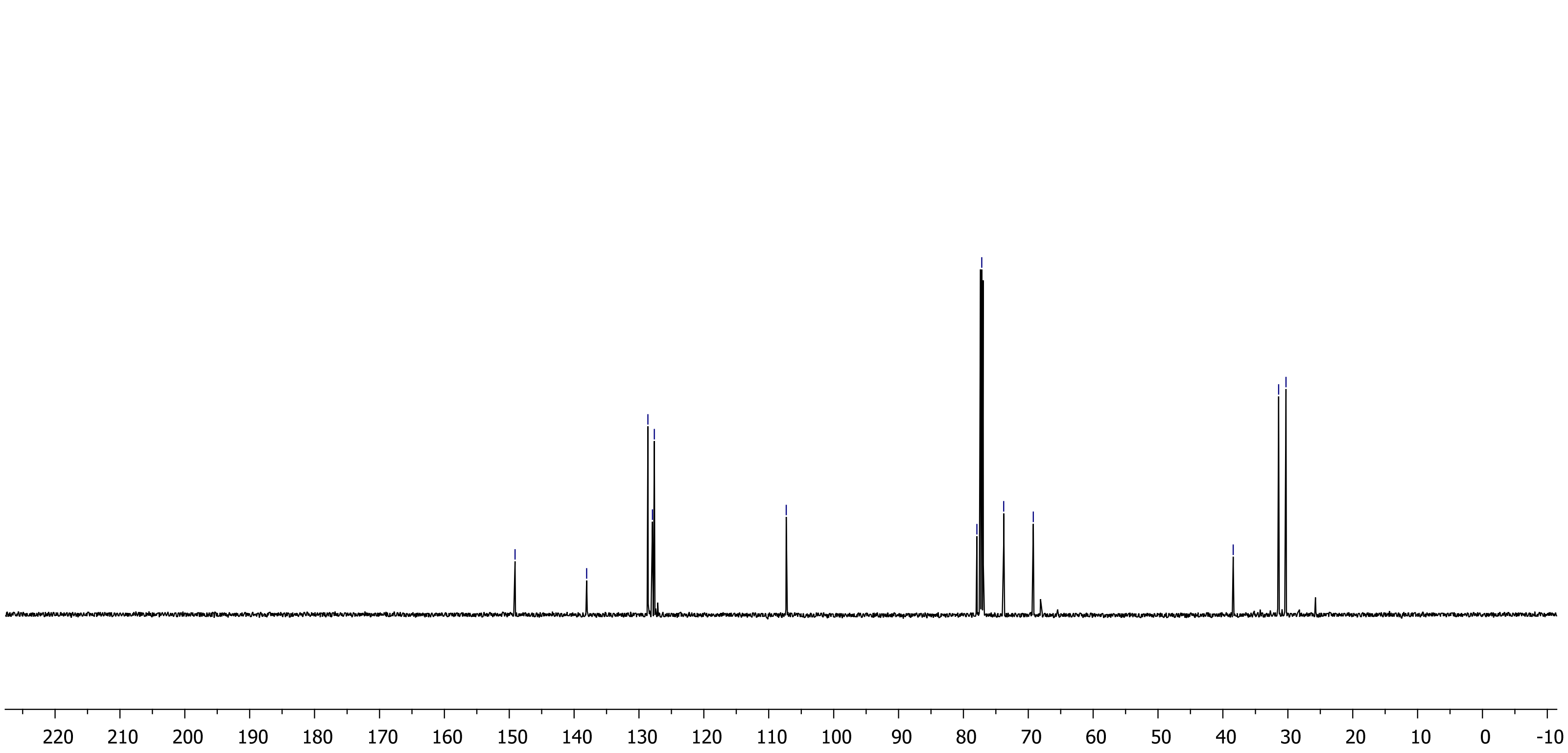

Compound 33

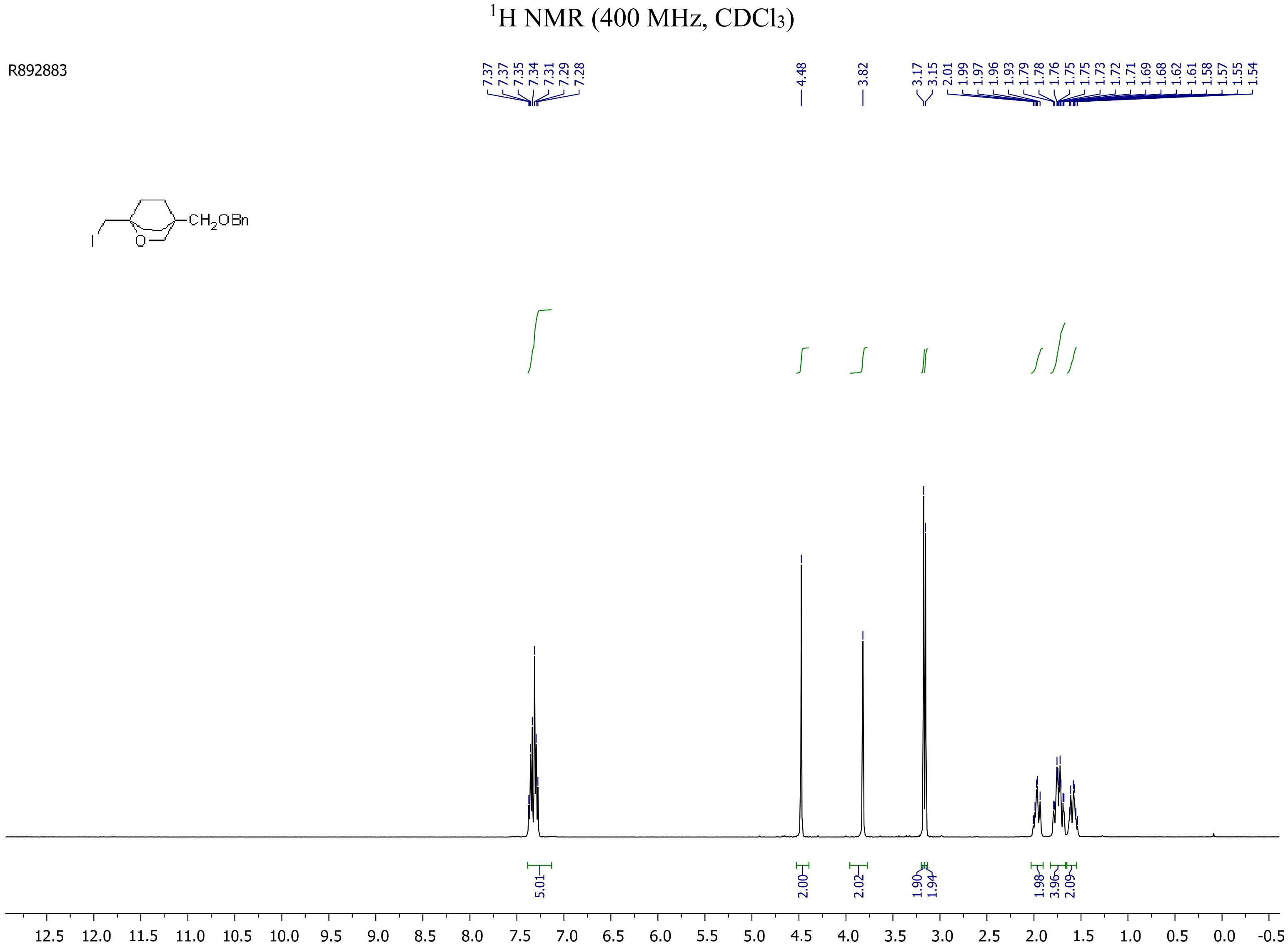

¹H NMR (400 MHz, CDCl3)
R892883
7.37
7.37
7.35
7.34
7.31
7.29
7.28
4.48
3.82
3.17
3.15
2.01
1.99
1.97
1.96
1.93
1.79
1.78
1.76
1.75
1.75
1.73
1.72
1.71
1.69
1.68
1.62
1.61
1.58
1.57
1.55
1.54
CH2OBn
O
I
5.01
2.00
2.02
1.90
1.94
1.98
3.96
2.09
12.5
12.0
11.5
11.0
10.5
10.0
9.5
9.0
8.5
8.0
7.5
7.0
6.5
6.0
5.5
5.0
4.5
4.0
3.5
3.0
2.5
2.0
1.5
1.0
0.5
0.0
-0.5

$^{13}C\{^{1}H\}$ NMR (126 MHz, $CDCl_3$)

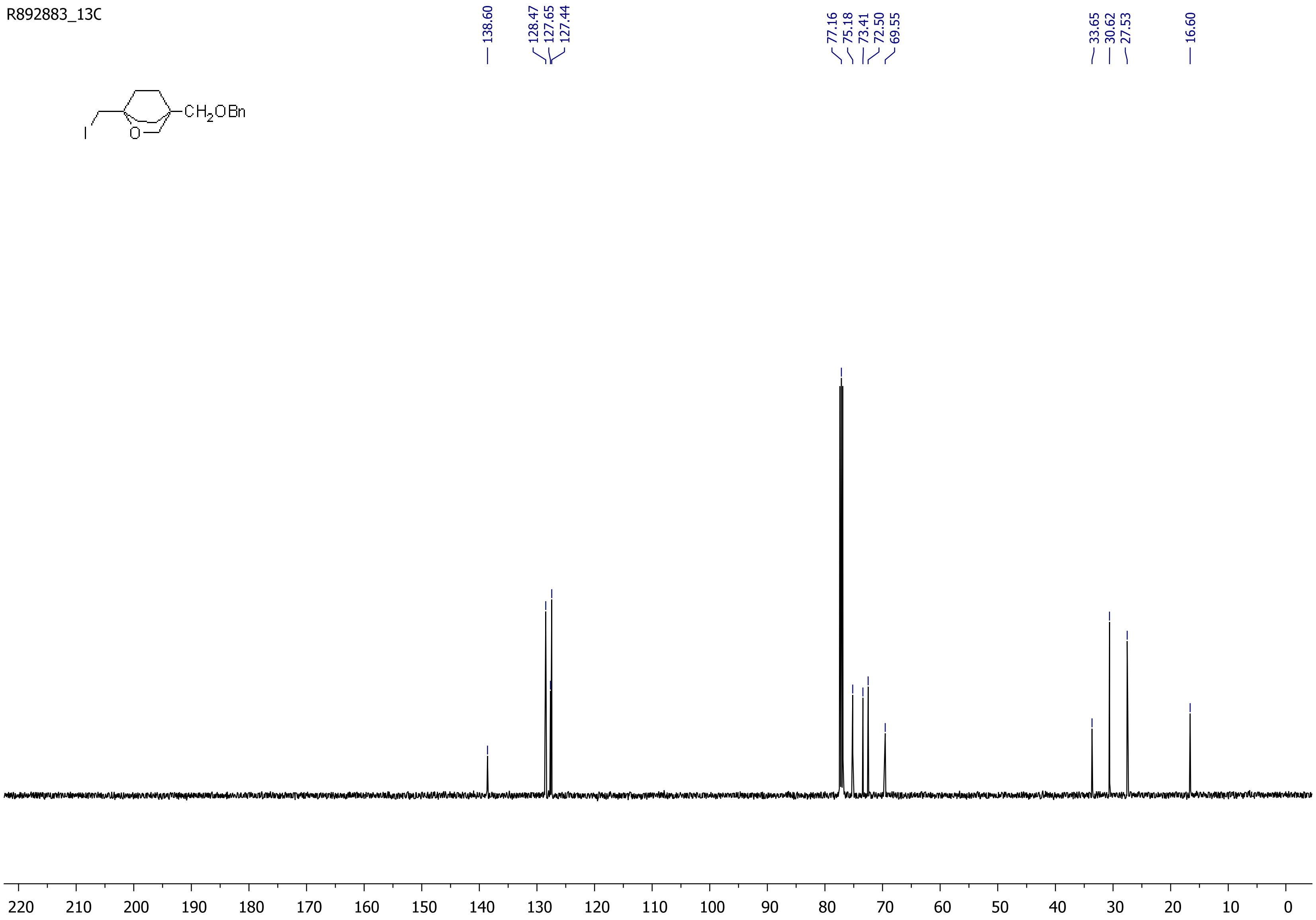

Compound SI-10

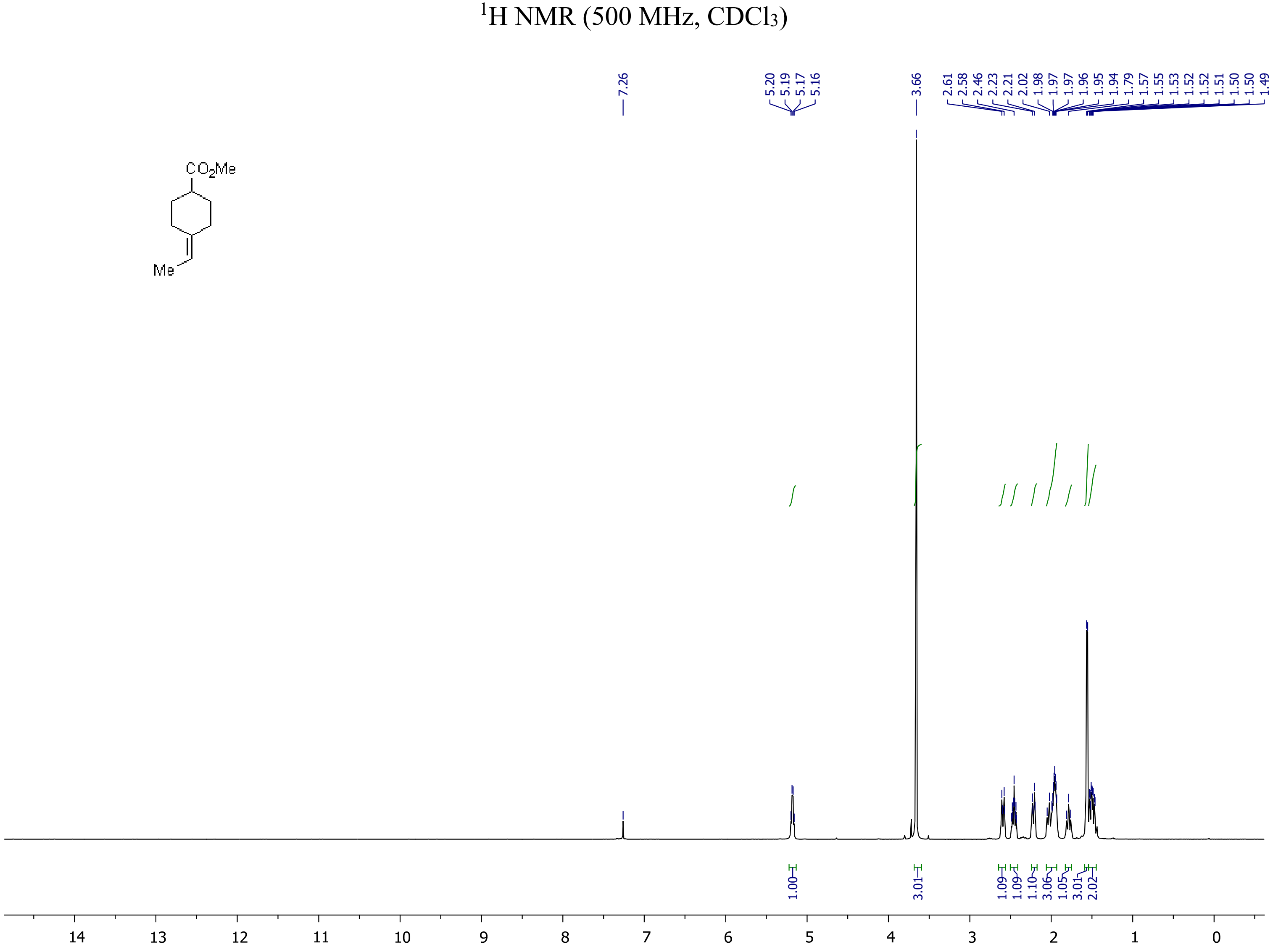

$^{13}C\{^{1}H\}$ NMR (126 MHz, $CDCl_3$)

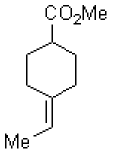

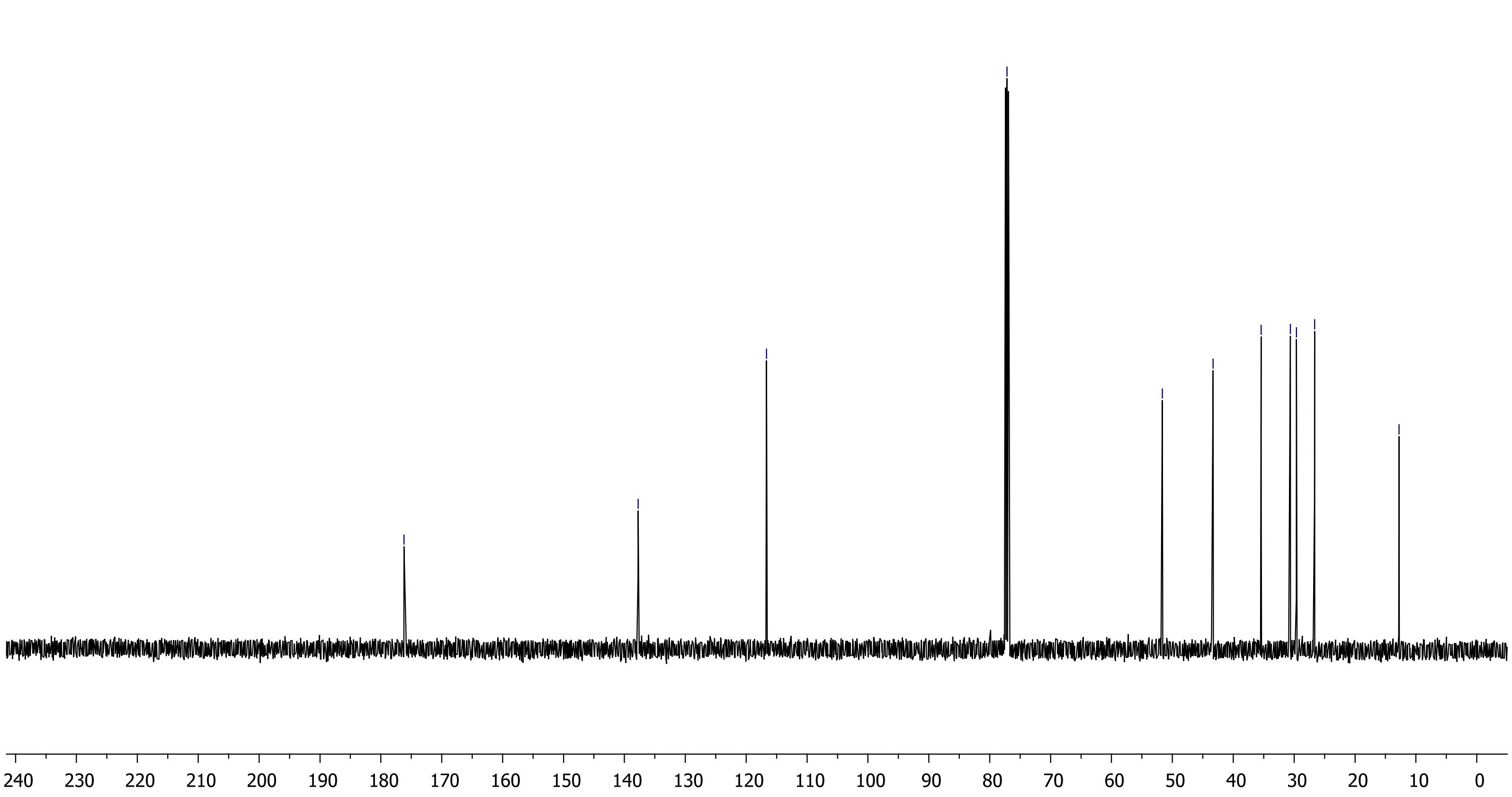

Compound 34

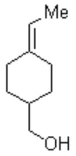

^{1}H NMR (500 MHz, CDCl$_3$)

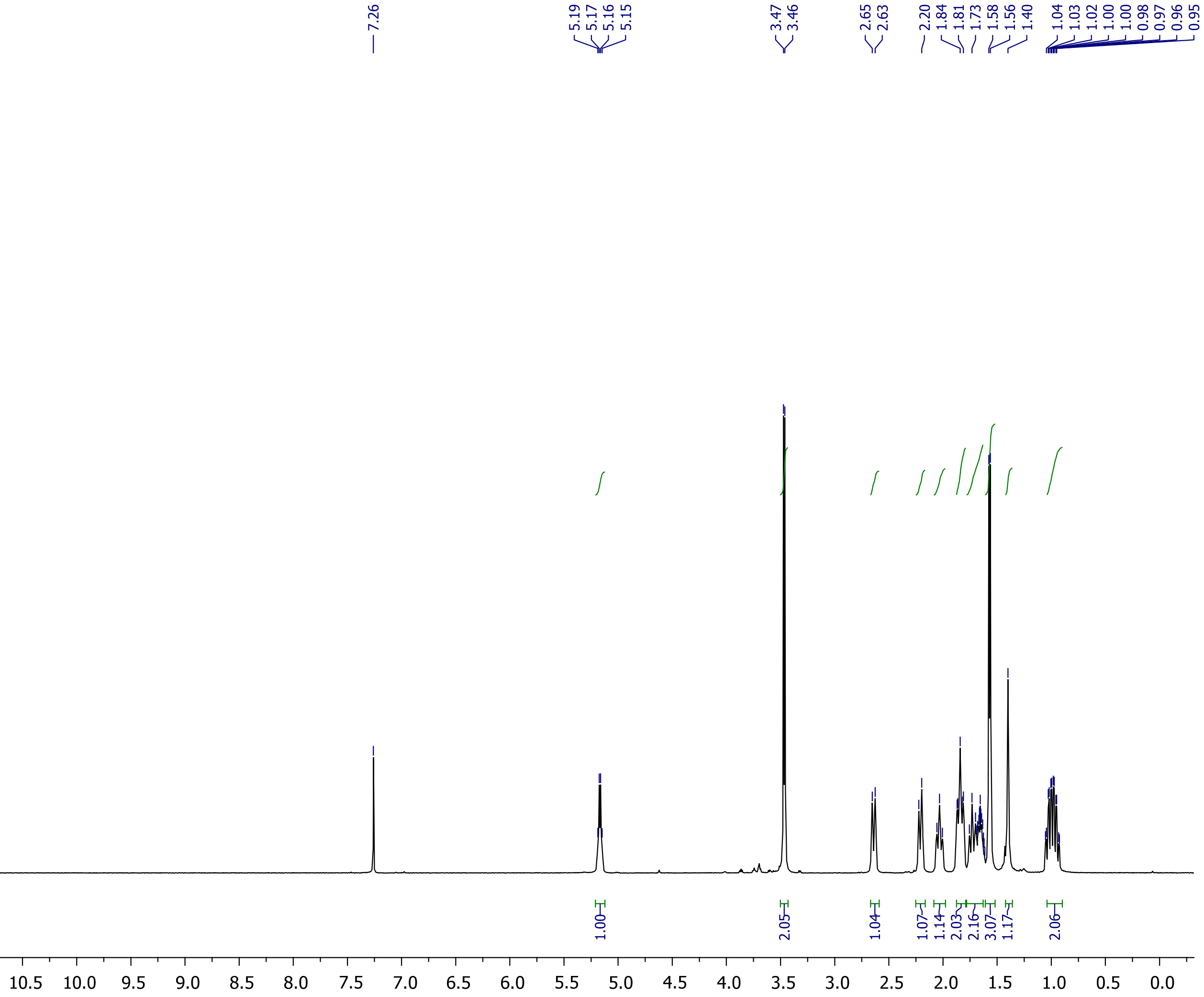

$^{13}C\{^{1}H\}$ NMR (126 MHz, $CDCl_3$)

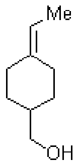

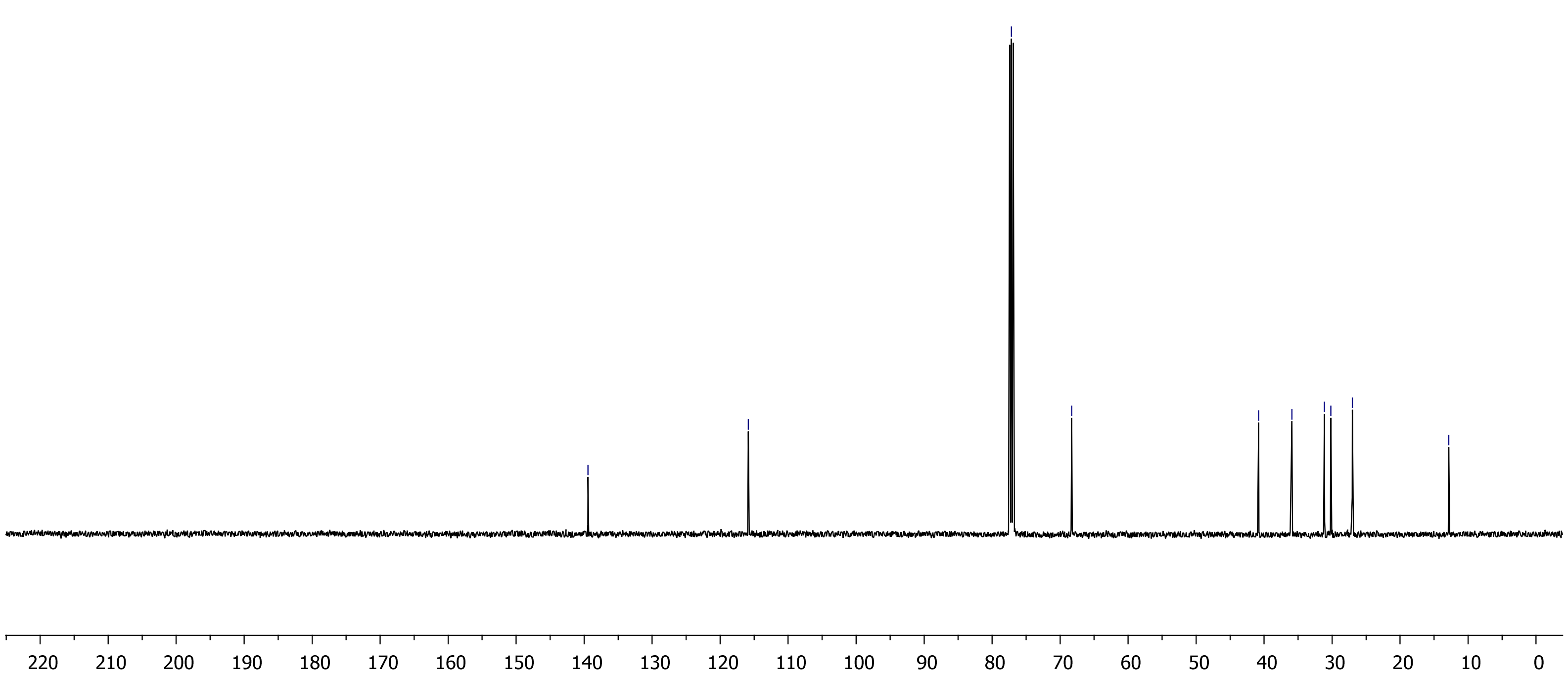

Compound 35

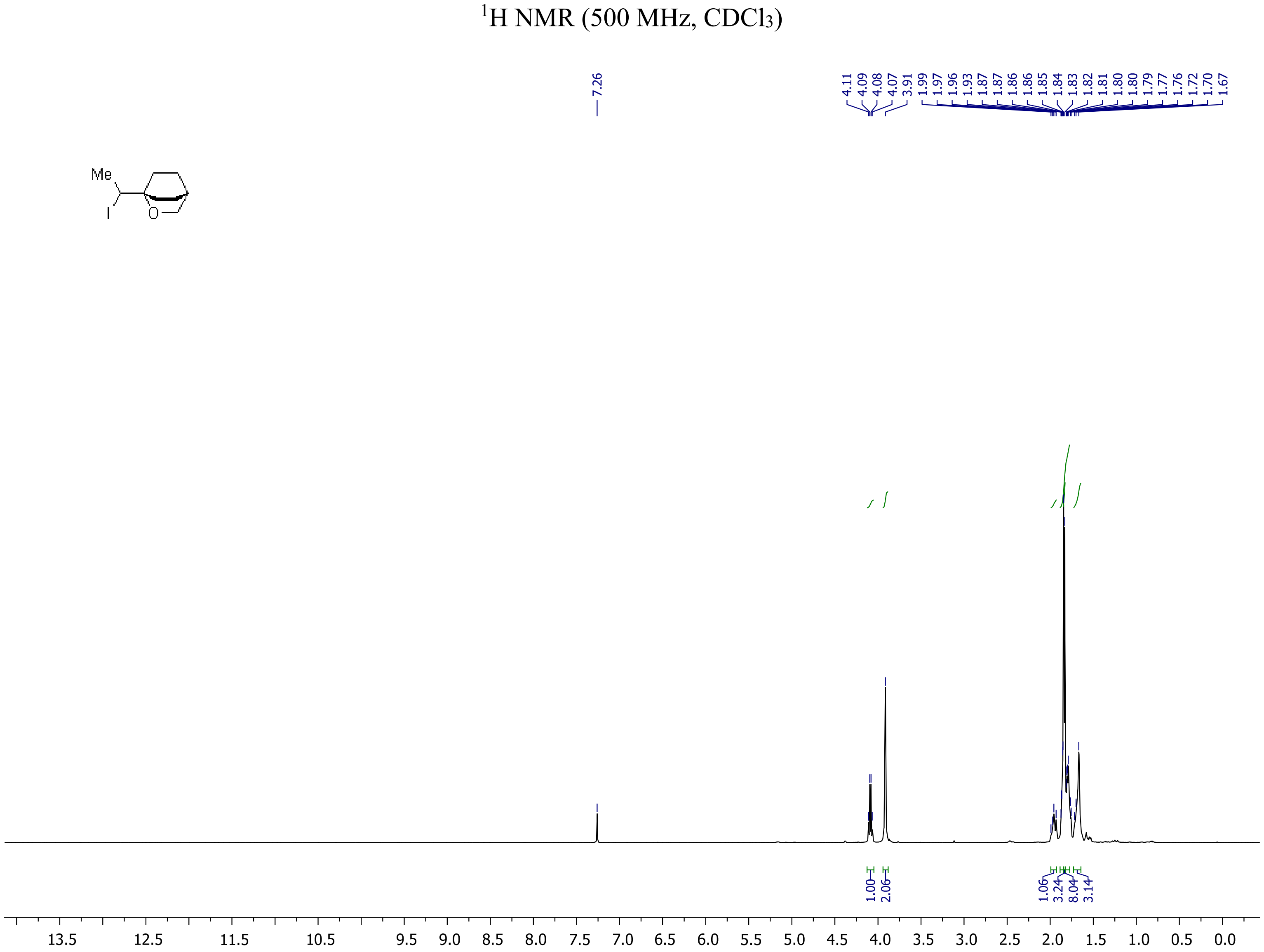
^{1}H NMR (500 MHz, $CDCl_3$)
Me
I
O
7.26
4.11 4.09 4.08 4.07 3.91 1.99 1.97 1.96 1.93 1.87 1.87 1.86 1.85 1.84 1.83 1.82 1.81 1.80 1.80 1.79 1.77 1.76 1.72 1.70 1.67
1.00 2.06 1.06 3.24 8.04 3.14
13.5 12.5 11.5 10.5 9.5 9.0 8.5 8.0 7.5 7.0 6.5 6.0 5.5 5.0 4.5 4.0 3.5 3.0 2.5 2.0 1.5 1.0 0.5 0.0

$^{13}C\{^{1}H\}$ NMR (151 MHz, $CDCl_3$)

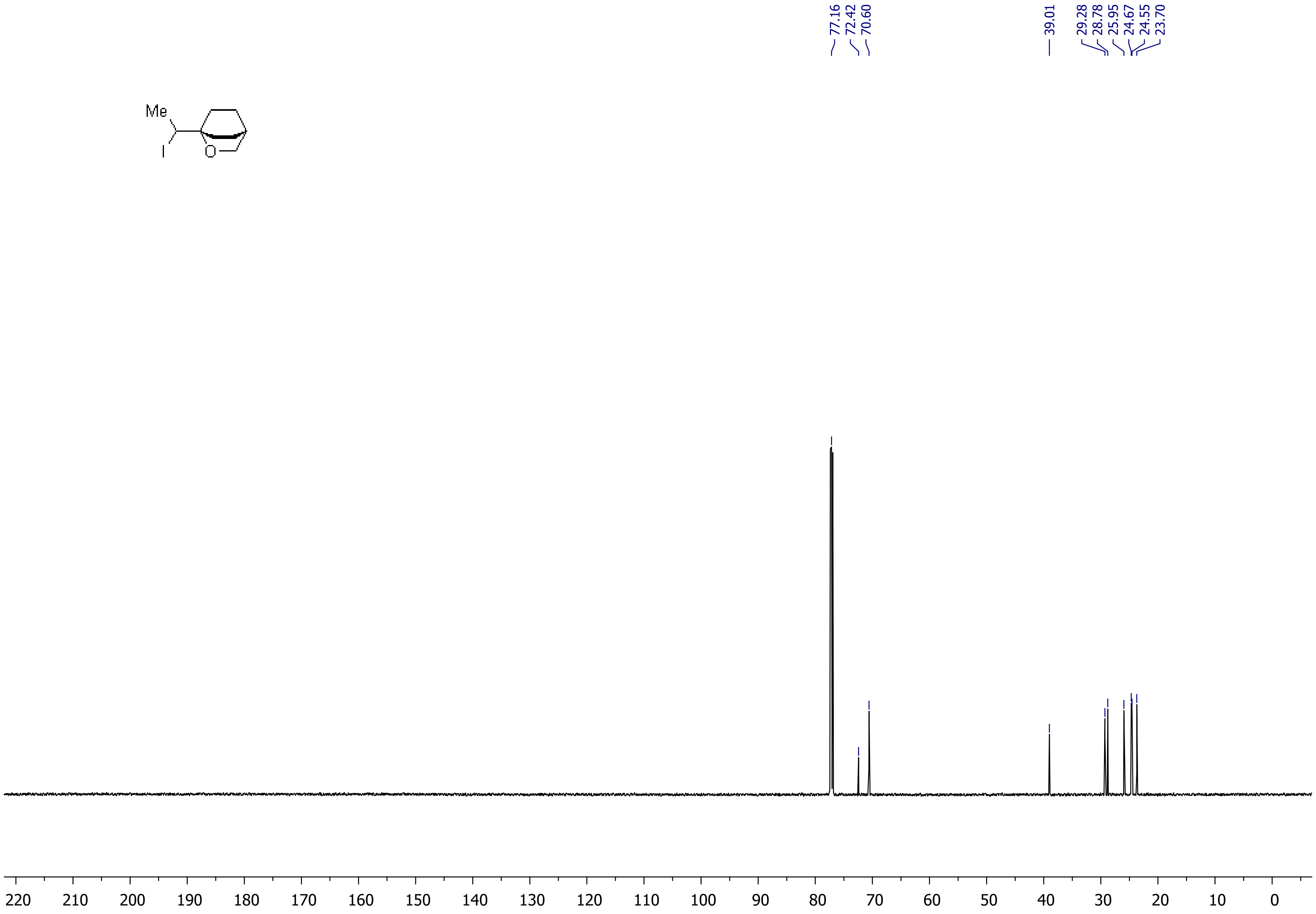

Compound SI-11

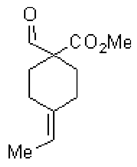

^{1}H NMR (500 MHz, $CDCl_3$)

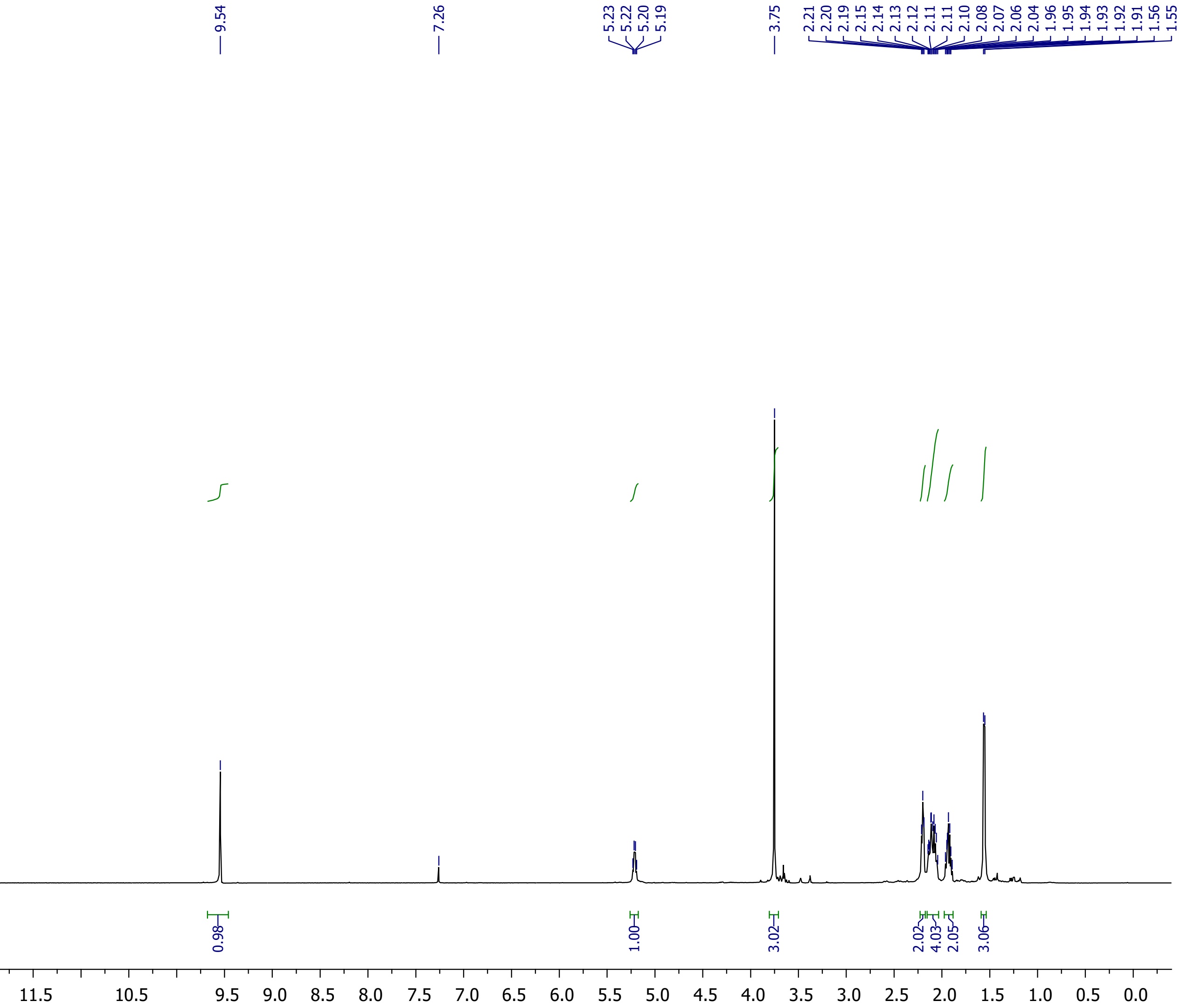

$^{13}C\{^{1}H\}$ NMR (126 MHz, $CDCl_3$)

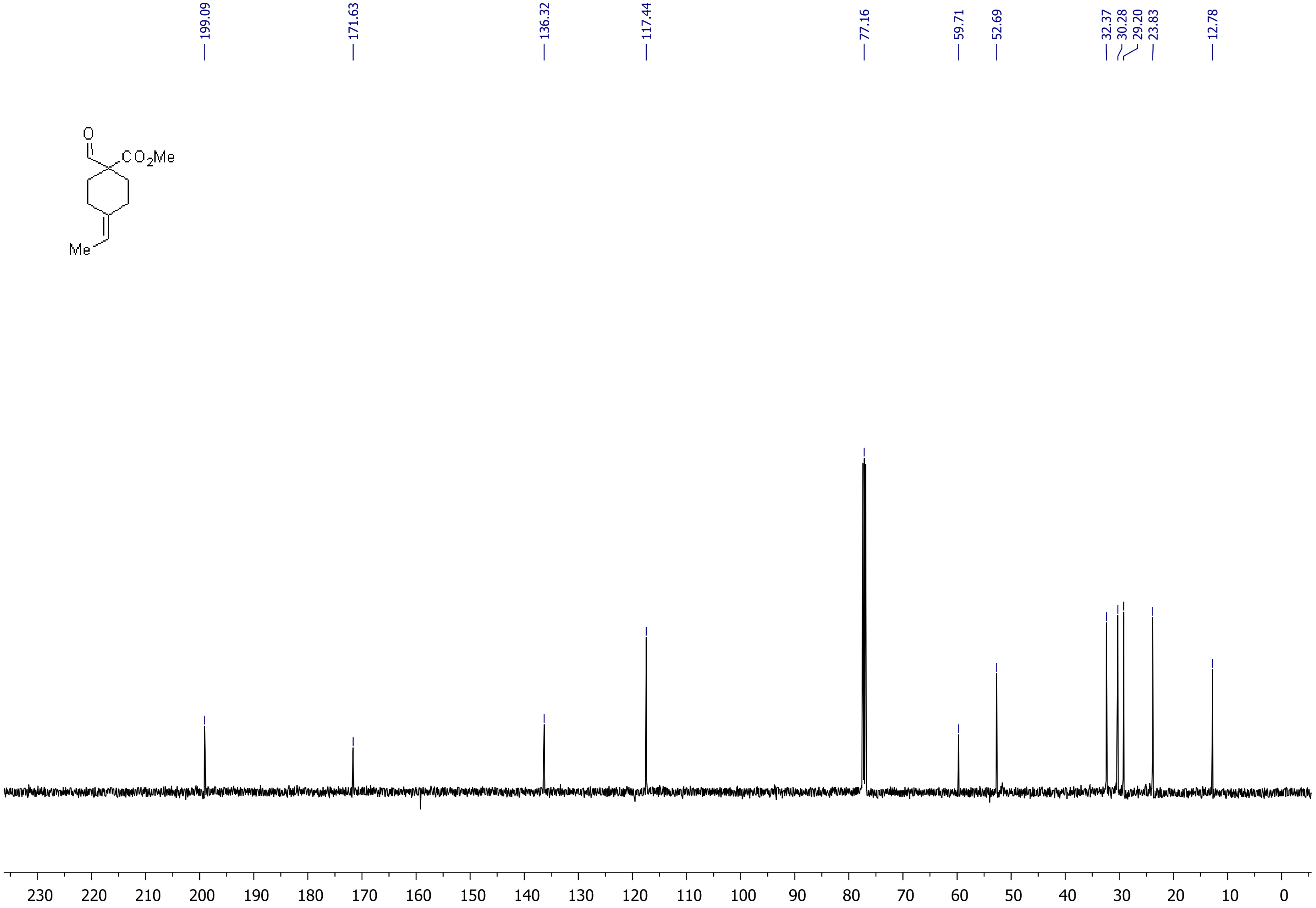

Compound 36

^{1}H NMR (500 MHz, CDCl$_3$)

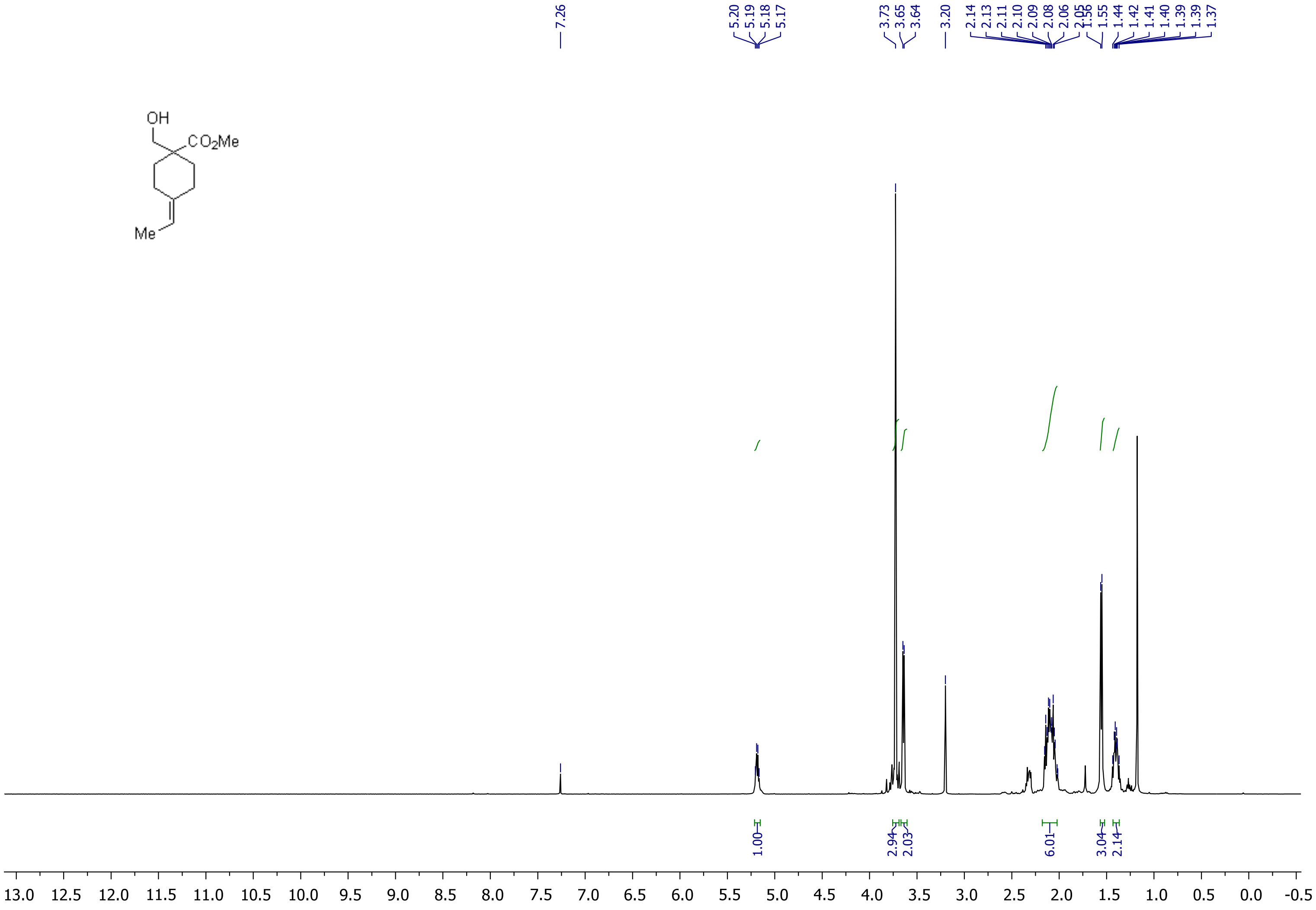

$^{13}C\{^1H\}$ NMR (126 MHz, $CDCl_3$)

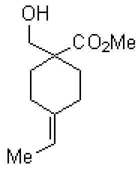

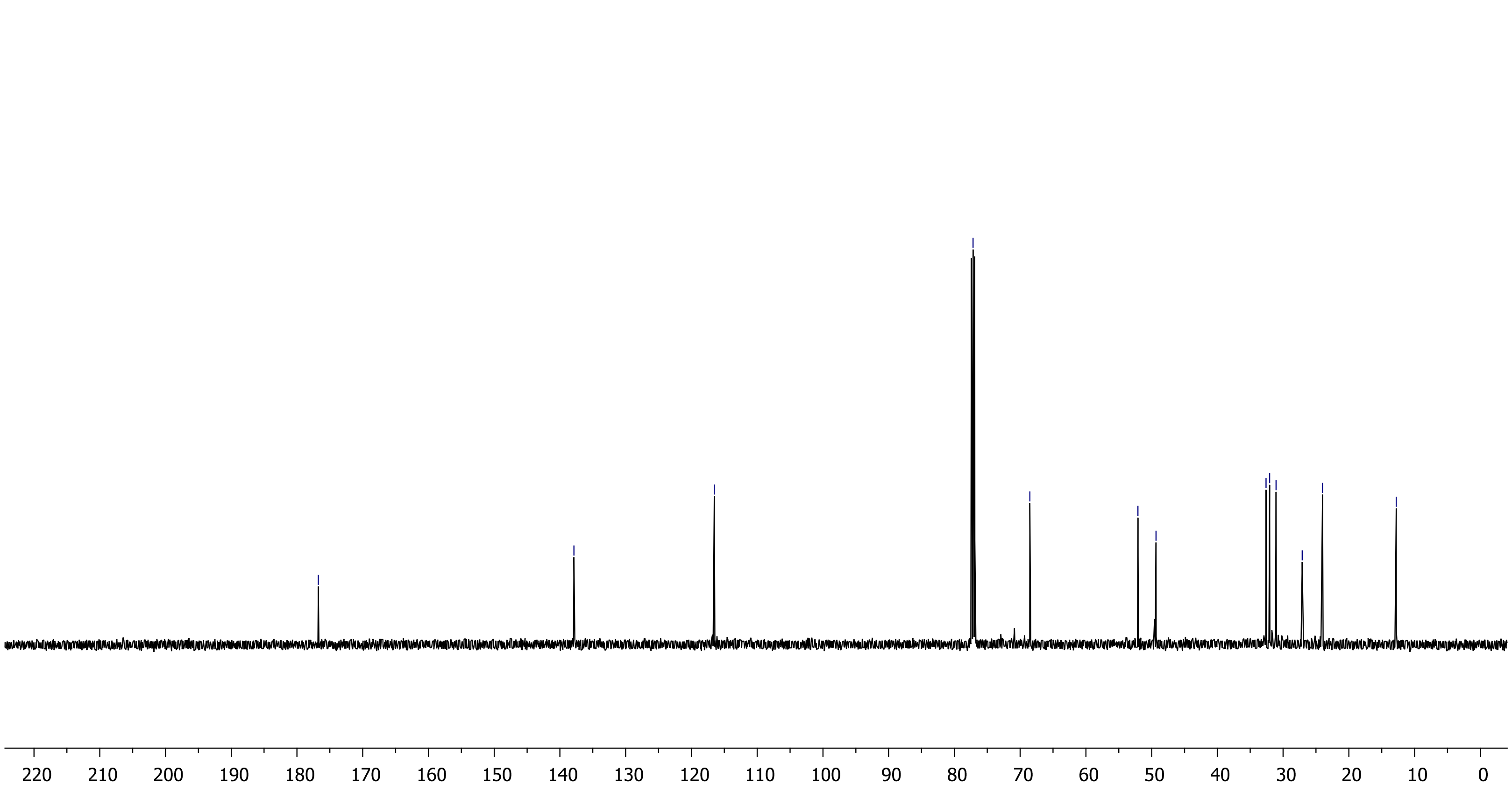

Compound 37

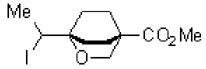

Me
I
O
CO2Me

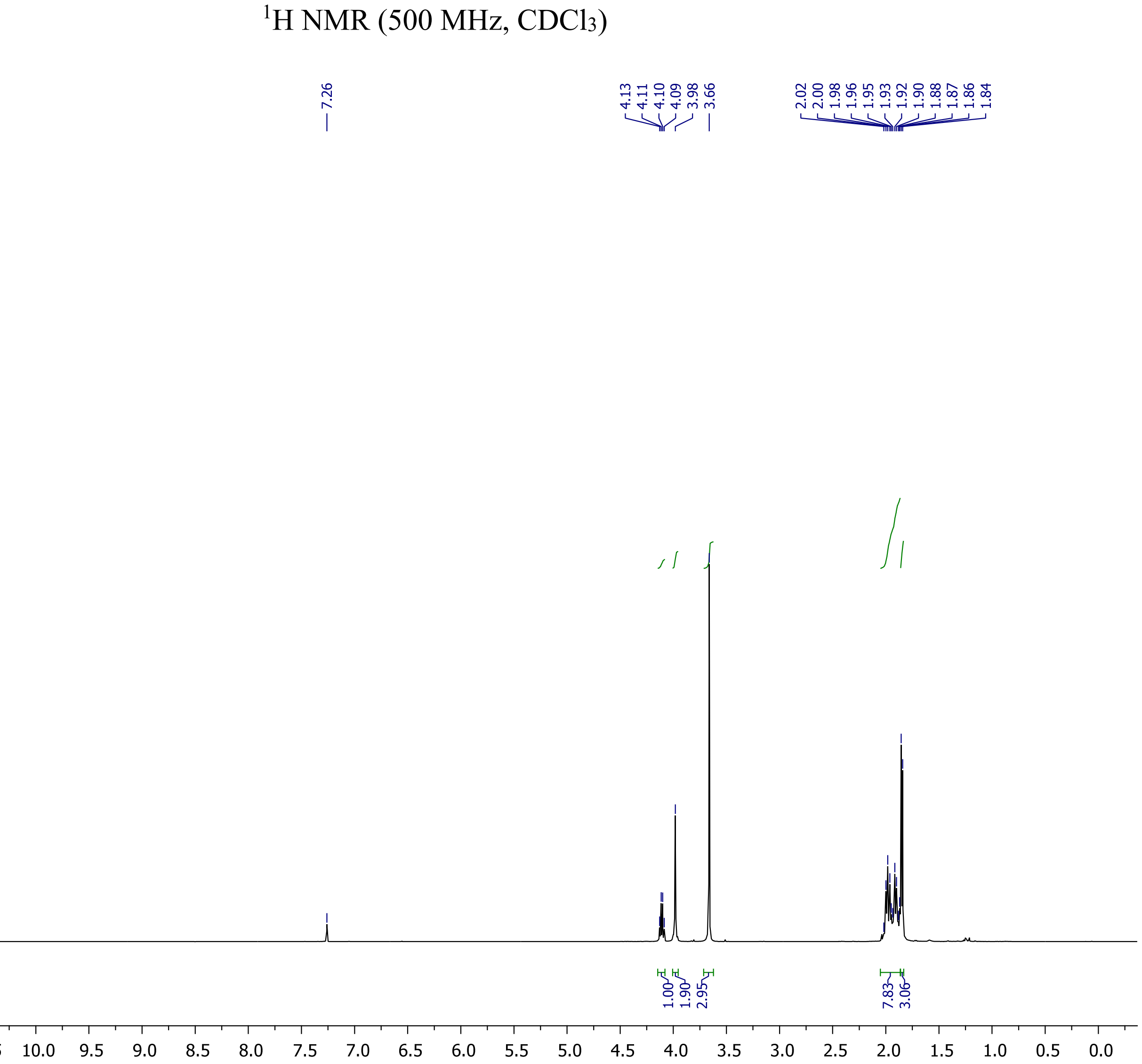

^{1}H NMR (500 MHz, CDCl3)
7.26
4.13
4.11
4.10
4.09
3.98
3.66
2.02
2.00
1.98
1.96
1.95
1.93
1.92
1.90
1.88
1.87
1.86
1.84
1.00
1.90
2.95
7.83
3.06
12.5 12.0 11.5 11.0 10.5 10.0 9.5 9.0 8.5 8.0 7.5 7.0 6.5 6.0 5.5 5.0 4.5 4.0 3.5 3.0 2.5 2.0 1.5 1.0 0.5 0.0

$^{13}C\{^1H\}$ NMR (101 MHz, $CDCl_3$)

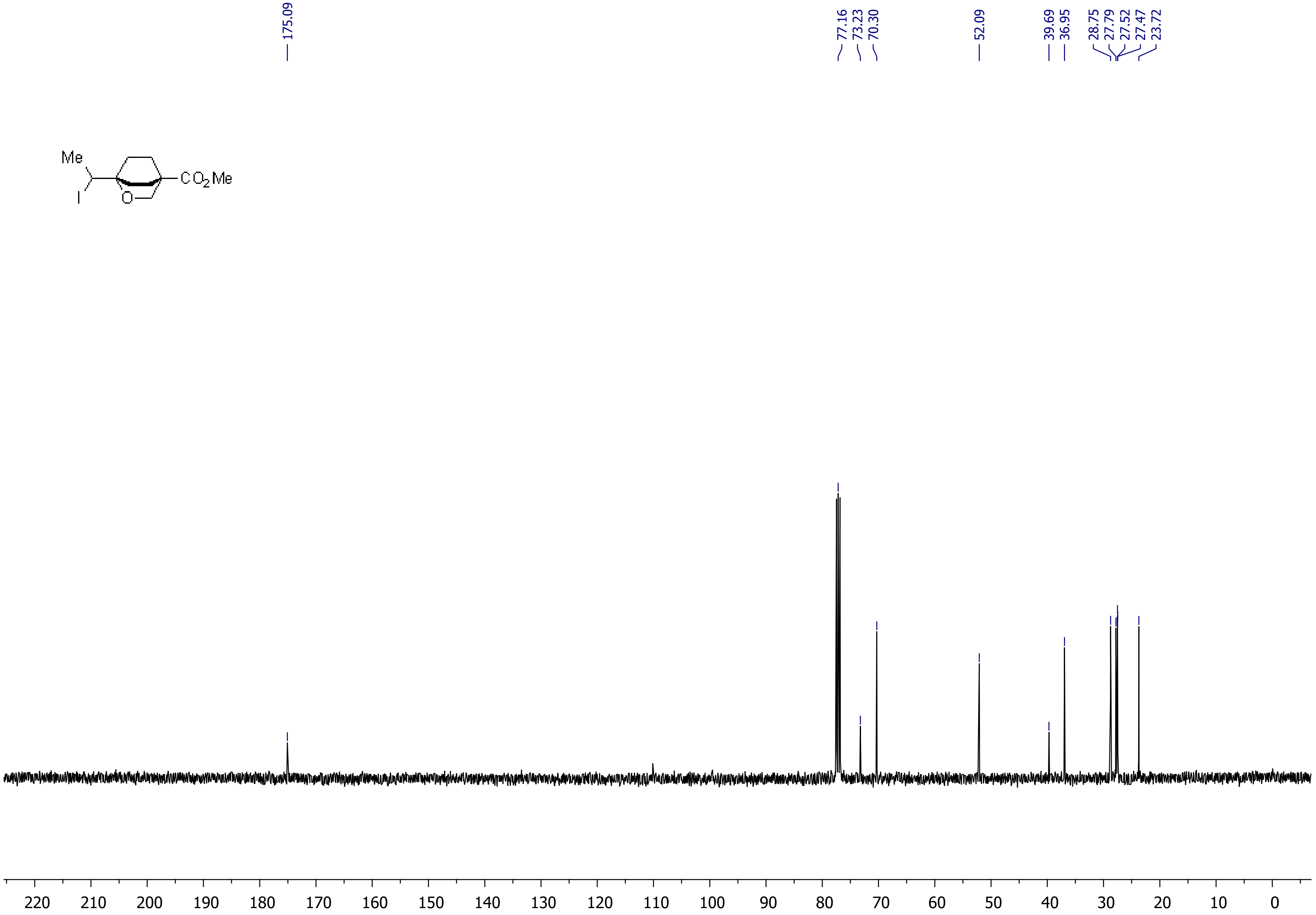

Compound 39

^{1}H NMR (400 MHz, $CDCl_3$)

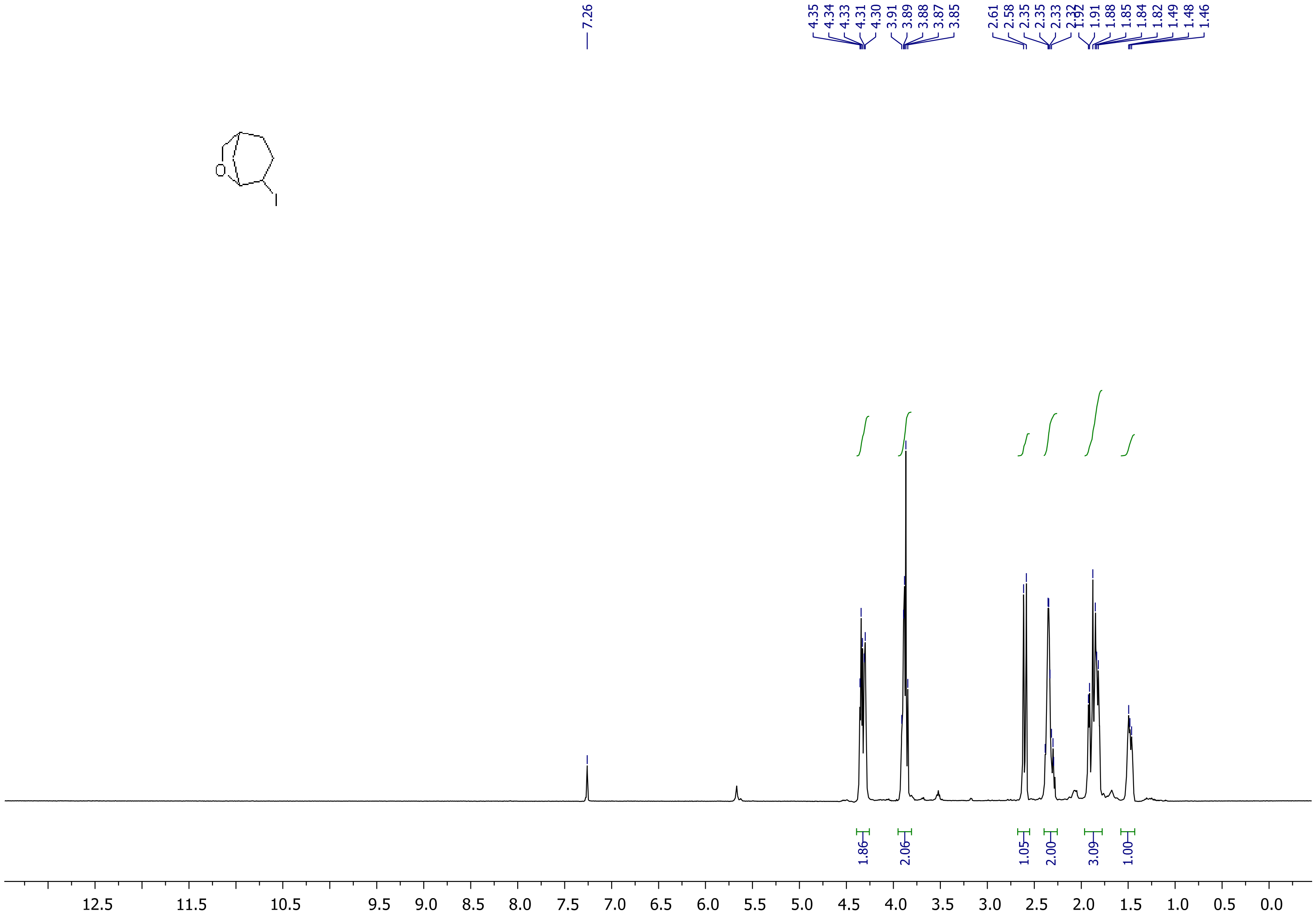

$^{13}C\{^1H\}$ NMR (126 MHz, $CDCl_3$)

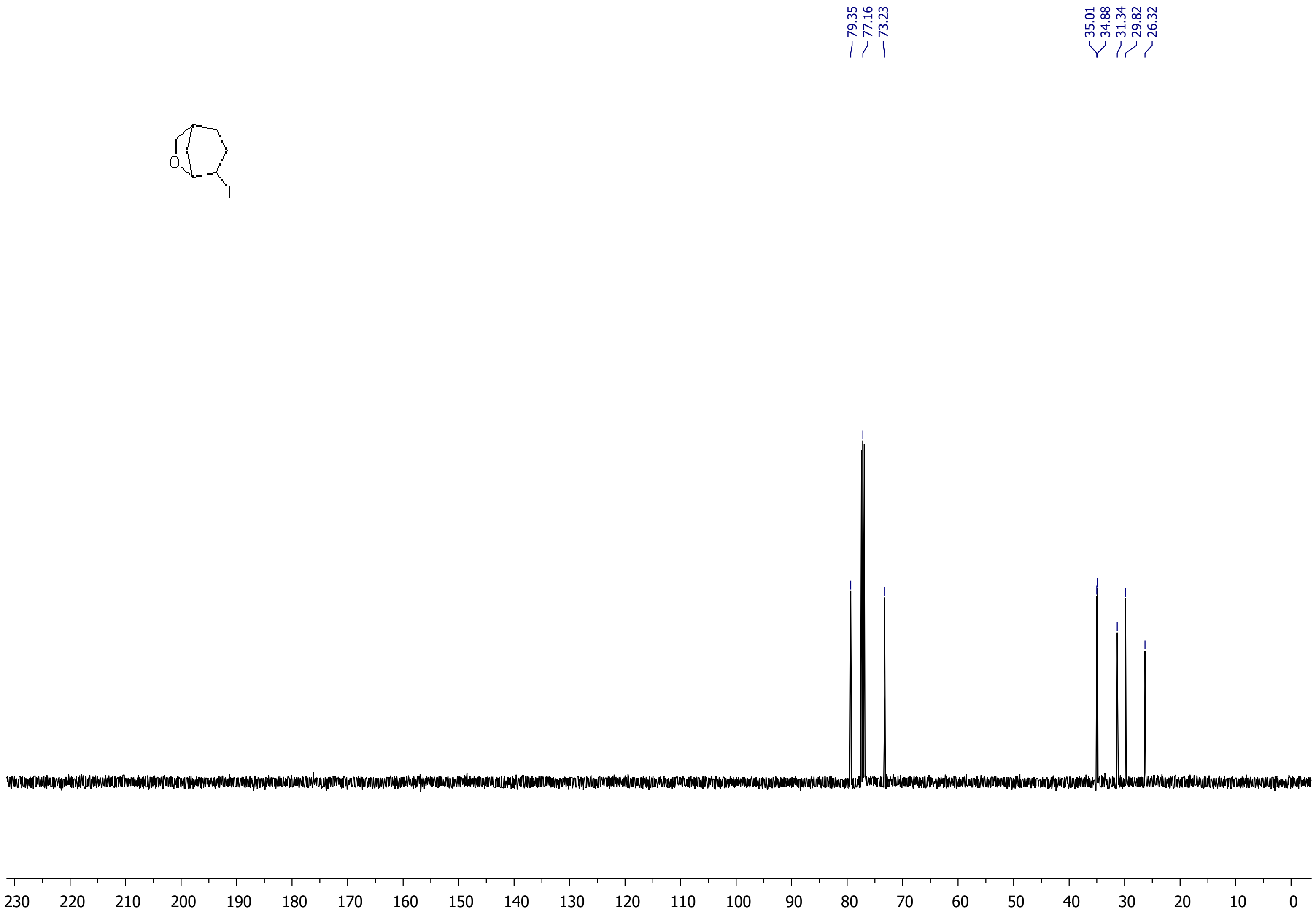

Compound 40

^{1}H NMR (500 MHz, $CDCl_3$)

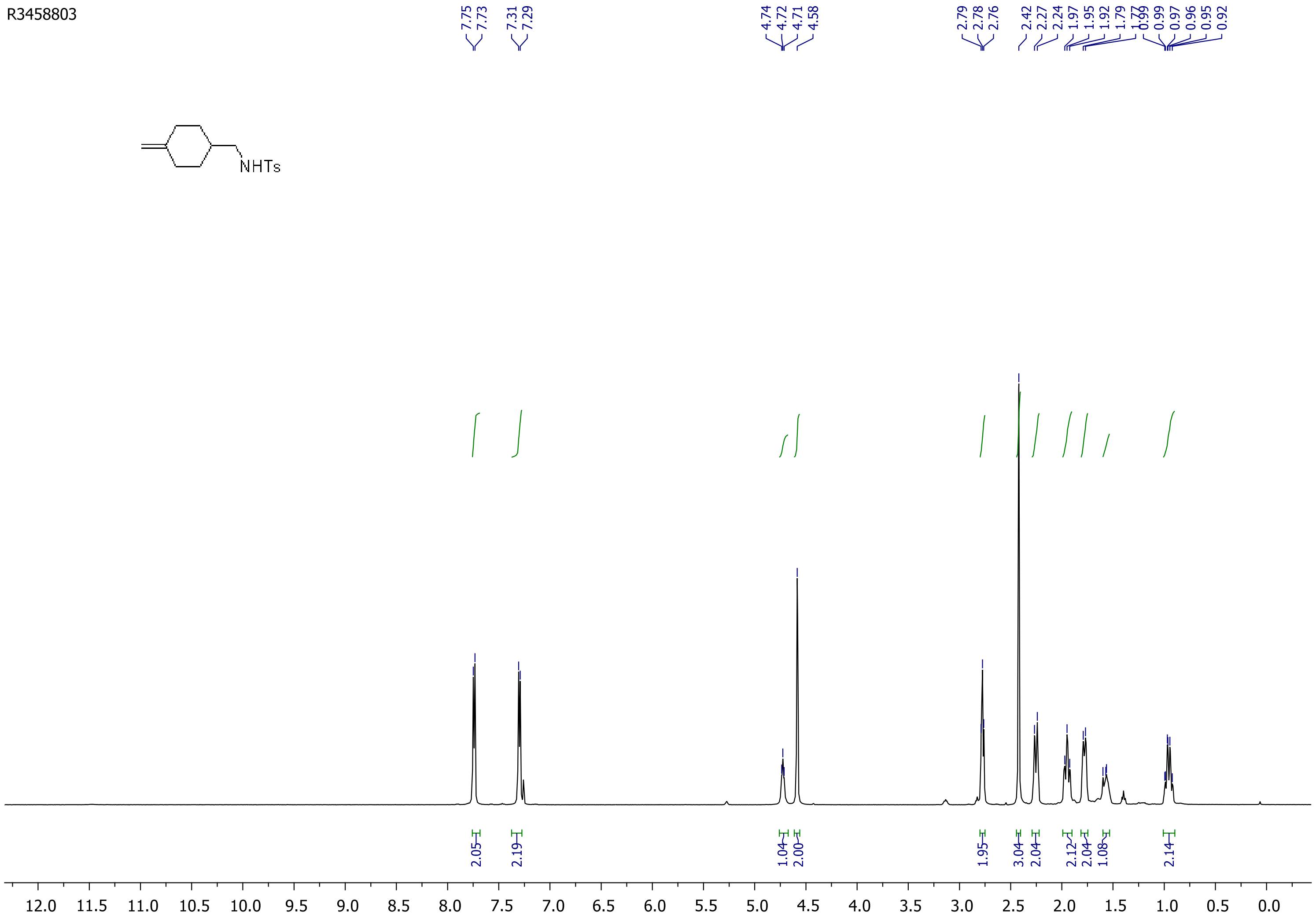

$^{13}C\{^{1}H\}$ NMR (126 MHz, $CDCl_3$)

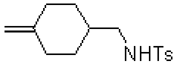

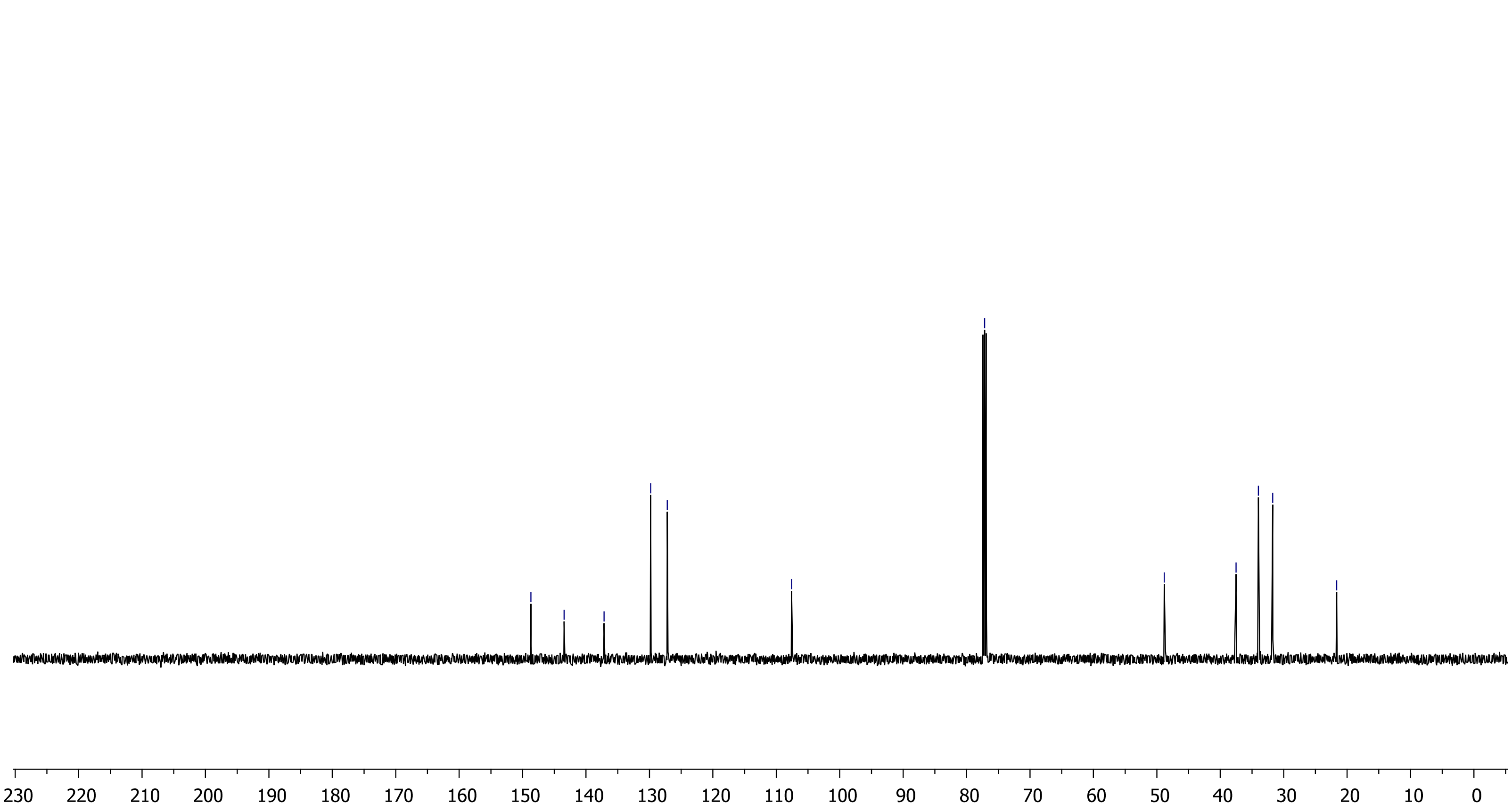

Compound SI-12

^{1}H NMR (500 MHz, $CDCl_3$)

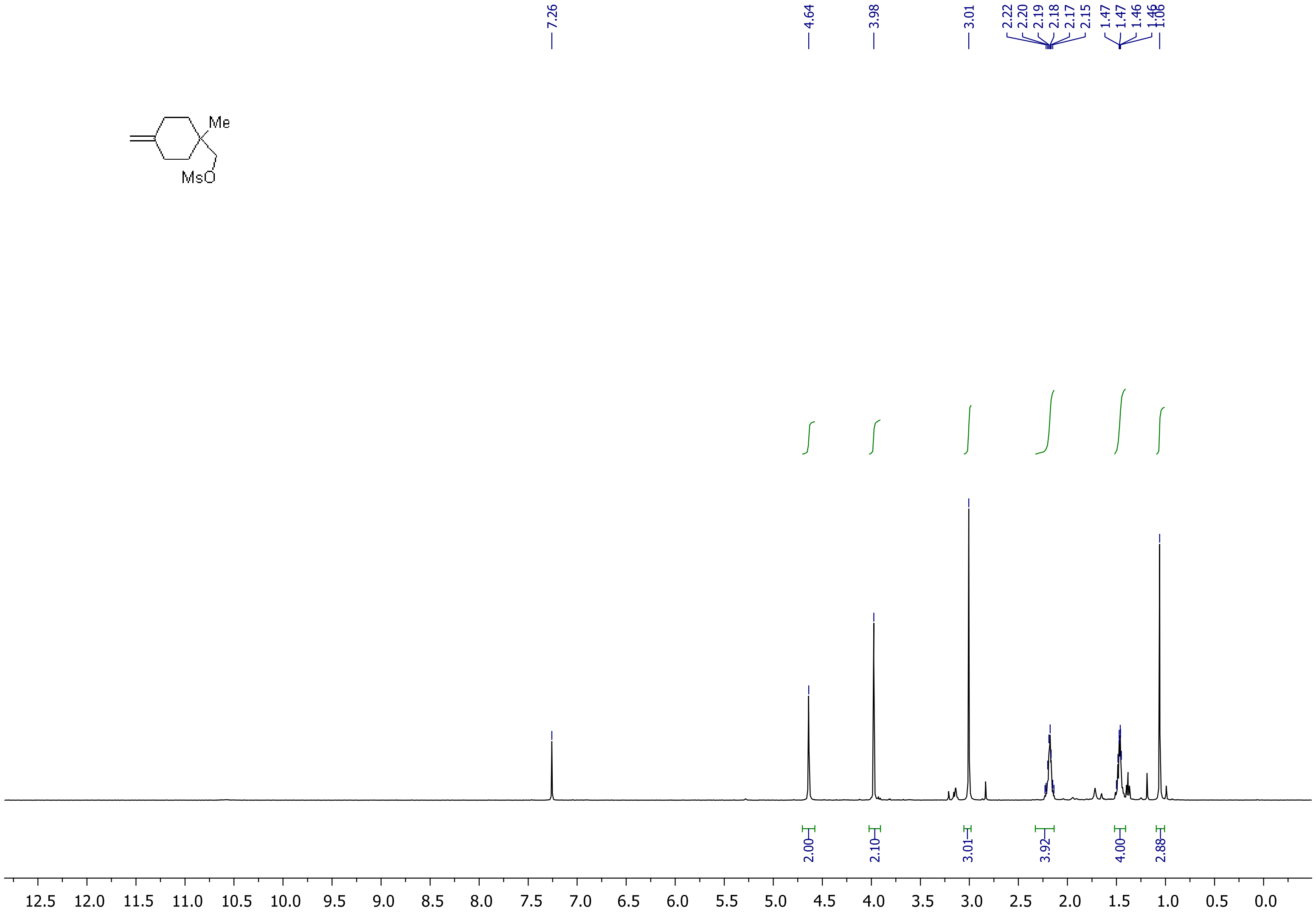

$^{13}C\{^{1}H\}$ NMR (151 MHz, $CDCl_3$)

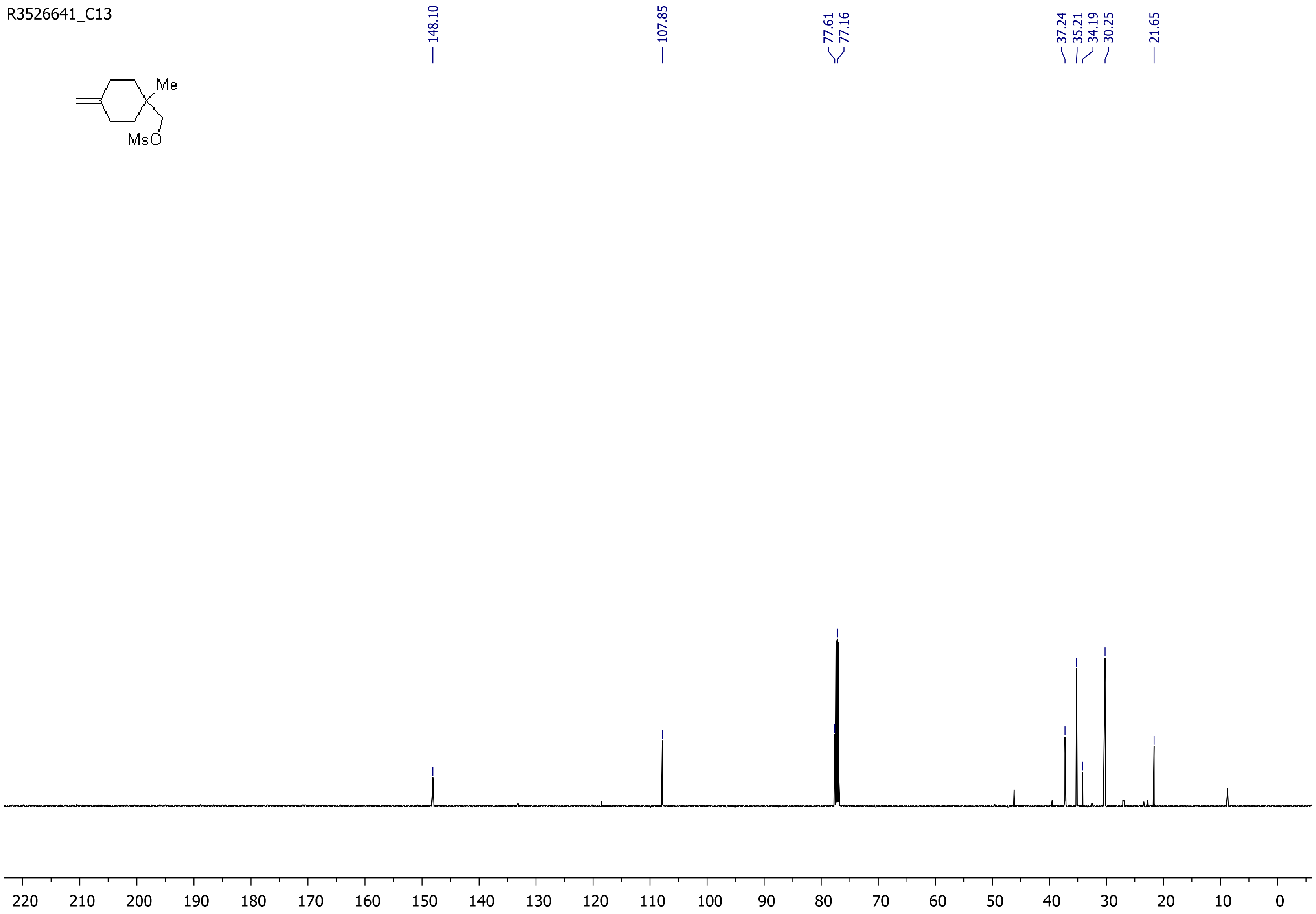

Compound SI-13

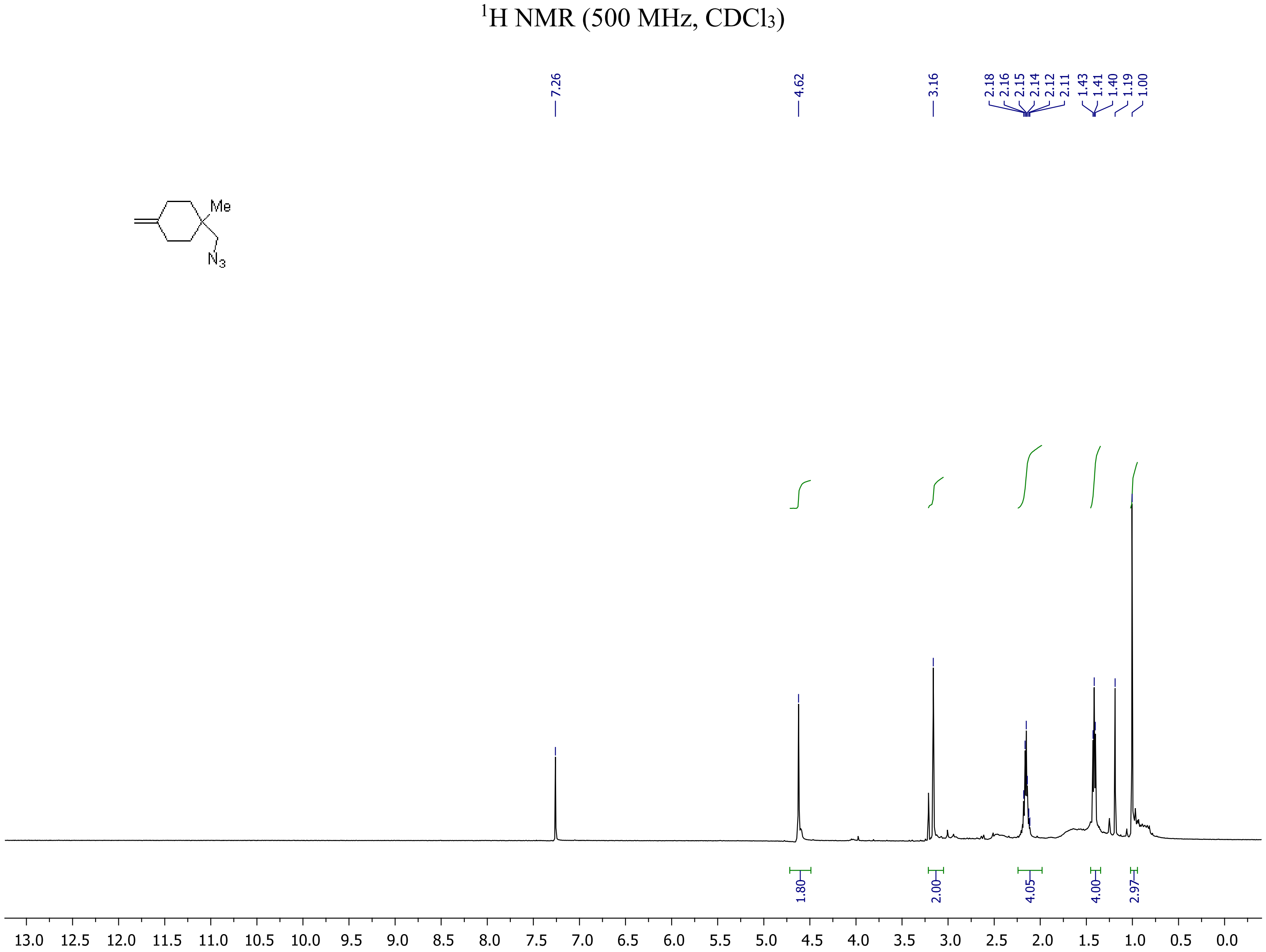

$^{13}C\{^{1}H\}$ NMR (101 MHz, $CDCl_3$)

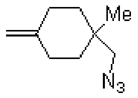

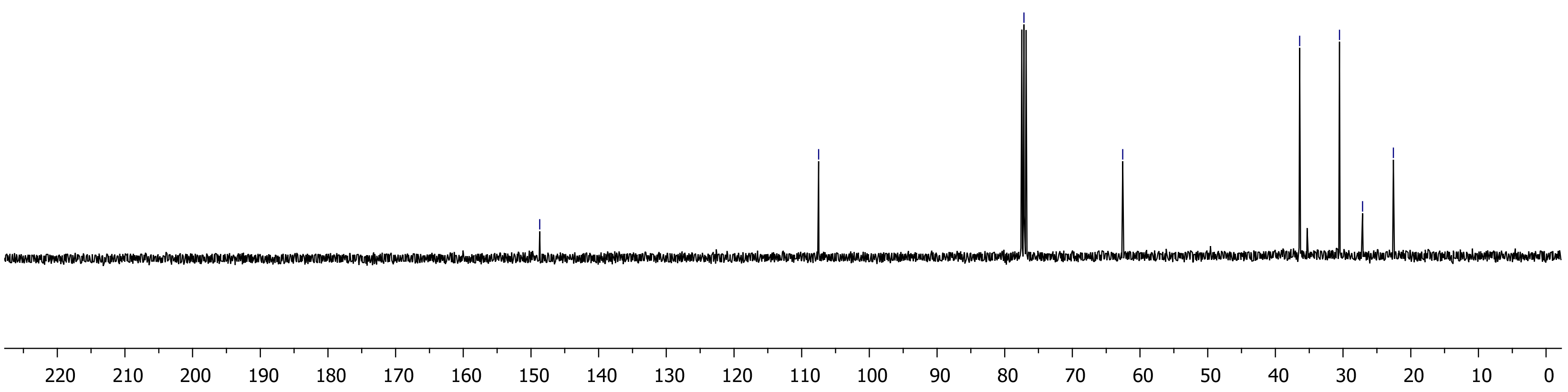

Compound SI-14

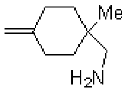

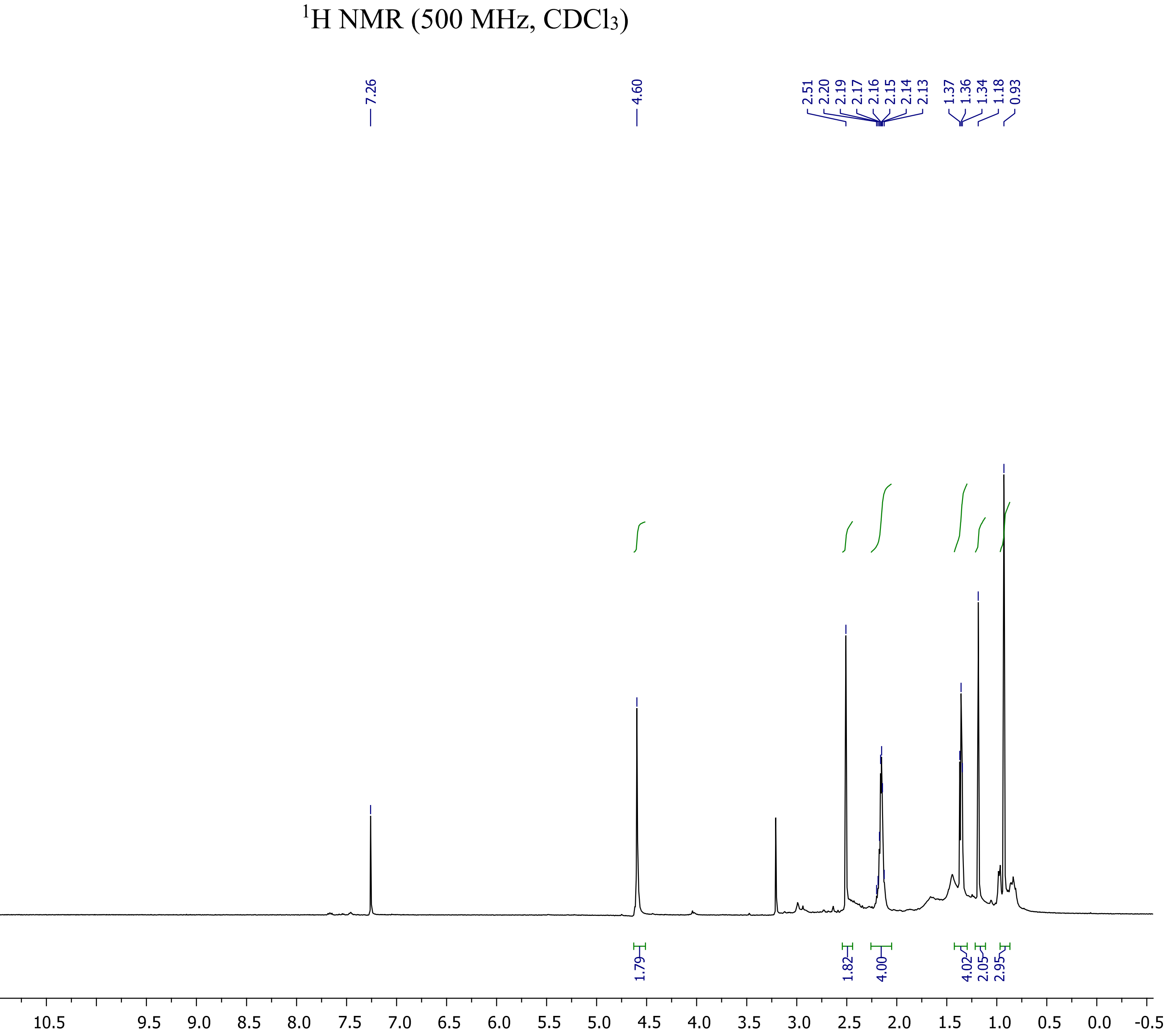

$^{13}C\{^1H\}$ NMR (151 MHz, $CDCl_3$)

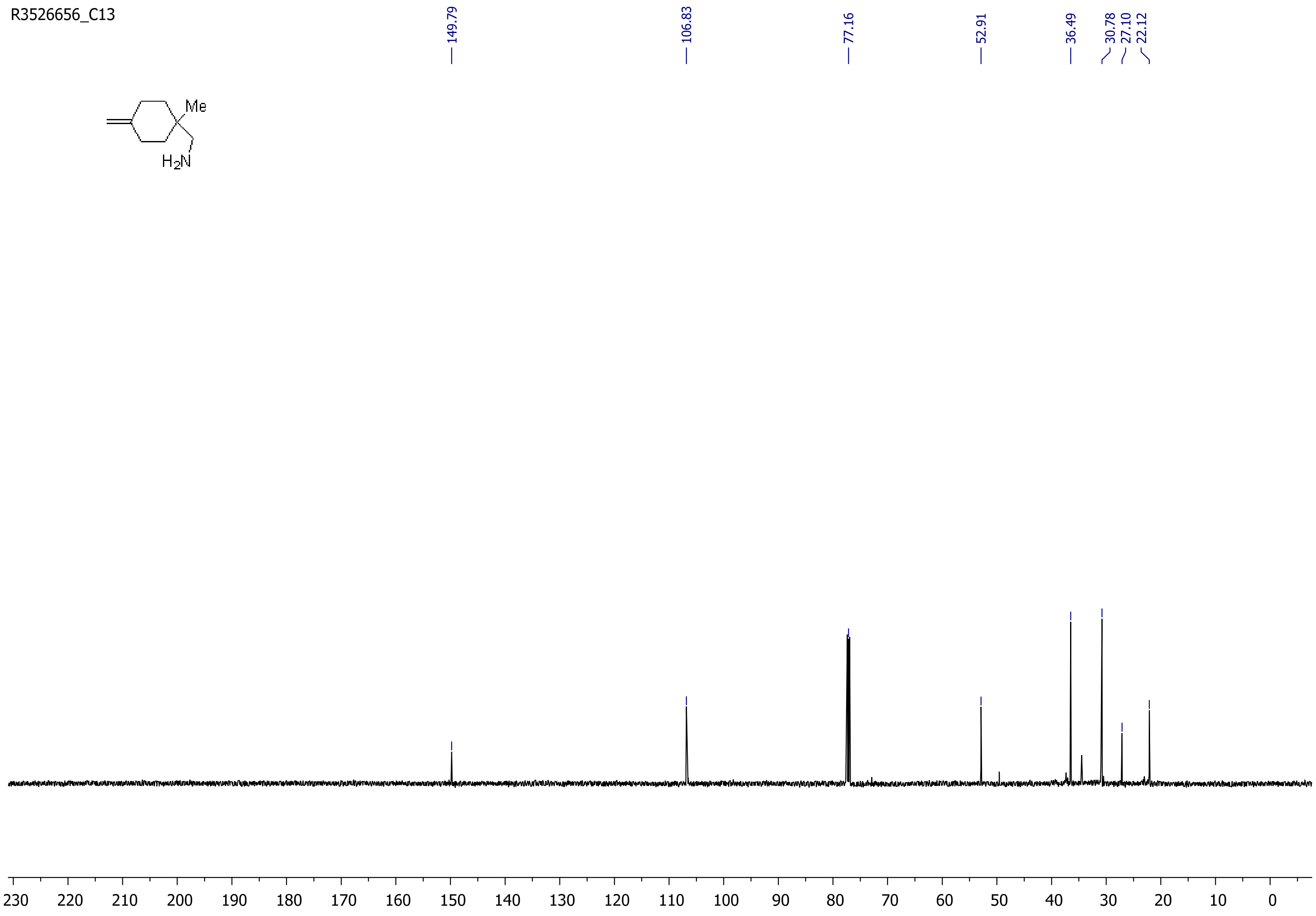

Compound 42

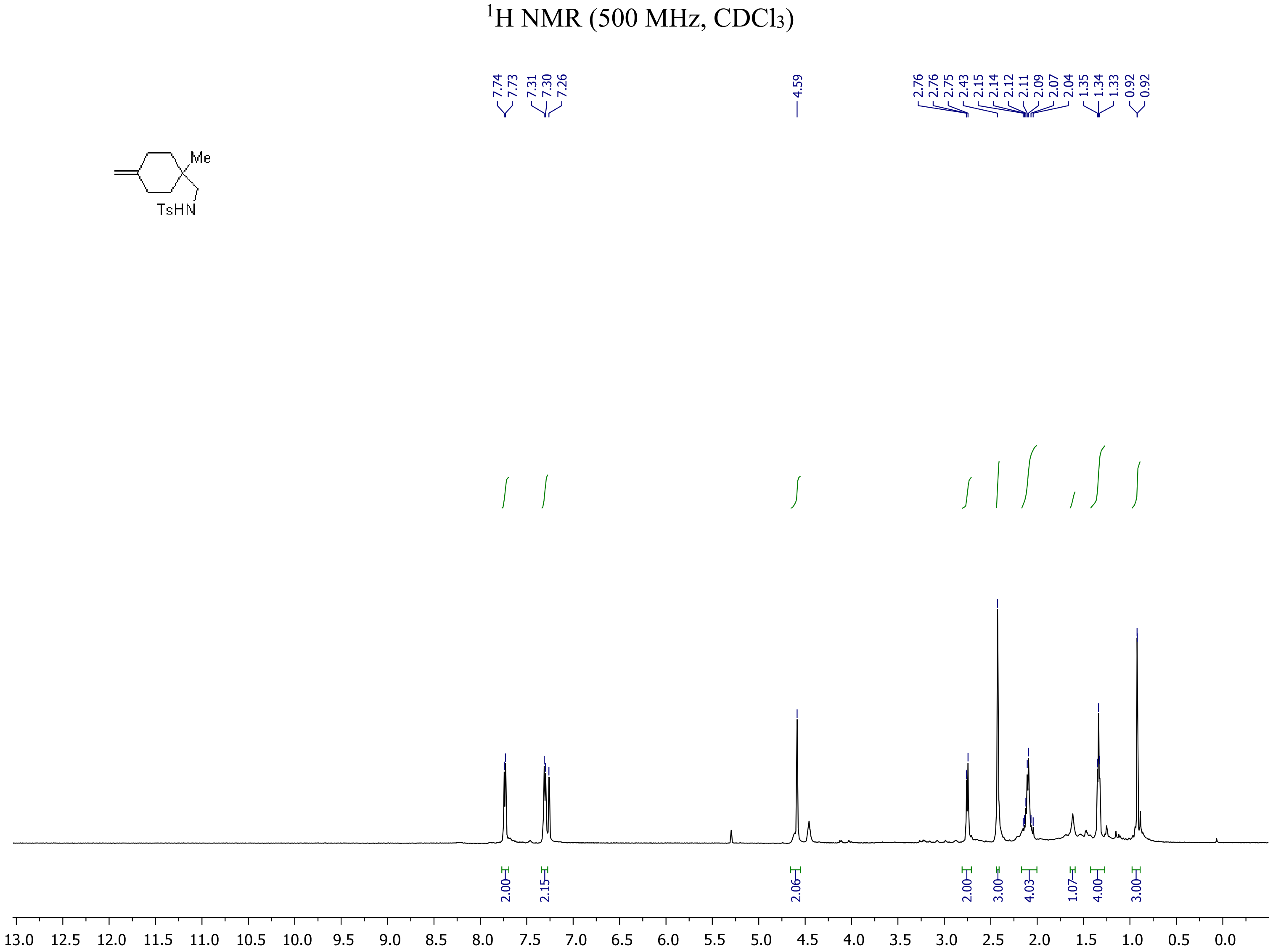

1H NMR (500 MHz, CDCl3)
7.74
7.73
7.31
7.30
7.26
4.59
2.76
2.76
2.75
2.43
2.15
2.14
2.12
2.11
2.09
2.07
2.04
1.35
1.34
1.33
0.92
0.92
2.00
2.15
2.06
2.00
3.00
4.03
1.07
4.00
3.00
13.0
12.5
12.0
11.5
11.0
10.5
10.0
9.5
9.0
8.5
8.0
7.5
7.0
6.5
6.0
5.5
5.0
4.5
4.0
3.5
3.0
2.5
2.0
1.5
1.0
0.5
0.0

$^{13}C\{^1H\}$ NMR (151 MHz, CDCl$_3$)

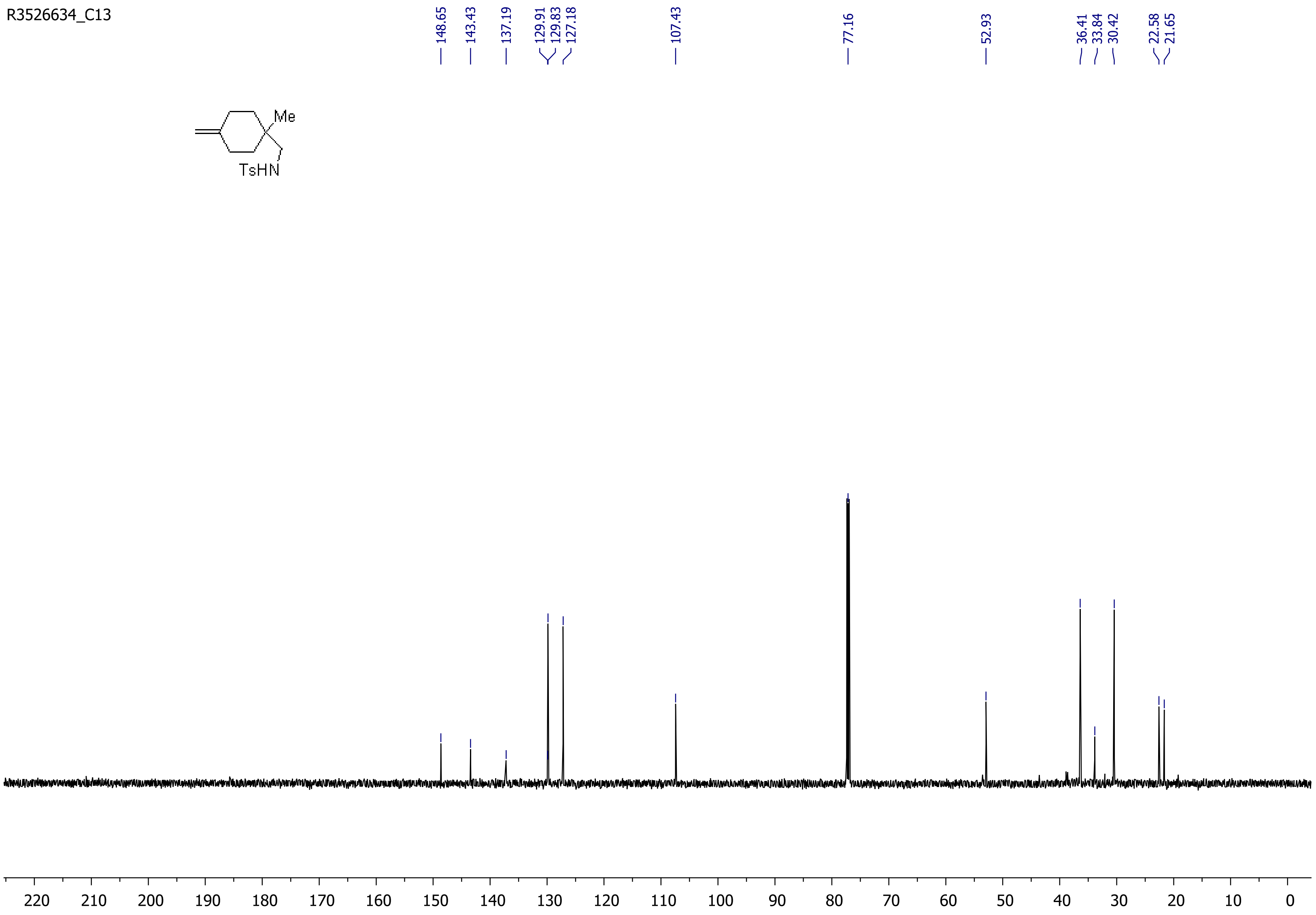

Compound 43

^{1}H NMR (500 MHz, $CDCl_3$)

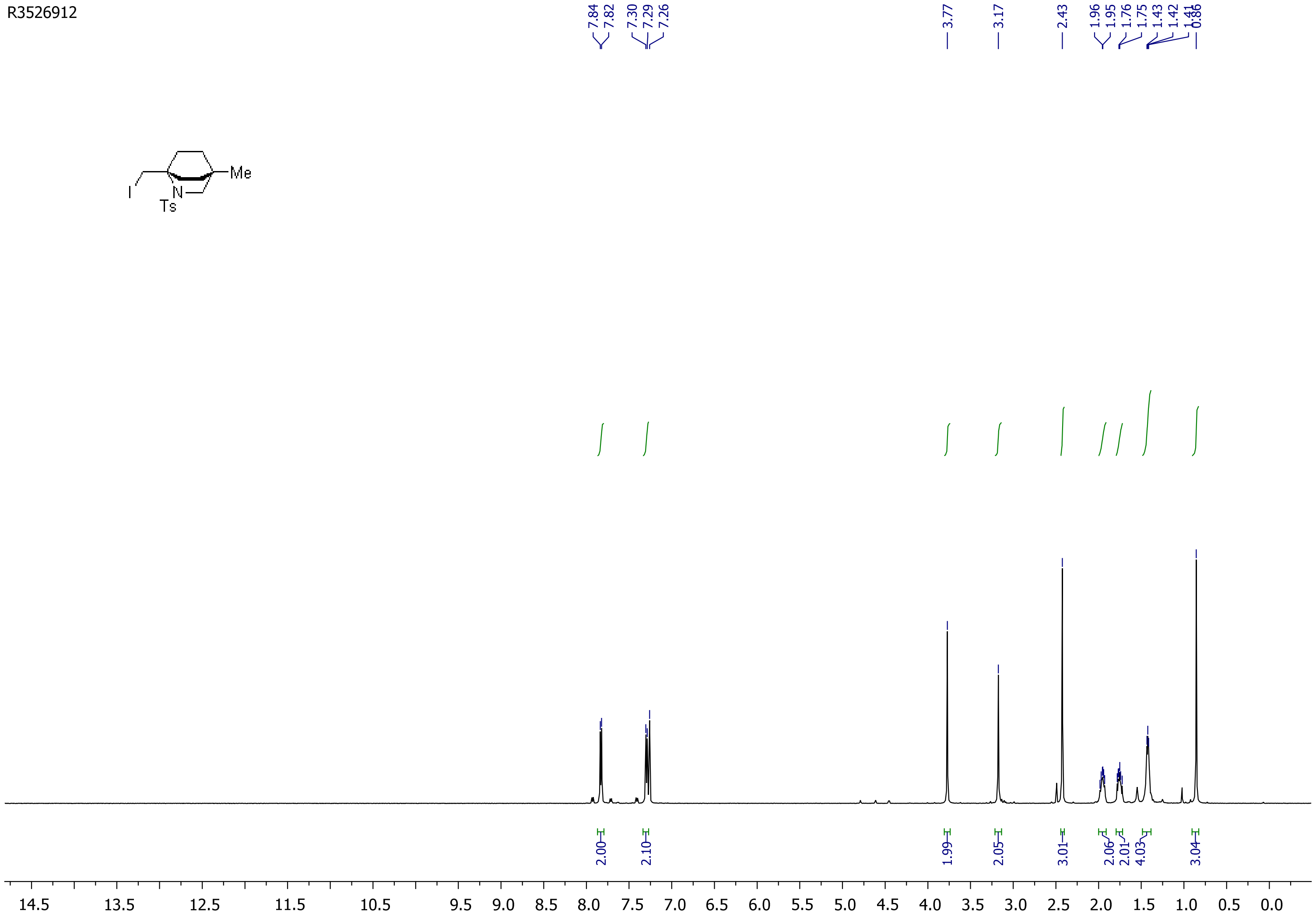

$^{13}C\{^{1}H\}$ NMR (151 MHz, $CDCl_3$)

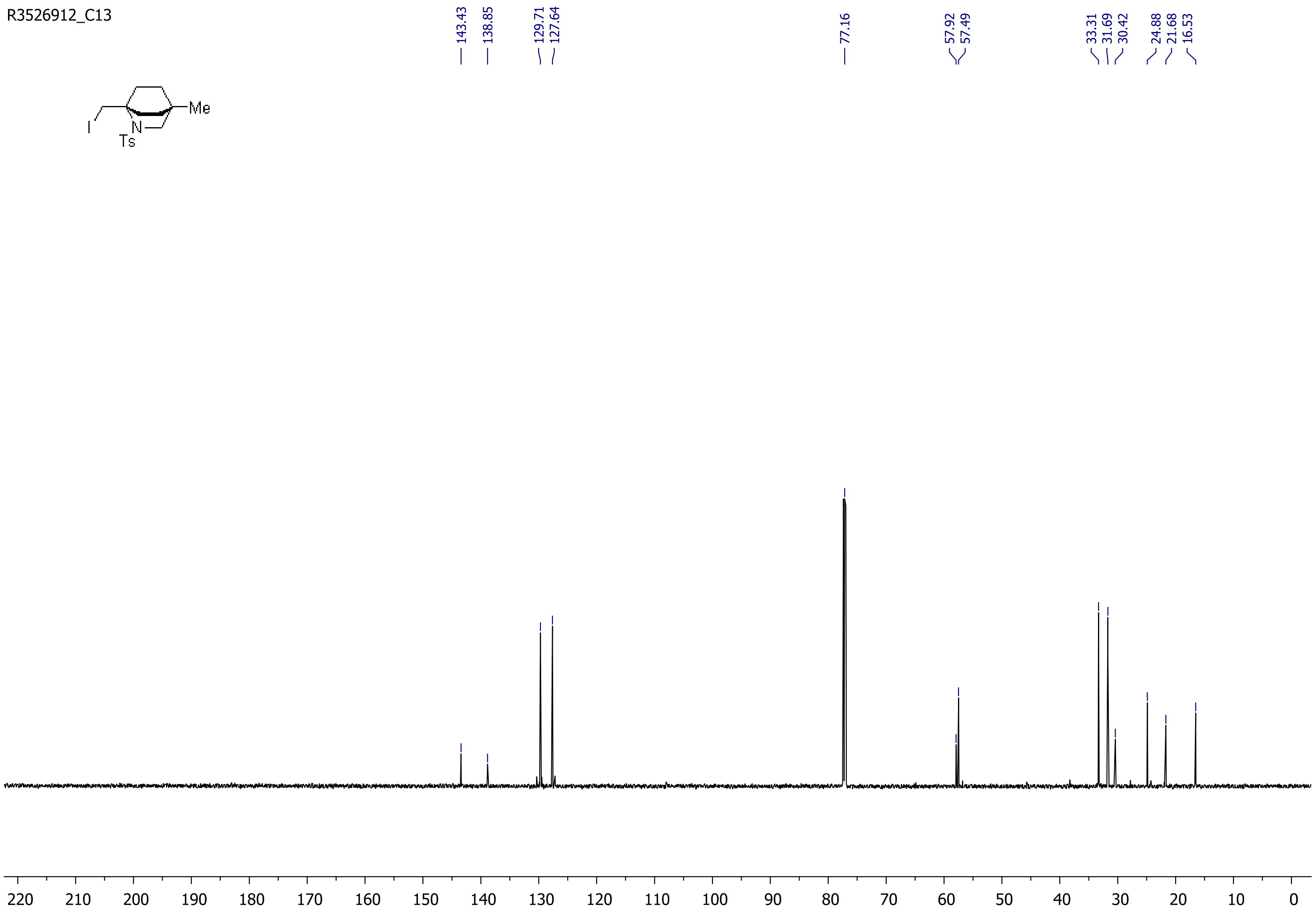

Compound 44

^{1}H NMR (500 MHz, $CDCl_3$)

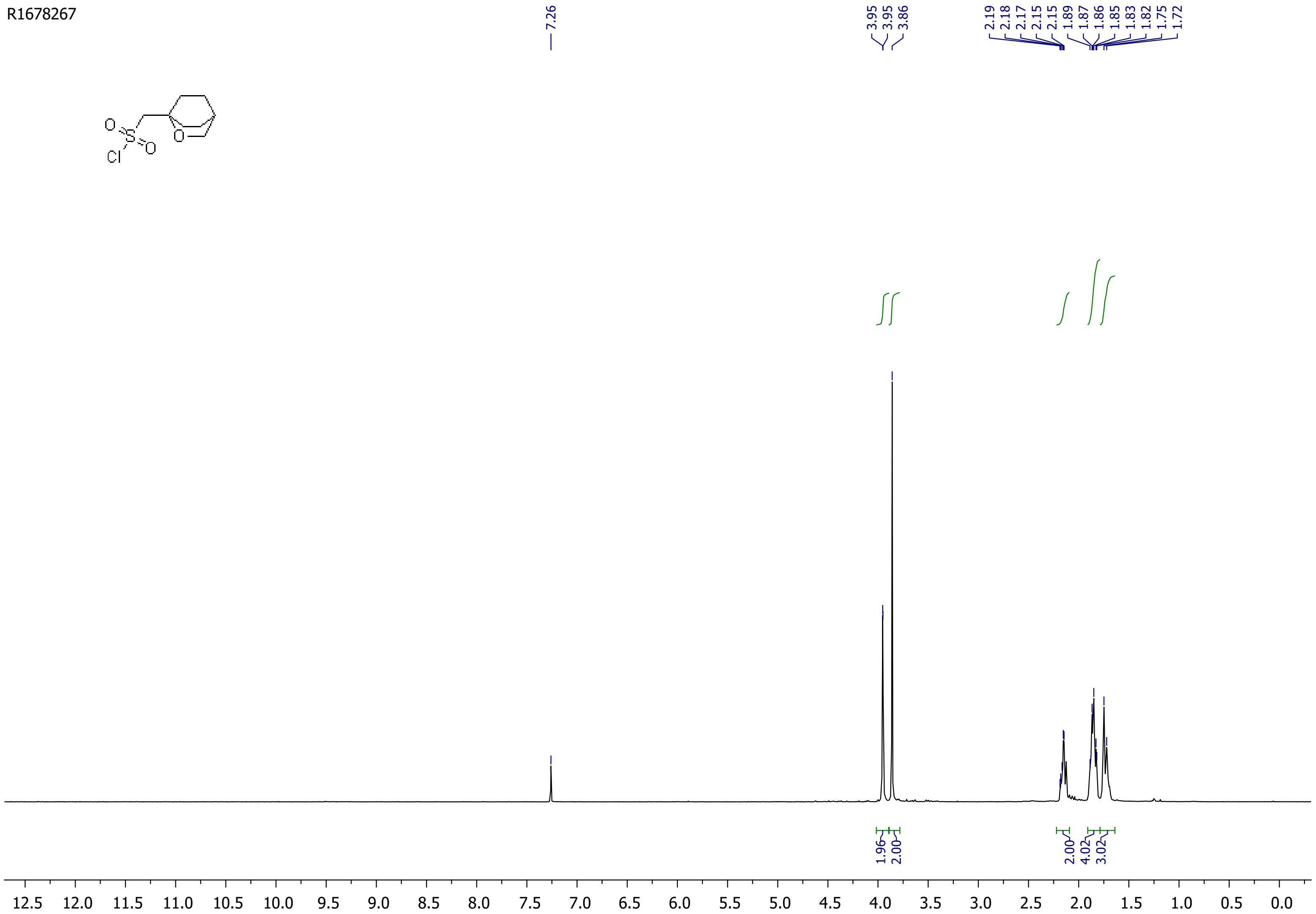

$^{13}C\{^{1}H\}$ NMR (126 MHz, $CDCl_3$)

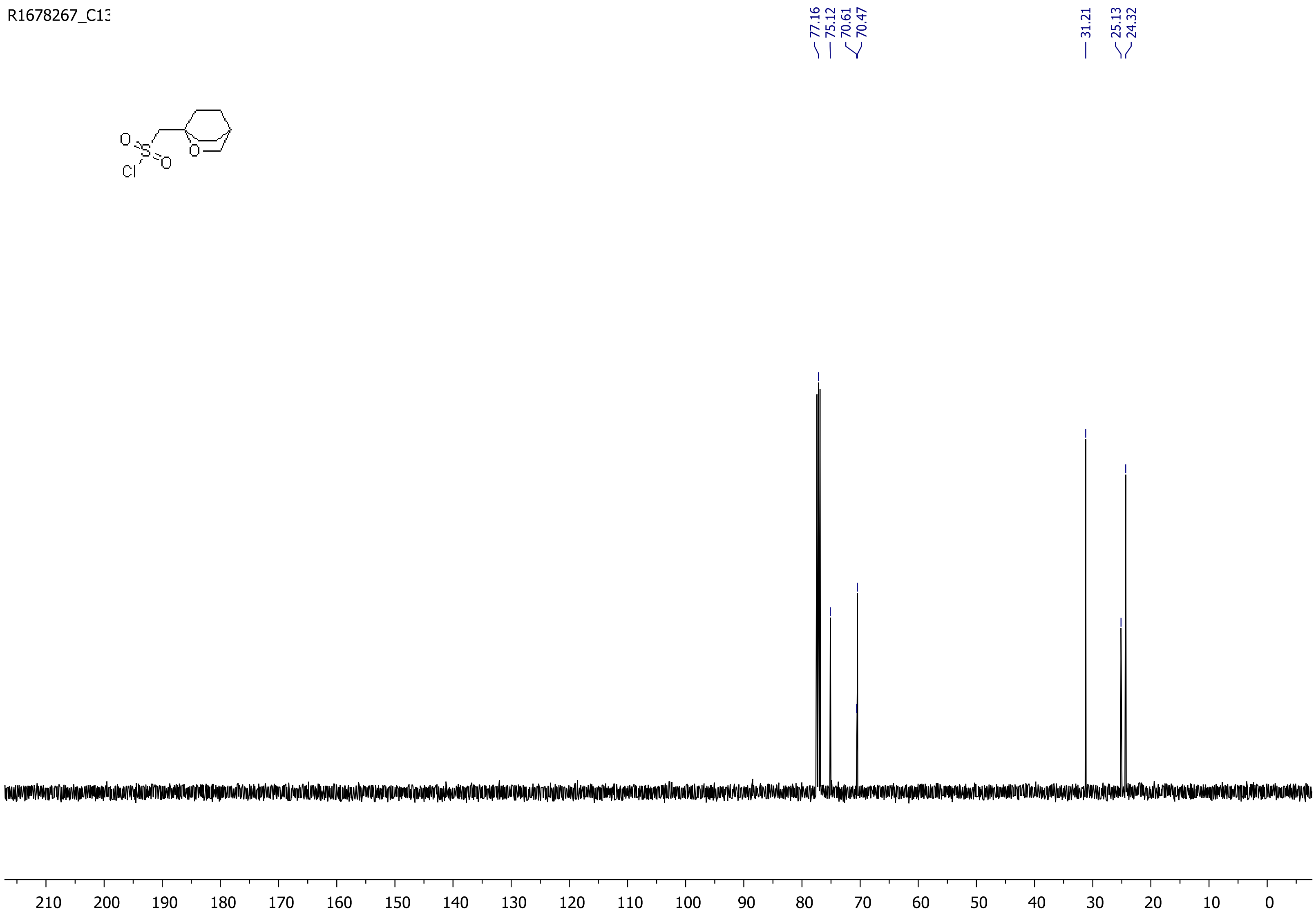

Compound 45

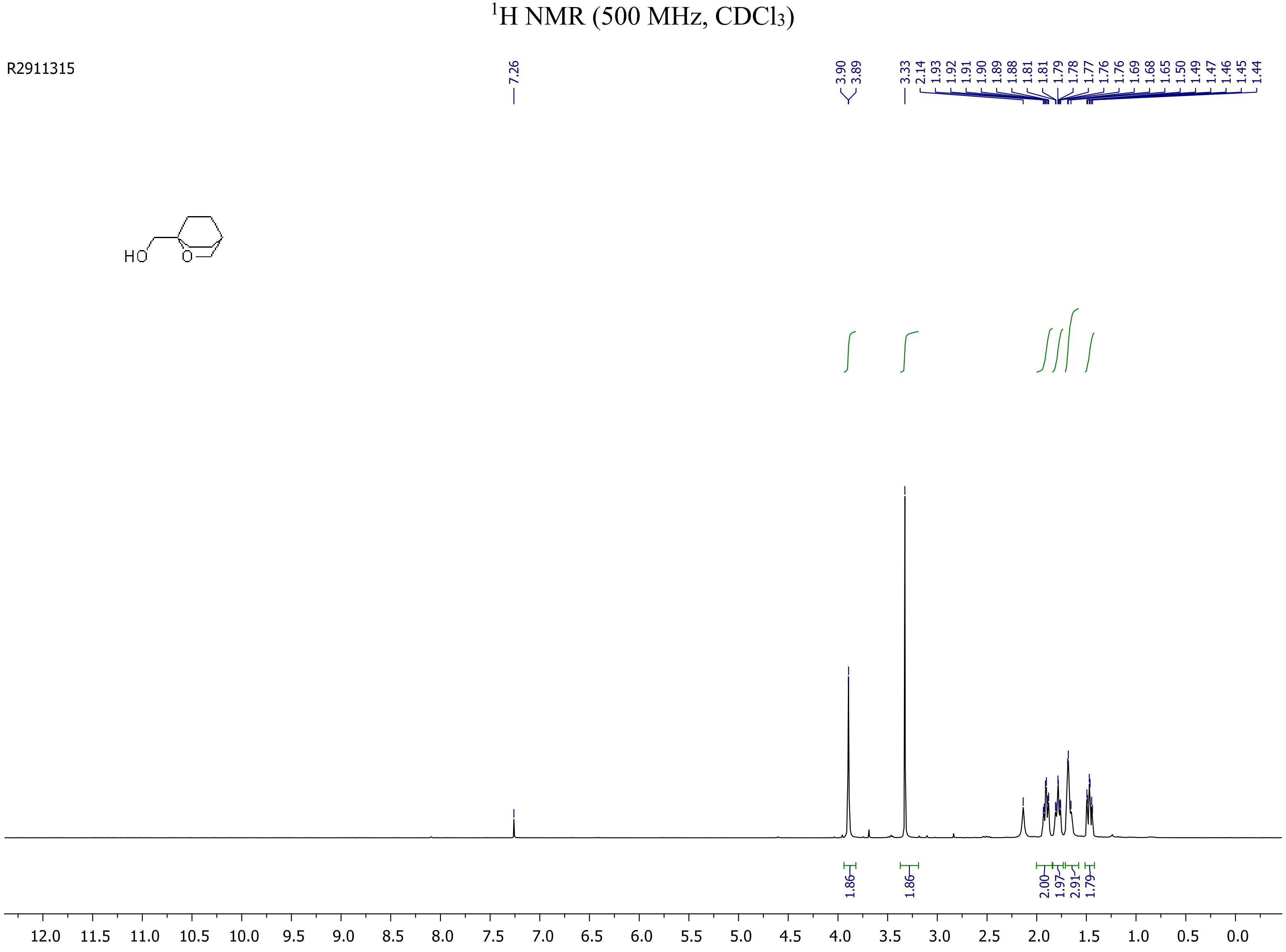

^{1}H NMR (500 MHz, $CDCl_3$)
R2911315
7.26
3.90
3.89
3.33
2.14
1.93
1.92
1.91
1.90
1.89
1.88
1.81
1.81
1.79
1.78
1.77
1.76
1.76
1.69
1.68
1.65
1.50
1.49
1.47
1.46
1.45
1.44
HO
O
1.86
1.86
2.00
1.97
2.91
1.79
12.0 11.5 11.0 10.5 10.0 9.5 9.0 8.5 8.0 7.5 7.0 6.5 6.0 5.5 5.0 4.5 4.0 3.5 3.0 2.5 2.0 1.5 1.0 0.5 0.0

$^{13}C\{^{1}H\}$ NMR (126 MHz, $CDCl_3$)

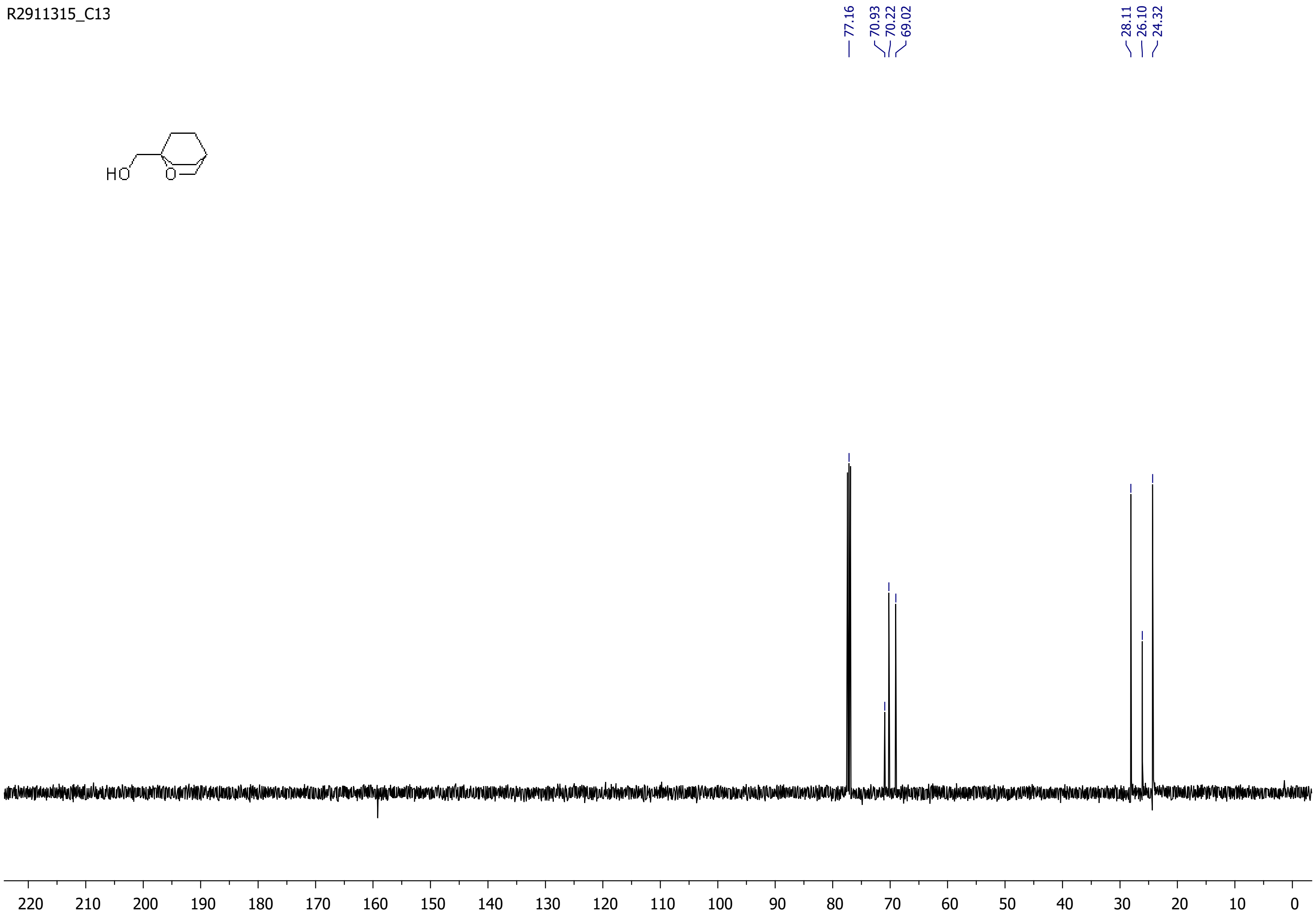

Compound 46

^{1}H NMR (400 MHz, $CDCl_3$)

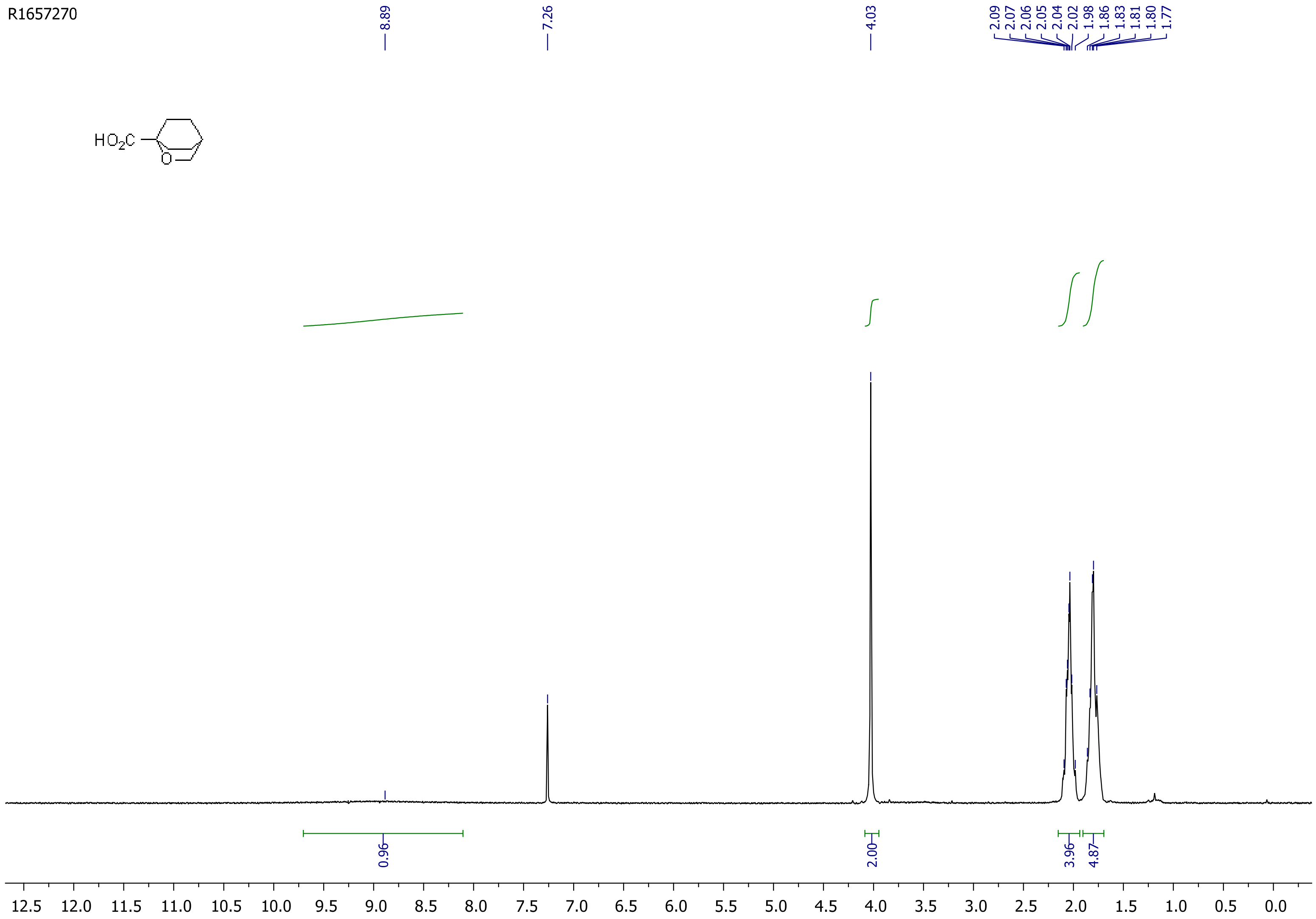

$^{13}C\{^1H\}$ NMR (126 MHz, $CDCl_3$)

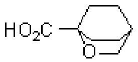

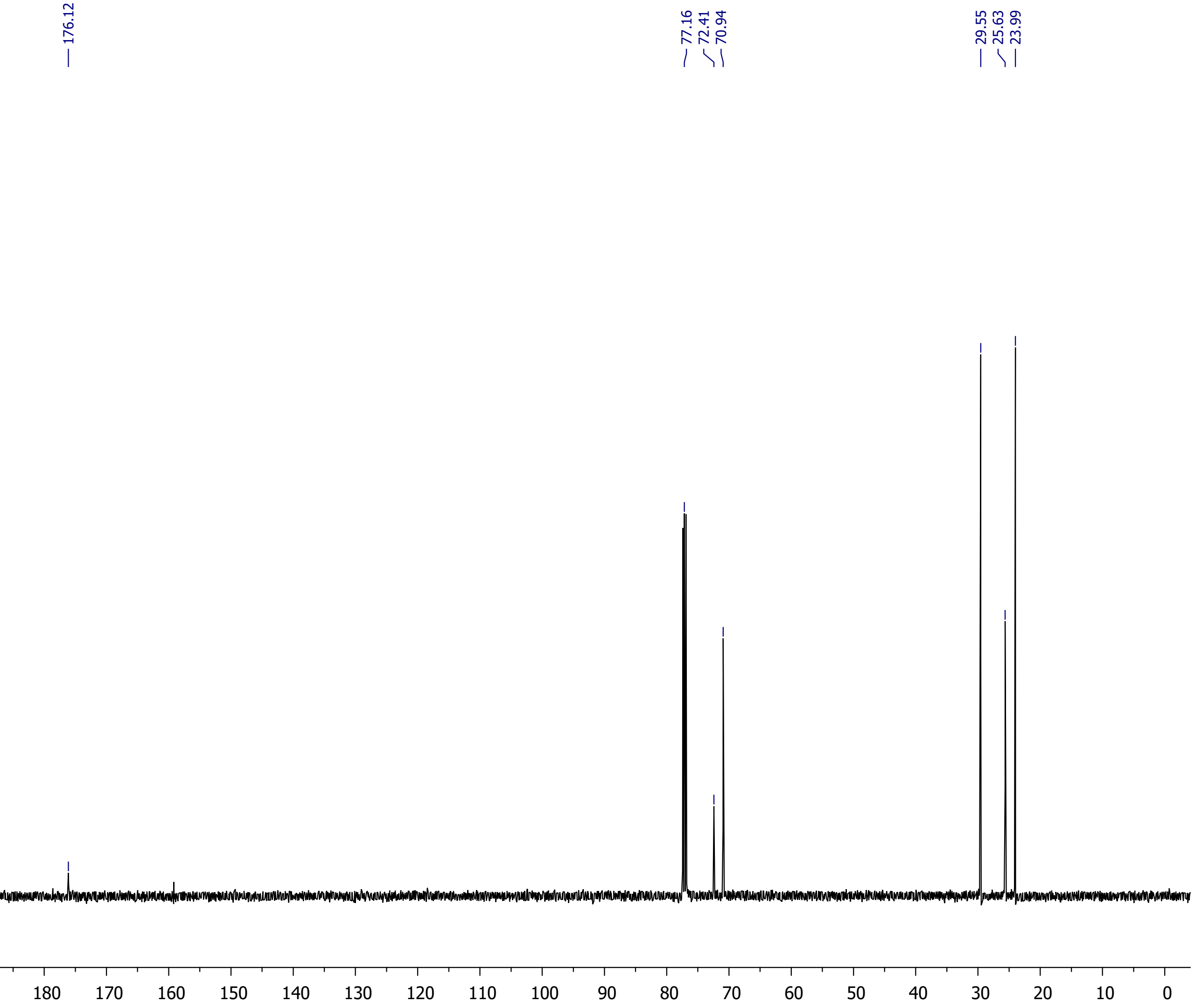

Compound SI-15

^{1}H NMR (500 MHz, $CDCl_3$)

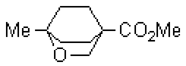

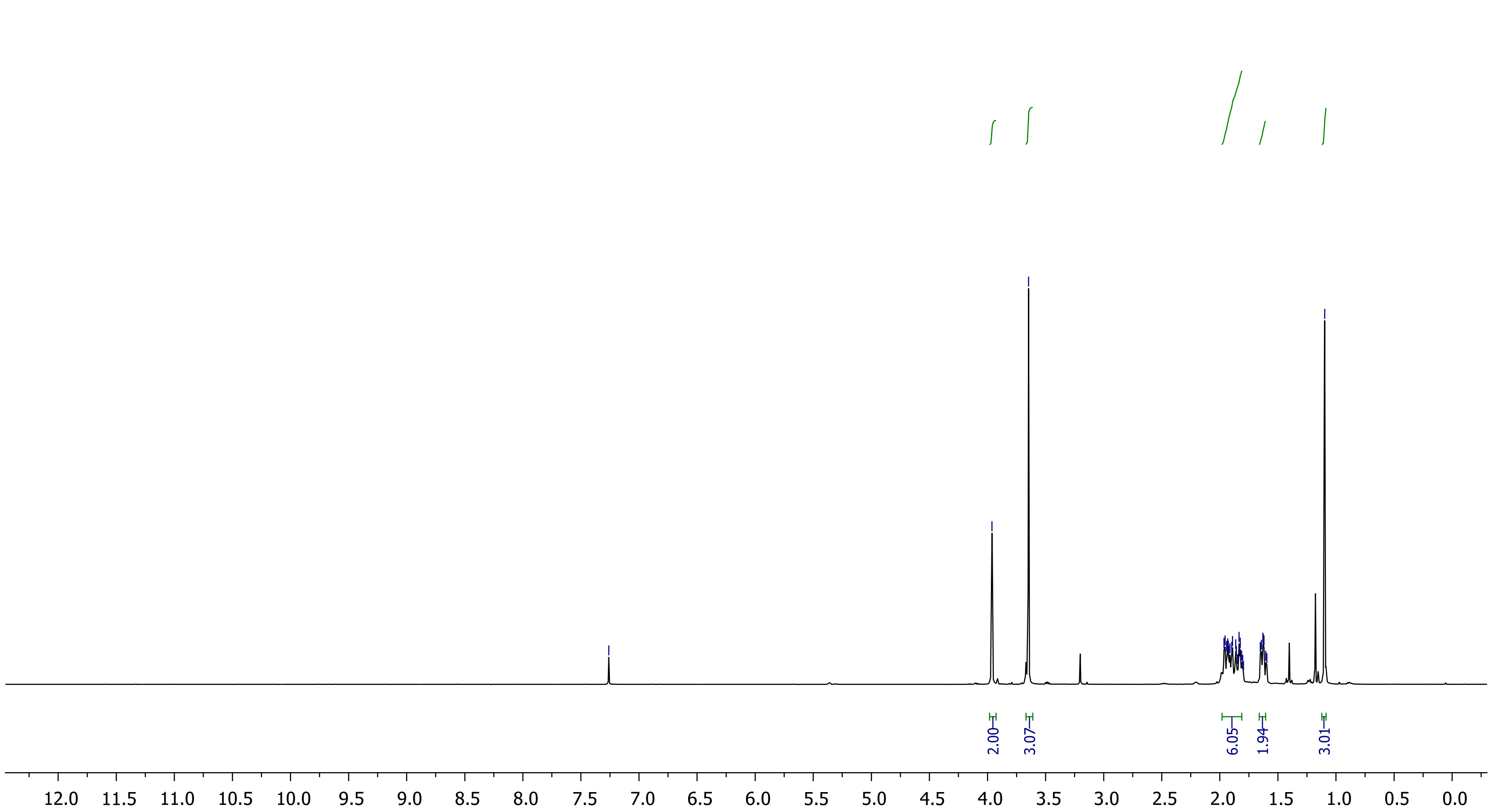

$^{13}C\{^1H\}$ NMR (151 MHz, $CDCl_3$)

R3205808_C13

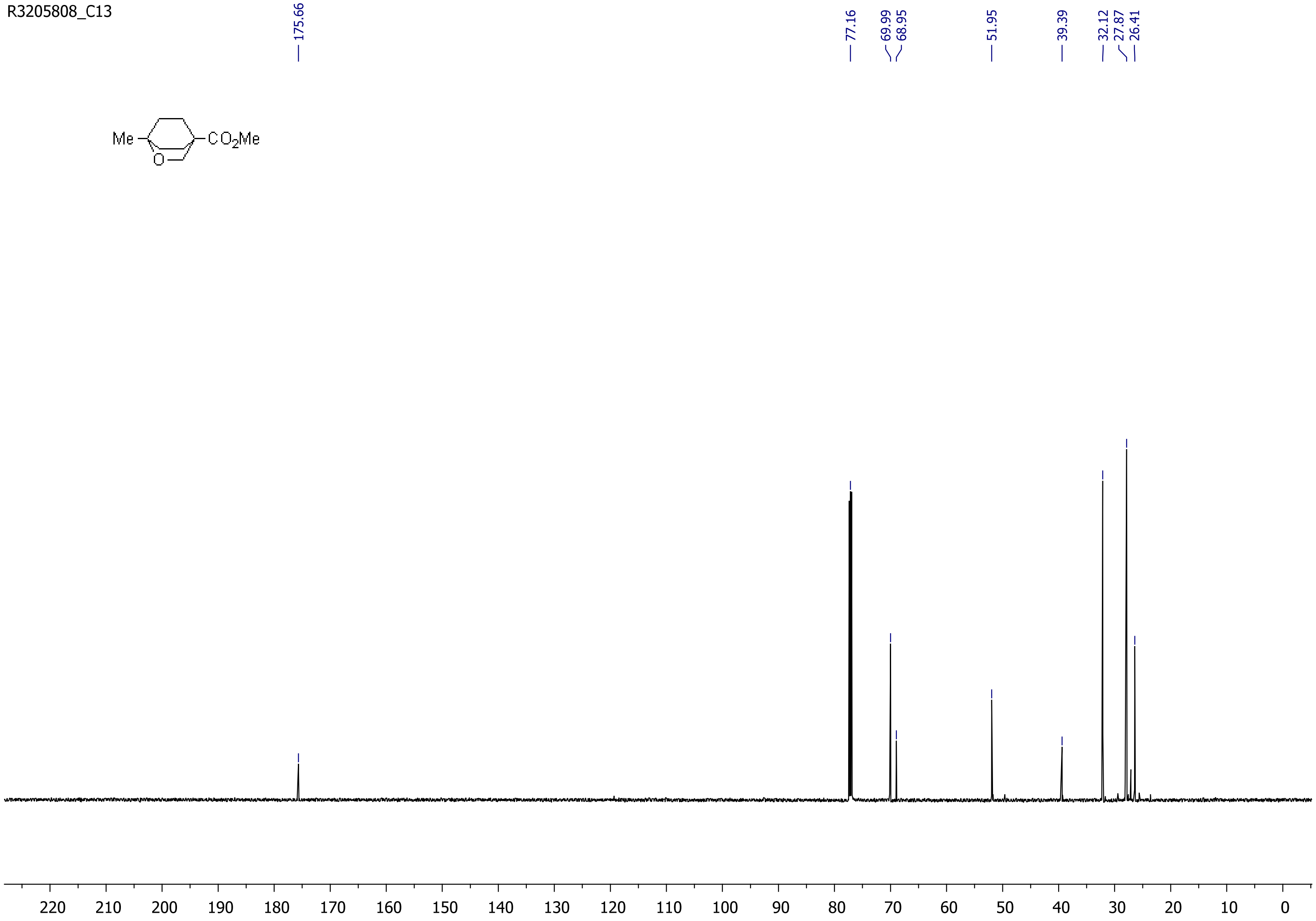

Compound 47

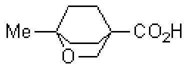

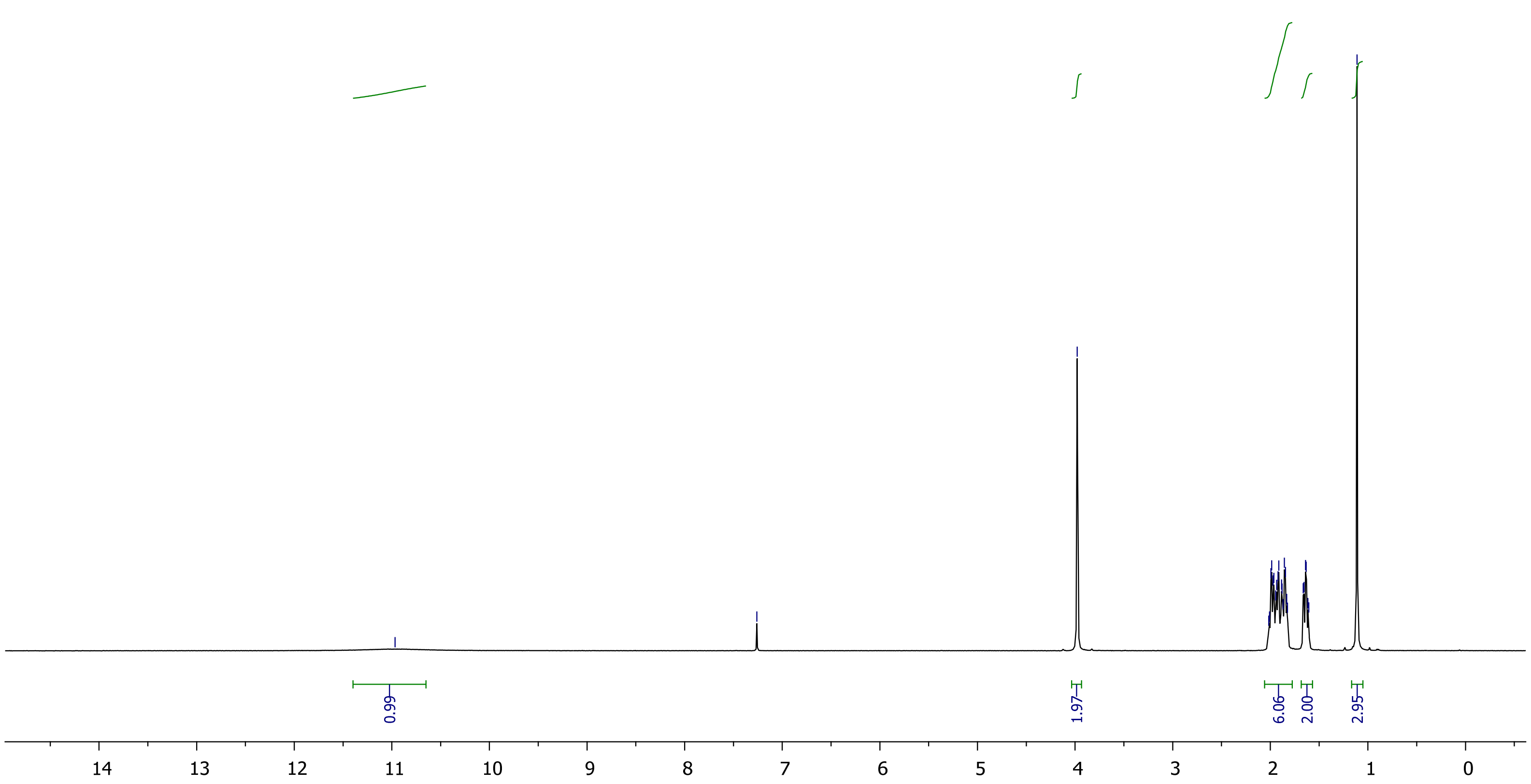

^{1}H NMR (500 MHz, $CDCl_3$)
R2840881
10.97
7.26
3.98
1.99
1.99
1.97
1.96
1.94
1.92
1.91
1.88
1.86
1.85
1.66
1.65
1.64
1.63
1.11
0.99
1.97
6.06
2.00
2.95
14
13
12
11
10
9
8
7
6
5
4
3
2
1
0

$^{13}C\{^1H\}$ NMR (126 MHz, $CDCl_3$)

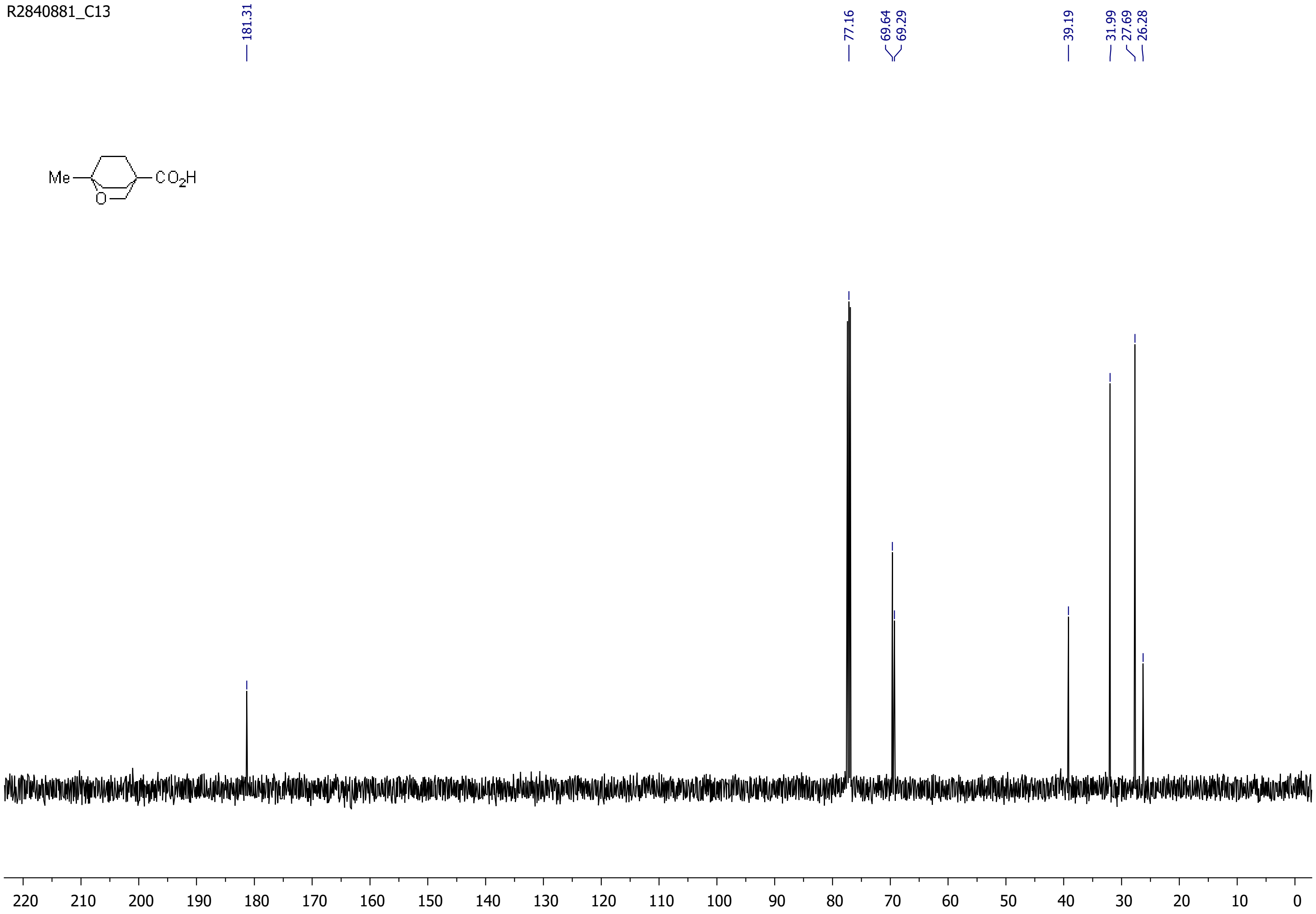

Compound 48

^{1}H NMR (500 MHz, DMSO-d$_6$)

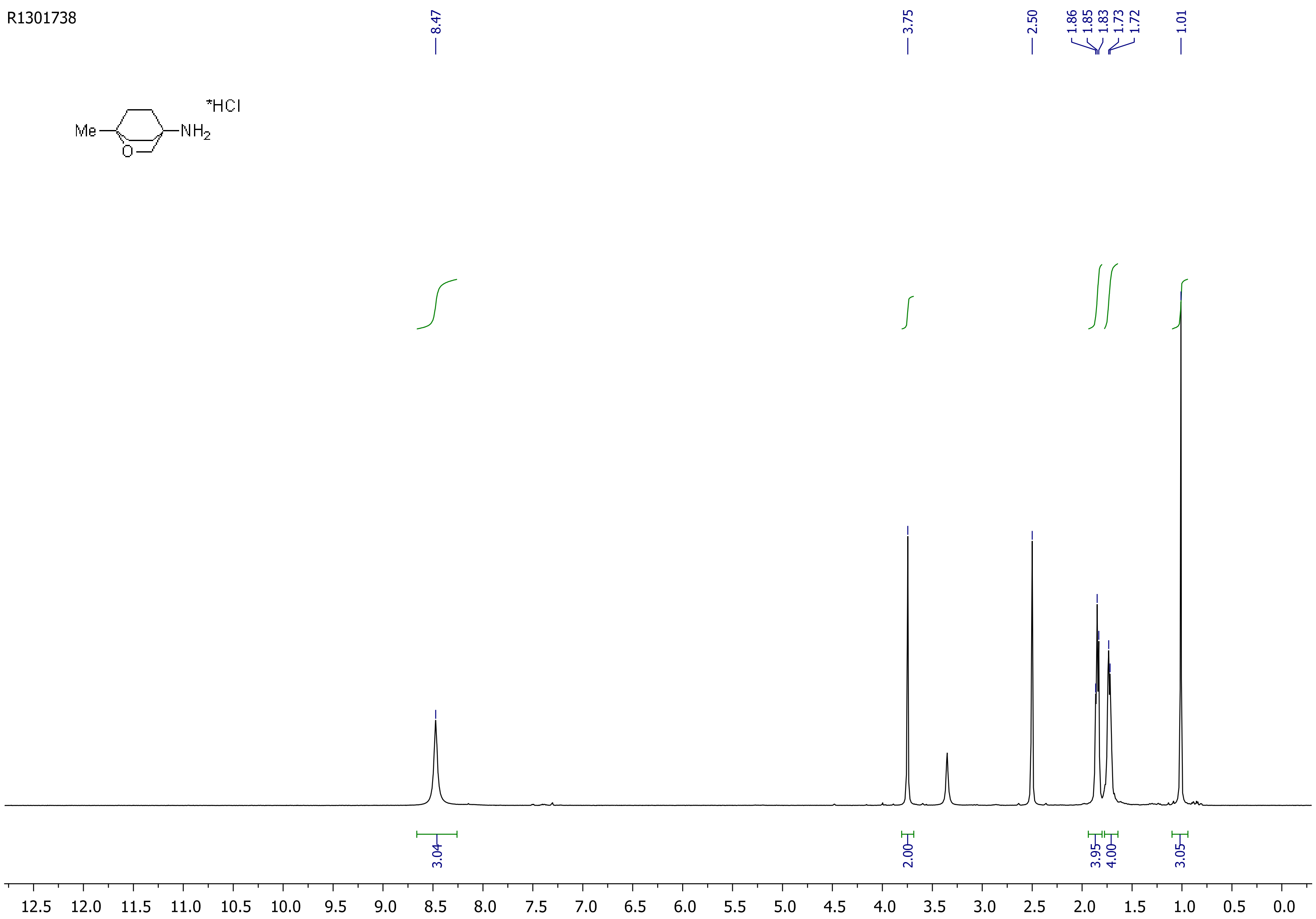

$^{13}C\{^1H\}$ NMR (126 MHz, DMSO-d6)

R1301738_C13

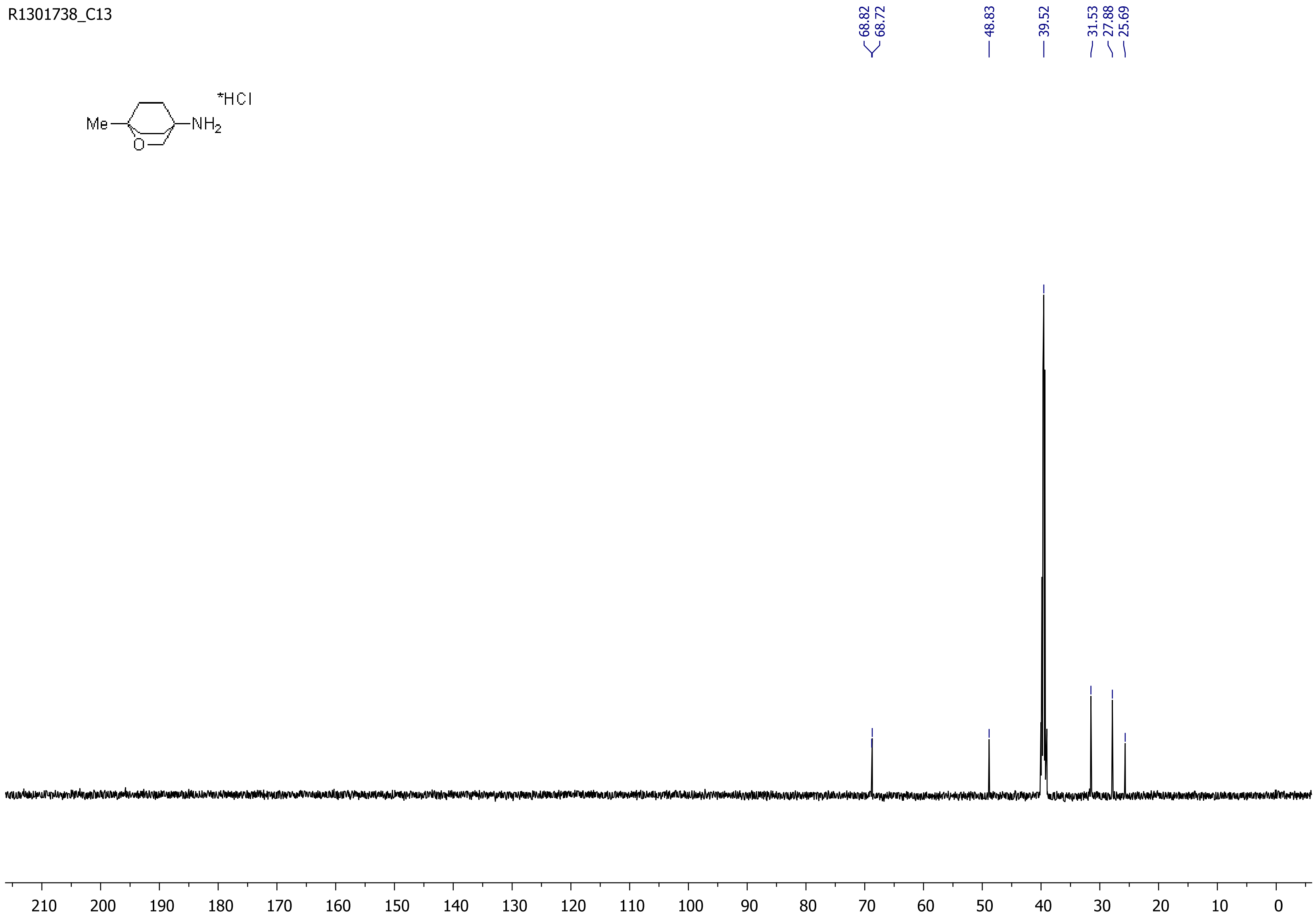

Compound 49

^{1}H NMR (500 MHz, $CDCl_3$)

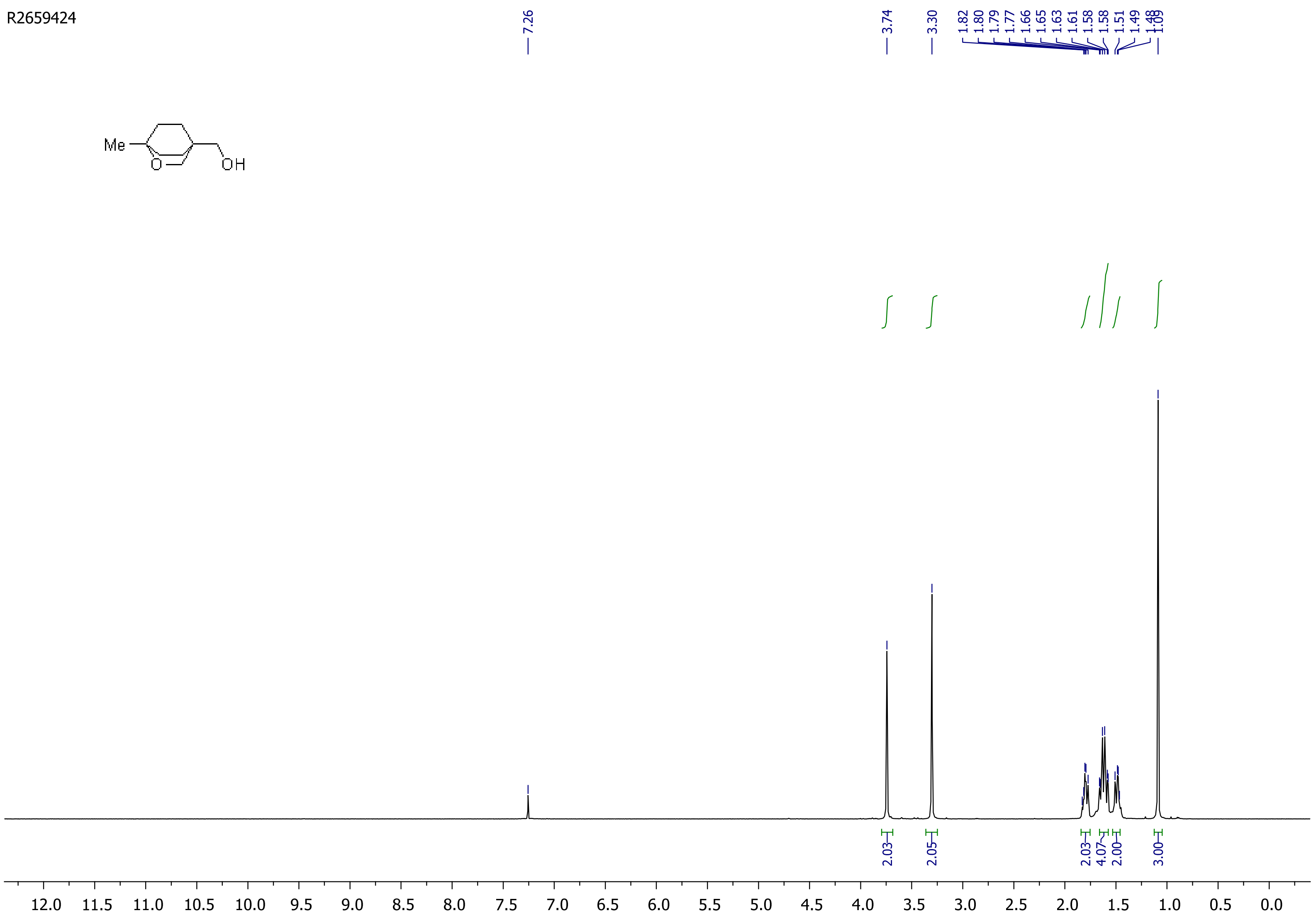

$^{13}C\{^1H\}$ NMR (126 MHz, $CDCl_3$)

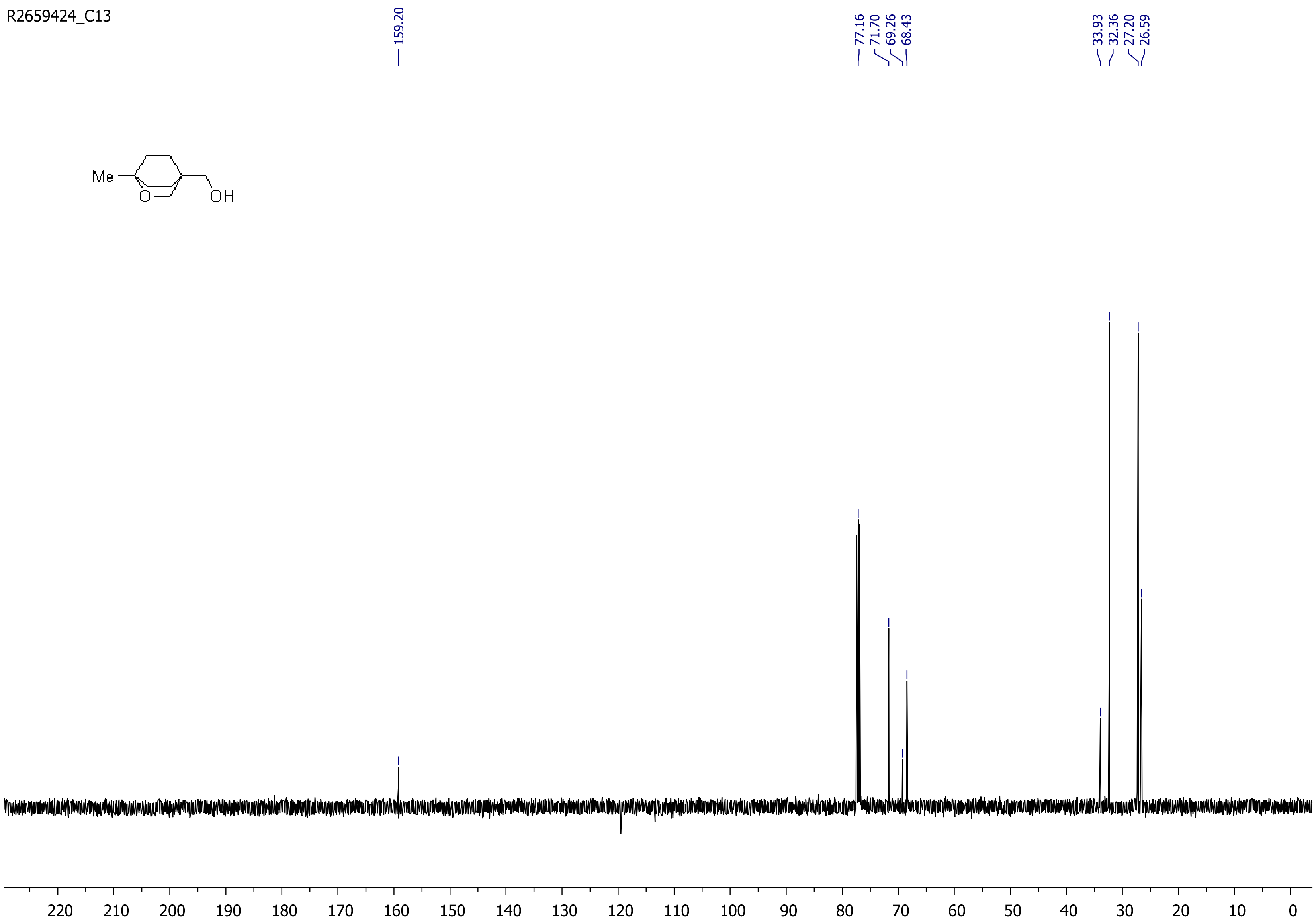

Compound 50

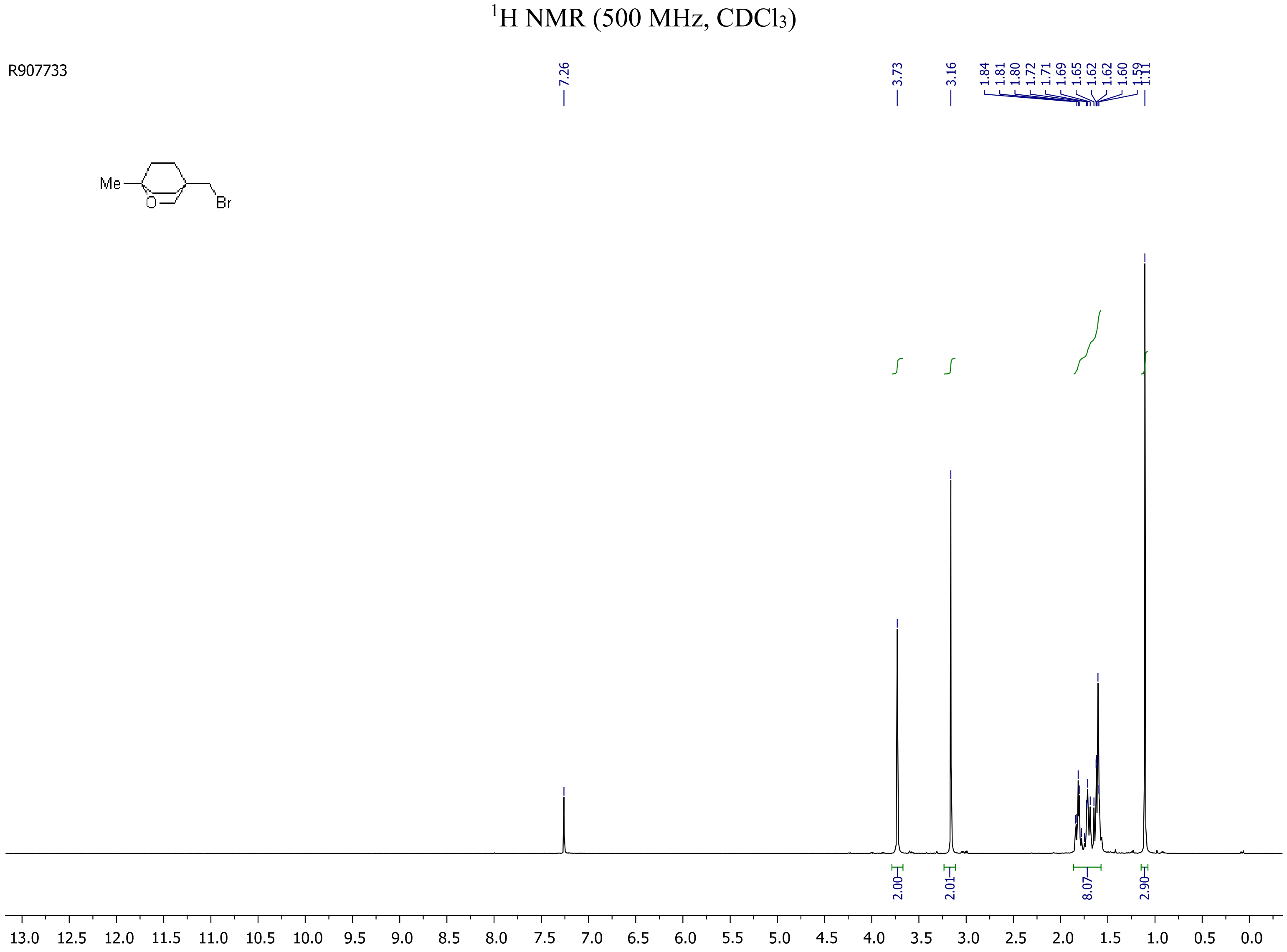

¹H NMR (500 MHz, CDCl3)
R907733
Me
O
Br
7.26
3.73
3.16
1.84
1.81
1.80
1.72
1.71
1.69
1.65
1.62
1.62
1.60
1.59
1.11
2.00
2.01
8.07
2.90
13.0
12.5
12.0
11.5
11.0
10.5
10.0
9.5
9.0
8.5
8.0
7.5
7.0
6.5
6.0
5.5
5.0
4.5
4.0
3.5
3.0
2.5
2.0
1.5
1.0
0.5
0.0

$^{13}C\{^{1}H\}$ NMR (126 MHz, $CDCl_3$)

R907733_C13

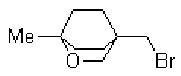

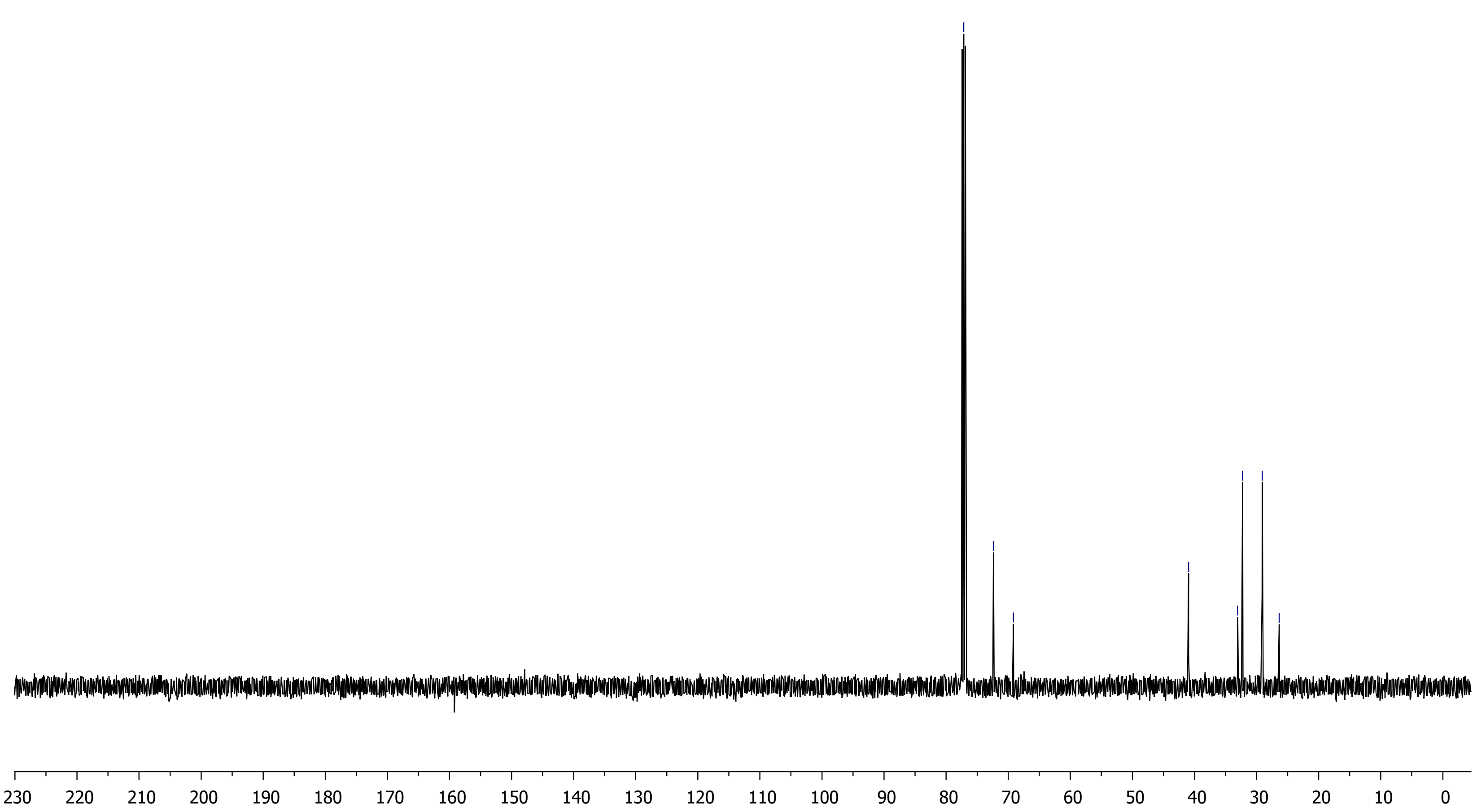

Compound 51

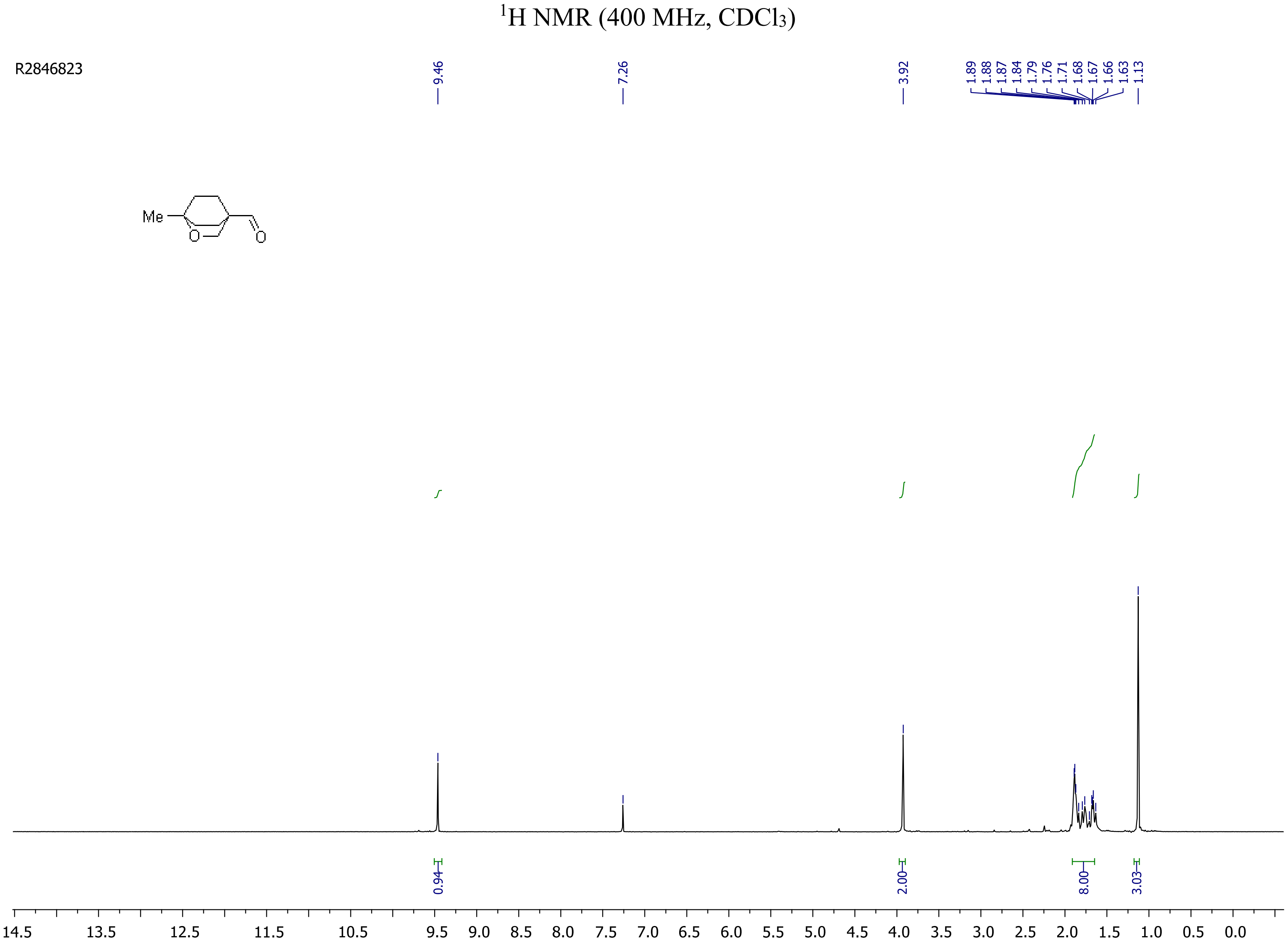
^{1}H NMR (400 MHz, CDCl$_3$)
R2846823
Me
O
O
9.46
7.26
3.92
1.89
1.88
1.87
1.84
1.79
1.76
1.71
1.68
1.67
1.66
1.63
1.13
0.94
2.00
8.00
3.03
14.5
13.5
12.5
11.5
10.5
9.5
9.0
8.5
8.0
7.5
7.0
6.5
6.0
5.5
5.0
4.5
4.0
3.5
3.0
2.5
2.0
1.5
1.0
0.5
0.0

$^{13}C\{^1H\}$ NMR (126 MHz, $CDCl_3$)

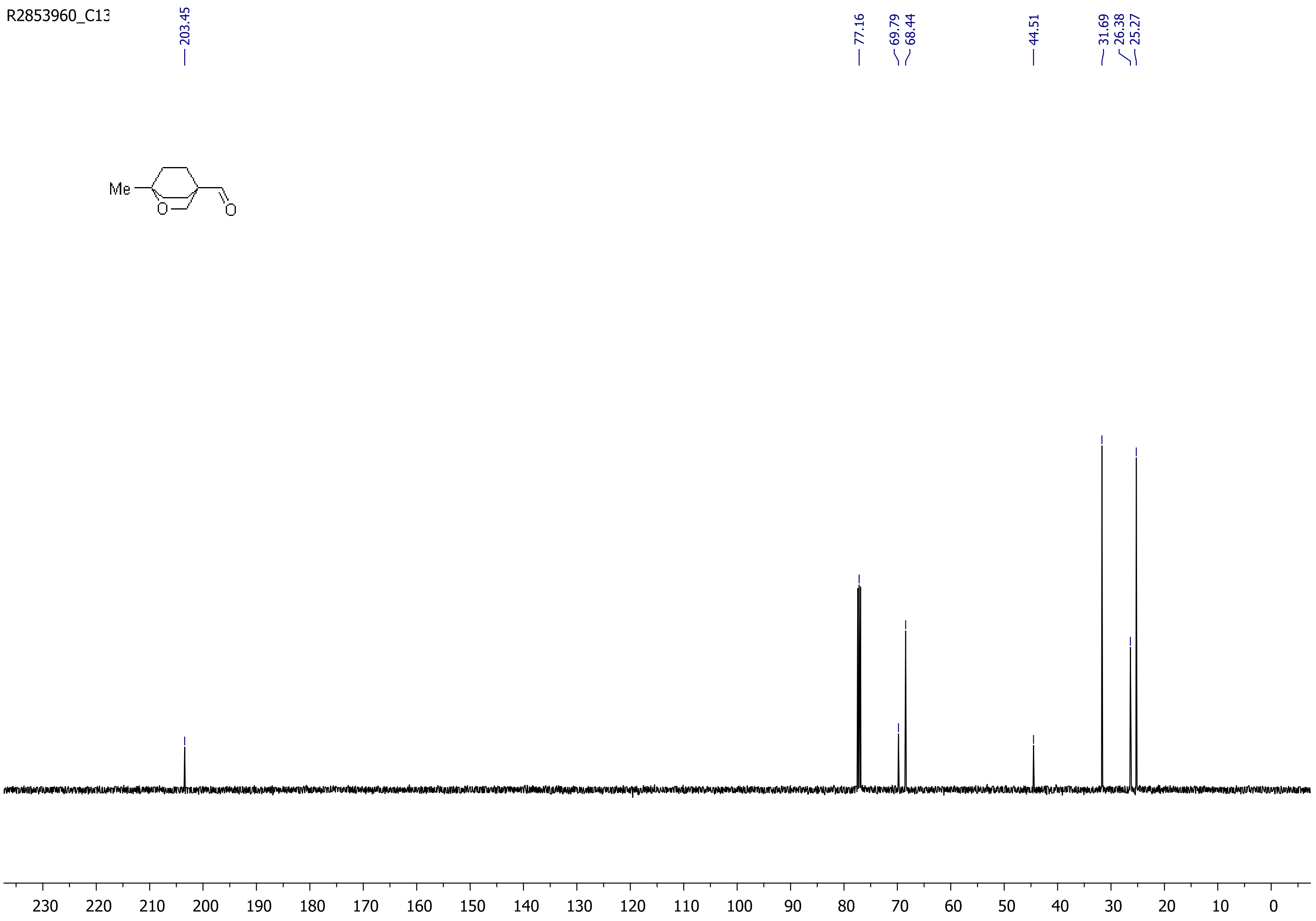

Compound SI-16

^{1}H NMR (500 MHz, $CDCl_3$)

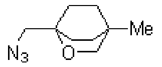

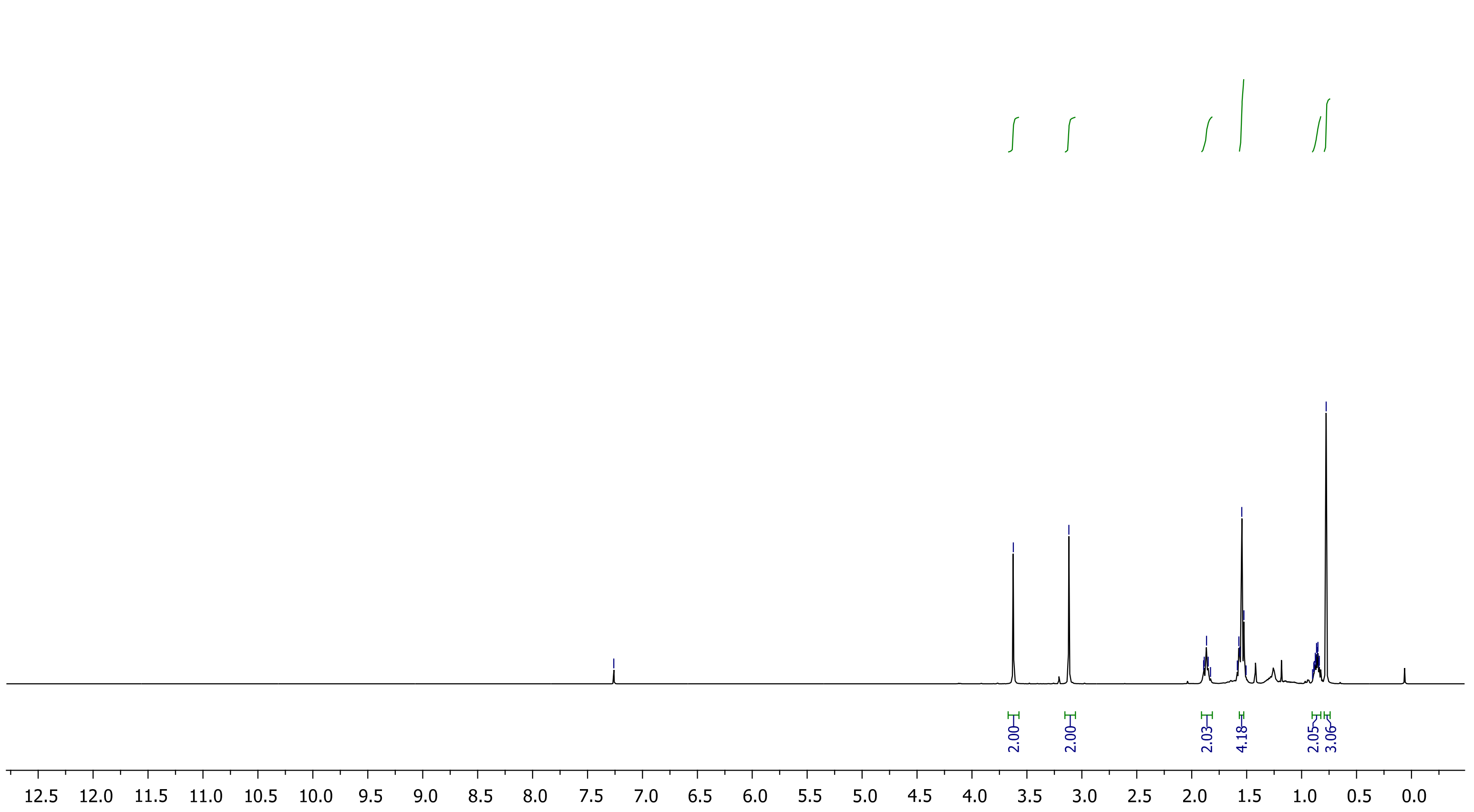

$^{13}C\{^{1}H\}$ NMR (126 MHz, $CDCl_3$)

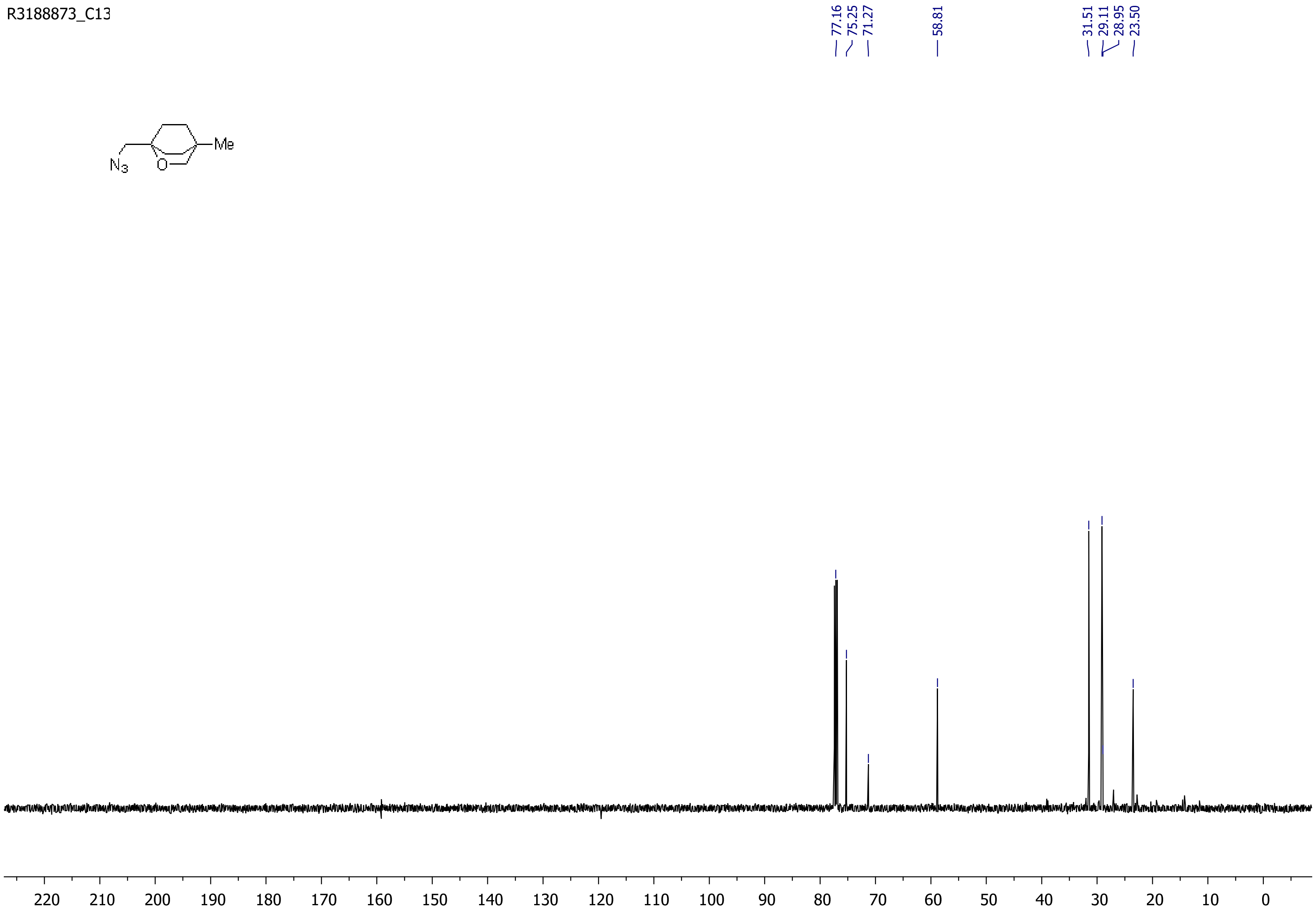

Compound 52

^{1}H NMR (500 MHz, DMSO-d_6)

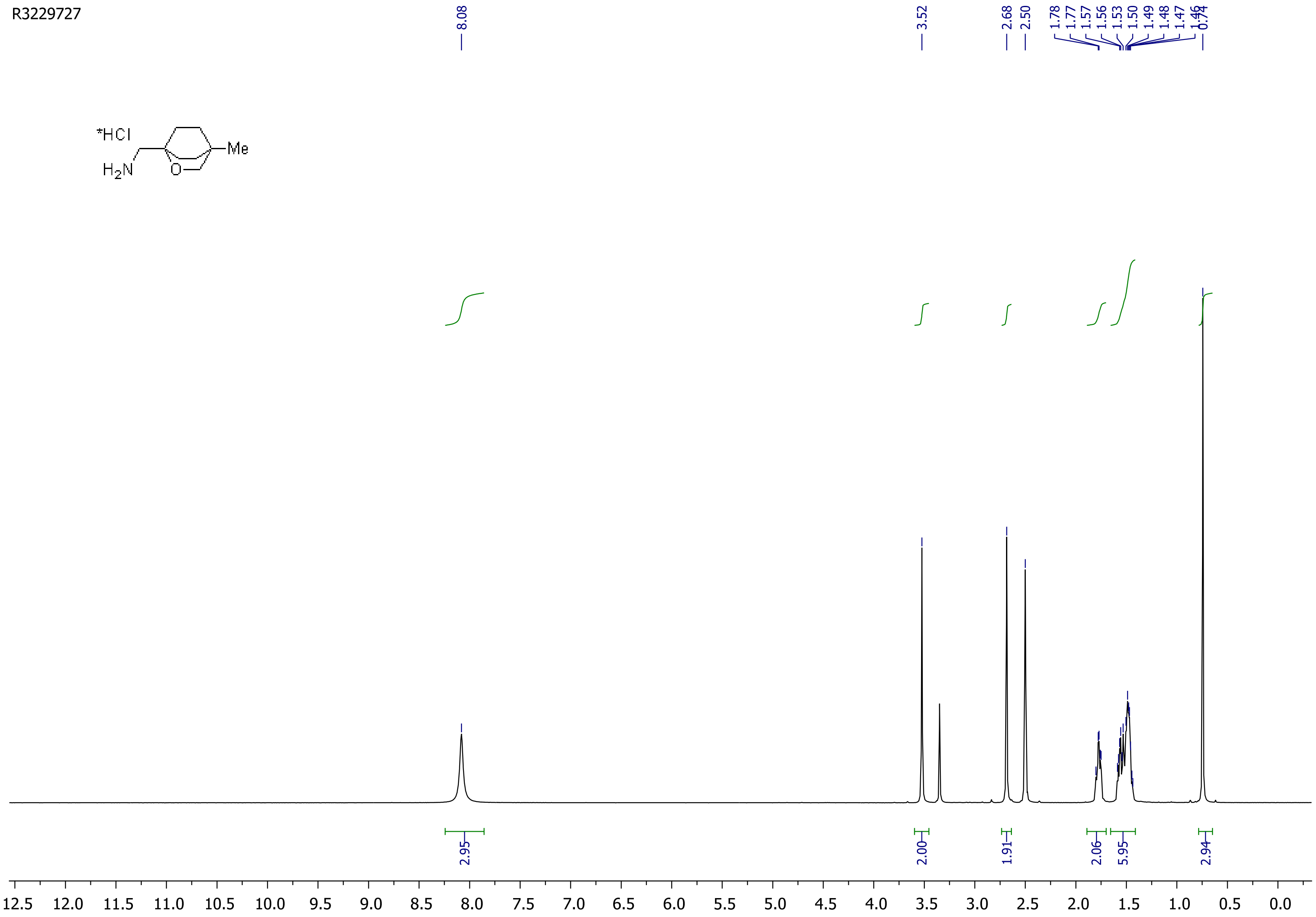

$^{13}C\{^1H\}$ NMR (126 MHz, DMSO-d6)

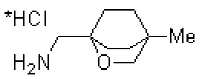

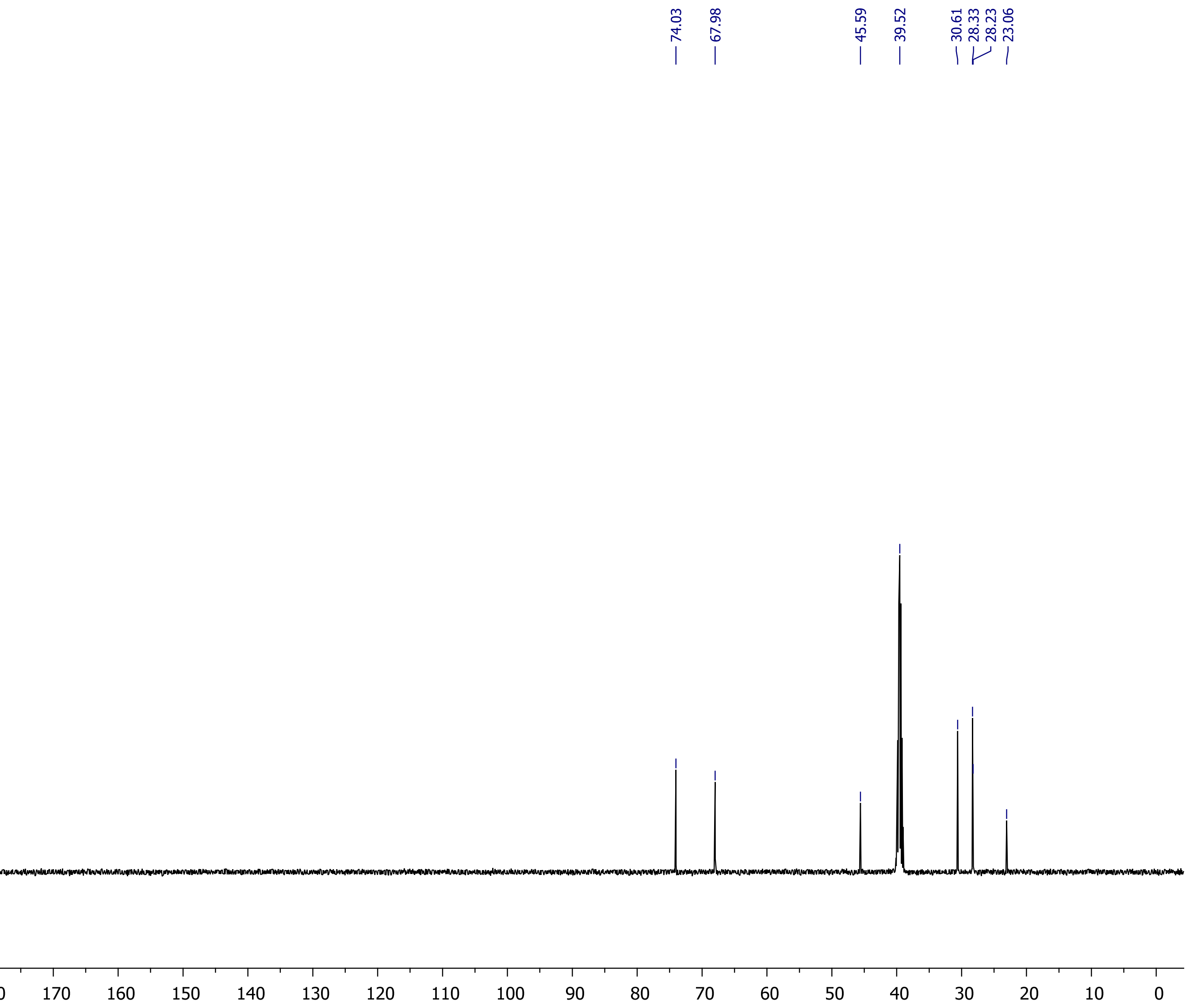

Compound SI-17

^{1}H NMR (500 MHz, $CDCl_3$)

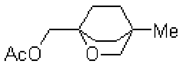

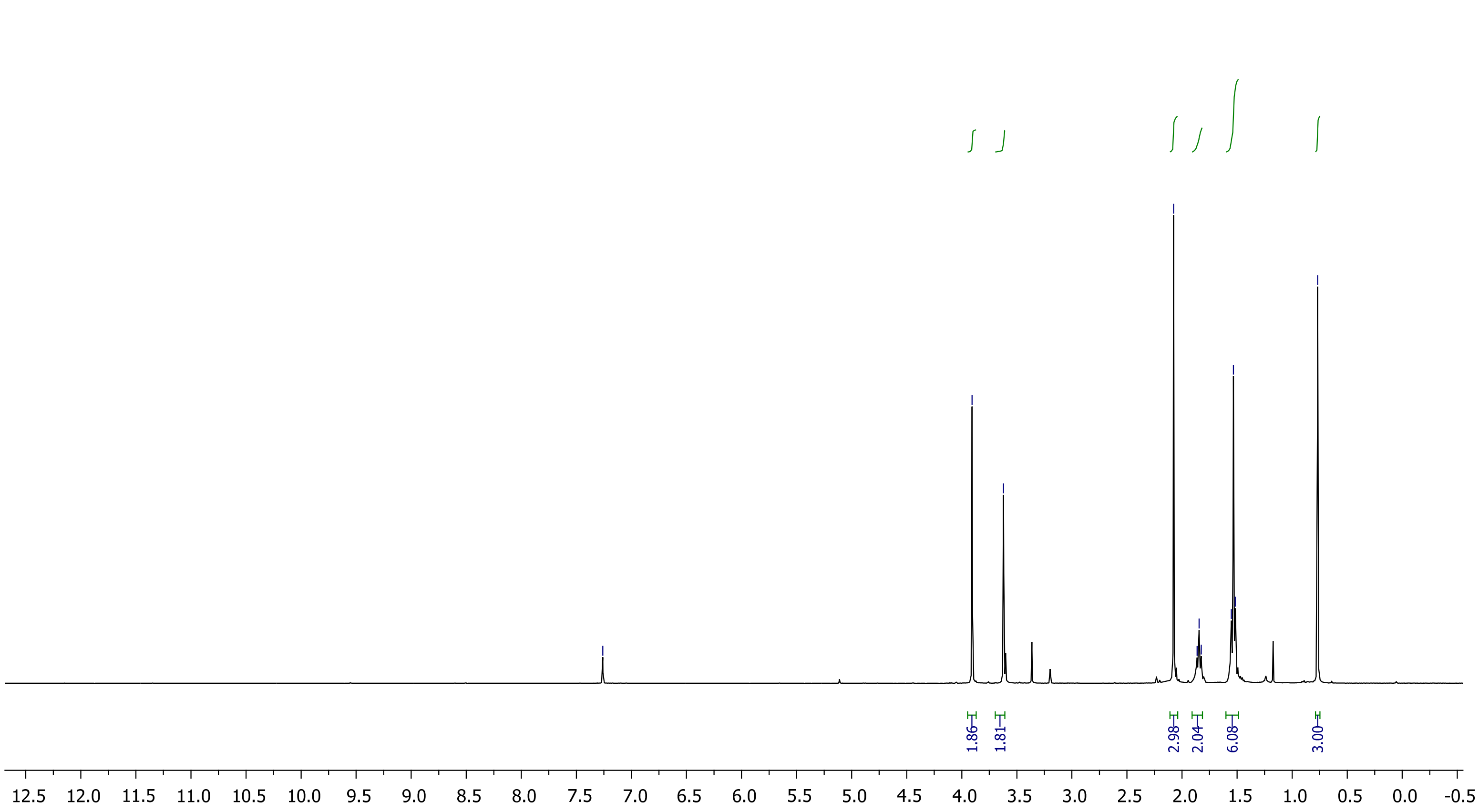

$^{13}C\{^{1}H\}$ NMR (151 MHz, $CDCl_3$)

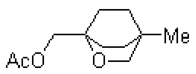

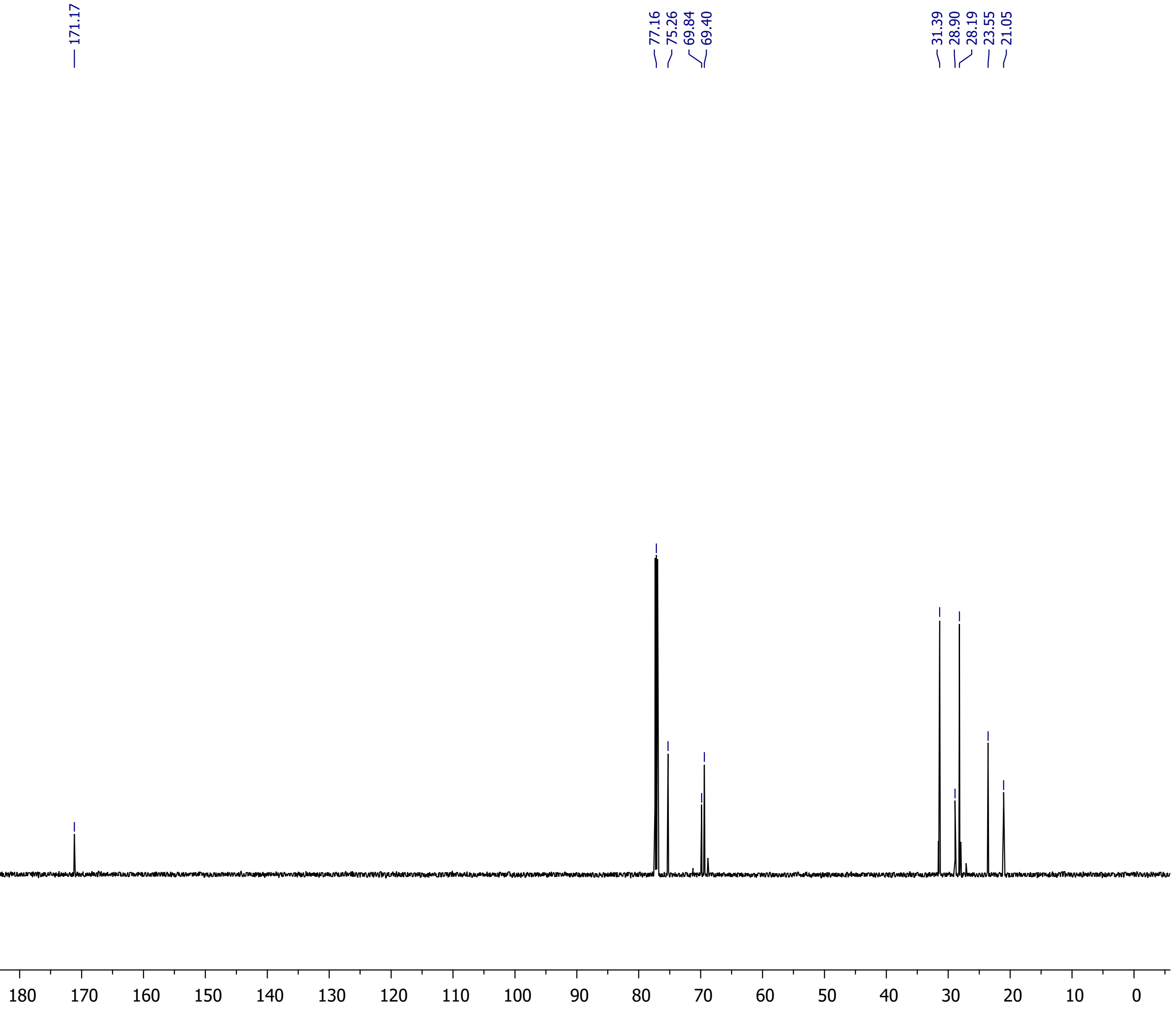

Compound 53

^{1}H NMR (500 MHz, CDCl$_3$)

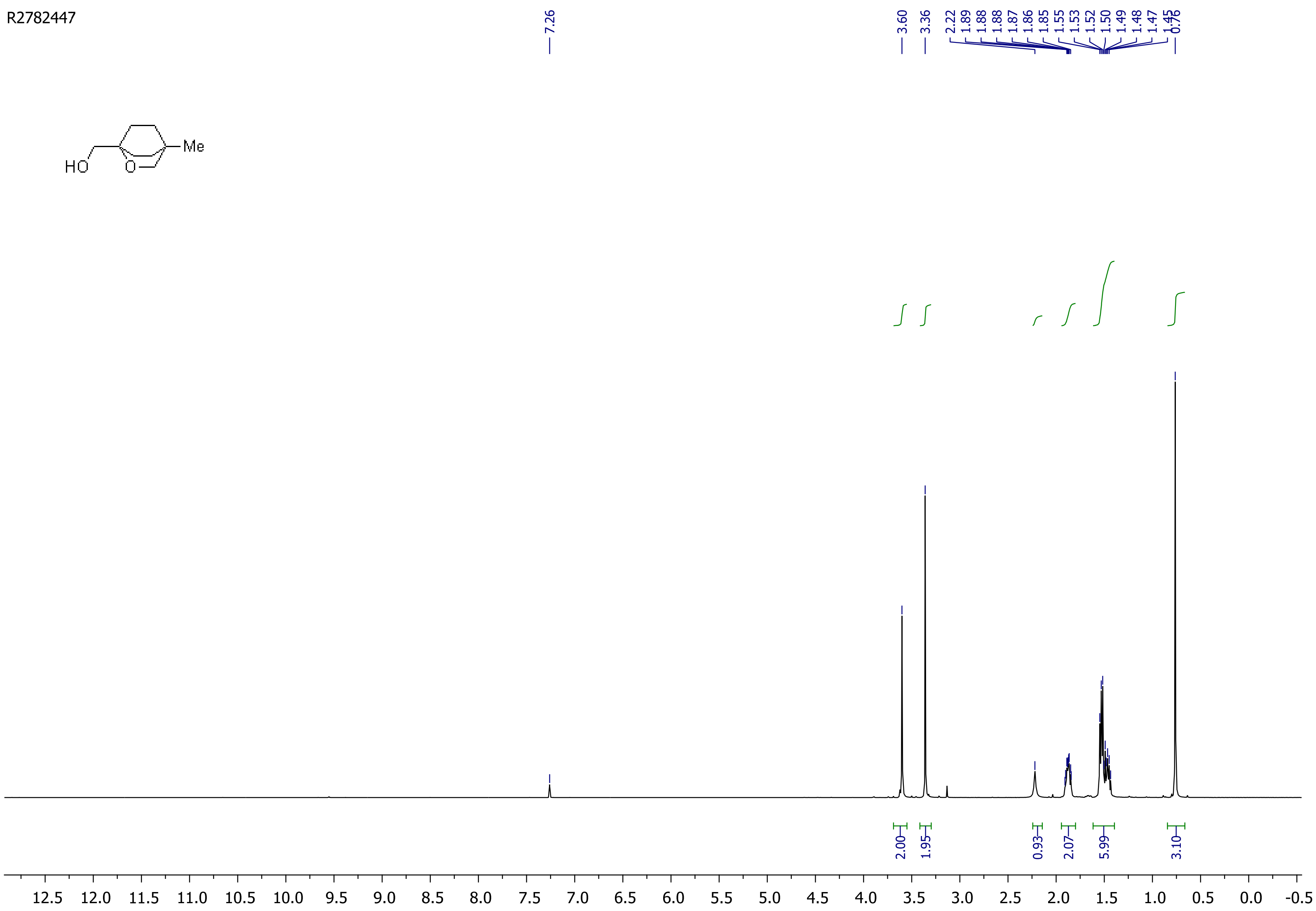

$^{13}C\{^1H\}$ NMR (126 MHz, $CDCl_3$)

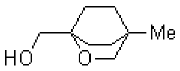

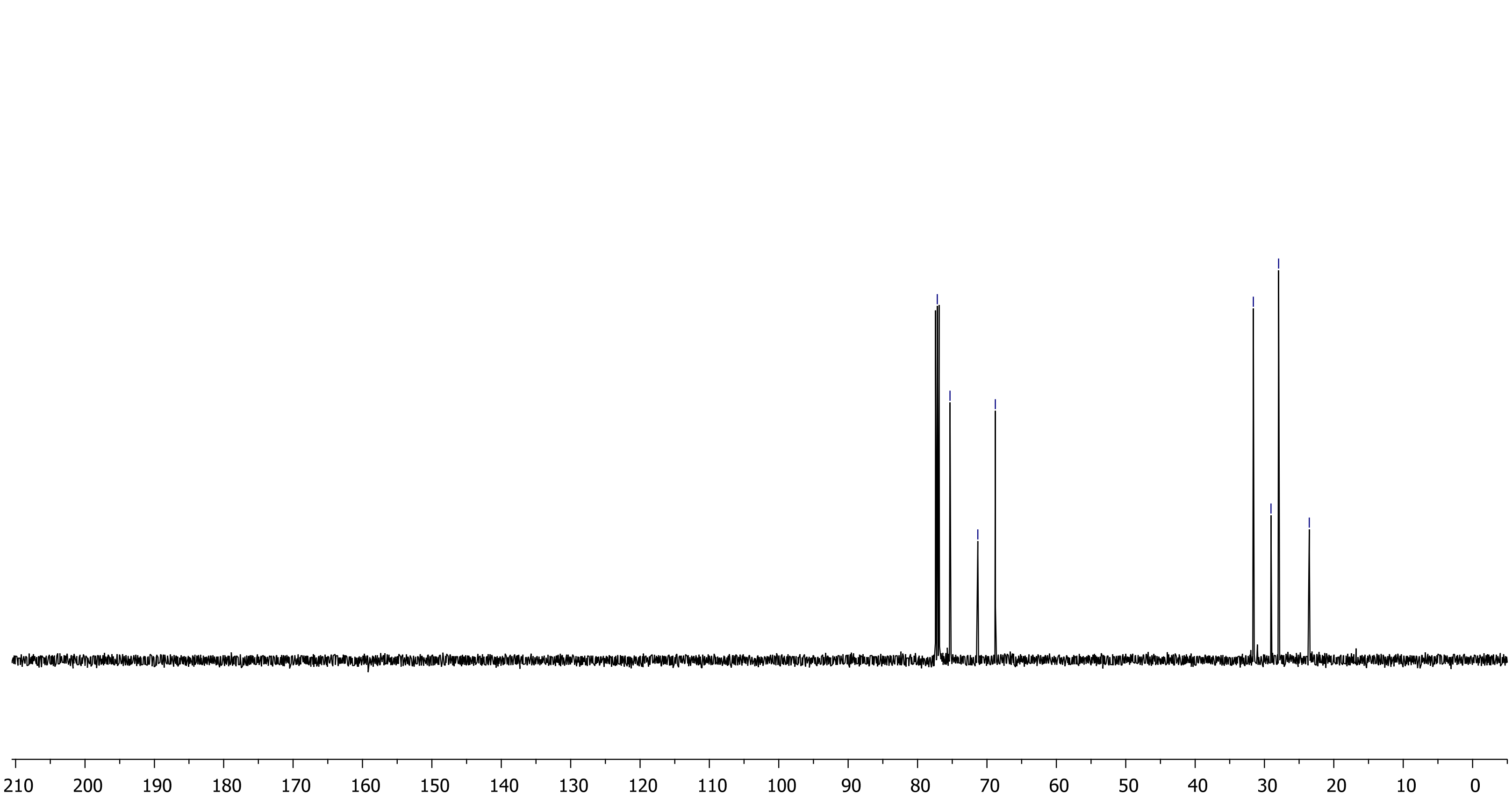

Compound 54

^{1}H NMR (500 MHz, $CDCl_3$)

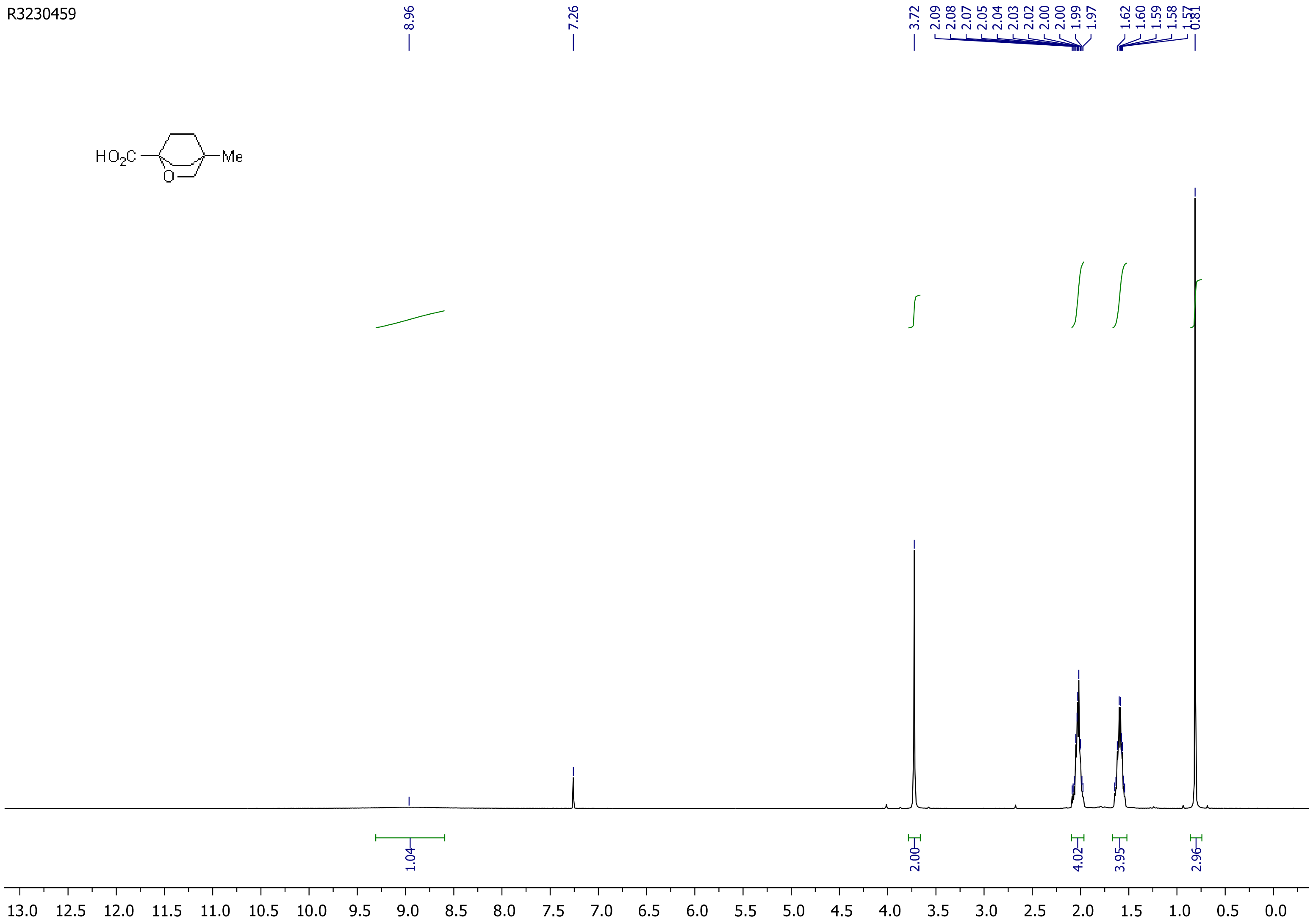

$^{13}C\{^1H\}$ NMR (126 MHz, $CDCl_3$)

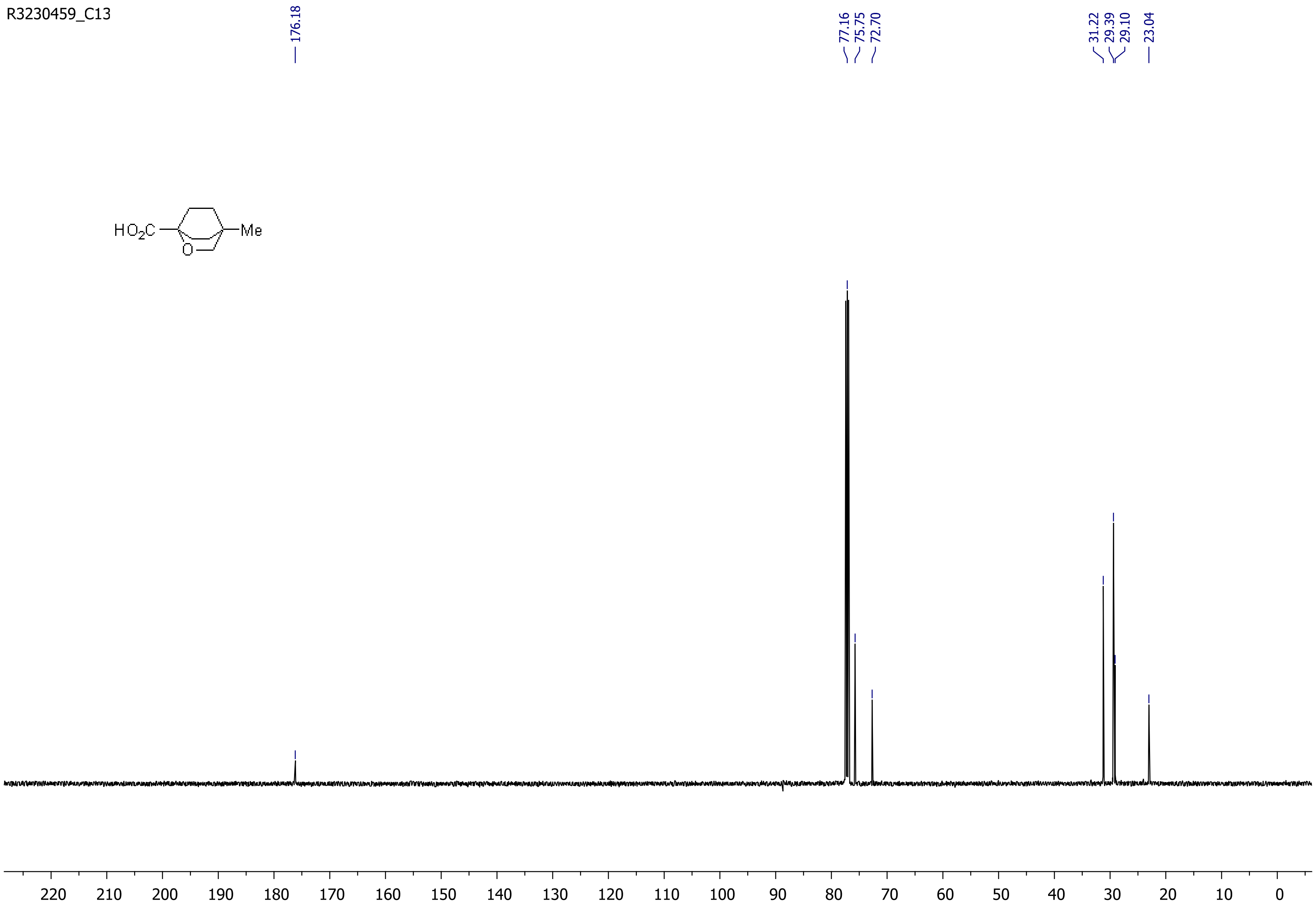

Compound SI-18

^{1}H NMR (500 MHz, $CDCl_3$)

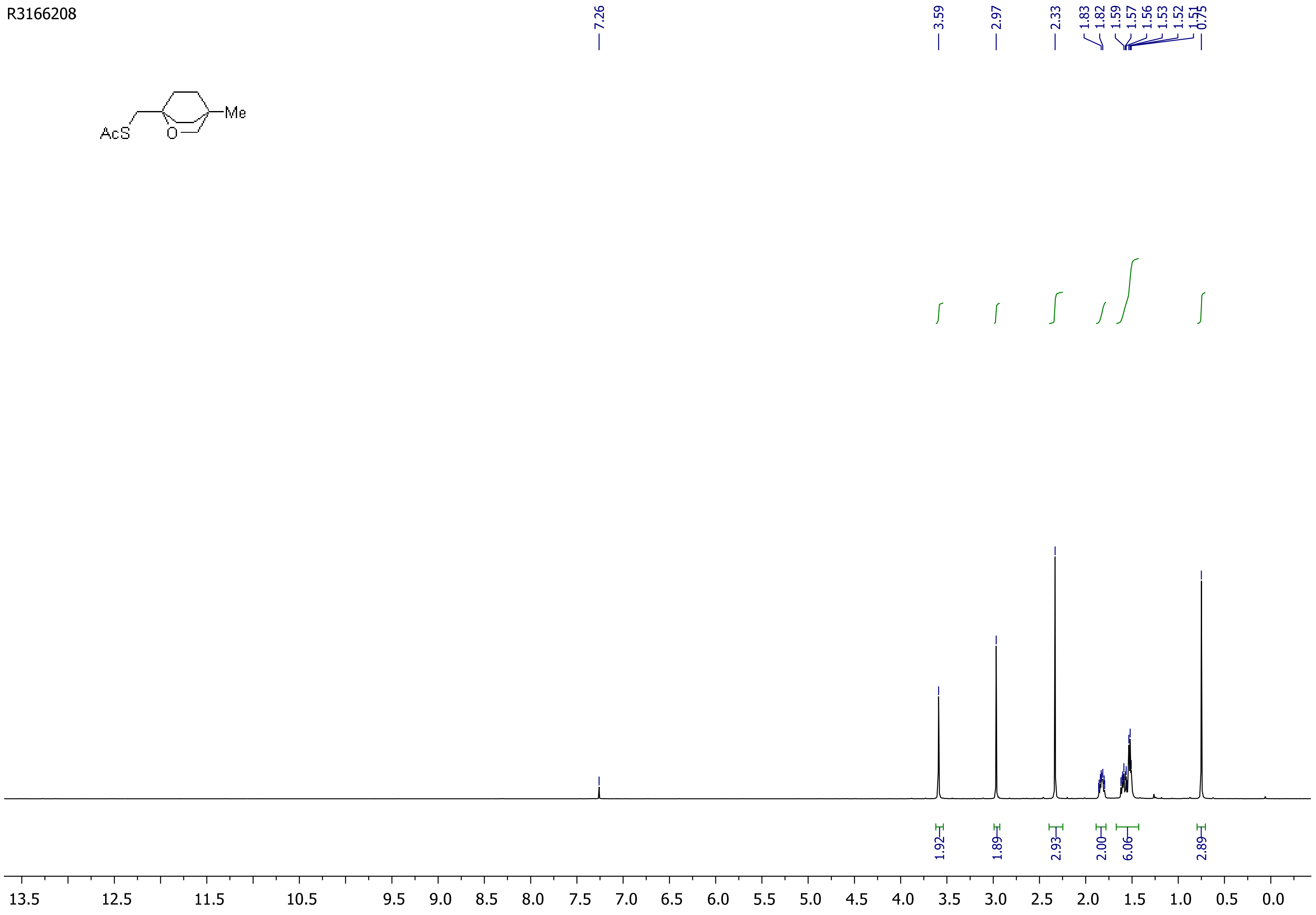

$^{13}C\{^1H\}$ NMR (126 MHz, $CDCl_3$)

R3166208_C13

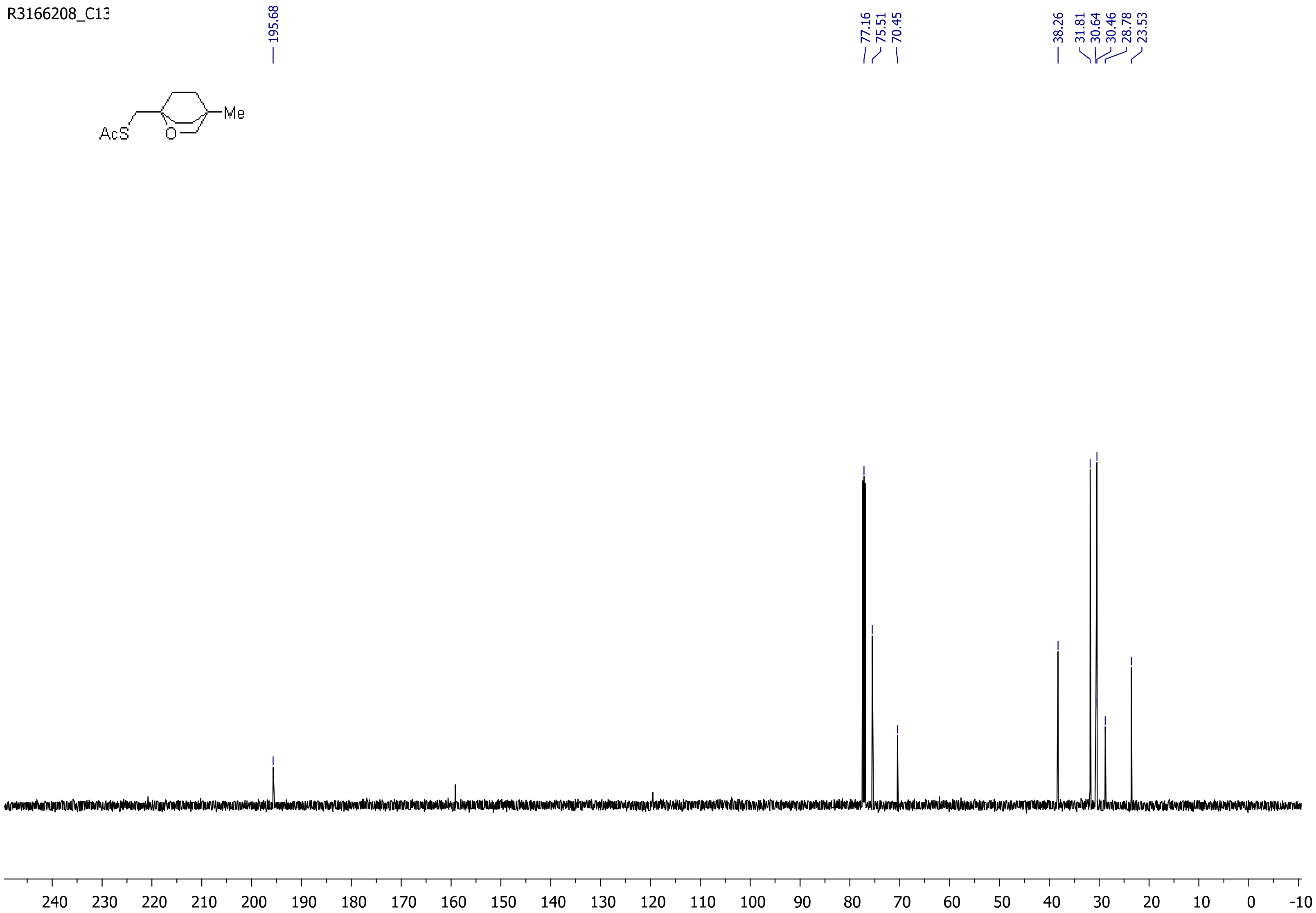

Compound 55

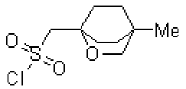

^1H NMR (500 MHz, $CDCl_3$)

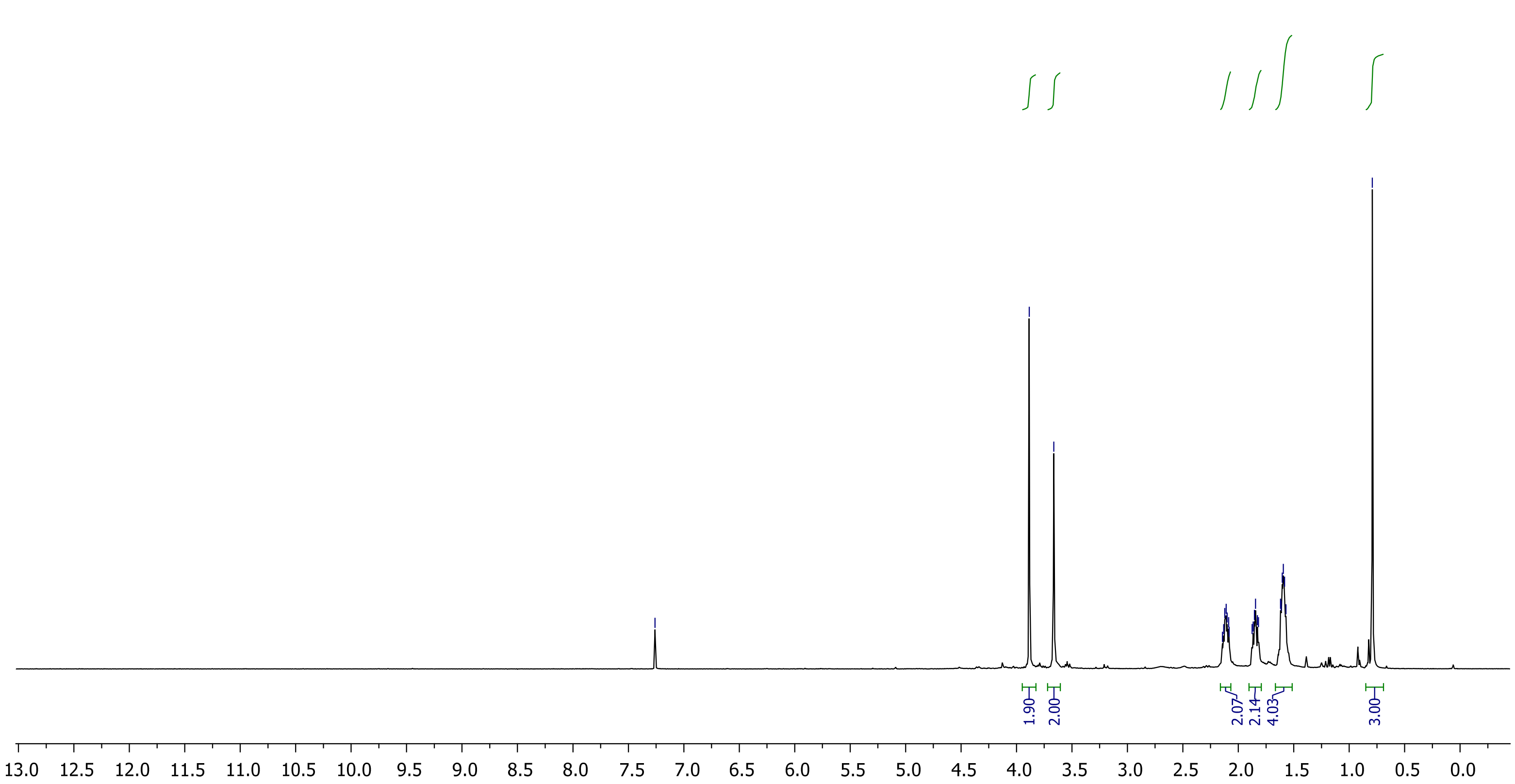

$^{13}C\{^1H\}$ NMR (126 MHz, $CDCl_3$)

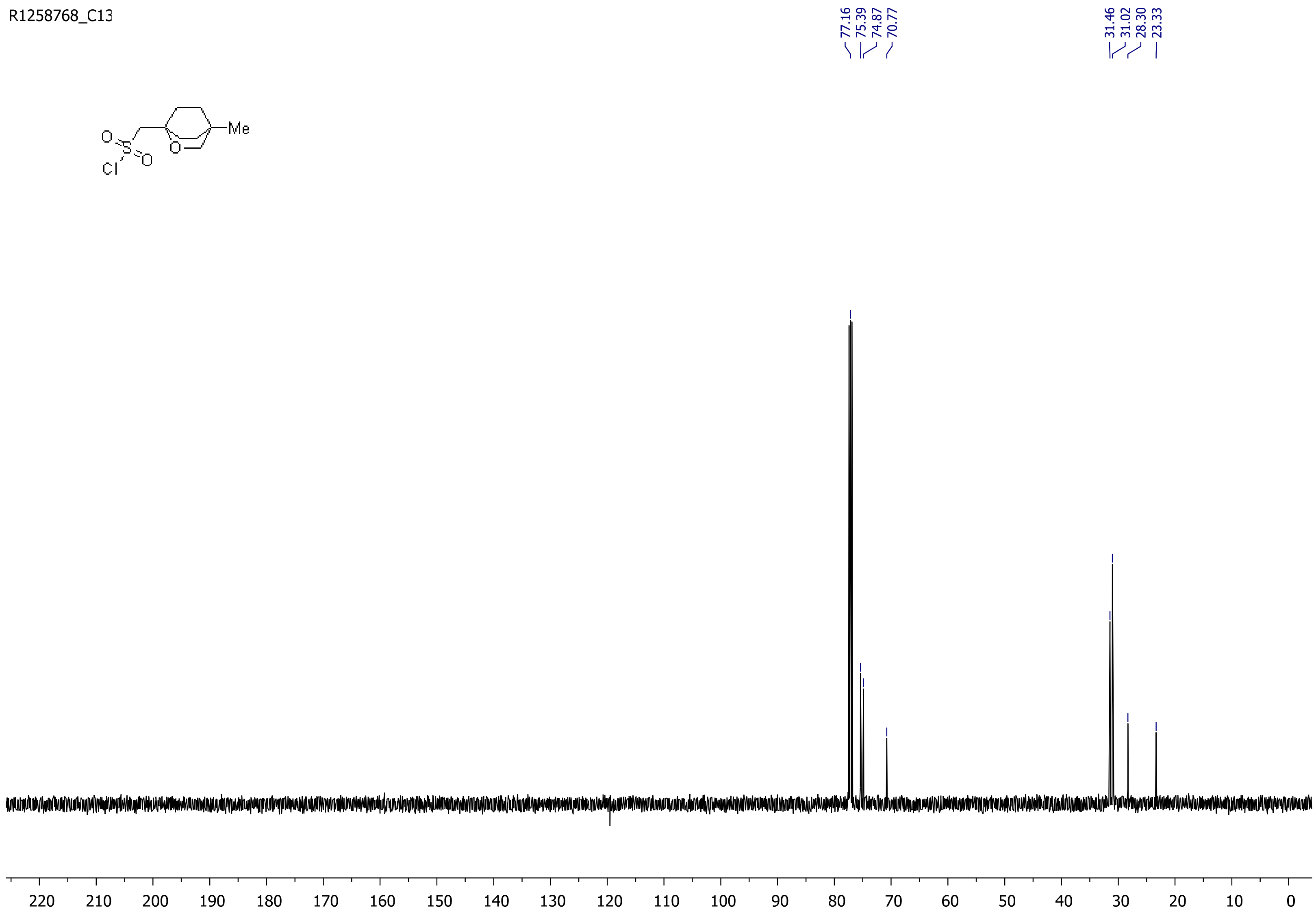

Compound 56

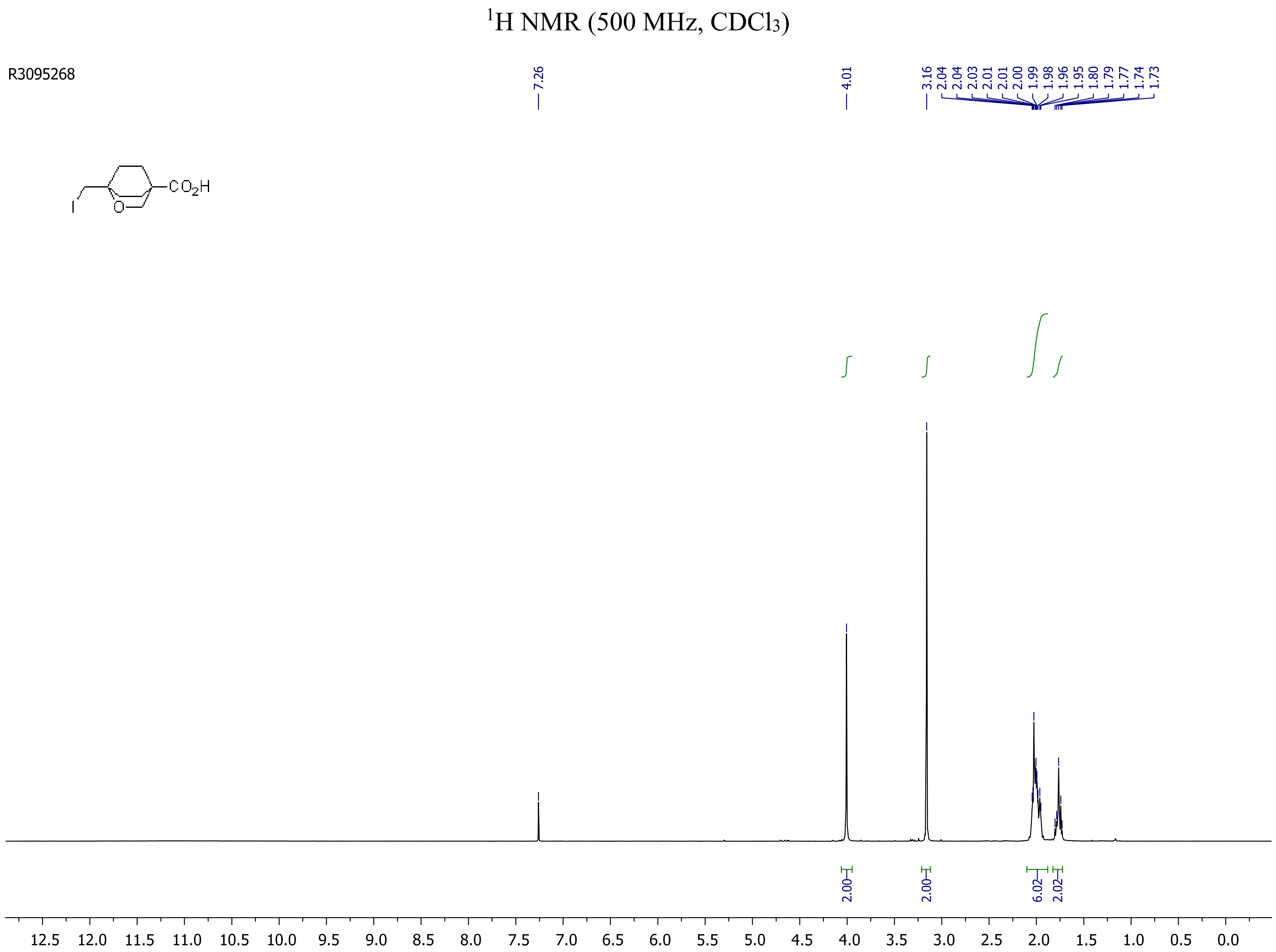

^{1}H NMR (500 MHz, CDCl$_3$)
R3095268
CO$_2$H
O
I
7.26
4.01
3.16
2.04
2.04
2.03
2.01
2.01
2.00
1.99
1.98
1.96
1.95
1.80
1.79
1.77
1.74
1.73
2.00
2.00
6.02
2.02
12.5 12.0 11.5 11.0 10.5 10.0 9.5 9.0 8.5 8.0 7.5 7.0 6.5 6.0 5.5 5.0 4.5 4.0 3.5 3.0 2.5 2.0 1.5 1.0 0.5 0.0

$^{13}C\{^1H\}$ NMR (126 MHz, $CDCl_3$)

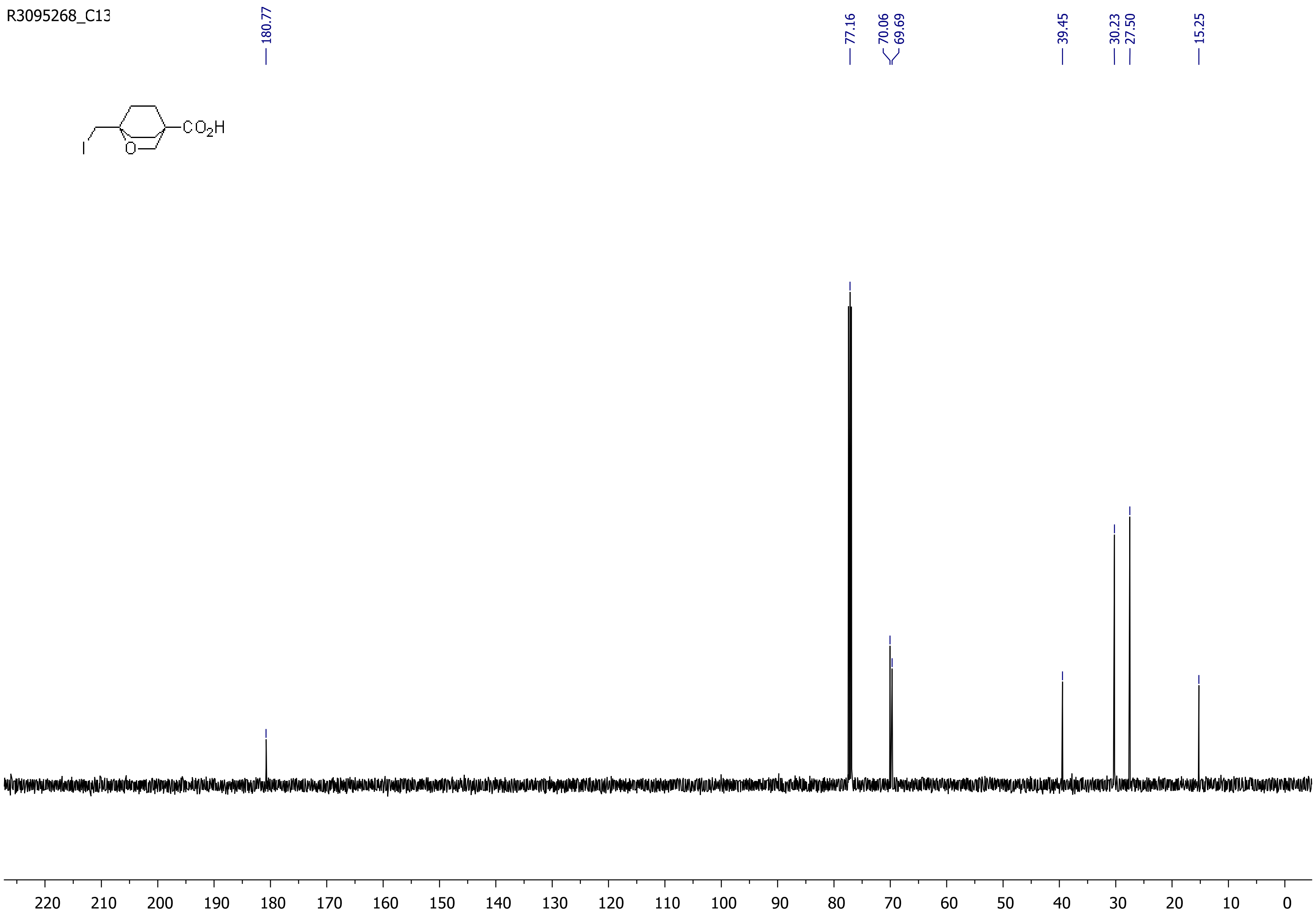

Compound 57

^{1}H NMR (500 MHz, $CDCl_3$)

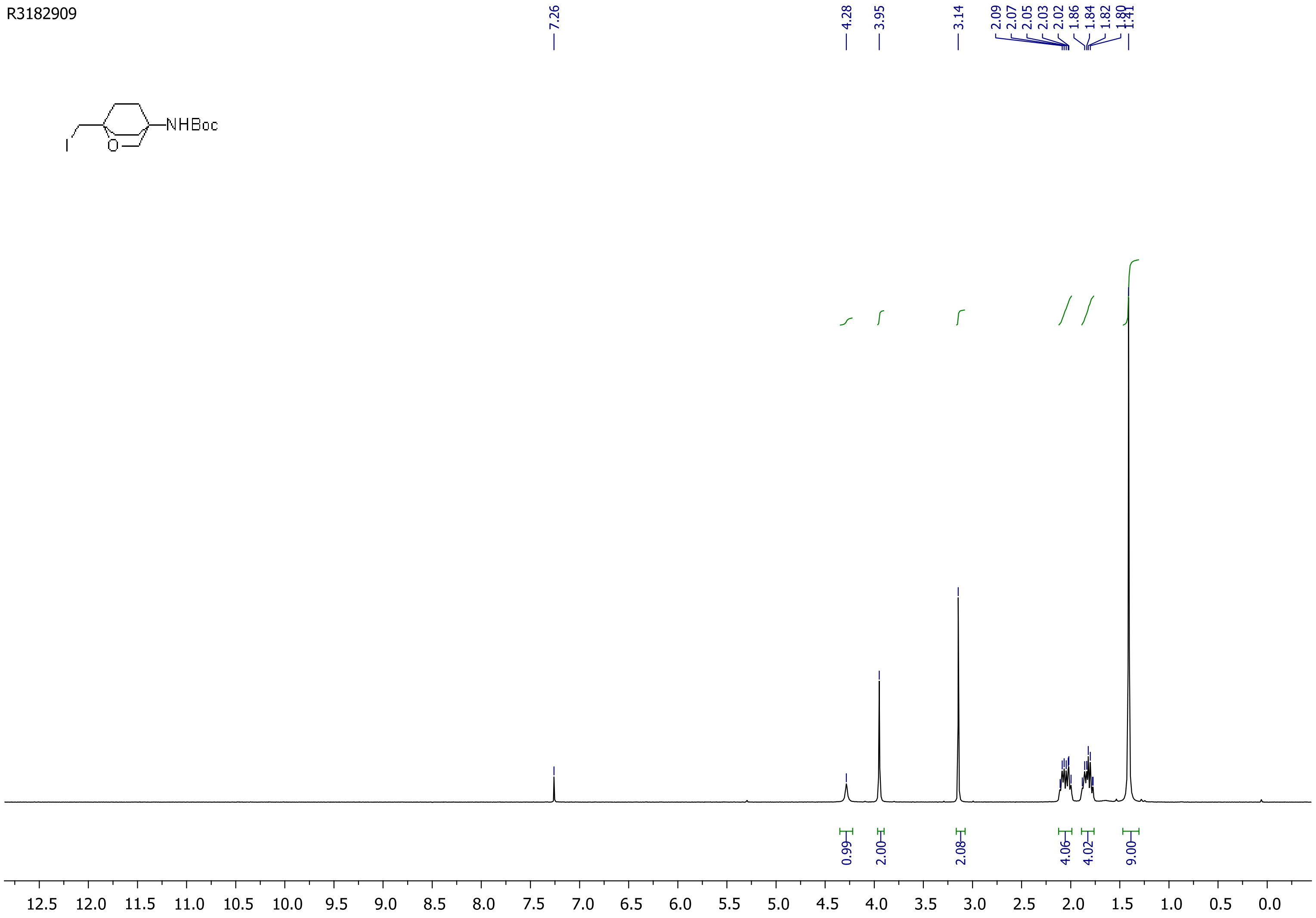

$^{13}C\{^1H\}$ NMR (151 MHz, $CDCl_3$)

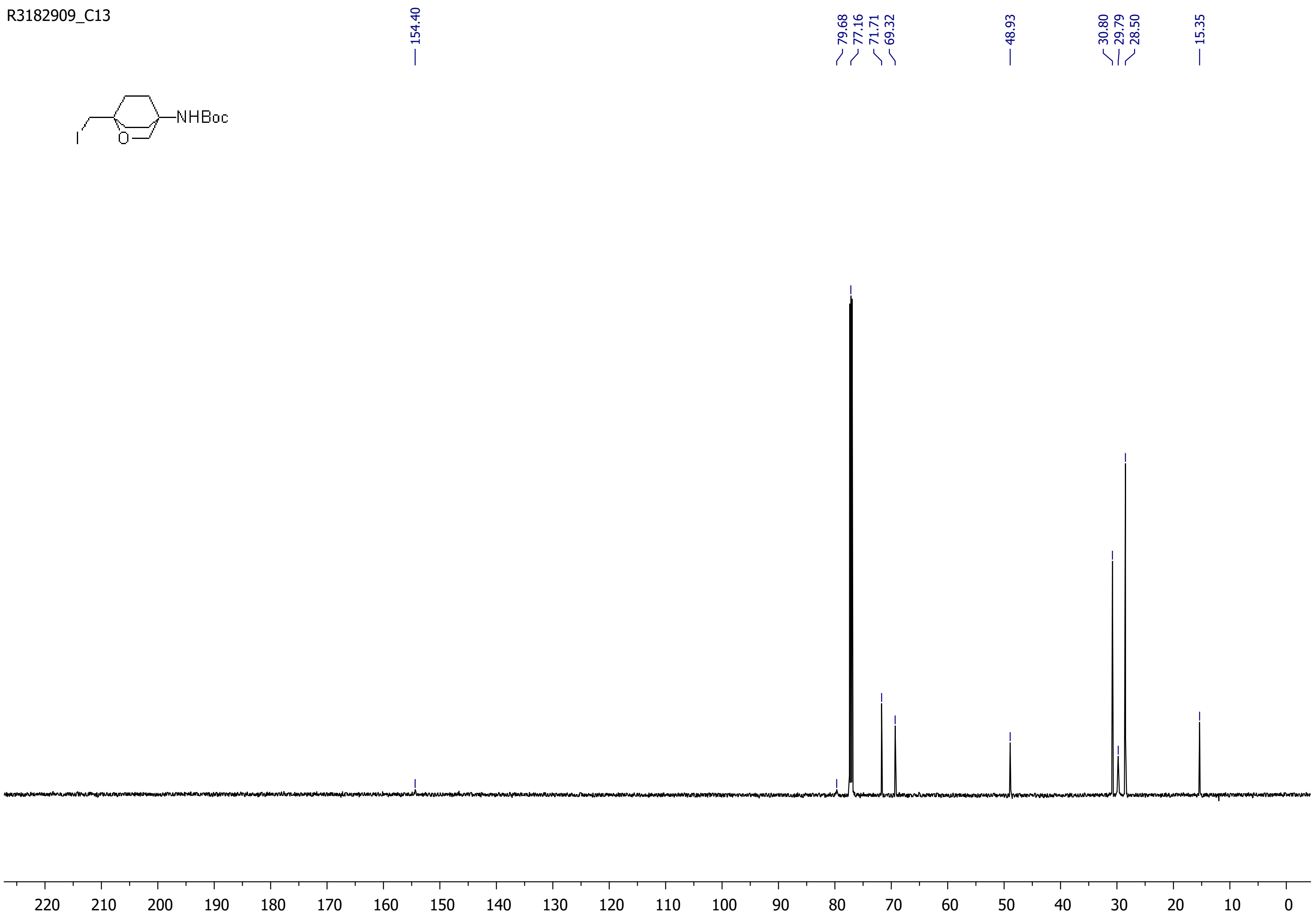

Compound SI-19

^1H NMR (500 MHz, $CDCl_3$)

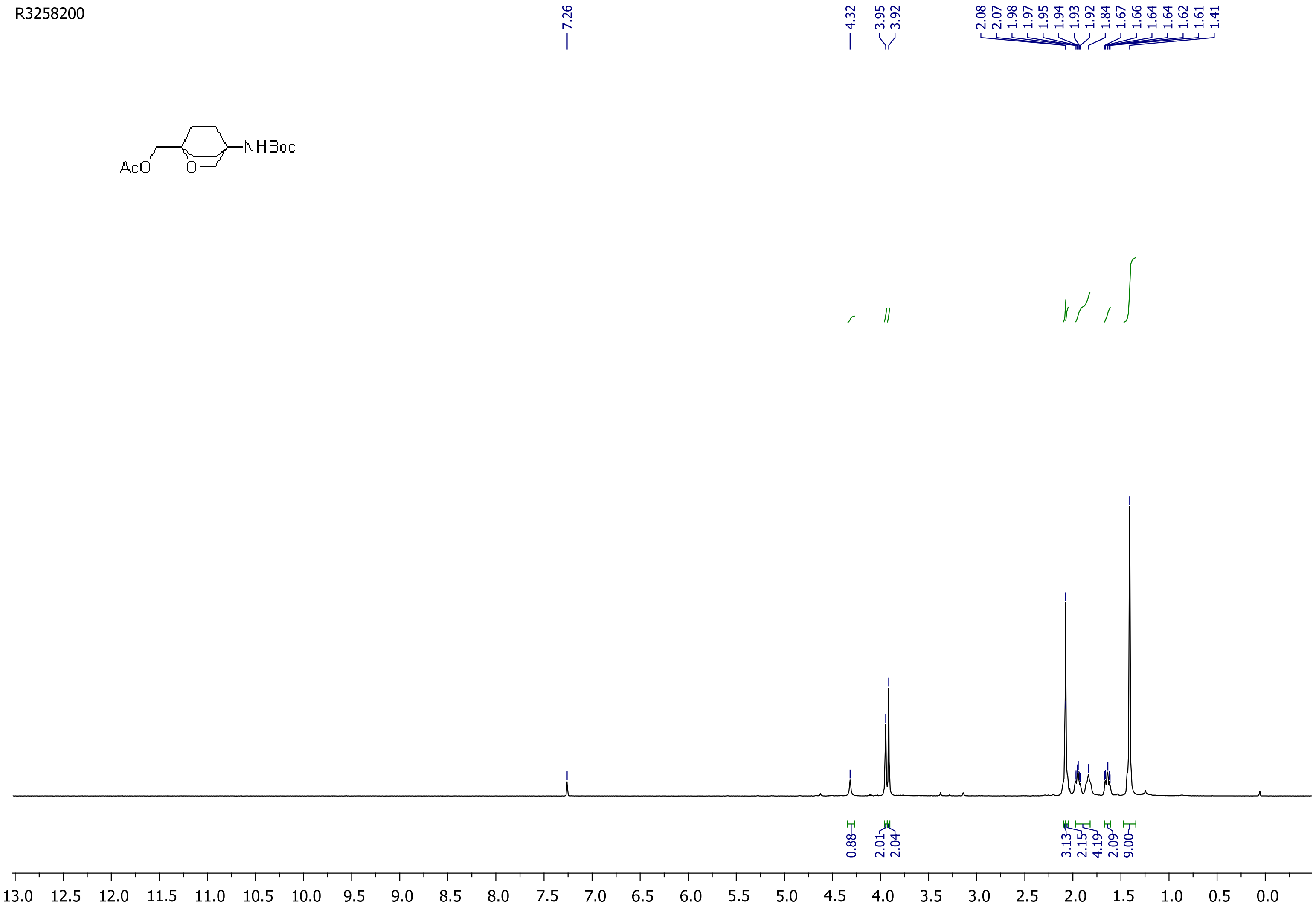

$^{13}C\{^{1}H\}$ NMR (126 MHz, $CDCl_3$)

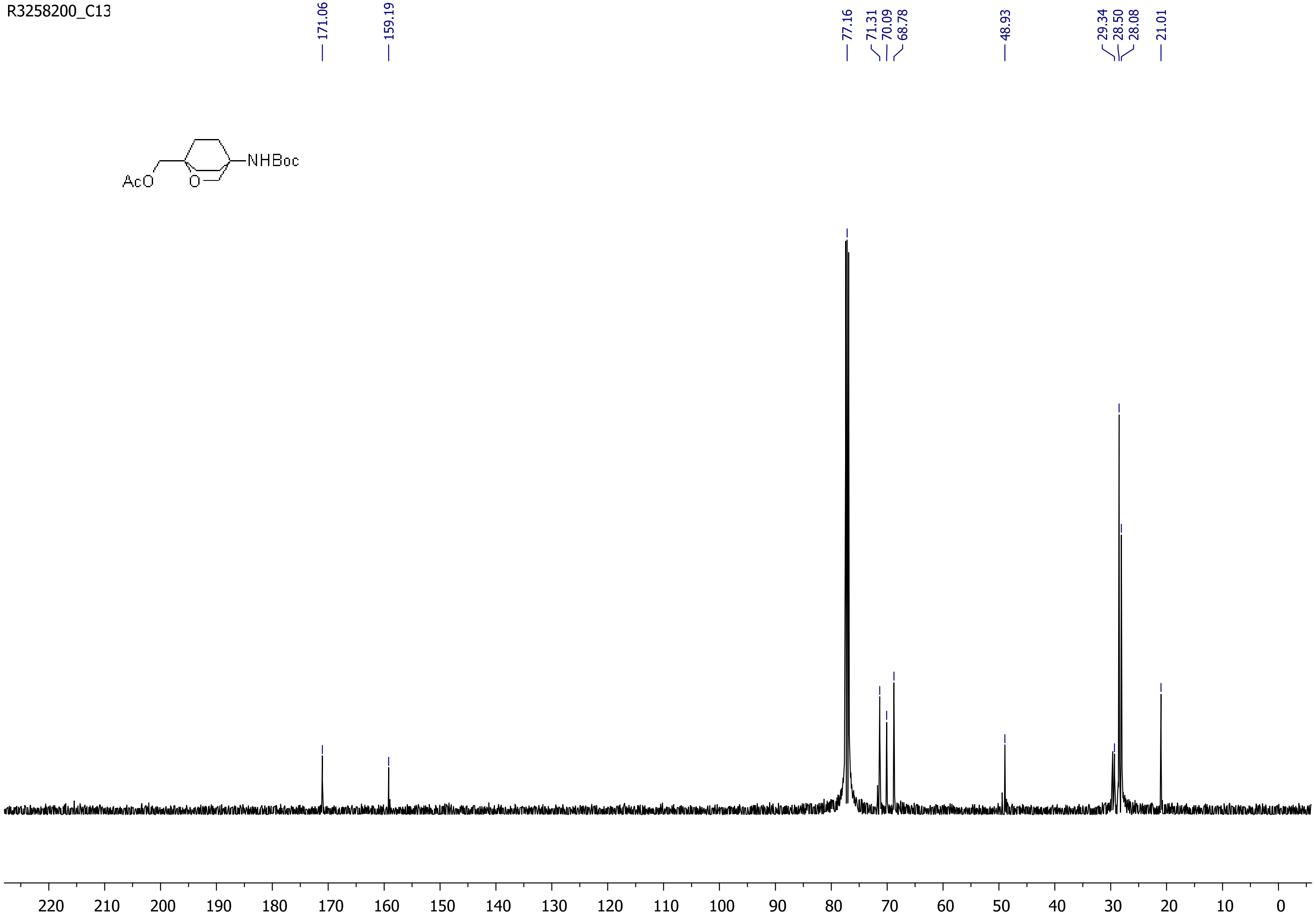

Compound 58

^{1}H NMR (500 MHz, $CDCl_3$)

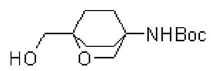

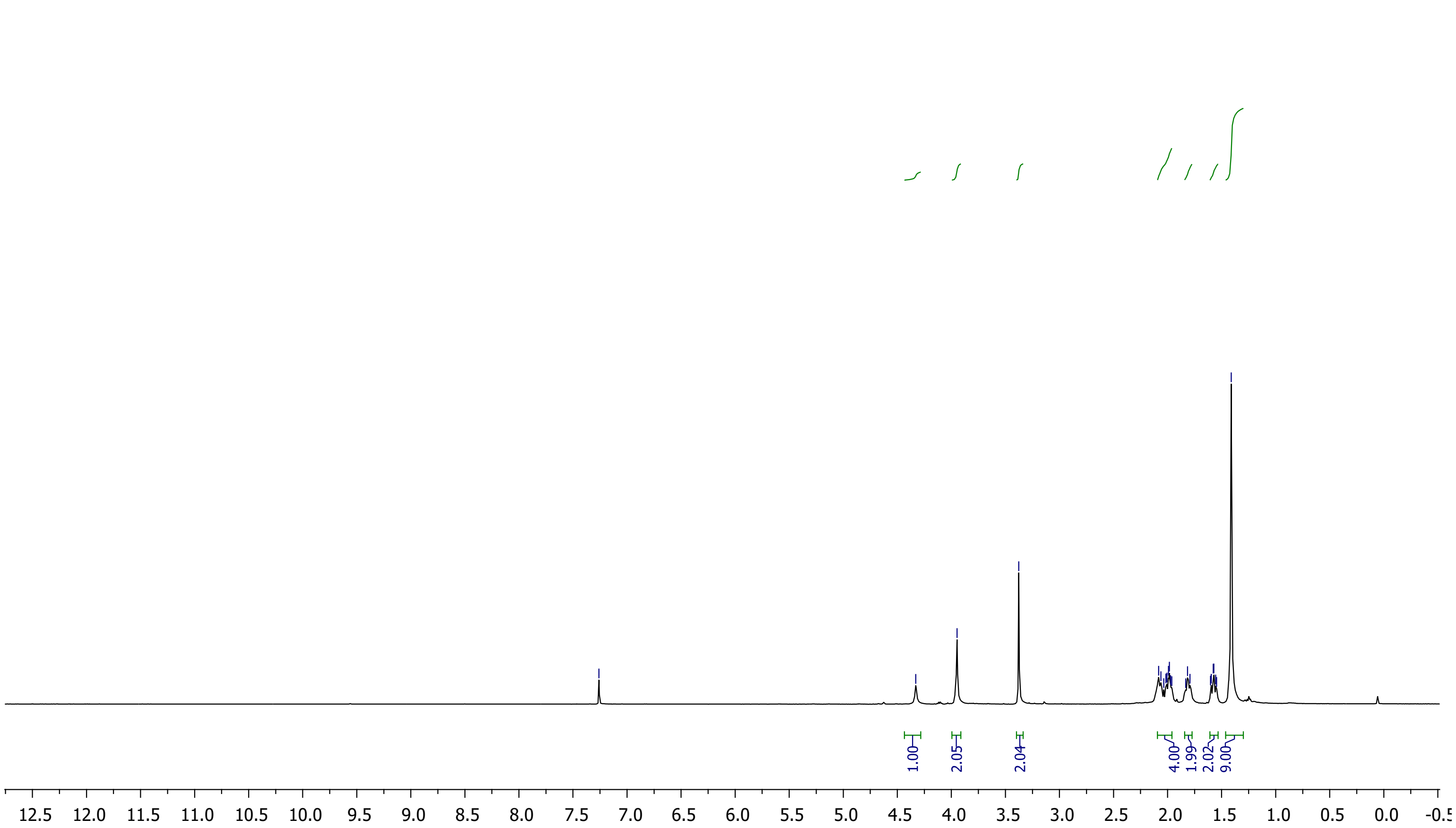

$^{13}C\{^1H\}$ NMR (126 MHz, $CDCl_3$)

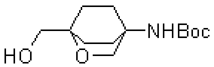

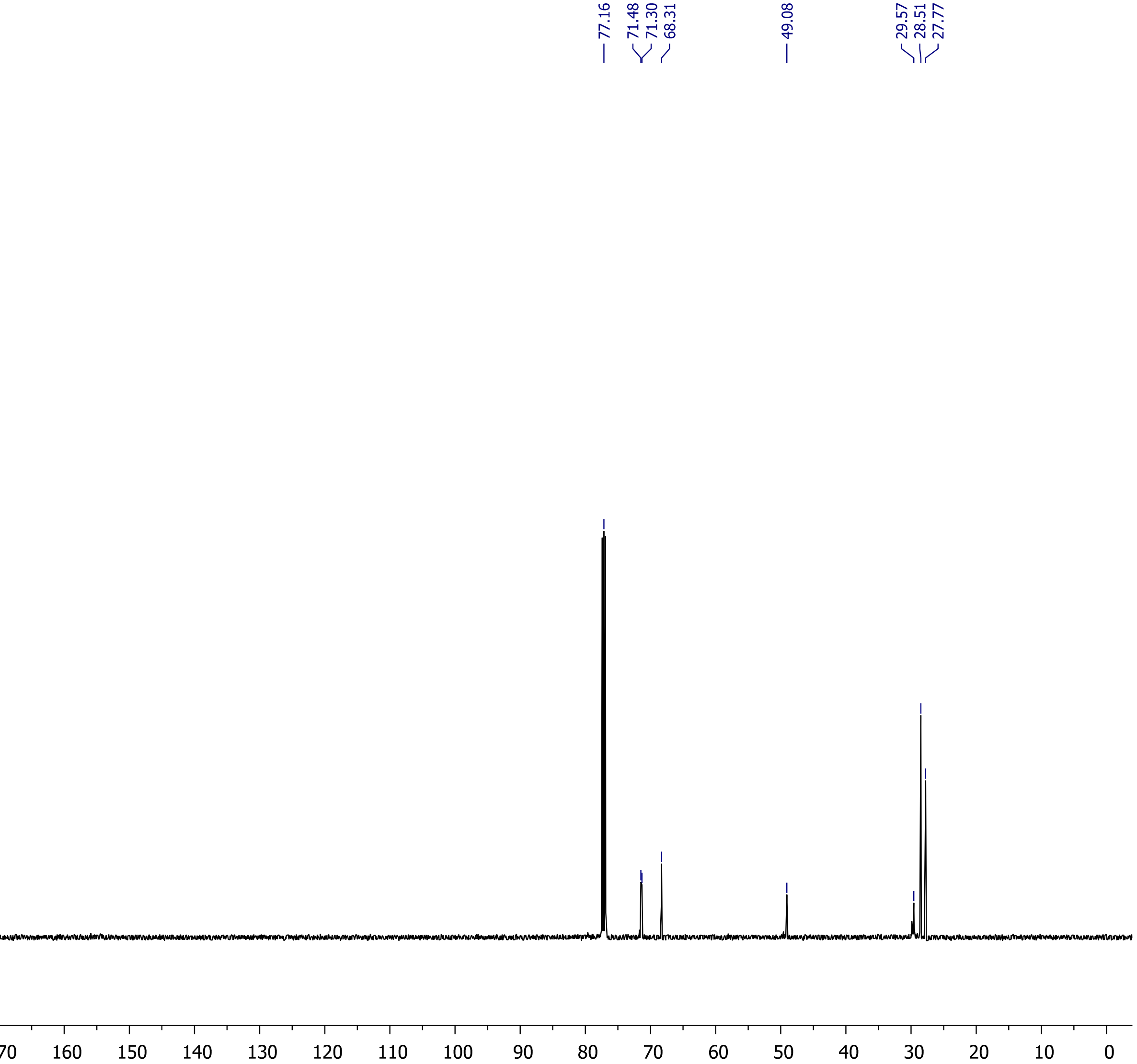

Compound 59

^{1}H NMR (500 MHz, DMSO-d$_6$)

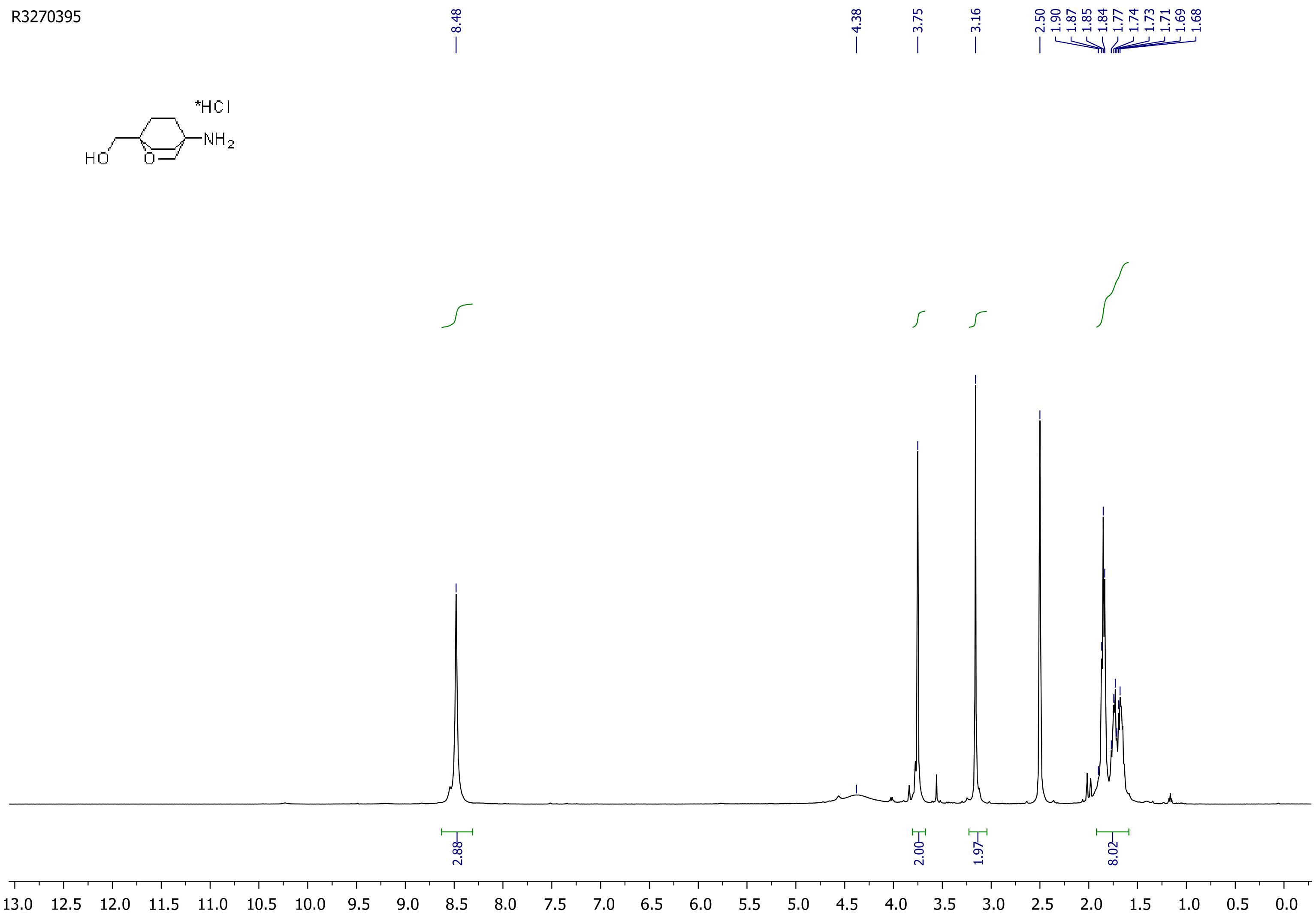

$^{13}C\{^{1}H\}$ NMR (151 MHz, DMSO-d_6)

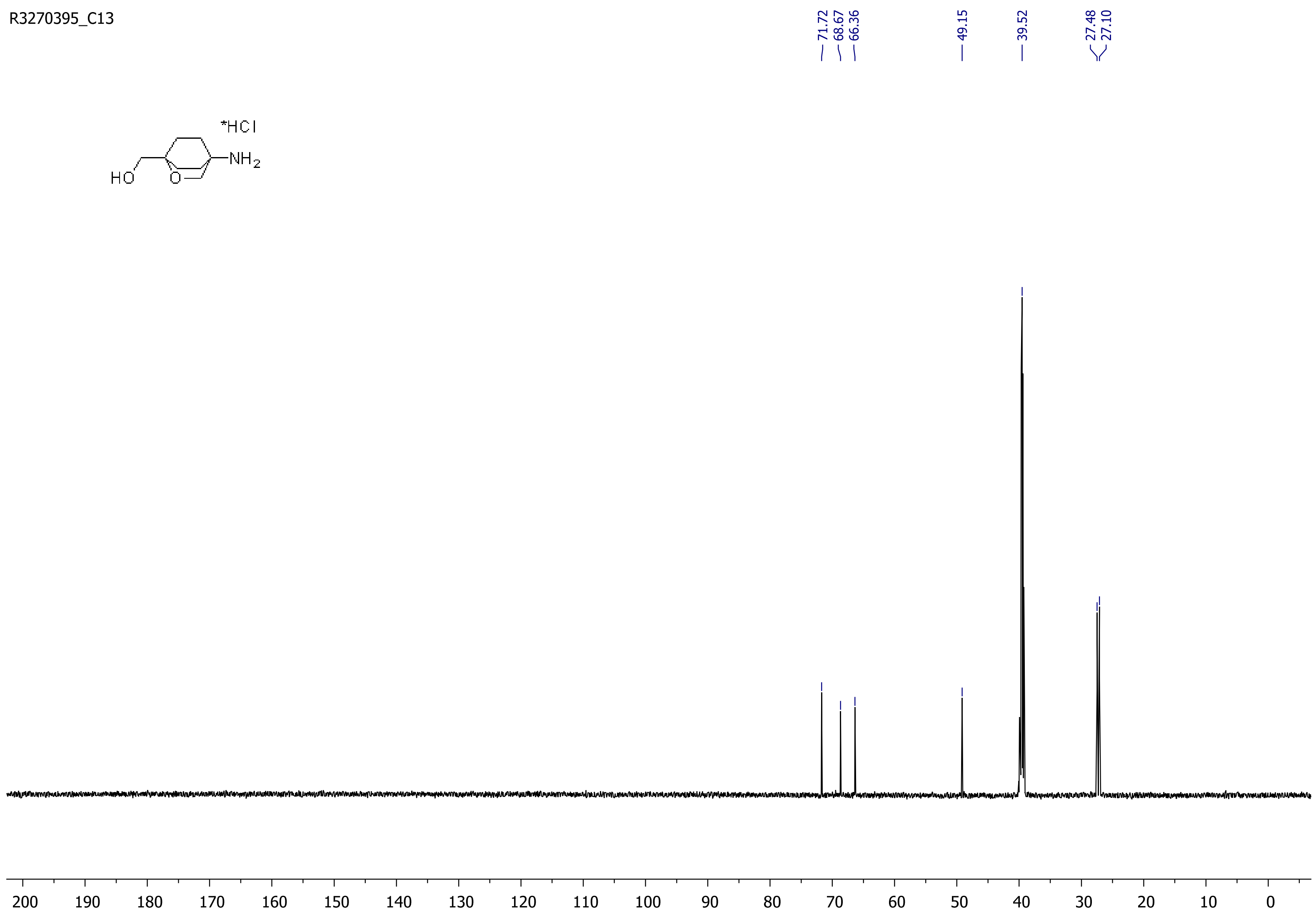

Compound 60

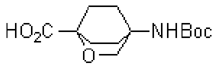
HO2C
NHBoc
O

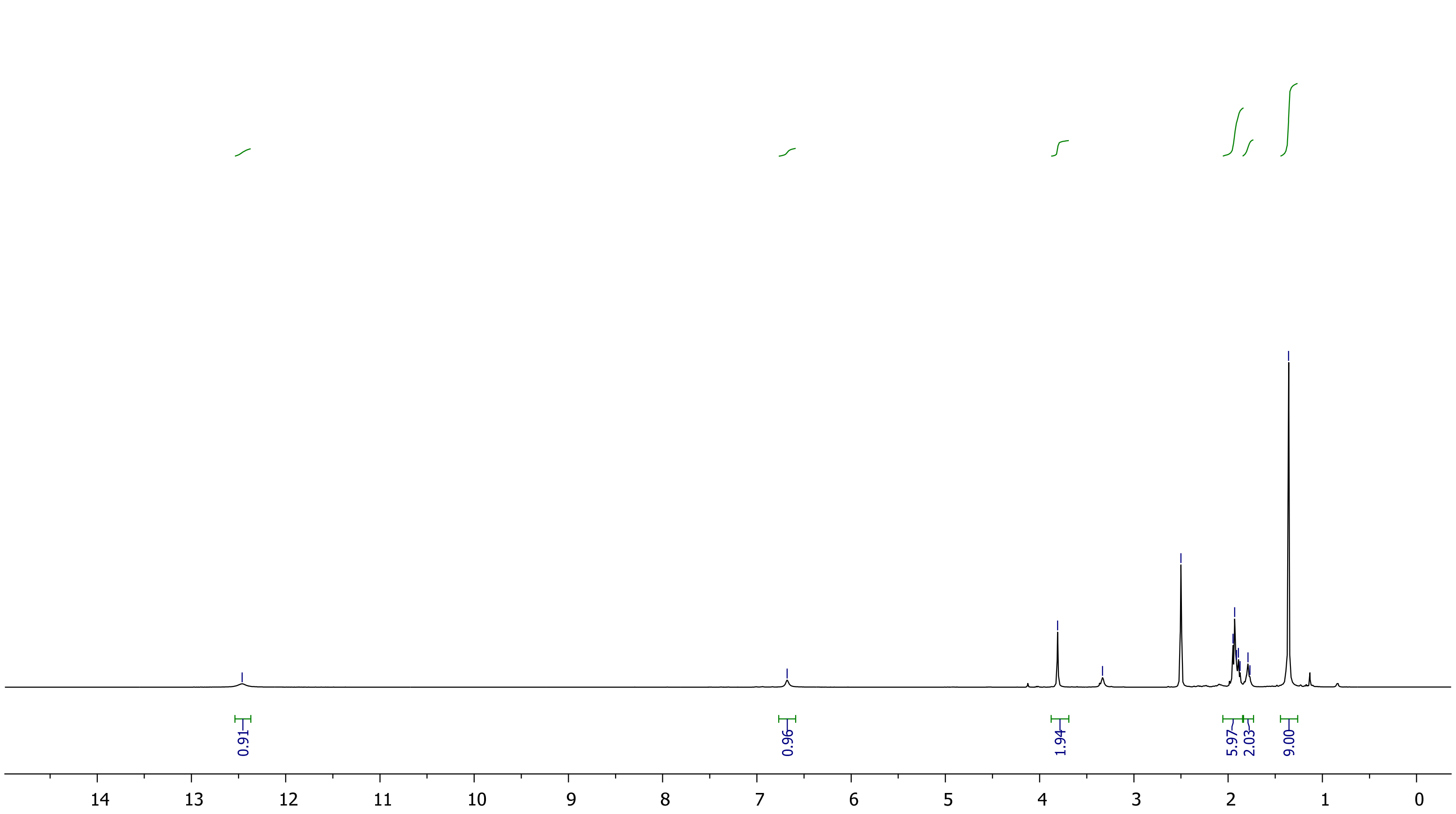
1H NMR (500 MHz, DMSO-d6)
R1627616
12.46
6.68
3.81
3.33
2.50
1.95
1.93
1.91
1.89
1.79
1.36
0.91
0.96
1.94
5.97
2.03
9.00
14
13
12
11
10
9
8
7
6
5
4
3
2
1
0

$^{13}C\{^{1}H\}$ NMR (151 MHz, DMSO-d6)

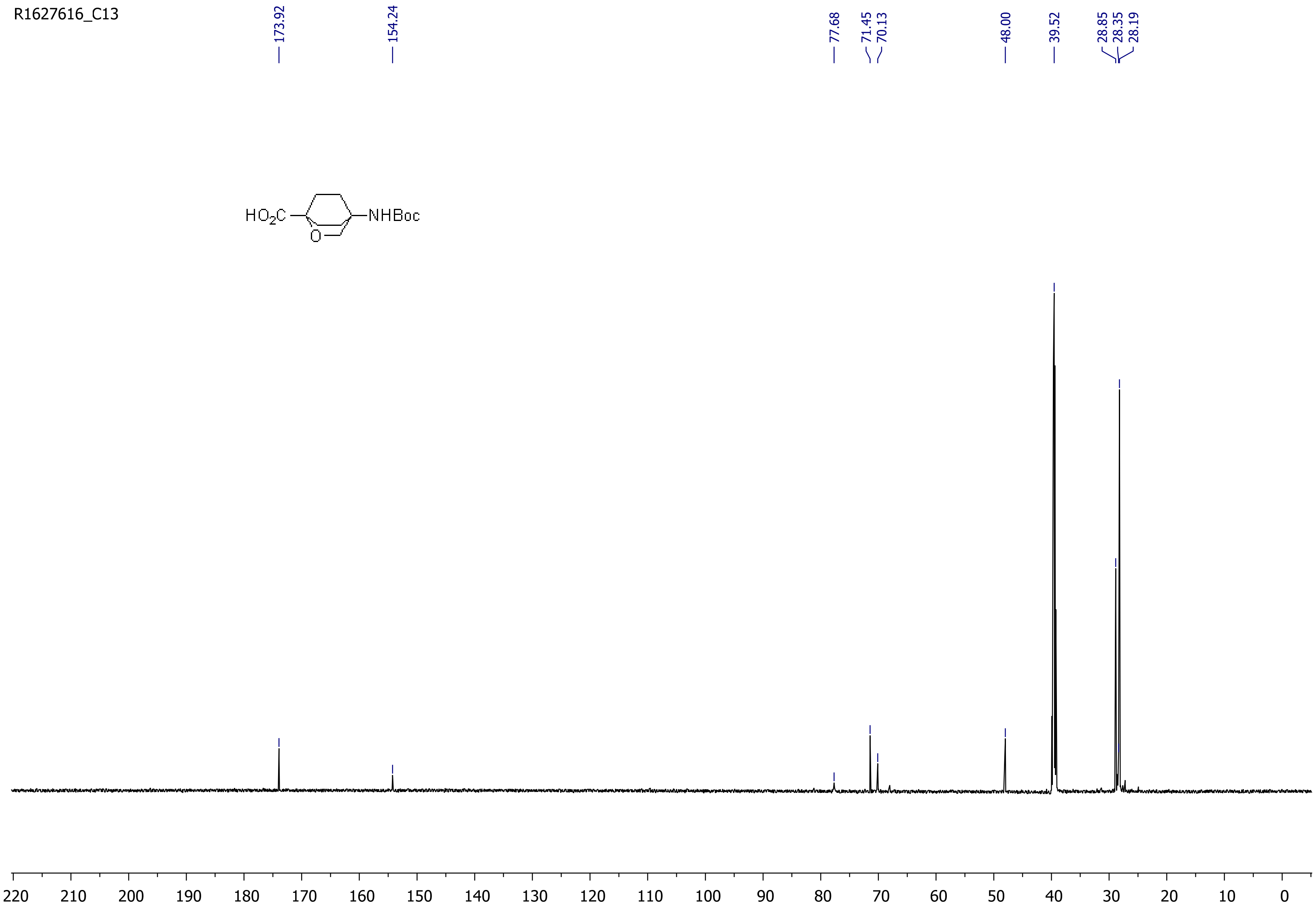

Compound 61

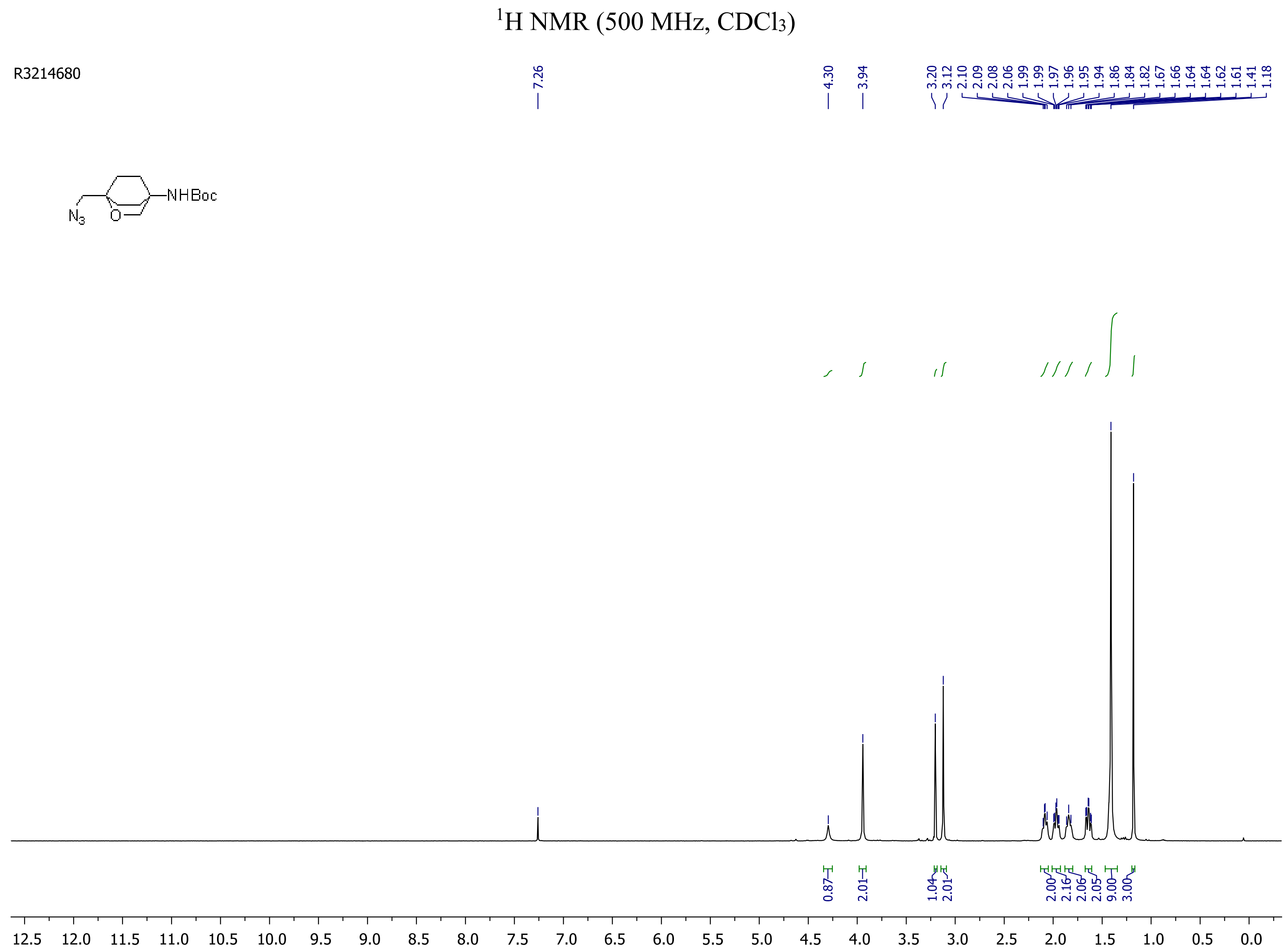

¹H NMR (500 MHz, CDCl3)
R3214680
NHBoc
N3
O
7.26
4.30
3.94
3.20
3.12
2.10
2.09
2.08
2.06
1.99
1.97
1.96
1.95
1.94
1.86
1.84
1.82
1.67
1.66
1.64
1.64
1.62
1.61
1.41
1.18
0.87
2.01
1.04
2.01
2.00
2.16
2.06
2.05
9.00
3.00
12.5 12.0 11.5 11.0 10.5 10.0 9.5 9.0 8.5 8.0 7.5 7.0 6.5 6.0 5.5 5.0 4.5 4.0 3.5 3.0 2.5 2.0 1.5 1.0 0.5 0.0

$^{13}C\{^1H\}$ NMR (151 MHz, $CDCl_3$)

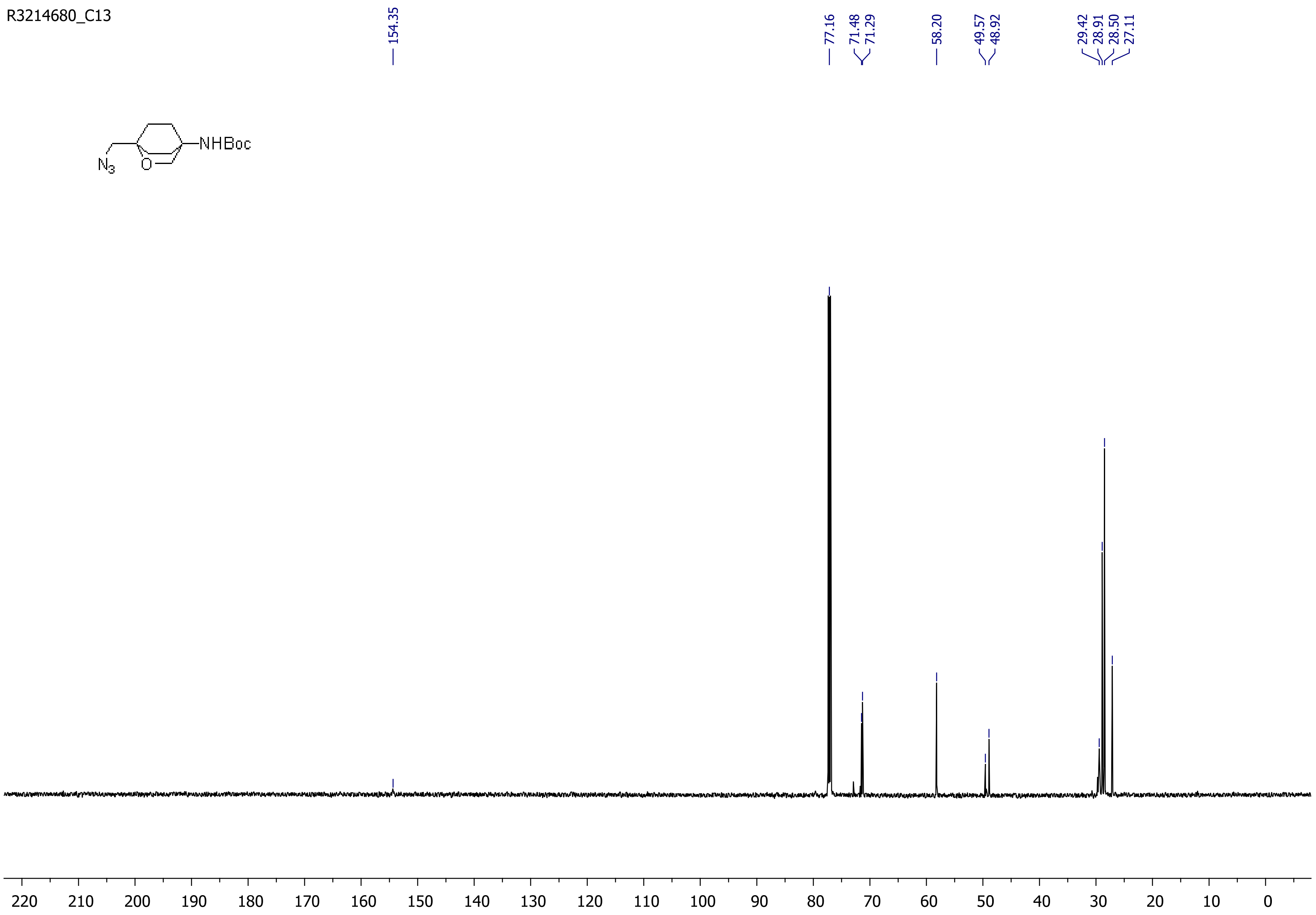

Compound 62

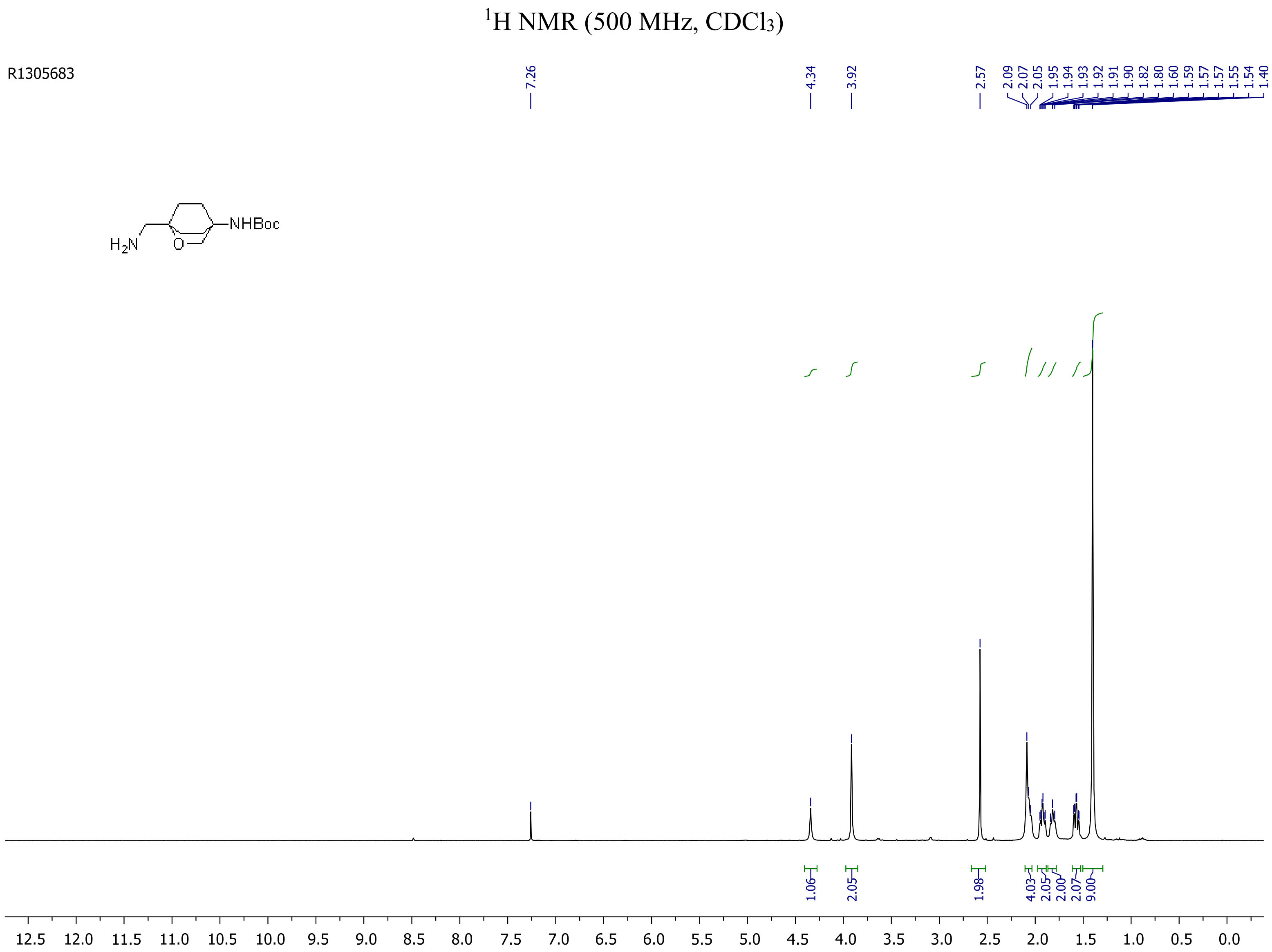

¹H NMR (500 MHz, CDCl3)
R1305683
7.26
4.34
3.92
2.57
2.09
2.07
2.05
1.95
1.94
1.93
1.92
1.91
1.90
1.82
1.80
1.60
1.59
1.57
1.57
1.55
1.54
1.40
NHBoc
H2N
O
1.06
2.05
1.98
4.03
2.05
2.00
2.07
9.00
12.5
12.0
11.5
11.0
10.5
10.0
9.5
9.0
8.5
8.0
7.5
7.0
6.5
6.0
5.5
5.0
4.5
4.0
3.5
3.0
2.5
2.0
1.5
1.0
0.5
0.0

$^{13}C\{^{1}H\}$ NMR (151 MHz, $CDCl_3$)

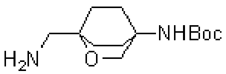

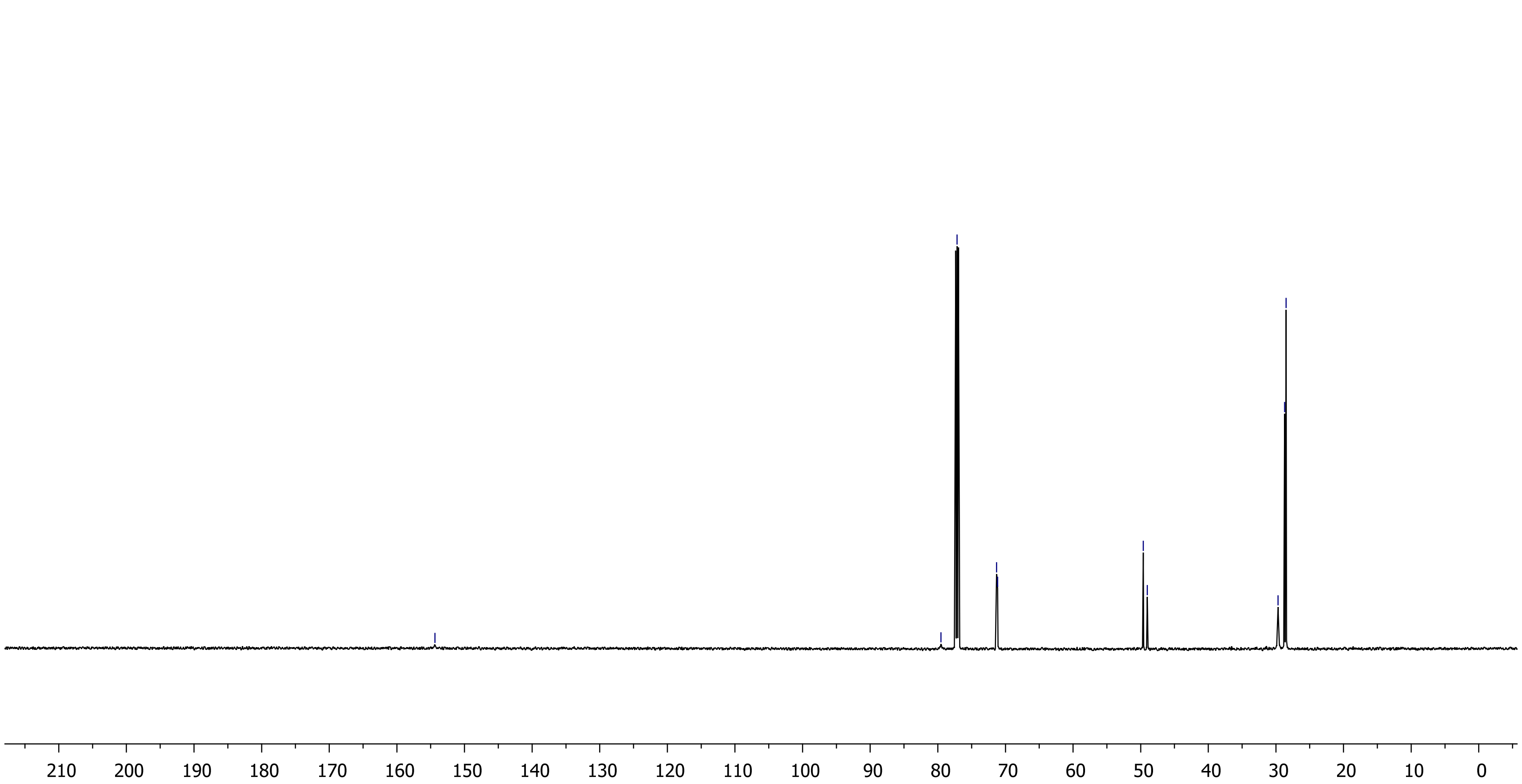

Compound 63

^{1}H NMR (500 MHz, $CDCl_3$)

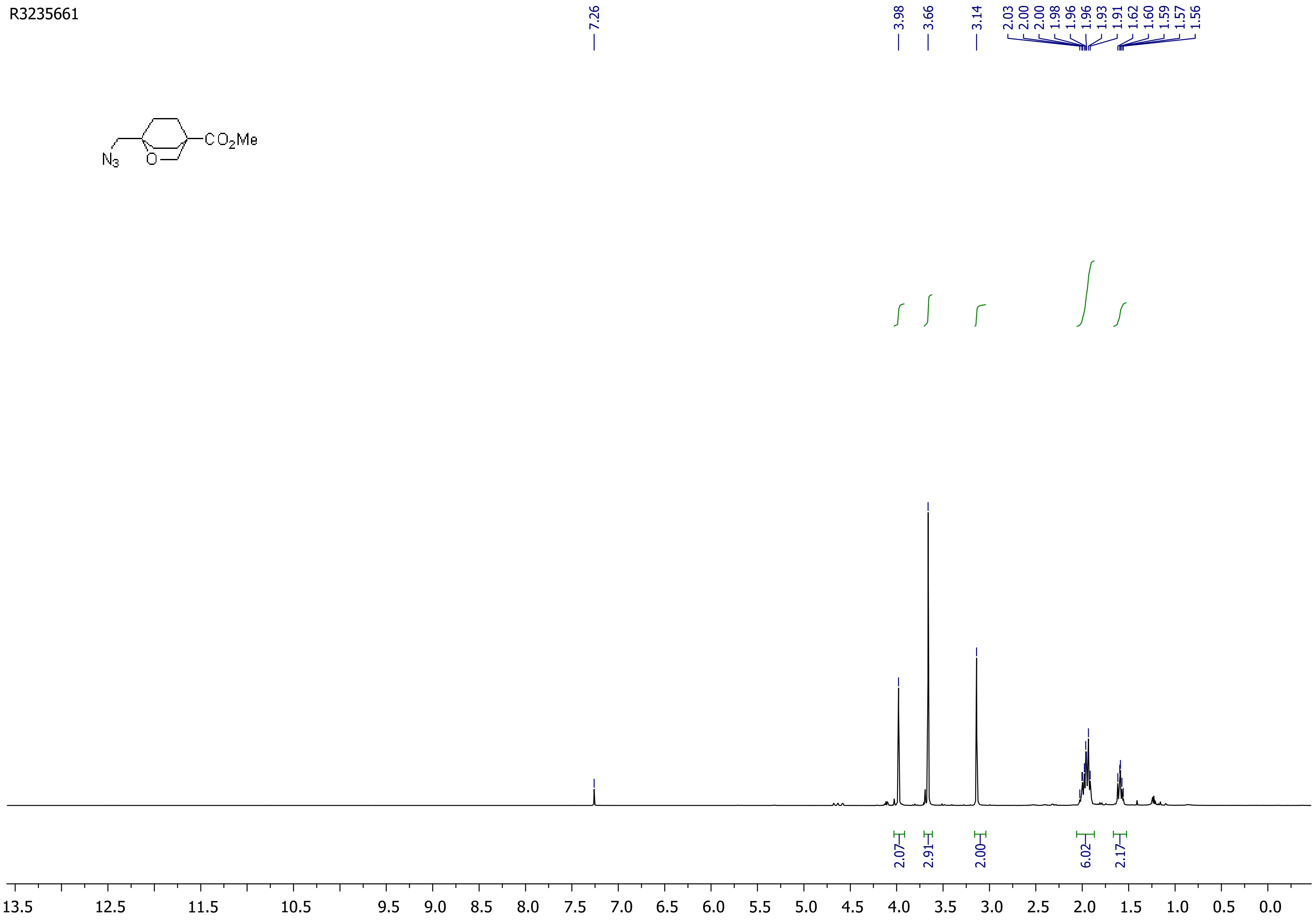

$^{13}C\{^{1}H\}$ NMR (151 MHz, $CDCl_3$)

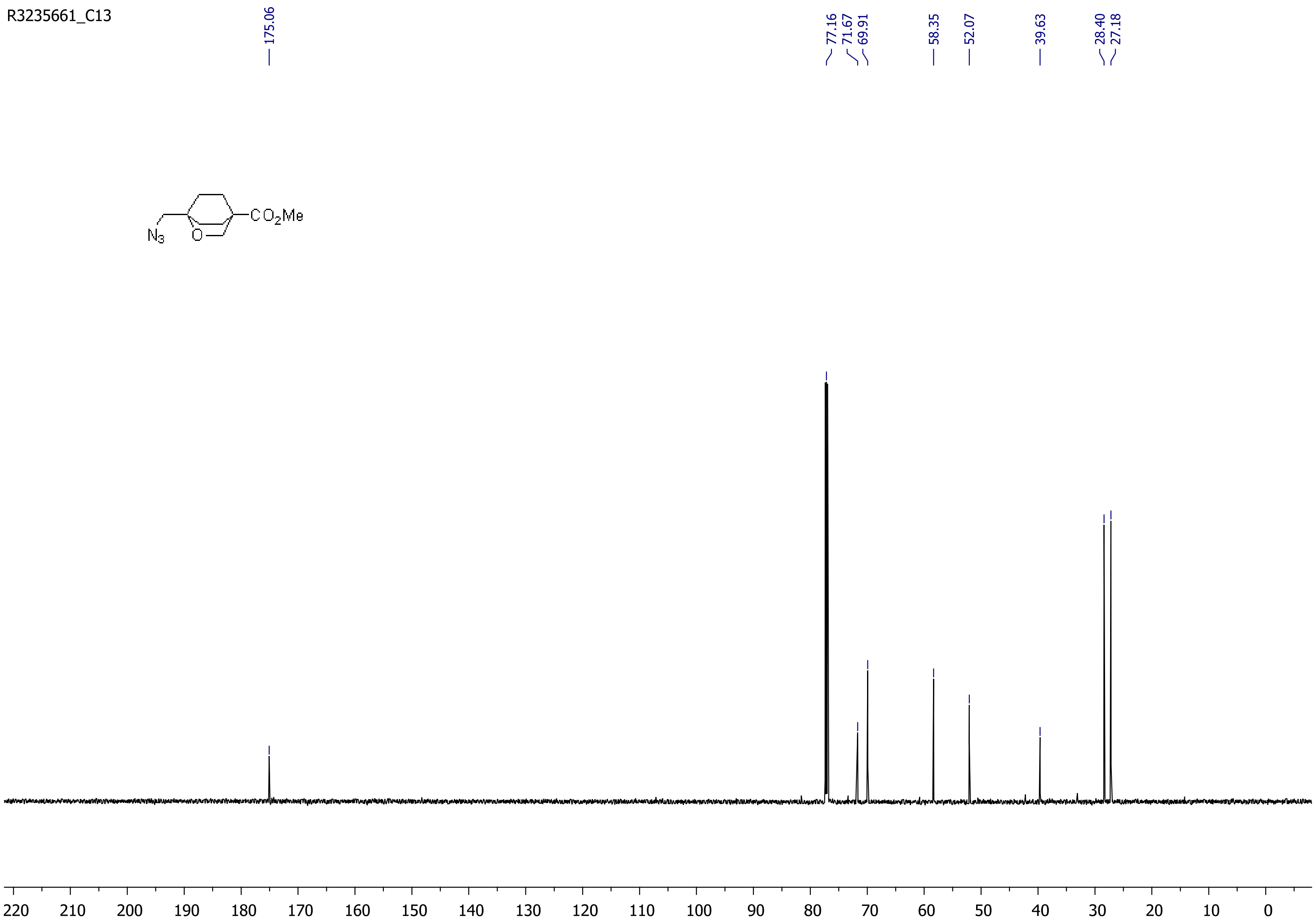

Compound 64

^{1}H NMR (500 MHz, DMSO-d$_6$)

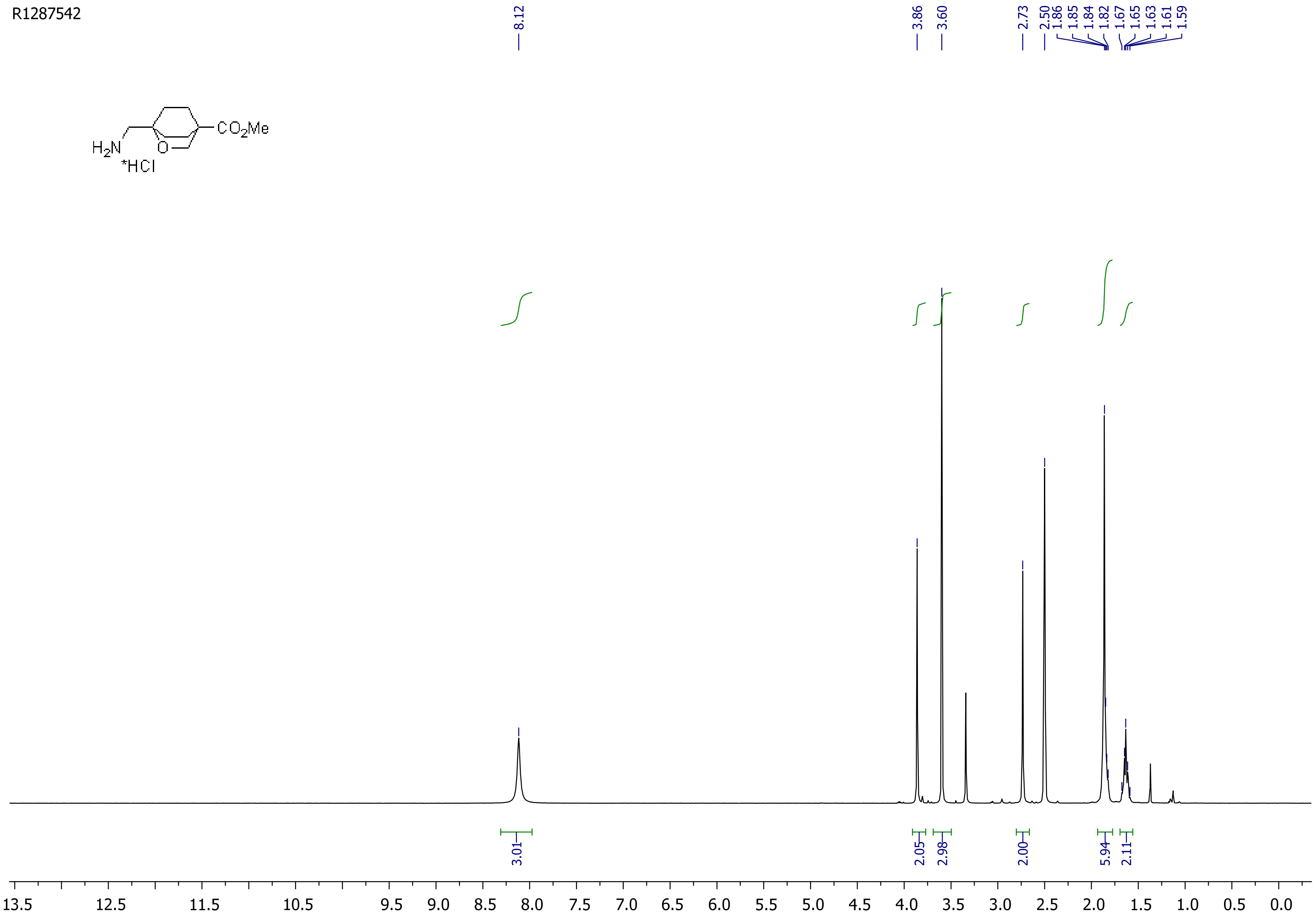

$^{13}C\{^1H\}$ NMR (126 MHz, DMSO-d6)

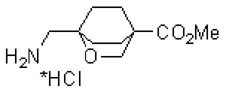

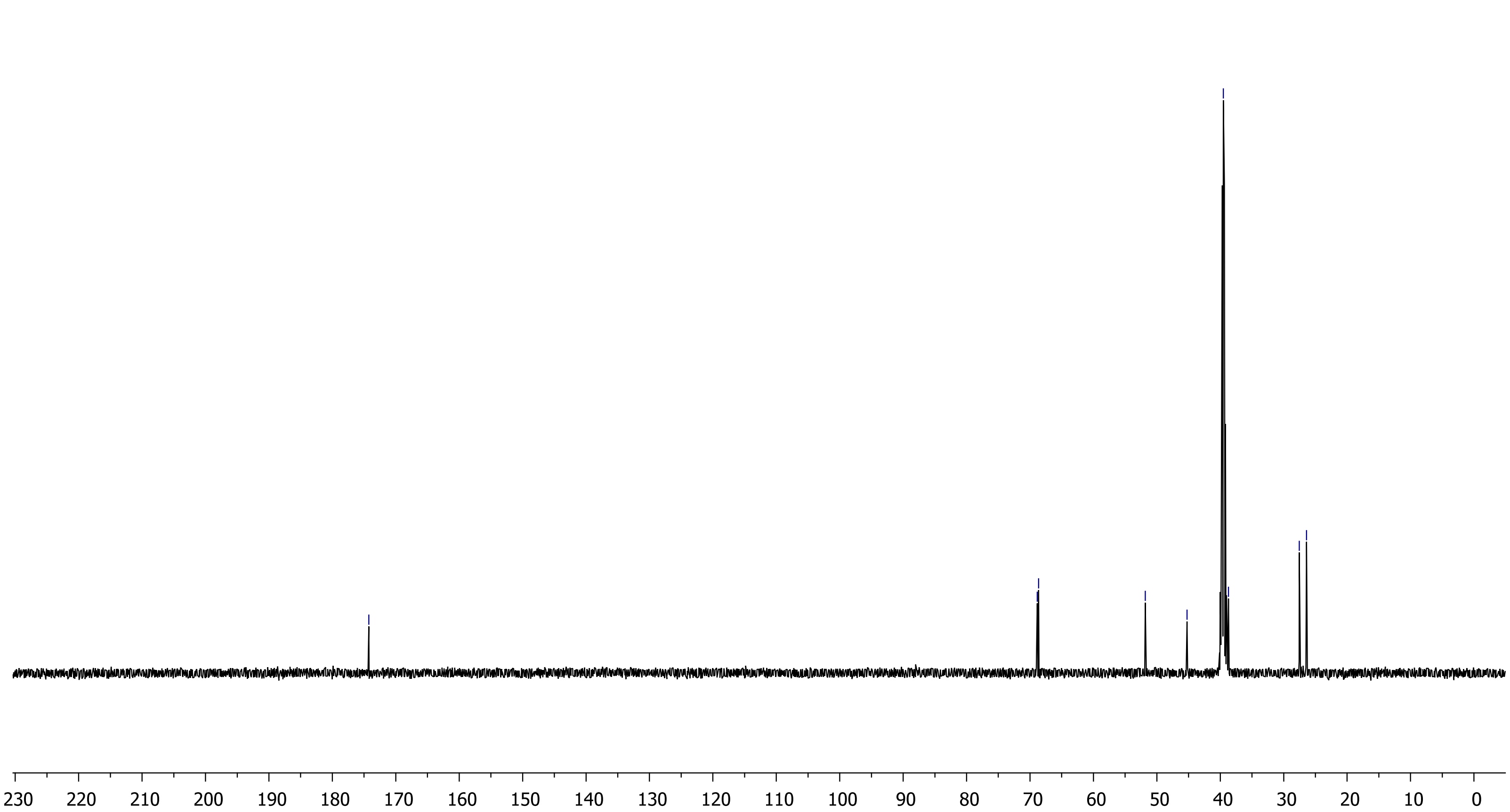

Compound SI-20

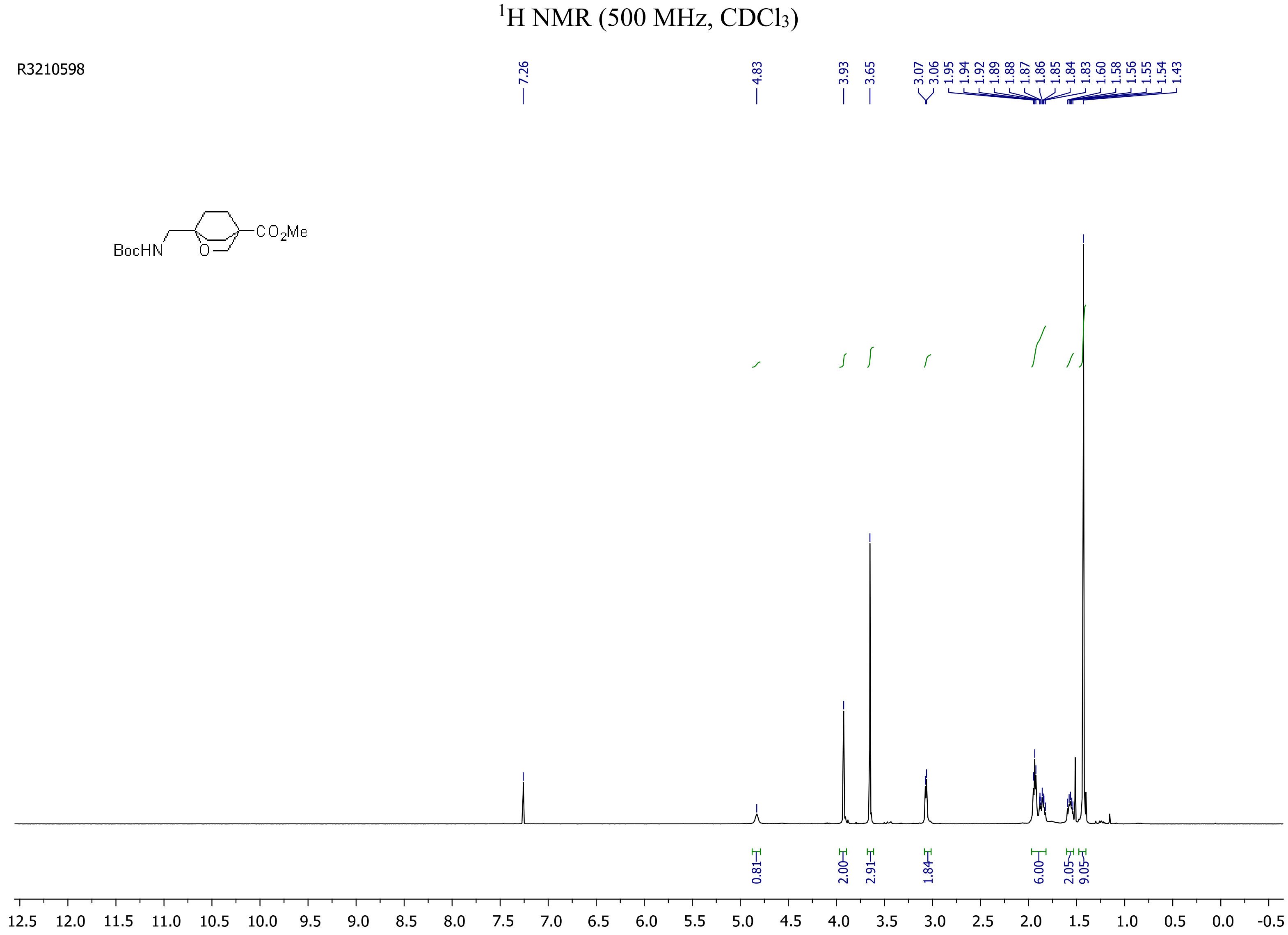

$^{13}C\{^1H\}$ NMR (151 MHz, $CDCl_3$)

R3210598_C13

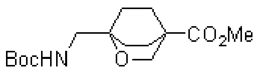

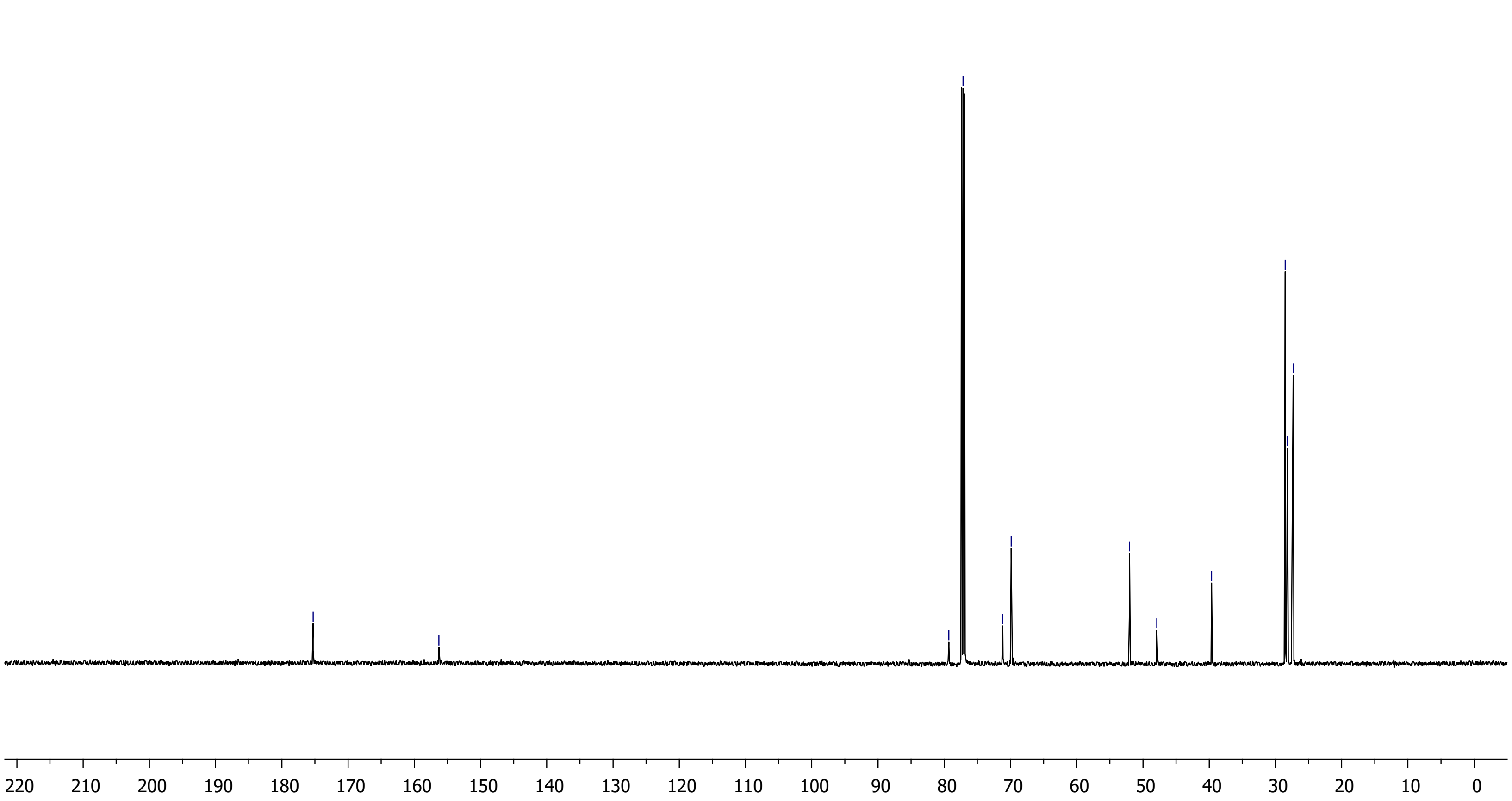

Compound 65

^1H NMR (500 MHz, $CDCl_3$)

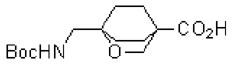

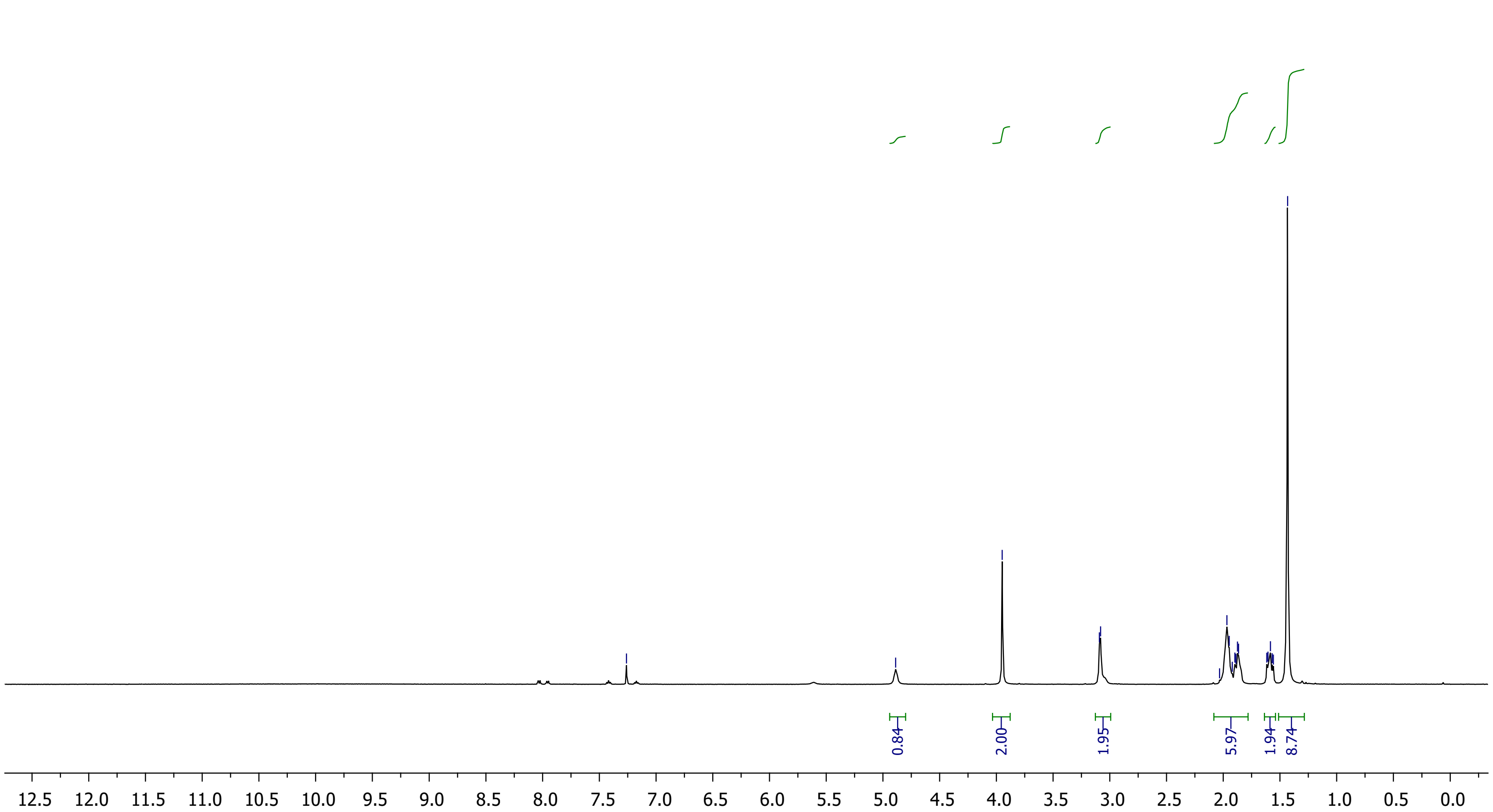

$^{13}C\{^1H\}$ NMR (151 MHz, $CDCl_3$)

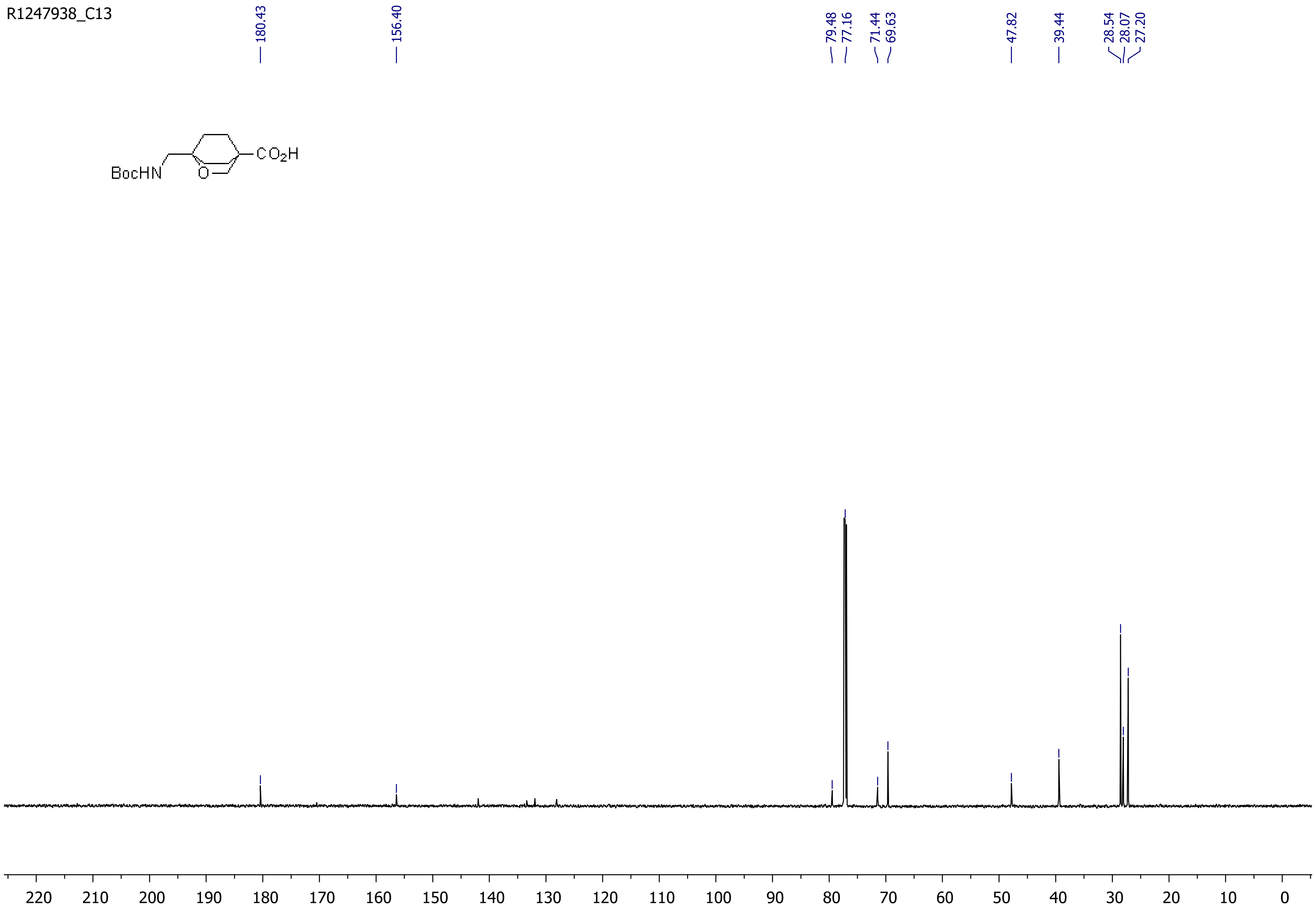

Compound SI-21

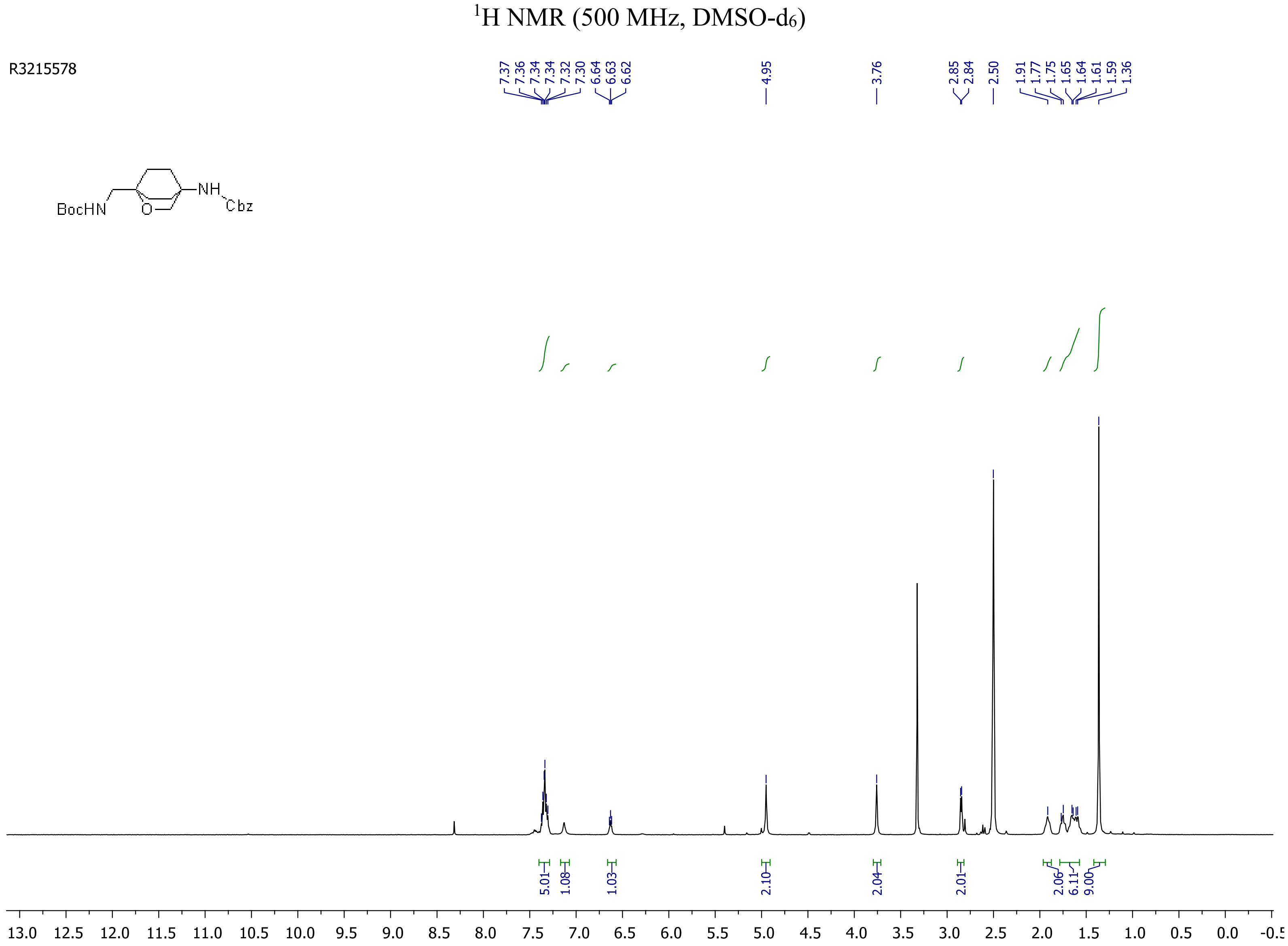

$^{13}C\{^1H\}$ NMR (126 MHz, DMSO-d6)

R3215578_C13

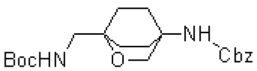

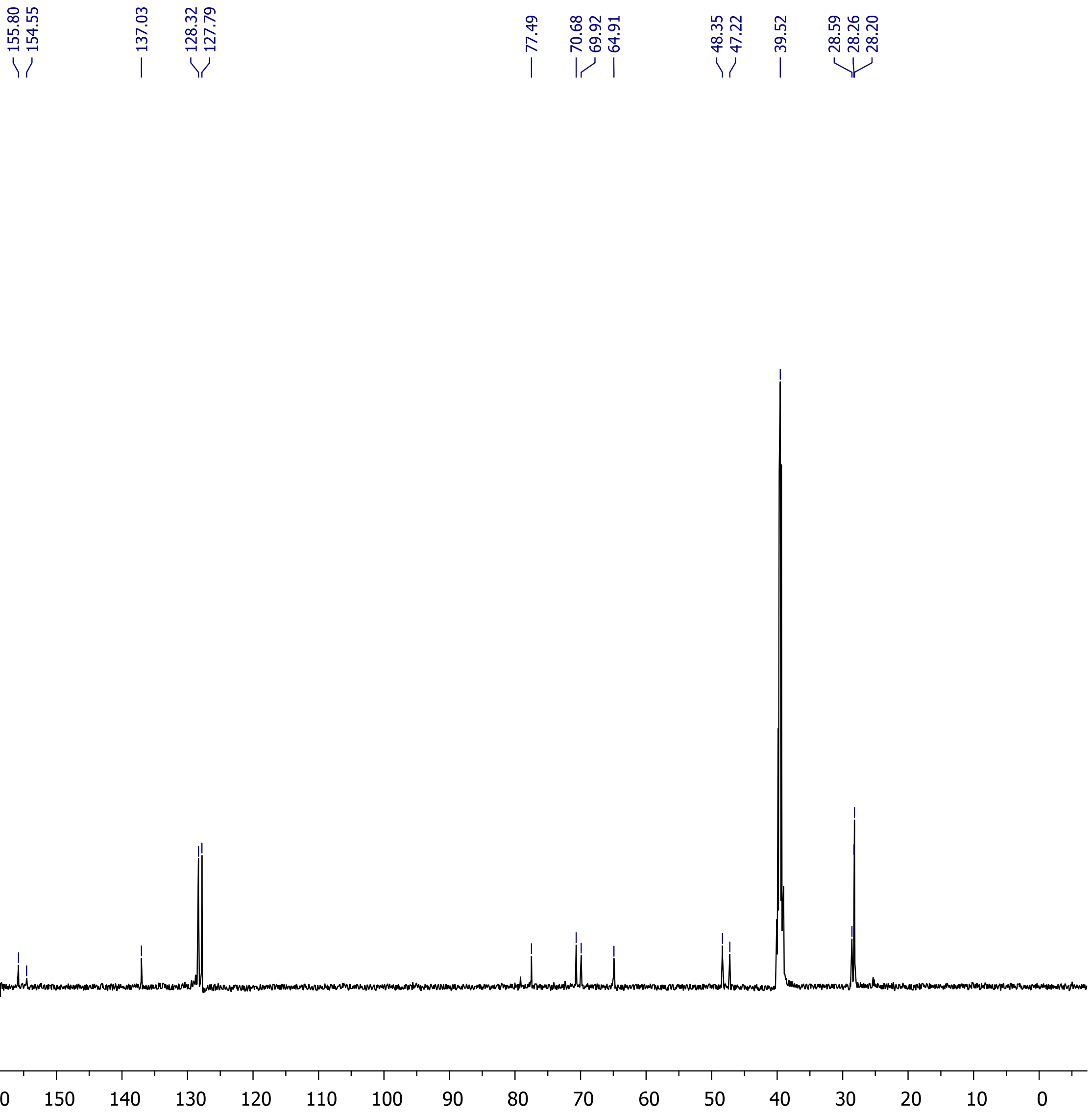

Compound 66

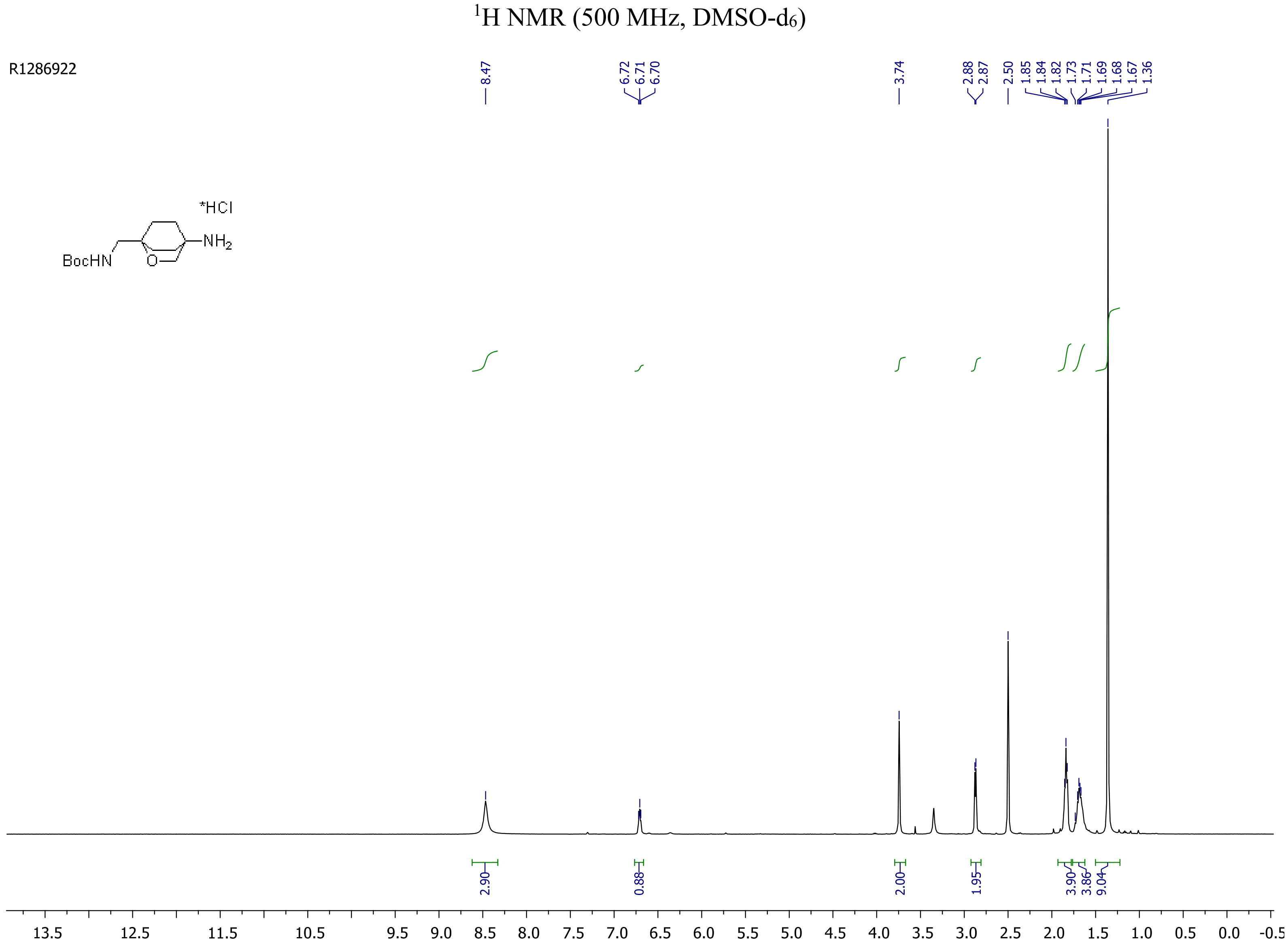

¹H NMR (500 MHz, DMSO-d6)
R1286922
*HCl
NH2
BocHN
O
8.47
6.72
6.71
6.70
3.74
2.88
2.87
2.50
1.85
1.84
1.82
1.73
1.71
1.69
1.68
1.67
1.36
2.90
0.88
2.00
1.95
3.90
3.86
9.04
13.5
12.5
11.5
10.5
9.5
9.0
8.5
8.0
7.5
7.0
6.5
6.0
5.5
5.0
4.5
4.0
3.5
3.0
2.5
2.0
1.5
1.0
0.5
0.0
-0.5

$^{13}C\{^{1}H\}$ NMR (126 MHz, DMSO-d6)

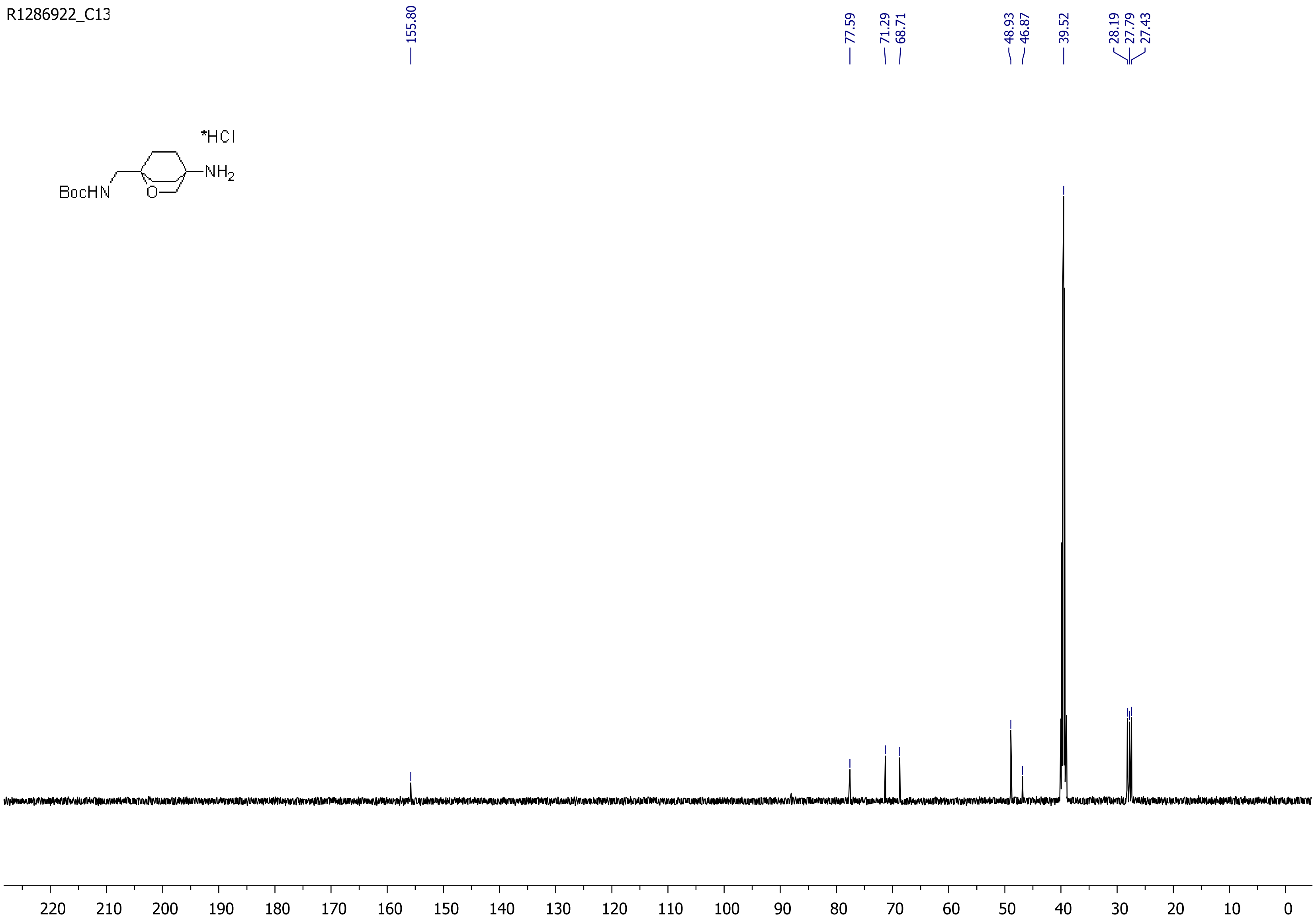

Compound SI-22

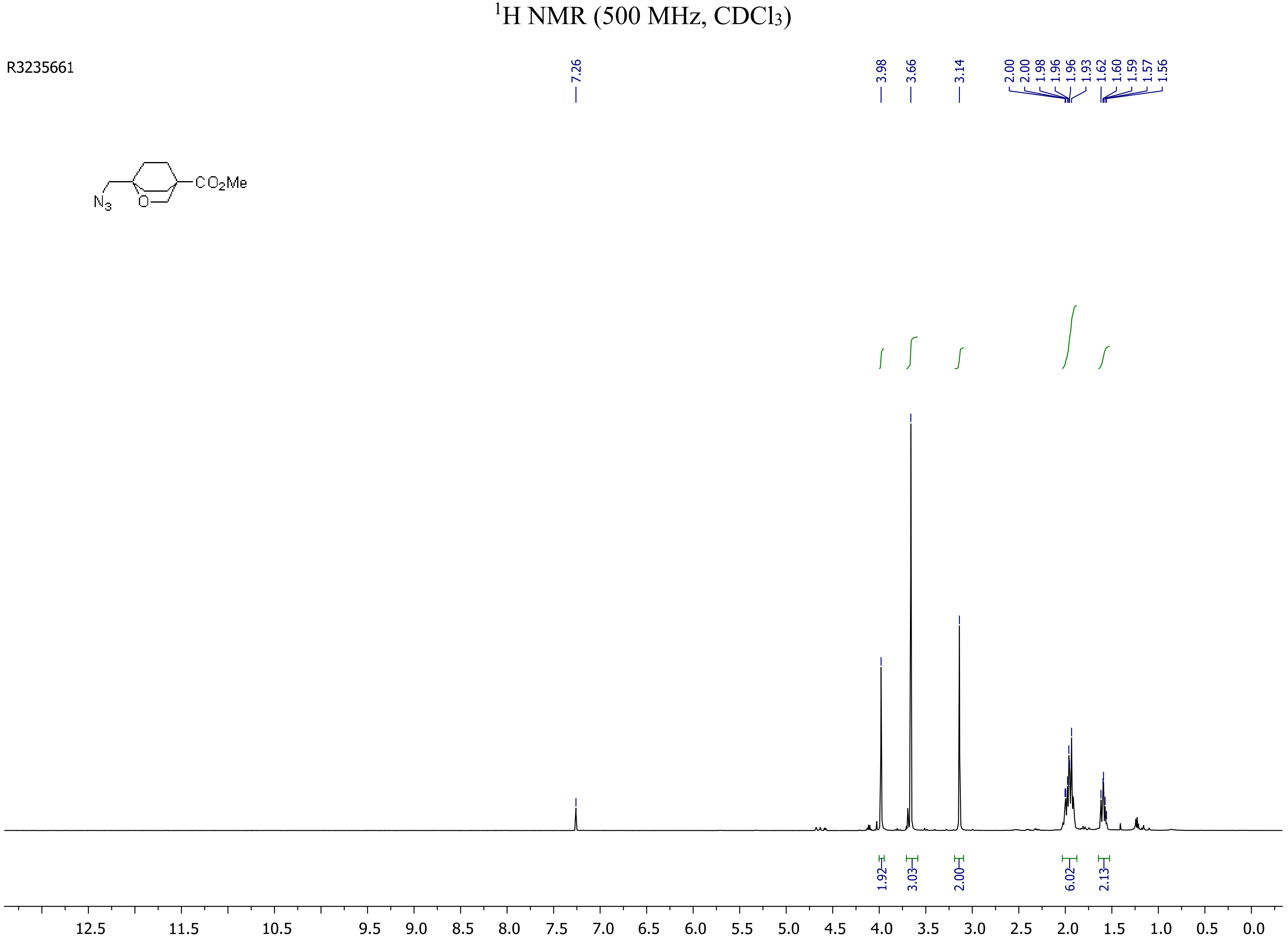

$^{13}C\{^1H\}$ NMR (151 MHz, $CDCl_3$)

R3235661_C13

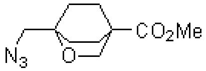

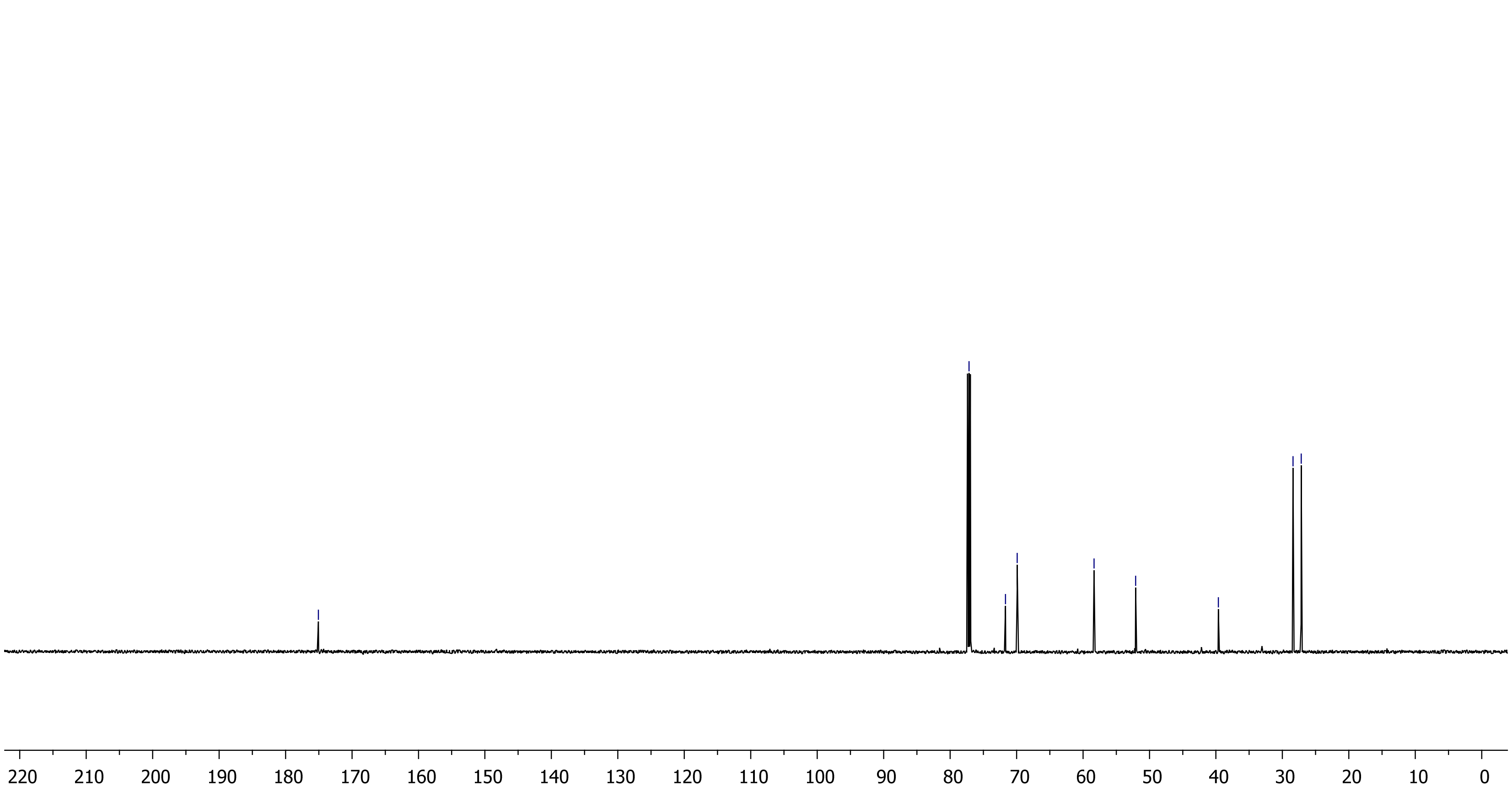

Compound 67

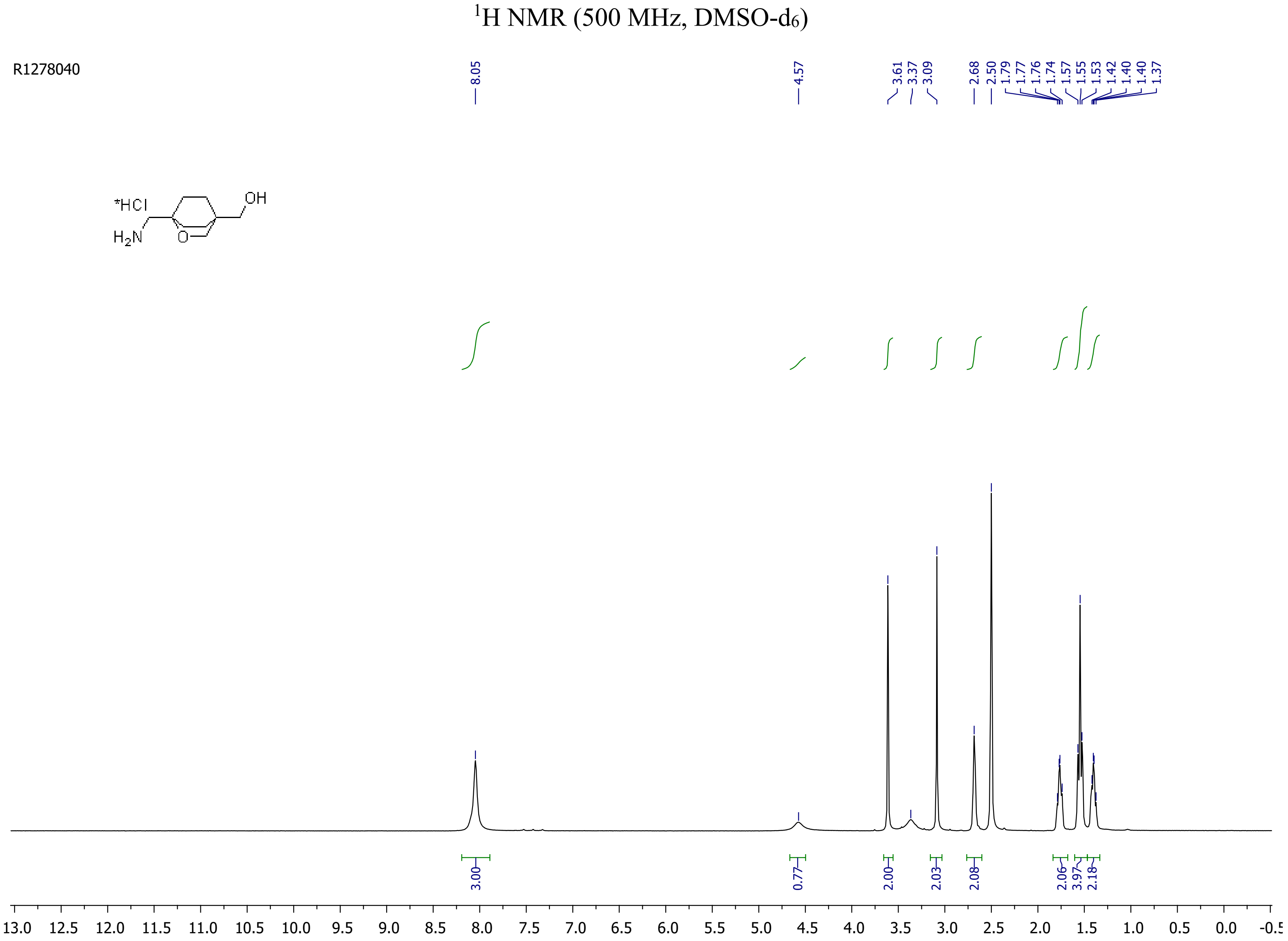

¹H NMR (500 MHz, DMSO-d6)
R1278040
8.05
4.57
3.61
3.37
3.09
2.68
2.50
1.79
1.77
1.76
1.74
1.57
1.55
1.53
1.42
1.40
1.37
*HCl
OH
H2N
O
3.00
0.77
2.00
2.03
2.08
2.06
3.97
2.18
13.0 12.5 12.0 11.5 11.0 10.5 10.0 9.5 9.0 8.5 8.0 7.5 7.0 6.5 6.0 5.5 5.0 4.5 4.0 3.5 3.0 2.5 2.0 1.5 1.0 0.5 0.0 -0.5

$^{13}C\{^1H\}$ NMR (126 MHz, $CDCl_3$)

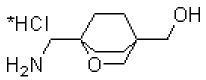

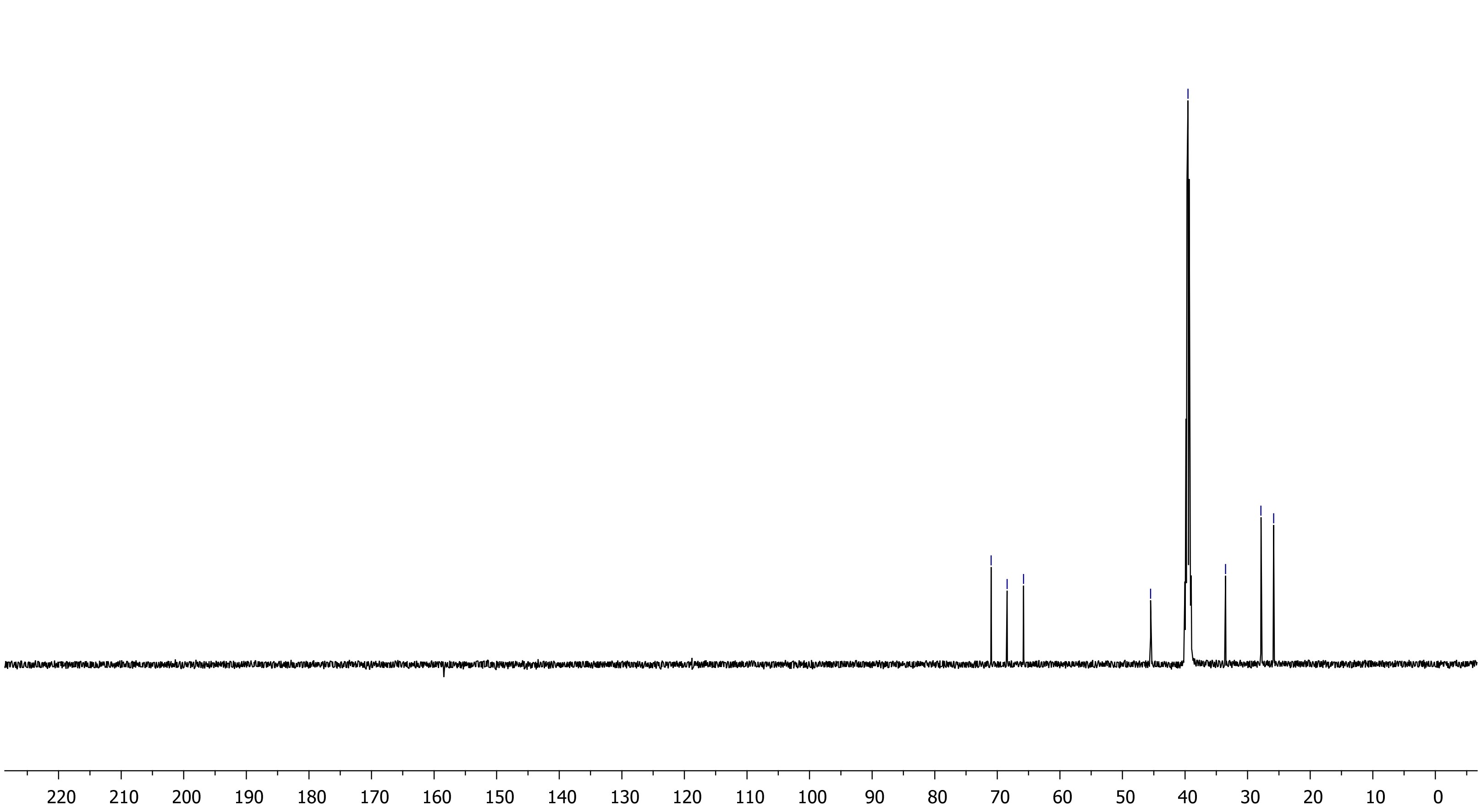

Compound 68

^{1}H NMR (500 MHz, $CDCl_3$)

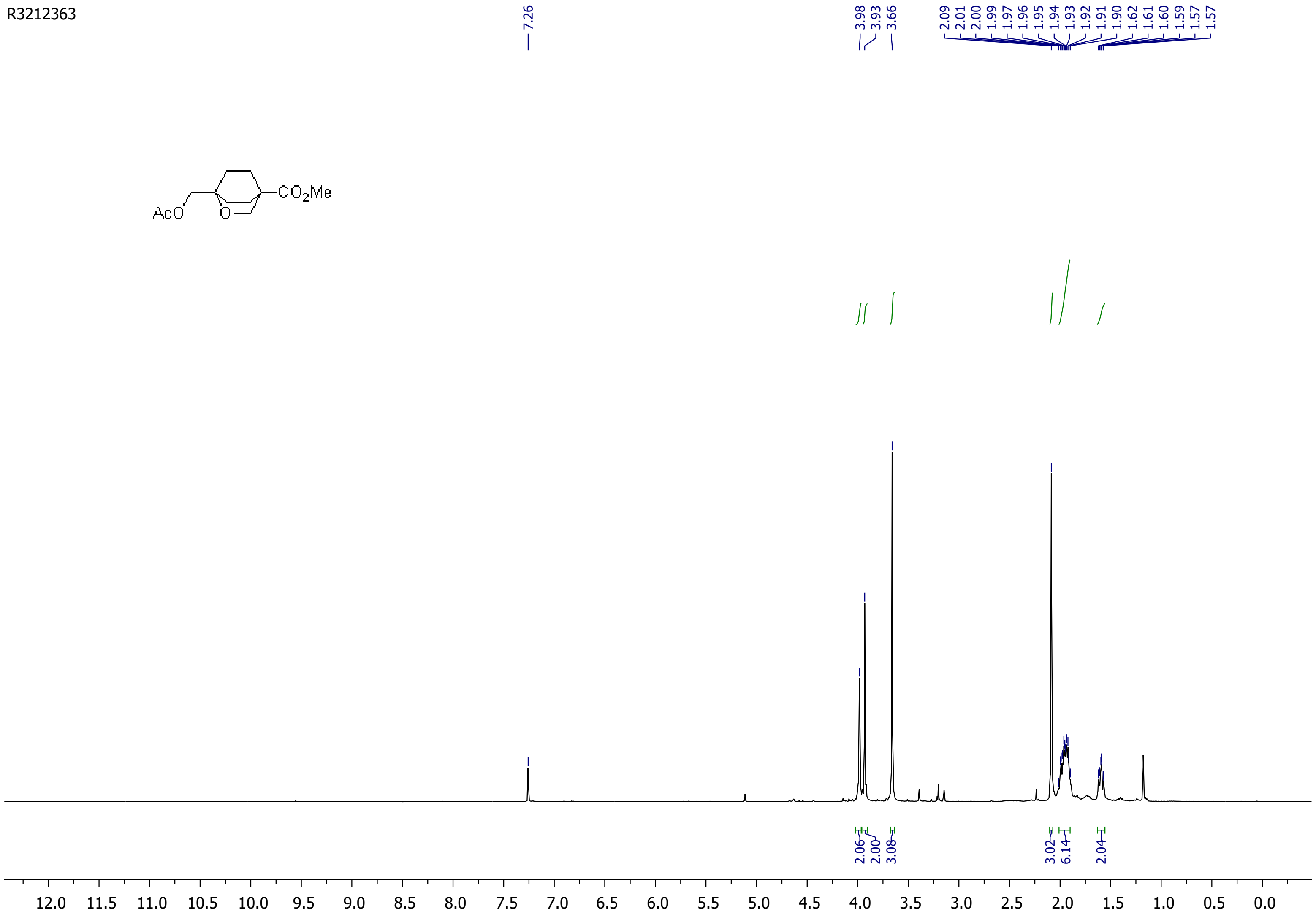

$^{13}C\{^1H\}$ NMR (126 MHz, $CDCl_3$)

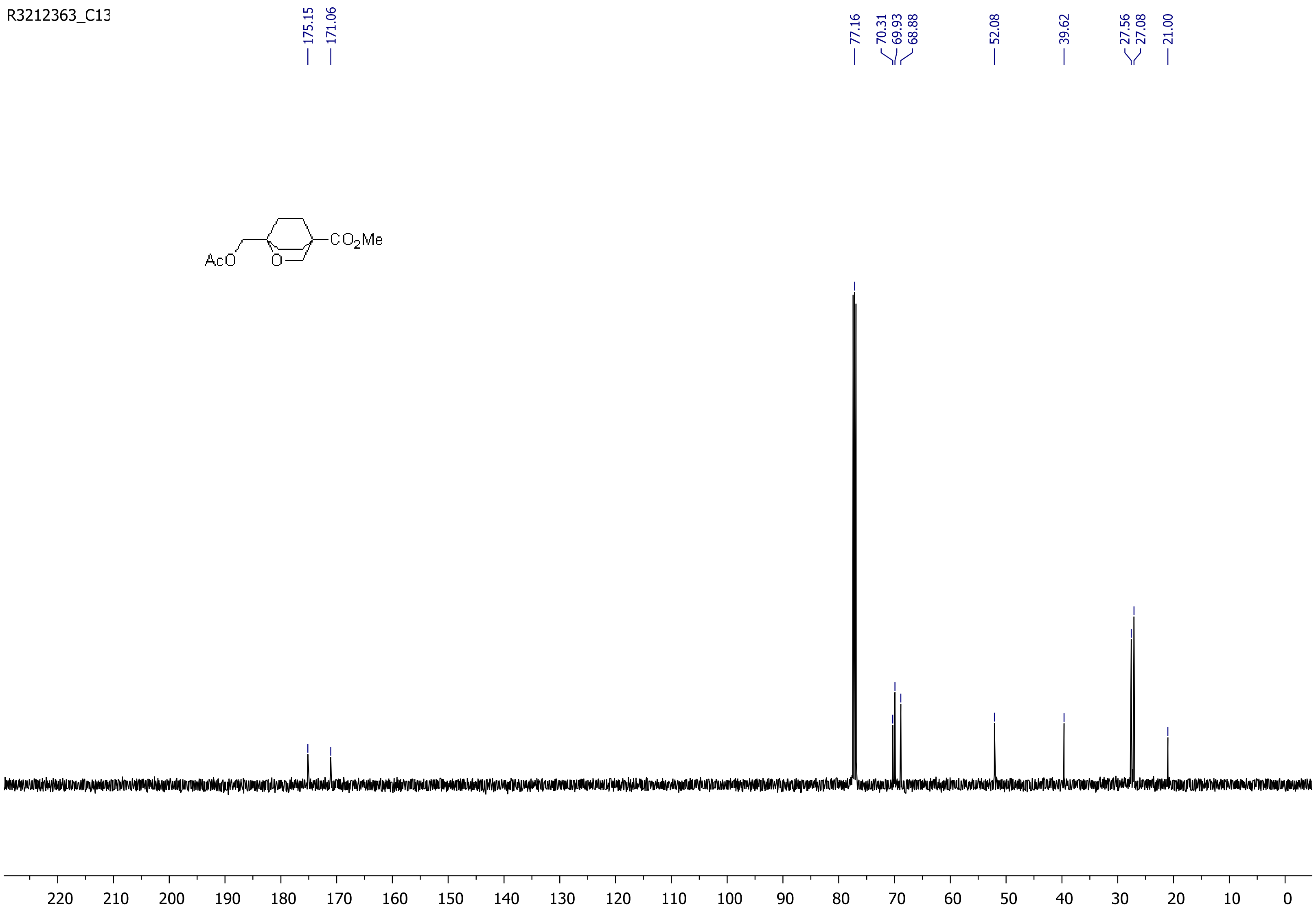

Compound SI-23

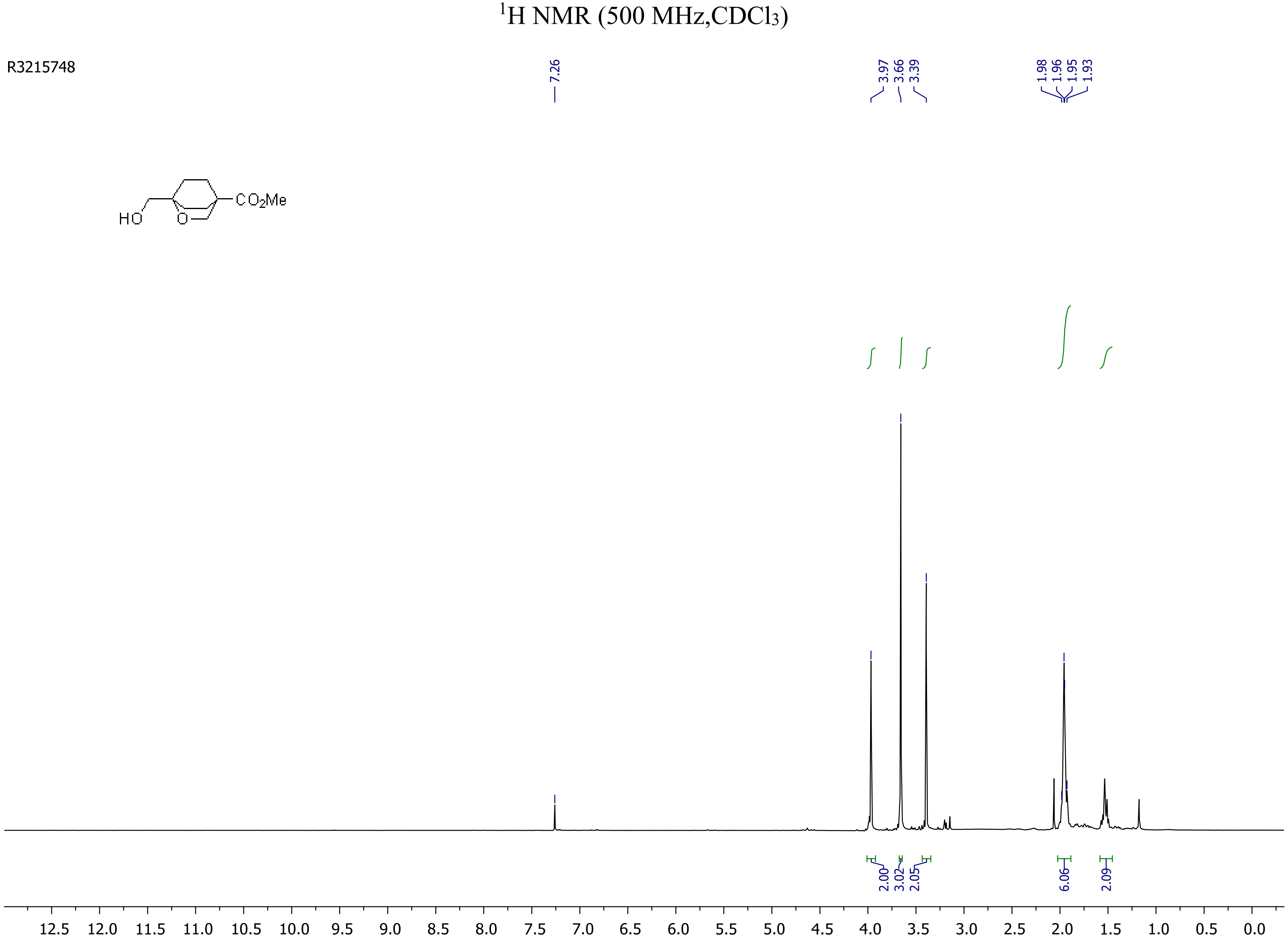

$^{13}C\{^1H\}$ NMR (151 MHz, $CDCl_3$)

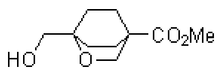

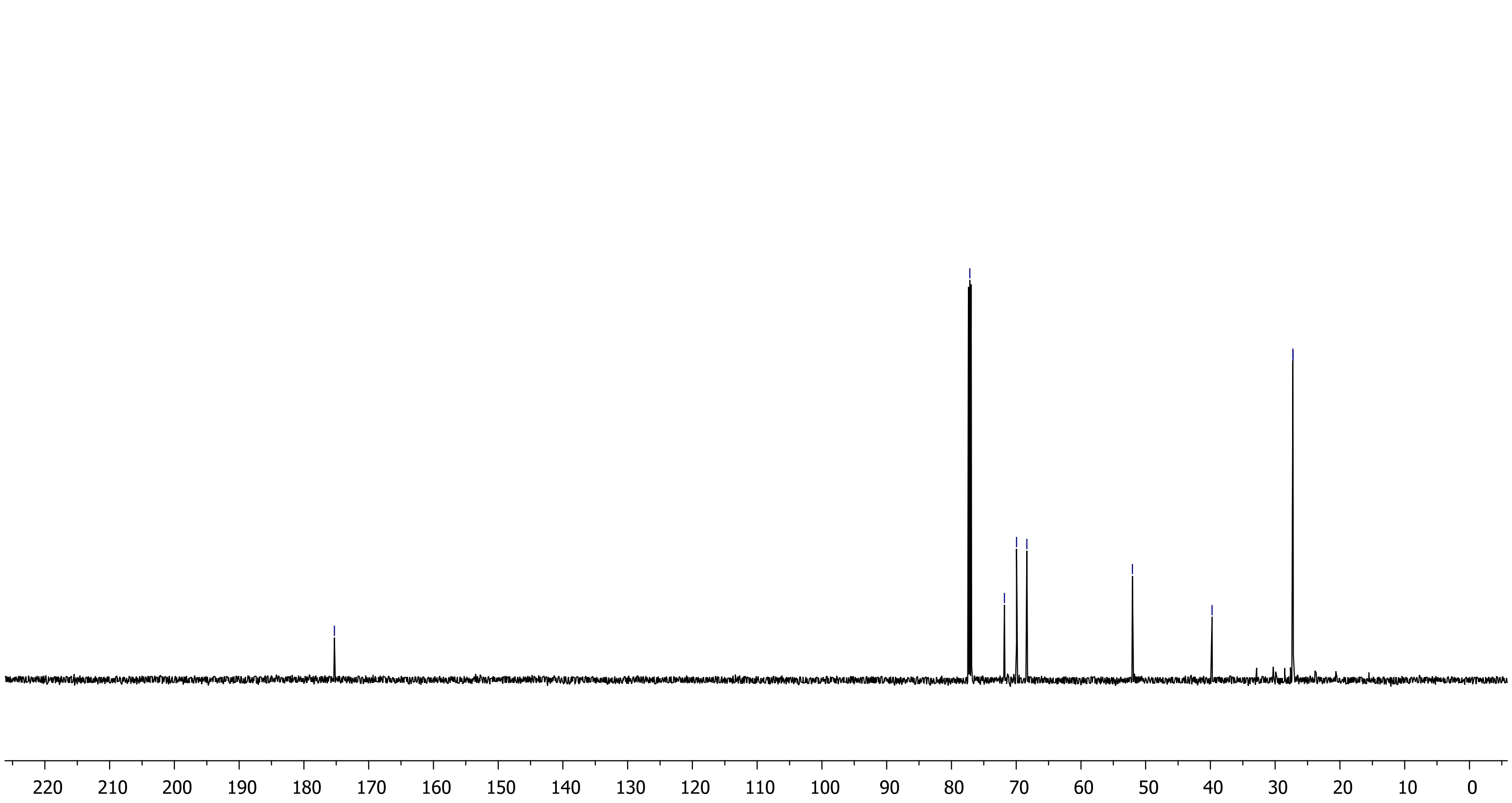

Compound 69

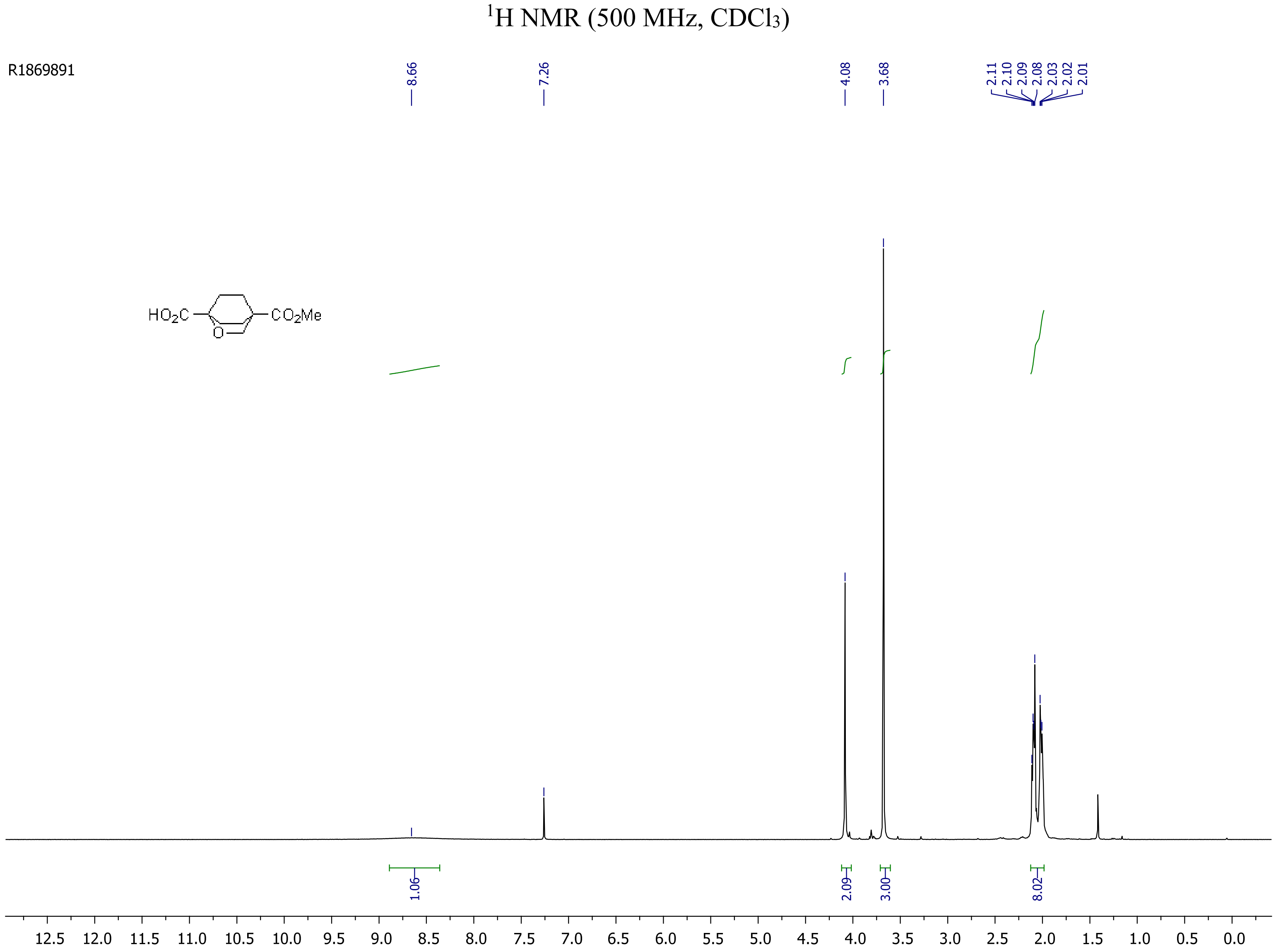

^{1}H NMR (500 MHz, CDCl$_3$)
R1869891
8.66
7.26
4.08
3.68
2.11
2.10
2.09
2.08
2.03
2.02
2.01
HO2C
CO2Me
O
1.06
2.09
3.00
8.02
12.5
12.0
11.5
11.0
10.5
10.0
9.5
9.0
8.5
8.0
7.5
7.0
6.5
6.0
5.5
5.0
4.5
4.0
3.5
3.0
2.5
2.0
1.5
1.0
0.5
0.0

$^{13}C\{^1H\}$ NMR (101 MHz, $CDCl_3$)

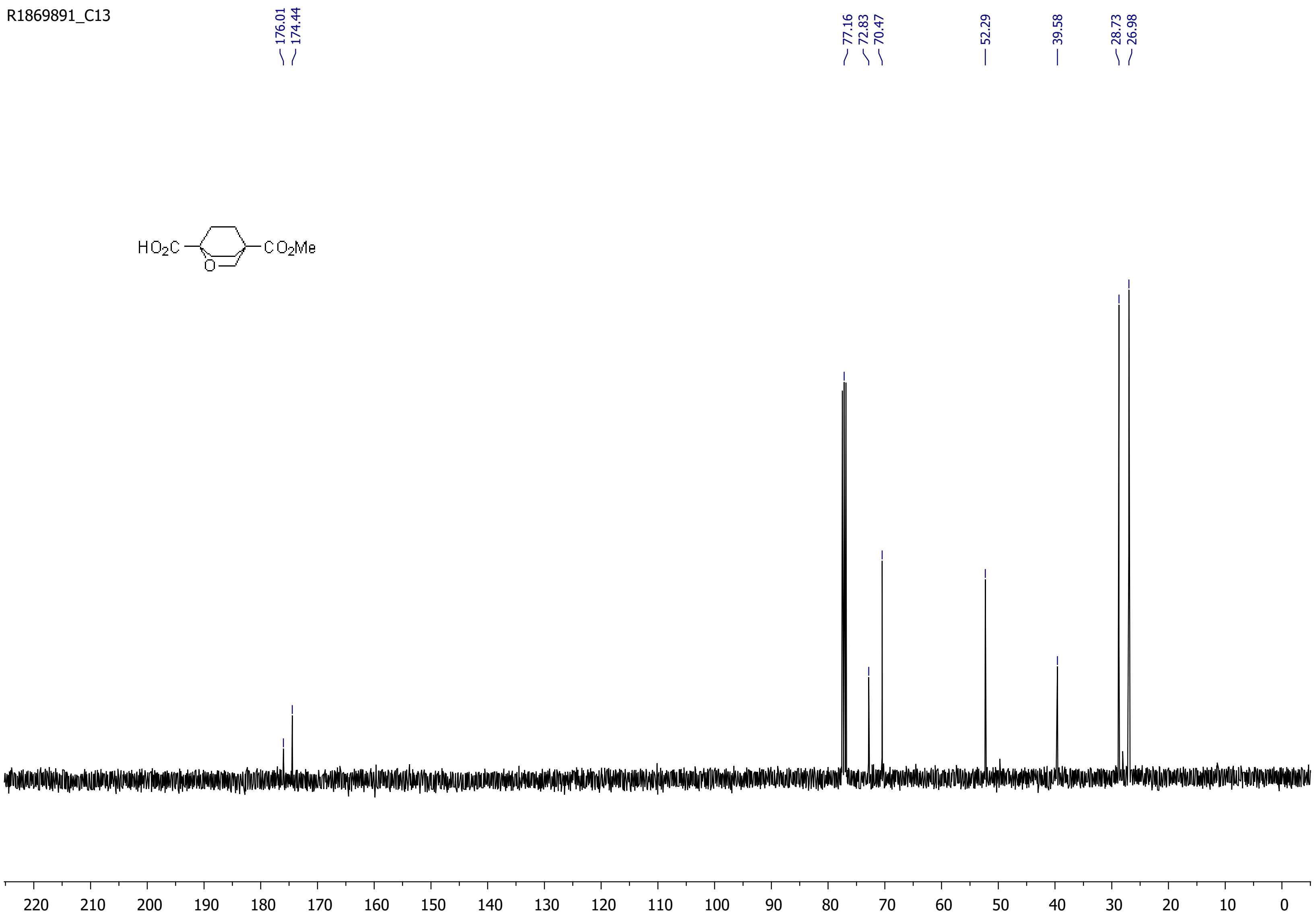

Compound 70

^{1}H NMR (500 MHz, DMSO-d$_6$)

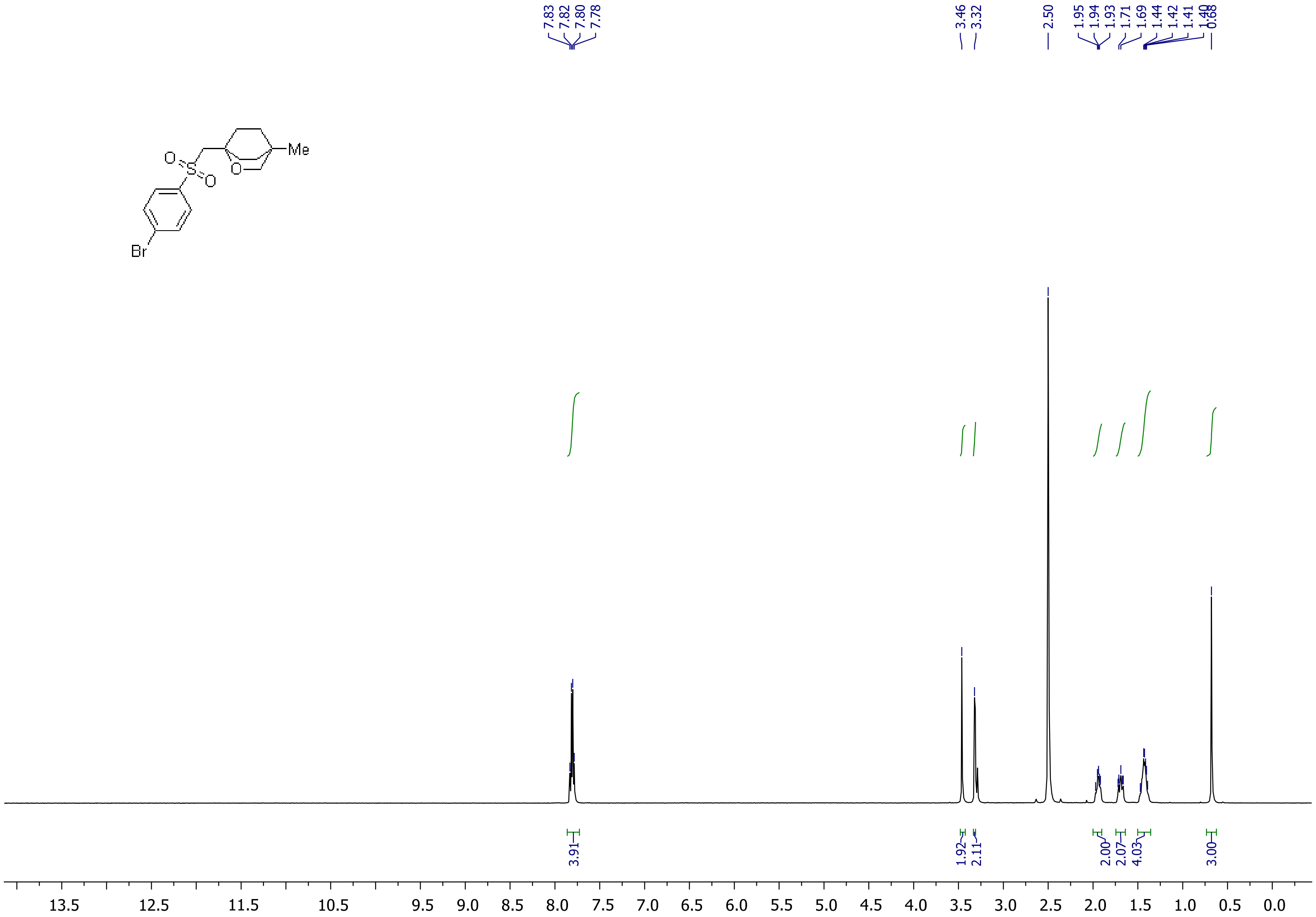

$^{13}C\{^1H\}$ NMR (151 MHz, $CDCl_3$)

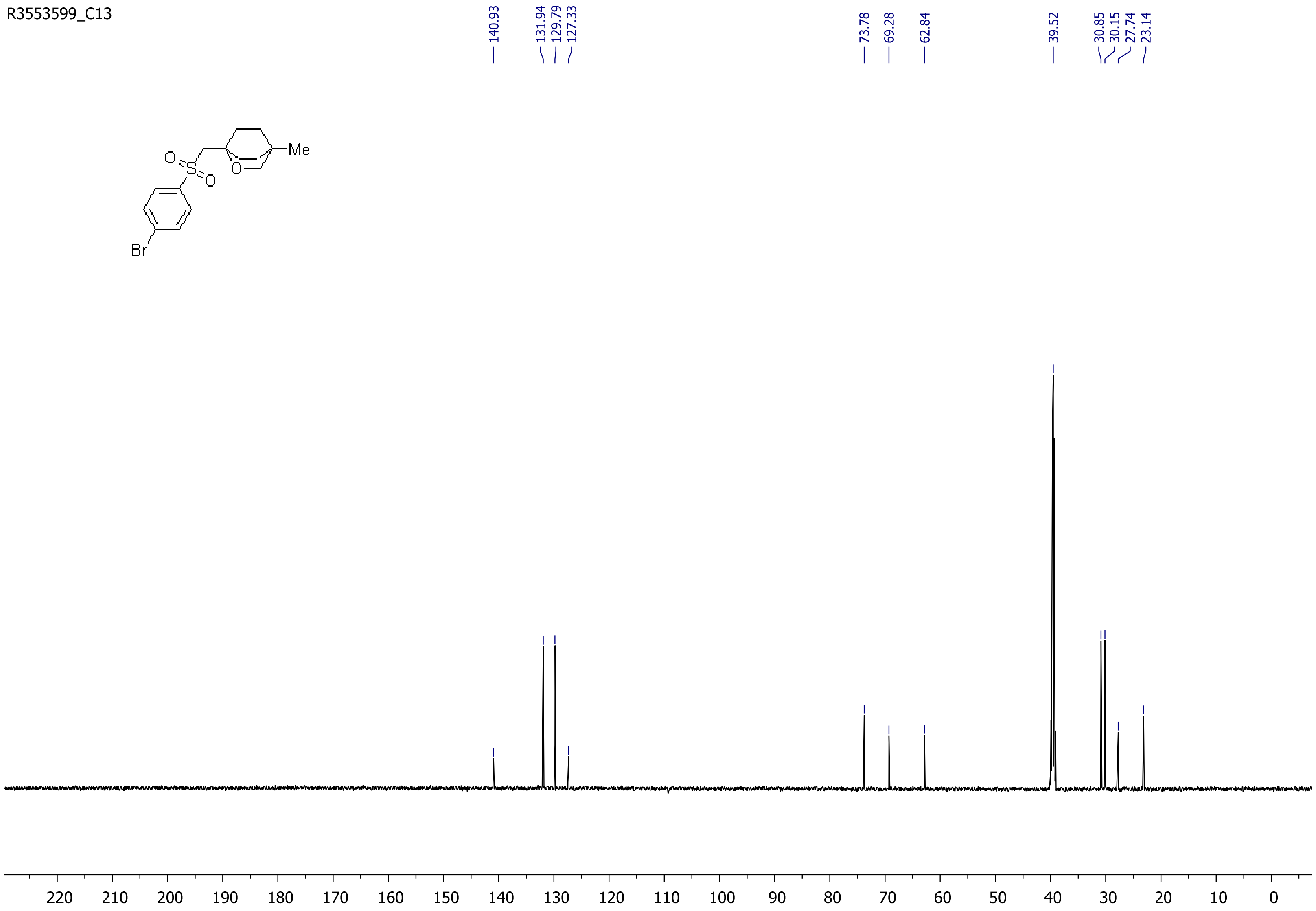

Compound 71

^{1}H NMR (500 MHz, DMSO-d6)

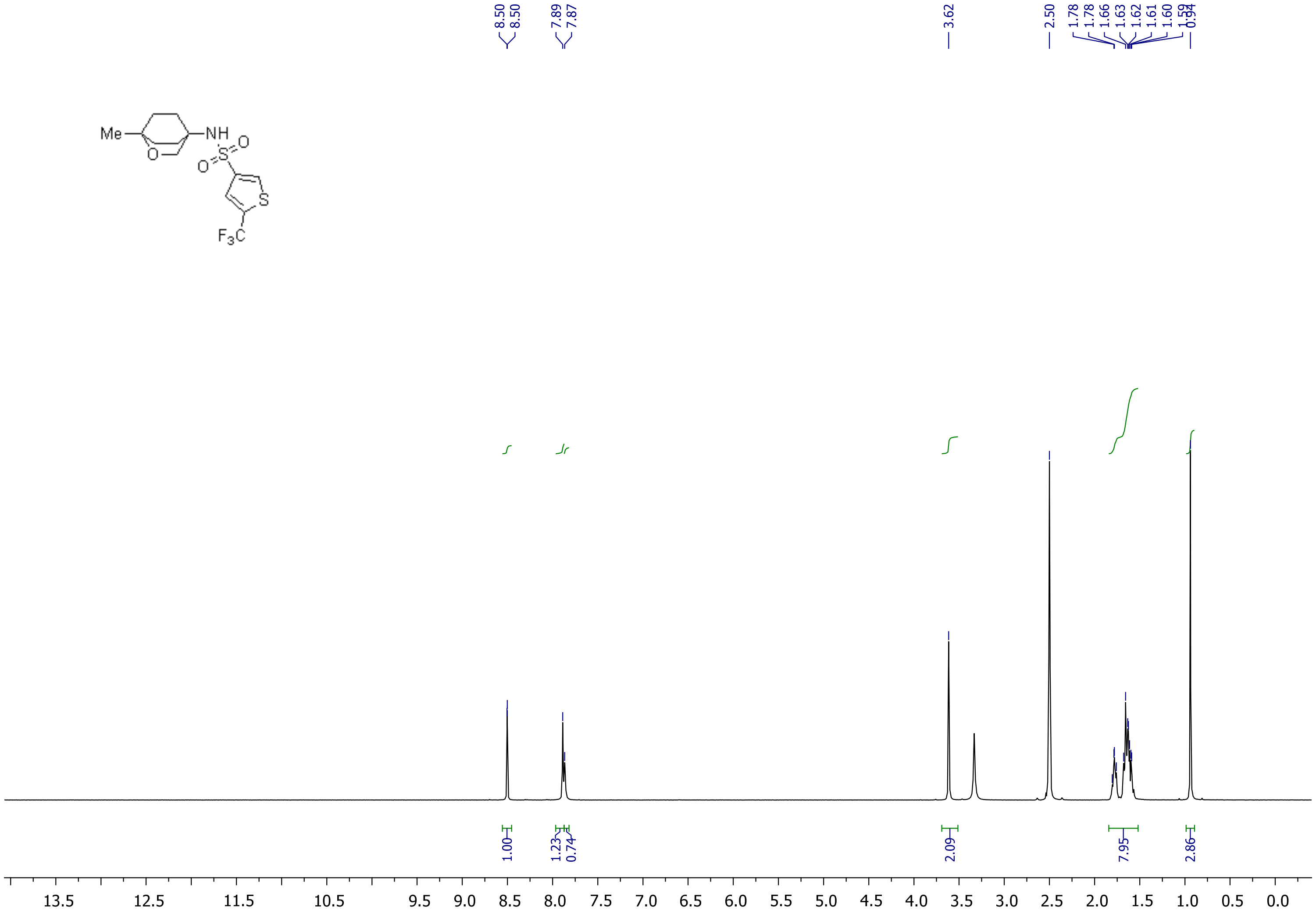

$^{13}C\{^{1}H\}$ NMR (151 MHz, DMSO-d6)

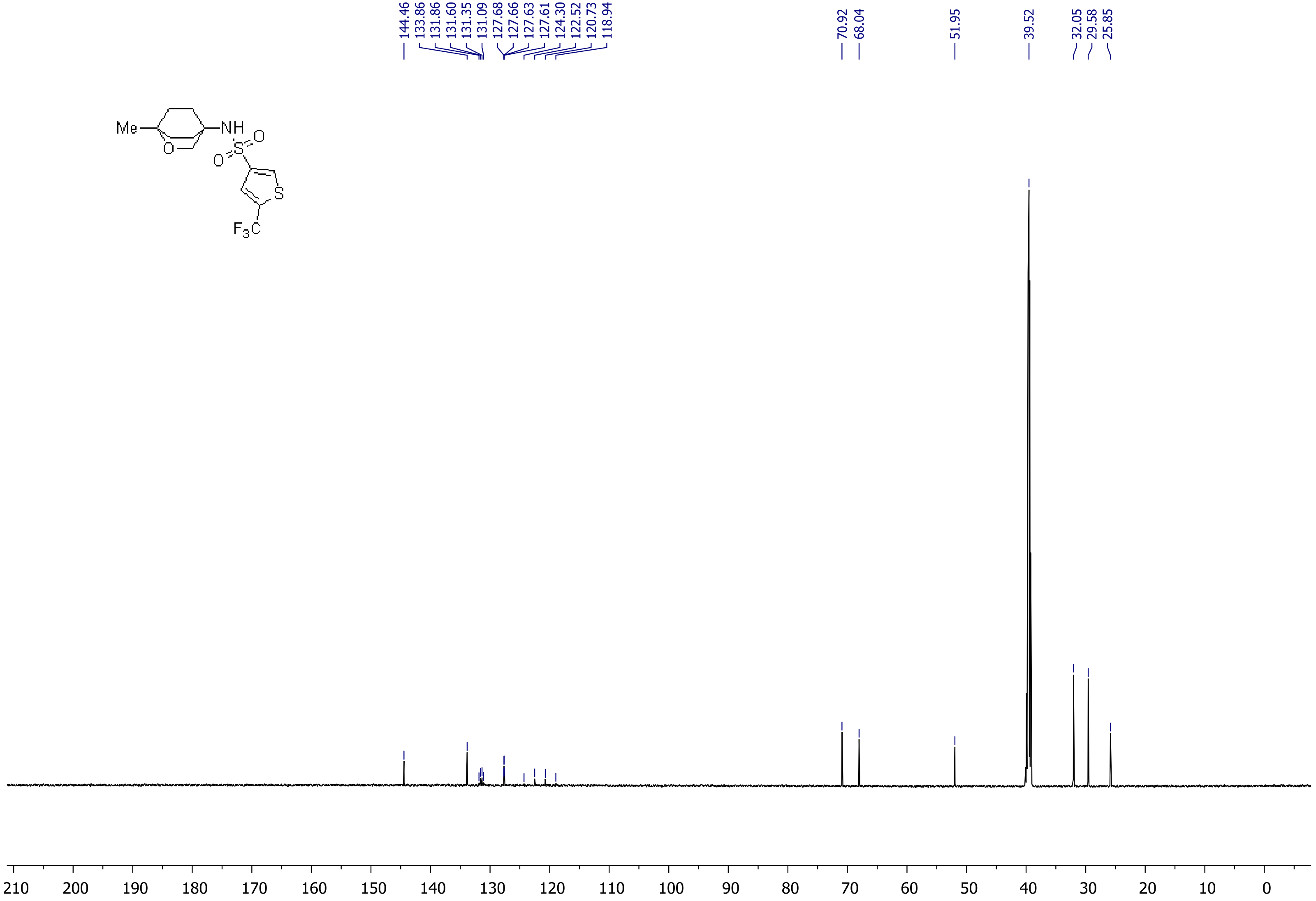

$^{19}F\{^1H\}$ NMR (376 MHz, DMSO-d6)

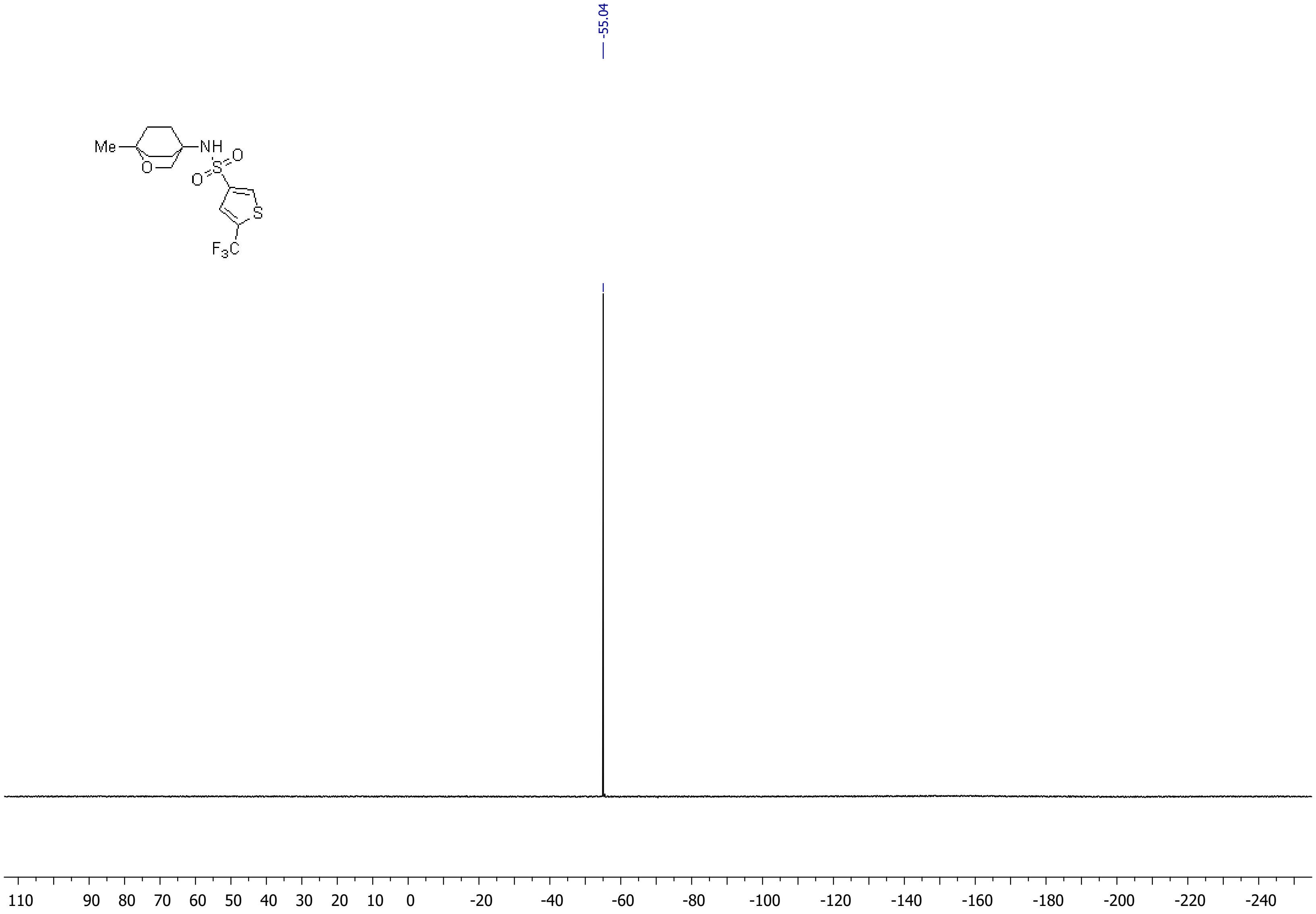

Compound 72

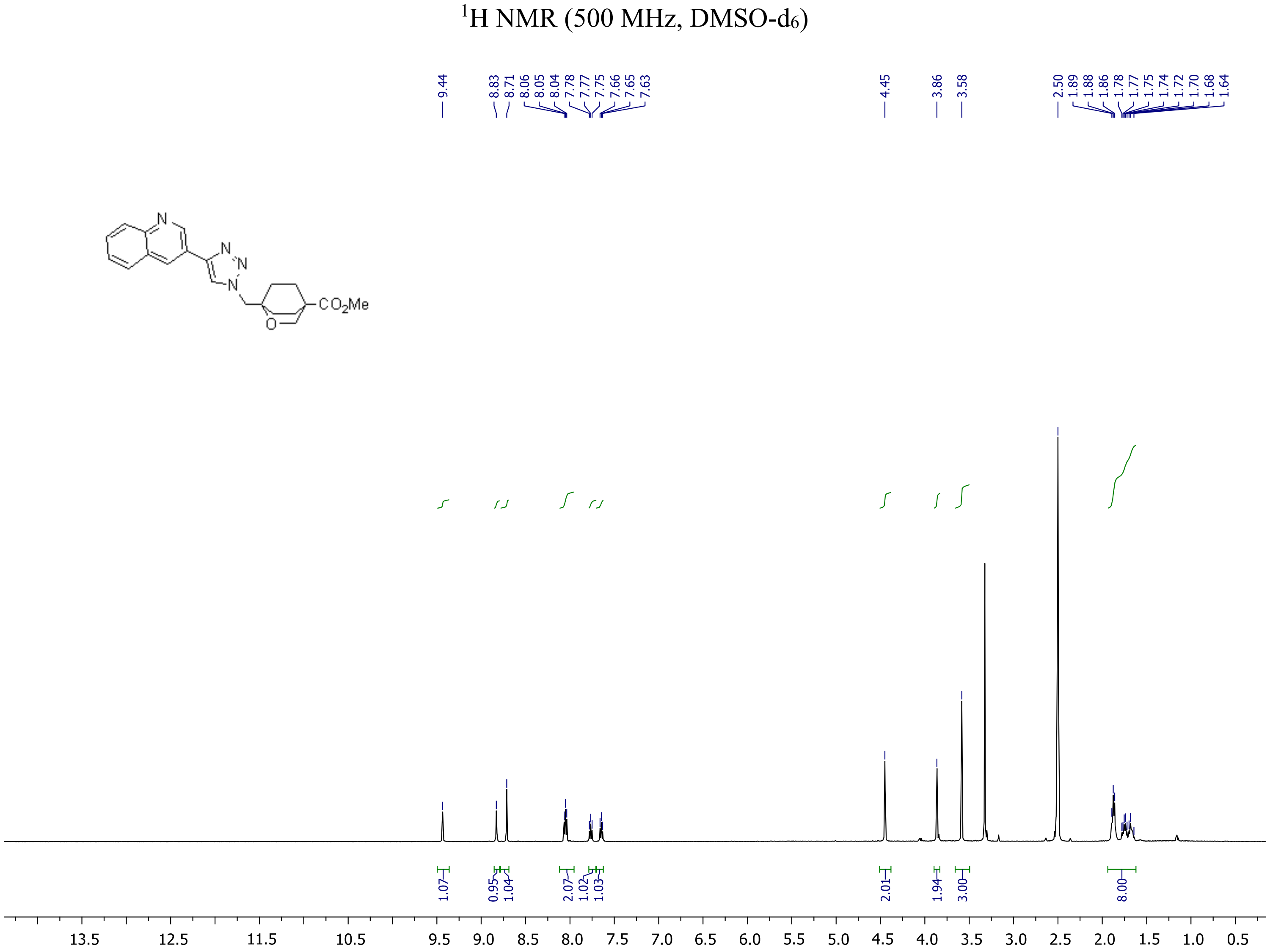

N
N
N
N
CO2Me
O
1H NMR (500 MHz, DMSO-d6)
9.44
8.83
8.71
8.06
8.05
8.04
7.78
7.77
7.75
7.66
7.65
7.63
4.45
3.86
3.58
2.50
1.89
1.88
1.86
1.78
1.77
1.75
1.74
1.72
1.70
1.68
1.64
1.07
0.95
1.04
2.07
1.02
1.03
2.01
1.94
3.00
8.00
13.5
12.5
11.5
10.5
9.5
9.0
8.5
8.0
7.5
7.0
6.5
6.0
5.5
5.0
4.5
4.0
3.5
3.0
2.5
2.0
1.5
1.0
0.5

$^{13}C\{^1H\}$ NMR (151 MHz, DMSO-d6)

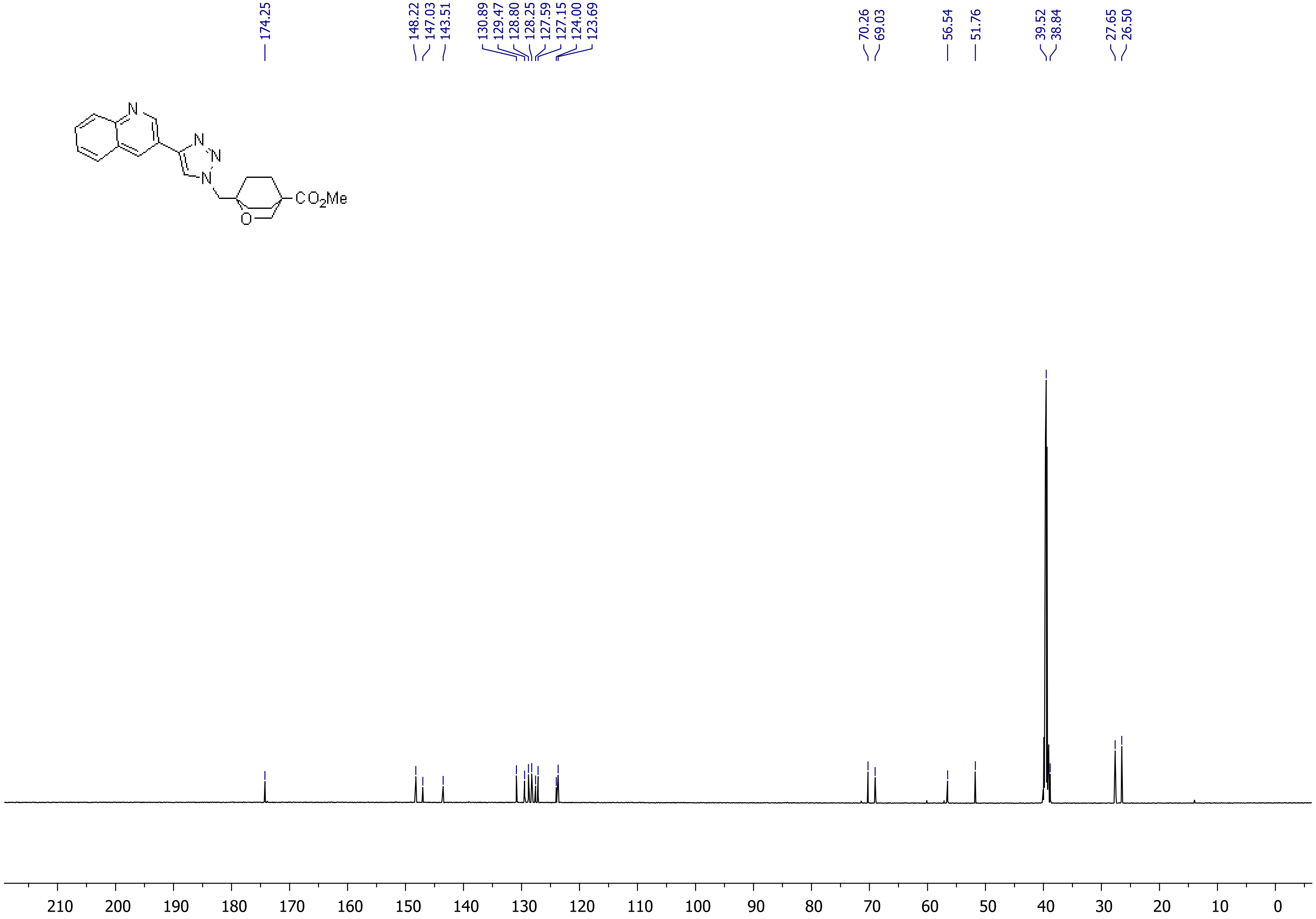

Compound 74

^{1}H NMR (500 MHz, DMSO-d$_6$)

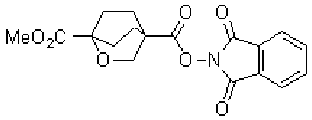

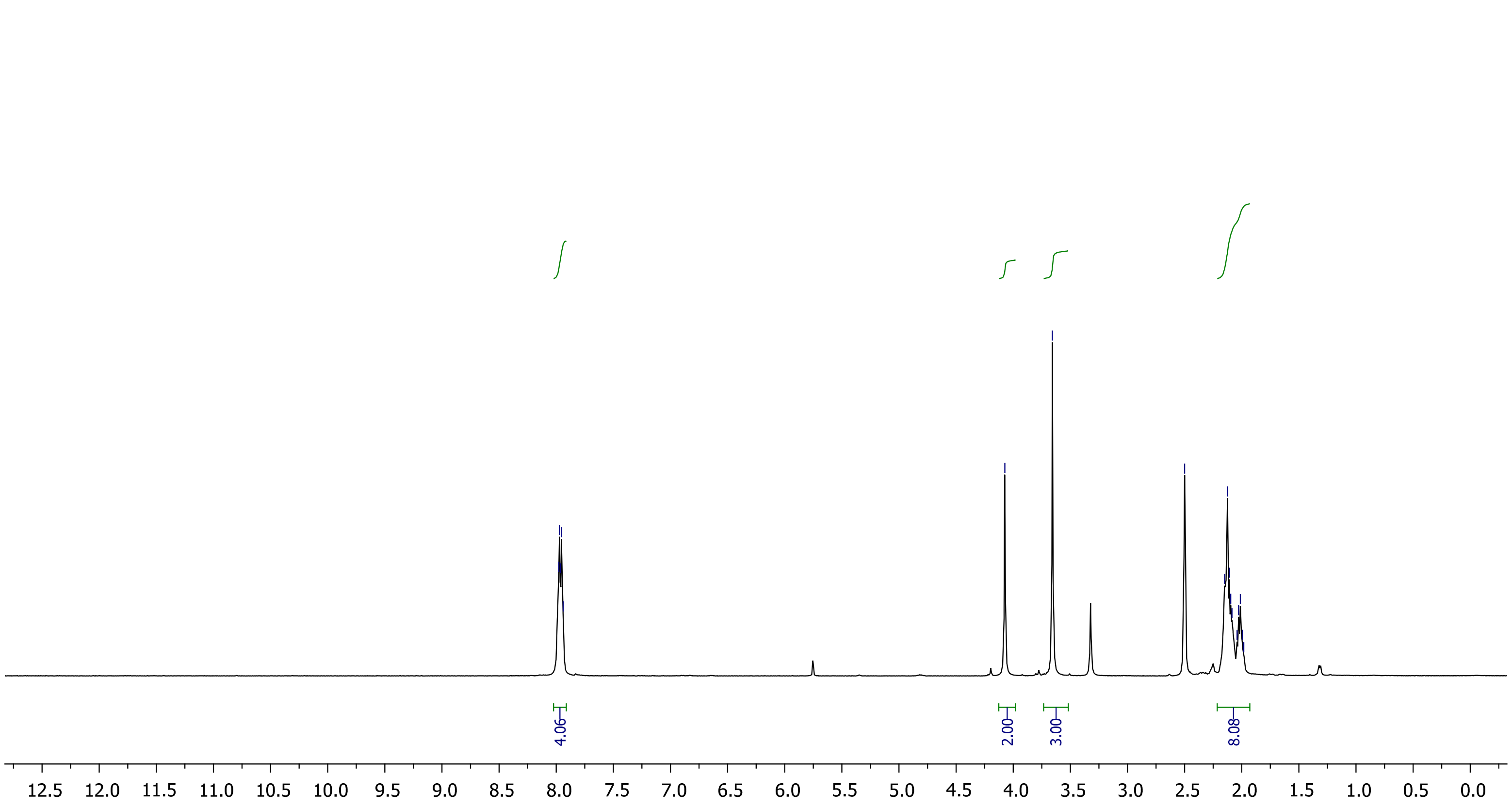

$^{13}C\{^1H\}$ NMR (151 MHz, DMSO-d6)

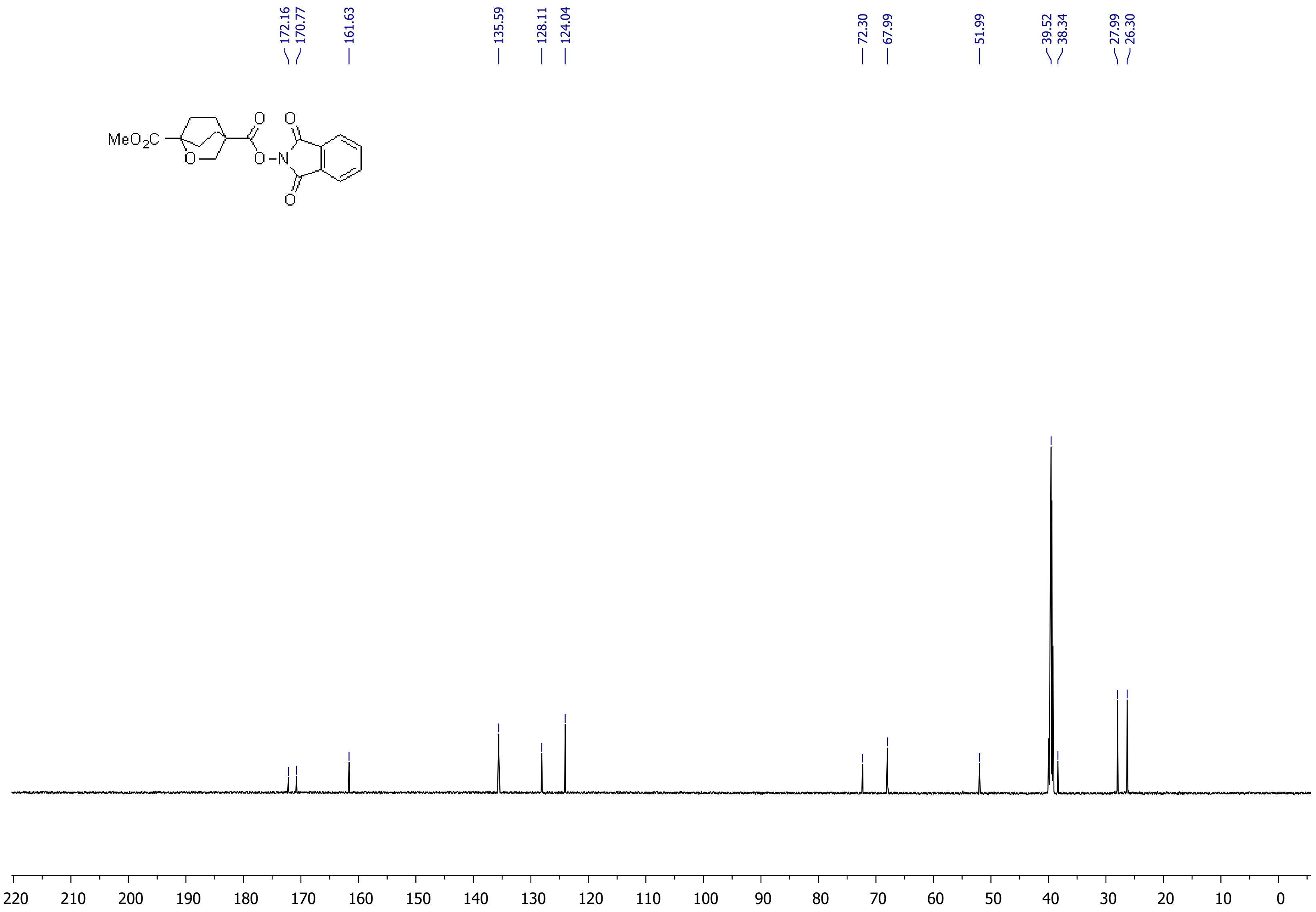

Compound 75

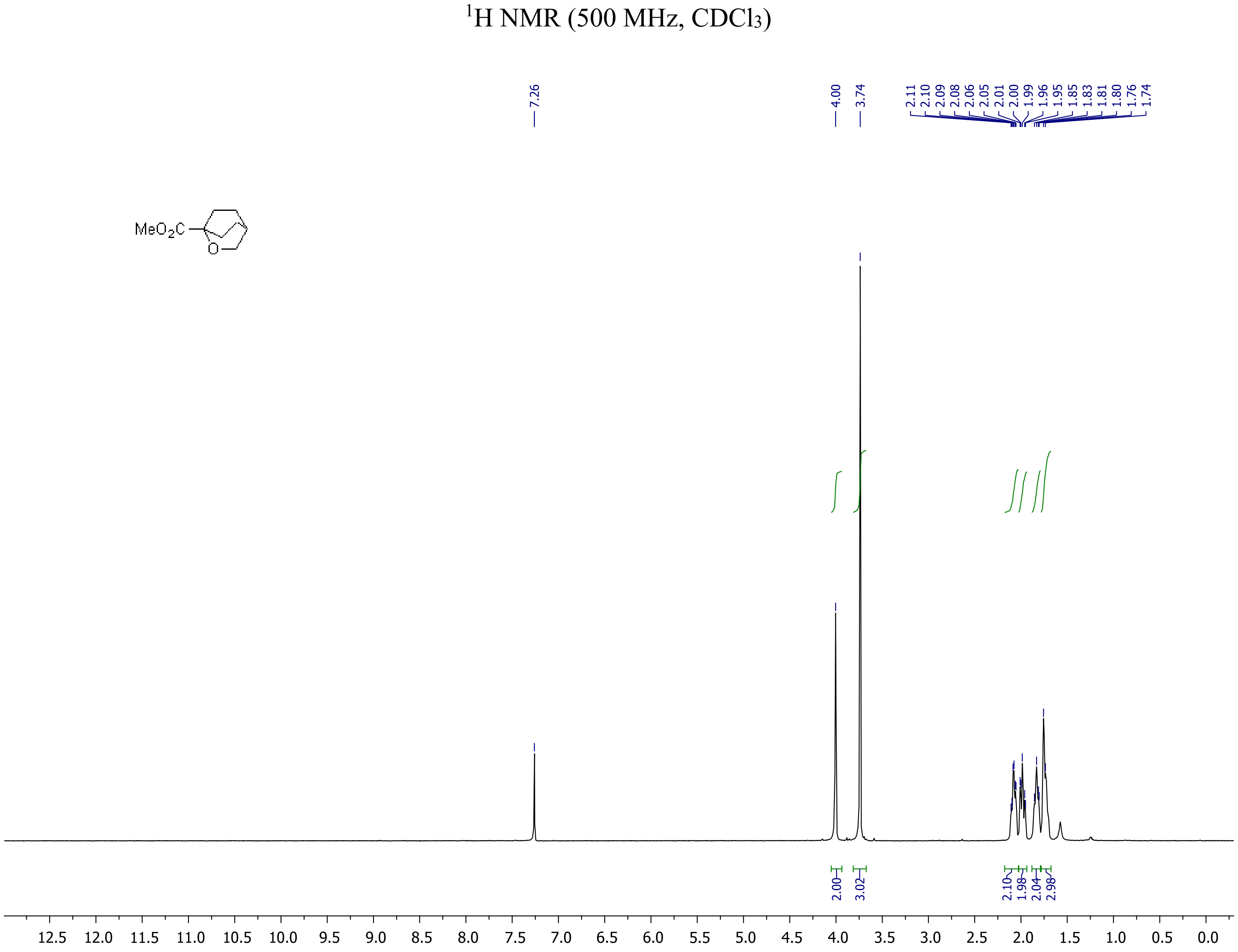

¹H NMR (500 MHz, CDCl3)
MeO2C
O
7.26
4.00
3.74
2.11
2.10
2.09
2.08
2.06
2.05
2.01
2.00
1.99
1.96
1.95
1.85
1.83
1.81
1.80
1.76
1.74
2.00
3.02
2.10
1.98
2.04
2.98
12.5 12.0 11.5 11.0 10.5 10.0 9.5 9.0 8.5 8.0 7.5 7.0 6.5 6.0 5.5 5.0 4.5 4.0 3.5 3.0 2.5 2.0 1.5 1.0 0.5 0.0

$^{13}C\{^{1}H\}$ NMR (151 MHz, $CDCl_3$)

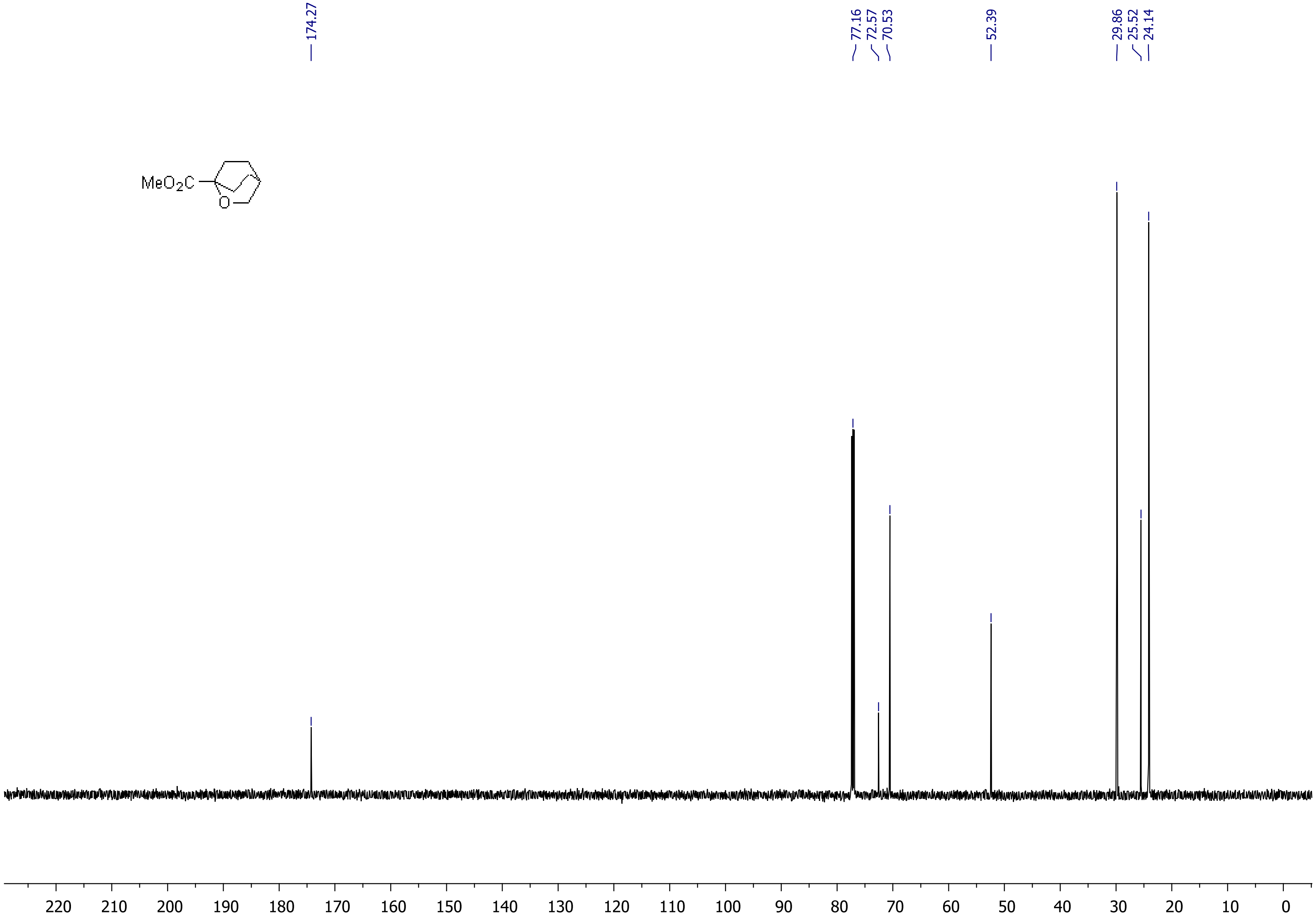

Compound 76

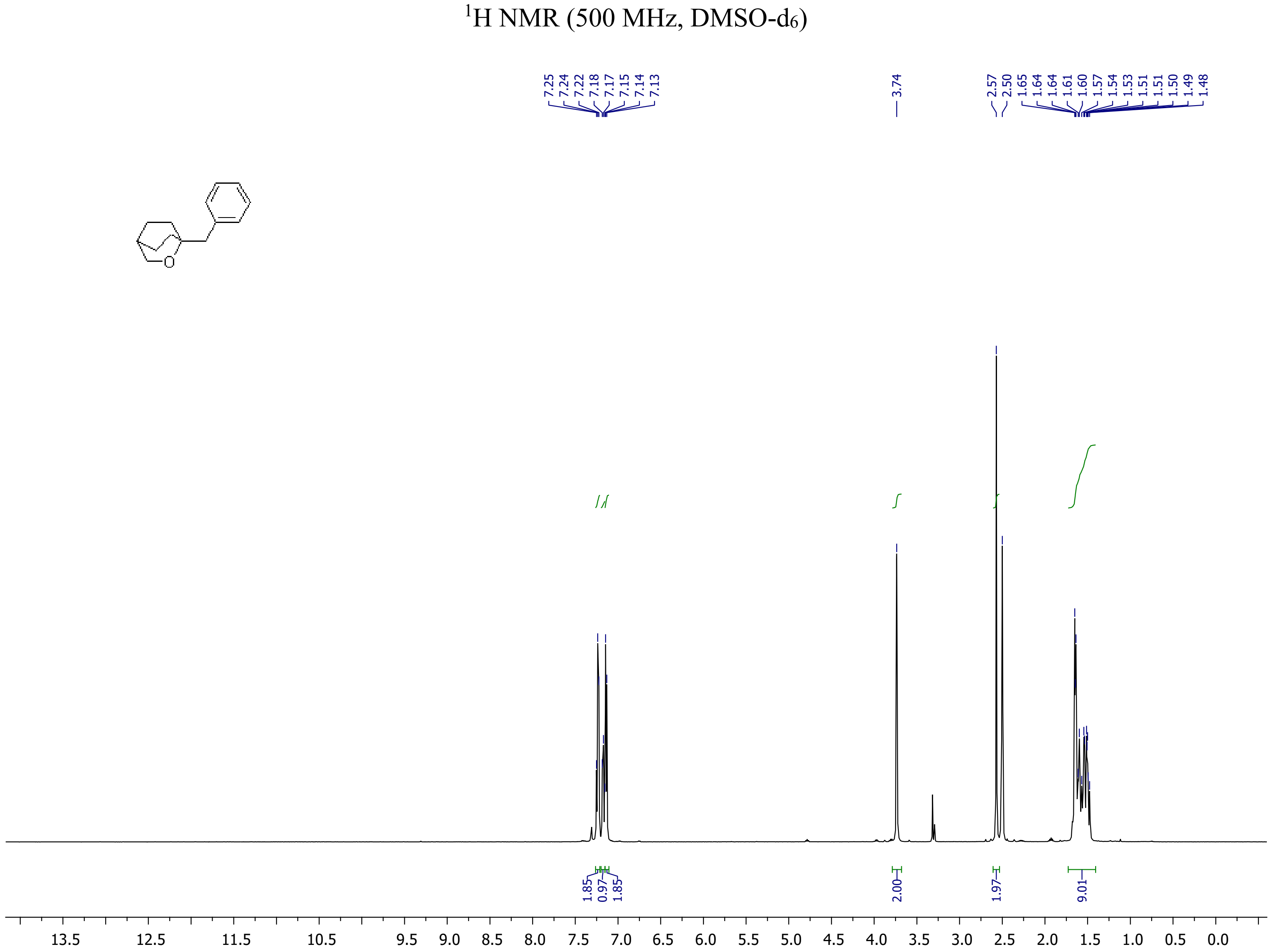

^{1}H NMR (500 MHz, DMSO-d6)
7.25
7.24
7.22
7.18
7.17
7.15
7.14
7.13
3.74
2.57
2.50
1.65
1.64
1.64
1.61
1.60
1.57
1.54
1.53
1.51
1.51
1.50
1.49
1.48
1.85
0.97
1.85
2.00
1.97
9.01
13.5
12.5
11.5
10.5
9.5
9.0
8.5
8.0
7.5
7.0
6.5
6.0
5.5
5.0
4.5
4.0
3.5
3.0
2.5
2.0
1.5
1.0
0.5
0.0

$^{13}C\{^{1}H\}$ NMR (126 MHz, DMSO-d$_6$)

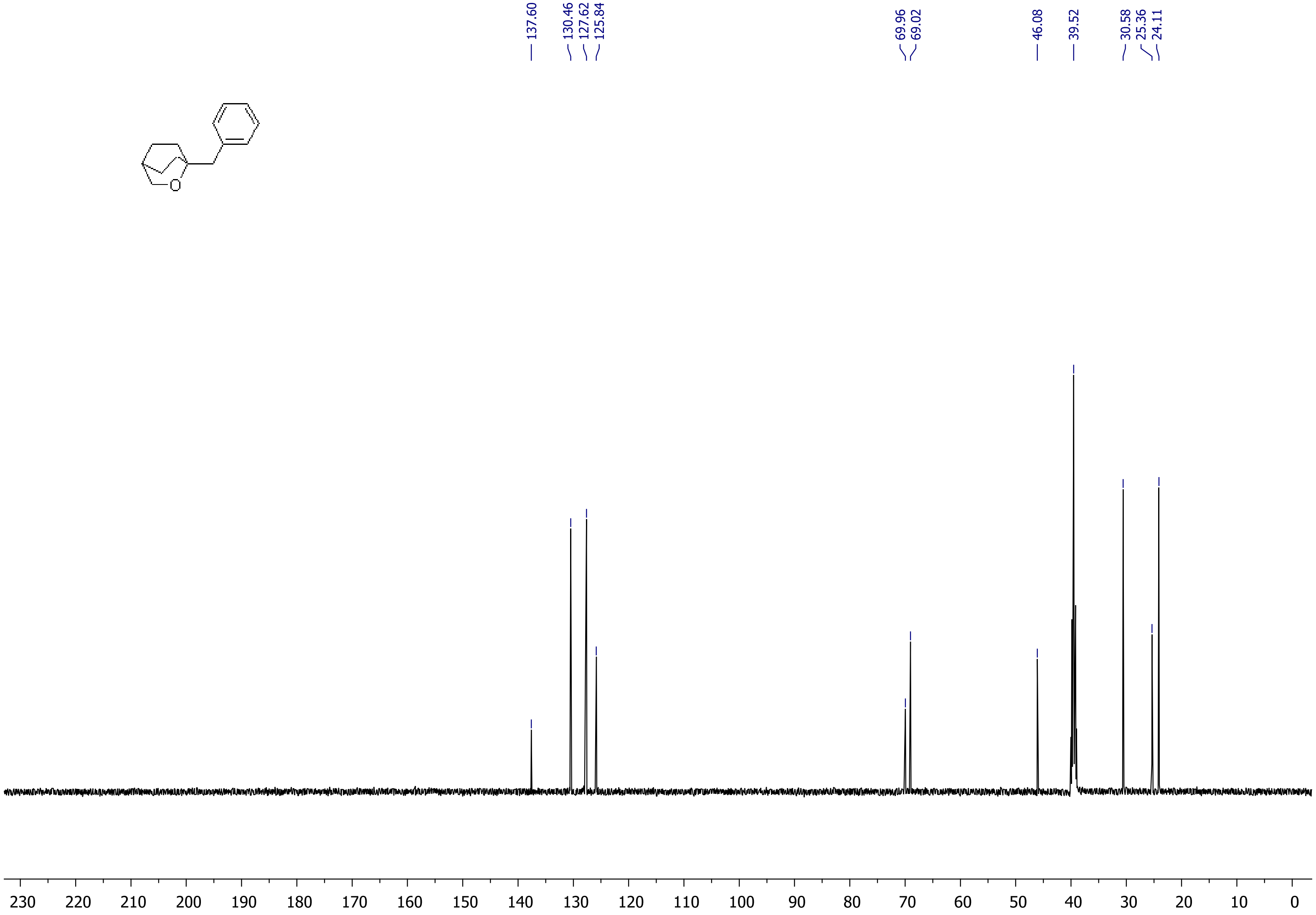

Compound 77

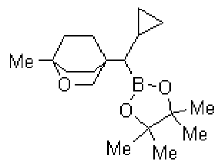
Me
O
B
O
O
Me
Me
Me
Me

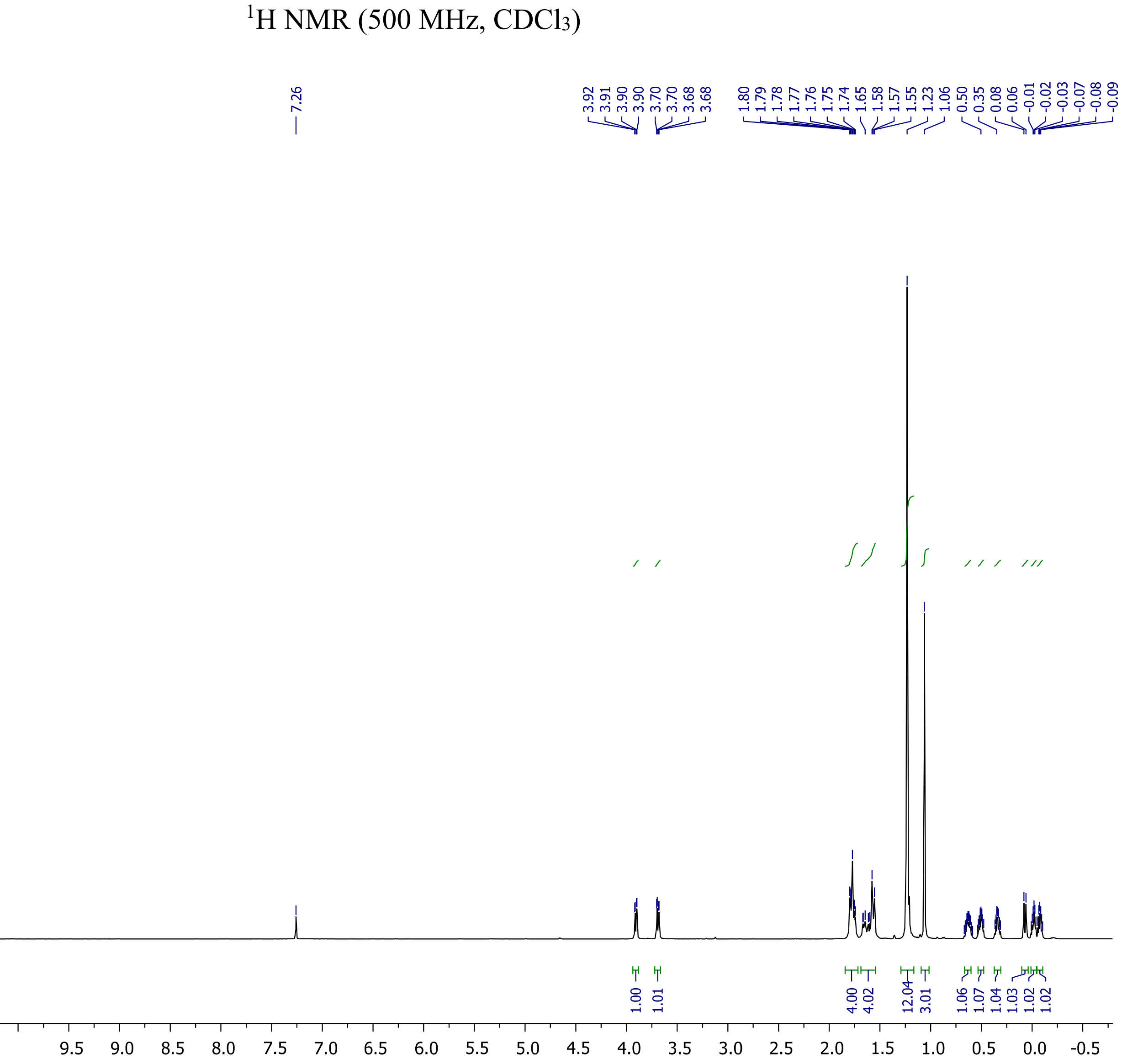
¹H NMR (500 MHz, CDCl3)
7.26
3.92
3.91
3.90
3.90
3.70
3.70
3.68
3.68
1.80
1.79
1.78
1.77
1.76
1.75
1.74
1.65
1.58
1.57
1.55
1.23
1.06
0.50
0.35
0.08
0.06
-0.01
-0.02
-0.03
-0.07
-0.08
-0.09
1.00
1.01
4.00
4.02
12.04
3.01
1.06
1.07
1.04
1.03
1.02
1.02
12.5
11.5
10.5
9.5
9.0
8.5
8.0
7.5
7.0
6.5
6.0
5.5
5.0
4.5
4.0
3.5
3.0
2.5
2.0
1.5
1.0
0.5
0.0
-0.5

$^{13}C\{^1H\}$ NMR (151 MHz, DMSO-d$_6$)

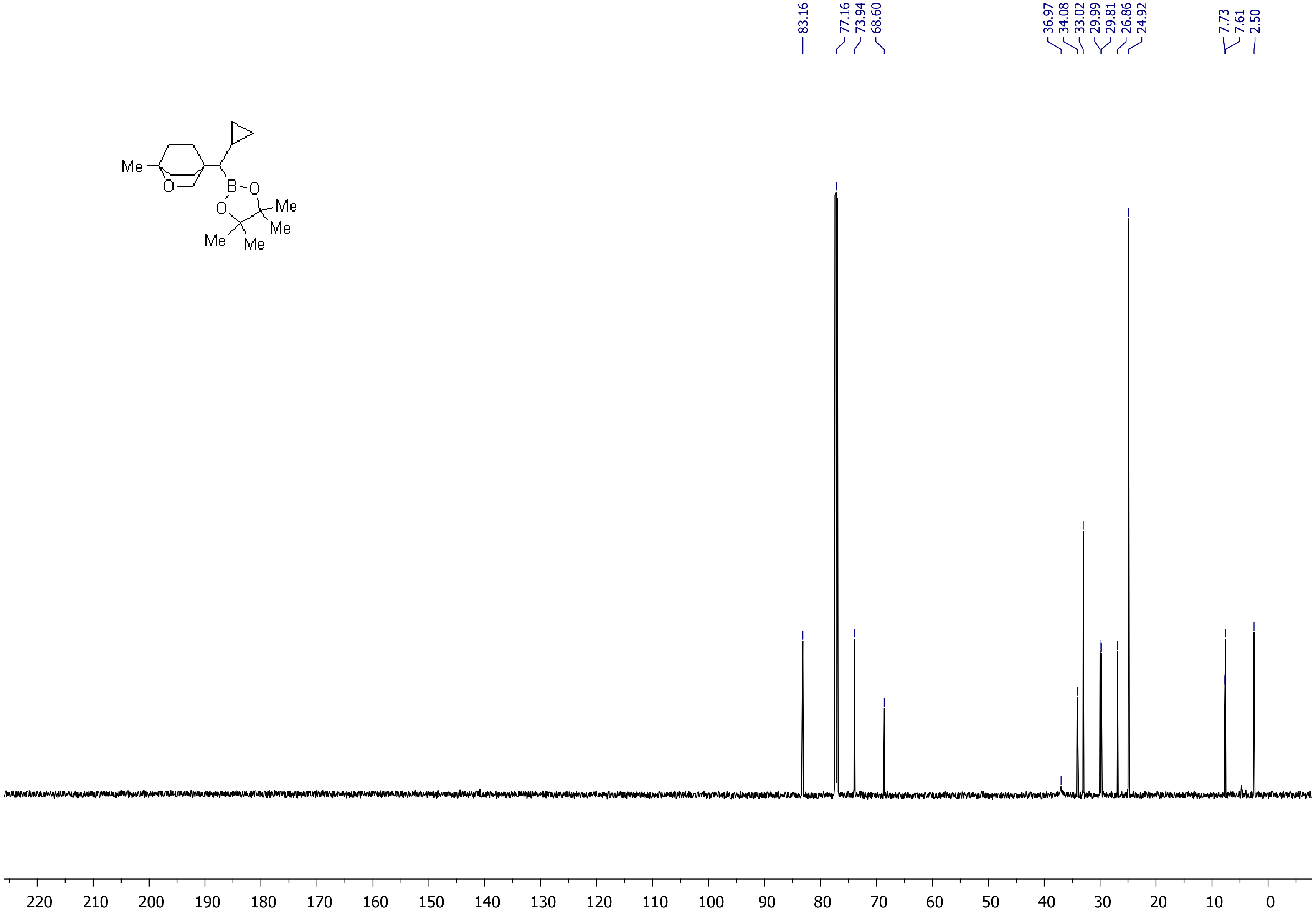

Compound 78

^{1}H NMR (500 MHz, CDCl$_3$)

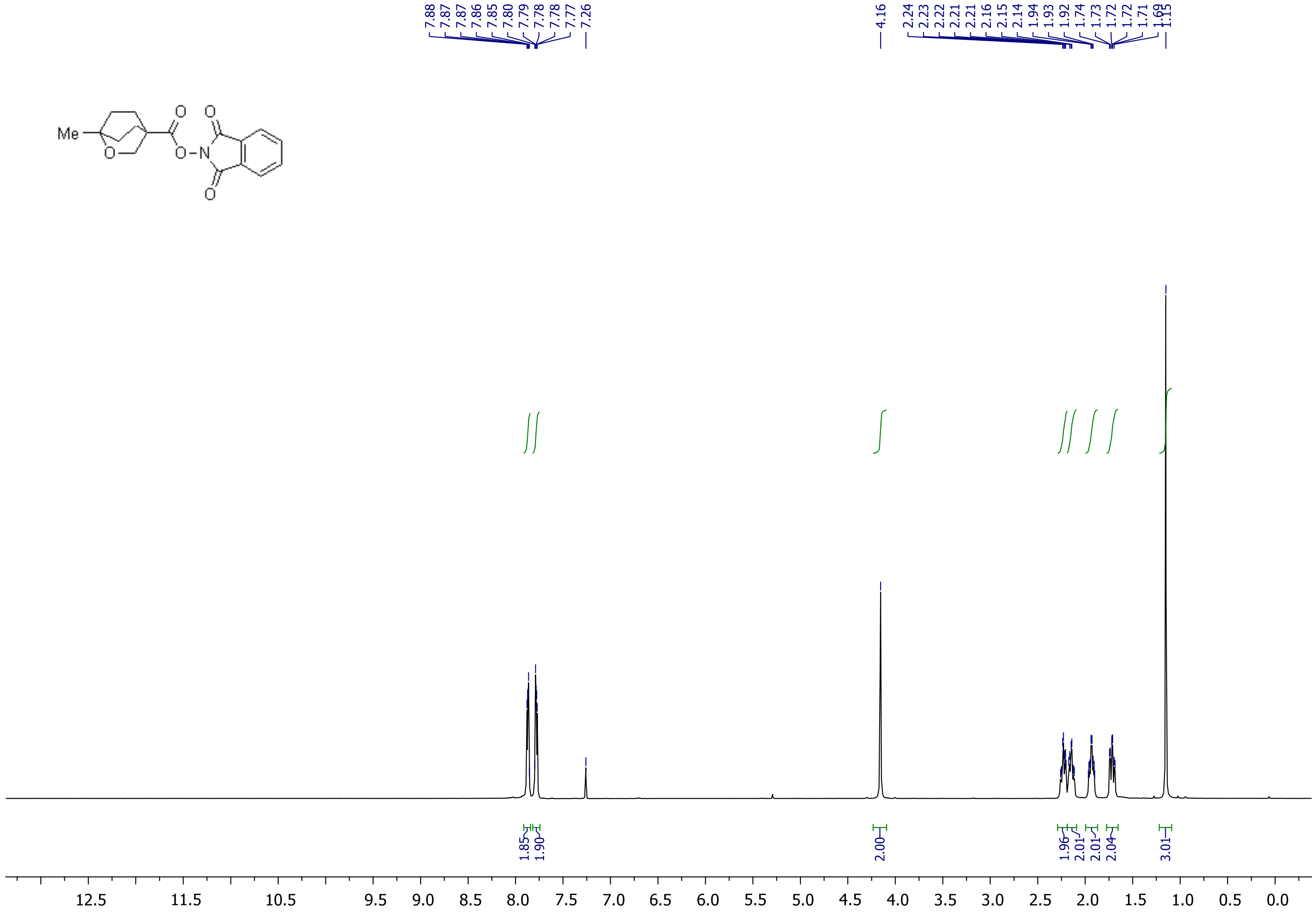

$^{13}C\{^1H\}$ NMR (151 MHz, $CDCl_3$)

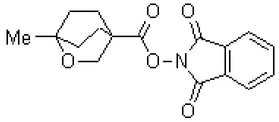

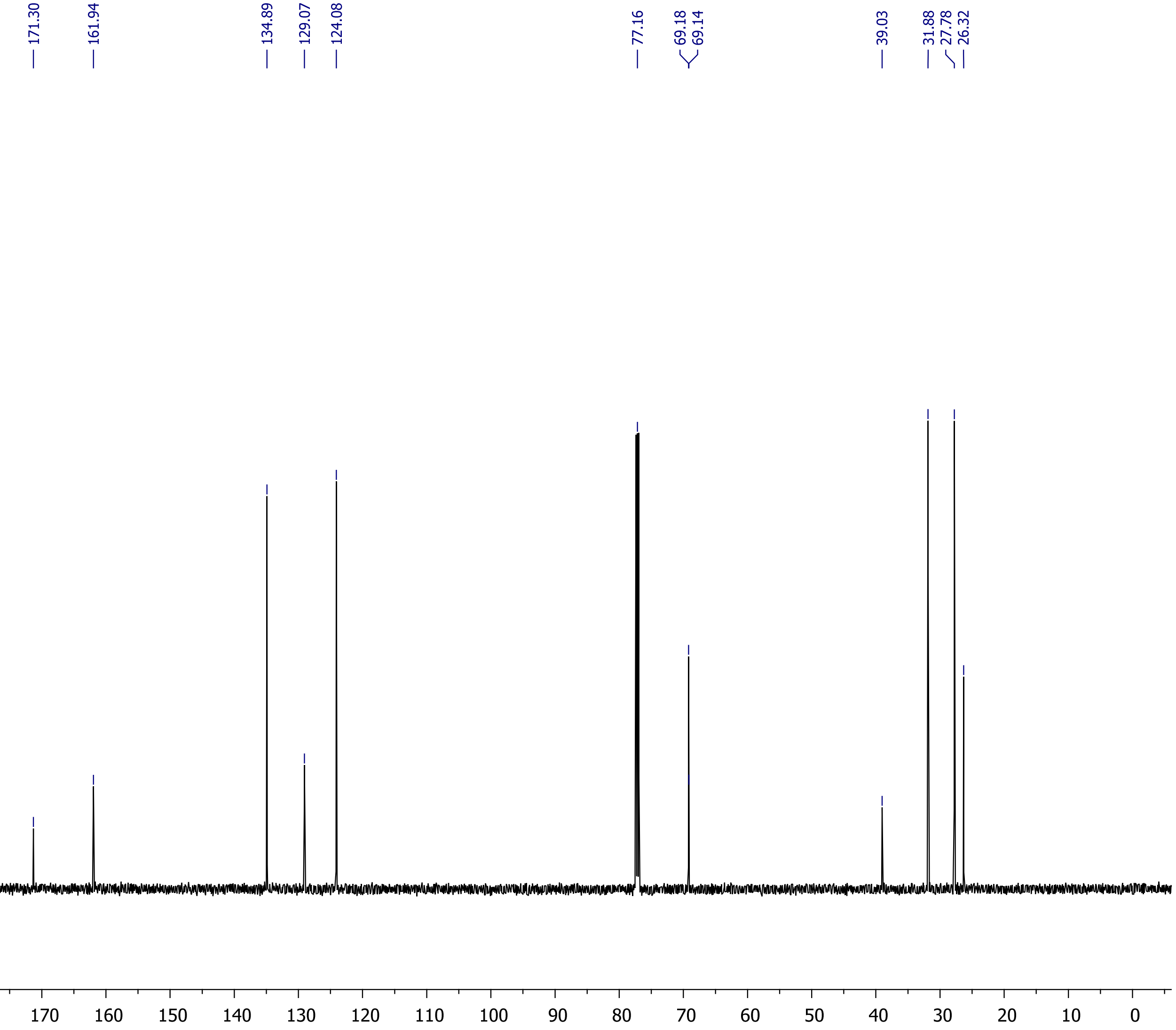

Compound 79

^{1}H NMR (500 MHz, $CDCl_3$)

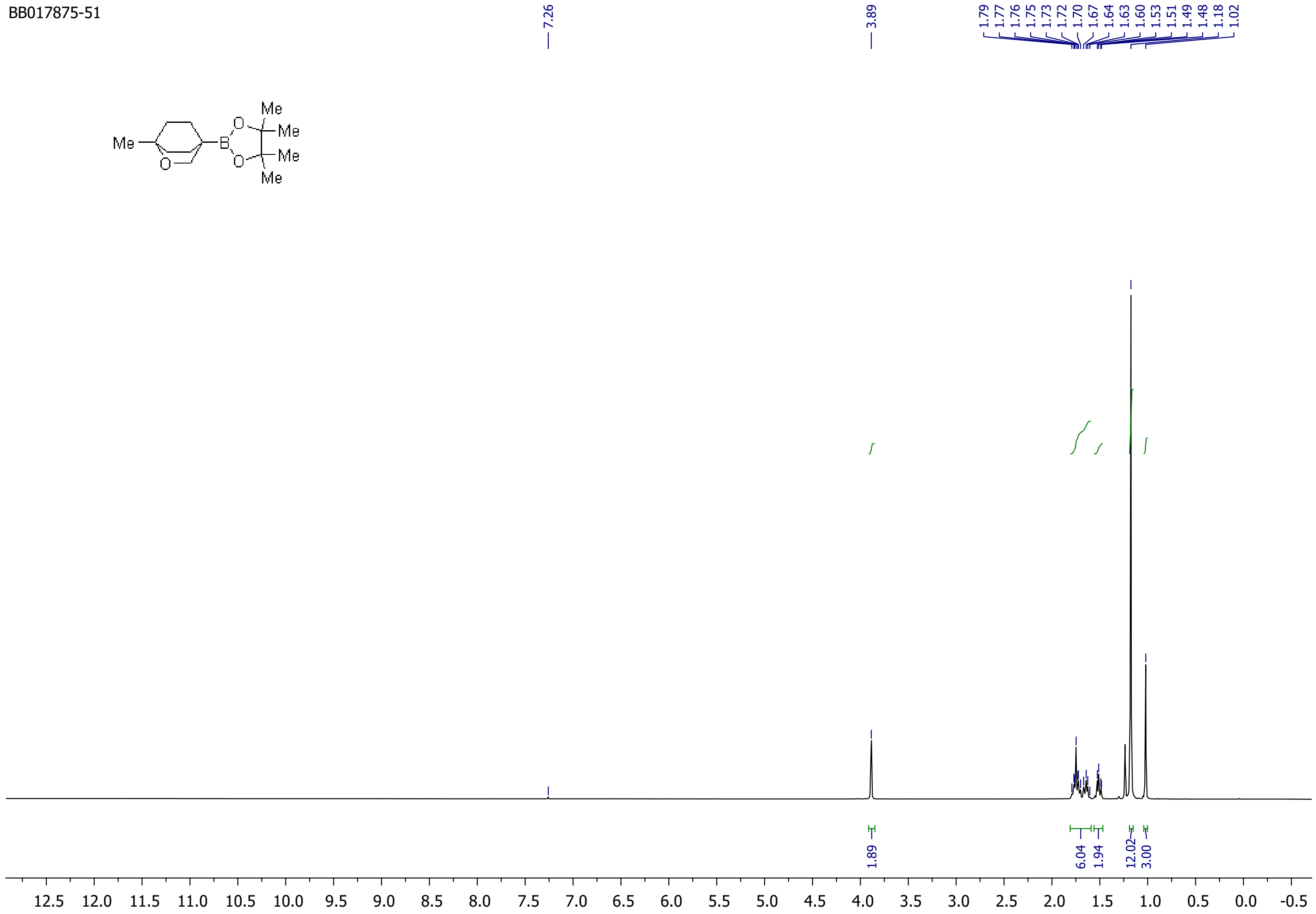

$^{13}C\{^1H\}$ NMR (126 MHz, $CDCl_3$)

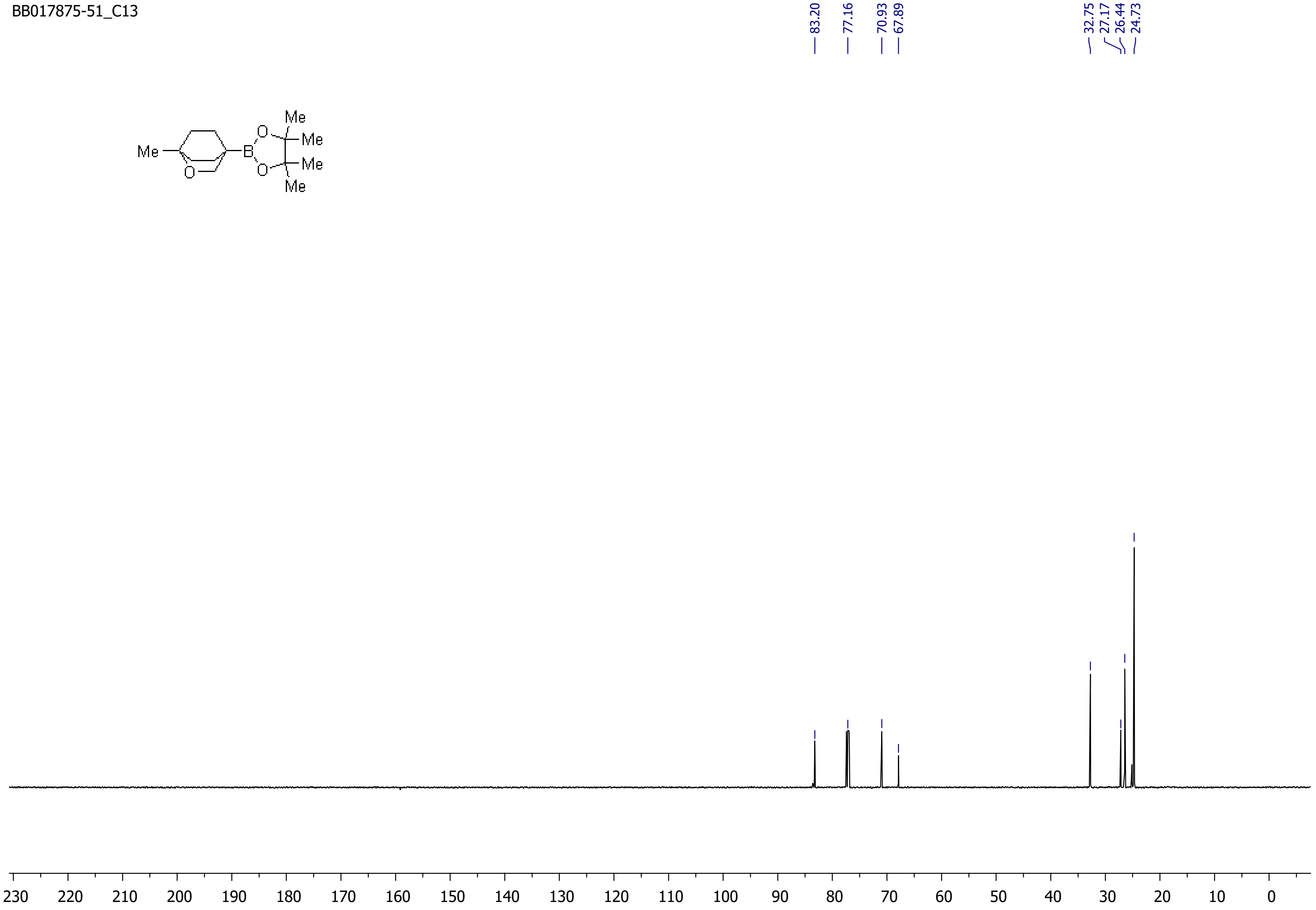

Compound 85

^{1}H NMR (500 MHz, $CDCl_3$)

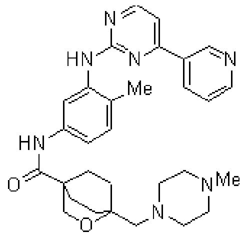

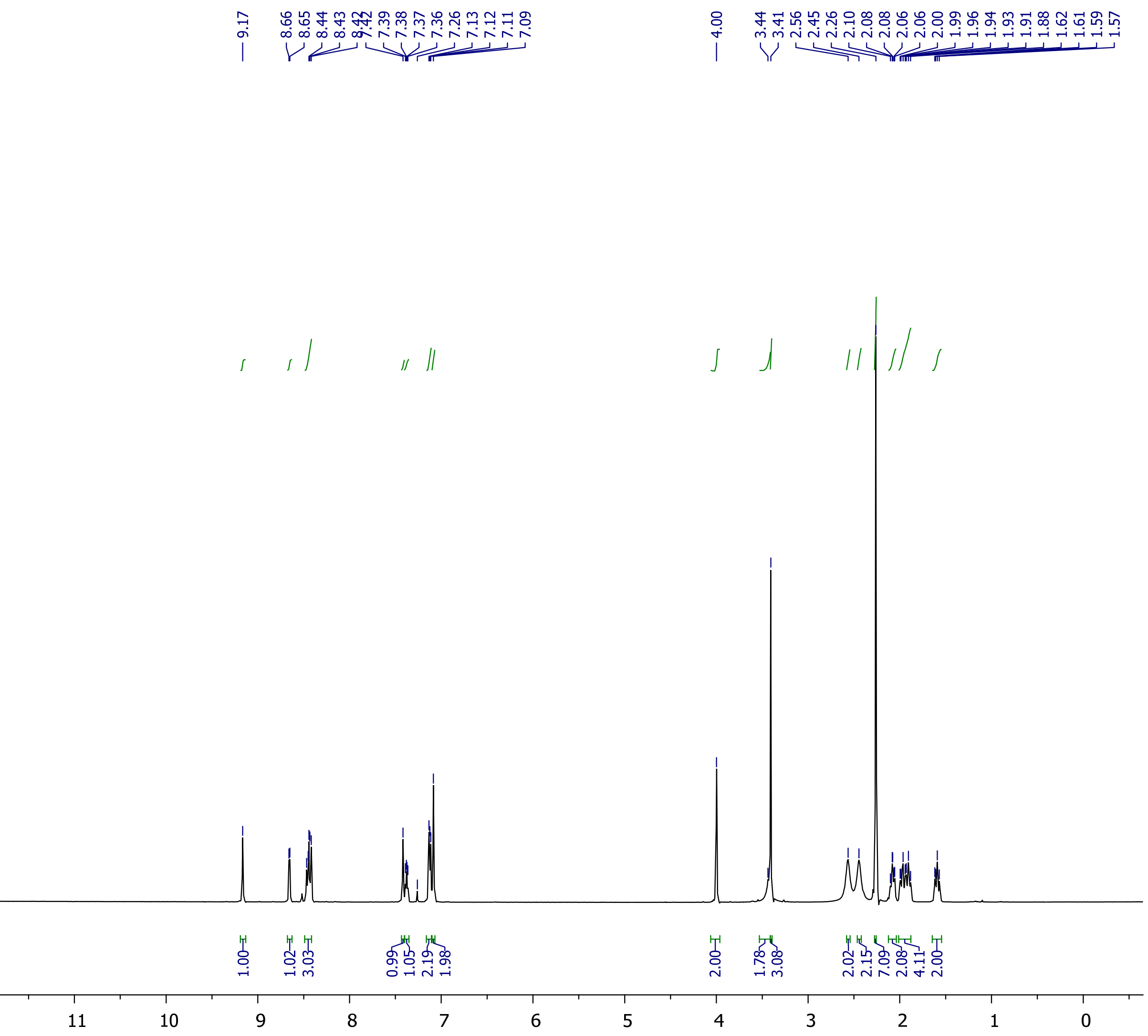

$^{13}C\{^{1}H\}$ NMR (126 MHz, $CDCl_3$)

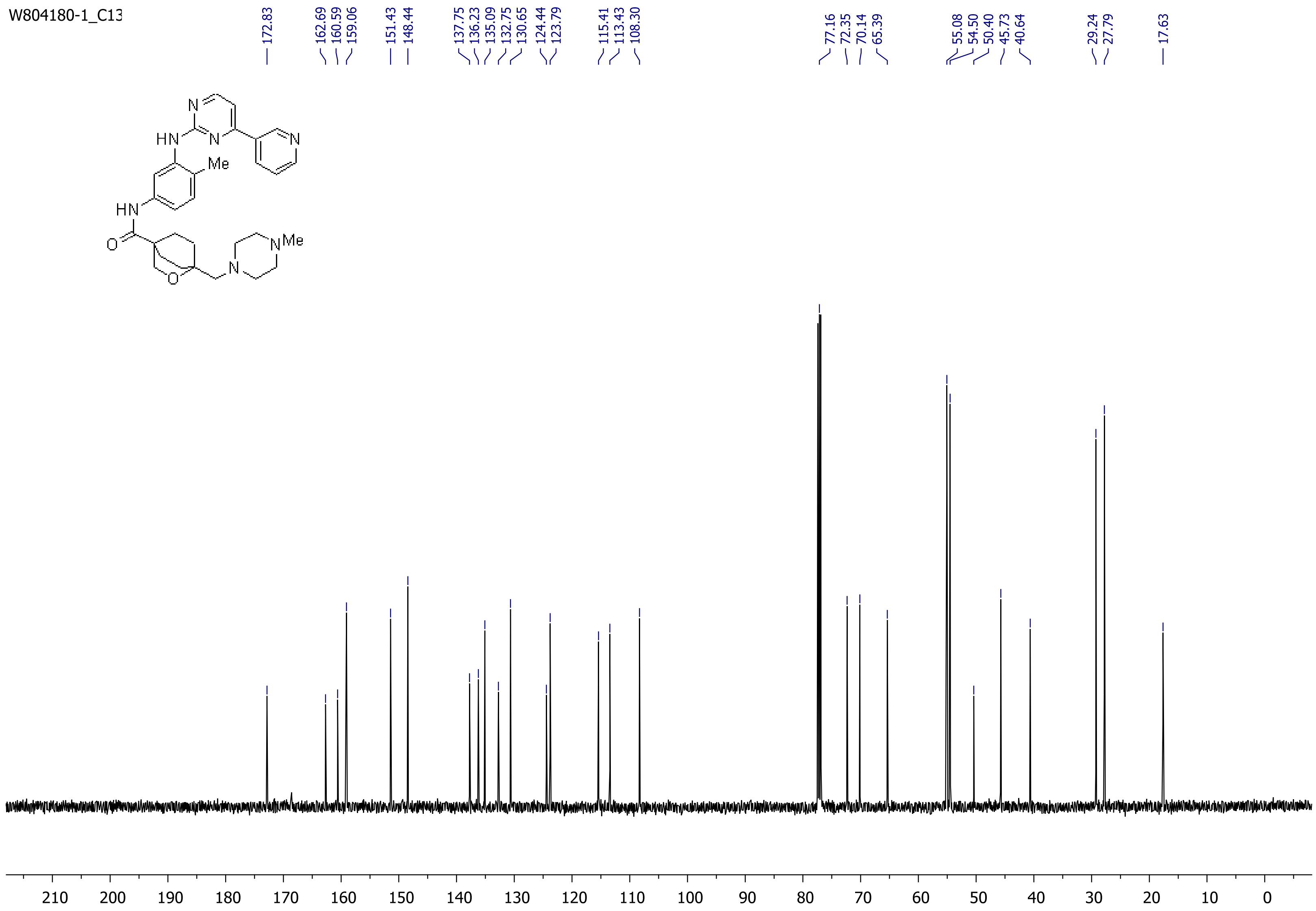

Compound SI-24

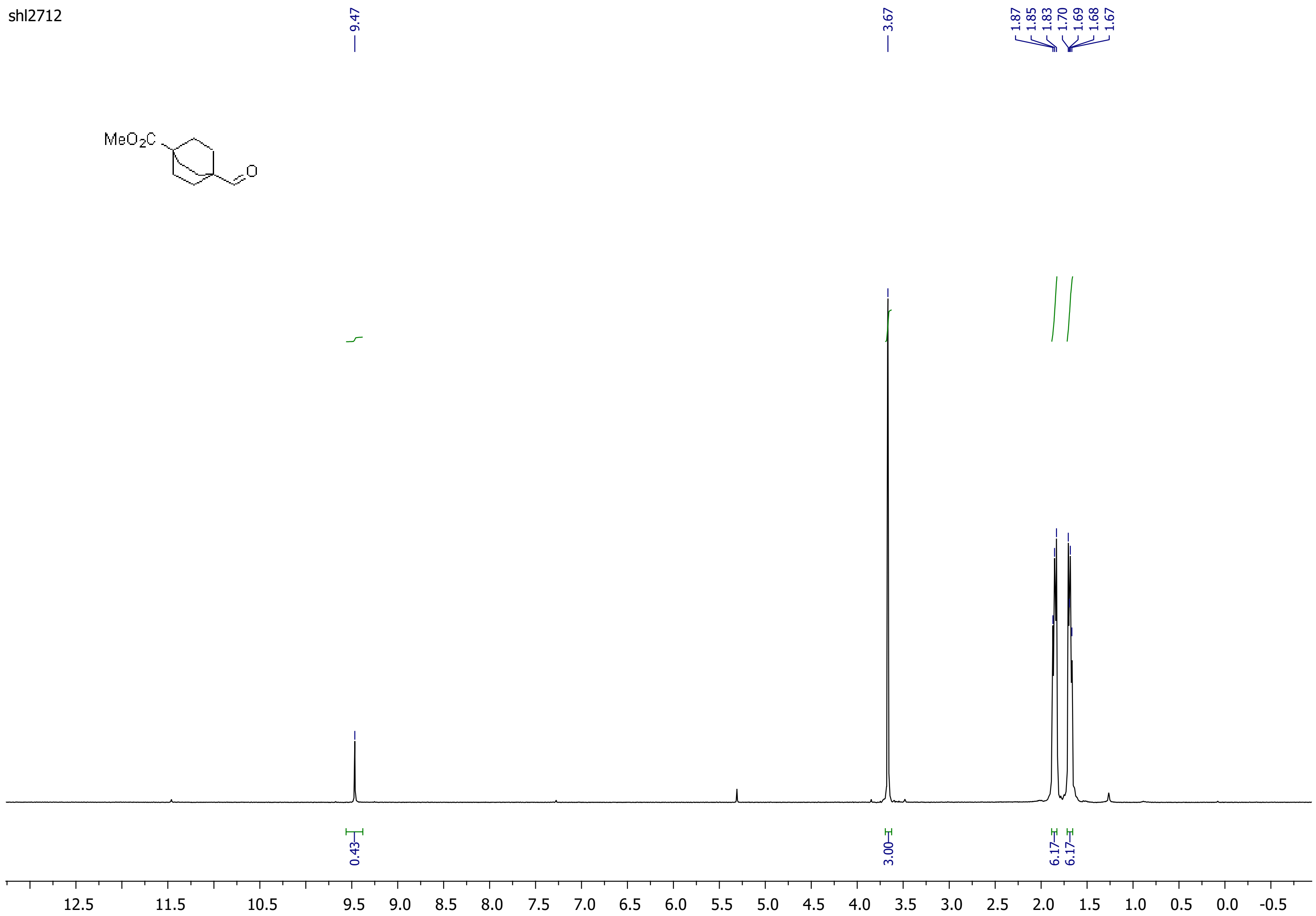

Compound SI-25

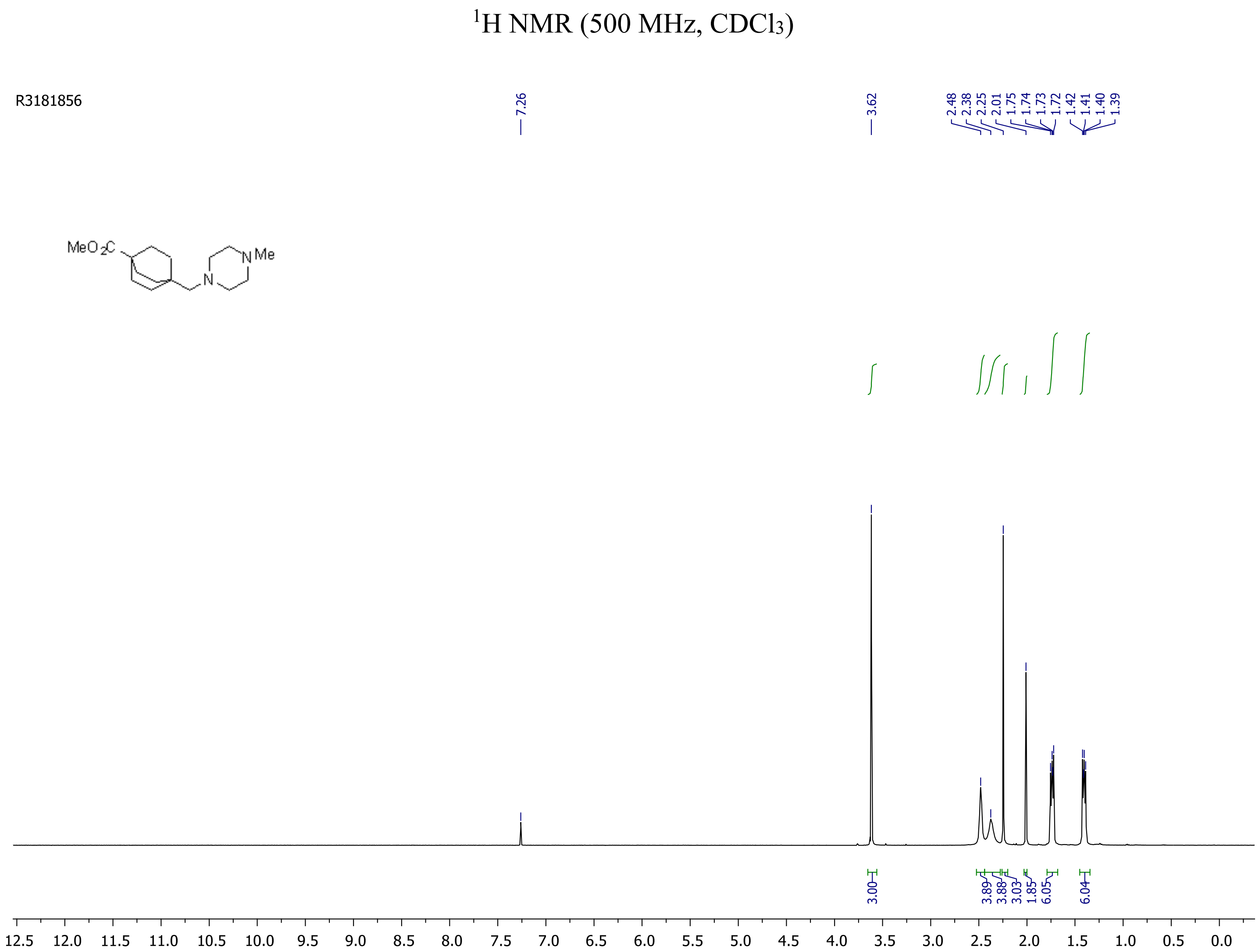

$^{13}C\{^1H\}$ NMR (126 MHz, $CDCl_3$)

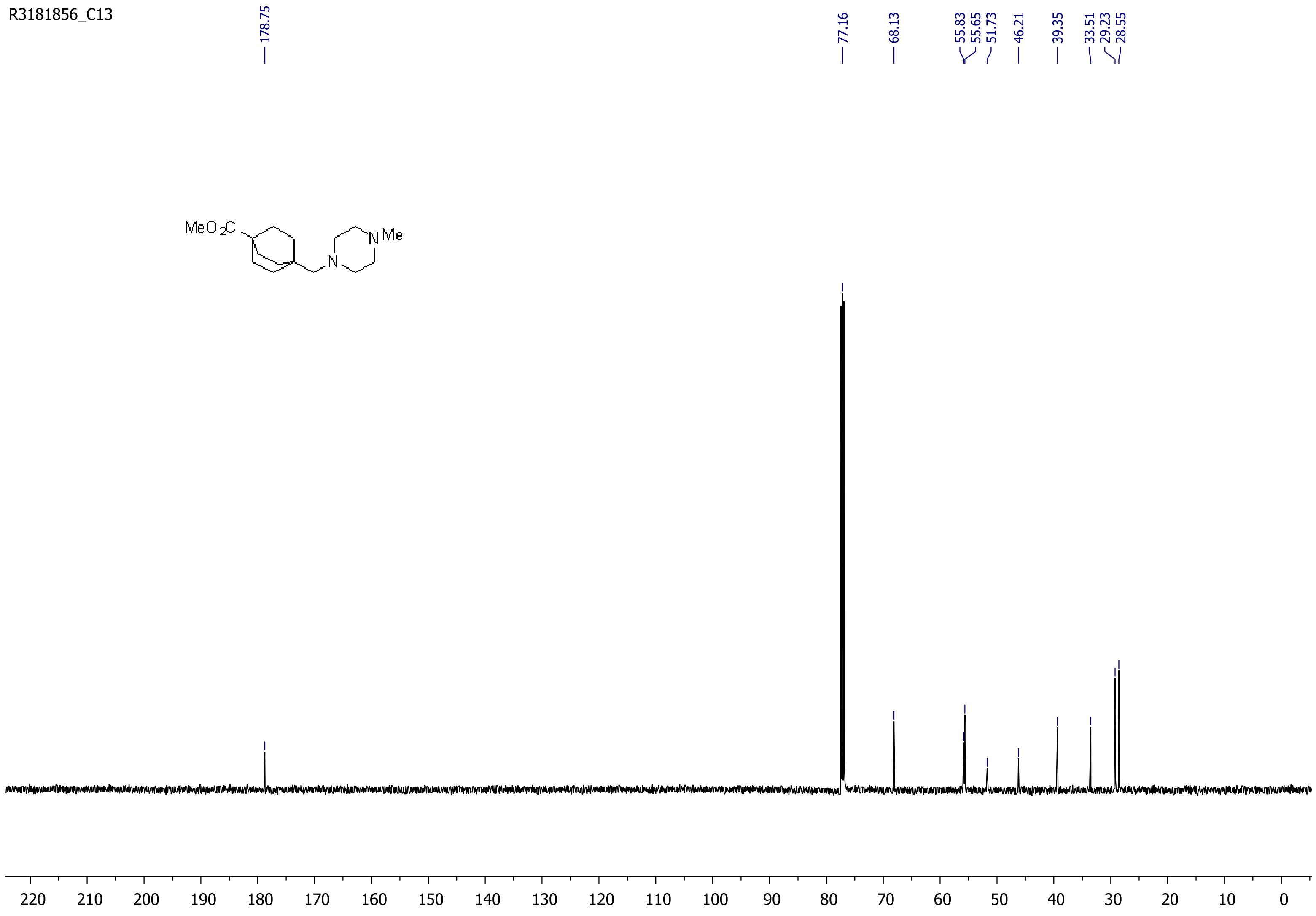

Compound SI-26

^1H NMR (500 MHz, D_2O)

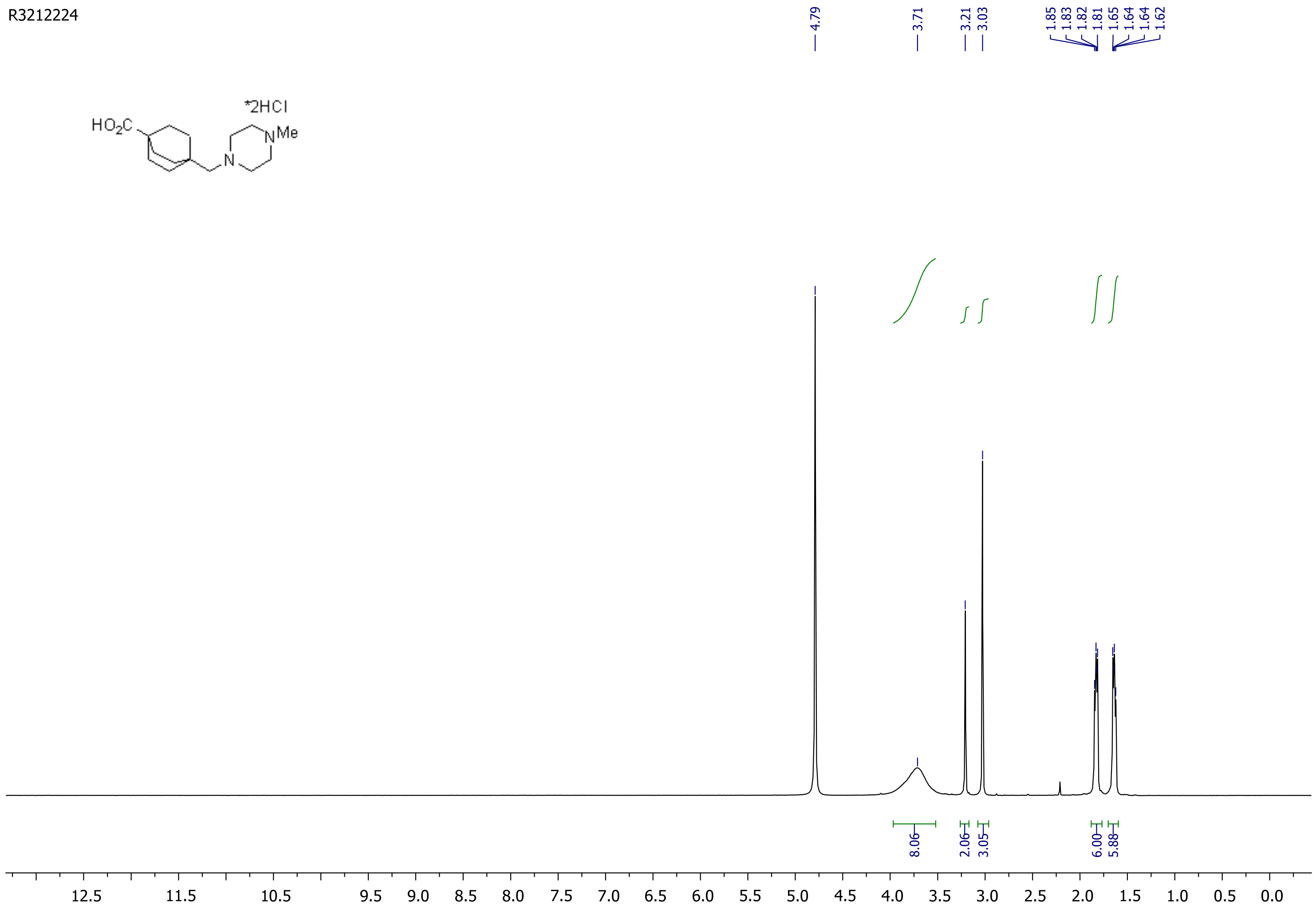

$^{13}C\{^{1}H\}$ NMR (151 MHz, D_2O)

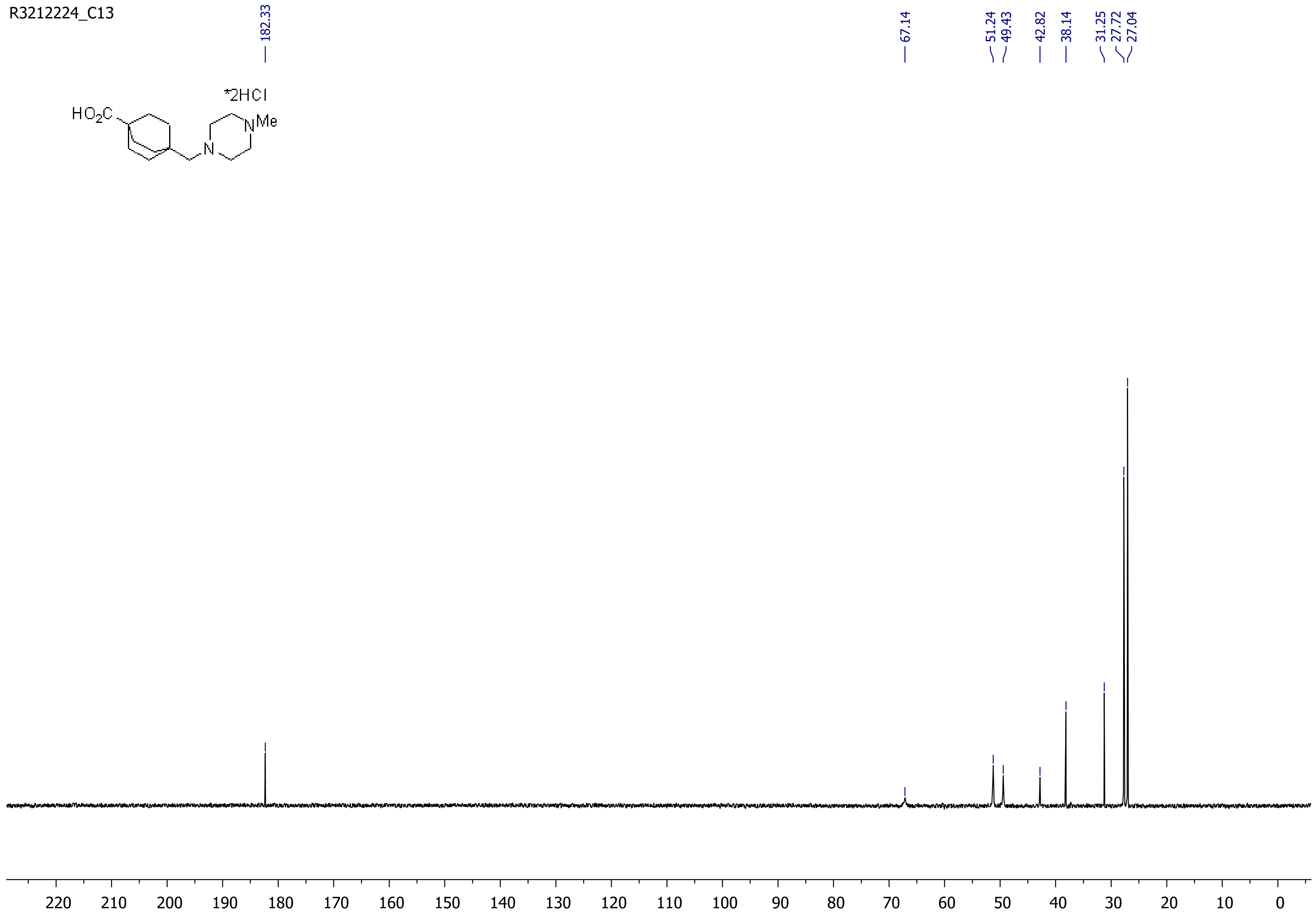

Compound 86

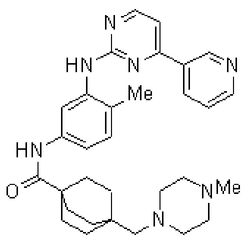

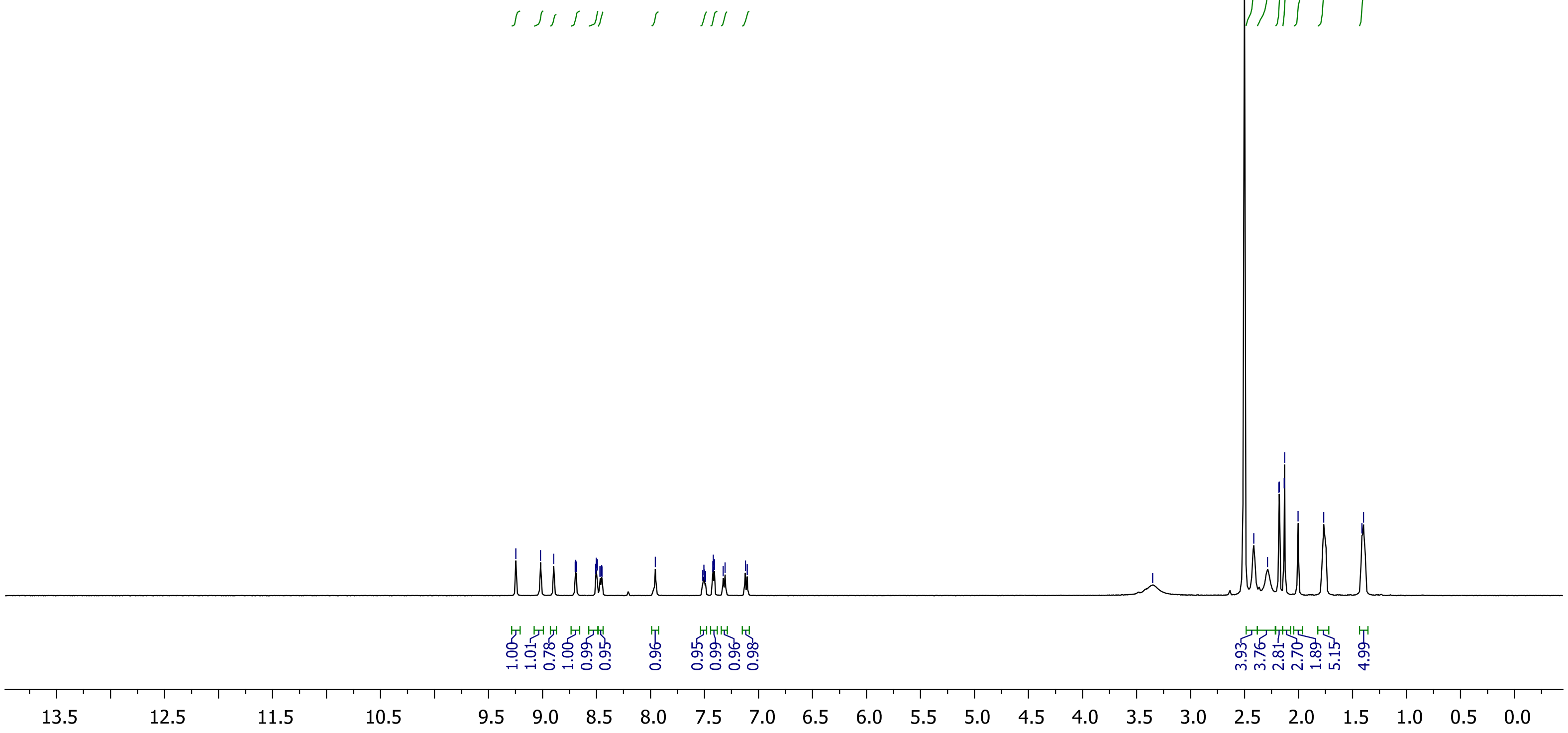

¹H NMR (500 MHz, DMSO-d6)
R2883227
9.25 9.02 8.90 8.70 8.69 8.69 8.51 8.50 8.50 8.49 8.47 8.47 8.45 8.45 7.96 7.52 7.51 7.50 7.49 7.42 7.42 7.41 7.41 7.33 7.31 7.12 7.10
3.35 2.50 2.41 2.29 2.18 2.18 2.13 2.13 2.00 1.77 1.41 1.40
1.00 1.01 0.78 1.00 0.99 0.95 0.96 0.95 0.99 0.96 0.98
3.93 3.76 2.81 2.70 1.89 5.15 4.99
13.5 12.5 11.5 10.5 9.5 9.0 8.5 8.0 7.5 7.0 6.5 6.0 5.5 5.0 4.5 4.0 3.5 3.0 2.5 2.0 1.5 1.0 0.5 0.0

$^{13}C\{^1H\}$ NMR (101 MHz, DMSO-d6)

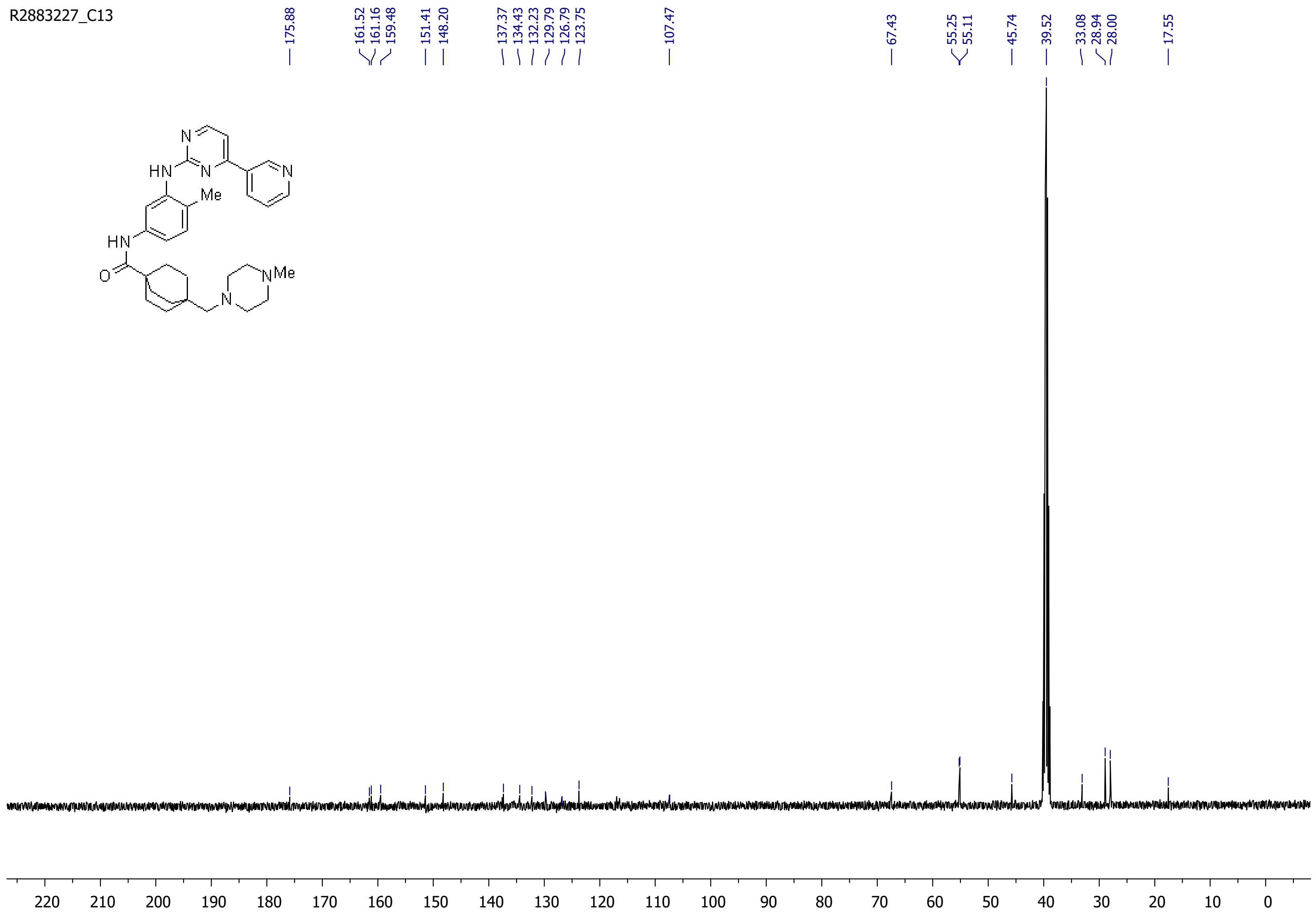

Compound SI-27

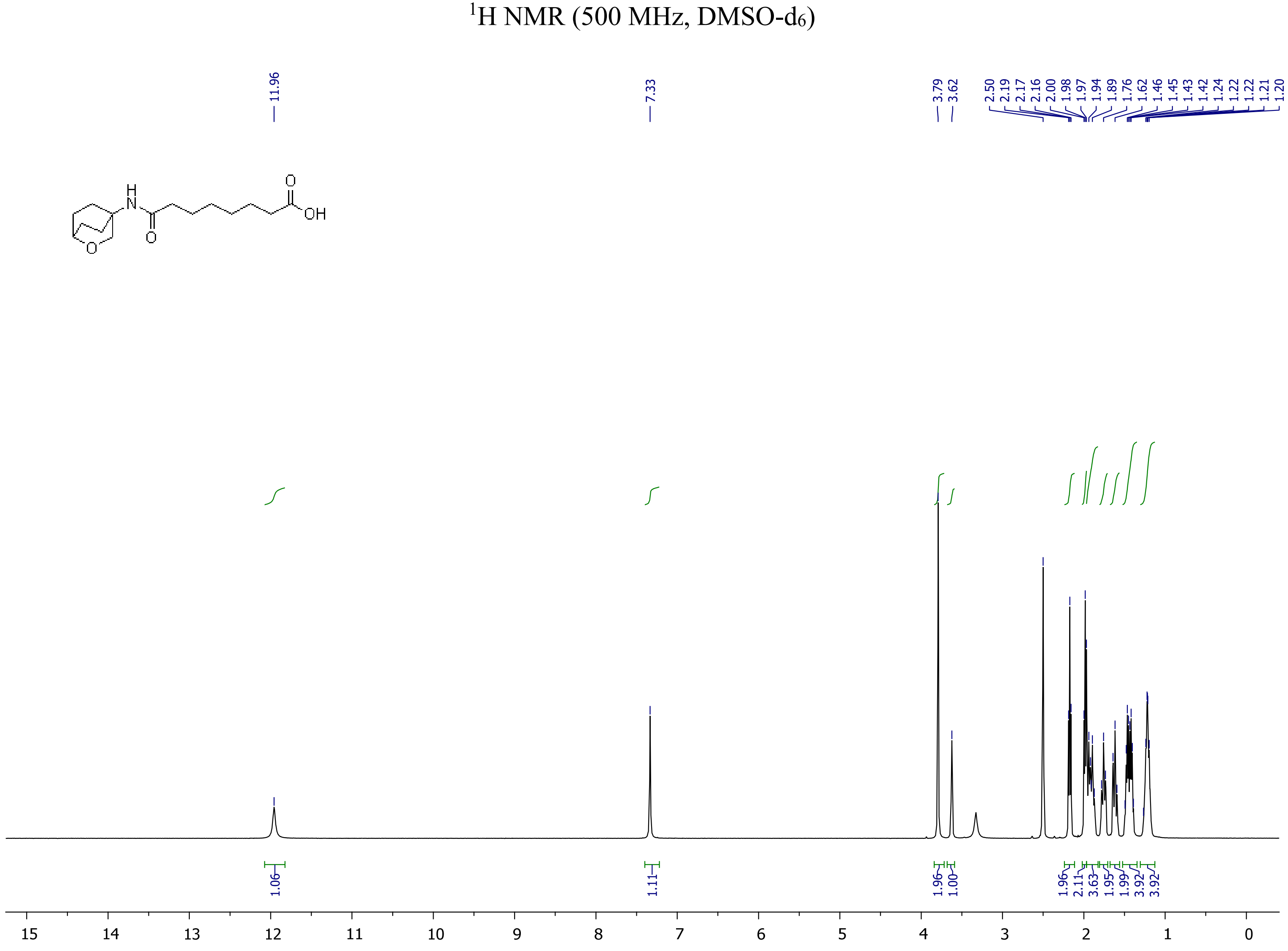

$^{13}C\{^1H\}$ NMR (126 MHz, DMSO-d6)

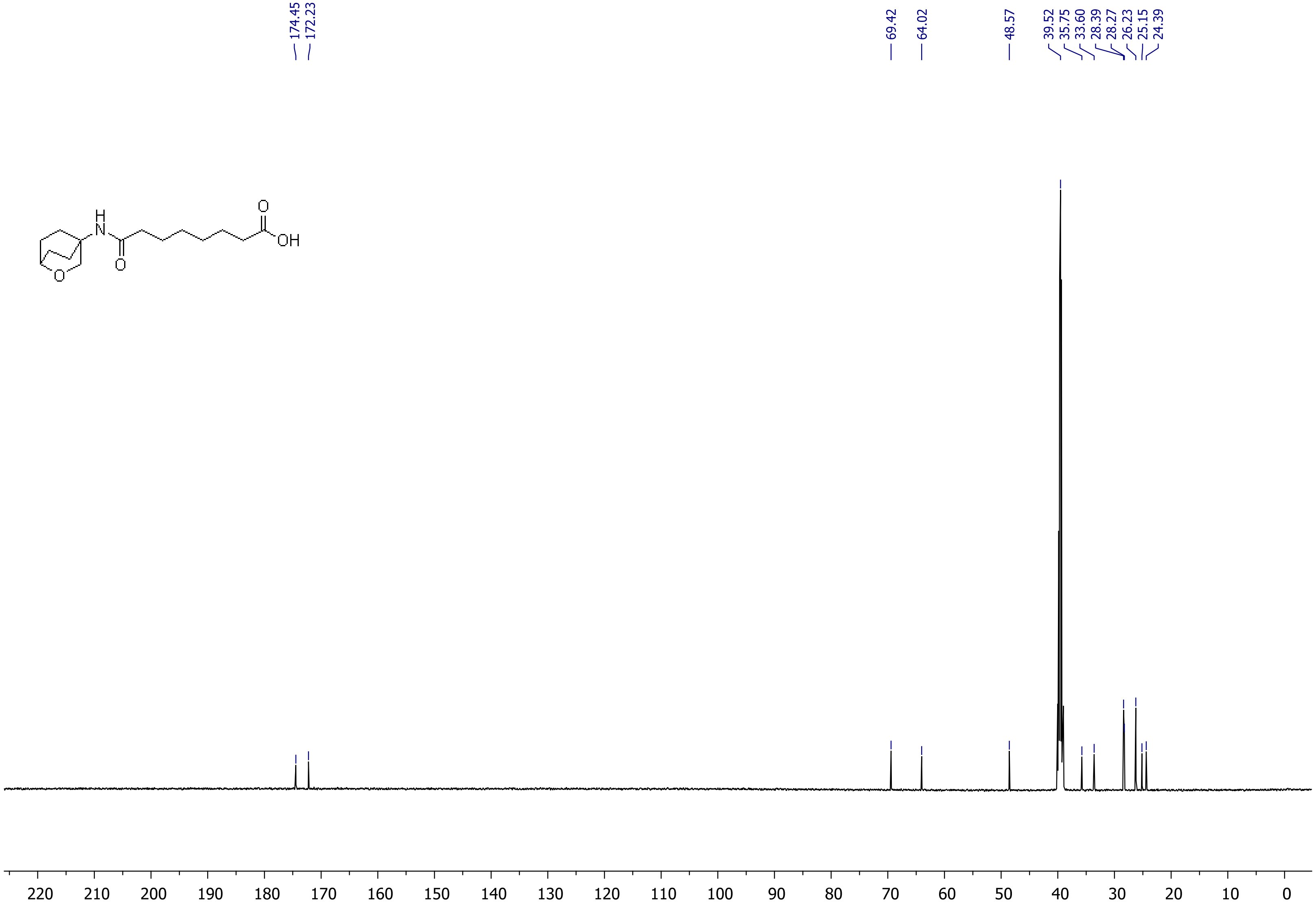

Compound 88

^{1}H NMR (500 MHz, DMSO-d_6)

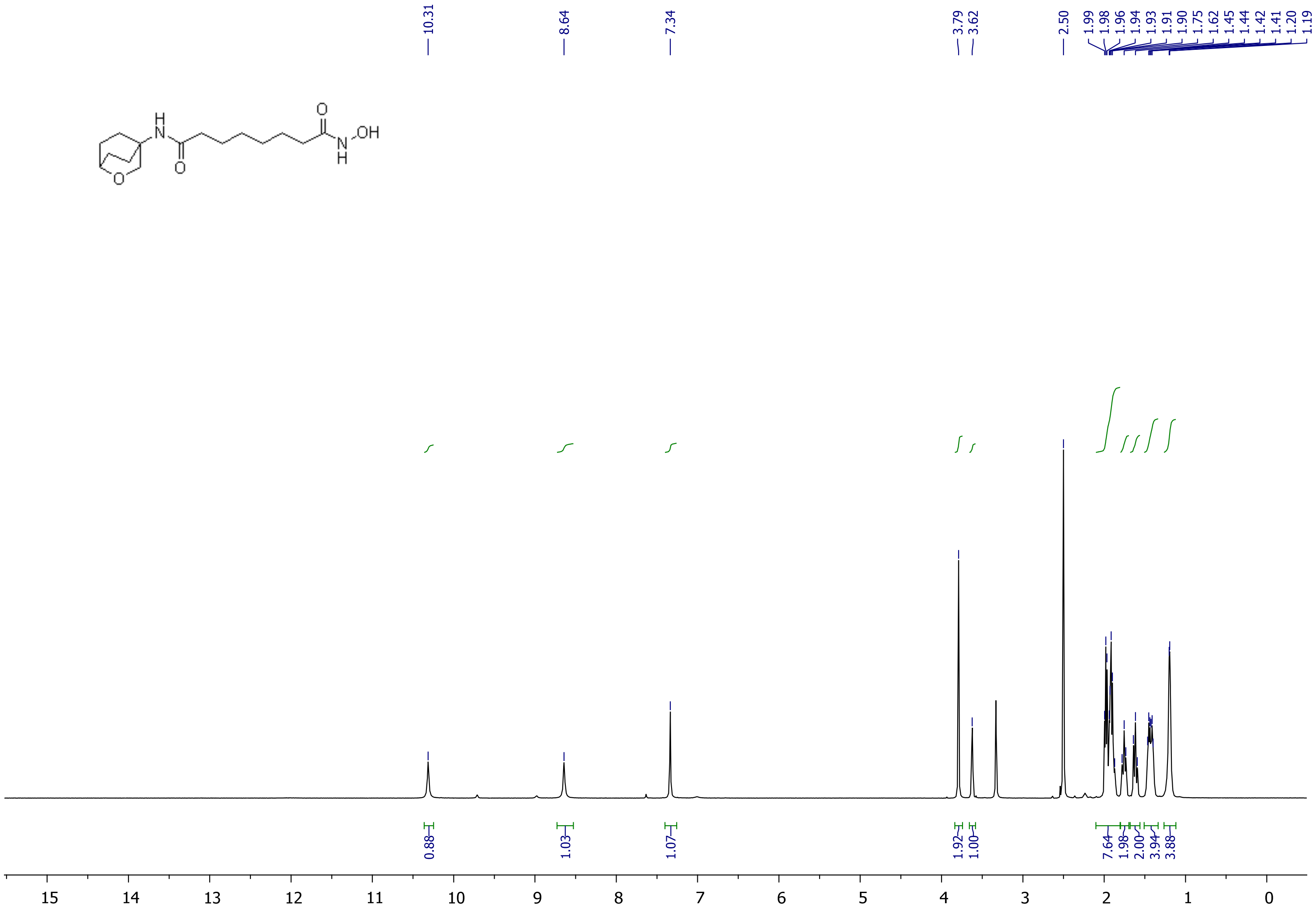

$^{13}C\{^{1}H\}$ NMR (151 MHz, DMSO-d6)

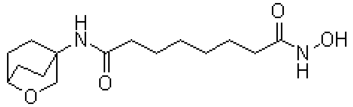

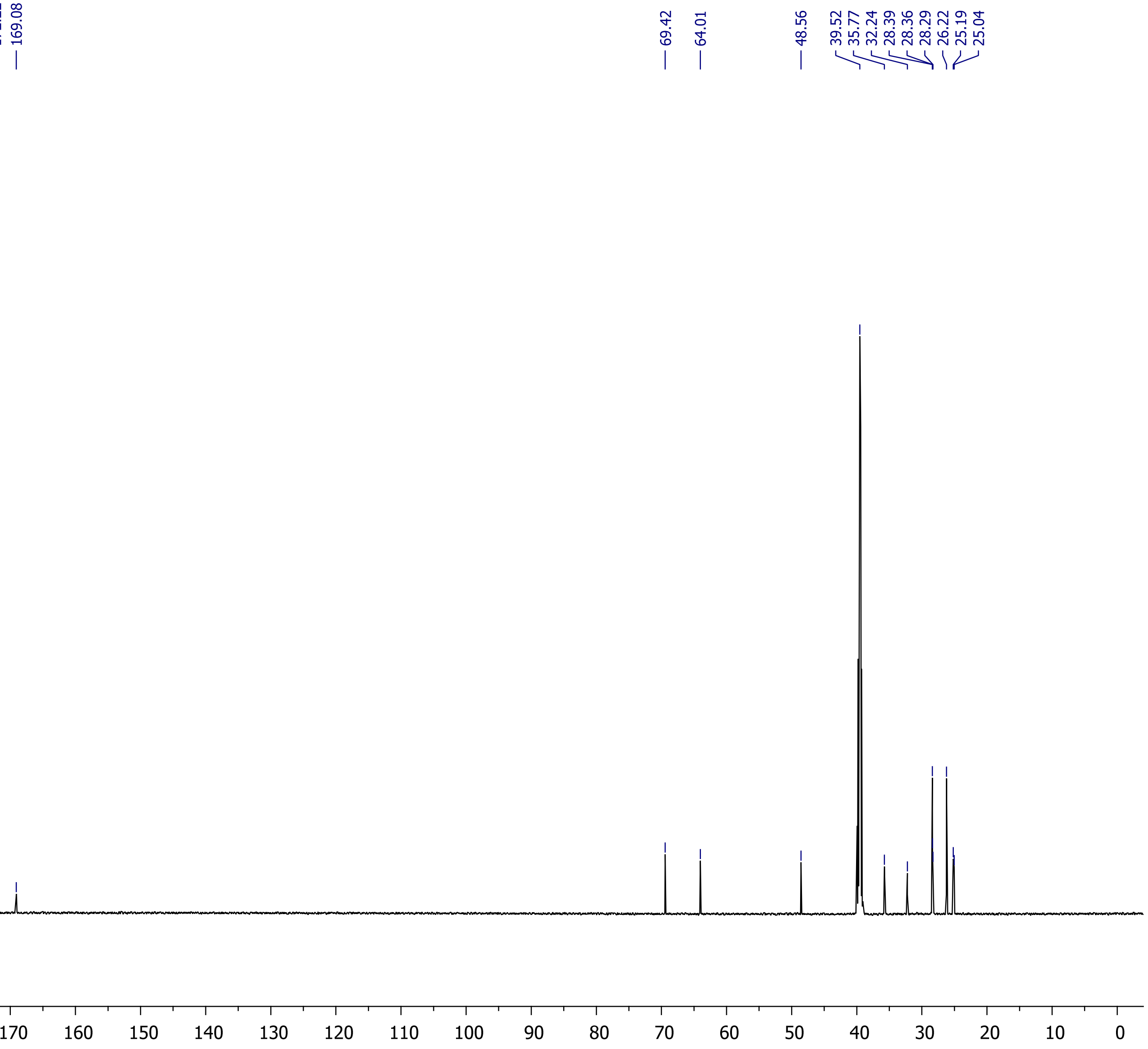

Compound SI-28

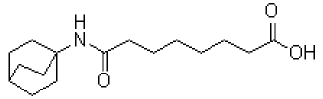

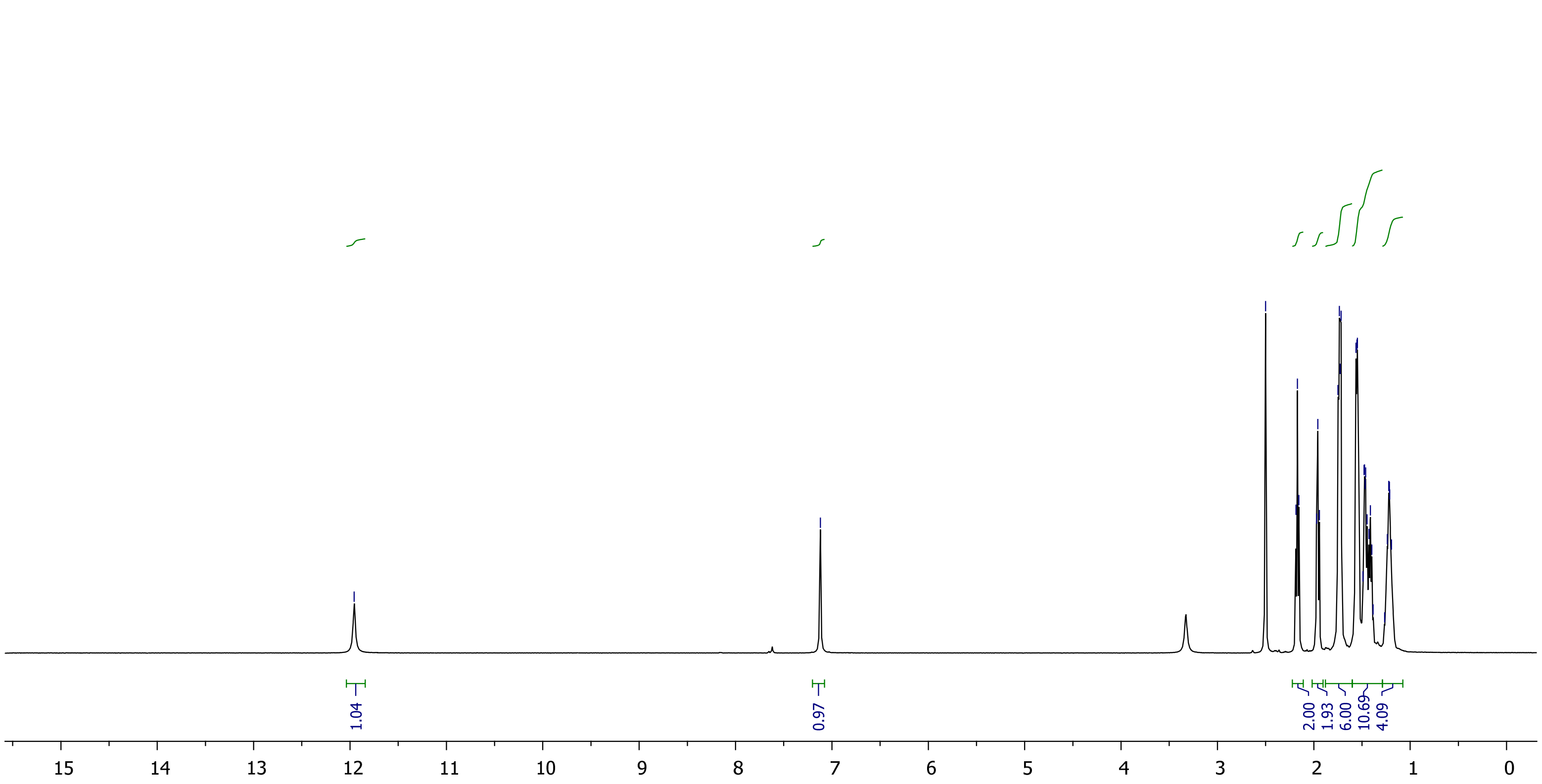

$^{13}C\{^{1}H\}$ NMR (151 MHz, DMSO-d_6)

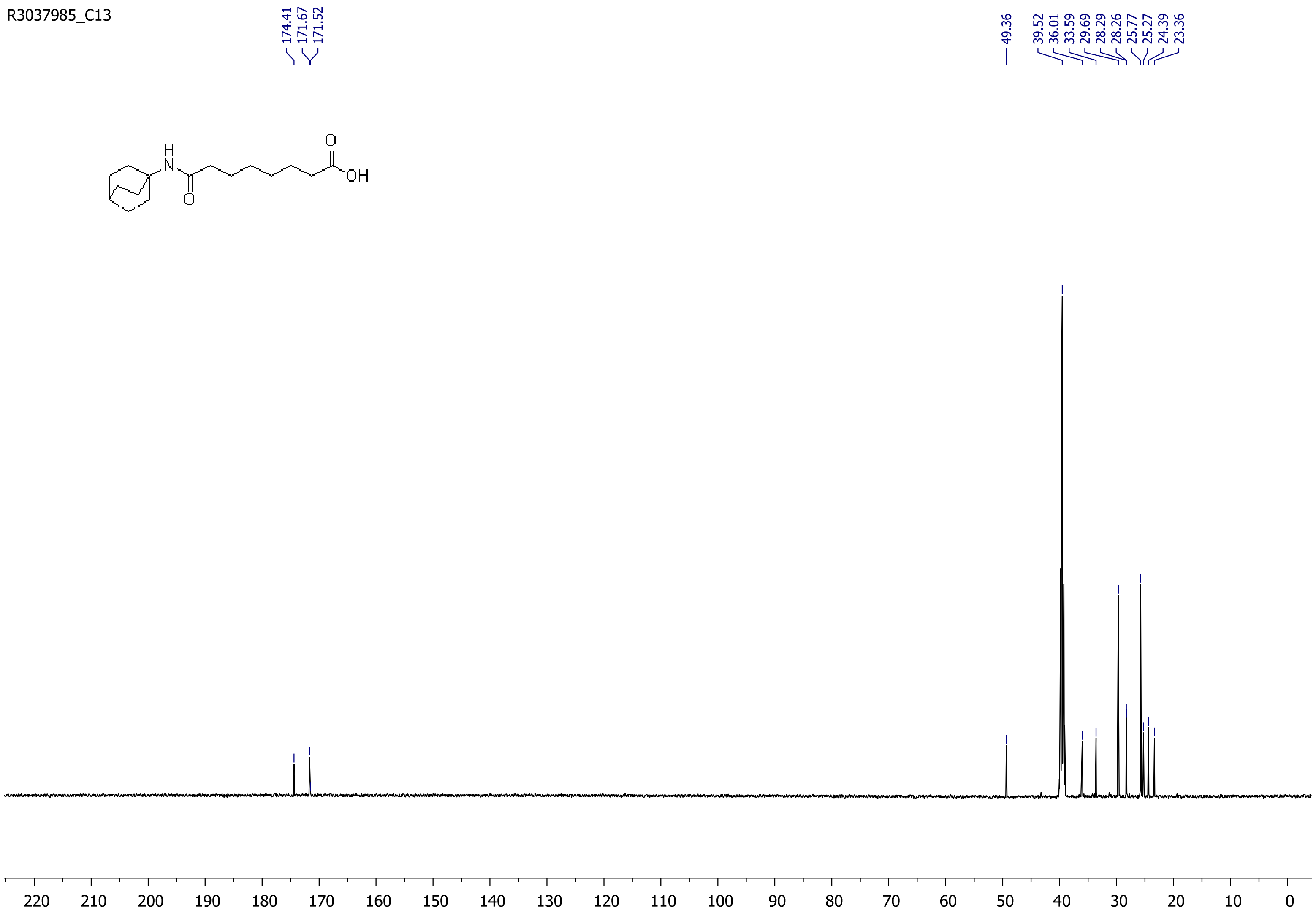

Compound 89

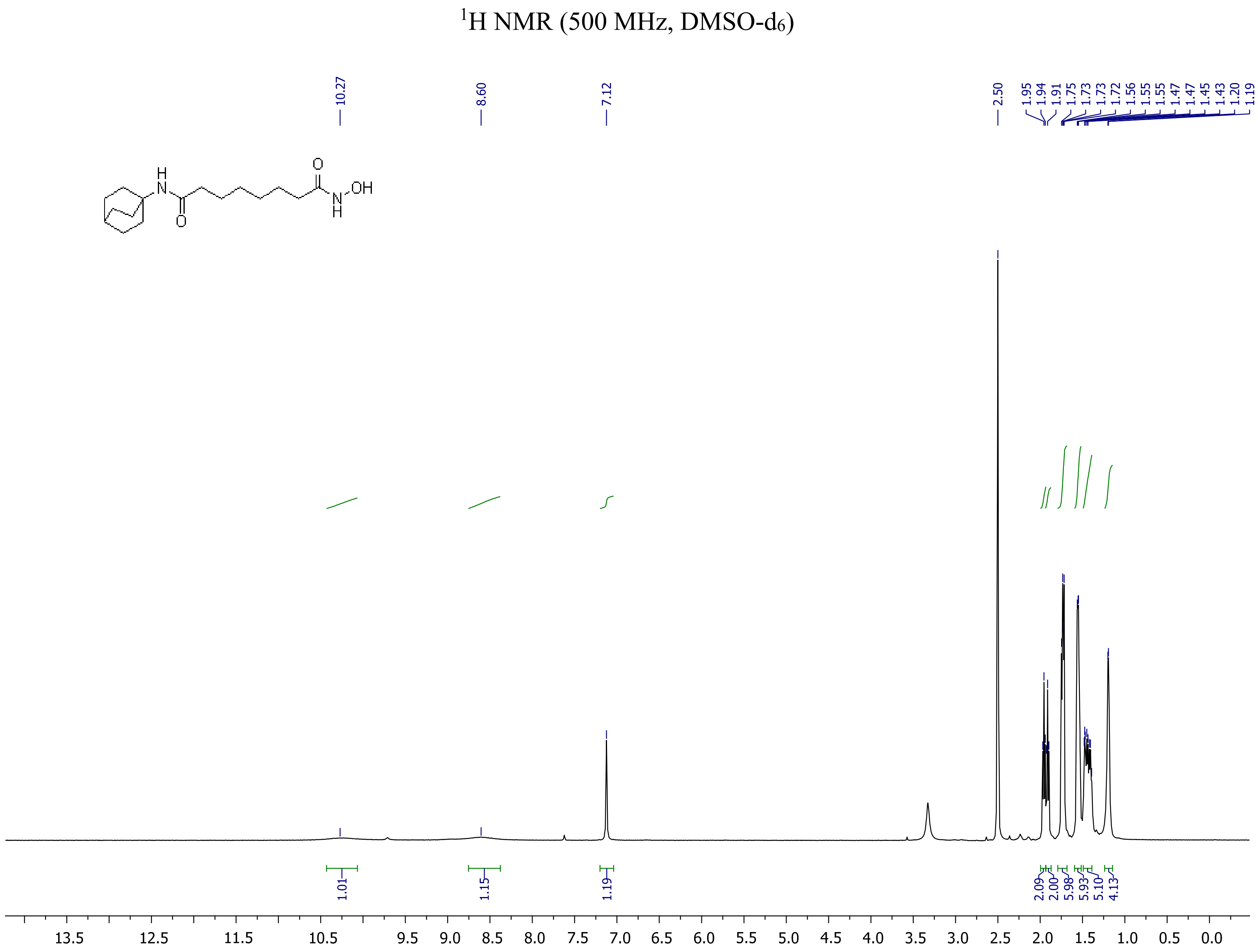

^{1}H NMR (500 MHz, DMSO-d$_6$)
10.27
8.60
7.12
2.50
1.95
1.94
1.91
1.75
1.73
1.73
1.72
1.56
1.55
1.55
1.47
1.45
1.43
1.20
1.19
1.01
1.15
1.19
2.09
2.00
5.98
5.93
5.10
4.13
13.5
12.5
11.5
10.5
9.5
9.0
8.5
8.0
7.5
7.0
6.5
6.0
5.5
5.0
4.5
4.0
3.5
3.0
2.5
2.0
1.5
1.0
0.5
0.0

$^{13}C\{^{1}H\}$ NMR (151 MHz, DMSO-d_6)

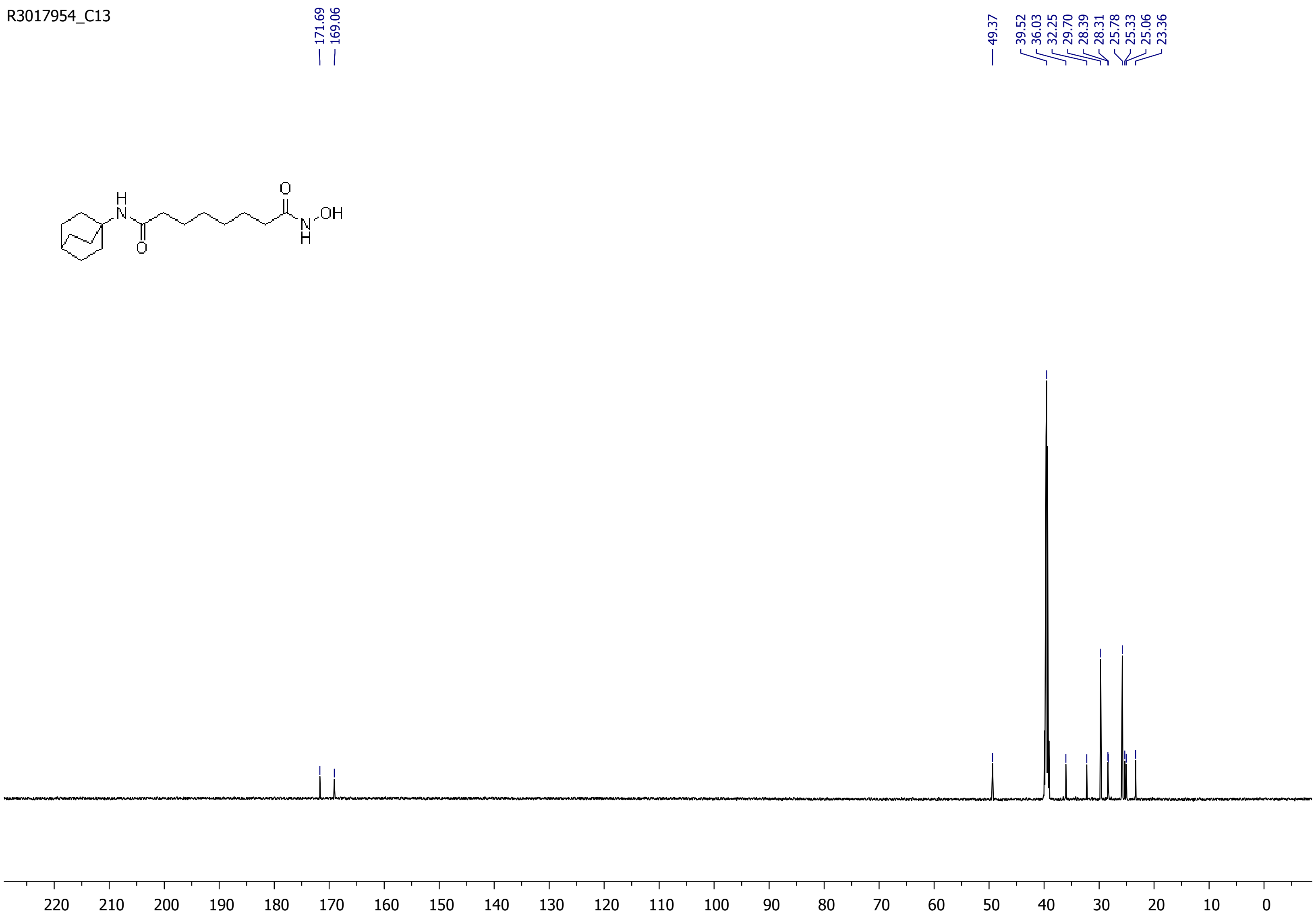

Crystallographic data (X-Ray)

Crystals of compounds **30**, **57**, **67**, **69** and **78** suitable for X-Ray diffraction studies were obtained by a low evaporation of a solution in methanol. Diffraction data were collected at room temperature on an Xcallibur-3 diffractometer with graphite-monochromated Mo Kα radiation (λ = 0.71073 Å) operating in the w-scans mode. The structure was solved by direct methods and refined by the full-matrix least-squares technique in the anisotropic approximation for non-hydrogen atoms using the SHELXTL program package. Crystallographic data for all structures in this paper have been deposited at Cambridge Crystallographic Data Centre. CCDC numbers: 2226162 (**30**), 2226164 (**57**), 2226872 (**67**), 2226163 (**69**) and 2266656 (**78**). Copies of the data can be obtained, free of charge, on application to CCDC, 12 Union Road, Cambridge CB21EZ, UK, (fax: +44-(0)1223-336033 or e-mail: deposit@ccdc.cam.ac.uk).

Compound **30b**

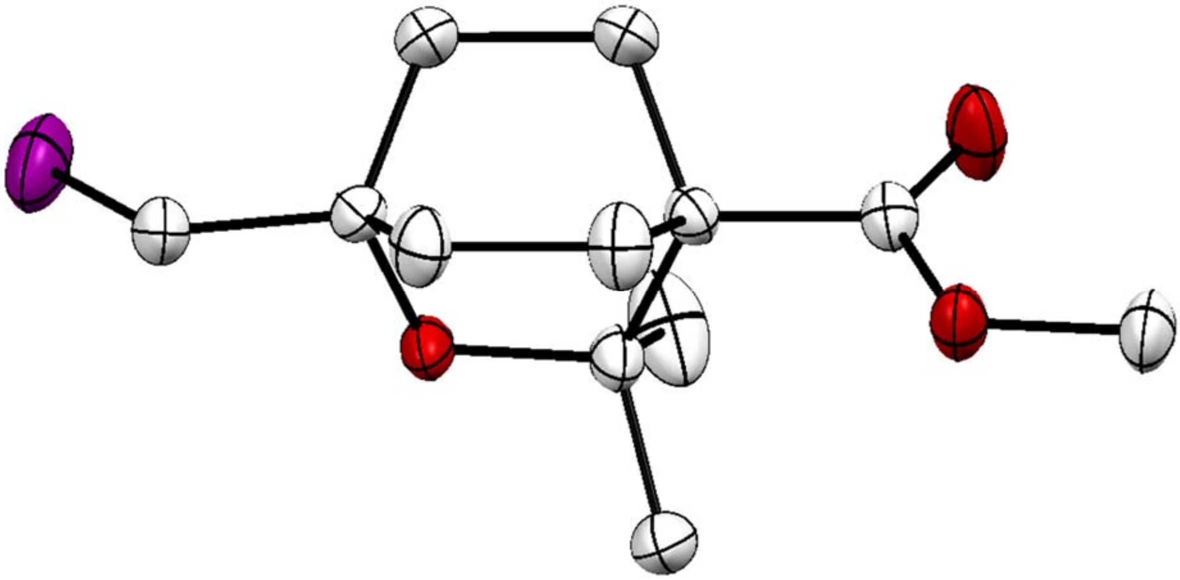

Supplementary Fig. 3. Molecular structure of **30** according to X-Ray diffraction data. Thermal ellipsoids are shown at 50% probability level.

Crystal structure determination of 30

data_v62

_chemical_formula_moiety 'C12 H19 I O3'

_chemical_formula_sum 'C12 H19 I O3'

_chemical_formula_weight 338.17

_space_group_crystal_system 'monoclinic'

_space_group_IT_number 14

_space_group_name_H-M_alt 'P 1 21/n 1'

_space_group_name_Hall '-P 2yn'

_cell_length_a 9.6946(17)

_cell_length_b 13.370(2)

_cell_length_c 10.843(2)

_cell_angle_alpha 90

_cell_angle_beta 106.970(10)

_cell_angle_gamma 90

_cell_volume 1344.3(4)

_cell_formula_units_Z 4

_cell_measurement_reflns_used 7682

_cell_measurement_temperature 296.15

_cell_measurement_theta_max 26.25

_cell_measurement_theta_min 2.48

_shelx_estimated_absorpt_T_max 0.797

_shelx_estimated_absorpt_T_min 0.648

_exptl_absorpt_coefficient_mu 2.374

_exptl_absorpt_correction_type none

_exptl_crystal_colour colourless

_exptl_crystal_colour_primary colourless

_exptl_crystal_density_diffrn 1.671

_exptl_crystal_description block

_exptl_crystal_F_000 672

_exptl_crystal_size_max 0.2

_exptl_crystal_size_mid 0.2

_exptl_crystal_size_min 0.1

_diffrn_reflns_av_R_equivalents 0.0541

_diffrn_reflns_av_unetI/netI 0.0273

_diffrn_reflns_Laue_measured_fraction_full 1.000

_diffrn_reflns_Laue_measured_fraction_max 1.000

_diffrn_reflns_limit_h_max 11

_diffrn_reflns_limit_h_min -11

_diffrn_reflns_limit_k_max 15

_diffrn_reflns_limit_k_min -15

_diffrn_reflns_limit_l_max 12

_diffrn_reflns_limit_l_min -12

_diffrn_reflns_number 18527

_diffrn_reflns_point_group_measured_fraction_full 1.000

_diffrn_reflns_point_group_measured_fraction_max 1.000

_diffrn_reflns_theta_full 24.999

_diffrn_reflns_theta_max 24.999

_diffrn_reflns_theta_min 2.483

_diffrn_ambient_temperature 296.15

_diffrn_measured_fraction_theta_full 1.000

_diffrn_measured_fraction_theta_max 1.000

_diffrn_measurement_device_type 'Bruker APEX-II CCD'

_diffrn_measurement_method '\f and \w scans'

_diffrn_radiation_type MoK\a

_diffrn_radiation_wavelength 0.71073

_diffrn_source_current 30.0

_diffrn_source_power 1.2

_diffrn_source_voltage 40.0

_diffrn_standards_number 0

_reflns_Friedel_coverage 0.000

_reflns_number_gt 1979

_reflns_number_total 2369

Compound **57**

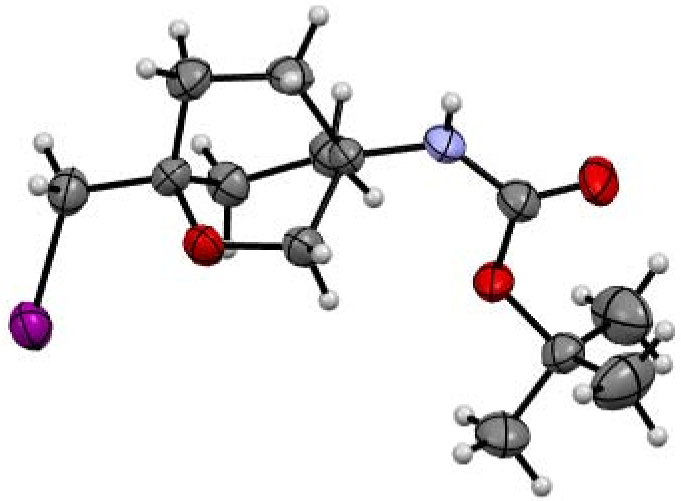

Supplementary Fig. 4. Molecular structure of **57** according to X-Ray diffraction data. Thermal ellipsoids are shown at 50% probability level.

Crystal structure determination of 57

data_v63

_chemical_formula_moiety 'C13 H22 I N O3'

_chemical_formula_sum 'C13 H22 I N O3'

_chemical_formula_weight 367.21

_space_group_crystal_system 'monoclinic'

_space_group_IT_number 14

_space_group_name_H-M_alt 'P 1 21/c 1'

_space_group_name_Hall '-P 2ybc'

_cell_length_a 11.113(2)

_cell_length_b 5.9106(11)

_cell_length_c 23.467(4)

_cell_angle_alpha 90

_cell_angle_beta 100.517(9)

_cell_angle_gamma 90

_cell_volume 1515.5(5)

_cell_formula_units_Z 4

_cell_measurement_reflns_used 4204

_cell_measurement_temperature 296.15
_cell_measurement_theta_max 24.00
_cell_measurement_theta_min 2.32
_shelx_estimated_absorpt_T_max 0.902
_shelx_estimated_absorpt_T_min 0.677
_exptl_absorpt_coefficient_mu 2.114
_exptl_absorpt_correction_type none
_exptl_crystal_colour colourless
_exptl_crystal_colour_primary colourless
_exptl_crystal_density_diffrn 1.609
_exptl_crystal_description needle
_exptl_crystal_F_000 736
_exptl_crystal_size_max 0.2
_exptl_crystal_size_mid 0.05
_exptl_crystal_size_min 0.05
_diffrn_reflns_av_R_equivalents 0.0776
_diffrn_reflns_av_unetI/netI 0.0469
_diffrn_reflns_Laue_measured_fraction_full 0.999
_diffrn_reflns_Laue_measured_fraction_max 0.999
_diffrn_reflns_limit_h_max 13
_diffrn_reflns_limit_h_min -13
_diffrn_reflns_limit_k_max 6
_diffrn_reflns_limit_k_min -7
_diffrn_reflns_limit_l_max 27
_diffrn_reflns_limit_l_min -27
_diffrn_reflns_number 19773
_diffrn_reflns_point_group_measured_fraction_full 0.999
_diffrn_reflns_point_group_measured_fraction_max 0.999
_diffrn_reflns_theta_full 25.000
_diffrn_reflns_theta_max 25.000
_diffrn_reflns_theta_min 2.322
_diffrn_ambient_temperature 296.15
_diffrn_measured_fraction_theta_full 0.999
_diffrn_measured_fraction_theta_max 0.999
_diffrn_measurement_device_type 'Bruker APEX-II CCD'

_diffrn_measurement_method '\f and \w scans'

_diffrn_radiation_type MoK\a

_diffrn_radiation_wavelength 0.71073

_diffrn_source_current 30.0

_diffrn_source_power 1.2

_diffrn_source_voltage 40.0

_diffrn_standards_number 0

_reflns_Friedel_coverage 0.000

_reflns_number_gt 2178

_reflns_number_total 2651

Compound **67**

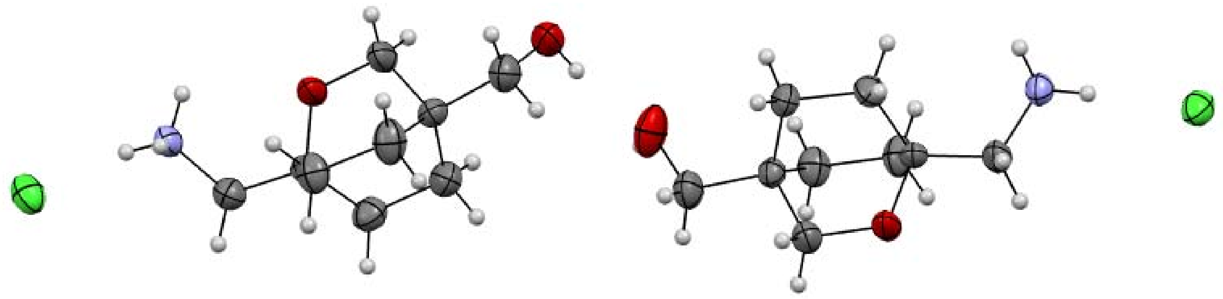

Supplementary Fig. 5. Molecular structure of **67** according to X-Ray diffraction data. Thermal ellipsoids are shown at 50% probability level.

Crystal structure determination of 67

data_v76

_chemical_formula_moiety 'Cl, C9 H18 N O2'

_chemical_formula_sum 'C9 H18 Cl N O2'

_chemical_formula_weight 207.69

_space_group_crystal_system 'monoclinic'

_space_group_IT_number 14

_space_group_name_H-M_alt 'P 1 21/n 1'

_space_group_name_Hall '-P 2yn'

_space_group_symop_operation_xyz

_cell_length_a 12.450(4)

_cell_length_b 6.248(2)

_cell_length_c 27.478(9)

_cell_angle_alpha 90

_cell_angle_beta 101.49(2)

_cell_angle_gamma 90

_cell_volume 2094.5(12)

_cell_formula_units_Z 8

_cell_measurement_reflns_used 2773

_cell_measurement_temperature 273.15

_cell_measurement_theta_max 20.56

_cell_measurement_theta_min 2.54

_shelx_estimated_absorpt_T_max 0.987

_shelx_estimated_absorpt_T_min 0.951

_exptl_absorpt_coefficient_mu 0.335

_exptl_absorpt_correction_type none

_exptl_crystal_colour colourless

_exptl_crystal_colour_primary colourless

_exptl_crystal_density_diffrn 1.317

_exptl_crystal_description plate

_exptl_crystal_F_000 896

_exptl_crystal_size_max 0.15

_exptl_crystal_size_mid 0.1

_exptl_crystal_size_min 0.04

_diffrn_reflns_av_R_equivalents 0.0833

_diffrn_reflns_av_unetI/netI 0.0539

_diffrn_reflns_Laue_measured_fraction_full 0.999

_diffrn_reflns_Laue_measured_fraction_max 0.999

_diffrn_reflns_limit_h_max 14

_diffrn_reflns_limit_h_min -14

_diffrn_reflns_limit_k_max 7

_diffrn_reflns_limit_k_min -7

_diffrn_reflns_limit_l_max 32

_diffrn_reflns_limit_l_min -32

_diffrn_reflns_number 24591

_diffrn_reflns_point_group_measured_fraction_full 0.999

_diffrn_reflns_point_group_measured_fraction_max 0.999

_diffrn_reflns_theta_full 24.998

_diffrn_reflns_theta_max 24.998

_diffrn_reflns_theta_min 1.690

_diffrn_ambient_temperature 273.15

_diffrn_measured_fraction_theta_full 0.999

_diffrn_measured_fraction_theta_max 0.999

_diffrn_measurement_device_type 'Bruker APEX-II CCD'

_diffrn_measurement_method '\f and \w scans'

_diffrn_radiation_type MoK\a

_diffrn_radiation_wavelength 0.71073

_diffrn_source_current 30.0

_diffrn_source_power 1.2

_diffrn_source_voltage 40.0

_diffrn_standards_number 0

_reflns_Friedel_coverage 0.000

_reflns_number_gt 2502

_reflns_number_total 3697

Compound **69**

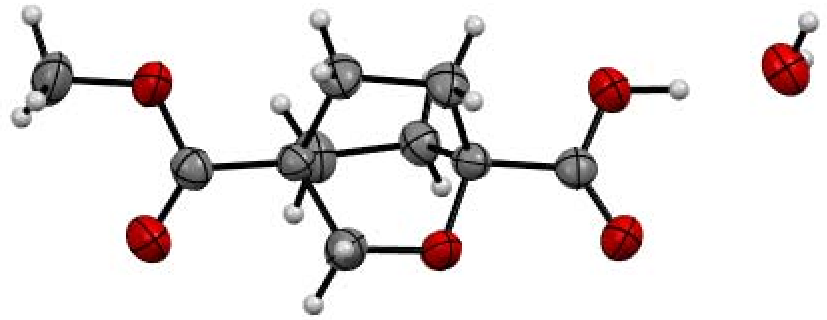

Supplementary Fig. 6. Molecular structure of **69** according to X-Ray diffraction data. Thermal ellipsoids are shown at 50% probability level.

Crystal structure determination of 69

data_v64

_chemical_formula_moiety 'C10 H14 O5, H2 O'

_chemical_formula_sum 'C10 H16 O6'

_chemical_formula_weight 232.23

_space_group_crystal_system 'monoclinic'

_space_group_IT_number 14

_space_group_name_H-M_alt 'P 1 21/c 1'

_space_group_name_Hall '-P 2ybc'

_cell_length_a 15.2107(11)

_cell_length_b 5.9409(4)

_cell_length_c 12.4791(9)

_cell_angle_alpha 90

_cell_angle_beta 101.580(4)

_cell_angle_gamma 90

_cell_volume 1104.72(14)

_cell_formula_units_Z 4

_cell_measurement_reflns_used 3925

_cell_measurement_temperature 273.15

_cell_measurement_theta_max 26.26

_cell_measurement_theta_min 2.73

_shelx_estimated_absorpt_T_max 0.993

_shelx_estimated_absorpt_T_min 0.977

_exptl_absorpt_coefficient_mu 0.116

_exptl_absorpt_correction_type none

_exptl_crystal_colour 'light yellow'

_exptl_crystal_colour_modifier light

_exptl_crystal_colour_primary yellow

_exptl_crystal_density_diffrn 1.396

_exptl_crystal_description block

_exptl_crystal_F_000 496

_exptl_crystal_size_max 0.2

_exptl_crystal_size_mid 0.06

_exptl_crystal_size_min 0.06

_diffrn_reflns_av_R_equivalents 0.0426

_diffrn_reflns_av_unetI/netI 0.0286

_diffrn_reflns_Laue_measured_fraction_full 0.999

_diffrn_reflns_Laue_measured_fraction_max 0.999

_diffrn_reflns_limit_h_max 18

_diffrn_reflns_limit_h_min -18
_diffrn_reflns_limit_k_max 6
_diffrn_reflns_limit_k_min -7
_diffrn_reflns_limit_l_max 14
_diffrn_reflns_limit_l_min -14
_diffrn_reflns_number 14878
_diffrn_reflns_point_group_measured_fraction_full 0.999
_diffrn_reflns_point_group_measured_fraction_max 0.999
_diffrn_reflns_theta_full 24.992
_diffrn_reflns_theta_max 24.992
_diffrn_reflns_theta_min 2.734
_diffrn_ambient_temperature 273.15
_diffrn_measured_fraction_theta_full 0.999
_diffrn_measured_fraction_theta_max 0.999
_diffrn_measurement_device_type 'Bruker APEX-II CCD'
_diffrn_measurement_method '\f and \w scans'
_diffrn_radiation_type MoK\a
_diffrn_radiation_wavelength 0.71073
_diffrn_source_current 30.0
_diffrn_source_power 1.2
_diffrn_source_voltage 40.0
_reflns_Friedel_coverage 0.000
_reflns_number_gt 1427
_reflns_number_total 1940

Compound **78**

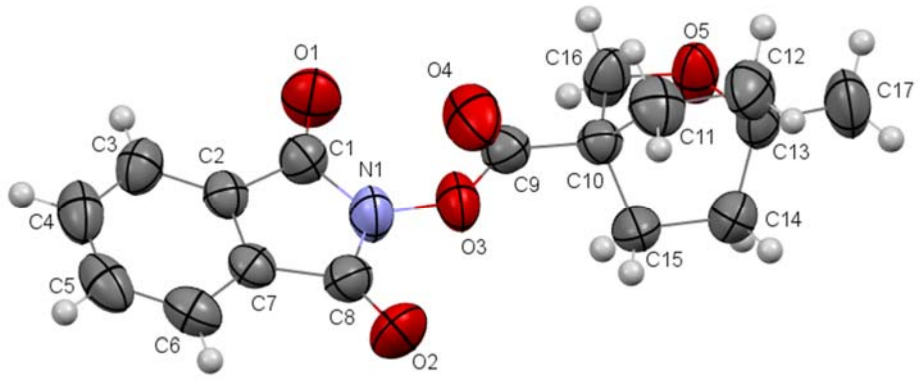

Supplementary Fig. 7. Molecular structure of **78** according to X-Ray diffraction data. Thermal ellipsoids are shown at 50% probability level.

Crystal structure determination of 78

data_vd189

_chemical_formula_moiety 'C17 H17 N O5'

_chemical_formula_sum 'C17 H17 N O5'

_chemical_formula_weight 315.31

_space_group_crystal_system 'triclinic'

_space_group_IT_number 2

_space_group_name_H-M_alt 'P -1'

_space_group_name_Hall '-P 1'

_cell_length_a 8.811(2)

_cell_length_b 9.891(2)

_cell_length_c 10.611(2)

_cell_angle_alpha 109.348(12)

_cell_angle_beta 94.652(13)

_cell_angle_gamma 111.984(12)

_cell_volume 786.5(3)

_cell_formula_units_Z 2

_cell_measurement_reflns_used 4965

_cell_measurement_temperature 296.15

_cell_measurement_theta_max 24.67

_cell_measurement_theta_min 2.41

_shelx_estimated_absorpt_T_max 0.988

_shelx_estimated_absorpt_T_min 0.977

_exptl_absorpt_coefficient_mu 0.099

_exptl_absorpt_correction_type none

_exptl_crystal_colour colourless

_exptl_crystal_colour_primary colourless

_exptl_crystal_density_diffrn 1.331

_exptl_crystal_description block

_exptl_crystal_F_000 332

_exptl_crystal_size_max 0.24

_exptl_crystal_size_mid 0.15

_exptl_crystal_size_min 0.12

_diffrn_reflns_av_R_equivalents 0.0398

_diffrn_reflns_av_unetI/netI 0.0297

_diffrn_reflns_Laue_measured_fraction_full 0.999

_diffrn_reflns_Laue_measured_fraction_max 0.999

_diffrn_reflns_limit_h_max 10

_diffrn_reflns_limit_h_min -10

_diffrn_reflns_limit_k_max 11

_diffrn_reflns_limit_k_min -11

_diffrn_reflns_limit_l_max 12

_diffrn_reflns_limit_l_min -12

_diffrn_reflns_number 11060

_diffrn_reflns_point_group_measured_fraction_full 0.999

_diffrn_reflns_point_group_measured_fraction_max 0.999

_diffrn_reflns_theta_full 24.995

_diffrn_reflns_theta_max 24.995

_diffrn_reflns_theta_min 2.093

_diffrn_ambient_temperature 296.15

_diffrn_measured_fraction_theta_full 0.999

_diffrn_measured_fraction_theta_max 0.999

_diffrn_measurement_device_type 'Bruker APEX-II CCD'

_diffrn_measurement_method '\f and \w scans'

_diffrn_radiation_type MoK\a

_diffrn_radiation_wavelength 0.71073

_diffrn_source_current	30.0
_diffrn_source_power	1.2
_diffrn_source_voltage	40.0
_diffrn_standards_number	0
_reflns_Friedel_coverage	0.000

Superposition of f 2-oxabicyclo[2.2.2]octane and *para*-substituted phenyl ring

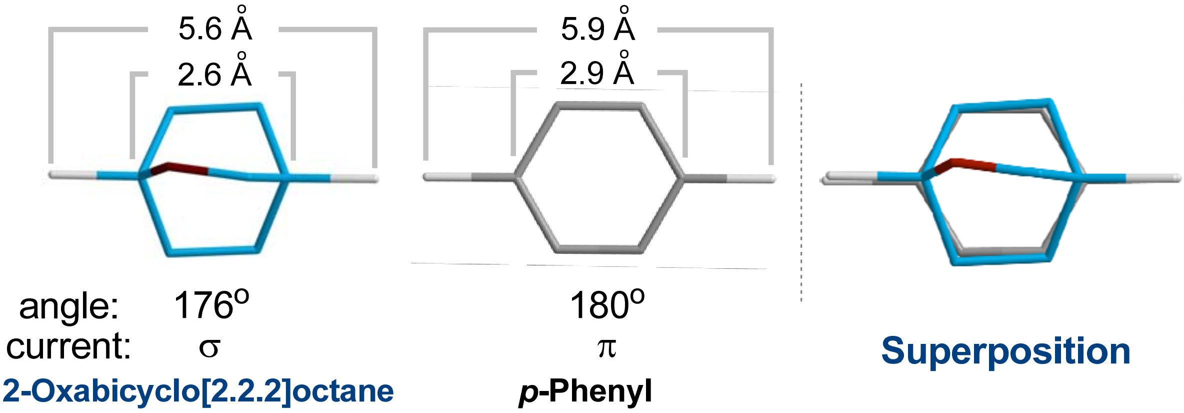

Supplementary Fig. 8. Visualized comparison of 2-oxabicyclo[2.2.2]octane and *para*-substituted phenyl ring. The ideal angle (180°) for the *para*-substituted phenyl ring is given (the observed angle in Imatinib is 176-179°).

Analysis of Aqueous Solubility

Test articles EN300-123057 (Imatinib), EN300-37156110 (**85**) and EN300-37350388 (**86**) and reference compound (Ondansetron) were assessed for kinetic solubility in phosphate-buffered saline, pH 7.4.

Reagents and consumables

Phosphate buffered saline, pH 7.4 (Sigma-Aldrich, USA; Cat #P3813)
Acetonitrile Chromasolv, gradient grade, for HPLC, ≥99.9% (Sigma-Aldrich, USA; Cat #34851)
Ondansetron base powder (Enamine, Ukraine, Cat # EN300-117273)
DMSO (Sigma-Aldrich, USA; Cat # 34869)
Costar 96 Well Assay Blocks (Corning, USA; Cat # 3958)
MultiScreen HTS 96 Well Filter Plates (Millipore, Ireland; Cat # MSGVS2210)
UV-Star® 96 Well Microplate (Greiner Bio-One, Germany; Cat #655801)
Matrix Disposable pipette tips (ThermoScientific, USA; Cat ## 8041, 7622, 7321)
Flex-Tubes Microcentrifuge Tubes, 1.5ml (Eppendorf, Germany; Cat # 22364111)
Matrix Storage tubes, 1.4 ml (ThermoScientific, USA; Cat # 4247)

Equipment

Water purification system Millipore Milli-Q Gradient A10 (Millipore, France)
Thermomixer R Block, 1.5 mL (Eppendorf, Germany; Cat # 5355)
Matrix Multichannel Electronic Pipette 2-125 µL, 5-250 µL, 15-1250 µL (Thermo Scientific, USA; Cat ## 2011, 2012, 2004)
SpectraMax Plus Microplate Reader (Molecular Devices, USA; Product # 02196)
Multi-Well Plate Vacuum Manifold (Pall Corporation, USA; Product # 5014)
Vacuum pump (Millipore, USA; Model # XX5500000)

Analytical System

The measurements were performed using SpectraMax Plus reader in UV-Vis mode. Acquisition and analysis of the data were performed using SoftMax Pro v.5.4 (Molecular Devices) and Excel 2010 data analysis software.

Methods

Kinetic solubility assay was performed according to the Enamine's aqueous solubility SOP. Briefly, using a 20 mM stock solution of the compound in 100% DMSO dilutions were prepared to a theoretical concentration of 400 µM in duplicates in phosphate-buffered saline pH 7.4

(138 mM NaCl, 2.7 mM KCl, 10 mM K-phosphate) with 2% final DMSO. The experimental compound dilutions in PBS were further allowed to equilibrate at 25 °C on a thermostatic shaker for two hours and then filtered through HTS filter plates using a vacuum manifold. The filtrates of test compounds were diluted 2-fold with acetonitrile with 2% DMSO before measuring.

In parallel, compound dilutions in acetonitrile/PBS (1:1) were prepared to theoretical concentrations of 0 µM (blank), 10 µM, 25 µM, 50 µM, 100 µM, and 200 µM with 2% final DMSO to generate calibration curves. Ondansetron was used as reference compound to control proper assay performance. 200 µl of each sample was transferred to 96-well plate and measured in 200-550 nm range with 5 nm step.

The concentrations of compounds in PBS filtrate are calculated using a dedicated Microsoft Excel calculation script. Proper absorbance wavelengths for calculations are selected for each compound manually based on absorbance maximums (absolute absorbance unit values for the minimum and maximum concentration points within 0 – 3 OD range). Each of the final datasets is additionally visually evaluated by the operator and goodness of fit (R2) is calculated for each calibration curve. The effective range of this assay is approximately 2-400 µM and the compounds returning values close to the upper limit of the range may have higher actual solubility (e.g. 5'-deoxy-5-fluorouridine). This method is not suitable for liquid (at 25 °C) substances (were not present among the tested compounds).

Results

Supplementary Tab. 1. The solubility data of the test and reference compounds The calibration curves are shown in the Appendix*.

ID	wavelength	concentration	total incubation volume	pH conditions	incubation time, h	Solubility
EN300-123057 Imatinib	260	400	200	7,4	2	351
EN300-37156110 (**85**)	260	400	200	7,4	2	389
EN300-37350388 (**86**)	260	200	200	7,4	2	113
Ondansetron	300	400	200	7,4	2	126

*Goodness of fit (R^2) in all titration curves as well as the variations between repeat measurements indicates high quality of the experimental data in the current batch of test articles.

**Ondansetron solubility data are consistent with previously obtained.

APPENDIX

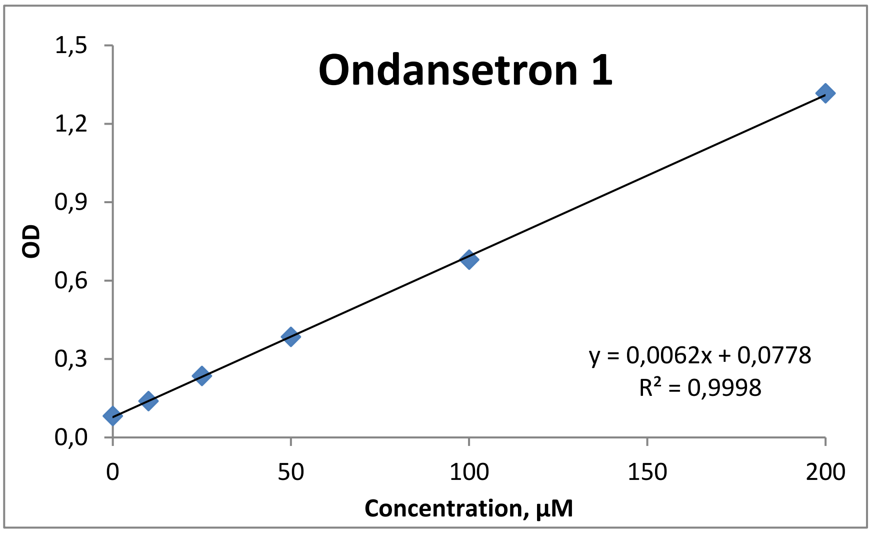

Supplementary Fig. 9. Calibration curve for Ondansetron

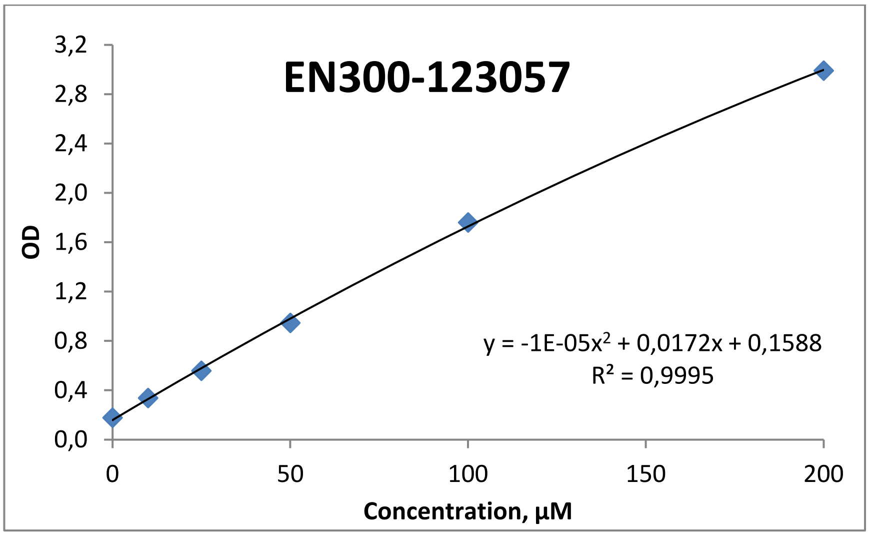

Supplementary Fig. 10. Calibration curve for EN300-123057 (Imatinib)

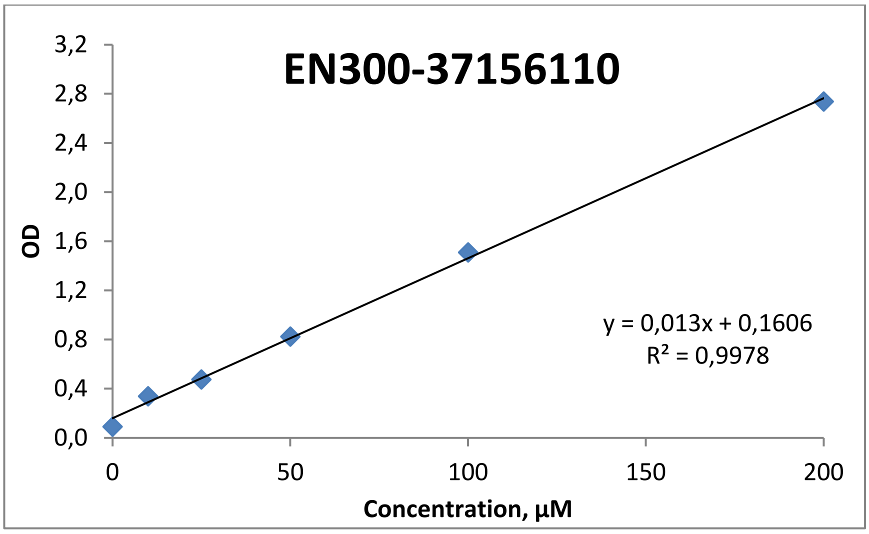

Supplementary Fig. 11. Calibration curve for EN300-37156110 (**85**)

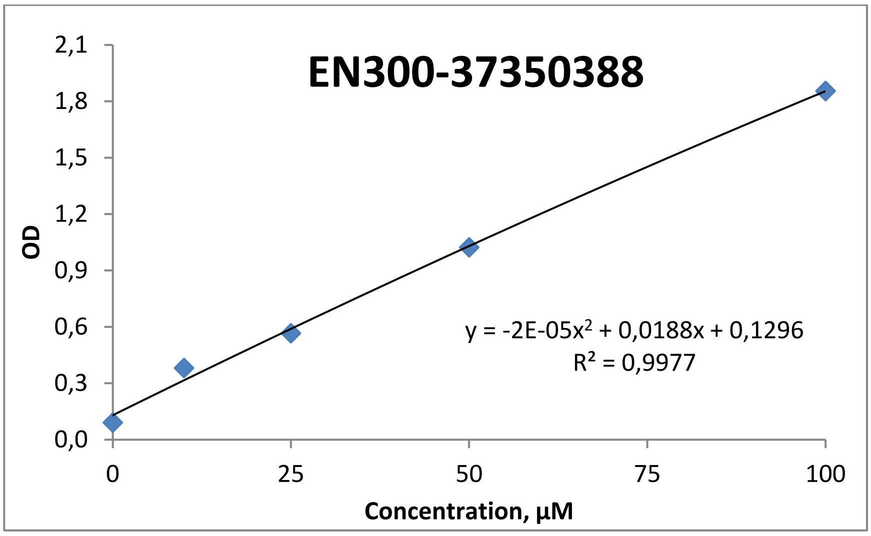

Supplementary Fig. 12. Calibration curve for EN300-37350388 (**86**)

Determination of Distribution Coefficient (LogD, pH 7.4)

Test articles EN300-123057 (Imatinib), EN300-37156110 (**85**), EN300-37350388 (**86**) and reference compound (Mebendazole) in *n*-octanol – phosphate buffered saline (PBS), pH 7.4. Distribution coefficient (or LogD) is a logarithm of the ratio of drug concentrations in two immiscible solvents, typically pH-buffered water and *n*-octanol. It is a measure of hydrophobic/hydrophilic properties of a given molecule. The partition of test compounds is determined using a shake-flask method, which involves mixing of a certain amount of the solute of interest in defined volumes of *n*-octanol and an aqueous buffer of choice followed by equilibration of the mixture by incubation with efficient mixing. Then, the distribution of the compounds in each solvent was controlled using LC-MS/MS.

Reagents and consumables

DMSO Chromasolv Plus, HPLC grade, ≥99.7% (Sigma-Aldrich, USA; Cat #34869)
Acetonitrile Chromasolv, gradient grade, for HPLC, ≥99.9% (Sigma-Aldrich, USA; Cat #34851)
Formic acid for mass spectrometry, ~98% (Fluka, USA; Cat #94318)
Phosphate buffered saline, tablet (Sigma-Aldrich, USA; Cat # P4417)
Acetic acid (Enamine, Ukraine.)
1-Octanol ACS grade, ≥99% (Sigma-Aldrich, USA; Cat # 472328)
Mebendazole analytical standard, ≥ 98%, HPLC (Sigma-Aldrich, USA; Cat # M2523)
DMSO stock solutions of the test compounds 10mM
Phenomenex Luna® C18 HPLC column, 2.1 × 50 mm, 5 µm (Cat #5291-126)
1.1 mL microtubes in microracks, pipettor tips (Thermo Scientific, USA).
National Scientific MicroTube™ Rack (Thermo Fisher Scientific, USA; Cat # TN094612R)

Equipment

Gradient HPLC system (Shimadzu, Japan)
Triple quadrapole mass-detector API 3000 with TurboIonSpray Ion Source (AB Sciex, Canada)
VWR Membrane Nitrogen Generators N2-04-L1466, nitrogen purity 99%+ (VWR, USA)
MTR22 Multi Mix Rotator (UNICO, USA)
Laboratory Centrifuge, Sigma 4-15C, Qiagen (SIGMA GmbH, Germany)
Water purification system Millipore Milli-Q Gradient A10 (Millipore, France)
Multichannel Electronic Pipettes 0.5-12.5 µL, 2-125 µL, 5-250 µL, 15-1250 µL, Matrix (Thermo Scientific, USA; Cat ## 2009, 2001, 2002, 2004)

Analytical System

All measurements were performed using a Shimadzu Prominence HPLC system including a vacuum degasser, gradient pumps, a reverse phase column, a column oven and an autosampler. Mass spectrometric analysis was performed using an API 4000 QTRAP mass spectrometer from Applied Biosystems/MDS Sciex (AB Sciex) with Turbo V ion source and TurboIonspray interface. The TurboIonSpray ion source was used in both positive and negative ion modes. Acquisition and analysis of the data were performed using Analyst 1.6.3 software.

Methods

Incubations were carried out in Eppendorf-type polypropylene microtubes in triplicates. 5 μL aliquot of 10 mM DMSO stock of a test compound was added into the previously mutually saturated mixture containing 500 μL of PBS (pH 7.4) and 500 μL of octanol. The solution was allowed to mix in a rotator for 1 h at 30 rpm. Phase separation was assured by centrifugation for 2 min at 5800 x g. The octanol phase was diluted 100-fold with 40% acetonitrile, and the aqueous phase (PBS buffer) was diluted 10-fold; for compounds Mebendazole, EN300-123057 (Imatinib), EN300-37156110 (**85**) and EN300-37350388 (**86**). The aqueous phase was analyzed without dilution. The samples (both phases) were analyzed using a HPLC system coupled with a tandem mass spectrometer. Mebendazole was used as a reference compound.

Calculations of the partition ratios were carried out using the equation below.

$$D = (d_o \times S_o) / (d_p \times S_p)$$

where: S_o – peak area of the analyte in octanol phase

S_p – peak area of the analyte in PBS buffer

d_o – dilution coefficient for octanol phase

d_p – dilution coefficient for aqueous phase

Results

LogD data for the reference compound (Mebendazole) and test compound is provided in the table below.

Supplementary Tab. 2. Experimental LogD, pH 7.4

	Mebendazole					
	Sample Name	RT(min)	Dilution	Area	Av. Area	LogD
1	Mebendazole-p-1-1	1,23	1	7,31E+05	6,98E+05	2,954
2	Mebendazole-p-1-2	1,22	1	6,78E+05		2,992
3	Mebendazole-p-1-3	1,22	1	6,85E+05		2,967
4	Mebendazole-o-100-1	1,22	100	6,56E+06	6,52E+06	
5	Mebendazole-o-100-2	1,22	100	6,65E+06		
6	Mebendazole-o-100-3	1,22	100	6,34E+06		
				LogD	2,98	

	EN300 123057 (Imatinib)					
	Sample Name	RT(min)	Dilution	Area	Av. Area	LogD
1	EN300 123057-p-1-1	1,04	1	1,10E+05	1,11E+05	2,675
2	EN300 123057-p-1-2	1,05	1	1,05E+05		2,64
3	EN300 123057-p-1-3	1,05	1	1,19E+05		2,595
4	EN300 123057-o-100-1	1,05	100	5,20E+05	4,82E+05	
5	EN300 123057-o-100-2	1,05	100	4,57E+05		
6	EN300 123057-o-100-3	1,06	100	4,70E+05		
				LogD	2,64	

	EN300 37156110 (**85**)					
	Sample Name	RT(min)	Dilution	Area	Av. Area	LogD
1	EN300 37156110-p-1-1	1,04	1	6,52E+05	6,57E+05	1,766
2	EN300 37156110-p-1-2	1,05	1	6,76E+05		1,798
3	EN300 37156110-p-1-3	1,05	1	6,43E+05		1,809
4	EN300 37156110-o-100-1	1,04	100	3,80E+05	4,06E+05	
5	EN300 37156110-o-100-2	1,04	100	4,24E+05		
6	EN300 37156110-o-100-3	1,05	100	4,14E+05		
				LogD	1,8	

	EN300 37350388 (**86**)					
	Sample Name	RT(min)	Dilution	Area	Av. Area	LogD
1	EN300 37350388-p-1-1	1,05	1	2,14E+05	2,06E+05	2,716
2	EN300 37350388-p-1-2	1,05	1	2,03E+05		2,741
3	EN300 37350388-p-1-3	1,06	1	2,03E+05		2,744
4	EN300 37350388-o-100-1	1,06	100	1,11E+06	1,12E+06	
5	EN300 37350388-o-100-2	1,05	100	1,11E+06		
6	EN300 37350388-o-100-3	1,05	100	1,13E+06		
				LogD	2,74	

Assessment of Metabolic Stability in Human Liver Microsomes

The objective of this study was to determine metabolic stability of 3 test articles EN300-123057 (Imatinib), EN300-37156110 (**85**) and EN300-37350388 (**86**) and reference compounds in human liver microsomes at five time points over 40 minutes using HPLC-MS. Metabolic stability is defined as the percentage of parent compound lost over time in the presence of a metabolically active test system.

Reagents and consumables

DMSO (Sigma-Aldrich, 34869 - Chromasolv Plus, for HPLC, ≥99.7%)
Acetonitrile (Sigma-Aldrich, 34851 - Chromasolv Plus, for HPLC, ≥99.9%)
Methanol, for HPLC, ≥99.9% (Sigma-Aldrich, Cat #34860)
Potassium phosphate monobasic (Helicon, Am-O781-0.5)
Potassium phosphate dibasic (Helicon, Am-O705-0.5)
Magnesium chloride hexahydrate (Helicon, Am-O288-0.1)
Human Liver Microsomes: pooled, mixed gender (XenoTech, H0630/lot #2010065)
Glucose-6-phosphate dehydrogenase from baker's yeast, type XV (Sigma-Aldrich, USA; G6378)
D-Glucose-6-phosphate monosodium salt (Santa Cruz Biotechnology, Inc., USA; sc-210728)
β-Nicotinamide adeninedinucleotide-2'-phosphate reduced, tetrasodium salt (Sigma Aldrich, USA; Cat #N1630)
Formic acid (Sigma-Aldrich, USA; 94318)
Niclosamide (Sigma-Aldrich, USA; Cat #N3510)
Verapamil hydrochloride (Sigma Aldrich, USA; Cat #V4629)
(+,-) Propranolol hydrochloride (Sigma-Aldrich, USA; P0884)
Diclofenac, 96% purity (Enamine, # EN300-119509)
Phenomenex Luna® C18 HPLC column, 2.1x50 mm, 5 µm (Cat #5291-126)
Matrix™ 0.75 ml blank tubes (Cat #4170), pipettor tips (Thermo Scientific).

Equipment

Gradient HPLC system (Shimadzu)

API 4000 QTRAP mass spectrometer with Turbo V ion source (AB Sciex)
Triple quadrapole mass-detector API 5000 with Turbo V Ion Source (AB Sciex, Canada)
Nitrogen generator N2-04-L1466, nitrogen purity 99%+ (Whatman)

Incubator/Shaker Innova 4080 (New Brunswick Scientific, USA)
Water purification system Millipore Milli-Q Gradient A10 (Millipore, France)
Multichannel pipettors 1-30 µL, 2-125 µL, 30-850 µL (Thermo Scientific)

Analytical System

All measurements were performed using Shimadzu HPLC system including vacuum degasser, gradient pumps, reverse phase HPLC column, column oven, and autosampler. Mass spectrometric analysis was performed using an API 4000 QTRAP mass spectrometer with Turbo V ion source (AB Sciex) and Triple quadrapole mass-detector API 5000 with Turbo V Ion Source (AB Sciex, Canada). The TurboIonSpray ion source was used in both positive and negative ion modes. The data acquisition and system control was performed using Analyst 1.6.3 software from AB Sciex.

Methods

Microsomal incubations were carried out in 96-well plates in 5 aliquots of 30 µL each (one for each time point). Liver microsomal incubation medium comprised of phosphate buffer (100 mM, pH 7.4), $MgCl_2$ (3.3 mM), NADPH (3 mM), glucose-6-phosphate (5.3 mM), glucose-6-phosphate dehydrogenase (0.67 units/mL) with 0.42 mg of liver microsomal protein per mL. In the control reactions the NADPH-cofactor system was substituted with phosphate buffer. Test compounds (2 µM, final solvent concentration 1.6%) were incubated with microsomes at 37 °C, shaking at 100 rpm. Each reaction was performed in duplicates. Five time points over 40 minutes were analyzed. The reactions were stopped by adding 5 volumes of methanol containing internal standard to incubation aliquots, followed by protein sedimentation by centrifuging at 4870 x g for 4 minutes. Supernatants were analyzed using the HPLC system coupled with tandem mass spectrometer.
The elimination constant (k_{el}), half-life ($t_{1/2}$) and intrinsic clearance (Cl_{int}) were determined in plot of ln(AUC) versus time, using linear regression analysis. In order to indicate the quality of the linear regression analysis, the R (correlation coefficient) values are provided. In some cases, the last time point is excluded from the calculations to ensure acceptable logarithmic linearity of decay.

$k_{el} = -slope$

$t_{1/2} = 0.693 / k$

$CL_{int} = (0.693 / t_{1/2}) \times (\mu L_{incubation} / mg_{microsomoes})$

Results

Human microsomal stability data for reference and test compounds is provided in the tables below.

Supplementary Tab. 3. Human microsomal stability (batch #1)

Compound ID	Time, min	Peak Area Ratio		Peak Area Ratio, Mean of 2	% Remaining, Mean of 2	R	k_{el}, min^{-1}	$t_{1/2}$, min	Cl_{int}, µl/min/mg	% Remaining without cofactor, Mean of 2
		Inc. 1	Inc. 2							
1	2	3	4	5	6	7	8	9	10	11
Diclofenac human	0	1.02E-01	1.06E-01	1.04E-01	100	1.000	0.123	5.6	296	100
	7	5.21E-02	4.87E-02	5.04E-02	48	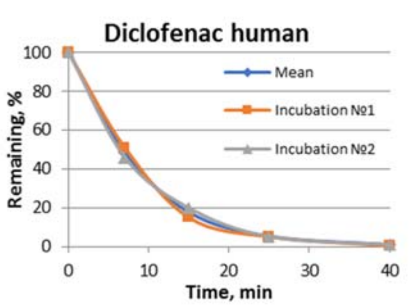				
	15	1.54E-02	2.10E-02	1.82E-02	18					
	25	5.33E-03	5.37E-03	5.35E-03	5					
	40	6.56E-04	9.20E-04	7.88E-04	1					98
Propranolol human	0	4.35E-02	4.53E-02	4.44E-02	100	0.963	0.011	61.4	27	100
	7	4.33E-02	4.58E-02	4.45E-02	100	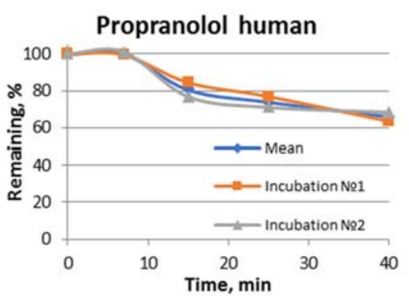				
	15	3.68E-02	3.49E-02	3.58E-02	81					
	25	3.35E-02	3.23E-02	3.29E-02	74					
	40	2.77E-02	3.11E-02	2.94E-02	66					85
EN300-123057 (Imatinib) human	0	5.40E+00	5.16E+00	5.28E+00	100	0.948	0.012	59.6	28	100
	7	5.88E+00	5.62E+00	5.75E+00	109	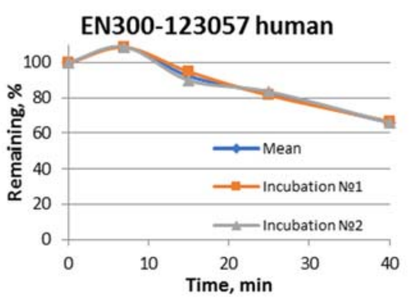				
	15	5.13E+00	4.64E+00	4.88E+00	93					
	25	4.40E+00	4.31E+00	4.36E+00	82					
	40	3.59E+00	3.41E+00	3.50E+00	66					88

**1**	**2**	**3**	**4**	**5**	**6**	**7**	**8**	**9**	**10**	**11**
EN300-37156110 (**85**)	0	2.09E+00	2.35E+00	2.22E+00	100	0.941	0.008	87.2	19	100
	7	2.33E+00	2.14E+00	2.24E+00	101	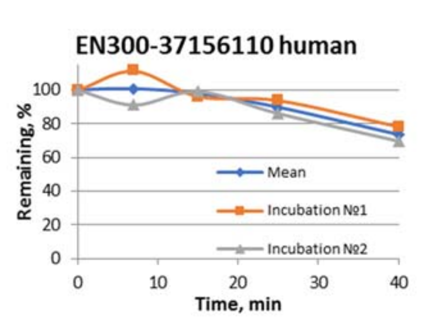				
	15	2.01E+00	2.33E+00	2.17E+00	98					
	25	1.96E+00	2.02E+00	1.99E+00	90					
	40	1.63E+00	1.63E+00	1.63E+00	73					118

Supplementary Tab. 4. Human microsomal stability (batch #2)

Compound ID	Time, min	Peak Area Ratio		Peak Area Ratio, Mean of 2	% Remaining, Mean of 2	R	k_{el}, min^{-1}	$t_{1/2}$, min	Cl_{int}, µl/min/mg	% Remaining without cofactor, Mean of 2
		Inc. 1	Inc. 2							
1	2	3	4	5	6	7	8	9	10	11
Diclofenac human	0	1.36E-01	1.43E-01	1.40E-01	100	0.999	0.119	5.8	286	100
	7	7.21E-02	6.45E-02	6.83E-02	49	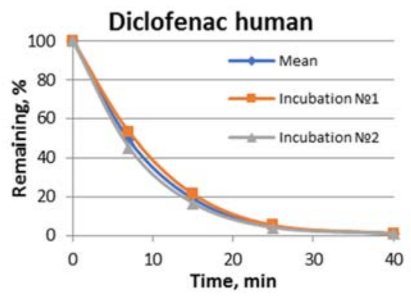				
	15	2.90E-02	2.37E-02	2.63E-02	19					
	25	7.24E-03	5.80E-03	6.52E-03	5					
	40	1.42E-03	1.24E-03	1.33E-03	1					91
Propranolol human	0	4.78E-02	4.59E-02	4.69E-02	100	0.975	0.008	82.5	20	100
	7	4.53E-02	4.74E-02	4.64E-02	99	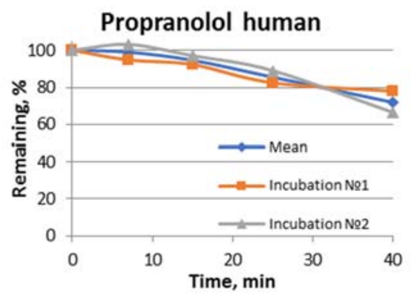				
	15	4.41E-02	4.46E-02	4.44E-02	95					
	25	3.94E-02	4.09E-02	4.01E-02	86					
	40	3.72E-02	3.05E-02	3.38E-02	72					97
EN300-37350388 **(86)** human	0	9.10E+00	7.58E+00	8.34E+00	100	0.915	0.006*	107.1*	16*	100
	7	8.65E+00	8.00E+00	8.32E+00	100	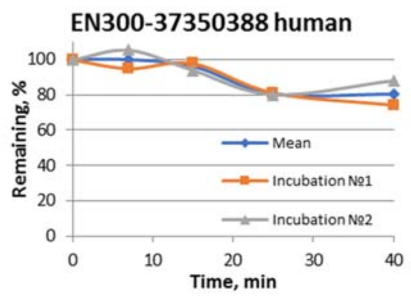				
	15	8.91E+00	7.12E+00	8.02E+00	96					
	25	7.40E+00	6.07E+00	6.74E+00	81					
	40	6.77E+00	6.68E+00	6.72E+00	81					106

*Parameter should be considered as approximate due to the high stability of the compound.

Interpretation of microsomal stability assay data

The test compounds can be classified in terms of their microsomal stability into low, medium and high clearance groups. The intrinsic clearance classification bands for mouse, rat, and human species are calculated according to the well stirred model equation:[1]

$$CL_{\text{int}} = CL_H / (f_u \times (1 - E))$$

where CL_H is a hepatic clearance (mL/min/kg), $CL_H = E \times Q_H$
Q_H = liver blood flow (mL/min/kg)[2]
E = extraction ratio, assumed at 0.3 for low clearance and at 0.7 for high clearance compounds
f_u = fraction unbound in plasma, assumed at 1.

The CL_{int} classification values were calculated for mouse, rat, and human species using the literature data on liver weight[3] and microsomal protein concentration[3,4] and are represented in the following table S5.

Supplementary Tab. 5. The intrinsic clearance groups for classification of test compounds

Classification group	Intrinsic clearance (µL/min/mg protein)		
	Mouse	Rat	Human
Low clearance	< 8.6	< 13	< 8.8
High clearance	> 48	> 72	> 48

Determination of pKa values

The tendency of a compound to donate proton is measured as its acid ionization constant (dissociation constant), or K_a. A more practical scale of representing acidity is pK_a which is the negative logarithm of the K_a (pK_a = -log Ka). pK_a of a test article is determined by pH-metric method based on potentiometric acid-base titration at 25 °C. The test compounds are dissolved in acidified methanol-water (1:4) solution of NaCl (150 mM, pH 2) and slowly titrated with 0.3 M sodium hydroxide methanol-water (1:4) solution, while recording pH of the solution as a function of NaOH volume used during the titration (construction of the titration curve). Titration of acidified NaCl solution in absence of any compounds is used for blank plotting.[5-8]

Buffering capacity is calculated in each point of titration curve as the ratio of the NaOH flow (constant) to the pH rise velocity. The pK_a value is determined from resulting plot of buffering capacity versus pH as the maximum of buffering capacity. pH-metric method allows to measure pK_as in the range of approximately between 2 and 12.

Acquisition and analysis of the data were performed using SmartLogger II 1.0.14 software (pH-meter, pHi®510 (Beckman Coulter, Canada; Cat# A58734). Data analysis was done using GraphPad Prism 6.01 software.

Reagents and consumables

Sodium hydroxide (Enamine, Ukraine; CAS # 1310-73-2)
Sodium chloride BioXtra, ≥99.5% (AT) (Sigma-Aldrich, USA; Cat # S7653)
Hydrochloric acid (Enamine, Ukraine; CAS # 7647-01-0)
Disposable pipette tips (Eppendorf, German; Matrix and Finntip, Thermo, USA)
Polypropylene graduated conical tubes, 50 mL (Kartell, Italy; Cat. # 84002)
Polypropylene syringe (10 mL) with tubing (Hemoplast, Ukraine)

Equipment

pH-meter, pHi®510 (Beckman Coulter, Canada; Cat# A58734)
Multichannel Electronic Pipettes 5-50 µL, 10-1000 µL (Thermo, USA)
Magnetic stirrer standard unit (IKA, USA)
Syringe Driver Mdl 100 (KDScientific, USA)

Supplementary Tab. 6. Experimental pK_a values of carboxylic acids.

Compound	pKa (exp.)
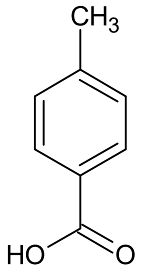	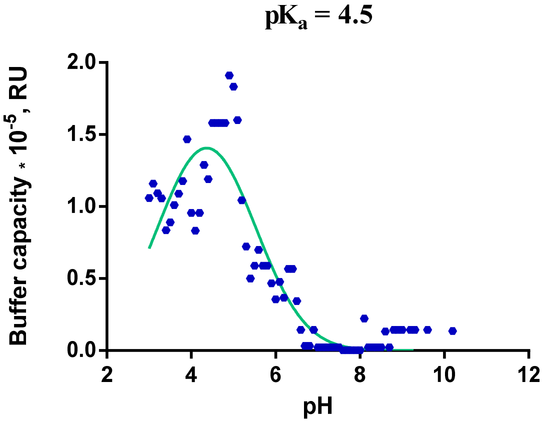
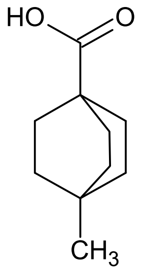	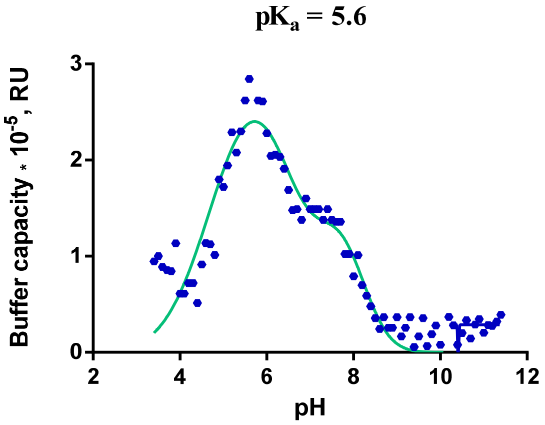
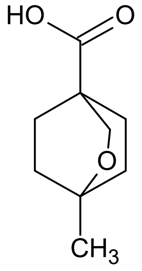	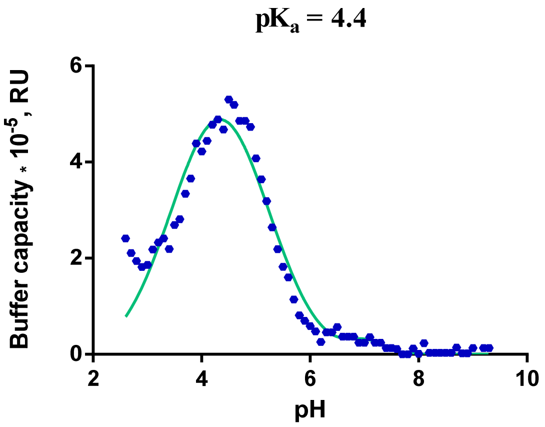

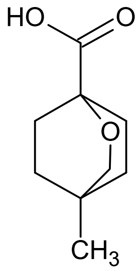	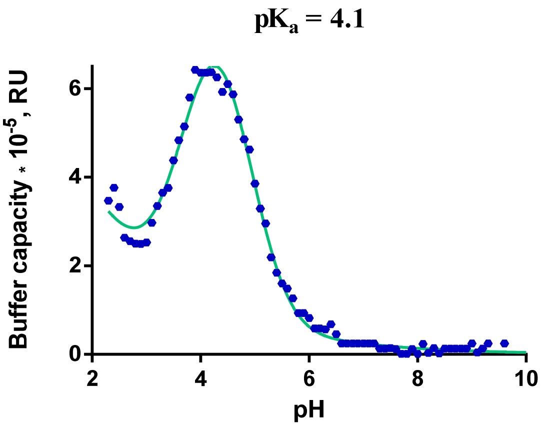

Data should be considered as approximate due to specific physicochemical properties of the compound, apparently compound forms colloids in the titration media. The pK_a values of reference compounds are consistent with published data, thus validating this study.

Biological activity of *Imatinib* and its analogues 85, 86. ABL1 Kinase Assay

The ADP-Glo™ Kinase Assay kit (Promega) was used to evaluate the inhibitory effect of studied compounds (Staurosporine, Imatinib, compound **85** and compound **86**) on the catalytic activity of ABL1 kinase. The luminescent signal generated by the assay system positively correlates with kinase activity, allowing to study inhibition of research compounds. For the initial assay conditions, we used the manufacturer's protocol (https://www.promega.jp/-/media/files/resources/protocols/kinase-enzyme-appnotes/ABL1-kinase-assay-protocol.pdf), particularly the reaction buffer formulation (40mM Tris-HCl (pH 7.5), 20mM $MgCl_2$, 50µM DTT, 0.1 mg/mL BSA) and the duration of the reaction incubations (60 min, 40 min and 30 min).

At the first stage of the study, we determined the Michaelis kinase constant for ATP and the optimal kinase concentration (1.5 nM), corresponding to an S/B ratio of about 20 and a substrate conversion corresponding 10%. Then we studied the influence of four compounds (*Staurosporine, Imatinib*, compound **85**, compound **86**) at 12 concentration points on the ABL1 catalytic activity in three independent experiments, each in four replicates. An expected inhibitory effect of *Imatinib* and *Staurosporine* on the target kinase was confirmed, and the half maximal inhibitory concentration obtained for *Staurosporine* (318 nM) (Figure S10) corresponds well to the data of the assay manufacturer (415 nM).[9] However, we did not observe any significant inhibitory effect on ABL1 of compound **85** and compound **86** (Supplementary Fig. 14, Supplementary Fig. 15).

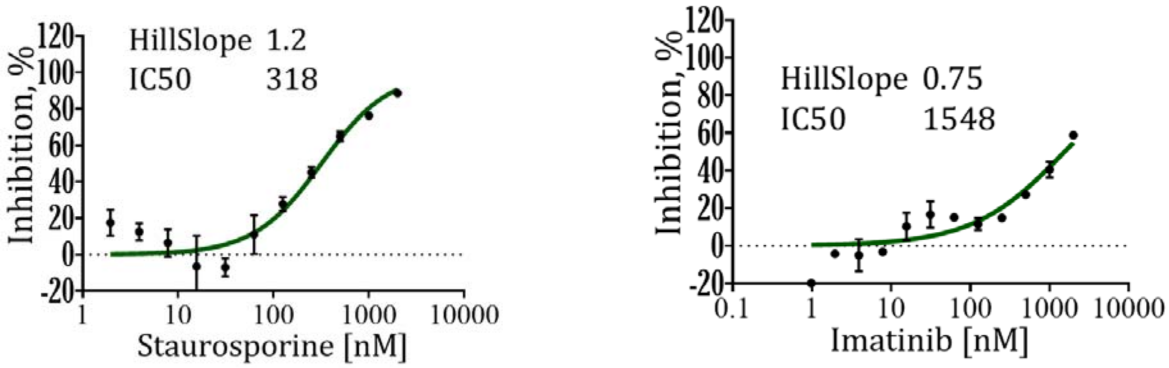

Supplementary Fig. 13. Dose-dependence curves illustrating the inhibitory effect of *Staurosporine* (n=4 technical replicates) and *Imatinib* (n=2 technical replicates) on the ABL1 catalytic activity. Data are presented as mean values +/- SD.

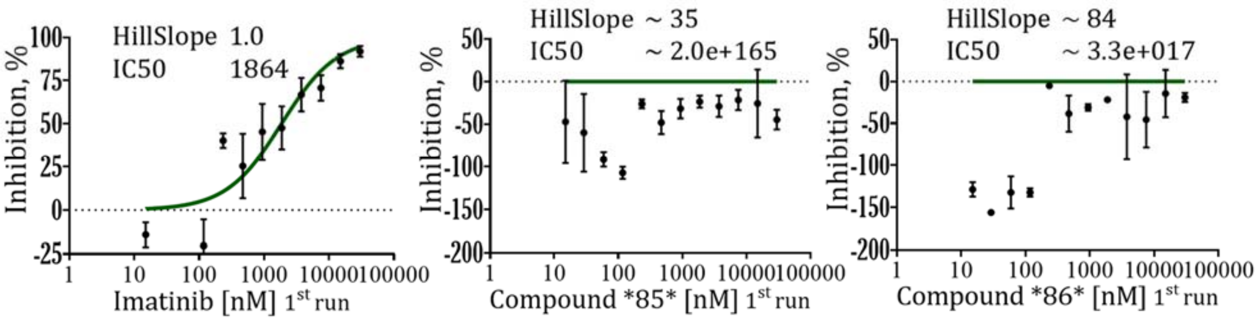

Supplementary Fig. 14. Dose-response curves illustrating the inhibitory effect of *Imatinib* (n=4 technical replicates), compound **85** (n=4 technical replicates), and compound **86** (n=2 technical replicates) on the ABL1 catalytic activity obtained in the first run. Data are presented as mean values +/- SD.

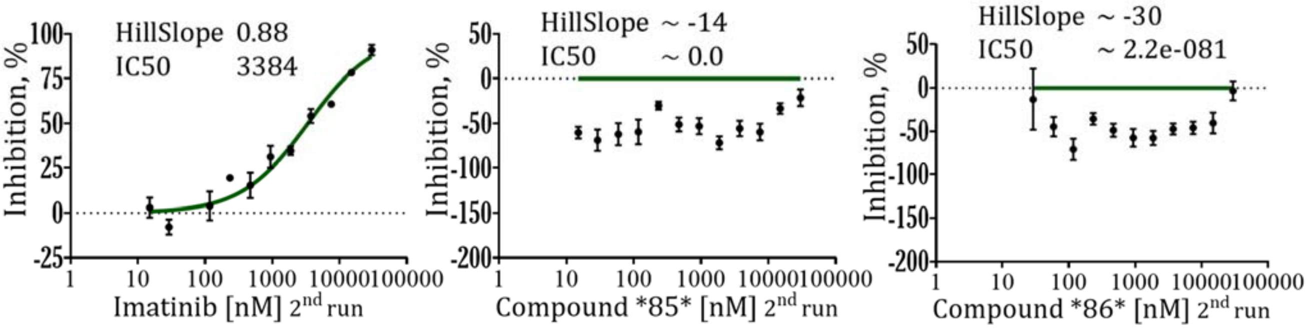

Supplementary Fig. 15. Dose-response curves illustrating the inhibitory effect of *Imatinib* (n=4 technical replicates), compound **85** (n=4 technical replicates), and compound **86** (n=4 technical replicates) on the ABL1 catalytic activity obtained in the second run. Data are presented as mean values +/- SD.

Biological activity of Vorinostat and its analogues 88, 89

Study objective

The aim of the study was to compare the effect of *Vorinostat* and compounds **88**, **89** on cell proliferation and cell death mechanisms, using High Content Imaging assay. The following parameters were assessed: nuclei count to measure inhibiting of the proliferation; cell membrane permeability to assess general cytotoxicity and necrosis; caspase activation to test apoptosis.

Reference compound MOA

Vorinostat is the first histone deacetylase inhibitor approved for oncology therapeutic area. Its anti-tumor action is caused by chromatin decondensation, which leads to the activation of certain genes. Additionally *Vorinostat* influences other epigenetic events, such as protein methylation and acetylation as well as microRNA function. In such way it regulates activity of transcriptional factors, in particular, proteins involved in cell growth and apoptosis. Thereby caspase-dependent apoptosis, autophagy and cell cycle arrest caused by *Vorinostat* determine its activity as a potent chemotherapeutic agent.[10,11]

Methods

Human hepatocellular carcinoma HepG2 cell line was used to test biological action of Vorinostat and its analogues. Cell line was obtained from Leibniz Institute DSMZ - German Collection of Microorganisms and Cell Cultures. Cells were cultured in DMEM medium (Hyclone, Cat# SH30003.04) supplemented with 10% fetal bovine serum (Sigma-Aldrich, Cat# F7524), 100 units/mL penicillin and 100 µg/mL streptomycin (Sigma-Aldrich, Cat# P4333). Before plating, the cells were first washed with PBS and then trypsinized with 0.01% trypsin solution (Sigma-Aldrich, Cat# T4174) in DPBS. The appropriate volume of culture medium was placed in a flask to stop further trypsinization, then the cells were centrifugated at 200 x g for 4 min, resuspended in fresh complete medium and counted using a counting chamber after being stained with Trypan Blue.

Cells were seeded in black 384-well plate (Perkin Elmer Product, Cat# 6057300), 4000 cells in 30 µL total volume per well and incubated at 37ºC/5%CO_2/95%RH overnight. *Vorinostat* and test compounds were dissolved in 100% DMSO at a concentration of 10 mM and stored at -20 °C as single-use aliquots. Next day 20 µL of medium with compounds was added to the cells to the final DMSO concentration 0.5. Treatment for 48 hours was performed under growth conditions. Compounds were assessed at the final concentrations 1, 5 and 50 µM (each in triplicates - 3 wells, 9 fields of view per well). Staining for nuclei, apoptotic cells with activated caspase 3/7 and dead

cells with permeabilized membrane was performed for 30 min under growth conditions in staining solution containing 5 µM Hoechst 33342 (Thermo Scientific, Cat# 62249), 5 µM CellEvent Caspase 3/7 Detection Reagent (Invitrogen, Cat# C10423) and 1 µM Propidium Iodide (Invitrogen, Cat# P3566) in medium complete. The cells were imaged with InCell Analyzer 6500, 20x obj. Image analysis was conducted using INCarta Software 1.13. Data were analyzed in GraphPad Prism 9.

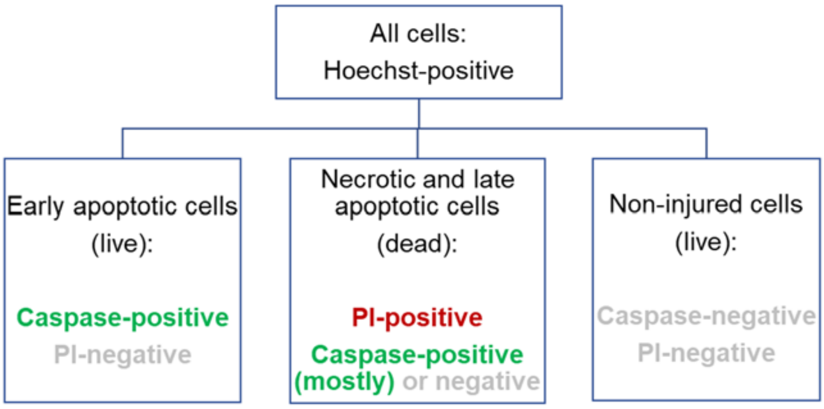

Supplementary Fig. 16. Workflow of multiplexed cells staining.

Multiplexed staining allows to test cell toxicity and distinguish between necrosis and apoptosis within one single experiment (Supplementary Fig. 16). Hoechst 33342 dye labels all cell nuclei. CellEvent Caspase-3/7 Green Detection Reagent labels cells with activated caspases. Propidium Iodide (PI) labels dead cells with permeabilized membrane. Hoechst-positive cells were counted to assess the % of cell toxicity. PI-positive cells are dead – this is a fraction of necrotic and late apoptotic cells, used for assessing of cytotoxicity/necrosis. Early apoptotic cells are caspase-positive, PI-negative because their membrane isn't compromised yet. Percentages of cells of certain type were obtained through direct calculation of cells exhibiting fluorescence intensities higher than background and comparing with overall number of cells.

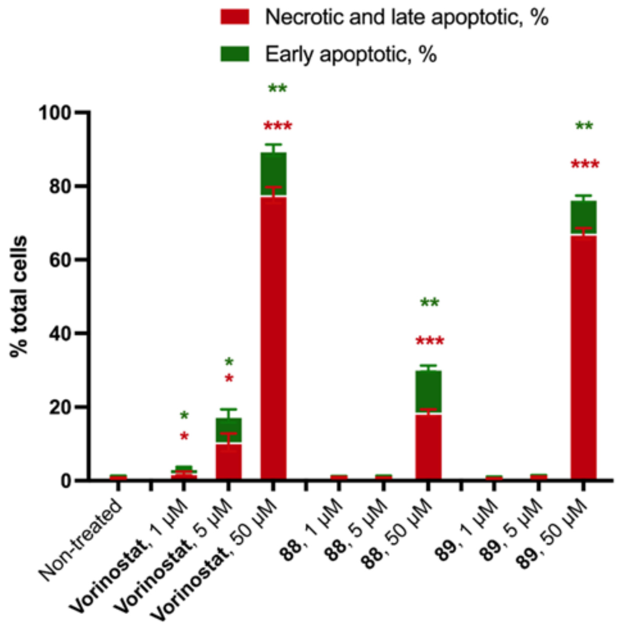

Supplementary Fig. 17. Types of HepG2 cells death (% of total cells) after treatment with *Vorinostat* and compounds **88**, **89** (1 µM, 5 µM and 50 µM) for 48h. Red: necrotic cell death. Green: early apoptotic cell death. The data were presented as mean ± SEM (n = 3, independent wells for every of which approx. 2000 visualised cells were analyzed).* - indicates P < 0.05, ** - indicates P < 0.01, *** - P < 0.001 compared with the non-treated group in two-tailed unpaired t test with Welch correction on each row of data.

Results

Cytotoxic and antiproliferative effects of compounds **88**, **89** and *Vorinostat* as a reference compound on the HepG2 cells are shown in the Supplementary Fig. 17, Supplementary Fig. 18.

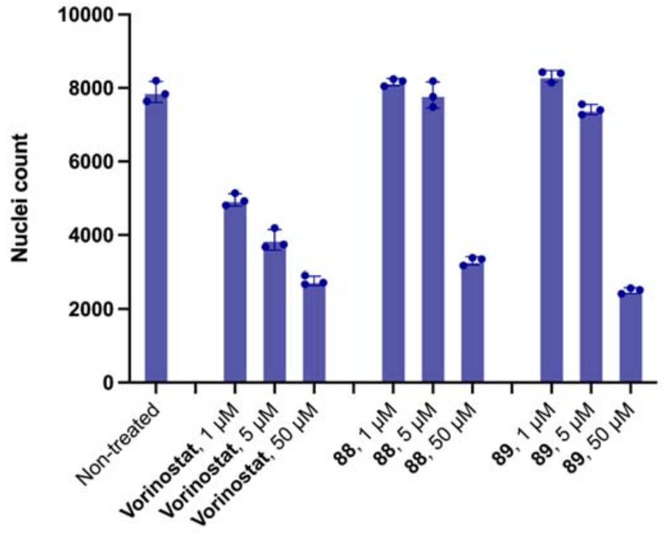

Supplementary Fig. 18. Nuclei count upon HepG2 cells treatment for 48 hours with *Vorinostat* and **88**, **89** at 1, 5 and 50 µM concentrations. The data were presented as mean ± SEM (n = 3, independent wells for every of which approx. 2000 visualised cells were analyzed).

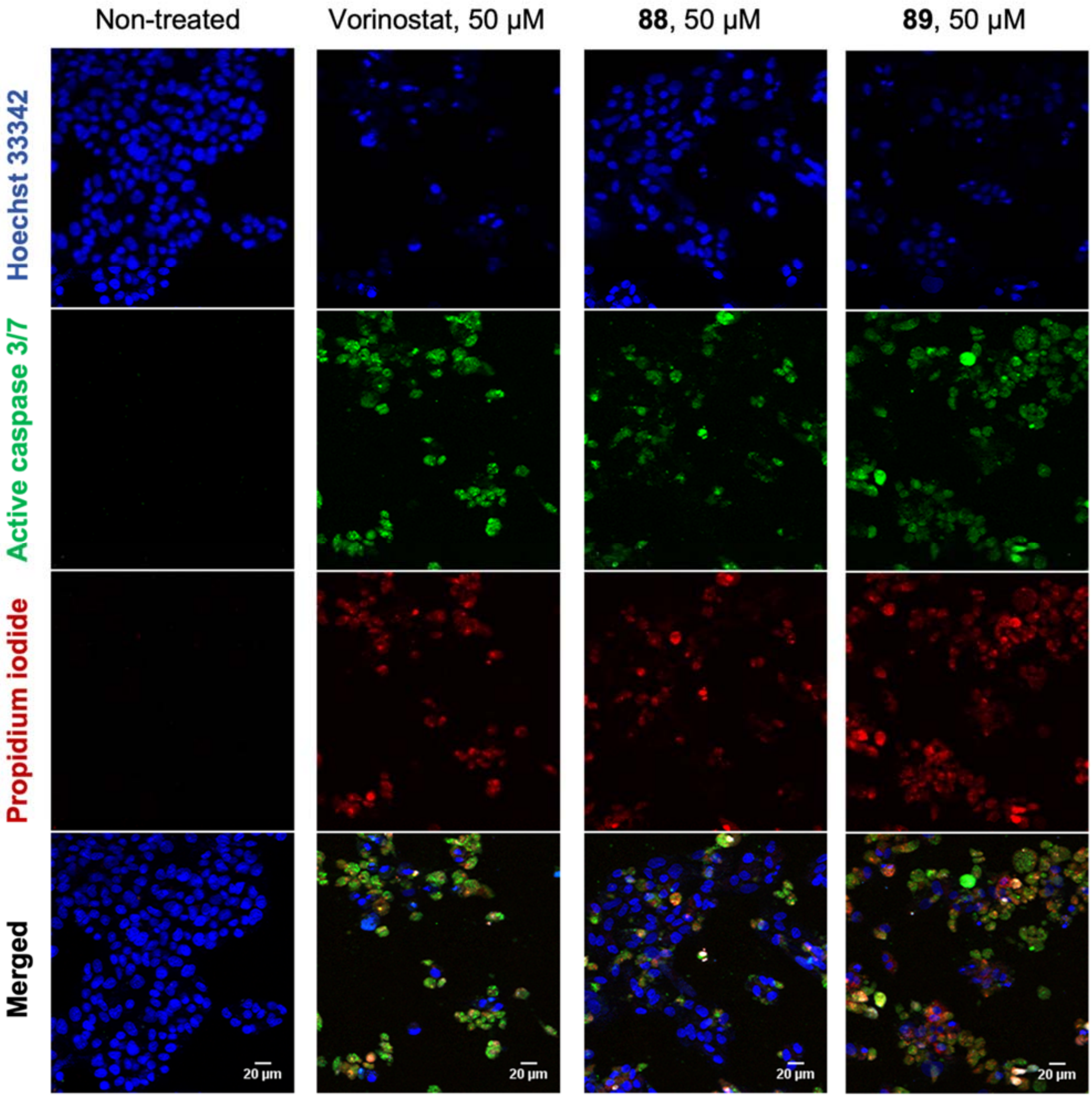

Supplementary Fig. 19. Fluorescent microscopy images of HepG2 cells incubated with *Vorinostat* and compounds **88**, **89** (50 μM) during 48 h. 1st row: nuclei of cells are marked by Hoechst 33342 (blue). 2nd row: apoptotic cells are marked by CellEvent Caspase-3/7 Green Detection Reagent (green). 3rd row: necrotic cells are marked by propidium iodide (red). 4th row: superposition of all three staining above. The experiment was conducted once.

Supplementary Tab. 7. Effect of *Vorinostat* and **88**, **89** on apoptosis regulation. Two-tailed unpaired t tests with Welch correction on each row of data were used to compare means for every experimental condition with non-treated control mean.

		% of PI positive (necrotic) cells					% of PI negative, caspase positive (early apoptotic) cells				
Compound	Concen-tration	% of necrotic and late apoptotic cells (PI positive)	p value (compari-son with control)		p value (comparison with vorinostat at corresponding concentration)		% of early apoptotic cells (PI negative, caspase positive)	p value (compari-son with control)		p value (comparison with vorinostat at corresponding concentration)	
Non-treated control	0	0.7					0.5				
Vorinostat	1 uM	2.0	0.0340	*			1.4	0.0158	*		
	5 uM	10.4	0.0196	*			7.2	0.0227	*		
	50 uM	77.5	0.0002	***			12.2	0.0056	**		
89	1 uM	0.6	0.3642		0.0338	*	0.4	0.8682		0.0242	*
	5 uM	0.9	0.3705		0.0200	*	0.6	0.4273		0.0240	*
	50 uM	67.1	0.0002	***	0.0034	**	9.5	0.0023	**	0.0794	
88	1 uM	0.9	0.2730		0.0452	*	0.3	0.3408		0.0219	*
	5 uM	0.8	0.4453		0.0203	*	0.5	0.9942		0.0233	*
	50 uM	18.5	0.0007	***	0.0001	***	12.0	0.0012	**	0.8365	

Vorinostat acts as a chemotherapeutic agent preventing cell proliferation. It induces caspase-dependent cell death (apoptosis) upon certain genes activation. *Vorinostat* treatment leads to decrease of cell-count and increases caspases activation. Both tested compounds **88** and **89** inhibited cell proliferation at 50 µM concentration to the same level as *Vorinostat*, but they did not have any significant effects at 1 and 5 µM concentrations unlike *Vorinostat* (Supplementary Tab. 7). **89** activity profile was the closest to V*orinostat*, demonstrating similar levels of cell toxicity and apoptosis/necrosis ratio. All tested compounds were able to activate caspases and induce apoptosis at 50 µM concentration. Compounds **88** and **89** did not demonstrate any significant cytotoxic effects at 1 and 5 µM concentrations. At 50 µM concentration *Vorinostat* cell toxicity level was 77%, **89** – 67% and **88** – 18%, correspondingly.

Cell toxicity effects, speed and mode of action observed upon treatment with compounds **88** and **89** are promising though they do not completely coincide with *Vorinostat*.

Generation and properties of virtual libraries based on *para*-aminobenzoic acid and 2-oxabicyclo[2.2.2]octane analog

Two virtual libraries based on *para*-aminobenzoic acid (**Lib-1**) and 2-oxabicyclo[2.2.2]octane (**Lib-2**) have been constructed (Supplementary Tab. 8).

1) For that we used Enamine's Building Blocks collection.[12] This collection comprises 39576 of primary amines, 27154 secondary amines and 39923 carboxylic acids. We have randomly selected 1000 amines (primary and secondary) and 1000 carboxylic acids for decoration of *para*-aminobenzoic acid and 2-oxabicyclo[2.2.2]octane analog.

2) We used amide synthesis reaction to decorate *para*-aminobenzoic acid at *N*-terminus (with 1000 acids) and *C*-terminus (with 1000 amines). A virtual library of 1.000.000 molecules was obtained. Analogous modifications were also performed with 2-oxabicyclo[2.2.2]octane analog.

SMARTS:

- Reaction SMARTS for **Lib-1**:
  '[C:1][NH:2].[OH:3][C:4](=[O:5])[c:6]1[c:7][c:8][c:9]([c:10][c:11]1)[NH2:12].[OH:13][C:14](=[O:15])>>[C:1][N:2][C:4](=[O:5])[c:6]1[c:7][c:8][c:9]([c:10][c:11]1)[NH:12][C:14](=[O:15]).[OH2:3].[OH2:13]'

- Reaction SMARTS for **Lib-2**:
  '[C:1][NH:2].[OH:3][C:4](=[O:5])[C:6]12[C:7][C:8][C:9]([C:10][C:11]1)([C:12][O:13]2)[NH2:14].[OH:15][C:16](=[O:17])>>[C:1][N:2][C:4](=[O:5])[C:6]12[C:7][C:8][C:9]([C:10][C:11]1)([C:12][O:13]2)[NH:14][C:16](=[O:17]).[OH2:3].[OH2:15]'

3) To minimize the influence of rotational degree of freedom in both molecular sets, we have further considered only the molecules with less than five (less or equal to 4) rotational bonds. This restriction led to 1.000.000 -> 76.588 molecules in each library.

4) To make these two libraries visually comparable via PMI-3D plots, we have reduced both libraries to 5000 molecules in each set via random sampling. For the sake of consistent comparison between **Lib-1** and **Lib-2**, we have preserved the same pairs of amine- and carboxylic acid-decorators in both libraries (Supplementary Tab. 8).

5) The ultimate sets of molecules were then subjected to an extensive conformational search procedure resulting in a set of lowest energy conformers for every molecular structure.[13] Only one lowest energy conformer per structure has been stored and used in PMI-3D plots. Corresponding PMI-3D calculations were carried out and then plotted (Supplementary Fig. 20, Supplementary Fig. 21) together with manually selected five known FDA-approved drugs, - Aminopterin, Conivaptan, Mitapivat, Deferasifox, Tetracaine, and their 2-oxabicyclo[2.2.2]octane-containing analogs.[14]

Both libraries, **Lib-1** and **Lib-2** occupy the same region of 3D-chemical space (Supplementary Fig. 20, Supplementary Fig. 21). The same is true for 4 drugs - Aminopterin, Conivaptan, Deferasifox, Tetracaine (Supplementary Fig. 20), - and their 2-oxabicyclo[2.2.2]octane-containing analogs (Supplementary Fig. 21). Only, the lowest-energy conformers of Mitapivat drug and its 2-oxabicyclo[2.2.2]octane-containing analog occupied different positions at PMI-plots (Supplementary Fig. 20, (Supplementary Fig. 21) .

At the same time, both libraries had different properties. The number of natural product-like compounds in virtual library based on *para*-aminobenzoic acid (**Lib-1**) was only 8, while the corresponding number of compounds in 2-oxabicyclo[2.2.2]octane-containing library was 495. Mean F(sp^3) index for **Lib-1** was 0.38, while that for **Lib-2** was already 0.64. Mean cLogP was for **Lib-1** was 4.0, while that one for **Lib-2** was more than one cLogP unit smaller – 2.9.

Supplementary Tab. 8. Properties of virtual libraries **Lib-1** (based on *para*-aminobenzoic acid) and **Lib-2** (based on 2-oxabicyclo[2.2.2]octane).

Virtual Library	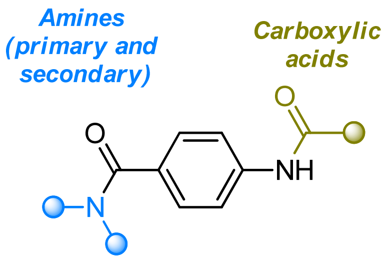 *para*-aminobenzoic acid **Lib-1**	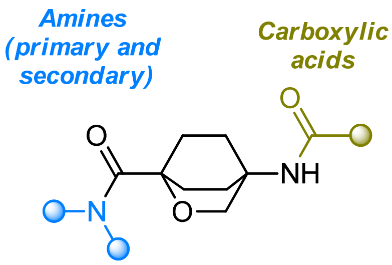 2-Oxabicyclo[2.2.2]octane **Lib-2**
Number of compounds	5000	5000
Number of natural product-like compounds	8	495
F(sp^3) (min/max/mean)	0.03/0.73/0.38	0.25/0.93/0.64
cLogP (min/max/mean)	-1.3/10.0/4.0	-2.3/8.9/2.9
TPSA (min/max/mean)	49/200/91	59/209/100

Supplementary Fig. 20. PMI plot of library **Lib-1** (based on *para*-aminobenzoic acid). Position of five FDA-approved drugs, - Aminopterin, Conivaptan, Mitapivat, Deferasifox, Tetracaine, - is shown on the plot with blue dots.

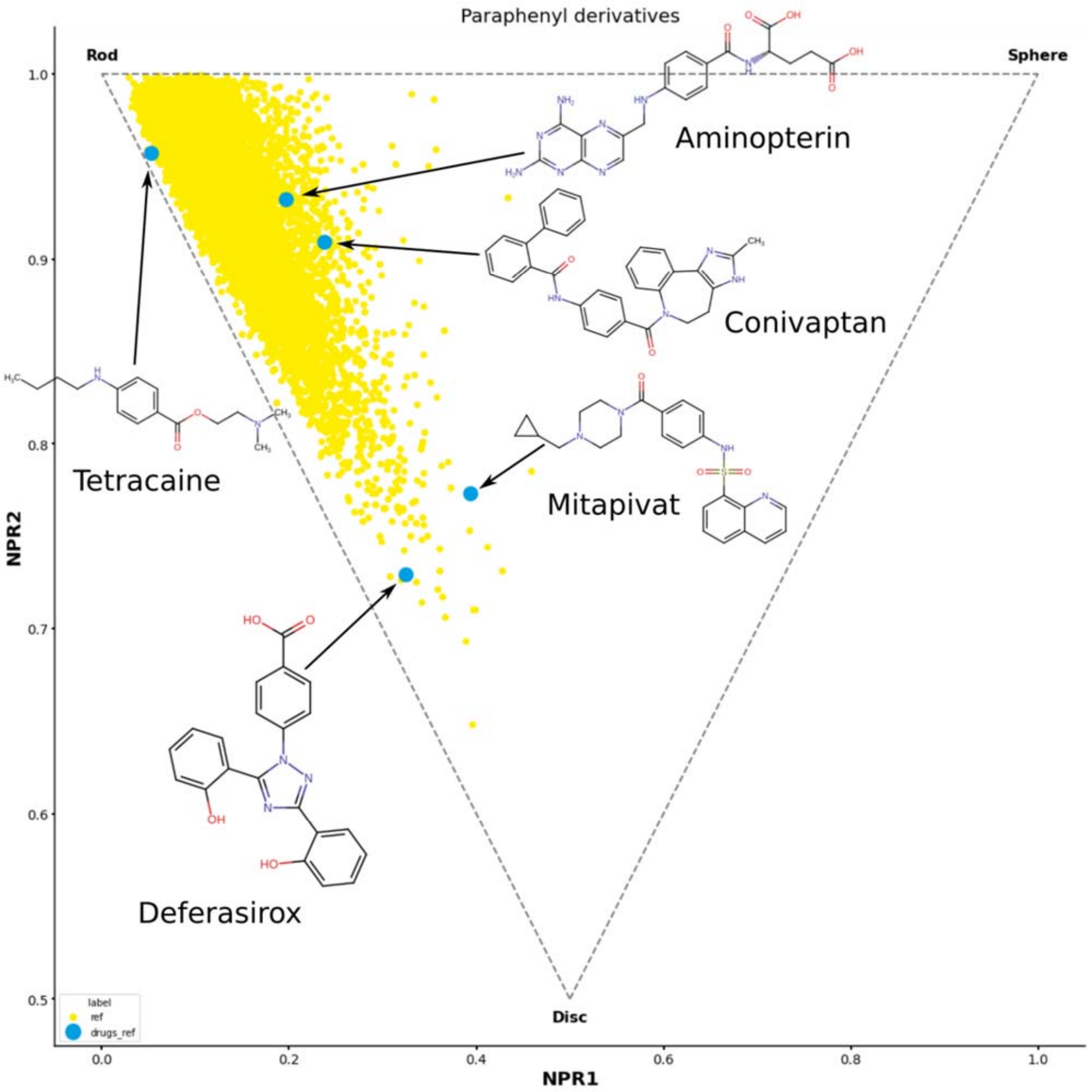

Supplementary Fig. 21. PMI plot of library **Lib-2** (based on 2-oxabicyclo[2.2.2]octane). Position 2-oxabicyclo[2.2.2]octane-containing analogs of drugs Aminopterin, Conivaptan, Mitapivat, Deferasifox, Tetracaine, is shown on the plot with blue dots.

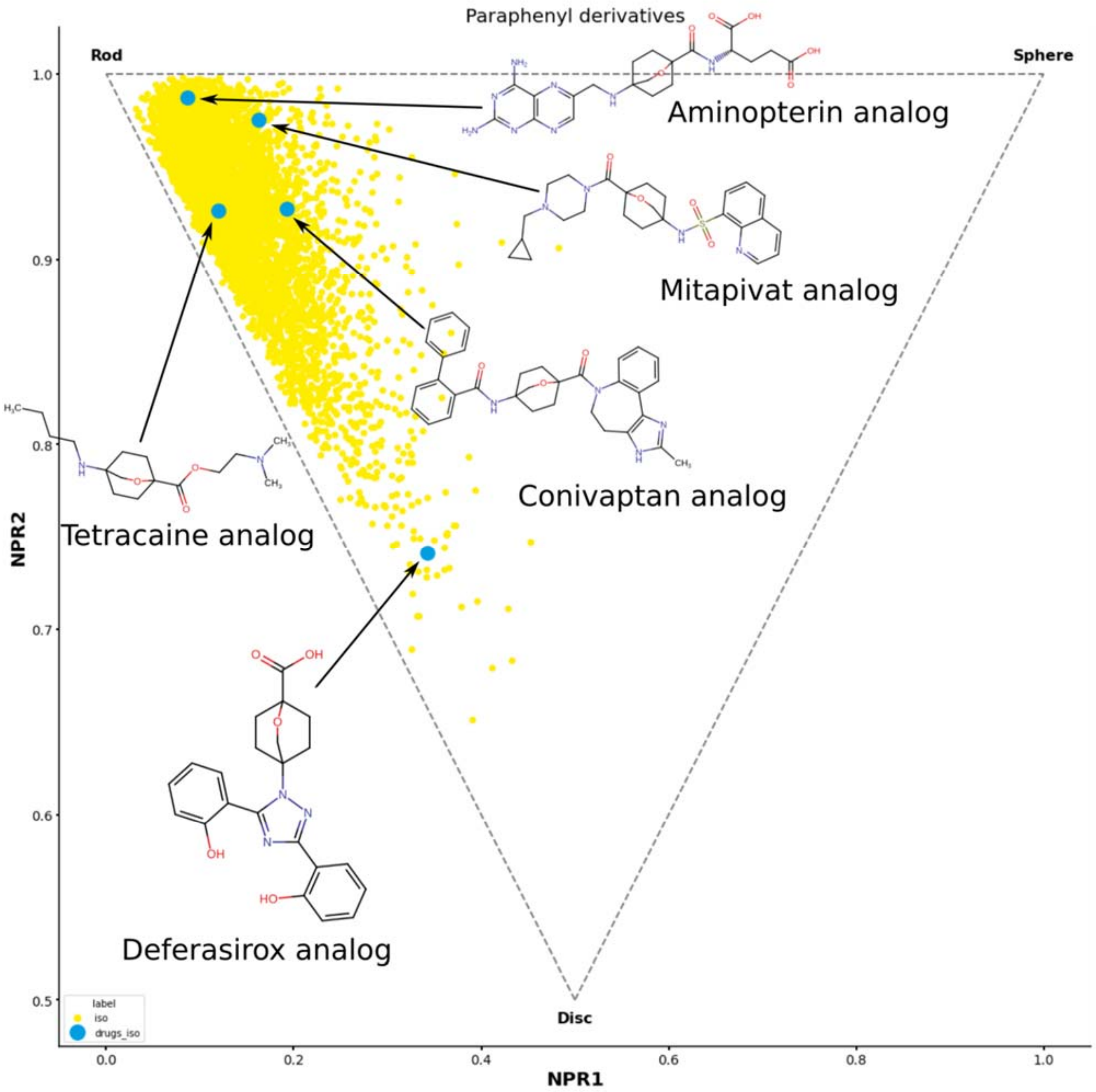

Supplementary references

1. Houston J. B. Utility of *in vitro* drug metabolism data in predicting *in vivo* metabolic clearance. *Biochem. Pharmacol.* **47**, 1469-1479 (1994).

2. Davies, B., Morris, T. Physiological parameters in laboratory animals and humans. *Pharm. Res.* **10**, 1093-1095 (1993).

3. Barter, Z. E. et al. Scaling factors for the extrapolation of *in vivo* metabolic drug clearance from *in vitro* data: reaching a consensus on values of human microsomal protein and hepatocellularity per gram of liver. *Curr. Drug Metab.* **8**, 33-45 (2007).

4. Iwatsubo, T. et al. Prediction of species differences (rats, dogs, humans) in the *in vivo* metabolic clearance of YM796 by the liver from *in vitro* data. *J. Pharmacol. Exp. Ther*. **283**, 462-469 (1997).

5. http://www.sirius-analytical.com/science/pka/methods-measuring-pka

6. http://www.mhra.gov.uk/home/groups/par/documents/websiteresources/con2023944.pdf

7. http://www.chem.wisc.edu/areas/reich/pkatable/pKa_compilation-1-Williams.pdf

8. http://www.jbc.org/content/218/2/961.full.pdf

9. https://www.promega.jp/-/media/files/resources/protocols/kinase-enzyme-appnotes/ABL1-kinase-assay-protocol.pdf

10. Richon, V. M. Targeting histone deacetylases: development of vorinostat for the treatment of cancer. *Epigenomics* **2**, 457-465 (2010).

11. Lee, J., R, S. H. Cancer Epigenetics: Mechanisms and Crosstalk of a HDAC Inhibitor, Vorinostat. Chemotherapy (Los Angel). **2**, 14934 (2013). doi: 10.4172/2167-7700.1000111.

12. https://enamine.net/building-blocks/building-blocks-catalog (in stock BB).

13. The conformational search procedure was realized using ICM-3.8.7c13 (MolSoft LLC, www.molsoft.com).

14. The calculations have been performed using RDKit, Seaborn and Matplotlib libraries for Python 3.9.
