# Supplementary material for: 2-Oxabicyclo[2.2.2]octane as a new bioisostere of the phenyl ring
Source: Nat Commun. 2023 Oct 2;14:5608. doi: 10.1038/s41467-023-41298-3 (PMC10545790; doi:10.1038/s41467-023-41298-3)
Supplement: Supplementary file 3 — Description of Additional Supplementary Files [file 41467_2023_41298_MOESM3_ESM.pdf]

Description of Additional Supplementary Files

File Name: Supplementary Data 1
Description: Crystallographic data for compound **30**; CCDC reference 2226162.

File Name: Supplementary Data 2
Description: Crystallographic data for compound **57**; CCDC reference 2226164.

File Name: Supplementary Data 3
Description: Crystallographic data for compound **67**; CCDC reference 2226872.

File Name: Supplementary Data 4
Description: Crystallographic data for compound **69**; CCDC reference 2226163.

File Name: Supplementary Data 5
Description: Crystallographic data for compound **78**; CCDC reference 2266656.

File Name: Supplementary Data 6
Description: Virtual library (Lib-1, 5000 molecules) based on paraaminobenzoic acid.

File Name: Supplementary Data 7
Description: Virtual library (Lib-2, 5000 molecules) based on 2- oxabicyclo[2.2.2]octane.
