# Supplementary material for: 2-Oxabicyclo[2.2.2]octane as a new bioisostere of the phenyl ring
Source: Nat Commun. 2023 Oct 2;14:5608. doi: 10.1038/s41467-023-41298-3 (PMC10545790; doi:10.1038/s41467-023-41298-3)
Supplement: Supplementary file 11 — Reporting Summary [file 41467_2023_41298_MOESM11_ESM.pdf]

nature research

Corresponding author(s): Pavel K. Mykhailiuk

Last updated by author(s): Aug 15, 2023

Reporting Summary

Nature Research wishes to improve the reproducibility of the work that we publish. This form provides structure for consistency and transparency in reporting. For further information on Nature Research policies, see our Editorial Policies and the Editorial Policy Checklist.

Statistics

For all statistical analyses, confirm that the following items are present in the figure legend, table legend, main text, or Methods section.

n/a	Confirmed	
☐	☒	The exact sample size (*n*) for each experimental group/condition, given as a discrete number and unit of measurement
☐	☒	A statement on whether measurements were taken from distinct samples or whether the same sample was measured repeatedly
☐	☒	The statistical test(s) used AND whether they are one- or two-sided<br>*Only common tests should be described solely by name; describe more complex techniques in the Methods section.*
☒	☐	A description of all covariates tested
☒	☐	A description of any assumptions or corrections, such as tests of normality and adjustment for multiple comparisons
☐	☒	A full description of the statistical parameters including central tendency (e.g. means) or other basic estimates (e.g. regression coefficient) AND variation (e.g. standard deviation) or associated estimates of uncertainty (e.g. confidence intervals)
☐	☒	For null hypothesis testing, the test statistic (e.g. *F*, *t*, *r*) with confidence intervals, effect sizes, degrees of freedom and *P* value noted<br>*Give P values as exact values whenever suitable.*
☒	☐	For Bayesian analysis, information on the choice of priors and Markov chain Monte Carlo settings
☒	☐	For hierarchical and complex designs, identification of the appropriate level for tests and full reporting of outcomes
☒	☐	Estimates of effect sizes (e.g. Cohen's *d*, Pearson's *r*), indicating how they were calculated

Our web collection on statistics for biologists contains articles on many of the points above.

Software and code

Policy information about availability of computer code

Data collection	Product purification was performed using HPLC AGILENT 1260 INFINITY (a column Chromatorex C18 SMB 100-5T, 100*19 mm, 5 microm) or PuriFlash XS420 Plus. The NMR data acquisition was performed using Varian UNITY III 400; Varian VNMRS 500; Bruker AVANCE DRX 500 and Bruker AVANCE III 400 spectrometers. HRMS data acquisition was performed using Agilent 6224 TOF LC/MS. Lipophilicity (clogP) was calculated with “Cxcalc” ChemAxon, version 22.5.0.<br>Microscopy data were acquired using InCell Analyzer 6500HS (Cytiva)
Data analysis	The NMR data analysis was performed using Mestrenova software (11.0.3-18688). The data acquisition and system control was performed using Analyst 1.6.3 software from AB Sciex.<br>Microscopy images analysis was performed with InCarta 1.13 (Cytiva).<br>Data analysis of biochemical and cell based experiments was performed using GraphPad Prism 9.

For manuscripts utilizing custom algorithms or software that are central to the research but not yet described in published literature, software must be made available to editors and reviewers. We strongly encourage code deposition in a community repository (e.g. GitHub). See the Nature Research guidelines for submitting code & software for further information.

Data

Policy information about availability of data

All manuscripts must include a data availability statement. This statement should provide the following information, where applicable:

- Accession codes, unique identifiers, or web links for publicly available datasets
- A list of figures that have associated raw data
- A description of any restrictions on data availability

Experimental data as well as characterization data for all new compounds prepared during these studies are provided in the Supplementary Information of this

manuscript. The X-ray crystallographic coordinates for compounds 30, 57, 67, 69, and 78 have been deposited at the Cambridge Crystallographic Data Centre (CCDC) with accession codes 2226162 (30), 2226164 (57), 2226872 (67), 2226163 (69), 2266656 (78). These data can be obtained free of charge from the Cambridge Crystallographic Data Centre via www.ccdc.cam.ac.uk/structures/. A source data file is available for biological activity of Imatinib with analogs 85, 86; and Vorinostat with analogs 88, 89.

Field-specific reporting

Please select the one below that is the best fit for your research. If you are not sure, read the appropriate sections before making your selection.

- [x] Life sciences
- [ ] Behavioural & social sciences
- [ ] Ecological, evolutionary & environmental sciences

For a reference copy of the document with all sections, see nature.com/documents/nr-reporting-summary-flat.pdf

Life sciences study design

All studies must disclose on these points even when the disclosure is negative.

Sample size	Cell-based assays: n = 3, independent wells, for every of which approx. 2000 visualised cells were analyzed.
Data exclusions	No data were excluded from the analysis.
Replication	3 replicates across independent wells.
Randomization	The completely randomized design (CRD) was used for all in vitro experiments. It is appropriate in situations in which the material is genetically and physiologically homogeneous (stable cell culture and recombinant protein), and all assays had been performed within a corresponding single assay plate for each type of experiment.
Blinding	Compared items and reference compund(s) are provided to the researcher in 96- or 384- well plates by separate department. Plate maps are not available for the researcher.

Reporting for specific materials, systems and methods

We require information from authors about some types of materials, experimental systems and methods used in many studies. Here, indicate whether each material, system or method listed is relevant to your study. If you are not sure if a list item applies to your research, read the appropriate section before selecting a response.

Materials & experimental systems

n/a	Involved in the study	
x		Antibodies
	x	Eukaryotic cell lines
x		Palaeontology and archaeology
x		Animals and other organisms
x		Human research participants
x		Clinical data
x		Dual use research of concern

Methods

n/a	Involved in the study	
x		ChIP-seq
x		Flow cytometry
x		MRI-based neuroimaging

Eukaryotic cell lines

Policy information about cell lines

Cell line source(s)	DSMZ: ACC-180<br>https://celldive.dsmz.de/cellline/ACC-180
Authentication	The cell line was not authenticated, as obtained from the commercial source.
Mycoplasma contamination	All cells tested negative for mycoplasma contamination by MycoSEQ™ Mycoplasma Detection Kits<br>Applied Biosystems™<br>Catalog number: 4460626
Commonly misidentified lines<br>(See ICLAC register)	n/a
